# Ni-catalyzed hydroalkylation of olefins with N-sulfonyl amines

**DOI:** 10.1038/s41467-021-26194-y

**Published:** 2021-10-07

**Authors:** Xiao-Biao Yan, Lun Li, Wen-Qiang Wu, Lun Xu, Ke Li, Yu-Cheng Liu, Hang Shi

**Affiliations:** 1 Key Laboratory of Precise Synthesis of Functional Molecules of Zhejiang Province, School of Science, Westlake University, 18 Shilongshan Road, Hangzhou, 310024 China; 2 Institute of Natural Sciences, Westlake Institute for Advanced Study, 18 Shilongshan Road, Hangzhou, 310024 China

**Keywords:** Homogeneous catalysis, Synthetic chemistry methodology

## Abstract

Hydroalkylation, the direct addition of a C(sp^3^)–H bond across an olefin, is a desirable strategy to produce valuable, complex structural motifs in functional materials, pharmaceuticals, and natural products. Herein, we report a reliable method for accessing α-branched amines via nickel-catalyzed hydroalkylation reactions. Specifically, by using bis(cyclooctadiene)nickel (Ni(cod)_2_) together with a phosphine ligand, we achieved a formal C(sp^3^)–H bond insertion reaction between olefins and N-sulfonyl amines without the need for an external hydride source. The amine not only provides the alkyl motif but also delivers hydride to the olefin by means of a nickel-engaged β–hydride elimination/reductive elimination process. This method provides a platform for constructing chiral α-branched amines by using a P-chiral ligand, demonstrating its potential utility in organic synthesis. Notably, a sulfonamidyl boronate complex formed in situ under basic conditions promotes ring-opening of the azanickellacycle reaction intermediate, leading to a significant improvement of the catalytic efficiency.

## Abstract (web summary)

Catalytic addition of a carbon chain and a hydrogen across a double bond has often required an added hydride source. Here the authors show a method to add alkanes with an amino functionality to olefins, wherein a nickel catalyst uses the amine itself as the hydride source, obviating an external hydride reagent.

## Introduction

**Fig. 1:**
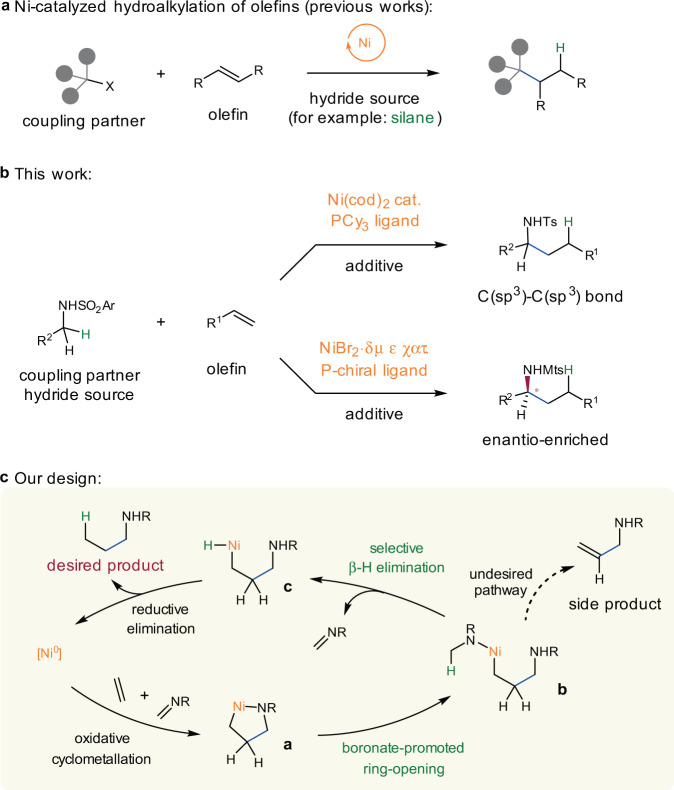
Nickel-catalyzed hydroalkylation of olefins. **a** Hydroalkylation of olefins with an additional hydride source. **b** An N-sulfonyl amine serves both as a hydride source and a coupling partner in the hydroalkylation of olefins. **c** Envisioned catalytic cycle and challenges. cat. catalyst, PCy_3_ tricyclohexylphosphine, Ar aryl, Ts tosyl, Mts mesitylen-2-sulfonyl.

Compounds featured with a carbon–carbon double bond serve as important precursors for complex aliphatic molecules because of their ready availability and versatility in transition-metal-catalyzed functionalizations^1–16^. In this respect, advances in nickel-complex-catalyzed hydrocarbonation of olefins have expanded the chemical space of accessible structures and enabled new synthetic disconnections^17–21^. Compared with hydroarylation^22–34^ and hydroalkenylation^26,32,35^, hydroalkylation produces molecules that are richer in sp^3^-hybridized carbon centers and contain more stereogeometric information^36–42^, a feature that may improve biological activity^43,44^. In the 1990s, Mori disclosed intramolecular C(sp^3^)–C(sp^3^) bond formation reactions between conjugated dienes and carbonyl groups with catalysis by a nickel hydride complex generated by treatment of Ni(cod)_2_ with Et_3_SiH^45^. In the past 5 years, an array of elegant Ni-catalyzed hydroalkylation reactions between olefins and alkyl halides in the presence of a silane-based hydride source, including both enantiospecific and enantioconvergent versions, have been established (Fig. 1a)^36–42^. In 2020, Koh’s group developed an aminoquinaldine-directed hydroalkylation reaction, in which one alkyl halide molecule provides an alkyl motif and another delivers a hydride via β-H elimination^46^. In addition to alkyl halides, imines or aldehyde can also be used as coupling partners for hydroalkylation reactions of tetrafluoroethylene with silanes^47,48^.

Inspired by Ni-engaged oxidative cyclometallation ([Ni^0^] to nickellacycle **a** in Fig. 1c), which have been successfully applied in alkenylation of imines with unsaturated molecules such as alkynes and olefins^49–56^, we envisioned that if an amine could serve both as a hydride source and an imine precursor, formal olefin insertion into the α-C–H bond of the amine could be accomplished (Fig. 1c). Although the Ni-catalyzed alkenylation reaction between alkynes and amines has been reported^57,58^, the above-described strategy poses a significant challenge in the form of competitive hydride elimination from one of the β-positions relative to the nickel atom (**b** to **c** vs **b** to side product). For instance, the alkenylation reactions between olefins and imines established by Zhou’s group provided unsaturated products, allylic amines^54^. When tetrafluoroethene bearing no hydrogen atom was subjected together with silane, alkylation of imines took place^47^. Moreover, Ogoshi’s group recently used carbonyl insertion to interrupt the facile β-H elimination; displacement of the nickel from the nickellacycle intermediate provides saturated γ-lactams^59,60^.

In this work, we report a Ni-catalyzed hydroalkylation of olefins with N-sulfonyl amines, which provides α-branched amines without the need for an exogenous hydride source, and obtains high enantioselectivity by using a P-chiral phosphine ligand (Fig. 1b).

## Results

### Reaction optimization

**Fig. 2:**
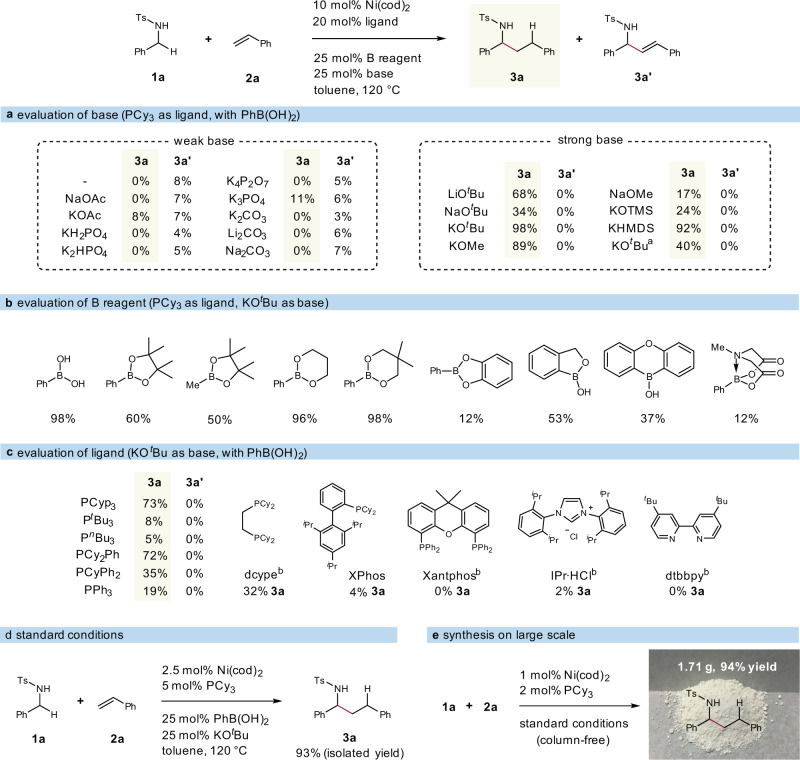
Reaction optimization. **a** Evaluation of base. **b** Evaluation of boron reagent. **c** Evaluation of ligand. **d** Standard reaction conditions. **e** Gram-scale reaction. Reaction conditions: **1a** (0.2 mmol), **2a** (0.4 mmol), Ni(cod)_2_ (10 mol%), ligand (20 mol%), PhB(OH)_2_ (25 mol%), base (25 mol%), toluene (0.3 mL), N_2_, 120 °C; yields were determined by ^1^H NMR spectroscopy. ^a^Without PhB(OH)_2_. ^b^Ligand (10 mol%). B boron, Ac acetyl, Bu butyl, Me methyl, TMS trimethylsilyl, KHMDS potassium bis(trimethylsilyl)amide, Ph phenyl, Pr propyl.

To evaluate the feasibility of our strategy, we selected N-tosyl benzylamine (**1a**) and styrene (**2a**) as coupling partners. We conducted the hydroalkylation by using a Ni(0) species Ni(cod)_2_ and a phosphine ligand PCy_3_. After trying a number of weak inorganic bases (Fig. 2a), including NaOAc and K_3_PO_4_, which are crucial in our previous studies^57^, we only detect a trace amount of the desired hydroalkylation product **3a** together with a side-product allylic amine **3a’** by ^1^H NMR spectroscopy. However, we found strong bases could dramatically promote the designed reaction profile, as well as suppress the competitive pathway that leads to **3a’**. For instance, KO^*t*^Bu provided **3a** in an almost quantitative yield (98% NMR yield) with a 10 mol% Ni catalyst. Phenyl boronic acid is not necessary, but it influenced the efficiency of this catalysis that a lower yield (40%) was obtained in the absence of it. Moreover, we evaluated other boron reagents and found that 2-phenyl-1,3,2-dioxaborinane and its analogue bearing no protons also gave high yields (Fig. 2b). Next, we moved to evaluate ligands beside PCy_3_, including monodentate and bidentate phosphines, as well as other type pivotal ligands: the analogues of PCy_3_, such as PCyp_3_ (Cyp, cyclopentyl group) and PCy_2_Ph, yielded product **3a** in around 70% yields; the use of other ligands resulted in much lower or even undetectable yields (Fig. 2c).

Although nickel is more earth-abundant and much less expensive than precious metals (Pd, Rh, Ir, etc.), carrying out reactions with less catalysts is vital from both an atom-economy and an environmentally friendly standpoint. Therefore, we carried out experiments with lower catalyst loadings and found that the loading of the nickel/phosphine catalyst could be reduced to 2.5 mol% with no obvious decrease in yield (Fig. 2d). In addition, a gram-scale reaction with a nickel loading of only 1 mol% afforded **3a** with no need for column chromatography (Fig. 2e), and the protecting group (Ts) could be easily displaced by a Boc group (see Supplementary Methods 2.4 for details).

### Substrate scope

**Fig. 3:**
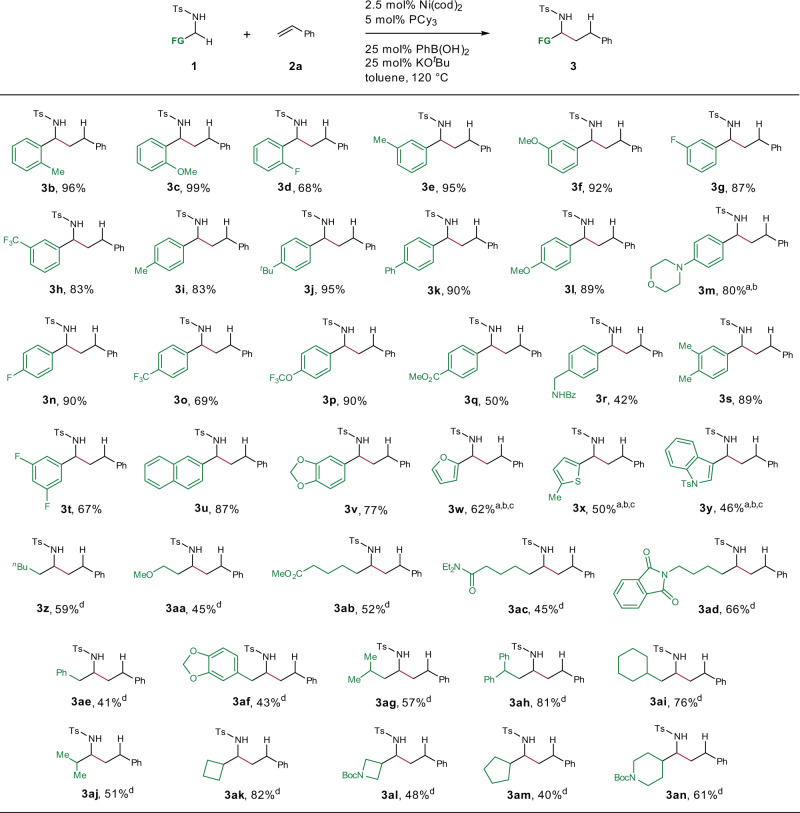
Substrate scope of N-tosyl amines. Reaction conditions: N-sulfonyl amine **1** (0.2 mmol), **2a** (0.4 mmol), Ni(cod)_2_ (2.5 mol%), PCy_3_ (5 mol%), PhB(OH)_2_ (25 mol%), KO^*t*^Bu (25 mol%), toluene (0.3 mL), N_2_, 120 °C; isolated yields were reported. ^a^Ni(cod)_2_ (5 mol%), PCy_3_ (10 mol%). ^b^PhB(OH)_2_ (100 mol%), KO^*t*^Bu (50 mol%). ^c^Pivaldehyde (2.0 equiv.) was added. ^d^Ni(cod)_2_ (5 mol%), PCy_3_ (10 mol%), PhB(OH)_2_ (100 mol%), KO^*t*^Bu (100 mol%). Bz, benzoyl, Et ethyl, Boc *tert*-butyloxycarbonyl.

With the optimized reaction conditions in hand, we investigated the generality of this method. First, we examined the scope of the reaction with respect to the N-tosyl amine (Fig. 3). A wide range of benzylic amines bearing an *ortho* (**3b**–**d**), *meta* (**3e**–**h**), or *para* (**3i**–**r**) substituent on the aromatic ring underwent the coupling reaction with styrene (**2a**), delivering the desired α-branched amines in 42–99% yields. Substrates with disubstituted phenyl rings (**3s**–**t** and **3v**) or a naphthyl ring (**3** **u**) were also well tolerated. Heterocycles containing an oxygen, sulfur, or nitrogen atom are prevalent in pharmaceuticals, but metal-catalyzed reactions involving such compounds are challenging because of coordination between the heteroatom and the metal. Indeed, we found that heteroatom-containing substrates gave low yields (**3w**–**y**) under the standard conditions. When a higher loading of the catalyst (5 mol%) and an additive, pivaldehyde, were used, the reactions afforded the desired products in moderate yields.

In addition to benzylic amines, various primary aliphatic amines were also acceptable substrates under modified conditions. Substrates with linear (**3z**–**af**), γ-branched (**3ag**–**ai**), β-branched (**3aj**), and cyclic (**3ak**–**an**) alkyl groups at the α-position of the nitrogen were tolerated, affording the corresponding unsymmetrical α-branched amines in moderate to good yields.

**Fig. 4:**
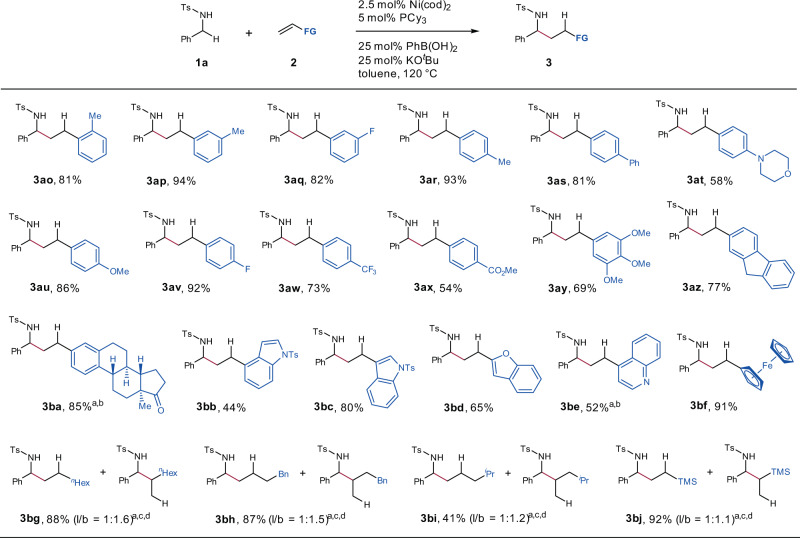
Substrate scope of olefins. Reaction conditions: **1a** (0.2 mmol), olefin **2** (0.4 mmol), Ni(cod)_2_ (2.5 mol%), PCy_3_ (5 mol%), PhB(OH)_2_ (25 mol%), KO^*t*^Bu (25 mol%), toluene (0.3 mL), N_2_, 120 °C; isolated yields were reported. ^a^Ni(cod)_2_ (5 mol%), PCy_3_ (10 mol%). ^b^PhB(OH)_2_ (100 mol%), KO^*t*^Bu (50 mol%). ^c^Pivaldehyde (2.0 equiv.) was added. ^d^The ratio of linear product to branched product (l/b) was determined by ^1^H NMR spectroscopy. Hex hexyl, Bn benzyl.

The scope of olefins was then examined with N-tosyl amine **1a** (Fig. 4). Both electron-donating and -withdrawing groups on the phenyl rings of olefins were well tolerated; desired products **3ao**–**ba** were obtained in moderate to excellent yields. Moreover, a diverse array of functionalities such as fluorine, trifluoromethyl, methoxy, ester, morpholino, and ketone were tolerated. Multisubstituted aryl olefins, including a molecule derived from estrone, were suitable substrates, affording **3ay**–**ba** in good yields. Olefins containing a heterocycle, such as indole, benzofuran, and quinoline, as well as ferrocene were also compatible with the reaction conditions. In addition, hydroalkylation of aliphatic olefins with **1a** in the presence of pivaldehyde provided mixtures of linear and branched products in 41–92% yields.

We next investigated whether this protocol could be applied to access enantioenriched α-branched N-tosyl amines, which are valuable and privileged motif found in nature products, pharmaceuticals, and functional molecules. We immediately encountered a significant challenge in that a chiral ligand such as P-chiral phosphine (*R*)-BI-DIME^61^ together with Ni(cod)_2_, provided the desired product with a poor enantioselectivity (57.2:42.8 er) (see Supplementary Table 11). We suspected that the 1,5-cyclooctadiene (cod) liberated from Ni(cod)_2_ may rebind to the metal center during the catalysis, leading to a disturbance in enantioselectivity control. To diminish such influence, we evaluated other Ni sources, and found that a Ni(II) precatalyst NiBr_2_·dme, which could be reduced to Ni(0) in situ, dramatically improved the enantioselectivity (79.6:20.4 er). Instead of the tosyl group, using a bulkier mesitylen-2-sulfonyl group (Mts) as the protecting group for nitrogen resulted in a slightly better result (83.6:17.4 er). Further optimization of the conditions, including solvent and reaction temperature allowed us to obtain **5a** in 55% isolated yield with 92.0:8.0 er (for more details, see Supplementary Table 11).

**Fig. 5:**
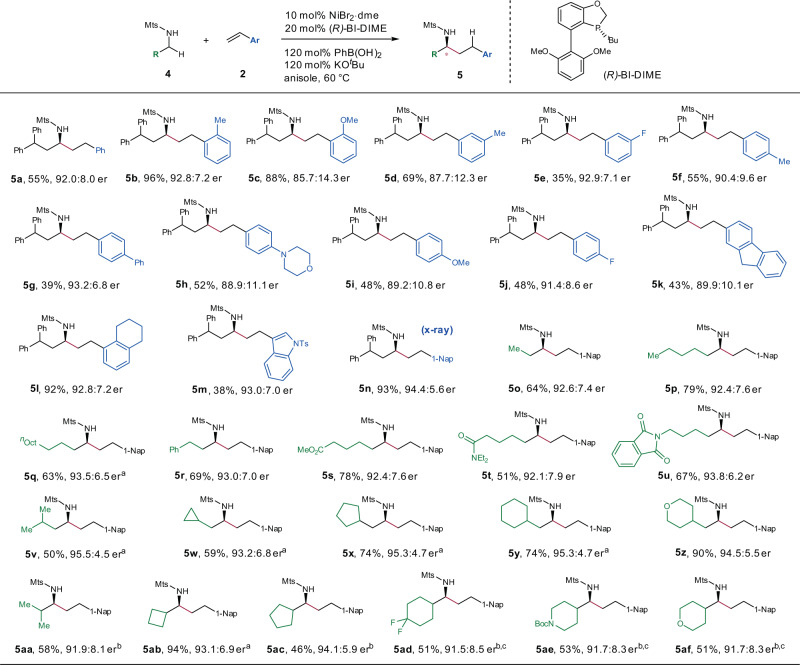
Substrate scope of enantioselective hydroalkylation. Reaction conditions: **4** (0.1 mmol), olefin **2** (0.2 mmol), NiBr_2_·dme (10 mol%), (*R*)-BI-DIME (20 mol%), PhB(OH)_2_ (120 mol%), KO^*t*^Bu (120 mol%), anisole (0.2 mL), N_2_, 60 °C; isolated yields were reported. ^a^Reaction was conducted at 50 ^o^C. ^b^Reaction was conducted at 80 ^o^C. ^c^(*R*)-BI-DIME (12 mol%) was used. Nap naphthyl, Oct octyl.

Then, we move to investigate the substrate scope of the enantioselective protocol by performing reactions of aliphatic N-Mts amines with olefins (Fig. 5). First, various styrene analogues were compatible under the optimized conditions, providing desired products in moderate yields with good enantioselectivities (**5a**–**n**). Second, the scope of amines is also broad that both acyclic (**5o**–**aa**) and cyclic (**5ab**–**af**) aliphatic amines were suitable. Notably, active functional groups, including ester (**5s**), amide (**5t**), imide (**5u**), carbamate (**5ae**), and cyclopropyl (**5w**) were well tolerated. The absolute configuration of (*S*)-**5n** was determined by X-ray crystallography (see Supplementary Note 2).

### Mechanistic studies

**Fig. 6:**
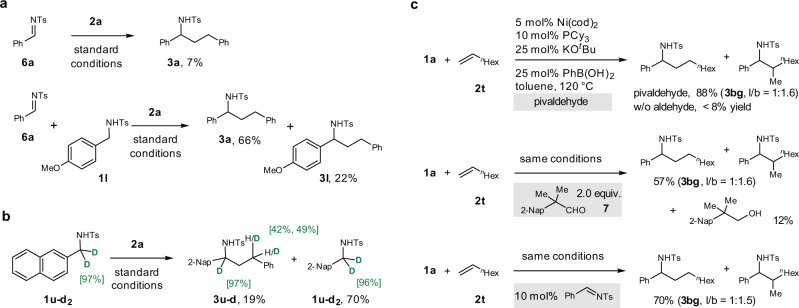
Mechanistic studies. **a** Hydroalkylation of styrene with N-tosyl imine. **b** Deuterium labeling experiment. **c** Effects of an aldehyde in the hydroalkylation of 1-octene.

To gain insights into the reaction mechanism, we performed mechanistic studies. First, we replaced amine **1a** with N-tosyl imine **6a** and found that the reaction yielded only a trace of desired product **3a** (Fig. 6a). Moreover, adding *para*-methoxybenzylamine (**1** **l**) to the above reaction mixture dramatically increased the yield of **3a**. These experiments suggested that the N-tosyl amine served not only as the precursor of the imine but also as the hydride source to terminate the catalytic cycle. Second, deuterium-labeling experiments were conducted (Fig. 6b). When the α-deuterated substrate **1u-d**_**2**_ was used, 45.5% average deuterium incorporation at the γ-position relative to the nitrogen of the product was detected, and the recovered substrate showed no loss of deuterium. This observation is consistent with our assumption that the hydrogen in the product was derived mainly from the N-tosyl amine. Third, control experiments were carried out to investigate the role played by pivaldehyde in the reactions of aliphatic olefins (Fig. 6c). In the absence of the aldehyde, only a very low yield was obtained (<8%). In contrast, when an α,α,α-trisubstituted aldehyde α-naphthalenyl isobutyraldehyde **7** (Compared to pivaldehyde, α-naphthalenyl isobutyraldehyde and its corresponding alcohol are easier to observe by ^1^H NMR spectroscopy) was used, **3bg** was obtained in 57% yield, along with an alcohol derived from the aldehyde. Moreover, a catalytic amount of N-tosyl imine (10 mol%) also promoted the reaction, which indicates that the aldehyde additive may accept hydride from the Ni–H species generated in the initial step (N-tosyl amine dehydration), prior to catalysis.

**Fig. 7:**
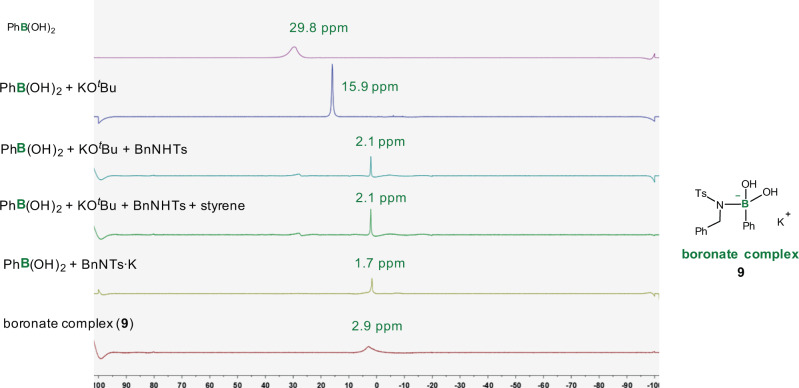
^11^B NMR (toluene-*d*_*8*_) studies. ^11^B NMR spectroscopy was used to evaluate interactions between PhB(OH)_2_ and reactants, including N-tosyl amine, styrene, and KO^*t*^Bu.

In addition, we investigated effects of the base and boron reagent. First, a strong base such as KO^*t*^Bu, LiO^*t*^Bu, and KOMe was found to be indispensable; while, beside PhB(OH)_2_, a borate such as 2-phenyl-1,3,2-dioxaborinane also dramatically promoted the reaction (Fig. 2). Second, we used ^11^B NMR to evaluate interactions between PhB(OH)_2_ and reactants, including N-tosyl amine, styrene, and KO^*t*^Bu (Fig. 7). After heating the reaction mixture for a while (in the absence of Ni/P-catalyst), a new peak appeared in the upfield, implying the formation of a boronate complex. Notably, this peak still appeared either in the absence of an olefin or when PhB(OH)_2_ was treated with potassium sulfonamide (**8**) directly. Furthermore, a boronate complex (**9**) bearing a B–N bond was isolated and characterized by NMR spectroscopy as well as elementary analysis ([C_20_H_21_BKNO_4_S], calcd. for B: 2.57%; found: 2.73%.). Beside KO^*t*^Bu, we subjected LiO^*t*^Bu into the above experiments, and also observed the formation of lithium boronate by ^11^B NMR spectroscopy (see Supplementary Methods 2.6 for details). Inspired by the previous observations that compounds bearing active protons (e.g. TsNH_2_, phenol) could promote opening of the five-membered nickellacycle intermediate via protonation^50,54,62–64^, as well as studies of transmetallation on boronates^65–69^, we proposed that a boronate facilitates exchange of the sulfonamidyl group on the nickel, leading to rapid opening of the nickellacycle (**a** to **b** in Fig. 1c).

**Fig. 8:**
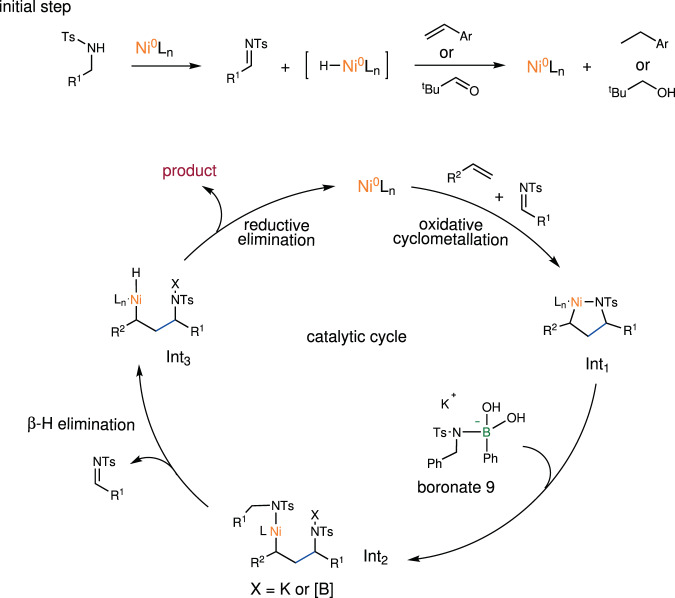
Proposed catalytic cycle. Possible reaction mechanism of the Ni-catalyzed hydroalkylation of olefins with N-sulfonyl amines.

On the basis of the aforementioned experiments and previous studies^49–51,54,56,57^, we proposed the mechanism outlined in Fig. 8. The process is initiated by dehydrogenation of a N-sulfonyl amine, liberating a catalytic amount of the corresponding imine together with a Ni-H species. Subsequently, styrene or the additive pivaldehyde accepts hydride to regenerate the active Ni(0) catalyst. In the catalytic cycle, oxidative cyclometallation produces a nickellacycle intermediate **Int**_**1**_, which is converted into the nickel intermediate **Int**_**2**_ through a boronate-promoted exchange of sulfonamidyl group. Finally, the desired product is derived from **Int**_**2**_ through a β-H elimination/reductive elimination process, completing the catalytic cycle.

In summary, we have developed a method for nickel-catalyzed hydroalkylation reactions between terminal olefins and linear N-sulfonyl amines to afford a variety of branched products. The method is atom economical because an exogenous hydride source is not required. Mechanistic studies suggested that a sulfonamidyl boronate complex formed in situ facilitates the transformation by promoting the opening of the nickellacycle. Further work aimed at extending this protocol to related transformations is currently underway in our laboratory.

## Methods

### General procedure for hydroalkylation of olefins with N-sulfonyl amines

In a N_2_-filled glovebox, a 4 mL oven-dried vial was charged with N-sulfonyl amine **1** (0.2 mmol, 1.0 equiv.), olefin **2** (0.4 mmol, 2.0 equiv.), Ni(cod)_2_ (0.005 mmol, 1.4 mg, 2.5 mol%), PCy_3_ (0.01 mmol, 2.8 mg, 5 mol%), PhB(OH)_2_ (0.05 mmol, 6.1 mg, 25 mol%) and KO^*t*^Bu (0.05 mmol, 5.6 mg, 25 mol%). Toluene (0.3 mL) was added. The vial was equipped with a magnetic stir bar, sealed, and the reaction mixture was stirred at 120 °C for 20 h. The reaction mixture was cooled to room temperature and concentrated under reduced pressure. Purification by column chromatography afforded the desired product.

### General procedure for enantioselective hydroalkylation of olefins with N-sulfonyl amines

In a N_2_-filled glovebox, a 4 mL oven-dried vial was charged with N-sulfonyl amine **4** (0.1 mmol, 1.0 equiv.), olefin **2** (0.2 mmol, 2.0 equiv.), NiBr_2_·dme (0.01 mmol, 3.1 mg, 10 mol%), (*R*)-BI-DIME (0.02 mmol, 6.6 mg, 20 mol%), PhB(OH)_2_ (0.12 mmol, 14.6 mg, 120 mol%) and KO^*t*^Bu (0.12 mmol, 13.4 mg, 120 mol%). Anisole (0.2 mL) was added. The vial was equipped with a magnetic stir bar, sealed, and the reaction mixture was stirred at 60 °C for 72 h. The reaction mixture was cooled to room temperature and concentrated under reduced pressure. Purification by column chromatography afforded the desired product.

## Supplementary information

Supplementary Information

## Data Availability

All data supporting the findings of this study are available within the article and Supplementary Information files, or from the corresponding author upon reasonable request. The X-ray crystallographic coordinates for structure of **5n** reported in this study have been deposited at the Cambridge Crystallographic Data Centre (CCDC), under deposition number 2092983. The data can be obtained free of charge from the Cambridge Crystallographic Data Centre via http://www.ccdc.cam.ac.uk/data_request/cif.

## References

[1] McDonald RI, Liu G, Stahl SS (2011). Palladium(II)-catalyzed alkene functionalization via nucleopalladation: stereochemical pathways and enantioselective catalytic applications. Chem. Rev..

[2] Saini V, Stokes BJ, Sigman MS (2013). Transition-metal-catalyzed laboratory-scale carbon-carbon bond-forming reactions of ethylene. Angew. Chem. Int. Ed..

[3] Crossley SWM, Obradors C, Martinez RM, Shenvi RA (2016). Mn-, Fe-, and Co-catalyzed radical hydrofunctionalizations of olefins. Chem. Rev..

[4] Dong Z, Ren Z, Thompson SJ, Xu Y, Dong G (2017). Transition-metal-catalyzed C−H alkylation using alkenes. Chem. Rev..

[5] Holmes M, Schwartz LA, Krische MJ (2018). Intermolecular metal-catalyzed reductive coupling of dienes, allenes, and enynes with carbonyl compounds and imines. Chem. Rev..

[6] Liu RY, Buchwald SL (2020). CuH-catalyzed olefin functionalization: from hydroamination to carbonyl addition. Acc. Chem. Res..

[7] Derosa J, Apolinar O, Kang T, Tran VT, Engle KM (2020). Recent developments in nickel-catalyzed intermolecular dicarbofunctionalization of alkenes. Chem. Sci..

[8] Jun, C.-H., Hwang, D.-C. & Na, S.-J. Chelation-assisted alkylation of benzylamine derivatives by Ru^0^ catalyst. *Chem. Commun*. 1405−1406 (1998).

[9] Chatani N (2001). Ru_3_(CO)_12_-catalyzed coupling reaction of sp^3^ C−H bonds adjacent to a nitrogen atom in alkylamines with alkenes. J. Am. Chem. Soc..

[10] Herzon SB, Hartwig JF (2007). Direct, catalytic hydroaminoalkylation of unactivated olefins with N-alkyl arylamines. J. Am. Chem. Soc..

[11] Kubiak R, Prochnow I, Doye S (2010). [Ind_2_TiMe_2_]: a catalyst for the hydroaminomethylation of alkenes and styrenes. Angew. Chem. Int. Ed..

[12] Reznichenko AL, Hultzsch KC (2012). The mechanism of hydroaminoalkylation catalyzed by Group 5 metal binaphtholate complexes. J. Am. Chem. Soc..

[13] Thullen SM, Rovis T (2017). A mild hydroaminoalkylation of conjugated dienes using a unified cobalt and photoredox catalytic system. J. Am. Chem. Soc..

[14] Koperniku A, Foth PJ, Sammis GM, Schafer LL (2019). Zirconium hydroaminoalkylation. an alternative disconnection for the catalytic synthesis of α-arylated primary amines. J. Am. Chem. Soc..

[15] Bielefeld J, Doye S (2020). Fast titanium-catalyzed hydroaminomethylation of alkenes and the formal conversion of methylamine. Angew. Chem. Int. Ed..

[16] Daneshmand P (2020). Cyclic ureate tantalum catalyst for preferential hydroaminoalkylation with aliphatic amines: mechanistic insights into substrate controlled reactivity. J. Am. Chem. Soc..

[17] Sommer H, Juliá-Hernández F, Martin R, Marek I (2018). Walking metals for remote functionalization. ACS Cent. Sci..

[18] Xiao L-J, Ye M-C, Zhou Q-L (2019). Nickel-catalyzed highly atom-economical C–C coupling reactions with π components. Synlett.

[19] Wang X-X, Lu X, Li Y, Wang J-W, Fu Y (2020). Recent advances in nickel-catalyzed reductive hydroalkylation and hydroarylation of electronically unbiased alkenes. Sci. China Chem..

[20] He Y, Cai Y, Zhu S (2017). Mild and regioselective benzylic C–H functionalization: Ni-catalyzed reductive arylation of remote and proximal olefins. J. Am. Chem. Soc..

[21] Gaydou M, Moragas T, Juliá-Hernández F, Martin R (2017). Site-selective catalytic carboxylation of unsaturated hydrocarbons with CO_2_ and water. J. Am. Chem. Soc..

[22] Bair JS (2014). Linear-selective hydroarylation of unactivated terminal and internal olefins with trifluoromethyl-substituted arenes. J. Am. Chem. Soc..

[23] Okumura S (2016). *para*-Selective alkylation of benzamides and aromatic ketones by cooperative nickel/aluminum catalysis. J. Am. Chem. Soc..

[24] Xiao L-J (2018). Nickel(0)-catalyzed hydroarylation of styrenes and 1,3-dienes with organoboron compounds. Angew. Chem. Int. Ed..

[25] Diesel J, Finogenova AM, Cramer N (2018). Nickel-catalyzed enantioselective pyridone C–H functionalizations enabled by a bulky N-heterocyclic carbene ligand. J. Am. Chem. Soc..

[26] Chen Y-G (2019). Nickel-catalyzed enantioselective hydroarylation and hydroalkenylation of styrenes. J. Am. Chem. Soc..

[27] Lv H (2019). Nickel-catalyzed intermolecular oxidative Heck arylation driven by transfer hydrogenation. Nat. Commun..

[28] Zhang W-B, Yang X-T, Ma J-B, Su Z-M, Shi S-L (2019). Regio- and enantioselective C–H cyclization of pyridines with alkenes enabled by a nickel/N-heterocyclic carbene catalysis. J. Am. Chem. Soc..

[29] Saper NI (2020). Nickel-catalysed anti-Markovnikov hydroarylation of unactivated alkenes with unactivated arenes facilitated by non-covalent interactions. Nat. Chem..

[30] Wang D-M, Feng W, Wu Y, Liu T, Wang P (2020). Redox-neutral nickel(II) catalysis: hydroarylation of unactivated alkenes with arylboronic acids. Angew. Chem. Int. Ed..

[31] He Y, Liu C, Yu L, Zhu S (2020). Enantio- and regioselective NiH-catalyzed reductive hydroarylation of vinylarenes with aryl iodides. Angew. Chem. Int. Ed..

[32] Li Z-Q (2020). Ligand-controlled regiodivergence in nickel-catalyzed hydroarylation and hydroalkenylation of alkenyl carboxylic acids. Angew. Chem. Int. Ed..

[33] Cuesta-Galisteo S, Schörgenhumer J, Wei X, Merino E, Nevado C (2021). Nickel-catalyzed asymmetric synthesis of α-arylbenzamides. Angew. Chem. Int. Ed..

[34] Huang X (2021). Enantioselective intermolecular Heck and reductive Heck reactions of aryl triflates, mesylates, and tosylates catalyzed by nickel. Angew. Chem. Int. Ed..

[35] Liu J, Gong H, Zhu S (2021). Nickel-catalyzed, regio- and enantioselective benzylic alkenylation of olefins with alkenyl bromide. Angew. Chem. Int. Ed..

[36] Lu X (2016). Practical carbon–carbon bond formation from olefins through nickel-catalyzed reductive olefin hydrocarbonation. Nat. Commun..

[37] Wang Z, Yin H, Fu GC (2018). Catalytic enantioconvergent coupling of secondary and tertiary electrophiles with olefins. Nature.

[38] Sun S-Z, Börjesson M, Martin-Montero R, Martin R (2018). Site-selective Ni-catalyzed reductive coupling of α-haloboranes with unactivated olefins. J. Am. Chem. Soc..

[39] He S-J (2020). Nickel-catalyzed enantioconvergent reductive hydroalkylation of olefins with α-heteroatom phosphorus or sulfur alkyl electrophiles. J. Am. Chem. Soc..

[40] Bera S, Mao R, Hu X (2021). Enantioselective C(sp^3^)–C(sp^3^) cross-coupling of non-activated alkyl electrophiles via nickel hydride catalysis. Nat. Chem..

[41] Shi L, Xing L-L, Hu W-B, Shu W (2021). Regio- and enantioselective Ni-catalyzed formal hydroalkylation, hydrobenzylation, and hydropropargylation of acrylamides to α-tertiary amides. Angew. Chem. Int. Ed..

[42] Qian D, Bera S, Hu X (2021). Chiral alkyl amine synthesis via catalytic enantioselective hydroalkylation of enecarbamates. J. Am. Chem. Soc..

[43] Ritchie TJ, Macdonald SJ (2009). The impact of aromatic ring count on compound developability-are too many aromatic rings a liability in drug design?. Drug Discov. Today.

[44] Lovering F, Bikker J, Humblet C (2009). Escape from flatland: increasing saturation as an approach to improving clinical success. J. Med. Chem..

[45] Sato Y (1994). Novel stereoselective cyclization via.pi.-allylnickel complex generated from 1,3-diene and hydride nickel complex. J. Am. Chem. Soc..

[46] Chen X, Rao W, Yang T, Koh MJ (2020). Alkyl halides as both hydride and alkyl sources in catalytic regioselective reductive olefin hydroalkylation. Nat. Commun..

[47] Shirataki H, Ono T, Ohashi M, Ogoshi S (2019). Ni(0)-catalyzed three-component coupling reaction of tetrafluoroethylene and N-sulfonyl-substituted imines with silanes via aza-nickelacycles. Org. Lett..

[48] Shirataki, H., Ohashi, M. & Ogoshi, S. Nickel-catalyzed three-component coupling reaction of tetrafluoroethylene and aldehydes with silanes via oxa-nickelacycles. *Eur. J. Org. Chem*. 1883–1887 (2019). 10.1021/acs.orglett.8b03674 30582815

[49] Ogoshi S, Oka M-a, Kurosawa H (2004). Direct observation of oxidative cyclization of η^2^-alkene and η^2^-aldehyde on Ni(0) center. significant acceleration by addition of Me_3_SiOTf. J. Am. Chem. Soc..

[50] Yeh C-H, Korivi RP, Cheng C-H (2008). Regioselective synthesis of γ-amino esters, nitriles, sulfones, and pyrrolidinones by nickel-catalyzed reductive coupling of aldimines and activated alkenes. Angew. Chem. Int. Ed..

[51] Jackson EP (2015). Mechanistic basis for regioselection and regiodivergence in nickel-catalyzed reductive couplings. Acc. Chem. Res..

[52] Patel SJ, Jamison TF (2003). Catalytic three-component coupling of alkynes, imines, and organoboron reagents. Angew. Chem. Int. Ed..

[53] Ohashi M, Kishizaki O, Ikeda H, Ogoshi S (2009). Ni(0)-catalyzed formation of azaaluminacyclopentenes via azanickelacyclopentenes: a unique nickel/aluminum double transmetalation reaction. J. Am. Chem. Soc..

[54] Xiao L-J (2018). Nickel(0)-catalyzed hydroalkenylation of imines with styrene and its derivatives. Angew. Chem. Int. Ed..

[55] Yao W-W, Li R, Li J-F, Sun J, Ye M (2019). NHC ligand-enabled Ni-catalyzed reductive coupling of alkynes and imines using isopropanol as a reductant. Green. Chem..

[56] Fan C, Lv X-Y, Xiao L-J, Xie J-H, Zhou Q-L (2019). Alkenyl exchange of allylamines via nickel(0)-catalyzed C–C bond cleavage. J. Am. Chem. Soc..

[57] Li L, Liu Y-C, Shi H (2021). Nickel-catalyzed enantioselective α-alkenylation of N-sulfonyl amines: modular access to chiral α-branched amines. J. Am. Chem. Soc..

[58] Yao W-W (2021). Ni-catalyzed hydroaminoalkylation of alkynes with amines. Nat. Commun..

[59] Hoshimoto Y (2017). Efficient synthesis of polycyclic γ-lactams by catalytic carbonylation of ene-imines via nickelacycle intermediates. Angew. Chem. Int. Ed..

[60] Ashida K (2020). Enantioselective synthesis of polycyclic γ-lactams with multiple chiral carbon centers via Ni(0)-catalyzed asymmetric carbonylative cycloadditions without stirring. J. Am. Chem. Soc..

[61] Xu G, Senanayake CH, Tang W (2019). P‑chiral phosphorus ligands based on a 2,3-dihydrobenzo[*d*][1,3]oxaphosphole motif for asymmetric catalysis. Acc. Chem. Res..

[62] Jenkins AD, Herath A, Song M, Montgomery J (2011). Synthesis of cyclopentenols and cyclopentenones via nickel-catalyzed reductive cycloaddition. J. Am. Chem. Soc..

[63] Han X-W (2018). Brønsted acid enabled nickel-catalyzed hydroalkenylation of aldehydes with styrene and its derivatives. Angew. Chem. Int. Ed..

[64] Han X-W, Zhang T, Yao W-W, Chen H, Ye M (2020). Nickel- and Brønsted acid-catalyzed redox-neutral coupling of 1,3-dienes and aldehydes for synthesis of dienols. CCS Chem..

[65] Matos K, Soderquist JA (1998). Alkylboranes in the Suzuki−Miyaura coupling: stereochemical and mechanistic studies. J. Org. Chem..

[66] Carrow BP, Hartwig JF (2011). Distinguishing between pathways for transmetalation in Suzuki−Miyaura reactions. J. Am. Chem. Soc..

[67] Thomas AA, Denmark SE (2016). Pre-transmetalation intermediates in the Suzuki-Miyaura reaction revealed: the missing link. Science.

[68] Payard P-A, Perego LA, Ciofini I, Grimaud L (2018). Taming nickel-catalyzed Suzuki-Miyaura coupling: a mechanistic focus on boron-to-nickel transmetalation. ACS Catal..

[69] Malapit CA, Bour JR, Laursen SR, Sanford MS (2019). Mechanism and scope of nickel-catalyzed decarbonylative borylation of carboxylic acid fluorides. J. Am. Chem. Soc..

