# Supplementary material for: Ni-catalyzed hydroalkylation of olefins with N-sulfonyl amines
Source: Nat Commun. 2021 Oct 7;12:5881. doi: 10.1038/s41467-021-26194-y (PMC8497516; doi:10.1038/s41467-021-26194-y)
Supplement: Supplementary file 1 — Supplementary Information [file 41467_2021_26194_MOESM1_ESM.pdf]

Supplementary Information

Ni-catalyzed hydroalkylation of olefins with N-sulfonyl amines

Xiao-Biao Yan[1,2,3], Lun Li[1,2,3], Wen-Qiang Wu[1], Lun Xu[1], Ke Li[1,2], Yu-Cheng Liu[1] & Hang Shi[1,2,*]

[1] Key Laboratory of Precise Synthesis of Functional Molecules of Zhejiang Province, School of Science, Westlake University, 18 Shilongshan Road, Hangzhou 310024, Zhejiang Province, China.
[2] Institute of Natural Sciences, Westlake Institute for Advanced Study, 18 Shilongshan Road, Hangzhou 310024, Zhejiang Province, China.
[3] These authors contributed equally: Xiao-Biao Yan, Lun Li.
[*] Corresponding authors. E-mail: shihang@westlake.edu.cn.

Table of Contents

1. Supplementary Note 1 .... S2

2. Supplementary Methods, Tables and Figures S3

2.1 Optimization of Reaction Conditions S3

2.2 Synthesis of Starting Materials S9

2.3 Nickel-Catalyzed Hydroalkylation of Olefins with N-Sulfonyl Amines S29

2.4 Gram-Scale Reaction and Protecting Group Transformation S51

2.5 Nickel-Catalyzed Enantioselective Hydroalkylation of Olefins with N-Sulfonyl Amines S53

2.6 Mechanistic Studies S86

2.7 NMR Spectra S98

3. Supplementary Note 2 .... S285

Crystal Data and Structure Refinement for **5n** S285

4. Supplementary References S287

1. Supplementary Note 1

All air-sensitive manipulations were conducted under an inert atmosphere in a nitrogen-filled glovebox. Unless otherwise noted, all chemicals used in the preparation of starting materials were commercially available and were used as received without further purification. $Ni(cod)_2$ and PCy_3 were purchased from Sinocompound and Energy Chemical Company, respectively. Anhydrous toluene was freshly distilled from sodium. Other anhydrous solvents were all purchased from J&K Chemical Company, stored in glovebox, and used without further purification.

Analytical thin layer chromatography was performed on 0.2 mm silica gel (HSGF254 indicator). Visualization was carried out with UV light or phosphomolybdic acid. Flash column chromatography was performed with silica gel (400 mesh), which purchased from Yantai Jiangyou Silica gel Development Company. NMR spectra were recorded on Bruker AVANCE NEO (500M Hz) instruments at the NMR facility of Westlake University. ^{1}H NMR spectra were reported in parts per million (ppm) downfield of tetramethylsilane (TMS) and were referenced to the signal of TMS (0 ppm) in $CDCl_3$ or residue solvent peak in CD_3OD. ^{13}C NMR spectra were reported in ppm relative to residual $CHCl_3$ (77 ppm) or CH_3OH (49 ppm). Coupling constants, *J*, are reported in hertz (Hz). ^{19}F NMR and ^{11}B NMR spectra were also collected on Bruker AVANCE NEO (500 MHz) instrument. The enantiomeric ratio value was detected on Agilent Infinity II SFC system with Chiralpak columns. High-resolution mass spectra (HRMS) were recorded on Waters high-resolution mass spectrometer in ESI mode. X-ray crystallographic analysis were performed on Bruker D8 Venture. Elementary analysis was recorded on Leeman Prodigy (ICP-OES) at Shanghai Institute of Organic Chemistry (Chinese Academy of Sciences).

2. Supplementary Methods, Tables and Figures

2.1 Optimization of Reaction Conditions

Supplementary Table 1 Optimization of Additives[a]

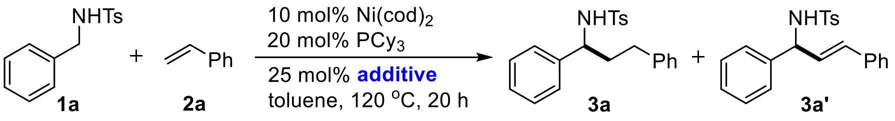

entry	additive	**3a** (%)	**3a'** (%)	entry	additive	**3a** (%)	**3a'** (%)
1	-	0	3	6	$TsNH_2$	0	3
2	$PhCO_2H$	0	0	7	$B(OH)_3$	0	1
3	$Ph_2P(O)OH$	0	0	8	$MeB(OH)_2$	0	1
4	**PA**	0	0	9	$C_6F_5B(OH)_2$	0	1
5	$TsOH{\cdot}H_2O$	0	0	10	$PhB(OH)_2$	0	8

[a] Conditions: **1a** (0.2 mmol), **2a** (2.0 equiv.), $Ni(cod)_2$ (10 mol%), PCy_3 (20 mol%), additive (25 mol%), toluene (0.3 mL), 120 °C, 20 h. Yields were determined by ^1H NMR spectroscopy using 1,1,2,2-tetrachloroethane as the internal standard.

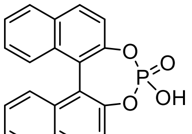

PA

Supplementary Table 2 Optimization of Bases[a]

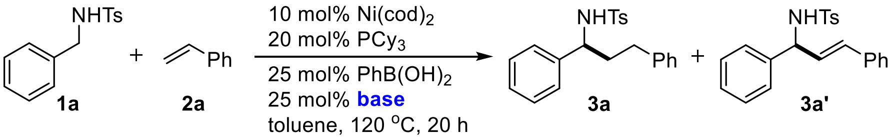

entry	base	**3a** (%)	**3a'** (%)	entry	base	**3a** (%)	**3a'** (%)
1	NaOAc	0	7	10	LiO^tBu	68	0
2	KOAc	8	7	11	NaO^tBu	34	0
3	KH_2PO_4	0	4	**12**	**KO^tBu**	**98**	**0**
4	K_2HPO_4	0	5	13	KOMe	89	0
5	$K_4P_2O_7$	0	5	14	NaOMe	17	0
6	K_3PO_4	11	6	15	KOTMS	24	0
7	Li_2CO_3	0	6	15	KHMDS	92	0
8	Na_2CO_3	0	7	17	KO^tBu	40[b]	0
9	K_2CO_3	0	3				

[a] Conditions: **1a** (0.2 mmol), **2a** (2.0 equiv.), $Ni(cod)_2$ (10 mol%), PCy_3 (20 mol%), $PhB(OH)_2$ (25 mol%), base (25 mol%), toluene (0.3 mL), 120 °C, 20 h. Yields were determined by ^1H NMR spectroscopy using 1,1,2,2-tetrachloroethane as the internal standard. [b] In the absence of $PhB(OH)_2$.

Supplementary Table 3 Optimization of B Reagents[a]

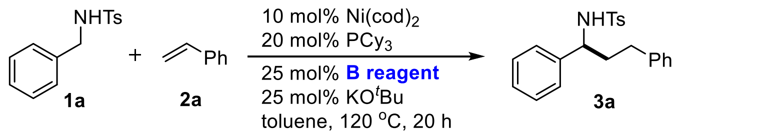

without B	$PhB(OH)_2$	$PhBF_3K$	PhBpin	MeBpin	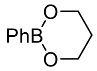
40%	98%	15%	60%	50%	96%
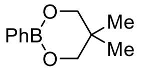	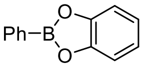	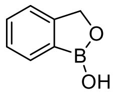	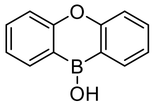	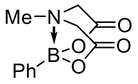	
98%	12%	53%	37%	12%	

[a] Conditions: **1a** (0.2 mmol), **2a** (2.0 equiv.), $Ni(cod)_2$ (10 mol%), PCy_3 (20 mol%), boron reagent (25 mol%), KO^tBu (25 mol%), toluene (0.3 mL), 120 °C, 20 h. Yields were determined by ^1H NMR spectroscopy using 1,1,2,2-tetrachloroethane as the internal standard.

Supplementary Table 4 Optimization of Ligands[a]

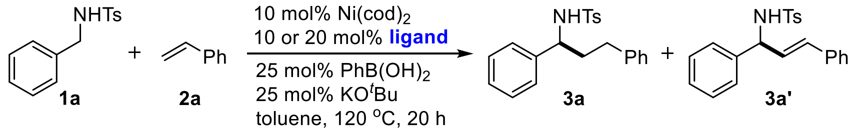

entry	ligand (mol%)	3a (%)	3a' (%)	entry	ligand (mol%)	3a (%)	3a' (%)
**1**	**PCy_3 (20)**	**98**	**0**	12	Davephos (20)	0	0
2	–	2	0	13	Xantphos (10)	0	0
3	PPh_3 (20)	19	0	14	(*R*)-BINAP (10)	0	0
4	PPh_2Cy (20)	35	0	15	IPr·HCl (10)	2	0
5	$PPhCy_2$ (20)	72	0	16	IMes·HCl (10)	4	0
6	P^nBu_3 (20)	5	0	17	bpy (10)	0	0
7	P^tBu_3 (20)	8	0	18	dtbbpy (10)	0	0
8	$PCyp_3$ (20)	73	0	19	phen (10)	0	0
9	dppe (10)	0	0	20	neo (10)	0	0
10	dcype (10)	32	0	21	**L** (10)	0	0
11	XPhos (20)	4	0				

[a] Conditions: **1a** (0.2 mmol), **2a** (2.0 equiv.), $Ni(cod)_2$ (10 mol%), ligand (10 or 20 mol%), $PhB(OH)_2$ (25 mol%), KO^tBu (25 mol%), toluene (0.3 mL), 120 °C, 20 h. Yields were determined by ^1H NMR spectroscopy using 1,1,2,2-tetrachloroethane as the internal standard. Cyp=cyclopentyl group.

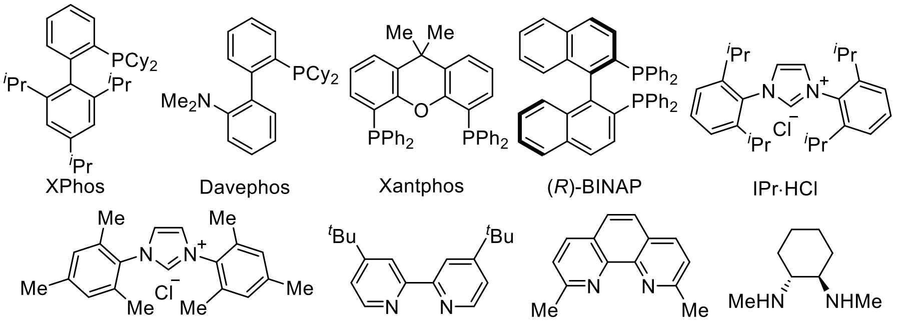

XPhos, Davephos, Xantphos, (*R*)-BINAP, IPr·HCl, IMes·HCl, dtbbpy, neo, **L**

Supplementary Table 5 Optimization of Solvent and Concentration[a]

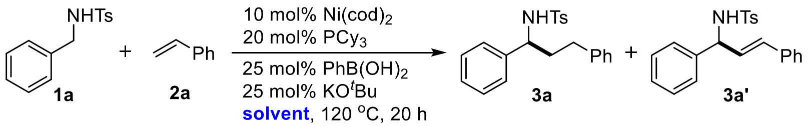

entry	solvent (mL)	**3a** (%)	**3a'** (%)
**1**	**toluene (0.3)**	**98**	**0**
2	hexane (0.3)	91	0
3	dioxane (0.3)	86	0
4	THF (0.3)	71	0
5	toluene (0.2)	91	0
6	toluene (0.5)	97	0

[a] Conditions: **1a** (0.2 mmol), **2a** (2.0 equiv.), $Ni(cod)_2$ (10 mol%), PCy_3 (20 mol%), $PhB(OH)_2$ (25 mol%), KO*ᵗ*Bu (25 mol%), solvent, 120 °C, 20 h. Yields were determined by ^1H NMR spectroscopy using 1,1,2,2-tetrachloroethane as the internal standard.

Supplementary Table 6 Optimization of Temperature[a]

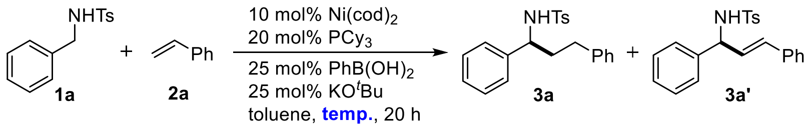

entry	temp. (°C)	**3a** (%)	**3a'** (%)
**1**	**120**	**98**	**0**
2	100	86	0
3	140	88	0

[a] Conditions: **1a** (0.2 mmol), **2a** (2.0 equiv.), $Ni(cod)_2$ (10 mol%), PCy_3 (20 mol%), $PhB(OH)_2$ (25 mol%), KO^tBu (25 mol%), toluene (0.3 mL), temp., 20 h. Yields were determined by ^1H NMR spectroscopy using 1,1,2,2-tetrachloroethane as the internal standard.

Supplementary Table 7 Optimization of the Amount of Styrene, KO^tBu and $PhB(OH)_2$[a]

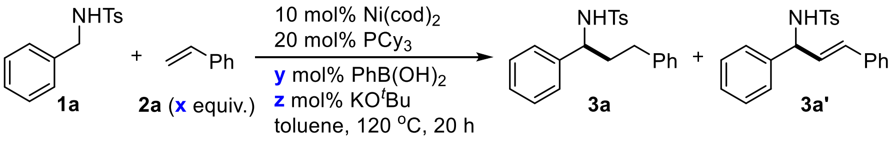

entry	**2a** (**x** equiv.)	$PhB(OH)_2$ (**y** mol%)	KO^tBu (**z** mol%)	**3a** (%)	**3a'** (%)
**1**	**2.0**	**25**	**25**	**98**	**0**
2	1.5	25	25	86	0
3	3.0	25	25	97	0
4	2.0	10	25	90	0
5	2.0	40	25	96	0
6	2.0	25	10	38	0
7	2.0	25	40	98	0
8	2.0	25	100	97	0

[a] Conditions: **1a** (0.2 mmol), **2a** (x equiv.), $Ni(cod)_2$ (10 mol%), PCy_3 (20 mol%), $PhB(OH)_2$ (y mol%), KO^tBu (z mol%), toluene (0.3 mL), 120 °C, 20 h. Yields were determined by ^1H NMR spectroscopy using 1,1,2,2-tetrachloroethane as the internal standard.

Supplementary Table 8 Optimization of the Amount of Catalyst and Ligand[a]

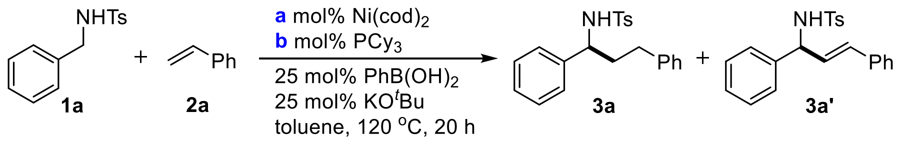

entry	$Ni(cod)_2$ (**a** mol%)	PCy_3 (**b** mol%)	**3a** (%)	**3a'** (%)
1	10	20	98	0
2	0	20	0	0
3	10	10	92	0
4	10	30	84	0
5	5	10	98 (88[b])	0
**6**	**2.5**	**5**	**quant. (93[b])**	**0**
7	1	2	91[c]	0

[a] Conditions: **1a** (0.2 mmol), **2a** (2.0 equiv.), $Ni(cod)_2$ (a mol%), PCy_3 (b mol%), $PhB(OH)_2$ (25 mol%), KO^{t}Bu (25 mol%), toluene (0.3 mL), 120 °C, 20 h. Yields were determined by ^{1}H NMR spectroscopy using 1,1,2,2-tetrachloroethane as the internal standard. [b] Isolated yield. [c] Reaction time: 38 h.

Supplementary Table 9 Optimization of Protecting Groups[a]

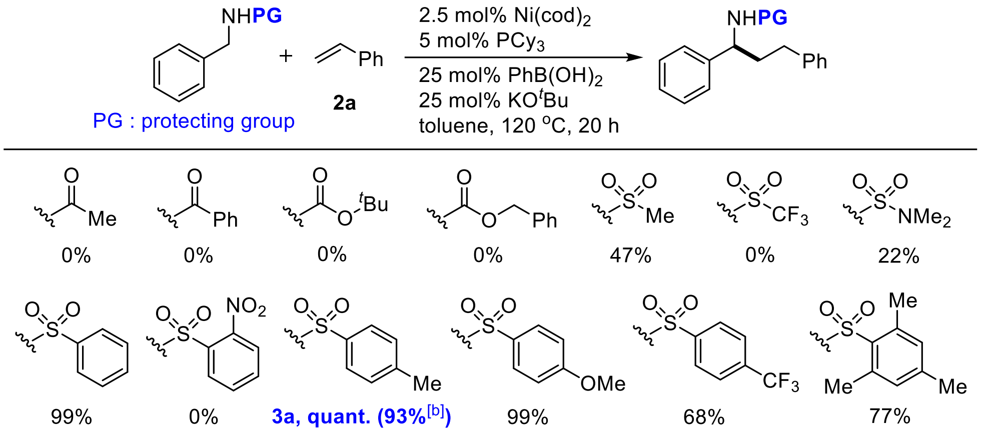

[a] Conditions: protected amine (0.2 mmol), **2a** (2.0 equiv.), $Ni(cod)_2$ (2.5 mol%), PCy_3 (5 mol%), $PhB(OH)_2$ (25 mol%), KO^{t}Bu (25 mol%), toluene (0.3 mL), 120 °C, 20 h. Yields were determined by ^{1}H NMR spectroscopy using 1,1,2,2-tetrachloroethane as the internal standard. [b] Isolated yield.

Supplementary Table 10 Condition Optimization of Sulfonyl Amine with Unactivated Alkene[a]

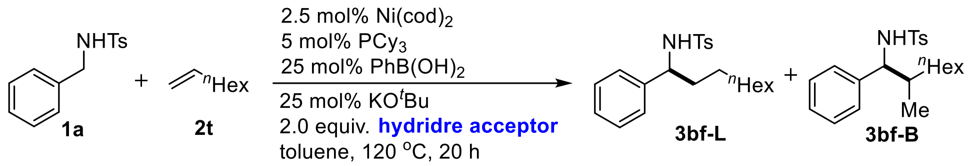

entry	hydride acceptor	**3bf** (%)	entry	hydride acceptor	**3bf** (%)
1	–	4	7	acetone	5
2	**HA_1**	0	8	**HA_5**	7
3	**HA_2**	3	9	**HA_6**	0
4	**HA_3**	5	10	**HA_7**	22 (1:1.3[e])
5	**HA_4**	6	**11[b]**	**pivaldehyde**	**81 (88[d], 1:1.6[e])**
6	pivaldehyde	57 (1:1.3[e])	12[c]	pivaldehyde	72 (1:2.2[e])

[a] Conditions: **1a** (0.2 mmol), **2t** (2.0 equiv.), $Ni(cod)_2$ (2.5 mol%), PCy_3 (5 mol%), $PhB(OH)_2$ (25 mol%), KO^{t}Bu (25 mol%), hydride acceptor (2.0 equiv.), toluene (0.3 mL), 120 °C, 20 h. Yields were determined by ^{1}H NMR spectroscopy using 1,1,2,2-tetrachloroethane as the internal standard. [b] $Ni(cod)_2$ (5 mol%) and PCy_3 (10 mol%) were used. [c] $Ni(cod)_2$ (10 mol%) and PCy_3 (20 mol%) were used. [d] Isolated yield. [e] The ratio of linear product to branched product (*l*/*b*) was determined by ^{1}H NMR spectroscopy.

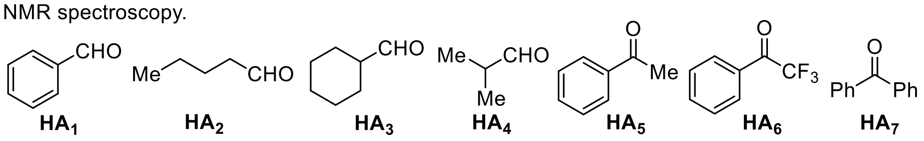

Supplementary Table 11 Condition Optimization of Enantioselective Hydroalkylation of Olefins with *N*-Sulfonyl Amines[a]

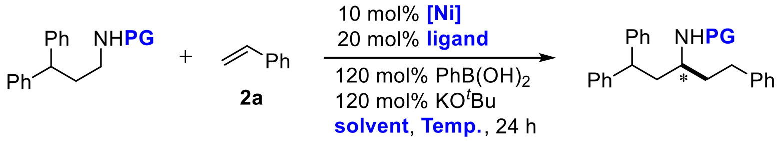

entry	PG	[Ni]	ligand	solvent (mL)	Temp. (°C)	yield (%)	er
1	Ts	Ni(cod)$_2$	(*R*)-BI-DIME	toluene	120	36[b]	57.2:42.8
2	Ts	NiBr$_2$	(*R*)-BI-DIME	toluene	120	98	75.2:24.8
3	Ts	NiBr$_2$·dme	(*R*)-BI-DIME	toluene	120	99	79.6:20.4
4	Ts	NiBr$_2$·dme	**L$_1$**	toluene	120	38	59.5:40.5
5	Ts	NiBr$_2$·dme	**L$_2$**	toluene	120	93	77.3:22.7
6	Ts	NiBr$_2$·dme	**L$_3$**	toluene	120	n.r.	n.d.
7	Ts	NiBr$_2$·dme	**L$_4$**	toluene	120	56	72.5:27.5
8	Ts	NiBr$_2$·dme	**L$_5$**	toluene	120	n.r.[c]	n.d.
9	Ts	NiBr$_2$·dme	**L$_6$**	toluene	120	43	55.5:44.5
10	Ts	NiBr$_2$·dme	**L$_7$**	toluene	120	96	54.0:46.0
11	Ts	NiBr$_2$·dme	**L$_8$**	toluene	120	n.r.	n.d.
12	Ts	NiBr$_2$·dme	**L$_9$**	toluene	120	34	52.5:47.5
13	Ts	NiBr$_2$·dme	**L$_{10}$**	toluene	120	77	68.8:31.2
14	Mts	NiBr$_2$·dme	(*R*)-BI-DIME	toluene	120	99	83.6:17.4
15	Mts	NiBr$_2$·dme	(*R*)-BI-DIME	toluene	100	98	86.3:13.7
16	Mts	NiBr$_2$·dme	(*R*)-BI-DIME	toluene	80	96	88.2:11.8
17	Mts	NiBr$_2$·dme	(*R*)-BI-DIME	dioxane	80	21	86.7:13.3
18	Mts	NiBr$_2$·dme	(*R*)-BI-DIME	THF	80	16	74.6:25.4
19	Mts	NiBr$_2$·dme	(*R*)-BI-DIME	anisole	80	98	89.6:10.4
**20**	**Mts**	**NiBr$_2$·dme**	**(*R*)-BI-DIME**	**anisole**	**60**	**68 (55[d,e])**	**92.0:8.0**

[a] Conditions: N-sulfonyl amine (0.1 mmol), **2a** (2.0 equiv.), [Ni] (10 mol%), ligand (20 mol%), $PhB(OH)_2$ (120 mol%), KO^{t}Bu (120 mol%), solvent (0.2 mL), 24 h. Yields were determined by ^{1}H NMR spectroscopy using 1,1,2,2-tetrachloroethane as the internal standard. Enantiomeric ratio (er) were determined by SFC analysis. [b] $PhB(OH)_2$ (100 mol%) and KO^{t}Bu (100 mol%) were used. [c] Ligand (10 mol%) was used. [d] Reaction time: 72 h. [e] Isolated yield.

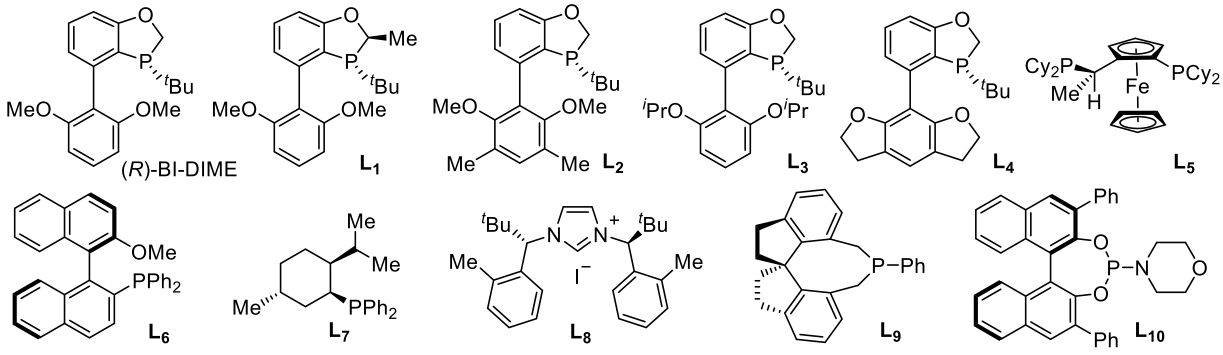

2.2 Synthesis of Starting Materials

2.2.1 Synthesis of *N*-Sulfonyl Amines

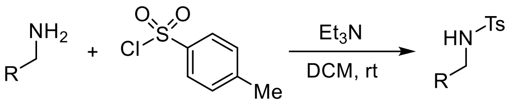

Method A: Amine (10.0 mmol, 1.0 equiv.) was weighed directly into a dry round-bottomed flask. Then, DCM (40 mL) was added to dissolve the amine, following by Et_3N (20.0 mmol, 2.8 mL, 2.0 equiv.) and TsCl (11.0 mmol, 2.1 g, 1.1 equiv.). Monitored the reaction mixture by TLC, after all of the amine was consumed, directly concentrated under reduced pressure. Purification by flash chromatography provided desired product (85-99% yield).

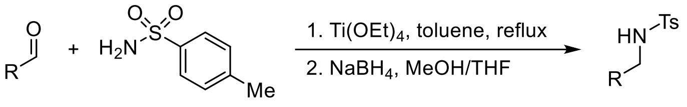

Method B: To a solution of aldehyde (5.0 mmol, 1.0 equiv.) in toluene (10 mL), $TsNH_2$ (7.5 mmol, 1.3 g, 1.5 equiv.) and $Ti(OEt)_4$ (10.0 mmol, 4.2 mL, 2.0 equiv.) were added and the mixture refluxed for 4 h. Then, after cooling at room temperature, the solvent was evaporated under reduced pressure. The residue was dissolved in MeOH/THF (1:1, 20 mL) and $NaBH_4$ (20.0 mmol, 0.8 g, 4.0 equiv.) was slowly added at 0 ºC. After 4 hours stirring at room temperature, water (1.0 mL) was slowly added at 0 ºC and the solvent was evaporated under reduced pressure. Purification by flash chromatography provided desired product (80-95% yield).

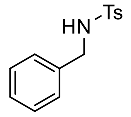

***N*-Benzyl-4-methylbenzenesulfonamide (1a)**

Following the **Method A**, the title compound was isolated as white solid. Known compound.[1] **^{1}H NMR (500 MHz, $CDCl_3$)** δ 7.75 (d, *J* = 8.5 Hz, 2 H), 7.31–7.23 (m, 5 H), 7.20–7.18 (m, 2 H), 4.77 (t, *J* = 6.5 Hz, 1 H), 4.11 (d, *J* = 6.5 Hz, 2 H), 2.43 (s, 3 H). **^{13}C NMR (125 MHz, $CDCl_3$)** δ 143.5, 136.8, 136.2, 129.7, 128.7, 127.9, 127.8, 127.2, 47.2, 21.5.

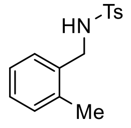

4-Methyl-*N*-(2-methylbenzyl)benzenesulfonamide (1b)

Following the **Method A**, the title compound was isolated as white solid. Known compound.[2] **^{1}H NMR (500 MHz, $CDCl_3$)** δ 7.76 (d, *J* = 8.0 Hz, 2 H), 7.31 (d, *J* = 8.0 Hz, 2 H), 7.19–7.14 (m, 1 H), 7.12–7.08 (m, 3 H), 4.53 (t, *J* = 6.0 Hz, 1 H), 4.08 (d, *J* = 6.0 Hz, 2 H), 2.44 (s, 3 H), 2.24 (s, 3 H). **^{13}C NMR (125 MHz, $CDCl_3$)** δ 143.5, 136.7, 136.6, 133.9, 130.6, 129.7, 128.8, 128.2, 127.2, 126.2, 45.4, 21.5, 18.8.

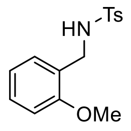

***N*-(2-Methoxybenzyl)-4-methylbenzenesulfonamide (1c)**

Following the **Method A**, the title compound was isolated as white solid. Known compound.[3] **^{1}H NMR (500 MHz, $CDCl_3$)** δ 7.66 (d, *J* = 8.0 Hz, 2 H), 7.20–7.17 (m, 3 H), 7.06 (d, *J* = 7.5 Hz, 1 H), 6.80 (t, *J* = 7.5 Hz, 1 H), 6.72 (d, *J* = 8.0 Hz, 1 H), 5.15 (t, *J* = 6.0 Hz, 1 H), 4.13 (d, *J* = 6.5 Hz, 2 H), 3.73 (s, 3 H), 2.38 (s, 3 H). **^{13}C NMR (125 MHz, $CDCl_3$)** δ 157.2, 143.0, 137.2, 129.7, 129.3, 129.2, 127.0, 124.3, 120.5, 110.0, 55.1, 43.9, 21.4.

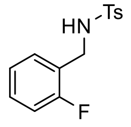

***N*-(2-Fluorobenzyl)-4-methylbenzenesulfonamide (1d)**

Following the **Method A**, the title compound was isolated as white solid. Known compound.[4] **^{1}H NMR (500 MHz, $CDCl_3$)** δ 7.71 (d, *J* = 8.5 Hz, 2 H), 7.26–7.19 (m, 4 H), 7.05–7.02 (m, 1 H), 6.96–6.92 (m, 1 H), 4.95 (t, *J* = 6.5 Hz, 1 H), 4.19 (d, *J* = 6.5 Hz, 2 H), 2.40 (s, 3 H). **^{13}C NMR (125 MHz, $CDCl_3$)** δ 160.6 (d, *J* = 244.9 Hz), 143.4, 136.8, 130.1 (d, *J* = 4.0 Hz), 129.62 (d, *J* = 8.0 Hz), 129.61, 127.1, 124.3 (d, *J* = 3.6 Hz), 123.5 (d, *J* = 14.4 Hz), 115.3 (d, *J* = 21.0 Hz), 41.3 (d, *J* = 3.9 Hz), 21.5. **^{19}F NMR (470 MHz, $CDCl_3$)** δ -118.8.

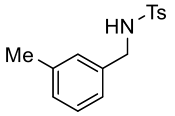

4-Methyl-*N*-(3-methylbenzyl)benzenesulfonamide (1e)

Following the **Method A**, the title compound was isolated as white solid. Known compound.[2] **^{1}H NMR (500 MHz, $CDCl_3$)** δ 7.75 (d, *J* = 8.0 Hz, 2 H), 7.30 (d, *J* = 8.5 Hz, 2 H), 7.17–7.13 (m, 1 H), 7.07–7.04 (m, 1 H), 6.97 (m, 2 H), 4.73 (t, *J* = 6.0 Hz, 1 H), 4.07 (d, *J* = 6.0 Hz, 2 H), 2.43 (s, 3 H), 2.27 (s, 3 H). **^{13}C NMR (125 MHz, $CDCl_3$)** δ 143.4, 138.4, 136.9, 136.1, 129.7, 128.574, 128.568, 128.5, 127.2, 124.8, 47.2, 21.5, 21.2.

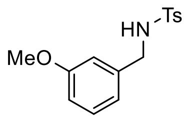

***N*-(3-Methoxybenzyl)-4-methylbenzenesulfonamide (1f)**

Following the **Method A**, the title compound was isolated as white solid. Known compound.[5] **^{1}H NMR (500 MHz, $CDCl_3$)** δ 7.75 (d, *J* = 8.0 Hz, 2 H), 7.30 (d, *J* = 8.5 Hz, 2 H), 7.17 (t, *J* = 8.0 Hz, 1 H), 6.79–6.75 (m, 2 H), 6.720–6.716 (m, 1 H), 4.80 (t, *J* = 6.0 Hz, 1 H), 4.09 (d, *J* = 6.0 Hz, 2 H), 3.73 (s, 3 H), 2.43 (s, 3 H). **^{13}C NMR (125 MHz, $CDCl_3$)** δ 159.8, 143.5, 137.8, 136.8, 129.71, 129.67, 127.2, 120.0, 113.6, 113.1, 55.2, 47.2, 21.5.

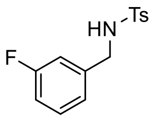

***N*-(3-Fluorobenzyl)-4-methylbenzenesulfonamide (1g)**

Following the **Method A**, the title compound was isolated as white solid. Known compound.[6] **^{1}H NMR (500 MHz, $CDCl_3$)** δ 7.74 (d, *J* = 8.5 Hz, 2 H), 7.29 (d, *J* = 8.0 Hz, 2 H), 7.25–7.20 (m, 1 H), 6.98 (d, *J* = 7.5 Hz, 1 H), 6.94–6.88 (m, 2 H), 5.00 (t, *J* = 6.5 Hz, 1 H), 4.11 (d, *J* = 6.5 Hz, 2 H), 2.43 (s, 3 H). **^{13}C NMR (125 MHz, $CDCl_3$)** δ 162.8 (d, *J* = 245.3 Hz), 143.7, 138.9 (d, *J* = 7.3 Hz), 136.7, 130.2 (d, *J* = 8.1 Hz), 129.7, 127.1, 123.3 (d, *J* = 2.9 Hz), 114.8, 114.6 (d, *J* = 1.9 Hz), 46.6 (d, *J* = 1.9 Hz), 21.5. **^{19}F NMR (470 MHz, $CDCl_3$)** δ -112.6.

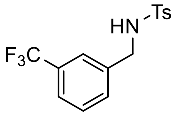

4-Methyl-*N*-(3-(trifluoromethyl)benzyl)benzenesulfonamide (1h)

Following the **Method A**, the title compound was isolated as white solid. Known compound.[7] **^{1}H NMR (500 MHz, $CDCl_3$)** δ 7.72 (d, *J* = 8.5 Hz, 2 H), 7.49 (d, *J* = 7.0 Hz, 1 H), 7.43–7.37 (m, 3 H), 7.27 (d, *J* = 8.0 Hz, 2 H), 5.07 (t, *J* = 6.5 Hz, 1 H), 4.20 (d, *J* = 6.5 Hz, 2 H), 2.42 (s, 3 H). **^{13}C NMR (125 MHz, $CDCl_3$)** δ 143.8, 137.4, 136.7, 131.2, 130.9 (q, *J* = 32.3 Hz), 129.8, 129.1, 127.1, 124.6 (q, *J* = 3.8 Hz), 124.5 (q, *J* = 3.8 Hz), 123.8 (q, *J* = 270.6 Hz), 46.7, 21.5. **^{19}F NMR (470 MHz, $CDCl_3$)** δ -62.7.

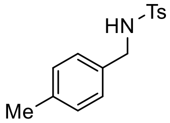

4-Methyl-*N*-(4-methylbenzyl)benzenesulfonamide (1i)

Following the **Method A**, the title compound was isolated as white solid. Known compound.[8] **^{1}H NMR (500 MHz, $CDCl_3$)** δ 7.76 (d, *J* = 8.0 Hz, 2 H), 7.31 (d, *J* = 8.0 Hz, 2 H), 7.09–7.06 (m, 4 H), 4.59 (t, *J* = 5.5 Hz, 1 H), 4.07 (t, *J* = 6.5 Hz, 2 H), 2.44 (s, 3 H), 2.31 (s, 3 H). **^{13}C NMR (125 MHz, $CDCl_3$)** δ 143.5, 137.7, 136.8, 133.2, 129.7, 129.3, 127.8, 127.2, 47.0, 21.5, 21.1.

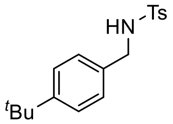

***N*-(4-(*tert*-Butyl)benzyl)-4-methylbenzenesulfonamide (1j)**

Following the **Method A**, the title compound was isolated as white solid. Known compound.[9] **^{1}H NMR (500 MHz, $CDCl_3$)** δ 7.74 (d, *J* = 8.5 Hz, 2 H), 7.29–7.27 (m, 4 H), 7.11 (d, *J* = 8.5 Hz, 2 H), 4.74 (t, *J* = 6.0 Hz, 1 H), 4.09 (d, *J*

= 6.5 Hz, 2 H), 2.42 (s, 3 H), 1.28 (s, 9 H). **^{13}C NMR (125 MHz, $CDCl_3$)** δ 150.9, 143.4, 136.9, 133.2, 129.6, 127.6, 127.2, 125.5, 46.9, 34.5, 31.2, 21.5.

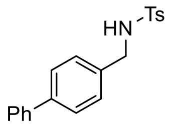

***N*-([1,1'-Biphenyl]-4-ylmethyl)-4-methylbenzenesulfonamide (1k)**

Following the **Method A**, the title compound was isolated as white solid. Known compound.[8] **^{1}H NMR (500 MHz, $CDCl_3$)** δ 7.76 (d, *J* = 8.0 Hz, 2 H), 7.54–7.52 (m, 2 H), 7.48 (d, *J* = 8.0 Hz, 2 H), 7.44–7.41 (m, 2 H), 7.36–7.32 (m, 1 H), 7.29 (d, *J* = 8.0 Hz, 2 H), 7.26 (d, *J* = 7.5 Hz, 2 H), 4.85 (t, *J* = 6.0 Hz, 1 H), 4.16 (d, *J* = 6.5 Hz, 2 H), 2.42 (s, 3 H). **^{13}C NMR (125 MHz, $CDCl_3$)** δ 143.5, 140.8, 140.5, 136.9, 135.3, 129.7, 128.8, 128.3, 127.4, 127.3, 127.2, 127.0, 46.9, 21.5.

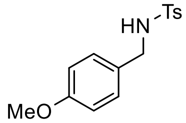

***N*-(4-Methoxybenzyl)-4-methylbenzenesulfonamide (1l)**

Following the **Method A**, the title compound was isolated as white solid. Known compound.[8] **^{1}H NMR (500 MHz, $CDCl_3$)** δ 7.75 (d, *J* = 8.5 Hz, 2 H), 7.30 (d, *J* = 8.0 Hz, 2 H), 7.10 (d, *J* = 8.5 Hz, 2 H), 6.79 (d, *J* = 8.5 Hz, 2 H), 4.70 (t, *J* = 6.0 Hz, 1 H), 4.04 (d, *J* = 6.5 Hz, 2 H), 3.77 (s, 3 H), 2.43 (s, 3 H). **^{13}C NMR (125 MHz, $CDCl_3$)** δ 159.3, 143.4, 136.9, 129.7, 129.2, 128.2, 127.2, 114.0, 55.2, 46.7, 21.5.

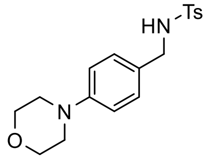

4-Methyl-*N*-(4-morpholinobenzyl)benzenesulfonamide (1m)

Following the **Method B**, the title compound was isolated as white solid. Known compound.[10] **^{1}H NMR (500 MHz, $CDCl_3$)** δ 7.76 (d, *J* = 8.5 Hz, 2 H), 7.31 (d, *J* = 8.0 Hz, 2 H), 7.09 (d, *J* = 8.5 Hz, 2 H), 6.81 (d, *J* = 8.5 Hz, 2 H), 4.55 (t, *J* = 6.0 Hz, 1 H), 4.04 (d, *J* = 6.0 Hz, 2 H), 3.84 (t, *J* = 5.0 Hz, 4 H), 3.11 (t, *J* = 5.0 Hz, 4 H), 2.44 (s, 3 H). **^{13}C NMR (125 MHz, $CDCl_3$)** δ 151.0, 143.4, 136.9, 129.7, 129.0, 127.3, 127.2, 115.6, 66.8, 49.1, 46.8, 21.5.

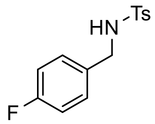

***N*-(4-Fluorobenzyl)-4-methylbenzenesulfonamide (1n)**

Following the **Method A**, the title compound was isolated as white solid. Known compound.[4] **^{1}H NMR (500 MHz, CDCl$_3$)** δ 7.73 (d, *J* = 8.0 Hz, 2 H), 7.29 (d, *J* = 8.0 Hz, 2 H), 7.18–7.15 (m, 2 H), 6.96–6.92 (m, 2 H), 4.94 (t, *J* = 6.0 Hz, 1 H), 4.08 (d, *J* = 6.5 Hz, 2 H), 2.43 (s, 3 H). **^{13}C NMR (125 MHz, CDCl$_3$)** δ 162.3 (d, *J* = 244.9 Hz), 143.6, 136.8, 132.1 (d, *J* = 3.1 Hz), 129.7, 129.6 (d, *J* = 8.1 Hz), 127.1, 115.5 (d, *J* = 21.5 Hz), 46.5, 21.5. **^{19}F NMR (470 MHz, CDCl$_3$)** δ -114.3.

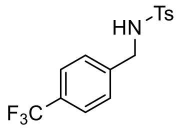

4-Methyl-*N*-(4-(trifluoromethyl)benzyl)benzenesulfonamide (1o)

Following the **Method A**, the title compound was isolated as white solid. Known compound.[8] **^{1}H NMR (500 MHz, CDCl$_3$)** δ 7.71 (d, *J* = 8.5 Hz, 2 H), 7.50 (d, *J* = 8.0 Hz, 2 H), 7.31 (d, *J* = 8.0 Hz, 2 H), 7.27 (d, *J* = 8.0 Hz, 2 H), 5.14 (t, *J* = 6.5 Hz, 1 H), 4.19 (d, *J* = 6.0 Hz, 2 H), 2.42 (s, 3 H). **^{13}C NMR (125 MHz, CDCl$_3$)** δ 143.8, 140.4, 136.7, 130.0 (q, *J* = 32.3 Hz), 129.7, 128.0, 127.1, 125.5 (q, *J* = 3.8 Hz), 123.9 (q, *J* = 270.4 Hz), 46.6, 21.5. **^{19}F NMR (470 MHz, CDCl$_3$)** δ -62.6.

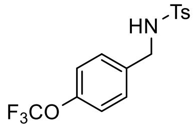

4-Methyl-*N*-(4-(trifluoromethoxy)benzyl)benzenesulfonamide (1p)

Following the **Method A**, the title compound was isolated as white solid. Known compound.[5] **^{1}H NMR (500 MHz, CDCl$_3$)** δ 7.71 (d, *J* = 8.5 Hz, 2 H), 7.27 (d, *J* = 8.5 Hz, 2 H), 7.22 (d, *J* = 9.0 Hz, 2 H), 7.09 (d, *J* = 8.5 Hz, 2 H), 5.04 (t, *J* = 6.5 Hz, 1 H), 4.13 (d, *J* = 6.5 Hz, 2 H), 2.42 (s, 3 H). **^{13}C NMR (125 MHz, CDCl$_3$)** δ 148.7 (q, *J* = 1.9 Hz), 143.7, 136.8, 135.1, 129.7, 129.3, 127.1, 121.1, 120.4 (q, *J* = 255.6 Hz), 46.4, 21.5. **^{19}F NMR (470 MHz, CDCl$_3$)** δ -57.9.

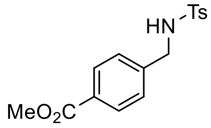

Methyl 4-(((4-methylphenyl)sulfonamido)methyl)benzoate (1q)

Following the **Method A**, the title compound was isolated as white solid. Known compound.[11] **^{1}H NMR (500 MHz, CDCl$_3$)** δ 7.92 (d, *J* = 8.5 Hz, 2 H), 7.74 (d, *J* = 8.5 Hz, 2 H), 7.30–7.26 (m, 4 H), 5.04 (t, *J* = 6.5 Hz, 1 H), 4.17 (d, *J* = 6.5 Hz, 2 H), 3.90 (s, 3 H), 2.43 (s, 3 H). **^{13}C NMR (125 MHz, CDCl$_3$)** δ 166.7, 143.7, 141.5, 136.7, 129.9, 129.8, 129.6, 127.6, 127.1, 52.1, 46.8, 21.5.

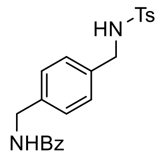

***N*-(4-(((4-Methylphenyl)sulfonamido)methyl)benzyl)benzamide (1r)**

Following the **Method A**, the title compound was isolated as white solid. Known compound.[12] **^{1}H NMR (500 MHz, $CDCl_3$)** δ 7.78–7.75 (m, 4 H), 7.50 (t, *J* = 7.5 Hz, 1 H), 7.42 (t, *J* = 7.5 Hz, 2 H), 7.32–7.26 (m, 4 H), 7.18 (d, *J* = 7.5 Hz, 2 H), 6.47 (s, 1 H), 4.74 (t, *J* = 6.5 Hz, 1 H), 4.59 (d, *J* = 5.5 Hz, 2 H), 4.09 (d, *J* = 6.5 Hz, 2 H), 2.43 (s, 3 H). **^{13}C NMR (125 MHz, $CDCl_3$)** δ 167.3, 143.6, 138.1, 136.8, 135.7, 134.2, 131.6, 129.8, 128.6, 128.3, 128.2, 127.2, 126.9, 46.9, 43.7, 21.5.

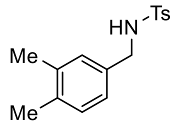

***N*-(3,4-Dimethylbenzyl)-4-methylbenzenesulfonamide (1s)**

Following the **Method A**, the title compound was isolated as white solid. **^{1}H NMR (500 MHz, $CDCl_3$)** δ 7.76–7.74 (m, 2 H), 7.30 (d, *J* = 8.0 Hz, 2 H), 7.02 (d, *J* = 7.5 Hz, 1 H), 6.91–6.89 (m, 2 H), 4.65 (t, *J* = 6.0 Hz, 1 H), 4.04 (d, *J* = 6.0 Hz, 2 H), 2.43 (s, 3 H), 2.20 (s, 3 H), 2.17 (s, 3 H). **^{13}C NMR (125 MHz, $CDCl_3$)** δ 143.4, 136.91, 136.89, 136.3, 133.5, 129.8, 129.7, 129.2, 127.2, 125.3, 47.0, 21.5, 19.6, 19.4. **HRMS** (ESI) m/z calcd. For $C_{16}H_{19}NNaO_2S$ $[M+Na]^+$: 312.1029, found: 312.1027.

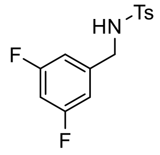

***N*-(3,5-Difluorobenzyl)-4-methylbenzenesulfonamide (1t)**

Following the **Method A**, the title compound was isolated as white solid. **^{1}H NMR (500 MHz, $CDCl_3$)** δ 7.73 (d, *J* = 8.5 Hz, 2 H), 7.30 (d, *J* = 8.0 Hz, 2 H), 6.75–6.71 (m, 2 H), 6.69–6.64 (m, 1 H), 5.13 (t, *J* = 6.5 Hz, 1 H), 4.10 (d, *J* = 6.5 Hz, 2 H), 2.43 (s, 3 H). **^{13}C NMR (125 MHz, $CDCl_3$)** δ 163.0 (dd, J_1 = 247.9 Hz, J_2 = 12.6 Hz), 143.9, 140.5 (t, *J* = 8.9 Hz), 136.6, 129.8, 127.1, 110.5 (dd, J_1 = 19.6 Hz, J_2 = 5.8 Hz), 103.1 (t, *J* = 25.1 Hz), 46.3 (t, *J* = 2.0 Hz), 21.5. **^{19}F NMR (470 MHz, $CDCl_3$)** δ -109.2. **HRMS** (ESI) m/z calcd. For $C_{14}H_{14}F_2NO_2S$ $[M+H]^+$: 298.0708, found: 298.0711.

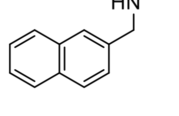

4-Methyl-*N*-(naphthalen-2-ylmethyl)benzenesulfonamide (1u)

Following the **Method A**, the title compound was isolated as white solid. Known compound.[8] **^{1}H NMR (500 MHz, $CDCl_3$)** δ 7.79–7.70 (m, 5 H), 7.59 (s, 1 H), 7.46–7.44 (m, 2 H), 7.29 (d, J = 8.0 Hz, 1 H), 7.24 (d, J = 7.5 Hz, 2 H), 4.90 (t, J = 6.0 Hz, 1 H), 4.27 (d, J = 6.5 Hz, 2 H), 2.39 (s, 3 H). **^{13}C NMR (125 MHz, $CDCl_3$)** δ 143.5, 136.9, 133.6, 133.1, 132.8, 129.7, 128.5, 127.7, 127.6, 127.2, 126.7, 126.3, 126.1, 125.6, 47.4, 21.5.

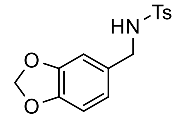

***N*-(Benzo[*d*][1,3]dioxol-5-ylmethyl)-4-methylbenzenesulfonamide (1v)**

Following the **Method A**, the title compound was isolated as white solid. Known compound.[13] **^{1}H NMR (500 MHz, $CDCl_3$)** δ 7.74 (d, J = 8.5 Hz, 2 H), 7.31 (d, J = 8.0 Hz, 2 H), 6.69–6.66 (m, 2 H), 6.64–6.62 (m, 1 H), 5.92 (s, 2 H), 4.70 (t, J = 6.0 Hz, 1 H), 4.01 (d, J = 6.0 Hz, 2 H), 2.44 (s, 3 H). **^{13}C NMR (125 MHz, $CDCl_3$)** δ 147.9, 147.3, 143.5, 136.8, 130.0, 129.7, 127.1, 121.3, 108.4, 108.2, 101.1, 47.1, 21.5.

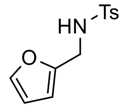

***N*-(Furan-2-ylmethyl)-4-methylbenzenesulfonamide (1w)**

Following the **Method A**, the title compound was isolated as white solid. Known compound.[2] **^{1}H NMR (500 MHz, $CDCl_3$)** δ 7.71 (d, J = 8.5 Hz, 2 H), 7.27 (d, J = 8.0 Hz, 2 H), 7.24 (d, J = 1.0 Hz, 1 H), 6.21 (dd, J_1 = 3.0 Hz, J_2 = 2.0 Hz, 1 H), 6.09 (d, J = 3.0 Hz, 1 H), 4.83 (t, J = 6.0 Hz, 1 H), 4.17 (d, J = 7.5 Hz, 2 H), 2.42 (s, 3 H). **^{13}C NMR (125 MHz, $CDCl_3$)** δ 149.5, 143.5, 142.5, 136.8, 129.6, 127.1, 110.4, 108.2, 40.1, 21.5.

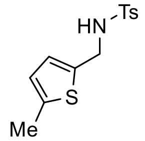

4-Methyl-*N*-((5-methylthiophen-2-yl)methyl)benzenesulfonamide (1x)

Following the **Method B**, the title compound was isolated as white solid. Known compound.[14] **^{1}H NMR (500 MHz, $CDCl_3$)** δ 7.74 (d, J = 8.5 Hz, 2 H), 7.30 (d, J = 8.0 Hz, 2 H), 6.61 (d, J = 3.0 Hz, 1 H), 6.51–6.50 (m, 1 H), 4.77–4.76 (m, 1 H), 4.22 (d, J = 6.0 Hz, 2 H), 2.43 (s, 3 H), 2.39 (s, 3 H). **^{13}C NMR (125 MHz, $CDCl_3$)** δ 143.5, 140.6, 136.8, 136.3, 129.7, 127.2, 126.5, 124.8, 42.3, 21.5, 15.3.

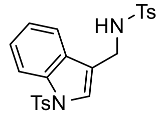

4-Methyl-*N*-((1-tosyl-1*H*-indol-3-yl)methyl)benzenesulfonamide (1y)

Following the **Method B**, the title compound was isolated as white solid. **^{1}H NMR (500 MHz, CDCl$_3$)** δ 7.91 (d, *J* = 8.5 Hz, 1 H), 7.71 (d, *J* = 8.0 Hz, 4 H), 7.42 (d, *J* = 8.0 Hz, 1 H), 7.35 (s, 1 H), 7.30 (t, *J* = 8.0 Hz, 1 H), 7.26–7.18 (m, 5 H), 4.67 (t, *J* = 5.5 Hz, 1 H), 4.23 (d, *J* = 6.0 Hz, 2 H), 2.42 (s, 3 H), 2.33 (s, 3 H). **^{13}C NMR (125 MHz, CDCl$_3$)** δ 145.1, 143.8, 136.4, 135.14, 135.06, 129.9, 129.7, 129.0, 127.1, 126.8, 125.1, 124.5, 123.3, 119.6, 117.3, 113.6, 38.6, 21.6, 21.5. **HRMS** (ESI) m/z calcd. For $C_{23}H_{22}N_2NaO_4S_2$ [M+Na]$^+$: 477.0913, found: 477.0915.

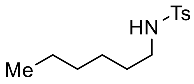

***N*-Hexyl-4-methylbenzenesulfonamide (1z)**

Following the **Method A**, the title compound was isolated as white solid. Known compound.[8] **^{1}H NMR (500 MHz, CDCl$_3$)** δ 7.76 (d, *J* = 8.5 Hz, 2 H), 7.31 (d, *J* = 8.0 Hz, 2 H), 4.64 (t, *J* = 6.0 Hz, 1 H), 2.92 (dd, J_1 = 13.5 Hz, J_2 = 7.0 Hz, 2 H), 2.43 (s, 3 H), 1.47–1.41 (m, 2 H), 1.28–1.15 (m, 6 H), 0.84 (t, *J* = 6.6 Hz, 3 H). **^{13}C NMR (125 MHz, CDCl$_3$)** δ 143.3, 137.0, 129.6, 127.1, 43.2, 31.2, 29.4, 26.1, 22.4, 21.5, 13.9.

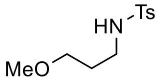

***N*-(3-Methoxypropyl)-4-methylbenzenesulfonamide (1aa)**

Following the **Method A**, the title compound was isolated as colorless oil. **^{1}H NMR (500 MHz, CDCl$_3$)** δ 7.75 (d, *J* = 8.0 Hz, 2 H), 7.30 (d, *J* = 8.5 Hz, 2 H), 5.20 (m, 1 H), 3.39 (t, *J* = 6.0 Hz, 2 H), 3.27 (s, 3 H), 3.05 (dd, J_1 = 12.0 Hz, J_2 = 6.0 Hz, 2 H), 2.43 (s, 3 H), 1.73–1.68 (m, 2 H). **^{13}C NMR (125 MHz, CDCl$_3$)** δ 143.2, 137.0, 129.6, 127.0, 71.3, 58.7, 41.9, 28.8, 21.4. **HRMS** (ESI) m/z calcd. For $C_{11}H_{17}NNaO_3S$ [M+Na]$^+$: 266.0821, found: 266.0821.

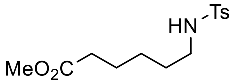

Methyl 6-((4-methylphenyl)sulfonamido)hexanoate (1ab)

Following the **Method A**, the title compound was isolated as colorless oil. **^{1}H NMR (500 MHz, CDCl$_3$)** δ 7.75 (d, *J* = 8.0 Hz, 2 H), 7.31 (d, *J* = 8.5 Hz, 2 H), 4.88 (t, *J* = 6.0 Hz, 1 H), 3.65 (s, 3 H), 2.92 (dd, J_1 = 14.0 Hz, J_2 = 7.0 Hz, 2 H), 2.43 (s, 3 H), 2.25 (t, *J* = 7.5 Hz, 2 H), 1.58–1.52 (m, 2 H), 1.50–1.44 (m, 2 H), 1.32–1.24 (m, 2 H). **^{13}C NMR (125 MHz, CDCl$_3$)** δ 173.9, 143.3, 136.9, 129.6, 127.0, 51.5, 42.8, 33.7, 29.1, 25.9, 24.2, 21.4. **HRMS** (ESI) m/z calcd. For $C_{14}H_{21}NNaO_4S$ [M+Na]$^+$: 322.1083, found: 322.1089.

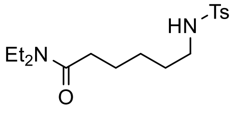

***N,N*-Diethyl-6-((4-methylphenyl)sulfonamido)hexanamide (1ac)**

Following the **Method A**, the title compound was isolated as colorless oil. **^{1}H NMR (500 MHz, CDCl$_3$)** δ 7.75 (d, *J* = 8.0 Hz, 2 H), 7.30 (d, *J* = 8.0 Hz, 2 H), 4.83 (t, *J* = 6.0 Hz, 1 H), 3.36 (q, *J* = 7.0 Hz, 2 H), 3.28 (q, *J* = 7.0 Hz, 2 H), 2.97–2.93 (m, 2 H), 2.42 (s, 3 H), 2.25 (t, *J* = 7.0 Hz, 2 H), 1.61–1.55 (m, 2 H), 1.53–1.47 (m, 2 H), 1.35–1.29 (m, 2 H), 1.16 (t, *J* = 7.0 Hz, 3 H), 1.10 (t, *J* = 7.0 Hz, 3 H). **^{13}C NMR (125 MHz, CDCl$_3$)** δ 171.8, 143.2, 137.1, 129.6, 127.1, 42.8, 41.9, 40.1, 32.6, 29.2, 26.1, 24.2, 21.5, 14.3, 13.1. **HRMS** (ESI) m/z calcd. For $C_{17}H_{28}N_2NaO_3S$ $[M+Na]^+$: 363.1713, found: 363.1721.

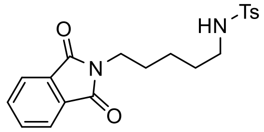

***N*-(5-(1,3-Dioxoisoindolin-2-yl)pentyl)-4-methylbenzenesulfonamide (1ad)**

Following the **Method A**, the title compound was isolated as white solid. **^{1}H NMR (500 MHz, CDCl$_3$)** δ 7.86–7.82 (m, 2 H), 7.74–7.70 (m, 4 H), 7.29 (d, *J* = 8.0 Hz, 2 H), 4.40 (t, *J* = 6.0 Hz, 1 H), 3.64 (t, *J* = 7.0 Hz, 2 H), 2.93 (dd, J_1 = 13.5 Hz, J_2 = 7.0 Hz, 2 H), 2.42 (s, 3 H), 1.66–1.60 (m, 2 H), 1.55–1.49 (m, 2 H), 1.33–1.27 (m, 2 H). **^{13}C NMR (125 MHz, CDCl$_3$)** δ 168.5, 143.3, 136.9, 134.0, 132.0, 129.7, 127.1, 123.2, 43.0, 37.5, 29.0, 28.0, 23.6, 21.5. **HRMS** (ESI) m/z calcd. For $C_{20}H_{22}N_2NaO_4S$ $[M+Na]^+$: 409.1192, found: 409.1197.

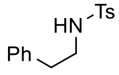

4-Methyl-*N*-phenethylbenzenesulfonamide (1ae)

Following the **Method A**, the title compound was isolated as white solid. Known compound.[2] **^{1}H NMR (500 MHz, CDCl$_3$)** δ 7.69 (d, *J* = 8.0 Hz, 2 H), 7.29–7.25 (m, 4 H), 7.23–7.20 (m, 1 H), 7.08 (d, *J* = 7.0 Hz, 2 H), 4.48 (t, *J* = 6.5 Hz, 1 H), 3.21 (dd, J_1 = 13.5 Hz, J_2 = 7.0 Hz, 2 H), 2.76 (t, *J* = 7.0 Hz, 2 H), 2.42 (s, 3 H). **^{13}C NMR (125 MHz, CDCl$_3$)** δ 143.4, 137.6, 136.8, 129.7, 128.71, 128.70, 127.1, 126.8, 44.2, 35.7, 21.5.

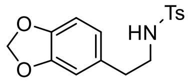

***N*-(2-(Benzo[*d*][1,3]dioxol-5-yl)ethyl)-4-methylbenzenesulfonamide (1af)**

Following the **Method A**, the title compound was isolated as white solid. Known compound.[15] **^{1}H NMR (500 MHz, CDCl$_3$)** δ 7.69 (d, *J* = 8.5 Hz, 2 H), 7.29 (d, *J* = 8.5 Hz, 2 H), 6.69 (d, *J* = 8.0 Hz, 1 H), 6.53–6.52 (m, 2 H), 5.92 (s, 2 H), 4.48 (t, *J* = 6.0 Hz, 1 H), 3.15 (dd, J_1 = 13.0 Hz, J_2 = 6.5 Hz, 2 H), 2.66 (t, *J* = 7.0 Hz, 2 H), 2.43 (s, 3 H). **^{13}C NMR (125 MHz, CDCl$_3$)** δ 147.8, 146.4, 143.4, 136.8, 131.3, 129.7, 127.1, 121.7, 108.9, 108.4, 100.9, 44.3, 35.4, 21.5.

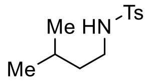

***N*-Isopentyl-4-methylbenzenesulfonamide (1ag)**

Following the **Method A**, the title compound was isolated as colorless oil. Known compound.[5] **^{1}H NMR (500 MHz, CDCl$_3$)** δ 7.76 (d, J = 8.0 Hz, 2 H), 7.31 (d, J = 8.5 Hz, 2 H), 4.61 (m, 1 H), 2.94 (dd, J_1 = 14.0 Hz, J_2 = 7.0 Hz, 2 H), 2.43 (s, 3 H), 1.62–1.53 (m, 1 H), 1.36–1.31 (m, 2 H), 0.82 (d, J = 6.5 Hz, 6 H). **^{13}C NMR (125 MHz, CDCl$_3$)** δ 143.3, 136.9, 129.6, 127.1, 41.4, 38.3, 25.4, 22.2, 21.5.

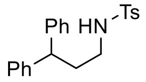

***N*-(3,3-Diphenylpropyl)-4-methylbenzenesulfonamide (1ah)**

Following the **Method A**, the title compound was isolated as white solid. Known compound.[16] **^{1}H NMR (500 MHz, CDCl$_3$)** δ 7.67 (d, J = 8.5 Hz, 2 H), 7.26–7.22 (m, 6 H), 7.17–7.12 (m, 6 H), 4.58 (t, J = 6.0 Hz, 1 H), 3.93 (t, J = 8.0 Hz, 1 H), 2.90 (dd, J_1 = 13.5 Hz, J_2 = 6.5 Hz, 2 H), 2.41 (s, 3 H), 2.23–2.18 (m, 2 H). **^{13}C NMR (125 MHz, CDCl$_3$)** δ 143.6, 143.3, 136.8, 129.7, 128.6, 127.7, 127.1, 126.4, 48.1, 41.6, 35.4, 21.5.

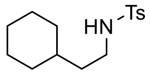

***N*-(2-Cyclohexylethyl)-4-methylbenzenesulfonamide (1ai)**

Following the **Method A**, the title compound was isolated as white solid. Known compound.[17] **^{1}H NMR (500 MHz, CDCl$_3$)** δ 7.76 (d, J = 8.5 Hz, 2 H), 7.31 (d, J = 8.0 Hz, 2 H), 4.59 (t, J = 6.0 Hz, 1 H), 2.94 (dd, J_1 = 13.5 Hz, J_2 = 6.5 Hz, 2 H), 2.43 (s, 3 H), 1.67–1.56 (m, 5 H), 1.35–1.31 (m, 2 H), 1.27–1.04 (m, 4 H), 0.85–0.77 (m, 2 H). **^{13}C NMR (125 MHz, CDCl$_3$)** δ 143.3, 136.9, 129.6, 127.1, 40.9, 36.9, 34.7, 32.9, 26.3, 26.0, 21.5.

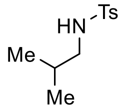

***N*-Isobutyl-4-methylbenzenesulfonamide (1aj)**

Following the **Method A**, the title compound was isolated as white solid. Known compound.[18] **^{1}H NMR (500 MHz, CDCl$_3$)** δ 7.75 (d, J = 8.5 Hz, 2 H), 7.31 (d, J = 8.5 Hz, 2 H), 4.70 (t, J = 6.5 Hz, 1 H), 2.74 (t, J = 6.5 Hz, 2 H), 2.43 (s, 3 H), 1.75–1.67 (m, 1 H), 0.87 (d, J = 6.5 Hz, 6 H). **^{13}C NMR (125 MHz, CDCl$_3$)** δ 143.2, 137.0, 129.6, 127.0, 50.5, 28.4, 21.5, 19.8.

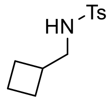

***N*-(Cyclobutylmethyl)-4-methylbenzenesulfonamide (1ak)**

Following the **Method A**, the title compound was isolated as white solid. **^{1}H NMR (500 MHz, CDCl$_3$)** δ 7.76 (d, J = 8.0 Hz, 2 H), 7.31 (d, J = 8.0 Hz, 2 H), 4.83 (t, J = 6.0 Hz, 1 H), 2.93 (t, J = 6.5 Hz, 2 H), 2.43–2.36 (m, 4 H),

2.01–1.94 (m, 2 H), 1.89–1.74 (m, 2 H), 1.63–1.56 (m, 2 H). **^{13}C NMR (125 MHz, CDCl$_3$)** δ 143.2, 136.9, 129.6, 127.0, 48.3, 34.6, 25.4, 21.4, 18.0. **HRMS** (ESI) m/z calcd. For $C_{12}H_{17}NNaO_2S$ $[M+Na]^+$: 252.0872, found: 252.0883.

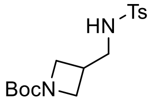

***tert*-Butyl 3-(((4-methylphenyl)sulfonamido)methyl)azetidine-1-carboxylate (1al)**

Following the **Method A**, the title compound was isolated as white solid. **^{1}H NMR (500 MHz, CDCl$_3$)** δ 7.75 (d, *J* = 8.0 Hz, 2 H), 7.32 (d, *J* = 8.0 Hz, 2 H), 5.23 (t, *J* = 6.0 Hz, 1 H), 3.92 (t, *J* = 8.5 Hz, 2 H), 3.55 (m, 2 H), 3.11 (t, *J* = 6.5 Hz, 2 H), 2.65–2.62 (m, 1 H), 2.43 (s, 3 H), 1.41 (s, 9 H). **^{13}C NMR (125 MHz, CDCl$_3$)** δ 156.2, 143.6, 136.8, 129.8, 127.0, 79.6, 51.5, 46.1, 28.4, 28.3, 21.5. **HRMS** (ESI) m/z calcd. For $C_{16}H_{24}N_2NaO_4S$ $[M+Na]^+$: 363.1349, found: 363.1356.

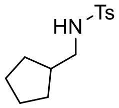

***N*-(Cyclopentylmethyl)-4-methylbenzenesulfonamide (1am)**

Following the **Method B**, the title compound was isolated as white solid. Known compound.[19] **^{1}H NMR (500 MHz, CDCl$_3$)** δ 7.74 (d, *J* = 8.0 Hz, 2 H), 7.31 (d, *J* = 8.0 Hz, 2 H), 4.36 (t, *J* = 6.0 Hz, 1 H), 2.86 (t, *J* = 6.5 Hz, 2 H), 2.43 (s, 3 H), 2.01–1.92 (m, 1 H), 1.73–1.67 (m, 2 H), 1.58–1.48 (m, 4 H), 1.14–1.07 (m, 2 H). **^{13}C NMR (125 MHz, CDCl$_3$)** δ 143.3, 137.0, 129.7, 127.1, 48.2, 39.5, 30.1, 25.1, 21.5.

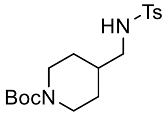

***tert*-Butyl 4-(((4-methylphenyl)sulfonamido)methyl)piperidine-1-carboxylate (1an)**

Following the **Method A**, the title compound was isolated as white solid. **H NMR (500 MHz, CDCl$_3$)** δ 7.74 (d, *J* = 8.0 Hz, 2 H), 7.31 (d, *J* = 8.0 Hz, 2 H), 4.85 (t, *J* = 6.0 Hz, 1 H), 4.14–4.06 (m, 2 H), 2.80 (t, *J* = 6.5 Hz, 2 H), 2.62–2.56 (m, 2 H), 2.43 (s, 3 H), 1.66–1.56 (m, 3 H), 1.44 (s, 9 H), 1.08–0.99 (m, 2 H). **^{13}C NMR (125 MHz, CDCl$_3$)** δ 154.7, 143.4, 136.9, 129.7, 127.0, 79.4, 48.5, 43.1, 36.4, 29.5, 28.4, 21.5. **HRMS** (ESI) m/z calcd. For $C_{18}H_{28}N_2NaO_4S$ $[M+Na]^+$: 391.1662, found: 391.1666.

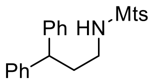

***N*-(3,3-Diphenylpropyl)-2,4,6-trimethylbenzenesulfonamide (4a)**

Following the **Method A**, but MtsCl (1.1 equiv.) was used. The title compound was isolated as white solid. **^{1}H NMR (500 MHz, $CDCl_3$)** δ 7.22 (t, J = 7.5 Hz, 4 H), 7.15 (t, J = 7.5 Hz, 2 H), 7.08 (d, J = 7.5 Hz, 4 H), 6.93 (s, 2 H), 4.55 (t, J = 6.5 Hz, 1 H), 3.87 (t, J = 6.5 Hz, 1 H), 2.87 (dd, J_1 = 13.5 Hz, J_2 = 6.5 Hz, 2 H), 2.55 (s, 6 H), 2.29 (s, 3 H), 2.16 (dd, J_1 = 14.5 Hz, J_2 = 7.5 Hz, 2 H). **^{13}C NMR (125 MHz, $CDCl_3$)** δ 143.6, 142.1, 139.0, 133.7, 131.9, 128.5, 127.6, 126.4, 48.2, 41.1, 35.4, 22.9, 20.9. **HRMS** (ESI) m/z calcd. For $C_{24}H_{27}NNaO_2S$ $[M+Na]^+$: 416.1655, found: 416.1658.

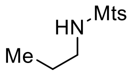

2,4,6-Trimethyl-*N*-propylbenzenesulfonamide (4b)

Following the **Method A**, but MtsCl (1.1 equiv.) was used. The title compound was isolated as white solid. Known compound.[20] **^{1}H NMR (500 MHz, $CDCl_3$)** δ 6.96 (s, 2 H), 4.57 (s, 1 H), 2.86 (dd, J_1 = 13.5 Hz, J_2 = 6.5 Hz, 2 H), 2.64 (s, 6 H), 2.30 (s, 3 H), 1.51–1.42 (m, 2 H), 0.86 (t, J = 7.5 Hz, 3 H). **^{13}C NMR (125 MHz, $CDCl_3$)** δ 142.0, 139.0, 133.6, 131.9, 44.3, 22.90, 22.85, 20.9, 11.1.

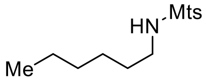

***N*-Hexyl-2,4,6-trimethylbenzenesulfonamide (4c)**

Following the **Method A**, but MtsCl (1.1 equiv.) was used. The title compound was isolated as white solid. **^{1}H NMR (500 MHz, $CDCl_3$)** δ 6.96 (s, 2 H), 4.45 (t, J = 6.0 Hz, 1 H), 2.88 (dd, J_1 = 13.5 Hz, J_2 = 7.0 Hz, 2 H), 2.64 (s, 6 H), 2.30 (s, 3 H), 1.46–1.40 (m, 2 H), 1.27–1.15 (m, 6 H), 0.84 (t, J = 7.5 Hz, 3 H). **^{13}C NMR (125 MHz, $CDCl_3$)** δ 142.1, 139.0, 133.6, 131.9, 42.6, 31.2, 29.5, 26.2, 22.9, 22.4, 20.9, 13.9. **HRMS** (ESI) m/z calcd. For $C_{15}H_{25}NNaO_2S$ $[M+Na]^+$: 306.1498, found: 306.1503.

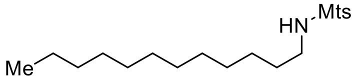

***N*-Dodecyl-2,4,6-trimethylbenzenesulfonamide (4d)**

Following the **Method A**, but MtsCl (1.1 equiv.) was used. The title compound was isolated as white solid. Known compound.[20] **^{1}H NMR (500 MHz, $CDCl_3$)** δ 6.96 (s, 2 H), 4.43 (t, J = 6.0 Hz, 1 H), 2.88 (dd, J_1 = 13.5 Hz, J_2 = 7.0 Hz, 2 H), 2.64 (s, 6 H), 2.30 (s, 3 H), 1.46–1.40 (m, 2 H), 1.31–1.19 (m, 18 H), 0.88 (t, J = 7.0 Hz, 3 H). **^{13}C NMR (125 MHz, $CDCl_3$)** δ 142.0, 139.0, 133.7, 131.9, 42.6, 31.9, 29.6, 29.5, 29.4, 29.3, 29.0, 26.5, 22.9, 22.7, 20.9, 14.1.

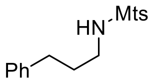

2,4,6-Trimethyl-*N*-(3-phenylpropyl)benzenesulfonamide (4e)

Following the **Method A**, but MtsCl (1.1 equiv.) was used. The title compound was isolated as white solid. **^{1}H NMR (500 MHz, CDCl$_3$)** δ 7.23 (t, *J* = 7.5 Hz, 2 H), 7.16 (t, *J* = 7.5 Hz, 1 H), 7.03 (d, *J* = 7.0 Hz, 2 H), 6.95 (s, 2 H), 4.57 (t, *J* = 6.0 Hz, 1 H), 2.92 (dd, J_1 = 13.5 Hz, J_2 = 7.0 Hz, 2 H), 2.62 (s, 6 H), 2.56 (t, *J* = 7.5 Hz, 2 H), 2.30 (s, 3 H), 1.78–1.72 (m, 2 H). **^{13}C NMR (125 MHz, CDCl$_3$)** δ 142.1, 140.8, 139.0, 133.7, 131.9, 128.4, 128.3, 126.0, 42.0, 32.7, 31.1, 22.9, 20.9. **HRMS** (ESI) m/z calcd. For $C_{18}H_{23}NNaO_2S$ $[M+Na]^+$: 340.1342, found: 340.1342.

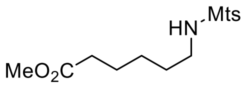

Methyl 6-((2,4,6-trimethylphenyl)sulfonamido)hexanoate (4f)

Following the **Method A**, but MtsCl (1.1 equiv.) was used. The title compound was isolated as white solid. **^{1}H NMR (500 MHz, CDCl$_3$)** δ 6.96 (s, 2 H), 4.54 (t, *J* = 6.0 Hz, 1 H), 3.66 (s, 3 H), 2.90 (dd, J_1 = 13.5 Hz, J_2 = 6.5 Hz, 2 H), 2.63 (s, 6 H), 2.30 (s, 3 H), 2.25 (t, *J* = 7.5 Hz, 2 H), 1.57–1.51 (m, 2 H), 1.49–1.43 (m, 2 H), 1.31–1.25 (m, 2 H). **^{13}C NMR (125 MHz, CDCl$_3$)** δ 173.9, 142.1, 139.0, 133.6, 131.9, 51.5, 42.3, 33.7, 29.2, 26.0, 24.2, 22.9, 20.9. **HRMS** (ESI) m/z calcd. For $C_{16}H_{25}NNaO_4S$ $[M+Na]^+$: 350.1397, found: 350.1405.

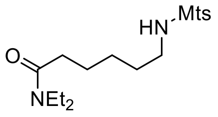

***N*,*N*-Diethyl-6-((2,4,6-trimethylphenyl)sulfonamido)hexanamide (4g)**

Following the **Method A**, but MtsCl (1.1 equiv.) was used. The title compound was isolated as white solid. **^{1}H NMR (500 MHz, CDCl$_3$)** δ 6.95 (s, 2 H), 4.74 (t, *J* = 6.0 Hz, 1 H), 3.36 (q, *J* = 7.0 Hz, 2 H), 3.28 (q, *J* = 7.5 Hz, 2 H), 2.91 (dd, J_1 = 13.5 Hz, J_2 = 6.5 Hz, 2 H), 2.64 (s, 6 H), 2.30 (s, 3 H), 2.25 (t, *J* = 7.0 Hz, 2 H), 1.61–1.55 (m, 2 H), 1.51–1.46 (m, 2 H), 1.34–1.28 (m, 2 H), 1.16 (t, *J* = 7.0 Hz, 3 H), 1.10 (t, *J* = 7.0 Hz, 3 H). **^{13}C NMR (125 MHz, CDCl$_3$)** δ 171.7, 142.0, 139.0, 133.8, 131.9, 42.2, 41.9, 40.1, 32.6, 29.3, 26.2, 24.4, 22.9, 20.9, 14.3, 13.1. **HRMS** (ESI) m/z calcd. For $C_{19}H_{32}N_2NaO_3S$ $[M+Na]^+$: 391.2026, found: 391.2026.

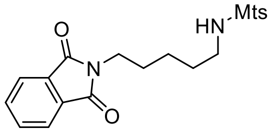

***N*-(5-(1,3-Dioxoisoindolin-2-yl)pentyl)-2,4,6-trimethylbenzenesulfonamide (4h)**

Following the **Method A**, but MtsCl (1.1 equiv.) was used. The title compound was isolated as white solid. **^{1}H NMR (500 MHz, CDCl$_3$)** δ 7.85–7.82 (m, 2 H), 7.73–7.71 (m, 2 H), 6.94 (s, 2 H), 4.44 (t, *J* = 6.0 Hz, 1 H), 3.63 (t, *J* = 7.0 Hz, 2 H), 2.89 (dd, J_1 = 13.5 Hz, J_2 = 6.5 Hz, 2 H), 2.62 (s, 6 H), 2.29 (s, 3 H), 1.63–1.59 (m, 2 H), 1.53–1.47 (m, 2 H), 1.33–1.26 (m, 2 H). **^{13}C NMR (125 MHz, CDCl$_3$)** δ 168.4, 142.1, 139.0, 134.0, 133.6, 132.1, 131.9, 123.2, 42.4, 37.4, 28.9, 28.0, 23.7, 22.9, 20.9. **HRMS** (ESI) m/z calcd. For $C_{22}H_{26}N_2NaO_4S$ $[M+Na]^+$: 437.1505, found: 437.1512.

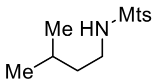

***N*-Isopentyl-2,4,6-trimethylbenzenesulfonamide (4i)**

Following the **Method A**, but MtsCl (1.1 equiv.) was used. The title compound was isolated as white solid. **^{1}H NMR (500 MHz, CDCl$_3$)** δ 6.96 (s, 2 H), 4.59 (t, *J* = 6.0 Hz, 1 H), 2.92–2.88 (m, 2 H), 2.64 (s, 6 H), 2.30 (s, 3 H), 1.61–1.50 (m, 1 H), 1.33 (dd, J_1 = 14.5 Hz, J_2 = 7.0 Hz, 2 H), 0.81 (d, *J* = 6.5 Hz, 6 H). **^{13}C NMR (125 MHz, CDCl$_3$)** δ 142.0, 139.0, 133.6, 131.9, 40.7, 38.3, 25.4, 22.9, 22.1, 20.8. **HRMS** (ESI) m/z calcd. For $C_{14}H_{23}NNaO_2S$ $[M+Na]^+$: 292.1342, found: 292.1346.

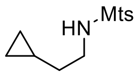

***N*-(2-Cyclopropylethyl)-2,4,6-trimethylbenzenesulfonamide (4j)**

Following the **Method A**, but MtsCl (1.1 equiv.) was used. The title compound was isolated as white solid. **^{1}H NMR (500 MHz, CDCl$_3$)** δ 6.96 (s, 2 H), 4.60 (t, *J* = 6.0 Hz, 1 H), 2.97 (dd, J_1 = 13.5 Hz, J_2 = 7.0 Hz, 2 H), 2.65 (s, 6 H), 2.30 (s, 3 H), 1.37 (dd, J_1 = 14.0 Hz, J_2 = 7.0 Hz, 2 H), 0.61–0.53 (m, 1 H), 0.45–0.38 (m, 2 H), 0.03–0.01 (m, 2 H). **^{13}C NMR (125 MHz, CDCl$_3$)** δ 142.1, 139.1, 133.5, 131.9, 42.9, 34.3, 22.9, 20.9, 8.2, 4.1. **HRMS** (ESI) m/z calcd. For $C_{14}H_{21}NNaO_2S$ $[M+Na]^+$: 290.1185, found: 290.1190.

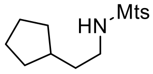

***N*-(2-Cyclopentylethyl)-2,4,6-trimethylbenzenesulfonamide (4k)**

Following the **Method A**, but MtsCl (1.1 equiv.) was used. The title compound was isolated as white solid. **^{1}H NMR (500 MHz, CDCl$_3$)** δ 6.96 (s, 2 H), 4.41 (t, *J* = 6.0 Hz, 1 H), 2.89 (dd, J_1 = 14.0 Hz, J_2 = 6.5 Hz, 2 H), 2.64 (s, 6 H), 2.30 (s, 3 H), 1.75–1.63 (m, 3 H), 1.57–1.52 (m, 2 H), 1.50–1.43 (m, 4 H), 1.03–0.96 (m, 2 H). **^{13}C NMR (125 MHz, CDCl$_3$)** δ 142.1, 139.1, 133.6, 131.9, 42.0, 37.3, 35.8, 32.4, 25.0, 22.9, 20.9. **HRMS** (ESI) m/z calcd. For $C_{16}H_{25}NNaO_2S$ $[M+Na]^+$: 318.1498, found: 318.1504.

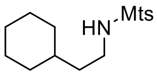

***N*-(2-Cyclohexylethyl)-2,4,6-trimethylbenzenesulfonamide (4l)**

Following the **Method A**, but MtsCl (1.1 equiv.) was used. The title compound was isolated as white solid. **^{1}H NMR (500 MHz, CDCl$_3$)** δ 6.96 (s, 2 H), 4.34 (t, *J* = 6.0 Hz, 1 H), 2.91 (dd, J_1 = 13.5 Hz, J_2 = 7.0 Hz, 2 H), 2.64 (s, 6 H), 2.30 (s, 3 H), 1.65–1.59 (m, 3 H), 1.55–1.53 (m, 2 H), 1.32 (dd, J_1 = 14.5 Hz, J_2 = 7.0 Hz, 2 H), 1,24–1.07 (m, 4 H), 0.84–0.76 (m, 2 H). **^{13}C NMR (125 MHz, CDCl$_3$)** δ 142.1, 139.1, 133.6, 131.9, 40.2, 36.9, 34.8, 32.9, 26.4, 26.1, 22.9, 20.9. **HRMS** (ESI) m/z calcd. For $C_{17}H_{27}NNaO_2S$ $[M+Na]^+$: 332.1655, found: 332.1662.

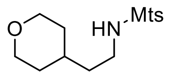

2,4,6-Trimethyl-*N*-(2-(tetrahydro-2*H*-pyran-4-yl)ethyl)benzenesulfonamide (4m)

Following the **Method A**, but MtsCl (1.1 equiv.) was used. The title compound was isolated as white solid. **^{1}H NMR (500 MHz, CDCl$_3$)** δ 6.96 (s, 2 H), 4.43 (t, J = 6.0 Hz, 1 H), 3.91–3.87 (m, 2 H), 3.31–3.26 (m, 2 H), 2.94 (dd, J_1 = 13.5 Hz, J_2 = 7.0 Hz, 2 H), 2.64 (s, 6 H), 2.30 (s, 3 H), 1.54–1.47 (m, 1 H), 1.46–1.37 (m, 4 H), 1.23–1.15 (m, 2 H). **^{13}C NMR (125 MHz, CDCl$_3$)** δ 142.2, 139.1, 133.5, 132.0, 67.8, 39.6, 36.5, 32.6, 32.1, 23.0, 20.9. **HRMS** (ESI) m/z calcd. For $C_{16}H_{25}NNaO_3S$ [M+Na]$^+$: 334.1447, found: 334.1456.

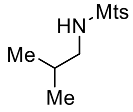

***N*-Isobutyl-2,4,6-trimethylbenzenesulfonamide (4n)**

Following the **Method A**, but MtsCl (1.1 equiv.) was used. The title compound was isolated as white solid. Known compound.[20] **^{1}H NMR (500 MHz, CDCl$_3$)** δ 6.96 (s, 2 H), 4.78 (t, J = 6.5 Hz, 1 H), 2.69 (t, J = 6.5 Hz, 2 H), 2.65 (s, 6 H), 2.30 (s, 3 H), 1.76–1.65 (m, 1 H), 0.85 (d, J = 6.5 Hz, 6 H). **^{13}C NMR (125 MHz, CDCl$_3$)** δ 142.0, 138.9, 133.6, 131.8, 49.9, 28.3, 22.8, 20.8, 19.9.

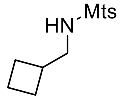

***N*-(Cyclobutylmethyl)-2,4,6-trimethylbenzenesulfonamide (4o)**

Following the **Method A**, but MtsCl (1.1 equiv.) was used. The title compound was isolated as white solid. Known compound.[20] **^{1}H NMR (500 MHz, CDCl$_3$)** δ 6.96 (s, 2 H), 4.38 (t, J = 6.0 Hz, 1 H), 2.91–2.88 (m, 2 H), 2.64 (s, 6 H), 2.43–2.33 (m, 1 H), 2.31 (s, 3 H), 2.01–1.95 (m, 2 H), 1.91–1.75 (m, 2 H), 1.61–1.53 (m, 2 H). **^{13}C NMR (125 MHz, CDCl$_3$)** δ 142.1, 139.0, 133.6, 131.9, 47.9, 34.7, 25.5, 22.9, 20.9, 18.0.

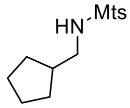

***N*-(Cyclopentylmethyl)-2,4,6-trimethylbenzenesulfonamide (4p)**

Following the **Method A**, but MtsCl (1.1 equiv.) was used. The title compound was isolated as white solid. Known compound.[20] **^{1}H NMR (500 MHz, CDCl$_3$)** δ 6.96 (s, 2 H), 4.51 (t, J = 6.0 Hz, 1 H), 2.80 (t, J = 7.0 Hz, 2 H), 2.64 (s, 6 H), 2.30 (s, 3 H), 2.00–1.91 (m, 1 H), 1.73–1.67 (m, 2 H), 1.60–1.46 (m, 4 H), 1.13–1.05 (m, 2 H). **^{13}C NMR (125 MHz, CDCl$_3$)** δ 142.0, 139.0, 133.5, 131.9, 47.6, 39.5, 30.2, 25.1, 22.9, 20.9.

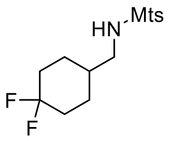

***N*-((4,4-Difluorocyclohexyl)methyl)-2,4,6-trimethylbenzenesulfonamide (4q)**

Following the **Method A**, but MtsCl (1.1 equiv.) was used. The title compound was isolated as white solid. Known compound.[20] **^{1}H NMR (500 MHz, $CDCl_3$)** δ 6.97 (s, 2 H), 4.68 (t, *J* = 6.5 Hz, 1 H), 2.77 (t, *J* = 7.0 Hz, 2 H), 2.63 (s, 6 H), 2.31 (s, 3 H), 2.08–2.02 (m, 2 H), 1.77–1.75 (m, 2 H), 1.71–1.57 (m, 2 H), 1.55–1.51 (m, 1 H), 1.24–1.16 (m, 2 H). **^{13}C NMR (125 MHz, $CDCl_3$)** δ 142.3, 139.0, 133.5, 132.0, 123.2 (dd, J_1 = 240.8 Hz, J_2 = 238.0 Hz), 47.4 (d, *J* = 2.9 Hz), 36.1 (d, *J* = 1.3 Hz), 32.9 (dd, J_1 = 25.4 Hz, J_2 = 23.0 Hz), 26.5 (d, *J* = 9.6 Hz), 22.9, 20.9. **^{19}F NMR (470 MHz, $CDCl_3$)** δ -92.1 (d, *J* = 236.4 Hz), -102.1 (d, *J* = 235.0 Hz).

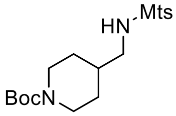

***tert*-Butyl 4-(((2,4,6-trimethylphenyl)sulfonamido)methyl)piperidine-1-carboxylate (4r)**

Following the **Method A**, but MtsCl (1.1 equiv.) was used. The title compound was isolated as white solid. Known compound.[20] **^{1}H NMR (500 MHz, $CDCl_3$)** δ 6.96 (s, 2 H), 4.59 (t, *J* = 6.5 Hz, 1 H), 4.06 (m, 2 H), 2.76 (t, *J* = 7.0 Hz, 2 H), 2.63–2.55 (m, 8 H), 2.31 (s, 3 H), 1.61–1.54 (m, 3 H), 1.44 (s, 9 H), 1.06–0.98 (m, 2 H). **^{13}C NMR (125 MHz, $CDCl_3$)** δ 154.7, 142.3, 139.0, 133.5, 132.0, 79.4, 48.0, 43.7, 36.4, 29.6, 28.4, 22.9, 20.9.

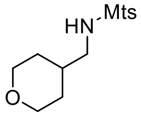

2,4,6-Trimethyl-*N*-((tetrahydro-2*H*-pyran-4-yl)methyl)benzenesulfonamide (4s)

Following the **Method A**, but MtsCl (1.1 equiv.) was used. The title compound was isolated as white solid. Known compound.[20] **^{1}H NMR (500 MHz, $CDCl_3$)** δ 6.96 (s, 2 H), 4.76 (t, *J* = 6.0 Hz, 1 H), 3.92 (dd, J_1 = 11.0 Hz, J_2 = 3.5 Hz, 2 H), 3.33–3.28 (m, 2 H), 2.76 (t, *J* = 6.5 Hz, 2 H), 2.64 (s, 6 H), 2.31 (s, 3 H), 1.71–1.63 (m, 1 H), 1.58–1.56 (m, 2 H), 1.24–1.15 (m, 2 H). **^{13}C NMR (125 MHz, $CDCl_3$)** δ 142.2, 138.9, 133.5, 131.9, 67.4, 48.2, 35.2, 30.4, 22.9, 20.9.

2.2.2 Synthesis of Olefins

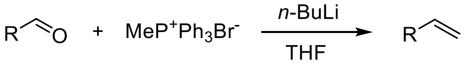

Method C: To a suspension of methyltriphenylphosphonium bromide (10.5 mmol, 3.8 g, 1.05 equiv.) in THF (30 mL) was added *n*-BuLi (11.0 mmol, 4.4 mL, 2.5 M in *n*-hexane, 1.1 equiv.) at 0 ºC under argon. After stirring for 15

min, aryl aldehyde (10.0 mmol, 1.0 equiv.) was added. The reaction mixture was warmed to room temperature and the progress of the reaction was monitored by TLC. After the reaction was completed, the reaction mixture was quenched with sat. NH_4Cl aqueous (20 mL) and extracted with EtOAc (40 mL). The organic layers were dried over Na_2SO_4 and concentrated under reduced pressure. Purification by flash chromatography provided desired product (90-99% yield).

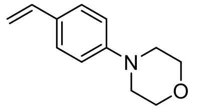

4-(4-Vinylphenyl)morpholine (2g)

Following the **Method C**, the title compound was isolated as white solid. Known compound.[21] **^{1}H NMR (500 MHz, $CDCl_3$)** δ 7.33 (d, *J* = 9.0 Hz, 2 H), 6.86 (d, *J* = 9.0 Hz, 2 H), 6.64 (dd, J_1 = 18.0 Hz, J_2 = 11.0 Hz, 1 H), 5.60 (dd, J_1 = 17.5 Hz, J_2 = 0.5 Hz, 1 H), 5.10 (dd, J_1 = 11.0 Hz, J_2 = 0.5 Hz, 1 H), 3.85 (t, *J* = 5.0 Hz, 4 H), 3.16 (t, *J* = 5.0 Hz, 4 H). **^{13}C NMR (125 MHz, $CDCl_3$)** δ 150.8, 136.2, 129.5, 127.1, 115.4, 111.1, 66.8, 49.1.

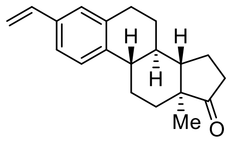

(8*R*,9*S*,13*S*,14*S*)-13-Methyl-3-vinyl-6,7,8,9,11,12,13,14,15,16-decahydro-17*H*-cyclopenta[*a*]phenanthren-17-one (2n)

The title compound was synthesized according to the reported literature[22]. Step 1: To a solution of estrone (3.7 mmol, 1.0 g, 1.0 equiv.) and Et_3N (7.4 mmol, 1.0 mL, 2.0 equiv.) in DCM (15 mL), Tf_2O (5.2 mmol, 0.9 mL, 1.4 equiv.) was slowly added at 0 ºC. The mixture was allowed to warm to room temperature and stirred at room temperature under argon for 3 h. The resulting brown mixture was diluted with DCM (15 mL), washed with sat. NH_4Cl aqueous (20 mL), and the aqueous layer was extracted with DCM (20 mL). The combined organic layers were dried over $MgSO_4$ and concentrated under reduced pressure. Purification by flash chromatography provided the corresponding trifluoromethanesulfonate (1.21 g, 81% yield).

Step 2: A 25 mL round-bottom flask equipped with a condenser was charged with the above-mentioned trifluoromethanesulfonate (2.0 mmol, 0.8 g, 1.0 equiv.), potassium vinyltrifluoroborate (2.0 mmol, 0.27 g, 1.0 equiv.), $PdCl_2$ (0.02 mmol, 3.6 mg, 2 mol%), PPh_3 (0.06 mmol, 15.7 mg, 6 mol%) and Cs_2CO_3 (6.0 mmol, 2.0 g, 3.0 equiv.) in an nitrogen-filled glovebox. Then THF (3.6 mL) were added and the flask was sealed and removed from the glovebox. H_2O (0.4 mL) was added, and the mixture was stirred at 85 ºC for 24 h. The resulting dark brown mixture was allowed to cool to room temperature, diluted with DCM (30 mL), and washed with H_2O (30 mL). The aqueous layer was extracted with DCM (20 mL). The combined organic layers were dried over $MgSO_4$ and concentrated under reduced pressure. Purification by flash chromatography provided the desired product (0.34 g, 61% yield).

White solid. Known compound.[21] **^{1}H NMR (500 MHz, $CDCl_3$)** δ 7.27–7.20 (m, 2 H), 7.14 (s, 1 H), 6.67 (dd, J_1 = 17.5 Hz, J_2 = 10.5 Hz, 1 H), 5.70 (d, J = 17.5 Hz, 1 H), 5.19 (d, J = 11.0 Hz, 1 H), 2.93–2.91 (m, 2 H), 2.53–2.38 (m, 2 H), 2.32–2.29 (m, 1 H), 2.18–1.96 (m, 4 H), 1.67–1.41 (m, 6 H), 0.91 (s, 3 H). **^{13}C NMR (125 MHz, $CDCl_3$)** δ 220.9, 139.5, 136.5, 135.2, 126.8, 125.5, 123.6, 113.2, 50.5, 48.0, 44.4, 38.1, 35.8, 31.6, 29.4, 26.5, 25.7, 21.6, 13.8.

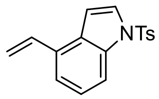

1-Tosyl-4-vinyl-1*H*-indole (2o)

Following the **Method C**, the title compound was isolated as white solid. Known compound.[23] **^{1}H NMR (500 MHz, $CDCl_3$)** δ 7.90 (d, J = 8.0 Hz, 1 H), 7.75 (d, J = 8.5 Hz, 2 H), 7.59 (d, J = 4.0 Hz, 1 H), 7.36 (d, J = 7.5 Hz, 1 H), 7.29–7.25 (m, 1 H), 7.20 (d, J = 8.5 Hz, 2 H), 6.98 (dd, J_1 = 17.5 Hz, J_2 = 11.0 Hz, 1 H), 6.83 (d, J = 3.5 Hz, 1 H), 5.81 (dd, J_1 = 17.5 Hz, J_2 = 0.5 Hz, 1 H), 5.38–5.36 (m, 1 H), 2.31 (s, 3 H). **^{13}C NMR (125 MHz, $CDCl_3$)** δ 145.0, 135.2, .135.1, 133.6, 130.8, 129.8, 128.9, 126.8, 126.4, 124.6, 119.9, 115.9, 112.8, 107.1, 21.5.

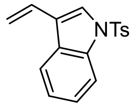

1-Tosyl-3-vinyl-1*H*-indole (2p)

Following the **Method C**, the title compound was isolated as white solid. Known compound.[24] **^{1}H NMR (500 MHz, $CDCl_3$)** δ 7.99 (d, J = 8.0 Hz, 1 H), 7.77–7.73 (m, 3 H), 7.60 (s, 1 H), 7.34–7.31 (m, 1 H), 7.28–7.25 (m, 1 H), 7.20 (d, J = 8.5 Hz, 2 H), 6.76 (dd, J_1 = 17.5 Hz, J_2 = 11.5 Hz, 1 H), 5.79 (d, J = 8.0 Hz, 1 H), 5.34 (dd, J_1 = 11.5 Hz, J_2 = 0.5 Hz, 1 H), 2.32 (s, 3 H). **^{13}C NMR (125 MHz, $CDCl_3$)** δ 145.0, 135.5, 135.1, 129.9, 129.0, 127.5, 126.8, 124.9, 124.0, 123.5, 120.9, 120.4, 115.3, 113.7, 21.5.

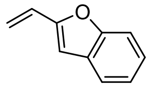

2-Vinylbenzofuran (2q)

Following the **Method C**, the title compound was isolated as colorless oil. Known compound.[25] **^{1}H NMR (500 MHz, $CDCl_3$)** δ 7.51 (d, J = 8.0 Hz, 1 H), 7.44 (d, J = 8.0 Hz, 1 H), 7.28–7.23 (m, 1 H), 7.18 (t, J = 7.5 Hz, 1 H), 6.66–6.58 (m, 2 H), 5.95 (d, J = 17.5 Hz, 1 H), 5.37 (d, J = 11.5 Hz, 1 H). **^{13}C NMR (125 MHz, $CDCl_3$)** δ 154.8, 154.7, 128.8, 125.3, 124.6, 122.8, 121.0, 115.7, 111.0, 104.7.

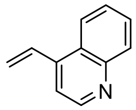

4-Vinylquinoline (2r)

Following the **Method C**, the title compound was isolated as light yellow oil. Known compound.[26] **^{1}H NMR (500 MHz, CDCl$_3$)** δ 8.87 (d, *J* = 4.5 Hz, 1 H), 8.13–8.08 (m, 2 H), 7.73–7.69 (m, 1 H), 7.57–7.54 (m, 1 H), 7.46–7.39 (m, 2 H), 5.97 (dd, J_1 = 17.0 Hz, J_2 = 1.0 Hz, 1 H), 5.66 (dd, J_1 = 11.0 Hz, J_2 = 1.0 Hz, 1 H). **^{13}C NMR (125 MHz, CDCl$_3$)** δ 150.2, 148.5, 143.4, 132.0, 130.0, 129.2, 126.5, 126.1, 123.5, 120.6, 117.4.

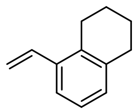

5-Vinyl-1,2,3,4-tetrahydronaphthalene (2x)

Following the same synthetic procedure of compound **2n**, the title compound was isolated as colorless oil. Known compound.[27] **^{1}H NMR (500 MHz, CDCl$_3$)** δ 7.28 (d, *J* = 7.5 Hz, 1 H), 7.08 (t, *J* = 7.5 Hz, 1 H), 7.00 (d, *J* = 7.5 Hz, 1 H), 6.93 (dd, J_1 = 17.5 Hz, J_2 = 11.0 Hz, 1 H), 5.58 (dd, J_1 = 17.5 Hz, J_2 = 1.5 Hz, 1 H), 5.26 (dd, J_1 = 11.0 Hz, J_2 = 1.5 Hz, 1 H), 2.79–2.73 (m, 4 H), 1.85–1.73 (m, 4 H). **^{13}C NMR (125 MHz, CDCl$_3$)** δ 137.3, 137.2, 135.0, 134.3, 128.8, 125.4, 123.1, 115.3, 30.1, 26.6, 23.3, 22.7.

2.2.3 Synthesis of *α,α,α*-Trisubstituted Aldehyde

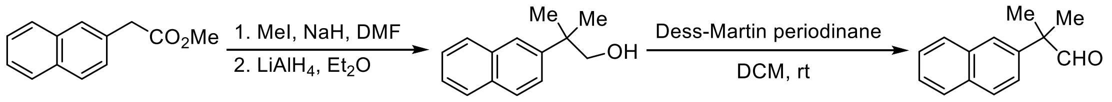

Step 1: To a solution of methyl 2-naphthylacetate (10.0 mmol, 2.0 g, 1.0 equiv.) in THF (60 mL), NaH (40.0 mmol, 1.6 g, 60% in mineral oil, 4.0 equiv.) was added in portions at 0 °C and the mixture was stirred at room temperature for 30 min. Then MeI (40.0 mmol, 2.5 mL, 4.0 equiv.) was slowly added to the above mixture at 0 °C. After stirring at room temperature for 6 h, the reaction was quenched by sat. NH_4Cl aqueous (100 mL), and the aqueous layer was extracted with EtOAc (60 mL). The organic layers were dried over Na_2SO_4 and concentrated in vacuo. The crude residue was dissolve in dry Et_2O (50 mL), and $LiAlH_4$ (13.0 mmol, 0.5 g, 1.3 equiv.) was slowly added in portions to the above mixture. The reaction mixture was stirred at room temperature for 6 h. Then H_2O (5 mL), NaOH (10%, 5 mL), H_2O (20 mL) were added successively at 0 °C. The mixture was filtered through Celite, washed with Et_2O (50 mL). The two phases were separated, and the organic layer was washed with brine, dried over $MgSO_4$ and concentrated under reduced pressure. Purification by flash chromatography provided the desired alcohol (1.89 g, 95% yield).

Step 2: To a solution of the above alcohol (5.0 mmol, 1.0 g, 1.0 equiv.) in DCM (10 mL) was added Dess-Martin periodinane (10.0 mmol, 4.2 g, 2.0 equiv.) dropwise over a period of 10 min. The reaction mixture was stirred for 30 min and the progress of the reaction was monitored by TLC. After the reaction was completed, the reaction mixture was quenched by the addition of sat. $NaHCO_3$ aqueous (26 mL), sat. $Na_2S_2O_3$ aqueous (26 mL), and Et_2O (50 mL). The mixture was stirred for 30 min whereupon the initially formed precipitate dissolved and the phases became clear. The two phases were separated, and the organic layer was washed with brine, dried over Na_2SO_4 and

concentrated under reduced pressure. Purification by flash chromatography provided the desired *α,α,α*-trisubstituted aldehyde (0.89 g, 90% yield).

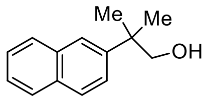

2-Methyl-2-(naphthalen-2-yl)propan-1-ol (7')

White solid. Known compound.[28] **^{1}H NMR (500 MHz, CDCl$_3$)** δ 7.84–7.80 (m, 4 H), 7.54 (dd, J_1 = 8.5 Hz, J_2 = 2.0 Hz, 1 H), 7.49–7.43 (m, 2 H), 3.72 (d, J = 6.5 Hz, 2 H), 1.44 (s, 6 H). **^{13}C NMR (125 MHz, CDCl$_3$)** δ 143.6, 133.3, 132.0, 128.1, 127.9, 127.4, 126.0, 125.7, 125.0, 124.6, 72.9, 40.3, 25.4.

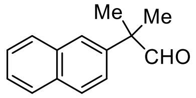

2-Methyl-2-(naphthalen-2-yl)propanal (7)

Colorless oil. Known compound.[28] **^{1}H NMR (500 MHz, CDCl$_3$)** δ 9.57 (s, 1 H), 7.86–7.81 (m, 3 H), 7.73 (d, J = 1.5 Hz, 1 H), 7.51–7.46 (m, 2 H), 7.37 (dd, J_1 = 8.5 Hz, J_2 = 2.0 Hz, 1 H), 1.57 (s, 6 H). **^{13}C NMR (125 MHz, CDCl$_3$)** δ 202.3, 138.6, 133.4, 132.4, 128.5, 128.0, 127.5, 126.4, 126.2, 125.5, 124.8, 50.6, 22.5.

2.3 Nickel-Catalyzed Hydroalkylation of Olefins with *N*-Sulfonyl Amines

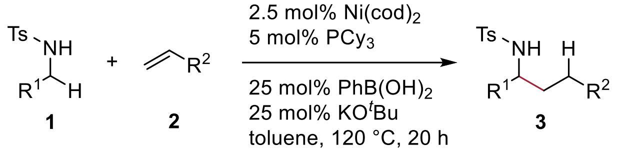

General Procedure A: In a nitrogen-filled glovebox, a 4 mL oven-dried vial was charged with *N*-tosyl amine **1** (0.2 mmol, 1.0 equiv.), olefin **2** (0.4 mmol, 2.0 equiv.), Ni(cod)$_2$ (0.005 mmol, 1.4 mg, 2.5 mol%), PCy$_3$ (0.01 mmol, 2.8 mg, 5 mol%), PhB(OH)$_2$ (0.05 mmol, 6.1 mg, 25 mol%) and KO^{t}Bu (0.05 mmol, 5.6 mg, 25 mol%). Toluene (0.3 mL) was added. The vial was equipped with a magnetic stir bar, sealed, and the reaction mixture was stirred at 120 °C for 20 h. The reaction mixture was cooled to room temperature and concentrated under reduced pressure. Purification by column chromatography afforded the desired product.

General Procedure B (for *N*-tosyl aliphatic amines): In a nitrogen-filled glovebox, a 4 mL oven-dried vial was charged with *N*-tosyl aliphatic amine **1** (0.2 mmol, 1.0 equiv.), styrene **2a** (0.6 mmol, 69 μL, 3.0 equiv.), Ni(cod)$_2$ (0.01 mmol, 2.8 mg, 5 mol%), PCy$_3$ (0.02 mmol, 5.6 mg, 10 mol%), PhB(OH)$_2$ (0.20 mmol, 24.4 mg, 100 mol%) and KO^{t}Bu (0.20 mmol, 22.4 mg, 100 mol%). Toluene (0.3 mL) was added. The vial was equipped with a magnetic stir bar, sealed, and the reaction mixture was stirred at 120 °C for 20 h. The reaction mixture was cooled to room temperature and concentrated under reduced pressure. Purification by column chromatography afforded the desired product.

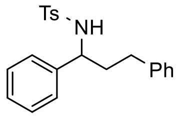

_N_-(1,3-Diphenylpropyl)-4-methylbenzenesulfonamide (3a)

Following the **General Procedure A**, the title compound was isolated as white solid (67.8 mg, 93% yield). Known compound.[29] **^{1}H NMR (500 MHz, CDCl$_3$)** δ 7.54 (d, J = 8.0 Hz, 2 H), 7.20–7.17 (m, 2 H), 7.14–7.08 (m, 4 H), 7.03–6.99 (m, 6 H), 6.01 (d, J = 8.0 Hz, 1 H), 4.28 (dd, J_1 = 15.0 Hz, J_2 = 7.5 Hz, 1 H), 2.58–2.52 (m, 1 H), 2.49–2.43 (m, 1 H), 2.29 (s, 3 H), 2.12–2.05 (m, 1 H), 1.99–1.92 (m, 1 H). **^{13}C NMR (125 MHz, CDCl$_3$)** δ 142.7, 140.8, 140.6, 137.5, 129.1, 128.24, 128.19, 127.1, 126.9, 126.5, 125.8, 57.8, 38.9, 32.0, 21.3. **HRMS** (ESI) m/z calcd. For $C_{22}H_{23}NNaO_2S$ [M+Na]$^+$: 388.1342, found: 388.1345.

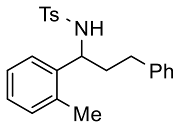

4-Methyl-_N_-(3-phenyl-1-(_o_-tolyl)propyl)benzenesulfonamide (3b)

Following the **General Procedure A**, the title compound was isolated as light yellow oil (72.5 mg, 96% yield). **^{1}H NMR (500 MHz, CDCl$_3$)** δ 7.51 (d, J = 8.5 Hz, 2 H), 7.23 (t, J = 7.5 Hz, 2 H), 7.16 (t, J = 7.5 Hz, 1 H), 7.06–7.05

(m, 5 H), 7.03–6.97 (m, 2 H), 6.95–6.93 (m, 1 H), 5.37 (d, J = 8.0 Hz, 1 H), 4.57 (dd, J_1 = 14.5 Hz, J_2 = 7.5 Hz, 1 H), 2.65–2.59 (m, 1 H), 2.55–2.49 (m, 1 H), 2.32 (s, 3 H), 2.13–2.05 (m, 1 H), 1.99 (s, 3 H), 1.97–1.89 (m, 1 H). **^{13}C NMR (125 MHz, $CDCl_3$)** δ 142.9, 140.9, 138.9, 137.5, 134.8, 130.3, 129.2, 128.4, 128.3, 127.0, 126.9, 126.3, 126.0, 125.5, 53.2, 38.8, 32.0, 21.4, 18.9. **HRMS** (ESI) m/z calcd. For $C_{23}H_{25}NNaO_2S$ $[M+Na]^+$: 402.1498, found: 402.1501.

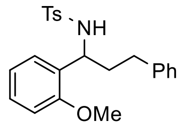

***N*-(1-(2-Methoxyphenyl)-3-phenylpropyl)-4-methylbenzenesulfonamide (3c)**

Following the **General Procedure A**, the title compound was isolated as light yellow oil (78.3 mg, 99% yield). Known compound.[29] **^{1}H NMR (500 MHz, $CDCl_3$)** δ 7.43 (d, J = 8.0 Hz, 2 H), 7.24 (t, J = 7.5 Hz, 2 H), 7.16 (t, J = 7.5 Hz, 1 H), 7.10 (d, J = 7.0 Hz, 2 H), 7.08–7.04 (m, 1 H), 6.97 (d, J = 8.0 Hz, 2 H), 6.78 (dd, J_1 = 7.5 Hz, J_2 = 1.5 Hz, 1 H), 6.68 (t, J = 7.5 Hz, 1 H), 6.58 (d, J = 8.5 Hz, 1 H), 5.67 (d, J = 10.0 Hz, 1 H), 4.37–4.32 (m, 1 H), 3.68 (s, 3 H), 2.72–2.66 (m, 1 H), 2.55–2.49 (m, 1 H), 2.27 (s, 3 H), 2.23–2.15 (m, 1 H), 2.05–1.97 (m, 1 H). **^{13}C NMR (125 MHz, $CDCl_3$)** δ 156.3, 142.5, 141.4, 137.6, 129.1, 128.8, 128.4, 128.3, 127.7, 126.8, 125.8, 120.4, 110.6, 57.4, 55.0, 37.3, 32.5, 21.3. **HRMS** (ESI) m/z calcd. For $C_{23}H_{25}NNaO_3S$ $[M+Na]^+$: 418.1447, found: 418.1452.

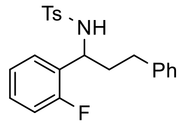

***N*-(1-(2-Fluorophenyl)-3-phenylpropyl)-4-methylbenzenesulfonamide (3d)**

Following the **General Procedure A**, the title compound was isolated as white solid (52.1 mg, 68% yield). **^{1}H NMR (500 MHz, $CDCl_3$)** δ 7.50 (d, J = 8.0 Hz, 2 H), 7.25 (t, J = 7.5 Hz, 2 H), 7.17 (t, J = 7.5 Hz, 1 H), 7.13–7.04 (m, 5 H), 6.98-6.90 (m, 2 H), 6.80 (dd, J_1 = 11.0 Hz, J_2 = 8.5 Hz, 1 H), 5.07 (d, J = 9.0 Hz, 1 H), 4.48 (dd, J_1 = 16.5 Hz, J_2 = 7.5 Hz, 1 H), 2.68–2.62 (m, 1 H), 2.55–2.49 (m, 1 H), 2.31 (s, 3 H), 2.18–2.10 (m, 1 H), 2.06–1.98 (m, 1 H). **^{13}C NMR (125 MHz, $CDCl_3$)** δ 160.1 (d, J = 243.8 Hz), 142.9, 140.6, 137.3, 129.2, 129.1 (d, J = 8.5 Hz), 128.9 (d, J = 4.8 Hz), 128.4, 128.3, 127.6 (d, J = 12.5 Hz), 126.9, 126.1, 124.1 (d, J = 3.3 Hz), 115.7 (d, J = 21.6 Hz), 54.3, 38.0, 32.2, 21.4. **^{19}F NMR (470 MHz, $CDCl_3$)** δ -118.3. **HRMS** (ESI) m/z calcd. For $C_{22}H_{22}FNNaO_2S$ $[M+Na]^+$: 406.1247, found: 406.1253.

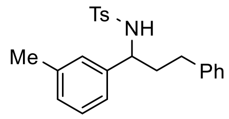

4-Methyl-*N*-(3-phenyl-1-(*m*-tolyl)propyl)benzenesulfonamide (3e)

Following the **General Procedure A**, the title compound was isolated as white solid (72.3 mg, 95% yield). **^{1}H NMR (500 MHz, $CDCl_3$)** δ 7.51 (d, J = 8.5 Hz, 2 H), 7.22 (t, J = 8.0 Hz, 2 H), 7.15 (t, J = 7.5 Hz, 1 H), 7.07–7.02 (m, 5

H), 6.92 (d, J = 8.0 Hz, 1 H), 6.82 (d, J = 7.5 Hz, 1 H), 6.70 (s, 1 H), 5.44 (d, J = 8.0 Hz, 1 H), 4.23 (dd, J_1 = 15.0 Hz, J_2 = 7.5 Hz, 1 H), 2.59–2.53 (m, 1 H), 2.51–2.45 (m, 1 H), 2.33 (s, 3 H), 2.16 (s, 3 H), 2.13–2.05 (m, 1 H), 2.01–1.94 (m, 1 H). **^{13}C NMR (125 MHz, CDCl$_3$)** δ 142.8, 141.0, 140.4, 137.9, 137.6, 129.1, 128.33, 128.30, 128.0, 127.3, 127.0 125.9, 123.5, 57.9, 38.9, 32.1, 21.3, 21.1. **HRMS** (ESI) m/z calcd. For $C_{23}H_{25}NNaO_2S$ [M+Na]$^+$: 402.1498, found: 402.1505.

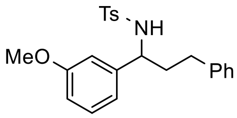

***N*-(1-(3-Methoxyphenyl)-3-phenylpropyl)-4-methylbenzenesulfonamide (3f)**

Following the **General Procedure A**, the title compound was isolated as light yellow oil (72.4 mg, 92% yield). Known compound.[29] **^{1}H NMR (500 MHz, CDCl$_3$)** δ 7.52 (d, J = 8.0 Hz, 2 H), 7.22 (t, J = 7.5 Hz, 2 H), 7.16 (t, J = 7.5 Hz, 1 H), 7.08–7.03 (m, 5 H), 6.67 (dd, J_1 = 8.0 Hz, J_2 = 2.0 Hz, 1 H), 6.61 (d, J = 7.5 Hz, 1 H), 6.49 (s, 1 H), 5.48 (d, J = 7.5 Hz, 1 H), 4.24 (dd, J_1 = 15.0 Hz, J_2 = 7.5 Hz, 1 H), 3.66 (s, 3 H), 2.59–2.46 (m, 2 H), 2.33 (s, 3 H), 2.13–2.06 (m, 1 H), 2.02–1.94 (m, 1 H). **^{13}C NMR (125 MHz, CDCl$_3$)** δ 159.5, 142.9, 142.1, 140.9, 137.5, 129.5, 129.2, 128.33, 128.32, 127.0, 125.9, 118.9, 113.0, 111.9, 57.9, 55.0, 38.9, 32.0, 21.4. **HRMS** (ESI) m/z calcd. For $C_{23}H_{25}NNaO_3S$ [M+Na]$^+$: 418.1447, found: 418.1451.

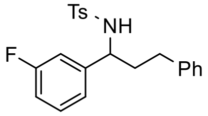

***N*-(1-(3-Fluorophenyl)-3-phenylpropyl)-4-methylbenzenesulfonamide (3g)**

Following the **General Procedure A**, the title compound was isolated as light yellow solid (66.8 mg, 87% yield). **^{1}H NMR (500 MHz, CDCl$_3$)** δ 7.53 (d, J = 8.0 Hz, 2 H), 7.23 (t, J = 7.5 Hz, 2 H), 7.18–7.10 (m, 4 H), 7.03 (d, J = 7.0 Hz, 2 H), 6.85–6.82 (m, 2 H), 6.67 (d, J = 9.5 Hz, 1 H), 5.35 (d, J = 6.5 Hz, 1 H), 4.28 (dd, J_1 = 13.5 Hz, J_2 = 6.5 Hz, 1 H), 2.59–2.45 (m, 2 H), 2.35 (s, 3 H), 2.11–2.03 (m, 1 H), 2.00–1.92 (m, 1 H). **^{13}C NMR (125 MHz, CDCl$_3$)** δ 162.7 (d, J = 245.0 Hz), 143.27, 143.22, 140.5, 137.4, 130.0 (d, J = 8.1 Hz), 129.3, 128.4, 128.3, 127.0, 126.1, 122.2 (d, J = 2.8 Hz), 114.3 (d, J = 21.0 Hz), 113.6 (d, J = 21.8 Hz), 57.3 (d, J = 1.6 Hz), 38.9, 31.9, 21.4. **^{19}F NMR (470 MHz, CDCl$_3$)** δ -112.8. **HRMS** (ESI) m/z calcd. For $C_{22}H_{22}FNNaO_2S$ [M+Na]$^+$: 406.1247, found: 406.1257.

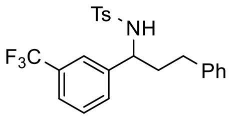

4-Methyl-*N*-(3-phenyl-1-(3-(trifluoromethyl)phenyl)propyl)benzenesulfonamide (3h)

Following the **General Procedure A**, the title compound was isolated as colorless oil (72.0 mg, 83% yield). **^{1}H NMR (500 MHz, CDCl$_3$)** δ 7.49 (d, J = 7.5 Hz, 2 H), 7.36 (t, J = 3.5 Hz, 1 H), 7.27–7.21 (m, 4 H), 7.18–7.15 (m, 1 H), 7.13 (s, 1 H), 7.05–7.03 (m, 4 H), 5.78–5.74 (m, 1 H), 4.37 (dd, J_1 = 14.5 Hz, J_2 = 7.5 Hz, 1 H), 2.63–2.57 (m, 1

H), 2.54–2.49 (m, 1 H), 2.31 (s, 3 H), 2.14–2.06 (m, 1 H), 2.00–1.93 (m, 1 H). **^{13}C NMR (125 MHz, CDCl$_3$)** δ 143.3, 141.6, 140.4, 137.2, 130.5 (q, J = 31.9 Hz), 130.0, 129.3, 129.0, 128.5, 128.3, 126.9, 126.1, 124.2 (q, J = 3.6 Hz), 123.8 (q, J = 270.8 Hz), 123.4 (q, J = 3.6 Hz), 57.4, 38.9, 32.0, 21.3. **^{19}F NMR (470 MHz, CDCl$_3$)** δ -62.7. **HRMS** (ESI) m/z calcd. For $C_{23}H_{22}F_3NNaO_2S$ [M+Na]$^+$: 456.1216, found: 456.1214.

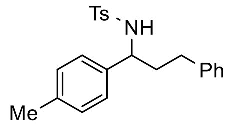

4-Methyl-*N*-(3-phenyl-1-(*p*-tolyl)propyl)benzenesulfonamide (3i)

Following the **General Procedure A**, the title compound was isolated as white solid (63.0 mg, 83% yield). Known compound.[29] **^{1}H NMR (500 MHz, CDCl$_3$)** δ 7.51 (d, J = 8.0 Hz, 2 H), 7.23 (t, J = 7.5 Hz, 2 H), 7.16 (t, J = 7.5 Hz, 1 H), 7.10 (d, J = 8.5 Hz, 2 H), 7.03 (d, J = 7.0 Hz, 2 H), 6.96 (d, J = 8.0 Hz, 2 H), 6.88 (d, J = 8.0 Hz, 2 H), 5.06 (d, J = 7.0 Hz, 1 H), 4.22 (dd, J_1 = 14.5 Hz, J_2 = 7.0 Hz, 1 H), 2.55–2.44 (m, 2 H), 2.36 (s, 3 H), 2.27 (s, 3 H), 2.15–2.08 (m, 1 H), 2.03–1.95 (m, 1 H). **^{13}C NMR (125 MHz, CDCl$_3$)** δ 142.9, 140.9, 137.6, 137.5, 137.2, 129.2, 129.1, 128.3, 127.1, 126.5, 125.9, 57.6, 38.8, 32.0, 21.4, 21.0. **HRMS** (ESI) m/z calcd. For $C_{23}H_{25}NNaO_2S$ [M+Na]$^+$: 402.1498, found: 402.1505.

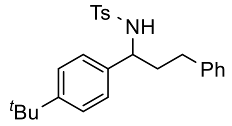

***N*-(1-(4-(*tert*-Butyl)phenyl)-3-phenylpropyl)-4-methylbenzenesulfonamide (3j)**

Following the **General Procedure A**, the title compound was isolated as white solid (79.7 mg, 95% yield). **^{1}H NMR (500 MHz, CDCl$_3$)** δ 7.47 (d, J = 8.5 Hz, 2 H), 7.23 (t, J = 7.5 Hz, 2 H), 7.16 (t, J = 7.5 Hz, 1 H), 7.12 (d, J = 8.5 Hz, 2 H), 7.06–7.03 (m, 4 H), 6.90 (d, J = 8.0 Hz, 2 H), 5.24 (d, J = 7.5 Hz, 1 H), 4.27 (dd, J_1 = 14.5 Hz, J_2 = 7.5 Hz, 1 H), 2.61–2.48 (m, 2 H), 2.31 (s, 3 H), 2.15–2.07 (m, 1 H), 2.03–1.96 (m, 1 H), 1.25 (s, 9 H). **^{13}C NMR (125 MHz, CDCl$_3$)** δ 150.3, 142.6, 141.0, 137.6, 137.3, 129.1, 128.4, 128.3, 127.0, 126.2, 125.9, 125.2, 57.6, 38.9, 34.3, 32.1, 31.3, 21.4. **HRMS** (ESI) m/z calcd. For $C_{26}H_{31}NNaO_2S$ [M+Na]$^+$: 444.1968, found: 444.1971.

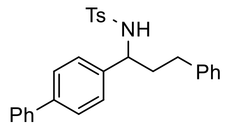

***N*-(1-([1,1'-Biphenyl]-4-yl)-3-phenylpropyl)-4-methylbenzenesulfonamide (3k)**

Following the **General Procedure A**, the title compound was isolated as light yellow solid (79.4 mg, 90% yield). Known compound.[29] **^{1}H NMR (500 MHz, CDCl$_3$)** δ 7.53 (d, J = 8.5 Hz, 2 H), 7.48 (d, J = 7.5 Hz, 2 H), 7.41 (t, J = 7.5 Hz, 2 H), 7.35–7.31 (m, 3 H), 7.24–7.21 (m, 2 H), 7.16 (t, J = 7.5 Hz, 1 H), 7.08–7.04 (m, 6 H), 5.54 (d, J = 8.0 Hz, 1 H), 4.34 (dd, J_1 = 15.0 Hz, J_2 = 7.5 Hz, 1 H), 2.62–2.50 (m, 2 H), 2.27 (s, 3 H), 2.18–2.10 (m, 1 H), 2.06–

1.99 (m, 1 H). **^{13}C NMR (125 MHz, $CDCl_3$)** δ 142.9, 140.8, 140.6, 140.3, 139.5, 137.6, 129.2, 128.7, 128.4, 127.3, 127.08, 127.05, 126.9, 126.0, 57.6, 38.8, 32.1, 21.4. **HRMS** (ESI) m/z calcd. For $C_{28}H_{27}NNaO_2S$ $[M+Na]^+$: 464.1655, found: 464.1660.

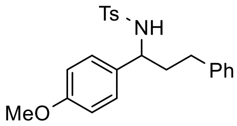

***N*-(1-(4-Methoxyphenyl)-3-phenylpropyl)-4-methylbenzenesulfonamide (3l)**

Following the **General Procedure A**, the title compound was isolated as light yellow solid (70.3 mg, 89% yield). Known compound.[29] **^{1}H NMR (500 MHz, $CDCl_3$)** δ 7.51 (d, J = 8.0 Hz, 2 H), 7.22 (t, J = 7.5 Hz, 2 H), 7.15 (t, J = 7.5 Hz, 1 H), 7.09 (d, J = 8.0 Hz, 2 H), 7.02 (d, J = 7.5 Hz, 2 H), 6.92 (d, J = 8.0 Hz, 2 H), 6.66 (d, J = 8.5 Hz, 2 H), 5.41 (d, J = 7.5 Hz, 1 H), 4.21 (dd, J_1 = 15.0 Hz, J_2 = 7.5 Hz, 1 H), 3.73 (s, 3 H), 2.54–2.42 (m, 2 H), 2.34 (s, 3 H), 2.14–2.06 (m, 1 H), 2.00–1.93 (m, 1 H). **^{13}C NMR (125 MHz, $CDCl_3$)** δ 158.8, 142.8, 140.9, 137.6, 132.6, 129.2, 128.31, 128.30, 127.7, 127.0, 125.9, 113.8, 57.3, 55.2, 38.8, 32.0, 21.4. **HRMS** (ESI) m/z calcd. For $C_{23}H_{25}NNaO_3S$ $[M+Na]^+$: 418.1447, found: 418.1448.

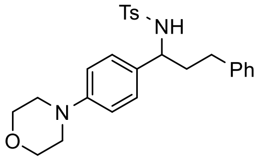

4-Methyl-*N*-(1-(4-morpholinophenyl)-3-phenylpropyl)benzenesulfonamide (3m)

Following the **General Procedure A**, but Ni(cod)$_2$ (0.01 mmol, 2.8 mg, 5 mol%), PCy$_3$ (0.02 mmol, 5.6 mg, 10 mol%), PhB(OH)$_2$ (0.2 mmol, 24.4 mg, 100 mol%) and KO^{t}Bu (0.1 mmol, 11.2 mg, 50 mol%) were used. The title compound was isolated as light yellow solid (71.7 mg, 80% yield). **^{1}H NMR (500 MHz, $CDCl_3$)** δ 7.51 (d, J = 8.0 Hz, 2 H), 7.23 (t, J = 7.5 Hz, 2 H), 7.16 (t, J = 7.5 Hz, 1 H), 7.10 (d, J = 8.0 Hz, 2 H), 7.04 (d, J = 7.5 Hz, 2 H), 6.90 (d, J = 8.0 Hz, 2 H), 6.69 (d, J = 8.0 Hz, 2 H), 4.98 (d, J = 7.0 Hz, 1 H), 4.19 (dd, J_1 = 14.0 Hz, J_2 = 7.0 Hz, 1 H), 3.84 (t, J = 4.5 Hz, 4 H), 3.08 (t, J = 4.5 Hz, 4 H), 2.53–2.43 (m, 2 H), 2.36 (s, 3 H), 2.16–2.08 (m, 1 H), 2.03–1.95 (m, 1 H). **^{13}C NMR (125 MHz, $CDCl_3$)** δ 150.5, 142.7, 141.0, 137.7, 131.7, 129.3, 128.4, 127.5, 127.1, 125.9, 115.6, 66.9, 57.3, 49.3, 38.7, 32.1, 21.5. **HRMS** (ESI) m/z calcd. For $C_{26}H_{31}N_2O_3S$ $[M+H]^+$: 451.2050, found: 451.2071.

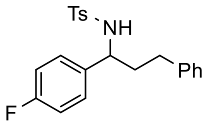

***N*-(1-(4-Fluorophenyl)-3-phenylpropyl)-4-methylbenzenesulfonamide (3n)**

Following the **General Procedure A**, the title compound was isolated as white solid (69.2 mg, 90% yield). Known compound.[29] **^{1}H NMR (500 MHz, $CDCl_3$)** δ 7.51 (d, J = 8.0 Hz, 2 H), 7.23 (t, J = 8.0 Hz, 2 H), 7.17 (t, J = 7.5 Hz,

1 H), 7.10 (d, J = 8.0 Hz, 2 H), 7.02 (d, J = 7.5 Hz, 2 H), 6.99–6.96 (m, 2 H), 6.82 (t, J = 8.5 Hz, 2 H), 5.43 (d, J = 7.5 Hz, 1 H), 4.27 (dd, J_1 = 14.5 Hz, J_2 = 7.5 Hz, 1 H), 2.56–2.44 (m, 2 H), 2.36 (s, 3 H), 2.12–2.05 (m, 1 H), 1.99–1.91 (m, 1 H). **^{13}C NMR (125 MHz, $CDCl_3$)** δ 162.0 (d, J = 244.5 Hz), 143.1, 140.6, 137.5, 136.4 (d, J = 3.3 Hz), 129.3, 128.4, 128.3, 128.2 (d, J = 8.1 Hz), 127.0, 126.0, 115.2 (d, J = 21.4 Hz), 57.1, 38.9, 32.0, 21.4. **^{19}F NMR (470 MHz, $CDCl_3$)** δ -114.9. **HRMS** (ESI) m/z calcd. For $C_{22}H_{22}FNNaO_2S$ $[M+Na]^+$: 406.1247, found: 406.1254.

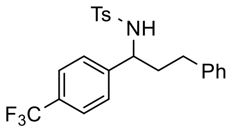

4-Methyl-*N*-(3-phenyl-1-(4-(trifluoromethyl)phenyl)propyl)benzenesulfonamide (3o)

Following the **General Procedure A**, the title compound was isolated as white solid (60.1 mg, 69% yield). Known compound.[29] **^{1}H NMR (500 MHz, $CDCl_3$)** δ 7.46 (d, J = 7.5 Hz, 2 H), 7.33 (d, J = 8.0 Hz, 2 H), 7.23 (t, J = 7.5 Hz, 2 H), 7.17 (t, J = 7.5 Hz, 1 H), 7.10 (d, J = 8.0 Hz, 2 H), 7.04–7.02 (m, 4 H), 5.73 (s, 1 H), 4.36 (dd, J_1 = 14.5 Hz, J_2 = 7.0 Hz, 1 H), 2.61–2.55 (m, 1 H), 2.53–2.47 (m, 1 H), 2.32 (s, 3 H), 2.13–2.06 (m, 1 H), 2.00–1.93 (m, 1 H). **^{13}C NMR (125 MHz, $CDCl_3$)** δ 144.5, 143.3, 140.3, 137.2, 129.5 (q, J = 32.4 Hz), 129.3, 128.5, 128.3, 127.05, 126.97, 126.2, 125.3 (q, J = 3.6 Hz), 123.9 (q, J = 270.3 Hz), 57.4, 38.8, 31.9, 21.2. **^{19}F NMR (470 MHz, $CDCl_3$)** δ -62.6. **HRMS** (ESI) m/z calcd. For $C_{23}H_{22}F_3NNaO_2S$ $[M+Na]^+$: 456.1216, found: 456.1207.

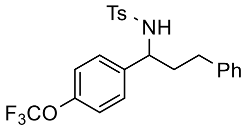

4-Methyl-*N*-(3-phenyl-1-(4-(trifluoromethoxy)phenyl)propyl)benzenesulfonamide (3p)

Following the **General Procedure A**, the title compound was isolated as white solid (80.6 mg, 90% yield). Known compound.[29] **^{1}H NMR (500 MHz, $CDCl_3$)** δ 7.48 (d, J = 8.0 Hz, 2 H), 7.23 (t, J = 7.5 Hz, 2 H), 7.17 (t, J = 7.5 Hz, 1 H), 7.06–7.01 (m, 6 H), 6.94 (d, J = 8.0 Hz, 2 H), 5.64 (d, J = 7.5 Hz, 1 H), 4.33 (dd, J_1 = 15.0 Hz, J_2 = 7.5 Hz, 1 H), 2.61–2.55 (m, 1 H), 2.53–2.47 (m, 1 H), 2.32 (s, 3 H), 2.12–2.05 (m, 1 H), 1.98–1.91 (m, 1 H). **^{13}C NMR (125 MHz, $CDCl_3$)** δ 148.2 (q, J = 1.8 Hz), 143.3, 140.5, 139.4, 137.4, 129.2, 128.4, 128.3, 128.0, 127.0, 126.1, 120.9, 120.3 (q, J = 255.4 Hz), 57.1, 39.0, 32.0, 21.3. **^{19}F NMR (470 MHz, $CDCl_3$)** δ -58.0. **HRMS** (ESI) m/z calcd. For $C_{23}H_{22}F_3NNaO_3S$ $[M+Na]^+$: 472.1165, found: 472.1161.

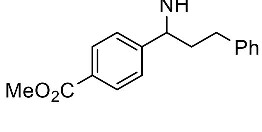

Methyl 4-(1-((4-methylphenyl)sulfonamido)-3-phenylpropyl)benzoate (3q)

Following the **General Procedure A**, the title compound was isolated as white solid (42.0 mg, 50% yield). **^{1}H NMR (500 MHz, $CDCl_3$)** δ 7.80 (d, J = 8.0 Hz, 2 H), 7.51 (d, J = 8.5 Hz, 2 H), 7.22 (t, J = 7.5 Hz, 2 H), 7.16 (t, J = 7.5

Hz, 1 H), 7.10–7.06 (m, 4 H), 7.01 (d, *J* = 7.0 Hz, 2 H), 5.68 (d, *J* = 8.0 Hz, 1 H), 4.34 (dd, J_1 = 14.5 Hz, J_2 = 7.5 Hz, 1 H), 3.89 (s, 3 H), 2.59–2.53 (m, 1 H), 2.51–2.45 (m, 1 H), 2.32 (s, 3 H), 2.12–2.05 (m, 1 H), 2.00–1.93 (m, 1 H). **^{13}C NMR (125 MHz, CDCl$_3$)** δ 166.7, 145.8, 143.2, 140.4, 137.3, 129.7, 129.3, 129.1, 128.4, 128.3, 127.0, 126.6, 126.1, 57.5, 52.1, 38.8, 31.9, 21.3. **HRMS** (ESI) m/z calcd. For $C_{24}H_{25}NNaO_4S$ [M+Na]$^+$: 446.1397, found: 446.1403.

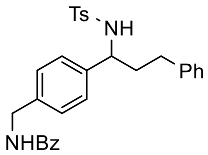

***N*-(4-(1-((4-Methylphenyl)sulfonamido)-3-phenylpropyl)benzyl)benzamide (3r)**

Following the **General Procedure A**, the title compound was isolated as white solid (42.3 mg, 42% yield). **^{1}H NMR (500 MHz, CDCl$_3$)** δ 7.79 (d, *J* = 7.5 Hz, 2 H), 7.53–7.48 (m, 3 H), 7.42 (t, *J* = 7.5 Hz, 2 H), 7.22 (t, *J* = 7.5 Hz, 2 H), 7.17–7.15 (m, 3 H), 7.10 (d, *J* = 8.5 Hz, 2 H), 7.03–6.99 (m, 4 H), 6.51 (t, *J* = 5.0 Hz, 1 H), 5.28 (t, *J* = 7.5 Hz, 1 H), 4.56 (d, *J* = 5.5 Hz, 2 H), 4.24 (dd, J_1 = 14.5 Hz, J_2 = 6.0 Hz, 1 H), 2.52–2.41 (m, 2 H), 2.33 (s, 3 H), 2.10–2.03 (m, 1 H), 2.00–1.93 (m, 1 H). **^{13}C NMR (125 MHz, CDCl$_3$)** δ 167.3, 143.1, 140.7, 140.2, 137.54, 137.47, 134.2, 131.6, 129.3, 128.6, 128.4, 128.3, 128.0, 127.03, 126.97, 126.95, 126.0, 57.5, 43.6, 38.8, 32.0, 21.4. **HRMS** (ESI) m/z calcd. For $C_{30}H_{30}N_2NaO_3S$ [M+Na]$^+$: 521.1869, found: 521.1881.

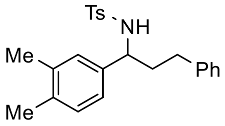

***N*-(1-(3,4-Dimethylphenyl)-3-phenylpropyl)-4-methylbenzenesulfonamide (3s)**

Following the **General Procedure A**, the title compound was isolated as white solid (70.1 mg, 89% yield). **^{1}H NMR (500 MHz, CDCl$_3$)** δ 7.50 (d, *J* = 8.0 Hz, 2 H), 7.23 (t, *J* = 7.5 Hz, 2 H), 7.16 (t, *J* = 7.5 Hz, 1 H), 7.08 (d, *J* = 8.5 Hz, 2 H), 7.04 (d, *J* = 7.0 Hz, 2 H), 6.92 (d, *J* = 8.0 Hz, 1 H), 6.75 (dd, J_1 = 7.5 Hz, J_2 = 1.5 Hz, 1 H), 6.65 (s, 1 H), 5.12 (d, *J* = 7.5 Hz, 1 H), 4.19 (dd, J_1 = 15.0 Hz, J_2 = 7.5 Hz, 1 H), 2.57–2.45 (m, 2 H), 2.35 (s, 3 H), 2.16 (s, 3 H), 2.14–2.08 (m, 4 H), 2.03–1.95 (m, 1 H). **^{13}C NMR (125 MHz, CDCl$_3$)** δ 142.7, 141.0, 137.8, 137.6, 136.5, 135.7, 129.6, 129.1, 128.35, 128.31, 127.9, 127.1, 125.9, 123.9, 57.7, 38.7, 32.1, 21.4, 19.6, 19.3. **HRMS** (ESI) m/z calcd. For $C_{24}H_{27}NNaO_2S$ [M+Na]$^+$: 416.1655, found: 416.1651.

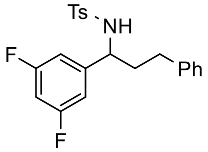

***N*-(1-(3,5-Difluorophenyl)-3-phenylpropyl)-4-methylbenzenesulfonamide (3t)**

Following the **General Procedure A**, the title compound was isolated as white solid (53.8 mg, 67% yield). **^{1}H NMR (500 MHz, CDCl$_3$)** δ 7.54 (d, *J* = 8.0 Hz, 2 H), 7.25 (t, *J* = 7.5 Hz, 2 H), 7.20–7.14 (m, 3 H), 7.03 (d, *J* = 7.0 Hz, 2 H), 6.60–6.53 (m, 3 H), 5.16 (s, 1 H), 4.27 (dd, J_1 = 13.5Hz, J_2 = 6.5 Hz, 1 H), 2.60–2.46 (m, 2 H), 2.37 (s, 3 H), 2.07–2.00 (m, 1 H), 1.97–1.90 (m, 1 H). **^{13}C NMR (125 MHz, CDCl$_3$)** δ 162.9 (dd, J_1 = 247.9 Hz, J_2 = 12.5 Hz), 144.8 (t, *J* = 8.2 Hz), 143.5, 140.2, 137.2, 129.4, 128.5, 128.3, 127.0, 126.2, 109.6 (dd, J_1 = 19.5 Hz, J_2 = 6.0 Hz), 102.7 (t, *J* = 25.1 Hz), 57.1, 38.7, 31.9, 21.4. **^{19}F NMR (470 MHz, CDCl$_3$)** δ -109.2. **HRMS** (ESI) m/z calcd. For $C_{22}H_{21}F_2NNaO_2S$ [M+Na]$^+$: 424.1153, found: 424.1157.

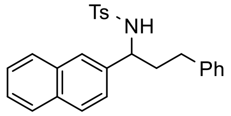

4-Methyl-*N*-(1-(naphthalen-2-yl)-3-phenylpropyl)benzenesulfonamide (3u)

Following the **General Procedure A**, the title compound was isolated as white solid (72.3 mg, 87% yield). Known compound.[29] **^{1}H NMR (500 MHz, CDCl$_3$)** δ 7.70–7.69 (m, 1 H), 7.58 (d, *J* = 8.0 Hz, 2 H), 7.48–7.46 (m, 2 H), 7.41–7.38 (m, 2 H), 7.33 (s, 1 H), 7.21–7.12 (m, 4 H), 7.02 (d, *J* = 7.5 Hz, 2 H), 6.82 (d, *J* = 8.0 Hz, 2 H), 5.93–5.78 (m, 1 H), 4.46–4.43 (m, 1 H), 2.60–2.57 (m, 1 H), 2.53–2.48 (m, 1 H), 2.22–2.15 (m, 1 H), 2.08–2.02 (m, 4 H). **^{13}C NMR (125 MHz, CDCl$_3$)** δ 142.8, 140.9, 137.4, 132.9, 132.6, 129.0, 128.4, 128.33, 128.30, 127.7, 127.4, 126.9, 126.02, 125.97, 125.90, 125.8, 124.0, 58.1, 38.6, 32.0, 21.1. **HRMS** (ESI) m/z calcd. For $C_{26}H_{25}NNaO_2S$ [M+Na]$^+$: 438.1498, found: 438.1514.

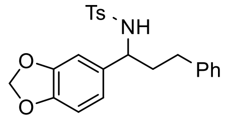

***N*-(1-(Benzo[*d*][1,3]dioxol-5-yl)-3-phenylpropyl)-4-methylbenzenesulfonamide (3v)**

Following the **General Procedure A**, the title compound was isolated as light yellow oil (63.1 mg, 77% yield). **^{1}H NMR (500 MHz, CDCl$_3$)** δ 7.53 (d, *J* = 8.0 Hz, 2 H), 7.23 (t, *J* = 7.5 Hz, 2 H), 7.16 (t, *J* = 7.5 Hz, 1 H), 7.12 (d, *J* = 8.0 Hz, 2 H), 7.03 (d, *J* = 7.5 Hz, 2 H), 6.58 (d, *J* = 8.0 Hz, 1 H), 6.49 (dd, J_1 = 8.0 Hz, J_2 = 2.0 Hz, 1 H), 6.45 (d, *J* = 1.5 Hz, 1 H), 5.86 (dd, J_1 = 10.0 Hz, J_2 = 1.0 Hz, 2 H), 5.32 (d, *J* = 7.5 Hz, 1 H), 4.18 (dd, J_1 = 14.5 Hz, J_2 = 7.5 Hz, 1 H), 2.55–2.43 (m, 2 H), 2.36 (s, 3 H), 2.11–2.04 (m, 1 H), 1.97–1.90 (m, 1 H). **^{13}C NMR (125 MHz, CDCl$_3$)** δ 147.6, 146.8, 142.9, 140.8, 137.6, 134.4, 129.2, 128.35, 128.31, 127.1, 126.0, 120.3, 108.0, 106.8, 100.9, 57.7, 38.8, 32.0, 21.4. **HRMS** (ESI) m/z calcd. For $C_{23}H_{23}NNaO_4S$ [M+Na]$^+$: 432.1240, found: 432.1249.

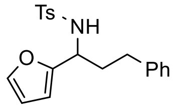

***N*-(1-(Furan-2-yl)-3-phenylpropyl)-4-methylbenzenesulfonamide (3w)**

Following the **General Procedure A**, but Ni(cod)$_2$ (0.01 mmol, 2.8 mg, 5 mol%), PCy$_3$ (0.02 mmol, 5.6 mg, 10 mol%), PhB(OH)$_2$ (0.2 mmol, 24.4 mg, 100 mol%), KO^{t}Bu (0.1 mmol, 11.2 mg, 50 mol%) were used and pivaldehyde (0.4 mmol, 44 μL, 2.0 equiv.) was added as an additive. The title compound was isolated as white solid (43.7 mg, 62% yield). Known compound.[30] **^{1}H NMR (500 MHz, CDCl$_3$)** δ 7.59 (d, *J* = 8.0 Hz, 2 H), 7.26–7.24 (m, 2 H), 7.19–7.14 (m, 4 H), 7.08 (d, *J* = 7.0 Hz, 2 H), 6.11 (dd, J_1 = 3.0 Hz, J_2 = 2.0 Hz, 1 H), 5.90 (d, *J* = 3.0 Hz, 1 H), 5.04 (d, *J* = 9.0 Hz, 1 H), 4.41 (dd, J_1 = 15.5 Hz, J_2 = 7.5 Hz, 1 H), 2.62–2.50 (m, 2 H), 2.37 (s, 3 H), 2.08 (dd, J_1 = 15.0 Hz, J_2 = 7.5 Hz, 2 H). **^{13}C NMR (125 MHz, CDCl$_3$)** δ 152.6, 143.0, 141.9, 140.7, 137.6, 129.3, 128.38, 128.37, 126.9, 126.0, 110.0, 107.0, 51.2, 36.4, 31.8, 21.4. **HRMS** (ESI) m/z calcd. For $C_{20}H_{21}NNaO_3S$ [M+Na]$^+$: 378.1134, found: 378.1135.

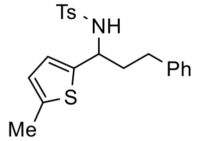

4-Methyl-*N*-(1-(5-methylthiophen-2-yl)-3-phenylpropyl)benzenesulfonamide (3x)

Following the **General Procedure A**, but Ni(cod)$_2$ (0.01 mmol, 2.8 mg, 5 mol%), PCy$_3$ (0.02 mmol, 5.6 mg, 10 mol%), PhB(OH)$_2$ (0.2 mmol, 24.4 mg, 100 mol%), KO^{t}Bu (0.1 mmol, 11.2 mg, 50 mol%) were used and pivaldehyde (0.4 mmol, 44 μL, 2.0 equiv.) was added as an additive. The title compound was isolated as yellow solid (38.8 mg, 50% yield). **^{1}H NMR (500 MHz, CDCl$_3$)** δ 7.59 (d, *J* = 8.0 Hz, 2 H), 7.26–7.24 (m, 2 H), 7.19–7.17 (m, 3 H), 7.07 (d, *J* = 7.0 Hz, 2 H), 6.45–6.42 (m, 2 H), 4.90 (d, *J* = 7.5 Hz, 1 H), 4.48 (dd, J_1 = 14.5 Hz, J_2 = 7.0 Hz, 1 H), 2.61–2.54 (m, 2 H), 2.39 (s, 3 H), 2.35 (s, 3 H), 2.18–2.11 (m, 1 H), 2.09–2.02 (m, 1 H). **^{13}C NMR (125 MHz, CDCl$_3$)** δ 143.0, 141.7, 140.8, 139.5, 137.6, 129.3, 128.4, 127.1, 126.0, 125.2, 124.5, 53.5, 39.3, 32.0, 21.5, 15.2. **HRMS** (ESI) m/z calcd. For $C_{21}H_{23}NNaO_2S_2$ [M+Na]$^+$: 408.1062, found: 408.1068.

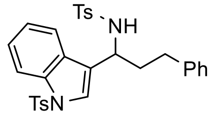

4-Methyl-*N*-(3-phenyl-1-(1-tosyl-1*H*-indol-3-yl)propyl)benzenesulfonamide (3y)

Following the **General Procedure A**, but Ni(cod)$_2$ (0.01 mmol, 2.8 mg, 5 mol%), PCy$_3$ (0.02 mmol, 5.6 mg, 10 mol%), PhB(OH)$_2$ (0.2 mmol, 24.4 mg, 100 mol%), KO^{t}Bu (0.1 mmol, 11.2 mg, 50 mol%) were used and pivaldehyde (0.4 mmol, 44 μL, 2.0 equiv.) was added as an additive. The title compound was isolated as brown oil (51.3 mg, 46% yield). **^{1}H NMR (500 MHz, CDCl$_3$)** δ 7.83 (d, *J* = 8.5 Hz, 1 H), 7.72 (d, *J* = 8.0 Hz, 2 H), 7.42 (d, *J* = 8.0 Hz, 2 H), 7.28 (s, 1 H), 7.24–7.16 (m, 7 H), 7.07 (t, *J* = 7.5 Hz, 1 H), 6.97 (d, *J* = 7.0 Hz, 2 H), 6.89 (d, *J* = 7.5 Hz, 2 H), 5.25 (s, 1 H), 4.51 (dd, J_1 = 13.5 Hz, J_2 = 6.5 Hz, 1 H), 2.50 (t, *J* = 7.5 Hz, 2 H), 2.31 (s, 3 H), 2.27 (s, 3 H), 2.23–2.10 (m, 2 H). **^{13}C NMR (125 MHz, CDCl$_3$)** δ 145.0, 143.1, 140.5, 136.9, 135.1, 129.9, 129.0, 128.37, 128.34, 128.1, 126.83, 126.80, 126.1, 124.7, 124.0, 123.0, 121.3, 119.8, 113.5, 50.4, 36.9, 32.1, 21.5, 21.3. **HRMS** (ESI) m/z calcd. For $C_{31}H_{30}N_2NaO_4S_2$ [M+Na]$^+$: 581.1539, found: 581.1548.

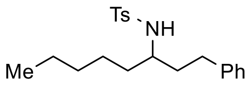

4-Methyl-*N*-(1-phenyloctan-3-yl)benzenesulfonamide (3z)

Following the **General Procedure B**, the title compound was isolated as white solid (42.6 mg, 59% yield). **^{1}H NMR (500 MHz, CDCl$_3$)** δ 7.74 (d, *J* = 8.0 Hz, 2 H), 7.28 (d, *J* = 8.0 Hz, 2 H), 7.24 (t, *J* = 7.5 Hz, 2 H), 7.16 (t, *J* = 7.5 Hz, 1 H), 7.03 (d, *J* = 7.5 Hz, 2 H), 4.50 (d, *J* = 8.5 Hz, 1 H), 3.29-3.22 (m, 1 H), 2.59–2.46 (m, 2 H), 2.42 (s, 3 H), 1.76–1.69 (m, 1 H), 1.66–1.58 (m, 1 H), 1.45–1.38 (m, 1 H), 1.34–1.27 (m, 1 H), 1.20–1.06 (m, 6 H), 0.80 (t, *J* = 7.0 Hz, 3 H). **^{13}C NMR (125 MHz, CDCl$_3$)** δ 143.1, 141.5, 138.3, 129.6, 128.33, 128.28, 127.0, 125.9, 53.8, 36.8, 34.8, 31.6, 31.4, 24.8, 22.4, 21.5, 13.9. **HRMS** (ESI) m/z calcd. For $C_{21}H_{29}NNaO_2S$ $[M+Na]^+$: 382.1811, found: 382.1809.

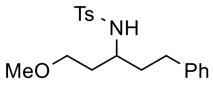

***N*-(1-Methoxy-5-phenylpentan-3-yl)-4-methylbenzenesulfonamide (3aa)**

Following the **General Procedure B**, the title compound was isolated as white solid (31.2 mg, 45% yield). **^{1}H NMR (500 MHz, CDCl$_3$)** δ 7.73 (d, *J* = 8.5 Hz, 2 H), 7.29–7.24 (m, 4 H), 7.18 (t, *J* = 7.5 Hz, 1 H), 7.09 (d, *J* = 8.0 Hz, 2 H), 5.28 (d, *J* = 7.0 Hz, 1 H), 3.45–3.40 (m, 1 H), 3.38–3.33 (m, 1 H), 3.27–3.24 (m, 4 H), 2.65–2.53 (m, 2 H), 2.42 (s, 3 H), 1.85–1.72 (m, 2 H), 1.71–1.64 (m, 1 H), 1.56–1.50 (m, 1 H). **^{13}C NMR (125 MHz, CDCl$_3$)** δ 143.0, 141.5, 138.1, 129.5, 128.4, 127.1, 125.9, 69.7, 58.8, 52.7, 36.6, 33.2, 31.8, 21.5. **HRMS** (ESI) m/z calcd. For $C_{19}H_{25}NNaO_3S$ $[M+Na]^+$: 370.1447, found: 370.1448.

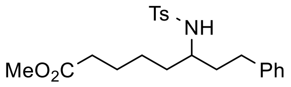

Methyl 6-((4-methylphenyl)sulfonamido)-8-phenyloctanoate (3ab)

Following the **General Procedure B**, the title compound was isolated as colorless oil (41.8 mg, 52% yield). **^{1}H NMR (500 MHz, CDCl$_3$)** δ 7.73 (d, *J* = 8.5 Hz, 2 H), 7.28 (d, *J* = 8.5 Hz, 2 H), 7.24 (t, *J* = 7.5 Hz, 2 H), 7.17 (t, *J* = 7.5 Hz, 1 H), 7.01 (d, *J* = 7.5 Hz, 2 H), 4.48 (d, *J* = 8.5 Hz, 1 H), 3.66 (s, 3 H), 3.29–3.23 (m, 1 H), 2.57–2.51 (m, 1 H), 2.49–2.43 (m, 4 H), 2.18 (t, *J* = 7.5 Hz, 2 H), 1.73–1.66 (m, 1 H), 1.63–1.56 (m, 1 H), 1.50–1.42 (m, 3 H), 1.38–1.28 (m, 1 H), 1.27–1.11 (m, 2 H). **^{13}C NMR (125 MHz, CDCl$_3$)** δ 173.8, 143.3, 141.3, 138.3, 129.6, 128.4, 128.2, 127.0, 125.9, 53.6, 51.5, 36.8, 34.6, 33.7, 31.6, 24.7, 24.5, 21.5. **HRMS** (ESI) m/z calcd. For $C_{22}H_{29}NNaO_4S$ $[M+Na]^+$: 426.1710, found: 426.1711.

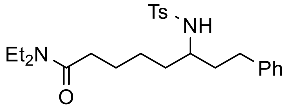

***N,N*-Diethyl-6-((4-methylphenyl)sulfonamido)-8-phenyloctanamide (3ac)**

Following the **General Procedure B**, the title compound was isolated as colorless oil (39.6 mg, 45% yield). **^{1}H NMR (500 MHz, CDCl$_3$)** δ 7.73 (d, *J* = 8.5 Hz, 2 H), 7.28 (d, *J* = 8.0 Hz, 2 H), 7.24–7.21 (m, 2 H), 7.16 (t, *J* = 7.5 Hz, 1 H), 7.00 (d, *J* = 7.0 Hz, 2 H), 4.62 (d, *J* = 8.5 Hz, 1 H), 3.36 (q, *J* = 7.0 Hz, 2 H), 3.29–3.25 (m, 3 H), 2.54–2.42 (m, 5 H), 2.22–2.19 (m, 2 H), 1.73–1.66 (m, 1 H), 1.62–1.45 (m, 4 H), 1.42–1.36 (m, 1 H), 1.35–1.27 (m, 1 H), 1.26–1.19 (m, 1 H), 1.15 (t, *J* = 7.0 Hz, 3 H), 1.10 (t, *J* = 7.0 Hz, 3 H). **^{13}C NMR (125 MHz, CDCl$_3$)** δ 171.8, 143.1, 141.3, 138.4, 129.6, 128.3, 128.2, 127.0, 125.9, 53.7, 41.9, 40.0, 36.8, 34.8, 32.8, 31.6, 25.0, 21.5, 14.4, 13.1. **HRMS** (ESI) m/z calcd. For $C_{25}H_{36}N_2NaO_3S$ $[M+Na]^+$: 467.2339, found: 467.2355.

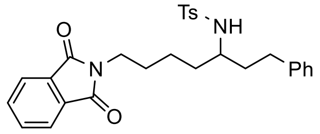

_N_-(7-(1,3-Dioxoisoindolin-2-yl)-1-phenylheptan-3-yl)-4-methylbenzenesulfonamide (3ad)

Following the **General Procedure B**, the title compound was isolated as white solid (64.3 mg, 66% yield). **^{1}H NMR (500 MHz, CDCl$_3$)** δ 7.87–7.84 (m, 2 H), 7.74–7.72 (m, 2 H), 7.69 (d, *J* = 8.0 Hz, 2 H), 7.25–7.22 (m, 4 H), 7.16 (t, *J* = 7.5 Hz, 1 H), 7.02 (d, *J* = 7.5 Hz, 2 H), 4.45 (d, *J* = 8.5 Hz, 1 H), 3.62–3.53 (m, 2 H), 3.28–3.22 (m, 1 H), 2.58–2.46 (m, 2 H), 2.39 (s, 3 H), 1.75–1.59 (m, 2 H), 1.58–1.38 (m, 4 H), 1.24–1.10 (m, 2 H). **^{13}C NMR (125 MHz, CDCl$_3$)** δ 168.4, 143.2, 141.3, 138.3, 134.0, 132.1, 129.6, 128.4, 128.3, 127.0, 125.9, 123.2, 53.7, 37.2, 37.0, 34.1, 31.6, 28.1, 22.2, 21.5. **HRMS** (ESI) m/z calcd. For $C_{28}H_{30}N_2NaO_4S$ $[M+Na]^+$: 513.1818, found: 513.1829.

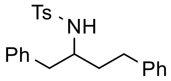

_N_-(1,4-Diphenylbutan-2-yl)-4-methylbenzenesulfonamide (3ae)

Following the **General Procedure B**, the title compound was isolated as light yellow solid (31.2 mg, 41% yield). **^{1}H NMR (500 MHz, CDCl$_3$)** δ 7.67 (d, *J* = 7.5 Hz, 2 H), 7.17–7.11 (m, 8 H), 6.96–6.95 (m, 4 H), 5.39 (d, *J* = 8.0 Hz, 1 H), 3.48–3.45 (m, 1 H), 2.68–2.60 (m, 3 H), 2.48–2.42 (m, 1 H), 2.31 (s, 3 H), 1.77–1.72 (m, 1 H), 1.67–1.60 (m, 1 H). **^{13}C NMR (125 MHz, CDCl$_3$)** δ 142.8, 141.1, 137.6, 137.1, 129.4, 129.1, 128.2, 128.09, 128.03, 126.7, 126.2, 125.5, 54.7, 41.0, 35.7, 31.4, 21.2. **HRMS** (ESI) m/z calcd. For $C_{23}H_{25}NNaO_2S$ $[M+Na]^+$: 402.1498, found: 402.1505.

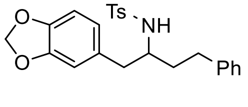

_N_-(1-(Benzo[*d*][1,3]dioxol-5-yl)-4-phenylbutan-2-yl)-4-methylbenzenesulfonamide (3af)

Following the **General Procedure B**, the title compound was isolated as light yellow solid (36.0 mg, 43% yield). **^{1}H NMR (500 MHz, CDCl$_3$)** δ 7.61 (d, *J* = 8.0 Hz, 2 H), 7.26–7.21 (m, 4 H), 7.17 (t, *J* = 7.5 Hz, 1 H), 7.05 (d, *J* = 7.0 Hz, 2 H), 6.63 (d, *J* = 7.5 Hz, 1 H), 6.43 (dd, J_1 = 7.5 Hz, J_2 = 1.5 Hz, 1 H), 6.38 (d, *J* = 1.5 Hz, 1 H), 5.90 (dd, J_1 = 5.0 Hz, J_2 = 1.5 Hz, 2 H), 4.42 (d, *J* = 8.0 Hz, 1 H), 3.42–3.35 (m, 1 H), 2.67–2.60 (m, 2 H), 2.57–2.50 (m, 2 H),

2.41 (s, 3 H), 1.83–1.76 (m, 1 H), 1.73–1.66 (m, 1 H). **^{13}C NMR (125 MHz, CDCl₃)** δ 147.6, 146.3, 143.2, 141.2, 137.5, 130.5, 129.5, 128.38, 128.33, 127.0, 125.9, 122.4, 109.5, 108.2, 100.9, 54.7, 40.7, 36.2, 31.7, 21.5. **HRMS** (ESI) m/z calcd. For $C_{24}H_{25}NNaO_4S$ [M+Na]$^+$: 446.1397, found: 446.1390.

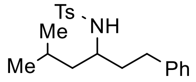

4-Methyl-*N*-(5-methyl-1-phenylhexan-3-yl)benzenesulfonamide (3ag)

Following the **General Procedure B**, the title compound was isolated as white solid (39.0 mg, 57% yield). **^{1}H NMR (500 MHz, CDCl₃)** δ 7.72 (d, *J* = 8.5 Hz, 2 H), 7.28 (d, *J* = 8.0 Hz, 2 H), 7.24 (t, *J* = 7.5 Hz, 2 H), 7.17 (t, *J* = 7.5 Hz, 1 H), 7.02 (d, *J* = 7.5 Hz, 2 H), 4.31 (d, *J* = 8.5 Hz, 1 H), 3.33–3.26 (m, 1 H), 2.58–2.47 (m, 2 H), 2.42 (s, 3 H), 1.75–1.68 (m, 1 H), 1.63–1.49 (m, 2 H), 1.29–1.17 (m, 2 H), 0.77 (d, *J* = 6.5 Hz, 3 H), 0.69 (t, *J* = 6.5 Hz, 3 H). **^{13}C NMR (125 MHz, CDCl₃)** δ 143.2, 141.5, 138.3, 129.6, 128.38, 128.36, 127.1, 125.9, 52.0, 44.6, 37.2, 31.5, 24.5, 22.7, 22.2, 21.5. **HRMS** (ESI) m/z calcd. For $C_{20}H_{27}NNaO_2S$ [M+Na]$^+$: 368.1655, found: 368.1656.

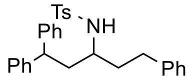

4-Methyl-*N*-(1,1,5-triphenylpentan-3-yl)benzenesulfonamide (3ah)

Following the **General Procedure B**, the title compound was isolated as white solid (75.6 mg, 81% yield). **^{1}H NMR (500 MHz, CDCl₃)** δ 7.56 (d, *J* = 8.0 Hz, 2 H), 7.25–7.14 (m, 11 H), 7.07–7.05 (m, 4 H), 6.87 (d, *J* = 7.0 Hz, 2 H), 4.47 (d, *J* = 8.5 Hz, 1 H), 3.91 (d, *J* = 7.5 Hz, 1 H), 3.17–3.10 (m, 1 H), 2.52–2.46 (m, 1 H), 2.42–2.36 (m, 4 H), 2.22–2.13 (m, 2 H), 1.82–1.75 (m, 1 H), 1.66–1.58 (m, 1 H). **^{13}C NMR (125 MHz, CDCl₃)** δ 143.9, 143.8, 143.2, 141.1, 137.8, 129.6, 128.6, 128.5, 128.4, 128.3, 127.8, 127.7, 127.2, 126.36, 126.34, 125.9, 51.9, 47.4, 41.4, 36.7, 31.3, 21.5. **HRMS** (ESI) m/z calcd. For $C_{30}H_{31}NNaO_2S$ [M+Na]$^+$: 492.1968, found: 492.1979.

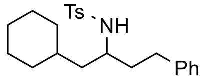

***N*-(1-Cyclohexyl-4-phenylbutan-2-yl)-4-methylbenzenesulfonamide (3ai)**

Following the **General Procedure B**, the title compound was isolated as white solid (58.9 mg, 76% yield). **^{1}H NMR (500 MHz, CDCl₃)** δ 7.71 (d, *J* = 8.0 Hz, 2 H), 7.28–7.23 (m, 4 H), 7.17 (t, *J* = 7.5 Hz, 1 H), 7.05 (d, *J* = 7.0 Hz, 2 H), 4.38 (d, *J* = 8.5 Hz, 1 H), 3.33–3.26 (m, 1 H), 2.59–2.49 (m, 2 H), 2.42 (s, 3 H), 1.76–1.46 (m, 6 H), 1.38–1.35 (m, 1 H), 1.29–1.23 (m, 1 H), 1.21–1.15 (m, 1 H), 1.13–1.02 (m, 3 H), 1.01–0.91 (m, 1 H), 0.80–0.72 (m, 1 H), 0.68–0.60 (m, 1 H). **^{13}C NMR (125 MHz, CDCl₃)** δ 143.1, 141.5, 138.2, 129.6, 128.35, 128.32 127.1, 125.8, 51.2, 43.0, 37.5, 33.8, 33.5, 32.8, 31.5, 26.4, 26.1, 25.9, 21.5. **HRMS** (ESI) m/z calcd. For $C_{23}H_{31}NNaO_2S$ [M+Na]$^+$: 408.1968, found: 408.1974.

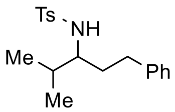

4-Methyl-*N*-(4-methyl-1-phenylpentan-3-yl)benzenesulfonamide (3aj)

Following the **General Procedure B**, the title compound was isolated as white solid (33.6 mg, 51% yield). **^{1}H NMR (500 MHz, CDCl$_3$)** δ 7.76 (d, *J* = 8.0 Hz, 2 H), 7.28 (d, *J* = 8.0 Hz, 2 H), 7.23 (t, *J* = 7.5 Hz, 2 H), 7.16 (t, *J* = 7.5 Hz, 1 H), 7.00 (d, *J* = 7.0 Hz, 2 H), 4.60 (d, *J* = 8.5 Hz, 1 H), 3.17–3.12 (m, 1 H), 2.54–2.48 (m, 1 H), 2.42–2.37 (m, 4 H), 1.80–1.66 (m, 2 H), 1.55–1.48 (m, 1 H), 0.79 (d, *J* = 6.5 Hz, 6 H). **^{13}C NMR (125 MHz, CDCl$_3$)** δ 143.1, 141.5, 138.5, 129.6, 128.3, 128.2, 127.0, 125.9, 58.9, 33.5, 32.0, 31.1, 21.5, 18.2, 17.6. **HRMS** (ESI) m/z calcd. For $C_{19}H_{25}NNaO_2S$ [M+Na]$^+$: 354.1498, found: 354.1502.

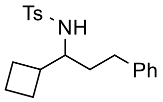

***N*-(1-Cyclobutyl-3-phenylpropyl)-4-methylbenzenesulfonamide (3ak)**

Following the **General Procedure B**, the title compound was isolated as white solid (56.4 mg, 82% yield). **^{1}H NMR (500 MHz, CDCl$_3$)** δ 7.76 (d, *J* = 8.5 Hz, 2 H), 7.28 (d, *J* = 8.0 Hz, 2 H), 7.23 (t, *J* = 7.5 Hz, 2 H), 7.16 (t, *J* = 7.5 Hz, 1 H), 7.01 (d, *J* = 7.5 Hz, 2 H), 4.41 (d, *J* = 8.5 Hz, 1 H), 3.25–3.19 (m, 1 H), 2.52–2.42 (m, 5 H), 2.37–2.28 (m, 1 H), 1.90–1.85 (m, 1 H), 1.82–1.61 (m, 5 H), 1.53–1.44 (m, 2 H). **^{13}C NMR (125 MHz, CDCl$_3$)** δ 143.2, 141.7, 138.5, 129.6, 128.3, 128.2, 127.1, 125.8, 58.5, 39.6, 34.6, 31.3, 25.4, 25.1, 21.5, 17.4. **HRMS** (ESI) m/z calcd. For $C_{20}H_{25}NNaO_2S$ [M+Na]$^+$: 366.1498, found: 366.1503.

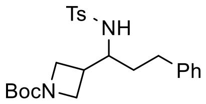

***tert*-Butyl 3-(1-((4-methylphenyl)sulfonamido)-3-phenylpropyl)azetidine-1-carboxylate (3al)**

Following the **General Procedure B**, the title compound was isolated as white solid (42.0 mg, 48% yield). **^{1}H NMR (500 MHz, CDCl$_3$)** δ 7.77 (d, *J* = 8.0 Hz, 2 H), 7.31 (d, *J* = 8.0 Hz, 2 H), 7.23 (t, *J* = 7.5 Hz, 2 H), 7.17 (t, *J* = 7.5 Hz, 1 H), 6.94 (d, *J* = 7.5 Hz, 2 H), 4.95 (s, 1 H), 3.84 (t, *J* = 8.5 Hz, 1 H), 3.79 (t, *J* = 8.5 Hz, 1 H), 3.61–3.58 (m, 2 H), 3.48–3.42 (m, 1 H), 2.70–2.61 (m, 1 H), 2.49–2.34 (m, 5 H), 1.68–1.61 (m, 1 H), 1.58–1.50 (m, 1 H), 1.41 (s, 9 H). **^{13}C NMR (125 MHz, CDCl$_3$)** δ 156.2, 143.5, 140.7, 138.3, 129.8, 128.5, 128.1, 127.0, 126.1, 79.5, 51.2, 34.3, 33.0, 31.3, 28.3, 21.5. **HRMS** (ESI) m/z calcd. For $C_{24}H_{32}N_2NaO_4S$ [M+Na]$^+$: 467.1975, found: 467.1983.

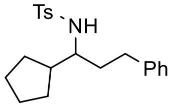

***N*-(1-Cyclopentyl-3-phenylpropyl)-4-methylbenzenesulfonamide (3am)**

Following the **General Procedure B**, the title compound was isolated as light yellow solid (28.8 mg, 40% yield). **^{1}H NMR (500 MHz, CDCl$_3$)** δ 7.75 (d, *J* = 8.0 Hz, 2 H), 7.28 (d, *J* = 8.0 Hz, 2 H), 7.23 (t, *J* = 7.5 Hz, 2 H), 7.16 (t, *J* = 7.5 Hz, 1 H), 6.99 (d, *J* = 7.0 Hz, 2 H), 4.46–4.45 (m, 1 H), 3.27–3.22 (m, 1 H), 2.53–2.46 (m, 2 H), 2.42 (s, 3 H), 1.98–1.90 (m, 1 H), 1.79–1.72 (m, 1 H), 1.65–1.44 (m, 7 H), 1.22–1.12 (m, 1 H), 1.09–1.01 (m, 1 H). **^{13}C NMR (125 MHz, CDCl$_3$)** δ 143.1, 141.7, 138.6, 129.6, 128.3, 128.2, 127.0, 125.8, 57.8, 43.8, 35.8, 31.3, 29.1, 28.8, 25.3, 25.1, 21.5. **HRMS** (ESI) m/z calcd. For $C_{21}H_{27}NNaO_2S$ [M+Na]$^+$: 380.1655, found: 380.1657.

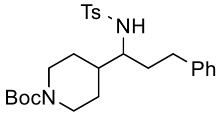

***tert*-Butyl 4-(1-((4-methylphenyl)sulfonamido)-3-phenylpropyl)piperidine-1-carboxylate (3an)**

Following the **General Procedure B**, the title compound was isolated as white solid (57.8 mg, 61% yield). **^{1}H NMR (500 MHz, CDCl$_3$)** δ 7.75 (d, *J* = 8.5 Hz, 2 H), 7.29 (d, *J* = 8.0 Hz, 2 H), 7.23 (t, *J* = 7.5 Hz, 2 H), 7.16 (t, *J* = 7.5 Hz, 1 H), 6.95 (d, *J* = 7.5 Hz, 2 H), 4.64 (d, *J* = 9.0 Hz, 1 H), 4.10 (s, 2 H), 3.21–3.16 (m, 1 H), 2.54–2.43 (m, 6 H), 2.37–2.30 (m, 1 H), 1.74–1.67 (m, 1 H), 1.58–1.48 (m, 4 H), 1.43 (s, 9 H), 1.20–1.04 (m, 2 H). **^{13}C NMR (125 MHz, CDCl$_3$)** δ 154.6, 143.4, 141.0, 138.3, 129.7, 128.4, 128.1, 127.0, 126.0, 79.4, 57.8, 43.7, 40.1, 33.4, 31.8, 28.4, 27.8, 21.5. **HRMS** (ESI) m/z calcd. For $C_{26}H_{36}N_2NaO_4S$ [M+Na]$^+$: 495.2288, found: 495.2300.

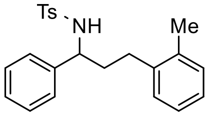

4-Methyl-*N*-(1-phenyl-3-(*o*-tolyl)propyl)benzenesulfonamide (3ao)

Following the **General Procedure A**, the title compound was isolated as white solid (61.2 mg, 81% yield). **^{1}H NMR (500 MHz, CDCl$_3$)** δ 7.54 (d, *J* = 8.0 Hz, 2 H), 7.16–7.15 (m, 3 H), 7.10–7.03 (m, 7 H), 6.98–6.96 (m, 1 H), 5.27 (d, *J* = 7.5 Hz, 1 H), 4.33 (dd, J_1 = 15.0 Hz, J_2 = 7.5 Hz, 1 H), 2.58–2.51 (m, 1 H), 2.42–2.36 (m, 1 H), 2.34 (s, 3 H), 2.11 (s, 3 H), 2.07–2.00 (m, 1 H), 1.98–1.89 (m, 1 H). **^{13}C NMR (125 MHz, CDCl$_3$)** δ 142.9, 140.6, 139.1, 137.6, 135.8, 130.2, 129.3, 128.6, 128.5, 127.4, 127.0, 126.5, 126.1, 125.9, 58.2, 37.6, 29.5, 21.4, 19.1. **HRMS** (ESI) m/z calcd. For $C_{23}H_{25}NNaO_2S$ [M+Na]$^+$: 402.1498, found: 402.1498.

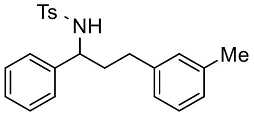

4-Methyl-*N*-(1-phenyl-3-(*m*-tolyl)propyl)benzenesulfonamide (3ap)

Following the **General Procedure A**, the title compound was isolated as light yellow oil (71.0 mg, 94% yield). **^{1}H NMR (500 MHz, CDCl$_3$)** δ 7.52 (d, *J* = 8.5 Hz, 2 H), 7.15–7.07 (m, 6 H), 7.02–6.97 (m, 3 H), 6.85–6.82 (m, 2 H), 5.29 (d, *J* = 7.5 Hz, 1 H), 4.28 (dd, J_1 = 15.0 Hz, J_2 = 7.5 Hz, 1 H), 2.54–2.48 (m, 1 H), 2.46–2.40 (m, 1 H), 2.34 (s, 3 H), 2.28 (s, 3 H), 2.13–2.05 (m, 1 H), 2.02–1.94 (m, 1 H). **^{13}C NMR (125 MHz, CDCl$_3$)** δ 142.9, 140.8, 140.6,

137.9, 137.6, 129.2, 129.1, 128.4, 128.2, 127.4, 127.0, 126.7, 126.5, 125.3, 57.9, 39.0, 32.0, 21.4, 21.3. **HRMS** (ESI) m/z calcd. For $C_{23}H_{25}NNaO_2S$ $[M+Na]^+$: 402.1498, found: 402.1497.

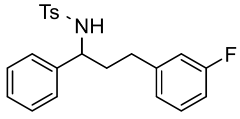

***N*-(3-(3-Fluorophenyl)-1-phenylpropyl)-4-methylbenzenesulfonamide (3aq)**

Following the **General Procedure A**, the title compound was isolated as white solid (62.8 mg, 82% yield). **^{1}H NMR (500 MHz, $CDCl_3$)** δ 7.53 (d, *J* = 8.0 Hz, 2 H), 7.21–7.15 (m, 4 H), 7.10 (d, *J* = 8.5 Hz, 2 H), 7.01–6.99 (m, 2 H), 6.87–6.81 (m, 2 H), 6.71 (d, *J* = 10.0 Hz, 1 H), 5.35–5.29 (m, 1 H), 4.25 (dd, J_1 = 15.0 Hz, J_2 = 7.5 Hz, 1 H), 2.58–2.45 (m, 2 H), 2.35 (s, 3 H), 2.13–2.06 (m, 1 H), 2.03–1.95 (m, 1 H). **^{13}C NMR (125 MHz, $CDCl_3$)** δ 162.8 (d, *J* = 243.9 Hz), 143.4 (d, *J* = 7.0 Hz), 143.1, 140.4, 137.4, 129.8 (d, *J* = 8.4 Hz), 129.3, 128.5, 127.5, 127.0, 126.5, 124.0 (d, *J* = 2.8 Hz), 115.2 (d, *J* = 20.9 Hz), 112.9 (d, *J* = 20.9 Hz), 57.7, 38.6, 31.8 (d, *J* = 1.5 Hz), 21.4. **^{19}F NMR (470 MHz, $CDCl_3$)** δ -113.6. **HRMS** (ESI) m/z calcd. For $C_{22}H_{22}FNNaO_2S$ $[M+Na]^+$: 406.1247, found: 406.1245.

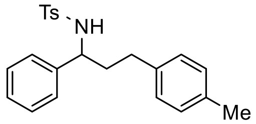

4-Methyl-*N*-(1-phenyl-3-(*p*-tolyl)propyl)benzenesulfonamide (3ar)

Following the **General Procedure A**, the title compound was isolated as white solid (70.2 mg, 93% yield). **^{1}H NMR (500 MHz, $CDCl_3$)** δ 7.51 (d, *J* = 8.5 Hz, 2 H), 7.15–7.13 (m, 3 H), 7.08 (d, *J* = 8.0 Hz, 2 H), 7.04 (d, *J* = 8.0 Hz, 2 H), 7.01–6.99 (m, 2 H), 6.92 (d, *J* = 8.0 Hz, 2 H), 5.28 (d, *J* = 7.5 Hz, 1 H), 4.27 (dd, J_1 = 15.0 Hz, J_2 = 7.5 Hz, 1 H), 2.53–2.40 (m, 2 H), 2.34 (s, 3 H), 2.30 (s, 3 H), 2.12–2.04 (m, 1 H), 2.00–1.93 (m, 1 H). **^{13}C NMR (125 MHz, $CDCl_3$)** δ 142.9, 140.6, 137.7, 137.6, 135.4, 129.2, 129.0, 128.4, 128.2, 127.3, 127.0, 126.5, 57.8, 39.1, 31.6, 21.4, 21.0. **HRMS** (ESI) m/z calcd. For $C_{23}H_{25}NNaO_2S$ $[M+Na]^+$: 402.1498, found: 402.1498.

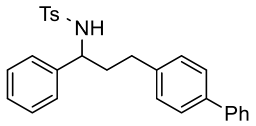

***N*-(3-([1,1'-Biphenyl]-4-yl)-1-phenylpropyl)-4-methylbenzenesulfonamide (3as)**

Following the **General Procedure A**, the title compound was isolated as white solid (71.5 mg, 81% yield). **^{1}H NMR (500 MHz, $CDCl_3$)** δ 7.58–7.56 (m, 2 H), 7.52 (d, *J* = 8.0 Hz, 2 H), 7.48 (d, *J* = 8.5 Hz, 2 H), 7.43 (t, *J* = 7.5 Hz, 2 H), 7.33 (t, *J* = 7.5 Hz, 1 H), 7.18–7.17 (m, 3 H), 7.13–7.08 (m, 4 H), 7.03–7.01 (m, 2 H), 4.99 (d, *J* = 7.5 Hz, 1 H), 4.30 (dd, J_1 = 14.5 Hz, J_2 = 6.0 Hz, 1 H), 2.62–2.51 (m, 2 H), 2.34 (s, 3 H), 2.21–2.13 (m, 1 H), 2.09–2.01 (m, 1 H). **^{13}C NMR (125 MHz, $CDCl_3$)** δ 143.0, 140.9, 140.5, 139.9, 139.0, 137.5, 129.3, 128.8, 128.7, 128.6, 127.5, 127.12, 127.07, 127.06, 126.95, 126.5, 57.9, 38.9, 31.7, 21.4. **HRMS** (ESI) m/z calcd. For $C_{28}H_{27}NNaO_2S$ $[M+Na]^+$: 464.1655, found: 464.1659.

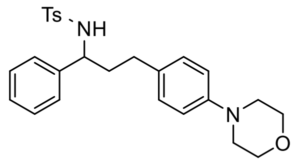

4-Methyl-*N*-(3-(4-morpholinophenyl)-1-phenylpropyl)benzenesulfonamide (3at)

Following the **General Procedure A**, the title compound was isolated as white solid (52.5 mg, 58% yield). **^{1}H NMR (500 MHz, CDCl$_3$)** δ 7.50 (d, J = 8.0 Hz, 2 H), 7.15–7.14 (m, 3 H), 7.08 (d, J = 8.0 Hz, 2 H), 7.00–6.96 (m, 4 H), 6.81 (d, J = 8.5 Hz, 2 H), 5.11 (d, J = 7.5 Hz, 1 H), 4.28 (dd, J_1 = 15.0 Hz, J_2 = 7.5 Hz, 1 H), 3.86 (t, J = 4.5 Hz, 4 H), 3.11 (t, J = 4.5 Hz, 4 H), 2.51–2.39 (m, 2 H), 2.34 (s, 3 H), 2.12–2.04 (m, 1 H), 2.00–1.92 (m, 1 H). **^{13}C NMR (125 MHz, CDCl$_3$)** δ 149.5, 142.9, 140.6, 137.6, 132.4, 129.2, 129.1, 128.4, 127.4, 127.0, 126.5, 115.9, 66.9, 57.8, 49.6, 39.1, 31.1, 21.4. **HRMS** (ESI) m/z calcd. For $C_{26}H_{30}N_2NaO_3S$ [M+Na]$^+$: 473.1869, found: 473.1870.

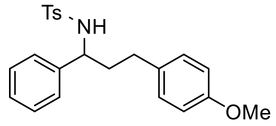

***N*-(3-(4-Methoxyphenyl)-1-phenylpropyl)-4-methylbenzenesulfonamide (3au)**

Following the **General Procedure A**, the title compound was isolated as light yellow oil (68.3 mg, 86% yield). **^{1}H NMR (500 MHz, CDCl$_3$)** δ 7.51 (d, J = 8.0 Hz, 2 H), 7.15–7.14 (m, 3 H), 7.08 (d, J = 8.0 Hz, 2 H), 7.00–6.98 (m, 2 H), 6.96 (d, J = 8.5 Hz, 2 H), 6.78 (d, J = 8.5 Hz, 2 H), 5.14 (d, J = 7.5 Hz, 1 H), 4.26 (dd, J_1 = 15.0 Hz, J_2 = 7.5 Hz, 1 H), 3.78 (s, 3 H), 2.51–2.40 (m, 2 H), 2.34 (s, 3 H), 2.11–2.04 (m, 1 H), 2.00–1.93 (m, 1 H). **^{13}C NMR (125 MHz, CDCl$_3$)** δ 157.8, 142.9, 140.6, 137.5, 132.8, 129.3, 128.5, 127.4, 127.0, 126.5, 113.8, 57.8, 55.2, 39.2, 31.1, 21.4. **HRMS** (ESI) m/z calcd. For $C_{23}H_{25}NNaO_3S$ [M+Na]$^+$: 418.1447, found: 418.1453.

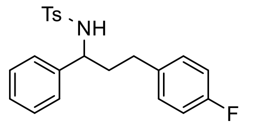

***N*-(3-(4-Fluorophenyl)-1-phenylpropyl)-4-methylbenzenesulfonamide (3av)**

Following the **General Procedure A**, the title compound was isolated as colorless oil (70.1 mg, 92% yield). **^{1}H NMR (500 MHz, CDCl$_3$)** δ 7.51 (d, J = 8.0 Hz, 2 H), 7.16–7.14 (m, 3 H), 7.09 (d, J = 8.0 Hz, 2 H), 7.01–6.97 (m, 4 H), 6.93–6.90 (m, 2 H), 5.20 (d, J = 8.0 Hz, 1 H), 4.24 (dd, J_1 = 15.0 Hz, J_2 = 7.5 Hz, 1 H), 2.56–2.45 (m, 2 H), 2.35 (s, 3 H), 2.13–2.06 (m, 1 H), 2.01–1.94 (m, 1 H). **^{13}C NMR (125 MHz, CDCl$_3$)** δ 161.3 (d, J = 242.3 Hz), 143.0, 140.4, 137.4, 136.4 (d, J = 3.1 Hz), 129.7 (d, J = 7.8 Hz), 129.3, 128.5, 127.5, 127.0, 126.5, 115.1 (d, J = 21.0 Hz), 57.7, 39.1, 31.2, 21.4. **^{19}F NMR (470 MHz, CDCl$_3$)** δ -117.4. **HRMS** (ESI) m/z calcd. For $C_{22}H_{22}FNNaO_2S$ [M+Na]$^+$: 406.1247, found: 406.1247.

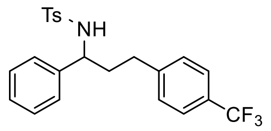

4-Methyl-*N*-(1-phenyl-3-(4-(trifluoromethyl)phenyl)propyl)benzenesulfonamide (3aw)

Following the **General Procedure A**, the title compound was isolated as colorless oil (63.0 mg, 73% yield). Known compound.[31] **^{1}H NMR (500 MHz, CDCl$_3$)** δ 7.51–7.48 (m, 4 H), 7.17–7.16 (m, 5 H), 7.09 (d, J = 8.5 Hz, 2 H), 6.99–6.96 (m, 2 H), 5.14 (d, J = 7.5 Hz, 1 H), 4.23 (dd, J_1 = 14.5 Hz, J_2 = 7.5 Hz, 1 H), 2.66–2.55 (m, 2 H), 2.35 (s, 3 H), 2.19–2.12 (m, 1 H), 2.06–1.99 (m, 1 H). **^{13}C NMR (125 MHz, CDCl$_3$)** δ 145.0, 143.1, 140.2, 137.3, 129.3, 128.8, 128.7, 128.4 (q, J = 32.1 Hz), 127.7, 127.0, 126.4, 125.3 (q, J = 3.8 Hz), 124.3 (q, J = 270.1 Hz), 57.7, 38.6, 31.9, 21.4. **^{19}F NMR (470 MHz, CDCl$_3$)** δ -62.3. **HRMS** (ESI) m/z calcd. For $C_{23}H_{22}F_3N_2O_2SNa$ [M+Na]$^+$: 456.1216, found: 456.1202.

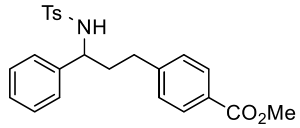

Methyl 4-(3-((4-methylphenyl)sulfonamido)-3-phenylpropyl)benzoate (3ax)

Following the **General Procedure A**, the title compound was isolated as colorless oil (45.3 mg, 54% yield). **^{1}H NMR (500 MHz, CDCl$_3$)** δ 7.91 (d, J = 8.0 Hz, 2 H), 7.51 (d, J = 8.0 Hz, 2 H), 7.16–7.08 (m, 7 H), 6.99 (d, J = 3.0 Hz, 2 H), 5.23 (s, 1 H), 4.25–4.24 (m, 1 H), 3.90 (s, 3 H), 2.65–2.53 (m, 2 H), 2.35 (s, 3 H), 2.14–2.12 (m, 1 H), 2.04–2.02 (m, 1 H). **^{13}C NMR (125 MHz, CDCl$_3$)** δ 167.0, 146.4, 143.1, 140.3, 137.4, 129.7, 129.3, 128.6, 128.4, 128.0, 127.6, 127.0, 126.5, 57.7, 52.0, 38.6, 32.1, 21.4. **HRMS** (ESI) m/z calcd. For $C_{24}H_{25}NNaO_4S$ [M+Na]$^+$: 446.1397, found: 446.1399.

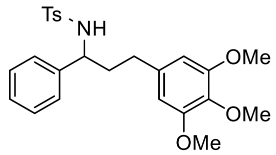

4-Methyl-*N*-(1-phenyl-3-(3,4,5-trimethoxyphenyl)propyl)benzenesulfonamide (3ay)

Following the **General Procedure A**, the title compound was isolated as light yellow oil (62.7 mg, 69% yield). **^{1}H NMR (500 MHz, CDCl$_3$)** δ 7.49 (d, J = 8.0 Hz, 2 H), 7.14–7.13 (m, 3 H), 7.07 (d, J = 7.5 Hz, 2 H), 6.98–6.97 (m, 2 H), 6.32 (s, 2 H), 5.32 (d, J = 5.5 Hz, 1 H), 4.30–4.29 (m, 1 H), 3.82 (s, 3 H), 3.81 (s, 6 H), 2.57–2.48 (m, 2 H), 2.33 (s, 3 H), 2.16–2.11 (m, 1 H), 2.04–1.96 (m, 1 H). **^{13}C NMR (125 MHz, CDCl$_3$)** δ 153.1, 143.0, 140.5, 137.4, 136.6, 136.1, 129.2, 128.4, 127.4, 127.0, 126.5, 105.3, 60.8, 57.7, 56.0, 39.1, 32.4, 21.4. **HRMS** (ESI) m/z calcd. For $C_{25}H_{29}NNaO_5S$ [M+Na]$^+$: 478.1659, found: 478.1660.

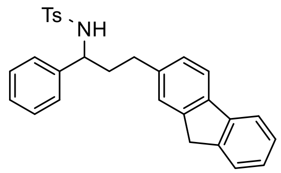

***N*-(3-(9*H*-Fluoren-2-yl)-1-phenylpropyl)-4-methylbenzenesulfonamide (3az)**

Following the **General Procedure A**, the title compound was isolated as yellow solid (69.7 mg, 77% yield). **^{1}H NMR (500 MHz, CDCl$_3$)** δ 7.74 (d, *J* = 7.5 Hz, 1 H), 7.65 (d, *J* = 8.0 Hz, 1 H), 7.53–7.49 (m, 3 H), 7.36 (d, *J* = 7.5 Hz, 1 H), 7.29–7.27 (m, 1 H), 7.24 (s, 1 H), 7.17–7.15 (m, 3 H), 7.07–7.05 (m, 3 H), 7.02–7.00 (m, 2 H), 4.97 (d, *J* = 7.5 Hz, 1 H), 4.30 (dd, J_1 = 14.5 Hz, J_2 = 7.5 Hz, 1 H), 3.83 (s, 2 H), 2.65–2.54 (m, 2 H), 2.32 (s, 3 H), 2.21–2.14 (m, 1 H), 2.10–2.02 (m, 1 H). **^{13}C NMR (125 MHz, CDCl$_3$)** δ 143.5, 143.1, 143.0, 141.6, 140.6, 139.7, 139.6, 137.5, 129.3, 128.5, 127.5, 127.1, 127.0, 126.7, 126.5, 126.4, 125.1, 125.0, 119.7, 119.6, 57.9, 39.2, 36.8, 32.2, 21.4. **HRMS** (ESI) m/z calcd. For $C_{29}H_{27}NNaO_2S$ $[M+Na]^+$: 476.1655, found: 476.1667.

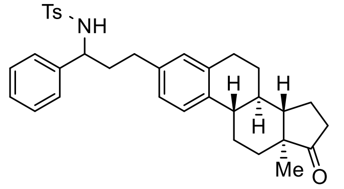

4-Methyl-*N*-(3-((8*R*,9*S*,13*S*,14*S*)-13-methyl-17-oxo-7,8,9,11,12,13,14,15,16,17-decahydro-6*H*-cyclopenta[*a*]phenanthren-3-yl)-1-phenylpropyl)benzenesulfonamide (3ba)

Following the **General Procedure A**, but Ni(cod)$_2$ (0.01 mmol, 2.8 mg, 5 mol%), PCy$_3$ (0.02 mmol, 5.6 mg, 10 mol%), PhB(OH)$_2$ (0.2 mmol, 24.4 mg, 100 mol%) and KO^{t}Bu (0.1 mmol, 11.2 mg, 50 mol%) were used. The title compound was isolated as colorless oil (92.2 mg, 85% yield). **^{1}H NMR (500 MHz, CDCl$_3$)** δ 7.52 (d, *J* = 8.0 Hz, 2 H), 7.16–7.15 (m, 4 H), 7.08 (d, *J* = 7.5 Hz, 2 H), 7.01 (m, 2 H), 6.84–6.79 (m, 2 H), 5.28 (d, *J* = 7.0 Hz, 1 H), 4.31 (dd, J_1 = 13.5 Hz, J_2 = 6.5 Hz, 1 H), 2.85–2.83 (m, 2 H), 2.53–2.47 (m, 2 H), 2.44–2.38 (m, 2 H), 2.34 (s, 3 H), 2.30–2.21 (m, 1 H), 2.18–1.94 (m, 6 H), 0.90 (s, 3 H). **^{13}C NMR (125 MHz, CDCl$_3$)** δ 221.0, 142.8, 140.6, 138.3, 137.6, 137.4, 136.4, 129.2, 128.9, 128.4, 127.3, 127.0, 126.5, 125.7, 125.3, 58.0, 50.4, 48.0, 44.2, 39.0, 38.1, 35.8, 31.55, 31.51, 29.29, 29.28, 26.5, 25.7, 21.5, 21.4, 13.8. **HRMS** (ESI) m/z calcd. For $C_{34}H_{39}NNaO_3S$ $[M+Na]^+$: 564.2543, found: 564.2569.

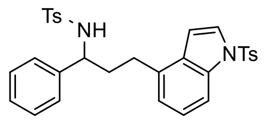

4-Methyl-*N*-(1-phenyl-3-(1-tosyl-1*H*-indol-4-yl)propyl)benzenesulfonamide (3bb)

Following the **General Procedure A**, the title compound was isolated as yellow solid (48.8 mg, 44% yield). **^{1}H NMR (500 MHz, CDCl$_3$)** δ 7.81 (d, *J* = 8.5 Hz, 1 H), 7.74 (d, *J* = 8.0 Hz, 2 H), 7.49 (d, *J* = 3.5 Hz, 1 H), 7.46 (d, *J* = 8.0 Hz, 2 H), 7.20–7.12 (m, 6 H), 7.05 (d, *J* = 8.0 Hz, 2 H), 6.99–6.98 (m, 2 H), 6.89 (d, *J* = 7.5 Hz, 1 H), 6.43 (d,

J = 3.5 Hz, 1 H), 5.17 (d, J = 7.5 Hz, 1 H), 4.27 (dd, J_1 = 14.5 Hz, J_2 = 7.5 Hz, 1 H), 2.75–2.69 (m, 1 H), 2.66–2.60 (m, 1 H), 2.33 (s, 3 H), 2.31 (s, 3 H), 2.16–2.09 (m, 1 H), 2.05–1.98 (m, 1 H). **^{13}C NMR (125 MHz, CDCl$_3$)** δ 144.9, 143.0, 140.3, 137.4, 135.2, 134.7, 133.8, 129.8, 129.7, 129.3, 128.5, 127.5, 126.9, 126.8, 126.5, 125.8, 124.6, 122.6, 111.4, 106.9, 58.0, 37.9, 29.2, 21.5, 21.4. **HRMS** (ESI) m/z calcd. For $C_{31}H_{30}N_2NaO_4S_2$ [M+Na]$^+$: 581.1539, found: 581.1546.

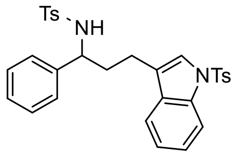

4-Methyl-*N*-(1-phenyl-3-(1-tosyl-1*H*-indol-3-yl)propyl)benzenesulfonamide (3bc)

Following the **General Procedure A**, the title compound was isolated as brown solid (88.8 mg, 80% yield). **^{1}H NMR (500 MHz, CDCl$_3$)** δ 7.96 (d, J = 8.0 Hz, 1 H), 7.73 (d, J = 8.0 Hz, 2 H), 7.50 (d, J = 8.0 Hz, 2 H), 7.29–7.23 (m, 3 H), 7.19–7.15 (m, 6 H), 7.04 (d, J = 8.0 Hz, 2 H), 6.99–6.98 (m, 2 H), 5.37 (d, J = 7.5 Hz, 1 H), 4.29 (dd, J_1 = 15.0 Hz, J_2 = 7.5 Hz, 1 H), 2.63–2.49 (m, 2 H), 2.32 (s, 3 H), 2.30 (s, 3 H), 2.19–2.12 (m, 1 H), 2.06–1.99 (m, 1 H). **^{13}C NMR (125 MHz, CDCl$_3$)** δ 144.7, 143.1, 140.4, 137.4, 135.25, 135.18, 130.6, 129.8, 129.3, 128.6, 127.5, 126.9, 126.7, 126.4, 124.6, 122.9, 122.8, 121.8, 119.3, 113.7, 57.8, 36.3, 21.5, 21.4, 21.3. **HRMS** (ESI) m/z calcd. For $C_{31}H_{30}N_2NaO_4S_2$ [M+Na]$^+$: 581.1539, found: 581.1544.

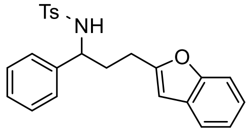

***N*-(3-(Benzofuran-2-yl)-1-phenylpropyl)-4-methylbenzenesulfonamide (3bd)**

Following the **General Procedure A**, the title compound was isolated as light yellow solid (53.0 mg, 65% yield). **^{1}H NMR (500 MHz, CDCl$_3$)** δ 7.52 (d, J = 8.5 Hz, 2 H), 7.47–7.46 (m, 1 H), 7.37 (d, J = 8.0 Hz, 1 H), 7.23–7.17 (m, 5 H), 7.06–7.02 (m, 4 H), 6.32 (s, 1 H), 5.07 (d, J = 7.5 Hz, 1 H), 4.33 (dd, J_1 = 14.5 Hz, J_2 = 7.5 Hz, 1 H), 2.75–2.65 (m, 2 H), 2.33 (s, 3 H), 2.29–2.22 (m, 1 H), 2.17–2.10 (m, 1 H). **^{13}C NMR (125 MHz, CDCl$_3$)** δ 157.6, 154.6, 143.1, 140.2, 137.3, 129.3, 128.7, 128.6, 127.7, 127.0, 126.5, 123.3, 122.5, 120.3, 110.7, 102.6, 57.7, 35.2, 25.0, 21.4. **HRMS** (ESI) m/z calcd. For $C_{24}H_{23}NNaO_3S$ [M+Na]$^+$: 428.1291, found: 428.1295.

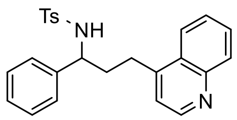

4-Methyl-*N*-(1-phenyl-3-(quinolin-4-yl)propyl)benzenesulfonamide (3be)

Following the **General Procedure A**, but Ni(cod)$_2$ (0.01 mmol, 2.8 mg, 5 mol%), PCy$_3$ (0.02 mmol, 5.6 mg, 10 mol%), PhB(OH)$_2$ (0.2 mmol, 24.4 mg, 100 mol%) and KO^{t}Bu (0.1 mmol, 11.2 mg, 50 mol%) were used. The title compound was isolated as white solid (43.6 mg, 52% yield). **^{1}H NMR (500 MHz, CDCl$_3$)** δ 8.74 (d, J = 4.5 Hz, 1

H), 8.08 (d, J = 8.5 Hz, 1 H), 7.76 (d, J = 8.0 Hz, 1 H), 7.67–7.64 (m, 1 H), 7.54 (d, J = 8.0 Hz, 2 H), 7.47–7.44 (m, 1 H), 7.18–7.16 (m, 3 H), 7.11 (d, J = 4.5 Hz, 1 H), 7.06–7.03 (m, 4 H), 5.64 (d, J = 8.0 Hz, 1 H), 4.40 (dd, J_1 = 15.0 Hz, J_2 = 7.0 Hz, 1 H), 3.13-3.07 (m, 1 H), 2.95–2.89 (m, 1 H), 2.32 (s, 3 H), 2.28–2.21 (m, 1 H), 2.16–2.09 (m, 1 H). **^{13}C NMR (125 MHz, $CDCl_3$)** δ 150.1, 148.2, 146.9, 143.1, 140.2, 137.4, 130.1, 129.3, 129.1, 128.7, 127.7, 127.2, 127.0, 126.44, 126.40, 123.3, 120.6, 58.1, 37.4, 28.4, 21.4. **HRMS** (ESI) m/z calcd. For $C_{25}H_{24}N_2NaO_2S$ $[M+Na]^+$: 439.1451, found: 439.1467.

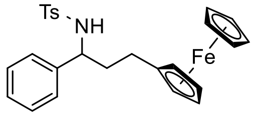

4-Methyl-*N*-(1-phenyl-3-(ferrocenyl)propyl)benzenesulfonamide (3bf)

Following the **General Procedure A**, the title compound was isolated as brown solid (86.2 mg, 91% yield). **^{1}H NMR (500 MHz, $CDCl_3$)** δ 7.54 (d, J = 8.0 Hz, 2 H), 7.16–7.14 (m, 3 H), 7.11 (d, J = 8.5 Hz, 2 H), 7.03–7.01 (m, 2 H), 5.18 (d, J = 7.5 Hz, 1 H), 4.29 (dd, J_1 = 14.5 Hz, J_2 = 7.0 Hz, 1 H), 4.01 (s, 7 H), 3.97 (s, 1 H), 3.93 (s, 1 H), 2.35 (s, 3 H), 2.30–2.24 (m, 1 H), 2.19–2.13 (m, 1 H), 2.02–1.95 (m, 1 H), 1.93–1.85 (m, 1 H). **^{13}C NMR (125 MHz, $CDCl_3$)** δ 142.9, 140.7, 137.6, 129.3, 128.5, 127.4, 127.0, 126.5, 87.7, 68.4, 67.88, 67.87, 67.24, 67.18, 58.1, 38.6, 25.8, 21.4. **HRMS** (ESI) m/z calcd. For $C_{26}H_{27}FeNNaO_2S$ $[M+Na]^+$: 496.1004, found: 496.1013.

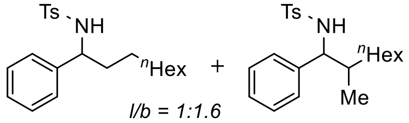

Mixture of 4-methyl-*N*-(1-phenylnonyl)benzenesulfonamide (3bg-L) and 4-methyl-*N*-(2-methyl-1-phenyloctyl)benzenesulfonamide (3bg-B)

Following the **General Procedure A**, but $Ni(cod)_2$ (0.01 mmol, 2.8 mg, 5 mol%), PCy_3 (0.02 mmol, 5.6 mg, 10 mol%) were used and pivaldehyde (0.4 mmol, 44 μL, 2.0 equiv.) was added as an additive. The mixture of linear and branched products were isolated as white solid (65.9 mg, 88% yield, *l*/*b* = 1:1.6 ratio was determined by ^{1}H NMR spectroscopy). **^{1}H NMR (500 MHz, $CDCl_3$)** δ 7.55–7.52 (m, 5 H), 7.16–7.05 (m, 13.4 H), 7.02–6.96 (m, 5 H), 5.41 (d, J = 8.5 Hz, 1.6 H), 5.30 (d, J = 7.5 Hz, 1 H), 4.27–4.24 (m, 1 H), 4.21 (dd, J_l = 8.5 Hz, J_2 = 6.0 Hz, 1.6 H), 2.34 (s, 3 H), 2.32 (s, 4.8 H), 1.78–1.71 (m, 2.6 H), 1.27–0.95 (m, 29.3 H), 0.87–0.83 (m, 12.3 H). **^{13}C NMR (125 MHz, $CDCl_3$)** δ 142.8, 142.7, 141.1, 140.3, 137.72, 137.66, 129.2, 129.1, 128.3, 128.0, 127.1, 127.0, 126.8, 126.7, 126.6, 62.2, 58.3, 39.5, 37.6, 33.2, 31.8, 31.7, 29.3, 29.12, 29.06, 26.9, 25.8, 22.58, 22.56, 21.37, 21.34, 14.9, 14.05, 14.02. **HRMS** (ESI) m/z calcd. For $C_{22}H_{31}NNaO_2S$ $[M+Na]^+$: 396.1968, found: 396.1968.

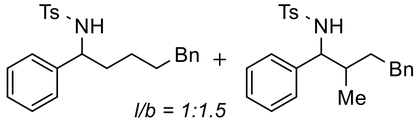

Mixture of *N*-(1,5-diphenylpentyl)-4-methylbenzenesulfonamide (3bh-L) and 4-methyl-*N*-(2-methyl-1,4-diphenylbutyl)benzenesulfonamide (3bh-B)

Following the **General Procedure A**, but Ni(cod)$_2$ (0.01 mmol, 2.8 mg, 5 mol%), PCy$_3$ (0.02 mmol, 5.6 mg, 10 mol%) were used and pivaldehyde (0.4 mmol, 44 μL, 2.0 equiv.) was added as an additive. The mixture of linear and branched products were isolated as white solid (68.7 mg, 87% yield, *l*/*b* = 1:1.5 ratio was determined by ^{1}H NMR spectroscopy). **^{1}H NMR (500 MHz, CDCl$_3$)** δ 7.55–7.51 (m, 5 H), 7.25–7.20 (m, 4 H), 7.16–7.12 (m, 6 H), 7.08–7.07 (m, 9 H), 7.03–6.99 (m, 8 H), 6.92–6.90 (m, 3 H), 5.49 (d, *J* = 9.0 Hz, 1.5 H), 5.36 (d, *J* = 7.5 Hz, 1 H), 4.29–4.22 (m, 2.5 H), 2.61–2.55 (m, 1.5 H), 2.49–2.45 (m, 3 H), 2.33 (s, 3 H), 2.29 (s, 4.5 H), 1.82–1.75 (m, 2.5 H), 1.72–1.67 (m, 1 H), 1.61–1.55 (m, 1.5 H), 1.52–1.49 (m, 2 H), 1.36–1.26 (m, 3 H), 1.20–1.12 (m, 1 H), 0.92 (d, *J* = 6.5 Hz, 4.5 H). **^{13}C NMR (125 MHz, CDCl$_3$)** δ 142.83, 142.77, 142.3, 141.9, 141.0, 139.9, 137.7, 137.6, 129.2, 129.1, 128.33, 128.26, 128.21, 128.20, 128.0, 127.2, 127.00, 126.97, 126.79, 126.75, 126.4, 125.7, 125.6, 62.0, 58.2, 38.9, 37.4, 35.5, 34.9, 33.0, 30.8, 25.5, 21.4, 21.3, 15.0. **HRMS** (ESI) m/z calcd. For $C_{24}H_{27}NNaO_2S$ [M+Na]$^+$: 416.1655, found: 416.1653.

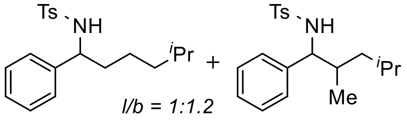

Mixture of 4-methyl-*N*-(5-methyl-1-phenylhexyl)benzenesulfonamide (3bi-L) and *N*-(2,4-dimethyl-1-phenylpentyl)-4-methylbenzenesulfonamide (3bi-B)

Following the **General Procedure A**, but Ni(cod)$_2$ (0.01 mmol, 2.8 mg, 5 mol%), PCy$_3$ (0.02 mmol, 5.6 mg, 10 mol%) were used and pivaldehyde (0.4 mmol, 44 μL, 2.0 equiv.) was added as an additive. The mixture of linear and branched products were isolated as white solid (28.4 mg, 41% yield, *l*/*b* = 1:1.2 ratio was determined by ^{1}H NMR spectroscopy). **^{1}H NMR (500 MHz, CDCl$_3$)** δ 7.55–7.52 (m, 4.4 H), 7.14–7.10 (m, 9 H), 7.06 (d, *J* = 8.5 Hz, 2.4 H), 7.03–7.01 (m, 2 H), 6.97–6.95 (m, 2 H), 5.24 (d, *J* = 8.5 Hz, 1.2 H), 5.15 (d, *J* = 7.5 Hz, 1 H), 4.25 (dd, J_1 = 14.5 Hz, J_2 = 7.0 Hz, 1 H), 4.20 (dd, J_1 = 8.5 Hz, J_2 = 6.0 Hz, 1.2 H), 2.35 (s, 3 H), 2.32 (s, 3.6 H), 1.85–1.80 (m, 1.2 H), 1.75–1.69 (m, 1 H), 1.67–1.61 (m, 2 H), 1.56–1.48 (m, 1.2 H), 1.43–1.36 (m, 1 H), 1.23–1.18 (m, 1 H), 1.08–1.05 (m, 3 H), 0.99–0.94 (m, 2 H), 0.82 (d, *J* = 7.0 Hz, 3.6 H), 0.79 (d, *J* = 6.5 Hz, 3.6 H), 0.78 (d, *J* = 2.5 Hz, 3 H), 0.76 (d, *J* = 2.5 Hz, 3 H), 0.70 (d, *J* = 6.5 Hz, 3.6 H). **^{13}C NMR (125 MHz, CDCl$_3$)** δ 142.85, 142.77, 141.1, 140.3, 137.7, 137.6, 129.2, 129.1, 128.3, 128.0, 127.2, 127.0, 126.8, 126.7, 126.6, 126.4, 62.3, 58.3, 42.6, 38.3, 37.9, 37.1, 27.7, 24.9, 23.6, 23.3, 22.5, 22.4, 21.6, 21.39, 21.35, 14.8. **HRMS** (ESI) m/z calcd. For $C_{20}H_{27}NNaO_2S$ [M+Na]$^+$: 368.1655, found: 368.1660.

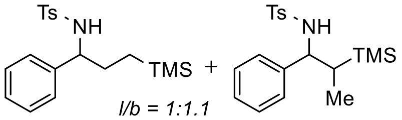

Mixture of 4-methyl-*N*-(1-phenyl-3-(trimethylsilyl)propyl)benzenesulfonamide (3bj-L) and 4-methyl-*N*-(1-phenyl-2-(trimethylsilyl)propyl)benzenesulfonamide (3bj-B)

Following the **General Procedure A**, but $Ni(cod)_2$ (0.01 mmol, 2.8 mg, 5 mol%), PCy_3 (0.02 mmol, 5.6 mg, 10 mol%) were used and pivaldehyde (0.4 mmol, 44 μL, 2.0 equiv.) was added as an additive. The mixture of linear and branched products were isolated as colorless oil (66.5 mg, 92% yield, *l*/*b* = 1:1.1 ratio was determined by ^{1}H NMR spectroscopy). **^{1}H NMR (500 MHz, $CDCl_3$)** δ 7.55 (d, *J* = 8.0 Hz, 2 H), 7.43 (d, *J* = 8.0 Hz, 1 H), 7.39 (d, *J* = 8.0 Hz, 1.2 H), 7.14–7.12 (m, 3 H), 7.09 (d, *J* = 8.3 Hz, 2 H), 7.03–6.95 (m, 7.5 H), 6.92–6.87 (m, 2.2 H), 5.37–5.34 (m, 1.5 H), 5.25 (d, *J* = 8.5 Hz, 0.6 H), 4.36–4.33 (m, 0.5 H), 4.30–4.27 (m, 0.6 H), 4.18 (dd, J_1 = 14.5 Hz, J_2 = 7.0 Hz, 1 H), 2.34 (s, 3 H), 2.28 (s, 3.3 H), 1.74–1.58 (m, 2.2 H), 1.20–1.14 (m, 0.7 H), 1.10–1.01 (m, 2 H), 0.72 (d, *J* = 7.5 Hz, 1.9 H), 0.48–0.42 (m, 1 H), 0.23–0.17 (m, 1.1 H), 0.02 (s, 5.6 H), -0.12 (s, 9 H), -0.21 (s, 4.3 H). **^{13}C NMR (125 MHz, $CDCl_3$)** δ 142.8, 142.5, 141.3, 140.8, 140.5, 137.8, 137.7, 137.6, 129.2, 128.9, 128.3, 128.0, 127.9, 127.1, 127.03, 127.02, 126.97, 126.93, 126.8, 126.7, 126.6, 61.1, 60.92, 60.88, 32.0, 28.7, 28.5, 21.4, 21.3, 12.6, 11.5, 11.1, -2.0, -2.1, -2.6. **HRMS** (ESI) m/z calcd. For $C_{19}H_{27}NNaO_2SSi$ $[M+Na]^+$: 384.1424, found: 384.1426.

2.4 Gram-Scale Reaction and Protecting Group Transformation

2.4.1 Gram-Scale Reaction

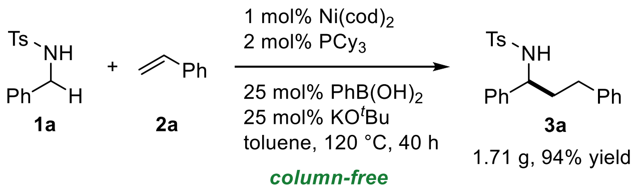

In a nitrogen-filled glovebox, a 50 mL oven-dried sealed tube was charged with *N*-tosyl amine **1a** (5 mmol, 1.3 g, 1.0 equiv.), styrene **2a** (10 mmol, 1.2 mL, 2.0 equiv.), $Ni(cod)_2$ (0.05 mmol, 13.8 mg, 1 mol%), PCy_3 (0.1 mmol, 28.0 mg, 2 mol%), $PhB(OH)_2$ (1.25 mmol, 0.15 g, 25 mol%) and KO^tBu (1.25 mmol, 0.14 g, 25 mol%). Toluene (7.5 mL) was added. The tube was equipped with a magnetic stir bar, sealed, and the reaction mixture was stirred at 120 °C for 40 h. After this time, the reaction was cooled to room temperature, and filtered with silica gel, then concentrated under reduced pressure. The crude product mixture was dissolved in a small amount of DCM. Then a large amount of pentane was added to precipitate the product out. Collected the precipitation, which was washed with pentane, affording the title compound (1.71 g, 94% yield).

2.4.2 Protecting Group Transformation

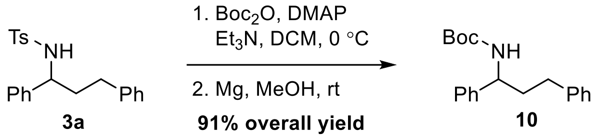

Step 1: A 4 ml vial was charged with α-branched *N*-tosyl amine **3a** (0.3 mmol, 109.5 mg, 1.0 equiv.), DMAP (0.18 mmol, 22.0 mg, 0.6 equiv.) and anhydrous DCM (2.0 mL). Boc_2O (0.6 mmol, 130.9 mg, 2.0 equiv.) and Et_3N (0.9 mmol, 125.0 μL, 3.0 equiv.) was added at 0 ºC. The reaction mixture was stirred at room temperature overnight and then concentrated under reduced pressure. Purification by column chromatography afforded *N*-Boc product.

Step 2: The above compound was dissolved in MeOH (15 mL). Mg powder (15 mmol, 360 mg, 50 equiv.) was added at 0 ºC. The reaction mixture was stirred vigorously until the starting material was consumed completely. The reaction mixture was quenched with sat. NH_4Cl aqueous, extracted with DCM (10 mL×3). The combined organic layer was dried over Na_2SO_4 and concentrated under reduced pressure. Purification by column chromatography afforded the desired product **10** (84.9 mg, 91% overall yield).

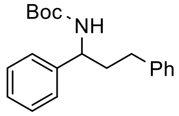

***tert*-Butyl (1,3-diphenylpropyl)carbamate (10)**

Following the above-mentioned procedure, the title compound was isolated as white solid (84.9 mg, 91% overall yield). **^{1}H NMR (500 MHz, $CDCl_3$)** δ 7.33 (t, *J* = 7.5 Hz, 2 H), 7.27–7.25 (m, 5 H), 7.18–7.14 (m, 3 H), 4.86 (d, *J* = 7.5 Hz, 1 H), 4.67–4.45 (m, 1 H), 2.68–2.62 (m, 1 H), 2.59–2.53 (m, 1 H), 2.09–2.03 (m, 2 H), 1.42 (s, 9 H). **^{13}C NMR (125 MHz, $CDCl_3$)** δ 155.1, 142.6, 141.3, 128.4, 128.25, 128.22, 127.1, 126.3, 125.8, 79.2, 54.5, 38.4, 32.4, 28.3. **HRMS** (ESI) m/z calcd. For $C_{20}H_{25}NNaO_2$ $[M+Na]^+$: 334.1778, found: 334.1772.

2.5 Nickel-Catalyzed Enantioselective Hydroalkylation of Olefins with *N*-Sulfonyl Amines

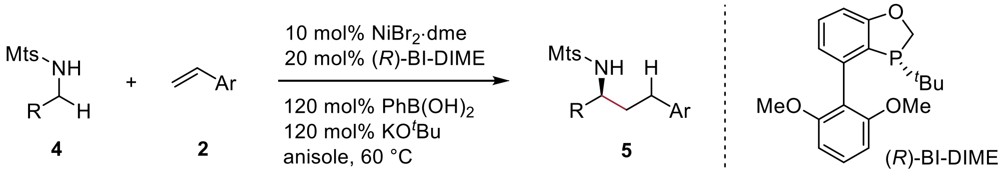

General Procedure C: In a nitrogen-filled glovebox, a 4 mL oven-dried vial was charged with *N*-sulfonyl amine **4** (0.1 mmol, 1.0 equiv.), olefin **2** (0.2 mmol, 2.0 equiv.), $NiBr_2$·dme (0.01 mmol, 3.1 mg, 10 mol%), (*R*)-BI-DIME (0.02 mmol, 6.6 mg, 20 mol%), $PhB(OH)_2$ (0.12 mmol, 14.6 mg, 120 mol%) and KO^tBu (0.12 mmol, 13.4 mg, 120 mol%). Anisole (0.2 mL) was added. The vial was equipped with a magnetic stir bar, sealed, and the reaction mixture was stirred at 60 °C for 72 h. The reaction mixture was cooled to room temperature and concentrated under reduced pressure. Purification by column chromatography afforded the desired product.

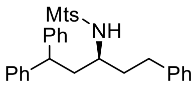

(*S*)-2,4,6-Trimethyl-*N*-(1,1,5-triphenylpentan-3-yl)benzenesulfonamide (5a)

Following the **General Procedure C**, the title compound was isolated as white solid (27.5 mg, 55% yield). **^{1}H NMR (500 MHz, $CDCl_3$)** δ 7.21–7.12 (m, 9 H), 7.00 (d, *J* = 7.0 Hz, 2 H), 6.95–6.94 (m, 4 H), 6.87 (d, *J* = 7.0 Hz, 2 H), 4.49 (d, *J* = 9.5 Hz, 1 H), 3.84 (t, *J* = 8.0 Hz, 1 H), 3.10–3.03 (m, 1 H), 2.54–2.41 (m, 8 H), 2.32 (s, 3 H), 2.23–2.17 (m, 1 H), 2.10–2.04 (m, 1 H), 1.84–1.77 (m, 1 H), 1.70–1.63 (m, 1 H). **^{13}C NMR (125 MHz, $CDCl_3$)** δ 144.0, 143.6, 142.0, 141.0, 139.0, 134.9, 132.0, 128.6, 128.5, 128.35, 128.29, 127.7, 127.5, 126.3, 125.9, 51.6, 47.4, 41.5, 37.0, 31.3, 22.9, 20.9. **HRMS** (ESI) m/z calcd. For $C_{32}H_{35}NNaO_2S$ $[M+Na]^+$: 520.2281, found: 520.2296.

The enantiomeric ratio was determined to be 92.0:8.0 by SFC analysis: IC-3 column, MeOH/CO_2 = 15:85, 1.0 mL/min, 210 nm, t_{major} = 8.6 min, t_{minor} = 9.7 min.

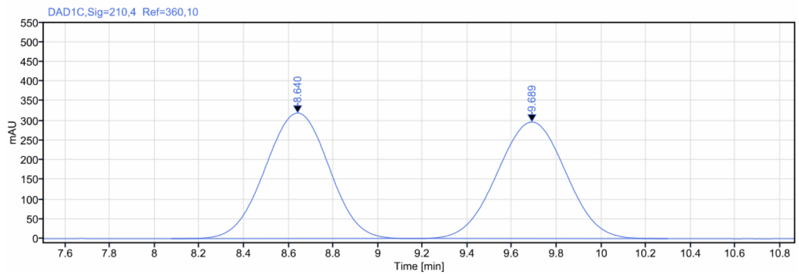

Signal: DAD1C,Sig=210,4 Ref=360,10

RT [min]	Type	Width [min]	Area	Height	Area%	Name
8.640	VB	1.07	6362.67	319.32	50.04	
9.689	BB	1.15	6352.11	296.43	49.96	
		Sum	12714.78			

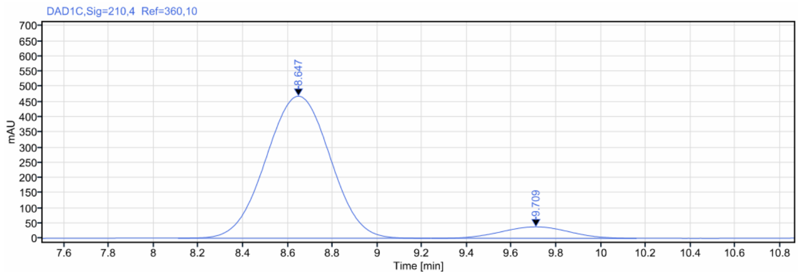

Signal: DAD1C,Sig=210,4 Ref=360,10

RT [min]	Type	Width [min]	Area	Height	Area%	Name
8.647	VB	1.13	9372.66	468.89	92.04	
9.709	BV	0.92	810.91	38.01	7.96	
		Sum	10183.57			

Supplementary Fig. 1 SFC spectra of **5a**

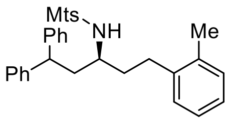

(*S*)-*N*-(1,1-Diphenyl-5-(*o*-tolyl)pentan-3-yl)-2,4,6-trimethylbenzenesulfonamide (5b)

Following the **General Procedure C**, the title compound was isolated as white solid (49.1 mg, 96% yield). **^{1}H NMR (500 MHz, CDCl$_3$)** δ 7.23–7.12 (m, 6 H), 7.08–7.04 (m, 4 H), 6.99–6.98 (m, 3 H), 6.91 (s, 2 H), 6.73 (d, *J* = 7.5 Hz, 1 H), 4.74–4.71 (m, 1 H), 3.87 (d, *J* = 7.5 Hz, 1 H), 3.11–3.04 (m, 1 H), 2.52–2.42 (m, 7 H), 2.38–2.28 (m, 4 H), 2.24–2.18 (m, 1 H), 2.15–2.08 (m, 4 H), 1.77–1.70 (m, 1 H), 1.61–1.54 (m, 1 H). **^{13}C NMR (125 MHz, CDCl$_3$)** δ 143.9, 143.7, 142.0, 139.3, 139.0, 135.7, 134.8, 132.0, 130.1, 128.60, 128.55, 128.4, 127.7, 127.5, 126.3, 126.0, 125.9, 51.9, 47.5, 41.6, 35.4, 28.9, 22.9, 20.9, 19.0. **HRMS** (ESI) m/z calcd. For $C_{33}H_{37}NNaO_2S$ $[M+Na]^+$: 534.2437, found: 534.2450.

The enantiomeric ratio was determined to be 92.8:7.2 by SFC analysis: IC-3 column, MeOH/CO_2 = 10:90, 1.0 mL/min, 210 nm, t_{major} = 17.3 min, t_{minor} = 19.8 min.

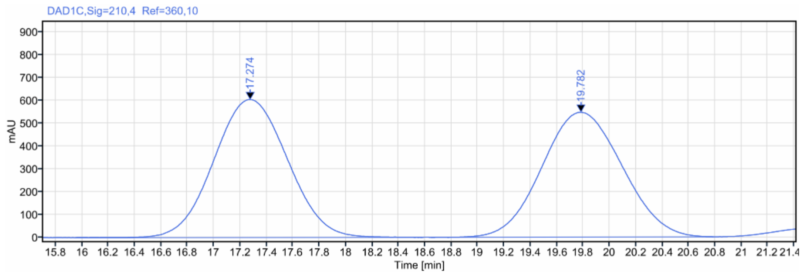

Signal: DAD1C,Sig=210,4 Ref=360,10

RT [min]	Type	Width [min]	Area	Height	Area%	Name
17.274	BB	2.63	23507.78	602.42	50.40	
19.782	BB	2.14	23137.45	545.26	49.60	
		Sum	46645.23			

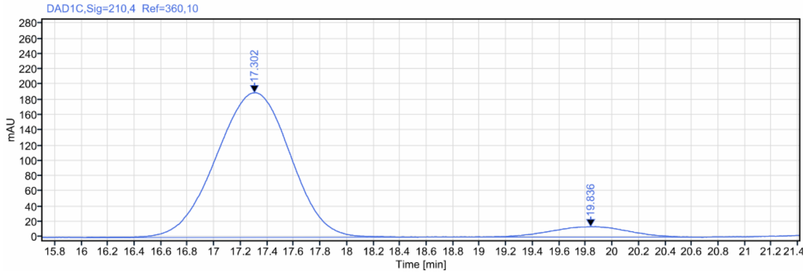

Signal: DAD1C,Sig=210,4 Ref=360,10

RT [min]	Type	Width [min]	Area	Height	Area%	Name
17.302	BB	2.50	7399.50	189.70	92.77	
19.836	BB	1.97	576.59	13.64	7.23	
		Sum	7976.09			

Supplementary Fig. 2 SFC spectra of **5b**

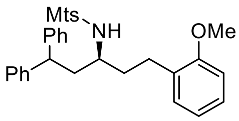

(*S*)-*N*-(5-(2-Methoxyphenyl)-1,1-diphenylpentan-3-yl)-2,4,6-trimethylbenzenesulfonamide (5c)

Following the **General Procedure C**, the title compound was isolated as white solid (46.3 mg, 88% yield). **^{1}H NMR (500 MHz, CDCl$_3$)** δ 7.22–7.12 (m, 7 H), 7.03–7.02 (m, 2 H), 6.99–6.97 (m, 2 H), 6.92 (s, 2 H), 6.81–6.76 (m, 2 H), 4.57 (d, J = 9.0 Hz, 1 H), 3.86 (t, J = 7.5 Hz, 1 H), 3.80 (s, 3 H), 3.12–3.05 (m, 1 H), 2.58–2.53 (m, 1 H), 2.49 (s, 6 H), 2.42–2.36 (m, 1 H), 2.31 (s, 3 H), 2.20–2.09 (m, 2 H), 1.81–1.74 (m, 1 H), 1.68–1.61 (m, 1 H). **^{13}C NMR (125 MHz, CDCl$_3$)** δ 157.1, 144.0, 143.9, 141.8, 139.0, 134.9, 131.9, 129.8, 129.6, 128.6, 128.4, 127.7, 127.6, 127.2, 126.3, 126.2, 120.5, 110.2, 55.1, 51.8, 47.5, 41.2, 34.8, 25.7, 22.9, 20.9. **HRMS** (ESI) m/z calcd. For $C_{33}H_{37}NNaO_3S$ [M+Na]$^+$: 550.2386, found: 550.2392.

The enantiomeric ratio was determined to be 85.7:14.3 by SFC analysis: IC-3 column, MeOH/CO_2 = 20:80, 1.0 mL/min, 210 nm, t_{major} = 6.4 min, t_{minor} = 7.6 min.

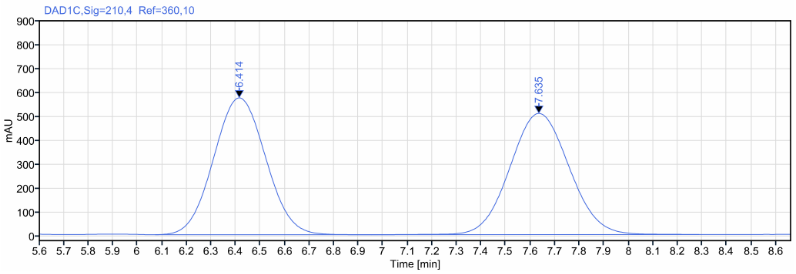

Signal: DAD1C,Sig=210,4 Ref=360,10

RT [min]	Type	Width [min]	Area	Height	Area%	Name
6.414	BB	0.88	8448.99	571.88	49.77	
7.635	BV	1.34	8527.63	506.66	50.23	
		Sum	16976.61			

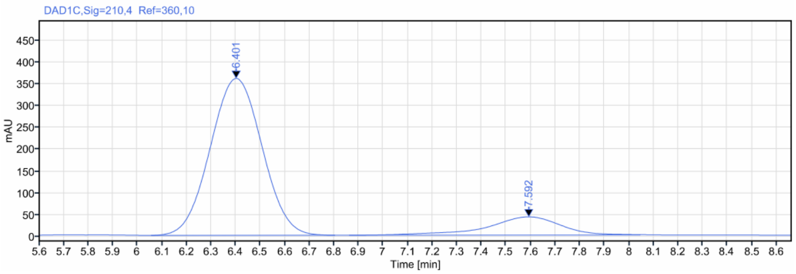

Signal: DAD1C,Sig=210,4 Ref=360,10

RT [min]	Type	Width [min]	Area	Height	Area%	Name
6.401	VV	0.75	5270.57	359.33	85.66	
7.592	BV	1.18	882.11	41.87	14.34	
		Sum	6152.68			

Supplementary Fig. 3 SFC spectra of **5c**

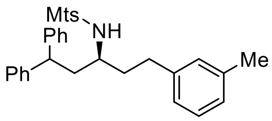

(*S*)-*N*-(1,1-Diphenyl-5-(*m*-tolyl)pentan-3-yl)-2,4,6-trimethylbenzenesulfonamide (5d)

Following the **General Procedure C**, the title compound was isolated as white solid (35.4 mg, 69% yield). **^{1}H NMR (500 MHz, CDCl$_3$)** δ 7.20–7.11 (m, 6 H), 7.08 (t, J = 7.5 Hz, 1 H), 6.99–6.93 (m, 7 H), 6.71–6.69 (m, 2 H), 4.62–4.60 (m, 1 H), 3.82 (t, J = 7.5 Hz, 1 H), 3.10–3.03 (m, 1 H), 2.51–2.44 (m, 7 H), 2.41–2.35 (m, 1 H), 2.31 (s, 3 H), 2.26 (s, 3 H), 2.23–2.15 (m, 1 H), 2.10–2.04 (m, 1 H), 1.82–1.75 (m, 1 H), 1.69–1.64 (m, 1 H). **^{13}C NMR (125 MHz, CDCl$_3$)** δ 144.0, 143.6, 141.9, 141.1, 139.0, 137.8, 134.9, 132.0, 129.1, 128.5, 128.4, 128.2, 127.7, 127.5, 126.6, 126.3, 126.2, 125.3, 51.6, 47.4, 41.5, 37.1, 31.2, 22.9, 21.3, 20.9. **HRMS** (ESI) m/z calcd. For $C_{33}H_{37}NNaO_2S$ [M+Na]$^+$: 534.2437, found: 534.2449.

The enantiomeric ratio was determined to be 87.7:12.3 by SFC analysis: IC-3 column, MeOH/CO_2 = 15:85, 1.0 mL/min, 210 nm, t_{major} = 9.3 min, t_{minor} = 11.0 min.

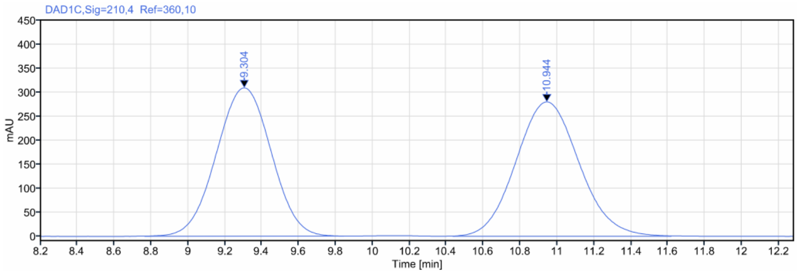

Signal: DAD1C,Sig=210,4 Ref=360,10

RT [min]	Type	Width [min]	Area	Height	Area%	Name
9.304	VB	1.08	6404.85	309.16	48.95	
10.944	VV	1.18	6679.85	280.07	51.05	
		Sum	13084.71			

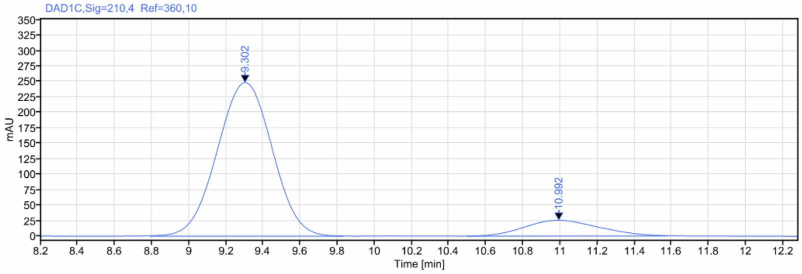

Signal: DAD1C,Sig=210,4 Ref=360,10

RT [min]	Type	Width [min]	Area	Height	Area%	Name
9.302	VV	1.04	5130.79	248.27	87.69	
10.992	BV	1.08	720.45	25.77	12.31	
		Sum	5851.25			

Supplementary Fig. 4 SFC spectra of **5d**

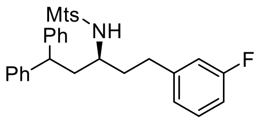

(*S*)-*N*-(5-(3-Fluorophenyl)-1,1-diphenylpentan-3-yl)-2,4,6-trimethylbenzenesulfonamide (5e)

Following the **General Procedure C**, the title compound was isolated as white solid (18.2 mg, 35% yield). **^{1}H NMR (500 MHz, CDCl$_3$)** δ 7.22–7.09 (m, 7 H), 7.01–6.99 (m, 2 H), 6.97–6.95 (m, 4 H), 6.85–6.81 (m, 1 H), 6.61 (d, *J* = 7.5 Hz, 1 H), 6.52–6.49 (m, 1 H), 4.49 (d, *J* = 9.5 Hz, 1 H), 3.84 (t, *J* = 7.5 Hz, 1 H), 3.08–3.00 (m, 1 H), 2.54–2.40 (m, 8 H), 2.33 (s, 3 H), 2.21–2.16 (m, 1 H), 2.11–2.05 (m, 1 H), 1.83–1.76 (m, 1 H), 1.68–1.59 (m, 1 H). **^{13}C NMR (125 MHz, CDCl$_3$)** δ 162.8 (d, *J* = 243.9 Hz), 143.9, 143.6, 143.5, 142.2, 139.0, 134.8, 132.1, 129.7 (d, *J* = 8.3 Hz), 128.6, 128.5, 127.7, 127.5, 126.4 (d, *J* = 5.0 Hz), 123.9 (d, *J* = 2.8 Hz), 115.1 (d, *J* = 20.8 Hz), 112.7 (d, *J* = 21.0 Hz), 51.4, 47.5, 41.6, 36.7, 31.1 (d, *J* = 1.5 Hz), 22.9, 20.9. **^{19}F NMR (470 MHz, CDCl$_3$)** δ -113.5. **HRMS** (ESI) m/z calcd. For $C_{32}H_{34}FNNaO_2S$ $[M+Na]^+$: 538.2186, found: 538.2195.

The enantiomeric ratio was determined to be 92.9:7.1 by SFC analysis: IC-3 column, MeOH/CO_2 = 10:90, 1.0 mL/min, 210 nm, t_{major} = 12.9 min, t_{minor} = 14.4 min.

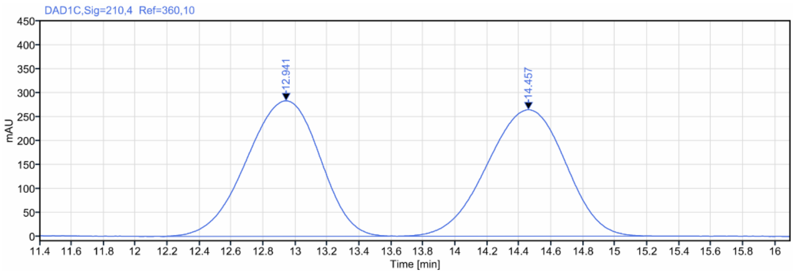

Signal: DAD1C,Sig=210,4 Ref=360,10

RT [min]	Type	Width [min]	Area	Height	Area%	Name
12.941	VV	1.42	9082.01	283.16	50.09	
14.457	VV	1.55	9048.21	264.23	49.91	
		Sum	18130.22			

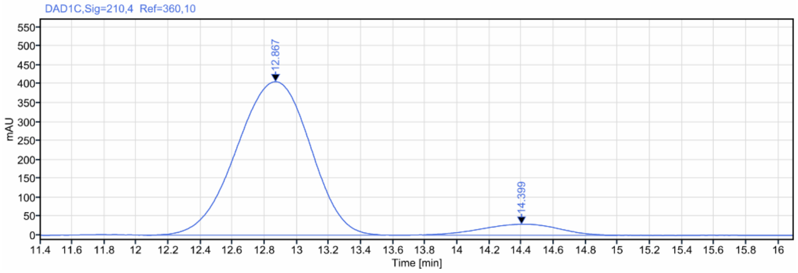

Signal: DAD1C,Sig=210,4 Ref=360,10

RT [min]	Type	Width [min]	Area	Height	Area%	Name
12.867	BV	1.49	12975.45	405.71	92.87	
14.399	VV	1.21	996.39	29.35	7.13	
		Sum	13971.84			

Supplementary Fig. 5 SFC spectra of **5e**

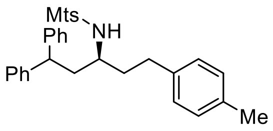

(*S*)-*N*-(1,1-Diphenyl-5-(*p*-tolyl)pentan-3-yl)-2,4,6-trimethylbenzenesulfonamide (5f)

Following the **General Procedure C**, the title compound was isolated as white solid (28.0 mg, 55% yield). **^{1}H NMR (500 MHz, CDCl$_3$)** δ 7.20–7.11 (m, 6 H), 7.00–6.92 (m, 8 H), 6.75 (d, J = 8.0 Hz, 2 H), 4.61 (d, J = 9.5 Hz, 1 H), 3.83 (t, J = 7.5 Hz, 1 H), 3.08–3.01 (m, 1 H), 2.50–2.42 (m, 7 H), 2.41–2.34 (m, 1 H), 2.31 (s, 3 H), 2.30 (s, 3 H), 2.21–2.16 (m, 1 H), 2.10–2.04 (m, 1 H), 1.81–1.74 (m, 1 H), 1.67–1.60 (m, 1 H). **^{13}C NMR (125 MHz, CDCl$_3$)** δ 144.0, 143.7, 141.9, 139.0, 138.0, 135.2, 134.9, 132.0, 129.0, 128.5, 128.4, 128.2, 127.7, 127.5, 126.25, 126.24, 51.5, 47.4, 41.5, 37.1, 30.9, 22.9, 20.94, 20.90. **HRMS** (ESI) m/z calcd. For $C_{33}H_{37}NNaO_2S$ [M+Na]$^+$: 534.2437, found: 534.2444.

The enantiomeric ratio was determined to be 90.4:9.6 by SFC analysis: IC-3 column, MeOH/CO_2 = 15:85, 1.0 mL/min, 210 nm, t_{major} = 9.6 min, t_{minor} = 10.8 min.

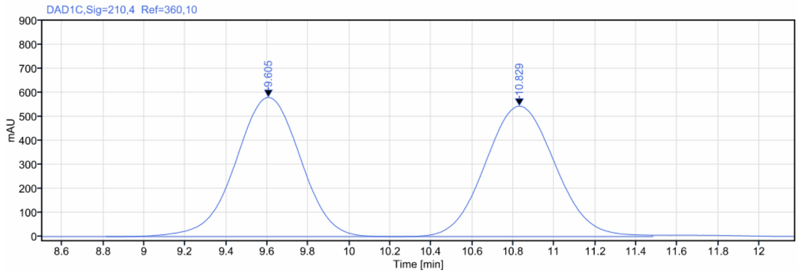

Signal: DAD1C,Sig=210,4 Ref=360,10

RT [min]	Type	Width [min]	Area	Height	Area%	Name
9.605	VB	1.41	12778.71	579.92	49.75	
10.829	BV	1.26	12905.19	543.42	50.25	
		Sum	25683.90			

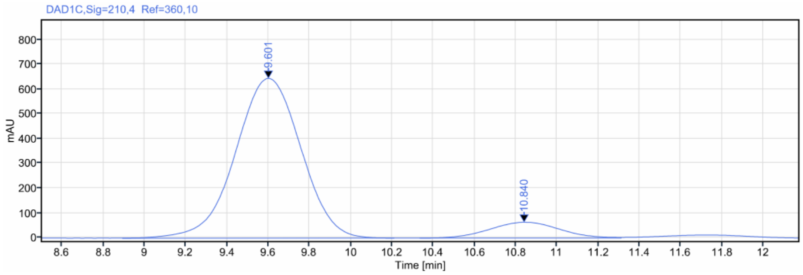

Signal: DAD1C,Sig=210,4 Ref=360,10

RT [min]	Type	Width [min]	Area	Height	Area%	Name
9.601	VV	1.32	14252.58	645.83	90.39	
10.840	VV	0.98	1514.70	64.09	9.61	
		Sum	15767.28			

Supplementary Fig. 6 SFC spectra of **5f**

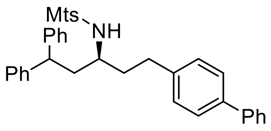

(*S*)-*N*-(5-([1,1'-Biphenyl]-4-yl)-1,1-diphenylpentan-3-yl)-2,4,6-trimethylbenzenesulfonamide (5g)

Following the **General Procedure C**, the title compound was isolated as white solid (22.4 mg, 39% yield). **^{1}H NMR (500 MHz, $CDCl_3$)** δ 7.57–7.55 (m, 2 H), 7.45–7.42 (m, 2 H), 7.40–7.38 (m, 2 H), 7.35–7.32 (m, 1 H), 7.20–7.11 (m, 6 H), 7.01–7.00 (m, 2 H), 6.95–6.90 (m, 6 H), 4.58 (d, *J* = 9.5 Hz, 1 H), 3.84 (t, *J* = 7.5 Hz, 1 H), 3.10–3.03 (m, 1 H), 2.59–2.47 (m, 8 H), 2.32 (s, 3 H), 2.24–2.19 (m, 1 H), 2.12–2.06 (m, 1 H), 1.88–1.81 (m, 1 H), 1.75–1.68 (m, 1 H). **^{13}C NMR (125 MHz, $CDCl_3$)** δ 143.9, 143.7, 142.0, 141.0, 140.1, 139.1, 138.8, 134.8, 132.0, 128.75, 128.73, 128.6, 128.5, 127.8, 127.5, 127.0, 126.9, 126.31, 126.29, 51.4, 47.4, 41.6, 37.0, 31.0, 22.9, 20.9. **HRMS** (ESI) m/z calcd. For $C_{38}H_{39}NNaO_2S$ $[M+Na]^+$: 596.2594, found: 596.2609.

The enantiomeric ratio was determined to be 93.2:6.8 by SFC analysis: IC-3 column, MeOH/CO_2 = 15:85, 1.0 mL/min, 210 nm, t_{major} = 23.9 min, t_{minor} = 25.6 min.

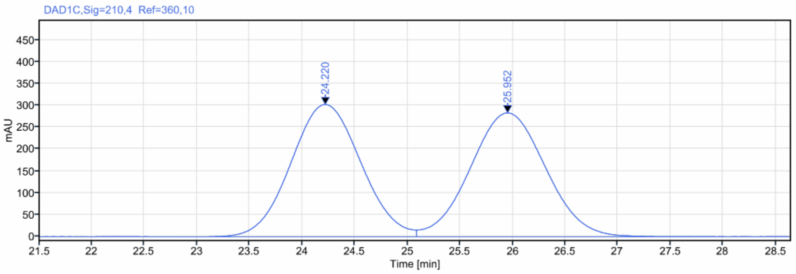

Signal: DAD1C,Sig=210,4 Ref=360,10

RT [min]	Type	Width [min]	Area	Height	Area%	Name
24.220	BV	2.26	14067.66	302.47	49.84	
25.952	VBA	2.94	14159.21	283.36	50.16	
		Sum	28226.87			

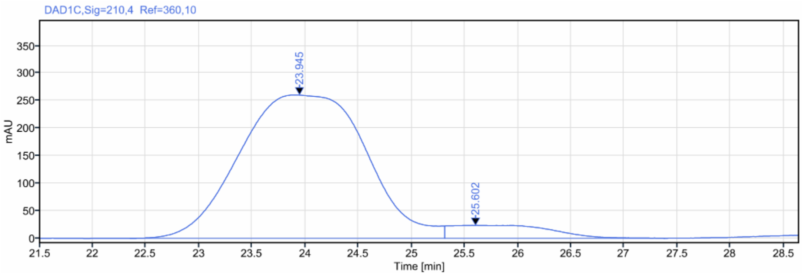

Signal: DAD1C,Sig=210,4 Ref=360,10

RT [min]	Type	Width [min]	Area	Height	Area%	Name
23.945	BV	2.96	21639.29	260.32	93.24	
25.602	VB	2.07	1569.81	23.48	6.76	
		Sum	23209.10			

Supplementary Fig. 7 SFC spectra of **5g**

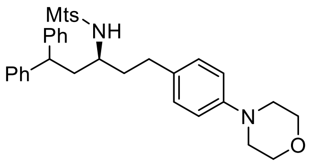

(*S*)-2,4,6-Trimethyl-*N*-(5-(4-morpholinophenyl)-1,1-diphenylpentan-3-yl)benzenesulfonamide (5h)

Following the **General Procedure C**, the title compound was isolated as white solid (30.1 mg, 52% yield). **^{1}H NMR (500 MHz, CDCl$_3$)** δ 7.21–7.11 (m, 6 H), 7.00 (d, *J* = 7.5 Hz, 2 H), 6.93–6.92 (m, 4 H), 6.81 (d, *J* = 8.5 Hz, 2 H), 6.74 (d, *J* = 8.5 Hz, 2 H), 4.59 (d, *J* = 9.5 Hz, 1 H), 3.87–3.80 (m, 5 H), 3.11–3.02 (m, 5 H), 2.49–2.32 (m, 11 H), 2.22–2.16 (m, 1 H), 2.08–2.02 (m, 1 H), 1.81–1.74 (m, 1 H), 1.68–1.61 (m, 1 H). **^{13}C NMR (125 MHz, CDCl$_3$)** δ 149.5, 144.0, 143.6, 141.9, 139.0, 134.9, 132.7, 132.0, 129.0, 128.5, 128.4, 127.7, 127.5, 126.3, 115.9, 66.9, 51.6, 49.7, 47.4, 41.4, 37.2, 30.4, 22.9, 20.9. **HRMS** (ESI) m/z calcd. For $C_{36}H_{42}N_2NaO_3S$ $[M+Na]^+$: 605.2808, found: 605.2827.

The enantiomeric ratio was determined to be 88.9:11.1 by SFC analysis: IC-3 column, MeOH/CO_2 = 25:75, 1.0 mL/min, 210 nm, t_{major} = 14.8 min, t_{minor} = 16.6 min.

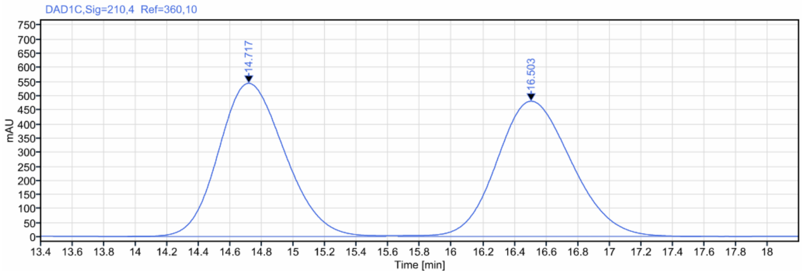

Signal: DAD1C,Sig=210,4 Ref=360,10

RT [min]	Type	Width [min]	Area	Height	Area%	Name
14.717	BV	1.63	15784.22	542.36	50.04	
16.503	VB	1.92	15757.54	478.90	49.96	
		Sum	31541.76			

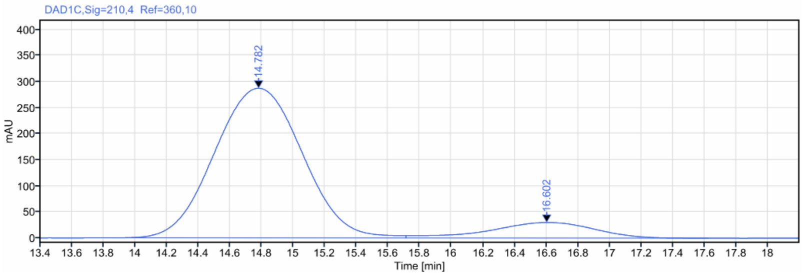

Signal: DAD1C,Sig=210,4 Ref=360,10

RT [min]	Type	Width [min]	Area	Height	Area%	Name
14.782	BV	1.86	11086.73	287.10	88.88	
16.602	VB	1.91	1387.51	30.19	11.12	
		Sum	12474.24			

Supplementary Fig. 8 SFC spectra of **5h**

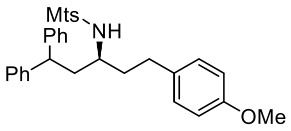

(*S*)-*N*-(5-(4-Methoxyphenyl)-1,1-diphenylpentan-3-yl)-2,4,6-trimethylbenzenesulfonamide (5i)

Following the **General Procedure C**, the title compound was isolated as white solid (25.4 mg, 48% yield). ^{1}**H NMR (500 MHz, CDCl₃)** δ 7.23–7.12 (m, 6 H), 7.01–6.99 (m, 2 H), 6.95–6.94 (m, 4 H), 6.78–6.76 (m, 2 H), 6.73–6.70 (m, 2 H), 4.51 (d, *J* = 10.0 Hz, 1 H), 3.83 (t, *J* = 7.5 Hz, 1 H), 3.78 (s, 3 H), 3.08–3.01 (m, 1 H), 2.46–2.35 (m, 8 H), 2.32 (s, 3 H), 2.22–2.14 (m, 1 H), 2.09–2.03 (m, 1 H), 1.80–1.73 (m, 1 H), 1.67–1.60 (m, 1 H). ^{13}**C NMR (125 MHz, CDCl₃)** δ 157.8, 144.0, 143.7, 142.0, 139.0, 134.9, 133.1, 132.0, 129.2, 128.6, 128.5, 128.4, 127.8, 127.5, 126.29, 126.28, 113.7, 55.2, 51.4, 47.4, 41.5, 37.3, 30.4, 22.9, 20.9. **HRMS** (ESI) m/z calcd. For $C_{33}H_{37}NNaO_3S$ $[M+Na]^+$: 550.2386, found: 550.2396.

The enantiomeric ratio was determined to be 89.2:10.8 by SFC analysis: IC-3 column, MeOH/CO_2 = 15:85, 1.0 mL/min, 210 nm, t_{major} = 12.9 min, t_{minor} = 15.0 min.

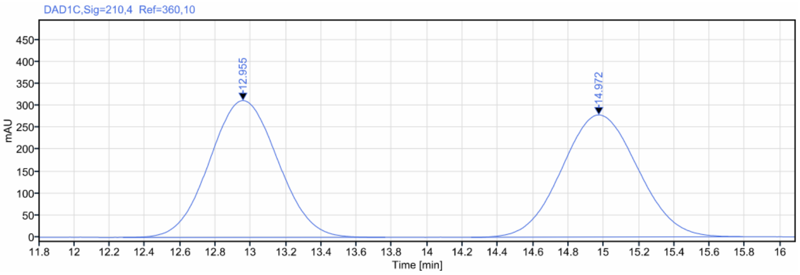

Signal: DAD1C,Sig=210,4 Ref=360,10

RT [min]	Type	Width [min]	Area	Height	Area%	Name
12.955	VB	1.48	8412.02	311.65	50.13	
14.972	VV	1.54	8369.41	278.34	49.87	
		Sum	16781.42			

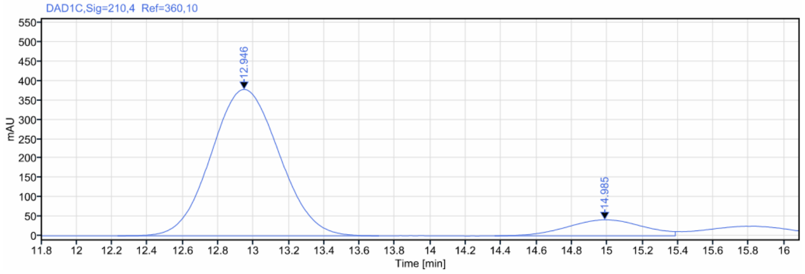

Signal: DAD1C,Sig=210,4 Ref=360,10

RT [min]	Type	Width [min]	Area	Height	Area%	Name
12.946	BB	1.48	10125.22	378.07	89.21	
14.985	BV	1.02	1224.48	41.40	10.79	
		Sum	11349.70			

Supplementary Fig. 9 SFC spectra of **5i**

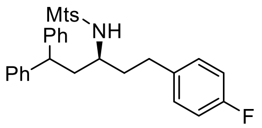

(*S*)-*N*-(5-(4-Fluorophenyl)-1,1-diphenylpentan-3-yl)-2,4,6-trimethylbenzenesulfonamide (5j)

Following the **General Procedure C**, the title compound was isolated as white solid (24.6 mg, 48% yield). **^{1}H NMR (500 MHz, $CDCl_3$)** δ 7.21–7.12 (m, 6 H), 6.99–6.97 (m, 2 H), 6.94–6.91 (m, 4 H), 6.85–6.81 (m, 2 H), 6.78–6.74 (m, 2 H), 4.51 (d, J = 10.0 Hz, 1 H), 3.81 (t, J = 7.5 Hz, 1 H), 3.05–2.98 (m, 1 H), 2.51–2.41 (m, 8 H), 2.33 (s, 3 H), 2.21–2.15 (m, 1 H), 2.08–2.03 (m, 1 H), 1.82–1.75 (m, 1 H), 1.68–1.61 (m, 1 H). **^{13}C NMR (125 MHz, $CDCl_3$)** δ 161.2 (d, J = 242.0 Hz), 143.9, 143.6, 142.1, 139.0, 136.55, 136.53, 134.8, 132.0, 129.7 (d, J = 7.8 Hz), 128.6, 128.5, 127.7, 127.5, 126.3 (d, J = 1.6 Hz), 115.0 (d, J = 21.0 Hz), 51.2, 47.4, 41.6, 37.2, 30.5, 22.9, 20.9. **^{19}F NMR (470 MHz, $CDCl_3$)** δ -117.7. **HRMS** (ESI) m/z calcd. For $C_{32}H_{34}FNNaO_2S$ $[M+Na]^+$: 538.2186, found: 538.2198.

The enantiomeric ratio was determined to be 91.4:8.6 by SFC analysis: IC-3 column, MeOH/CO_2 = 10:90, 1.0 mL/min, 210 nm, t_{major} = 12.9 min, t_{minor} = 14.2 min.

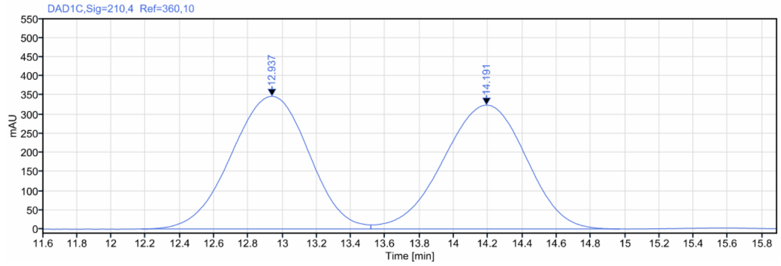

Signal: DAD1C,Sig=210,4 Ref=360,10

RT [min]	Type	Width [min]	Area	Height	Area%	Name
12.937	BV	1.34	10948.09	347.09	50.00	
14.191	VB	1.45	10946.19	323.68	50.00	
		Sum	21894.28			

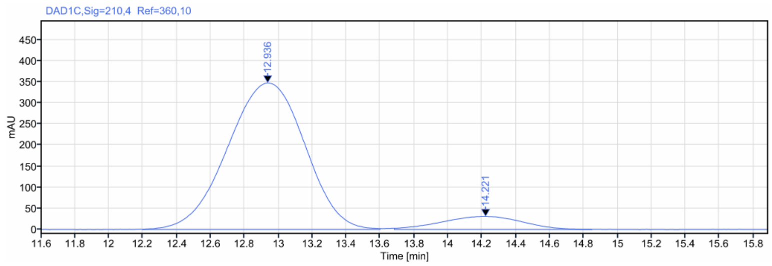

Signal: DAD1C,Sig=210,4 Ref=360,10

RT [min]	Type	Width [min]	Area	Height	Area%	Name
12.936	VV	1.40	11023.30	347.69	91.39	
14.221	VV	1.16	1038.43	31.10	8.61	
		Sum	12061.72			

Supplementary Fig. 10 SFC spectra of **5j**

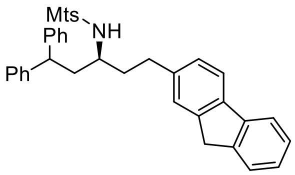

(*S*)-*N*-(5-(9*H*-Fluoren-2-yl)-1,1-diphenylpentan-3-yl)-2,4,6-trimethylbenzenesulfonamide (5k)

Following the **General Procedure C**, the title compound was isolated as white solid (24.9 mg, 43% yield). **^{1}H NMR (500 MHz, CDCl$_3$)** δ 7.74 (d, *J* = 7.5 Hz, 1 H), 7.58 (d, *J* = 8.0 Hz, 1 H), 7.53 (d, *J* = 7.5 Hz, 1 H), 7.37 (t, *J* = 7.0 Hz, 1 H), 7.30–7.27 (m, 1 H), 7.19–7.11 (m, 5 H), 7.09–7.06 (m, 1 H), 6.97–6.95 (m, 5 H), 6.91–6.89 (m, 3 H), 4.58 (d, *J* = 10.0 Hz, 1 H), 3.82 (t, *J* = 7.5 Hz, 1 H), 3.77 (s, 2 H), 3.11–3.04 (m, 1 H), 2.61–2.49 (m, 2 H), 2.46 (s, 6 H), 2.31 (s, 3 H), 2.24–2.18 (m, 1 H), 2.13–2.07 (m, 1 H), 1.89–1.82 (m, 1 H), 1.74–1.67 (m, 1 H). **^{13}C NMR (125 MHz, CDCl$_3$)** δ 143.9, 143.7, 143.4, 143.1, 142.0, 141.6, 139.9, 139.6, 139.0, 134.8, 132.0, 128.5, 128.4, 127.7, 127.5, 127.0, 126.7, 126.33, 126.30, 126.2, 125.1, 125.0, 119.7, 119.6, 51.3, 47.5, 41.6, 37.4, 36.7, 31.5, 22.9, 20.9. **HRMS** (ESI) m/z calcd. For $C_{39}H_{39}NNaO_2S$ $[M+Na]^+$: 608.2594, found: 608.2605.

The enantiomeric ratio was determined to be 89.9:10.1 by SFC analysis: IC-3 column, MeOH/CO_2 = 25:75, 1.0 mL/min, 210 nm, t_{major} = 14.4 min, t_{minor} = 16.6 min.

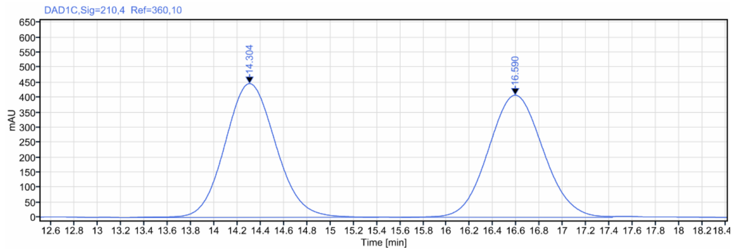

Signal: DAD1C,Sig=210,4 Ref=360,10

RT [min]	Type	Width [min]	Area	Height	Area%	Name
14.304	BV	2.17	13574.89	446.78	50.35	
16.590	BV	1.82	13385.95	407.90	49.65	
		Sum	26960.84			

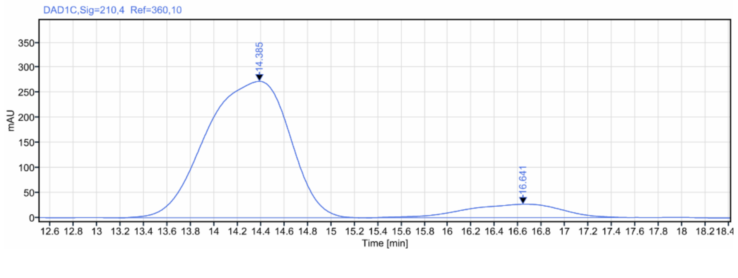

Signal: DAD1C,Sig=210,4 Ref=360,10

RT [min]	Type	Width [min]	Area	Height	Area%	Name
14.385	BV	2.06	13528.94	272.23	89.89	
16.641	BB	2.25	1521.54	26.75	10.11	
		Sum	15050.47			

Supplementary Fig. 11 SFC spectra of **5k**

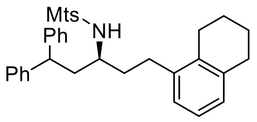

(*S*)-*N*-(1,1-Diphenyl-5-(5,6,7,8-tetrahydronaphthalen-1-yl)pentan-3-yl)-2,4,6-trimethylbenzenesulfonamide (5l)

Following the **General Procedure C**, the title compound was isolated as white solid (50.9 mg, 92% yield). **^{1}H NMR (500 MHz, CDCl$_3$)** δ 7.23–7.11 (m, 6 H), 7.06 (d, *J* = 7.0 Hz, 2 H), 6.99 (d, *J* = 7.5 Hz, 2 H), 6.95–6.88 (m, 4 H), 6.65 (d, *J* = 7.0 Hz, 1 H), 4.71 (d, *J* = 9.5 Hz, 1 H), 3.88 (t, *J* = 7.5 Hz, 1 H), 3.14–3.07 (m, 1 H), 2.74 (t, *J* = 6.0 Hz, 2 H), 2.49 (s, 6 H), 2.46–2.38 (m, 3 H), 2.30–2.23 (m, 4 H), 2.22–2.12 (m, 2 H), 1.76–1.69 (m, 5 H), 1.61–1.53 (m, 1 H). **^{13}C NMR (125 MHz, CDCl$_3$)** δ 143.8, 143.7, 141.9, 139.4, 138.9, 137.3, 134.9, 134.7, 131.9, 128.5, 128.4, 127.7, 127.6, 127.2, 126.30, 126.29, 125.9, 125.2, 52.2, 47.6, 41.6, 35.3, 30.1, 28.4, 25.8, 23.3, 23.0, 22.8, 20.9. **HRMS** (ESI) m/z calcd. For $C_{36}H_{41}NNaO_2S$ $[M+Na]^+$: 574.2750, found: 574.2756.

The enantiomeric ratio was determined to be 92.8:7.2 by SFC analysis: IC-3 column, MeOH/CO_2 = 25:75, 1.0 mL/min, 210 nm, t_{major} = 6.6 min, t_{minor} = 7.1 min.

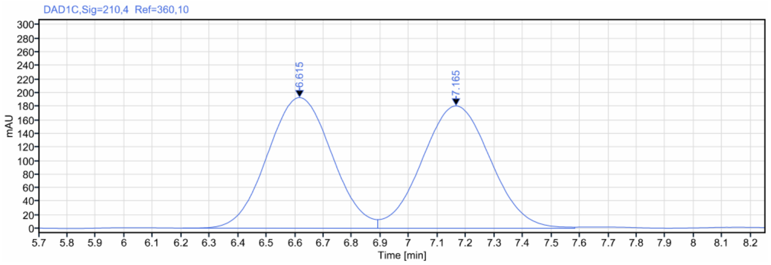

Signal: DAD1C,Sig=210,4 Ref=360,10

RT [min]	Type	Width [min]	Area	Height	Area%	Name
6.615	BV	0.69	3025.75	192.48	50.12	
7.165	VV	0.69	3011.08	180.18	49.88	
		Sum	6036.82			

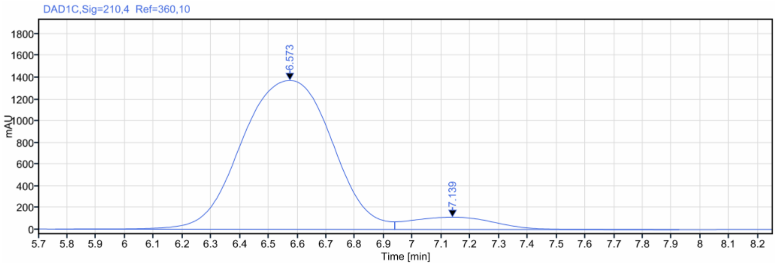

Signal: DAD1C,Sig=210,4 Ref=360,10

RT [min]	Type	Width [min]	Area	Height	Area%	Name
6.573	VV	1.18	29943.59	1370.01	92.83	
7.139	VB	0.99	2313.16	112.73	7.17	
		Sum	32256.75			

Supplementary Fig. 12 SFC spectra of **5l**

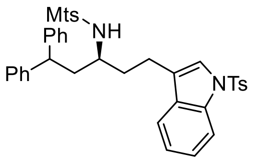

(*S*)-*N*-(1,1-Diphenyl-5-(1-tosyl-1*H*-indol-3-yl)pentan-3-yl)-2,4,6-trimethylbenzenesulfonamide (5m)

Following the **General Procedure C**, the title compound was isolated as light yellow solid (26.3 mg, 38% yield). **^{1}H NMR (500 MHz, CDCl$_3$)** δ 7.96 (d, *J* = 8.5 Hz, 1 H), 7.69 (d, *J* = 8.5 Hz, 2 H), 7.31–7.27 (m, 2 H), 7.20–7.11 (m, 9 H), 7.04 (s, 1 H), 6.96–6.94 (m, 2 H), 6.92 (s, 2 H), 6.88–6.86 (m, 2 H), 4.72–4.70 (m, 1 H), 3.77 (t, *J* = 7.5 Hz, 1 H), 3.14–3.06 (m, 1 H), 2.61–2.55 (m, 1 H), 2.49–2.42 (m, 7 H), 2.31 (s, 3 H), 2.30 (s, 3 H), 2.19–2.09 (m, 2 H), 1.93–1.87 (m, 1 H), 1.71–1.64 (m, 1 H). **^{13}C NMR (125 MHz, CDCl$_3$)** δ 144.6, 143.6, 143.4, 142.1, 138.9, 135.4, 135.2, 134.9, 132.1, 130.6, 129.8, 128.6, 128.5, 127.6, 127.4, 126.7, 126.41, 126.40, 124.5, 122.9, 122.7, 121.8, 119.2, 113.7, 51.7, 47.6, 41.6, 34.2, 23.0, 21.5, 20.9, 20.6. **HRMS** (ESI) m/z calcd. For $C_{41}H_{42}N_2NaO_4S_2$ $[M+Na]^+$: 713.2478, found: 713.2490.

The enantiomeric ratio was determined to be 93.0:7.0 by SFC analysis: IC-3 column, MeOH/CO_2 = 20:80, 1.0 mL/min, 210 nm, t_{major} = 33.4 min, t_{minor} = 37.7 min.

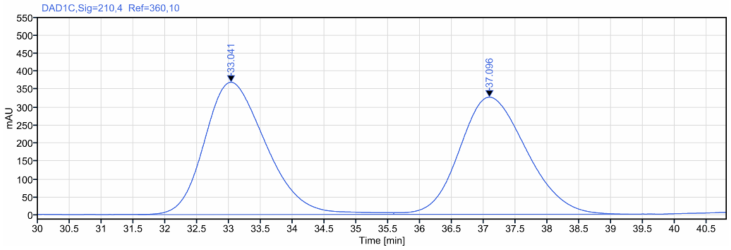

Signal: DAD1C,Sig=210,4 Ref=360,10

RT [min]	Type	Width [min]	Area	Height	Area%	Name
33.041	VV	4.01	24682.56	367.67	50.95	
37.096	VB	3.68	23760.28	325.49	49.05	
		Sum	48442.83			

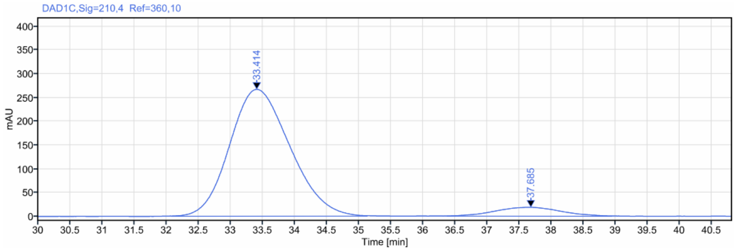

Signal: DAD1C,Sig=210,4 Ref=360,10

RT [min]	Type	Width [min]	Area	Height	Area%	Name
33.414	VV	3.05	17461.65	266.62	92.96	
37.685	VV	2.56	1321.93	18.76	7.04	
		Sum	18783.58			

Supplementary Fig. 13 SFC spectra of **5m**

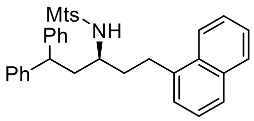

(*S*)-2,4,6-Trimethyl-*N*-(5-(naphthalen-1-yl)-1,1-diphenylpentan-3-yl)benzenesulfonamide (5n)

Following the **General Procedure C**, the title compound was isolated as white solid (51.0 mg, 93% yield). **^{1}H NMR (500 MHz, CDCl$_3$)** δ 7.85–7.82 (m, 1 H), 7.76–7.74 (m, 1 H), 7.67 (d, J = 8.5 Hz, 1 H), 7.48–7.43 (m, 2 H), 7.26–7.13 (m, 7 H), 7.01–6.97 (m, 4 H), 6.89 (d, J = 7.0 Hz, 1 H), 6.87 (s, 2 H), 4.55 (d, J = 10.0 Hz, 1 H), 3.86 (t, J = 7.5 Hz, 1 H), 3.20–3.13 (m, 1 H), 2.99–2.94 (m, 1 H), 2.86–2.80 (m, 1 H), 2.47 (s, 6 H), 2.27 (s, 3 H), 2.25–2.15 (m, 2 H), 2.01–1.94 (m, 1 H), 1.76–1.68 (m, 1 H). **^{13}C NMR (125 MHz, CDCl$_3$)** δ 143.8, 143.6, 142.0, 138.9, 137.1, 134.8, 133.9, 132.0, 131.5, 128.8, 128.6, 128.5, 127.7, 127.6, 126.7, 126.4, 125.84, 125.78, 125.5, 125.4, 123.4, 52.0, 47.6, 41.8, 35.8, 28.6, 23.0, 21.0. **HRMS** (ESI) m/z calcd. For $C_{36}H_{37}NNaO_2S$ [M+Na]$^+$: 570.2437, found: 570.2449.

The enantiomeric ratio was determined to be 94.4:5.6 by SFC analysis: IC-3 column, MeOH/CO_2 = 20:80, 1.0 mL/min, 210 nm, t_{major} = 11.2 min, t_{minor} = 14.9 min.

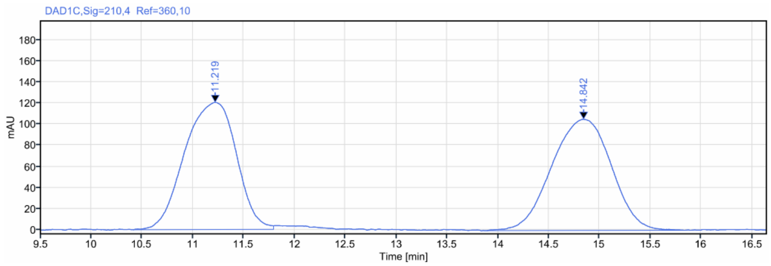

Signal: DAD1C,Sig=210,4 Ref=360,10

RT [min]	Type	Width [min]	Area	Height	Area%	Name
11.219	BV	1.38	4271.41	120.44	49.83	
14.842	BB	1.94	4300.88	105.27	50.17	
		Sum	8572.29			

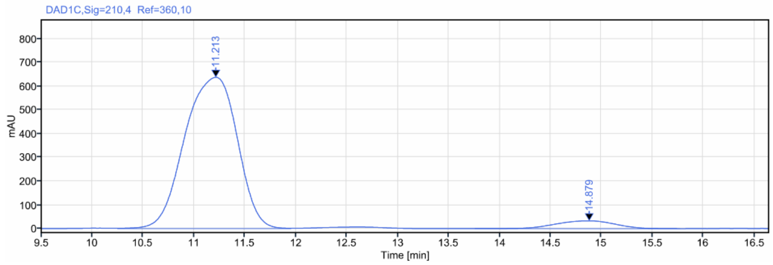

Signal: DAD1C,Sig=210,4 Ref=360,10

RT [min]	Type	Width [min]	Area	Height	Area%	Name
11.213	VV	1.70	22946.27	637.89	94.38	
14.879	BB	2.01	1366.51	33.14	5.62	
		Sum	24312.79			

Supplementary Fig. 14 SFC spectra of **5n**

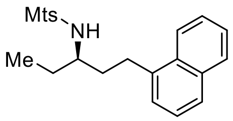

(*R*)-2,4,6-Trimethyl-*N*-(1-(naphthalen-1-yl)pentan-3-yl)benzenesulfonamide (5o)

Following the **General Procedure C**, the title compound was isolated as white solid (25.1 mg, 64% yield). **^{1}H NMR (500 MHz, CDCl$_3$)** δ 7.84–7.80 (m, 1 H), 7.78–7.77 (m, 1 H), 7.68 (d, *J* = 8.0 Hz, 1 H), 7.47–7.43 (m, 2 H), 7.33 (t, *J* = 7.5 Hz, 1 H), 7.11 (d, *J* = 7.0 Hz, 1 H), 6.90 (s, 2 H), 4.48 (d, *J* = 9.0 Hz, 1 H), 3.33–3.27 (m, 1 H), 3.02–2.96 (m, 1 H), 2.88–2.82 (m, 1 H), 2.63 (s, 6 H), 2.27 (s, 3 H), 1.87–1.80 (m, 1 H), 1.74–1.66 (m, 1 H), 1.64–1.57 (m, 1 H), 1.54–1.45 (m, 1 H), 0.85 (t, *J* = 7.0 Hz, 3 H). **^{13}C NMR (125 MHz, CDCl$_3$)** δ 141.9, 138.6, 137.5, 135.1, 133.8, 132.0, 131.6, 128.8, 126.7, 125.8, 125.6, 125.5, 125.4, 123.4, 55.2, 35.4, 28.8, 28.0, 23.1, 20.9, 9.7. **HRMS** (ESI) m/z calcd. For $C_{24}H_{29}NNaO_2S$ $[M+Na]^+$: 418.1811, found: 418.1815.

The enantiomeric ratio was determined to be 92.6:7.4 by SFC analysis: OJ-3 column, MeOH/CO_2 = 5:95, 1.0 mL/min, 210 nm, t_{major} = 21.1 min, t_{minor} = 22.2 min.

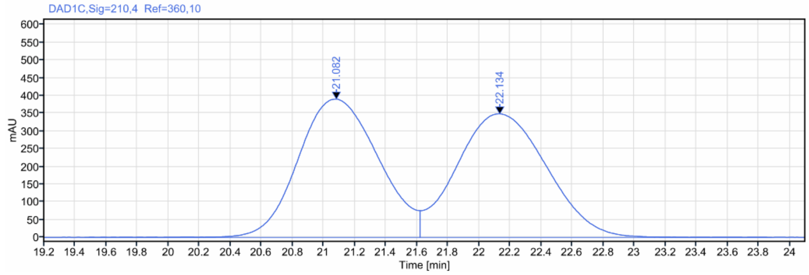

Signal: DAD1C,Sig=210,4 Ref=360,10

RT [min]	Type	Width [min]	Area	Height	Area%	Name
21.082	BV	2.16	14046.82	389.59	49.89	
22.134	VB	2.26	14106.04	347.79	50.11	
		Sum	28152.86			

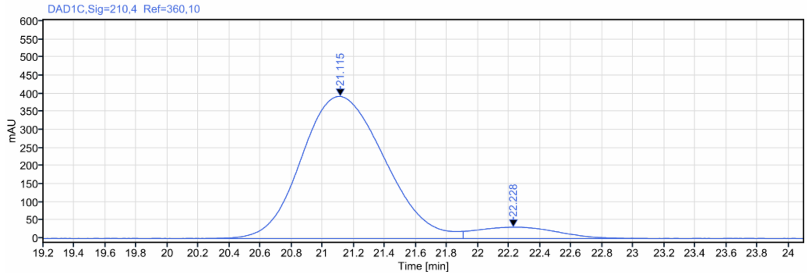

Signal: DAD1C,Sig=210,4 Ref=360,10

RT [min]	Type	Width [min]	Area	Height	Area%	Name
21.115	BV	2.02	14666.97	393.27	92.64	
22.228	VB	1.45	1165.55	31.62	7.36	
		Sum	15832.52			

Supplementary Fig. 15 SFC spectra of **5o**

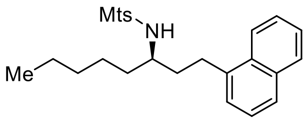

(*R*)-2,4,6-Trimethyl-*N*-(1-(naphthalen-1-yl)octan-3-yl)benzenesulfonamide (5p)

Following the **General Procedure C**, the title compound was isolated as white solid (34.6 mg, 79% yield). **^{1}H NMR (500 MHz, $CDCl_3$)** δ 7.84–7.78 (m, 2 H), 7.68 (d, *J* = 8.0 Hz, 1 H), 7.47–7.43 (m, 2 H), 7.33 (t, *J* = 7.5 Hz, 1 H), 7.13 (d, *J* = 7.0 Hz, 1 H), 6.90 (s, 2 H), 4.50 (d, *J* = 9.0 Hz, 1 H), 3.37–3.30 (m, 1 H), 3.05–2.99 (m, 1 H), 2.90–2.84 (m, 1 H), 2.63 (s, 6 H), 2.26 (s, 3 H), 1.88–1.81 (m, 1 H), 1.75–1.67 (m, 1 H), 1.54–1.47 (m, 1 H), 1.46–1.39 (m, 1 H), 1.25–1.08 (m, 6 H), 0.82 (t, *J* = 6.5 Hz, 3 H). **^{13}C NMR (125 MHz, $CDCl_3$)** δ 141.9, 138.6, 137.5, 135.1, 133.8, 131.9, 131.6, 128.7, 126.7, 125.8, 125.7, 125.5, 125.4, 123.5, 53.9, 36.0, 35.2, 31.5, 28.8, 24.9, 23.1, 22.4, 20.9, 13.9. **HRMS** (ESI) m/z calcd. For $C_{27}H_{35}NNaO_2S$ $[M+Na]^+$: 460.2281, found: 460.2281.

The enantiomeric ratio was determined to be 92.4:7.6 by SFC analysis: IC-3 column, $MeOH/CO_2$ = 15:85, 1.0 mL/min, 210 nm, t_{major} = 8.0 min, t_{minor} = 9.6 min.

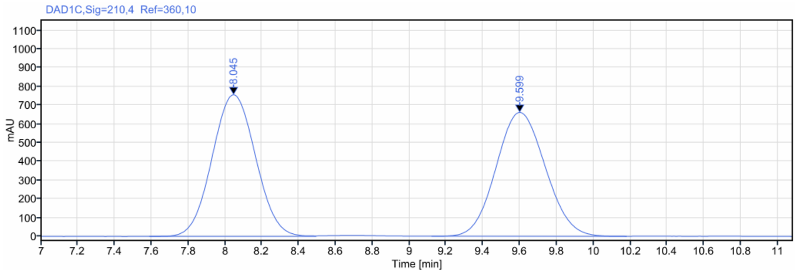

Signal: DAD1C,Sig=210,4 Ref=360,10

RT [min]	Type	Width [min]	Area	Height	Area%	Name
8.045	BV	0.90	12398.31	755.96	50.22	
9.599	VB	1.06	12289.69	661.20	49.78	
		Sum	24688.00			

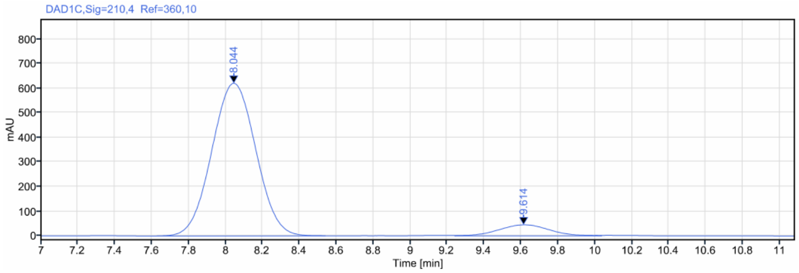

Signal: DAD1C,Sig=210,4 Ref=360,10

RT [min]	Type	Width [min]	Area	Height	Area%	Name
8.044	BV	0.95	10311.07	621.33	92.40	
9.614	VV	0.79	848.50	44.72	7.60	
		Sum	11159.57			

Supplementary Fig. 16 SFC spectra of **5p**

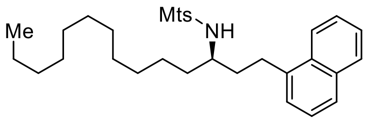

(*R*)-2,4,6-Trimethyl-*N*-(1-(naphthalen-1-yl)tetradecan-3-yl)benzenesulfonamide (5q)

Following the **General Procedure C**, but the reaction was conducted at 50 °C. The title compound was isolated as white solid (32.6 mg, 63% yield). **^{1}H NMR (500 MHz, CDCl$_3$)** δ 7.84–7.78 (m, 2 H), 7.68 (d, *J* = 8.0 Hz, 1 H), 7.47–7.43 (m, 2 H), 7.33 (dd, J_1 = 8.0 Hz, J_2 = 7.0 Hz, 1 H), 7.14 (d, *J* = 7.0 Hz, 1 H), 6.90 (s, 2 H), 4.47 (d, *J* = 9.0 Hz, 1 H), 3.37–3.31 (m, 1 H), 3.05–2.99 (m, 1 H), 2.90–2.84 (m, 1 H), 2.63 (s, 6 H), 2.27 (s, 3 H), 1.88–1.81 (m, 1 H), 1.75–1.68 (m, 1 H), 1.53–1.47 (m, 1 H), 1.45–1.38 (m, 1 H), 1.30–1.13 (m, 18 H), 0.90–0.87 (m, 3 H). **^{13}C NMR (125 MHz, CDCl$_3$)** δ 141.9, 138.6, 137.5, 135.1, 133.8, 132.0, 131.6, 128.7, 126.7, 125.8, 125.7, 125.5, 125.4, 123.5, 53.9, 36.0, 35.2, 31.9, 29.6, 29.5, 29.4, 29.35, 29.30, 28.8, 25.2, 23.1, 22.7, 20.9, 14.1. **HRMS** (ESI) m/z calcd. For $C_{33}H_{47}NNaO_2S$ [M+Na]$^+$: 544.3220, found: 544.3228.

The enantiomeric ratio was determined to be 93.5:6.5 by SFC analysis: IC-3 column, MeOH/CO_2 = 15:85, 1.0 mL/min, 210 nm, t_{major} = 9.6 min, t_{minor} = 12.0 min.

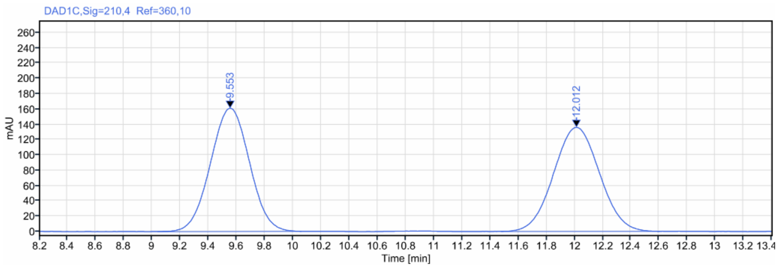

Signal: DAD1C,Sig=210,4 Ref=360,10

RT [min]	Type	Width [min]	Area	Height	Area%	Name
9.553	VV	1.02	3108.06	161.06	50.12	
12.012	VV	1.12	3093.11	135.98	49.88	
		Sum	6201.17			

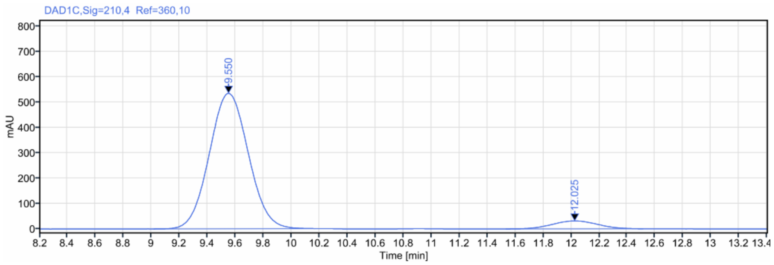

Signal: DAD1C,Sig=210,4 Ref=360,10

RT [min]	Type	Width [min]	Area	Height	Area%	Name
9.550	BV	1.15	10411.30	535.51	93.48	
12.025	VV	0.98	726.70	31.80	6.52	
		Sum	11137.99			

Supplementary Fig. 17 SFC spectra of **5q**

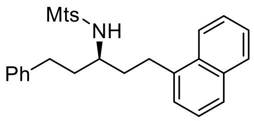

(*R*)-2,4,6-Trimethyl-*N*-(1-(naphthalen-1-yl)-5-phenylpentan-3-yl)benzenesulfonamide (5r)

Following the **General Procedure C**, the title compound was isolated as white solid (32.3 mg, 69% yield). **^{1}H NMR (500 MHz, $CDCl_3$)** δ 7.85–7.82 (m, 1 H), 7.81–7.76 (m, 1 H), 7.68 (d, J = 8.0 Hz, 1 H), 7.48–7.43 (m, 2 H), 7.30 (dd, J_1 = 8.5 Hz, J_2 = 7.5 Hz, 1 H), 7.23–7.19 (m, 2 H), 7.18–7.15 (m, 1 H), 7.04 (d, J = 7.0 Hz, 1 H), 6.96–6.94 (m, 2 H), 6.88 (s, 2 H), 4.56 (d, J = 9.5 Hz, 1 H), 3.37–3.30 (m, 1 H), 3.04–2.98 (m, 1 H), 2.91–2.85 (m, 1 H), 2.61–2.49 (m, 8 H), 2.27 (s, 3 H), 1.94–1.80 (m, 2 H), 1.77–1.69 (m, 2 H). **^{13}C NMR (125 MHz, $CDCl_3$)** δ 142.0, 141.0, 138.7, 137.2, 134.9, 133.8, 132.0, 131.6, 128.8, 128.4, 128.3, 126.8, 126.0, 125.9, 125.8, 125.5, 125.4, 123.4, 53.2, 37.0, 35.8, 31.7, 28.8, 23.1, 20.9. **HRMS** (ESI) m/z calcd. For $C_{30}H_{33}NNaO_2S$ $[M+Na]^+$: 494.2124, found: 494.2132.

The enantiomeric ratio was determined to be 93.0:7.0 by SFC analysis: IC-3 column, $MeOH/CO_2$ = 20:80, 1.0 mL/min, 210 nm, t_{major} = 9.4 min, t_{minor} = 10.3 min.

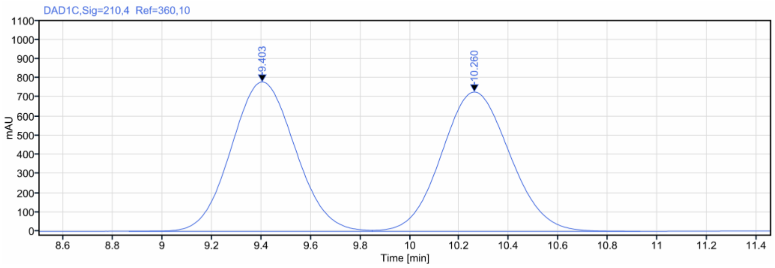

Signal: DAD1C,Sig=210,4 Ref=360,10

RT [min]	Type	Width [min]	Area	Height	Area%	Name
9.403	VV	0.98	14073.80	780.37	50.13	
10.260	VB	1.08	14001.48	728.10	49.87	
		Sum	28075.28			

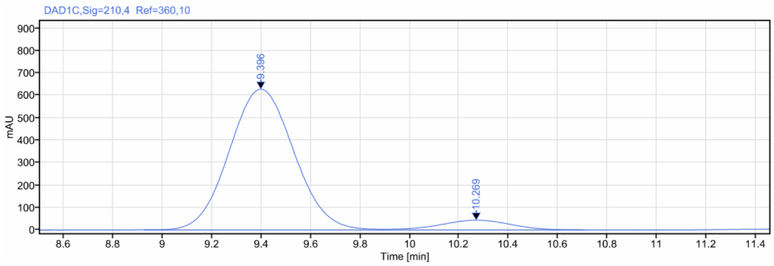

Signal: DAD1C,Sig=210,4 Ref=360,10

RT [min]	Type	Width [min]	Area	Height	Area%	Name
9.396	VV	0.97	11482.02	628.57	92.98	
10.269	VB	0.82	867.01	43.73	7.02	
		Sum	12349.03			

Supplementary Fig. 18 SFC spectra of **5r**

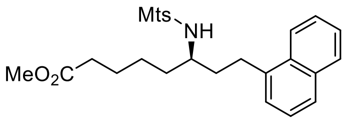

Methyl (*R*)-8-(naphthalen-1-yl)-6-((2,4,6-trimethylphenyl)sulfonamido)octanoate (5s)

Following the **General Procedure C**, the title compound was isolated as white solid (37.4 mg, 78% yield). **^{1}H NMR (500 MHz, CDCl$_3$)** δ 7.83–7.81 (m, 1 H), 7.77–7.76 (m, 1 H), 7.68 (d, J = 8.0 Hz, 1 H), 7.47–7.43 (m, 2 H), 7.33 (t, J = 7.5 Hz, 1 H), 7.11 (d, J = 7.0 Hz, 1 H), 6.90 (s, 2 H), 4.54 (d, J = 9.0 Hz, 1 H), 3.65 (s, 3 H), 3.37–3.31 (m, 1 H), 3.02–2.96 (m, 1 H), 2.88–2.82 (m, 1 H), 2.61 (s, 6 H), 2.27 (s, 3 H), 2.18 (t, J = 7.0 Hz, 2 H), 1.85–1.78 (m, 1 H), 1.73–1.66 (m, 1 H), 1.59–1.41 (m, 4 H), 1.35–1.18 (m, 2 H). **^{13}C NMR (125 MHz, CDCl$_3$)** δ 173.9, 142.0, 138.6, 137.3, 135.1, 133.8, 132.0, 131.6, 128.8, 126.7, 125.8, 125.7, 125.5, 125.4, 123.4, 53.7, 51.5, 36.0, 34.9, 33.7, 28.8, 24.8, 24.5, 23.1, 20.9. **HRMS** (ESI) m/z calcd. For $C_{28}H_{35}NNaO_4S$ $[M+Na]^+$: 504.2179, found: 504.2195.

The enantiomeric ratio was determined to be 92.4:7.6 by SFC analysis: IC-3 column, MeOH/CO_2 = 20:80, 1.0 mL/min, 210 nm, t_{major} = 9.9 min, t_{minor} = 10.5 min.

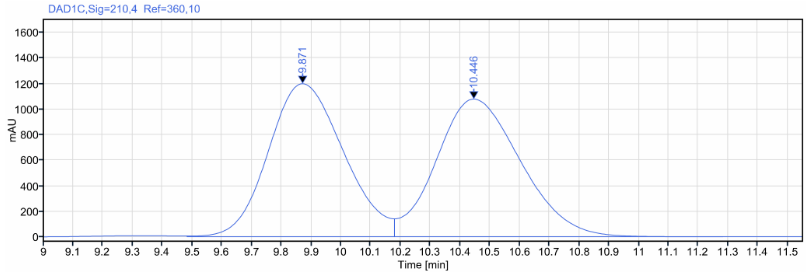

Signal: DAD1C,Sig=210,4 Ref=360,10

RT [min]	Type	Width [min]	Area	Height	Area%	Name
9.871	VV	0.70	21032.39	1198.88	49.72	
10.446	VB	1.10	21273.01	1078.41	50.28	
		Sum	42305.40			

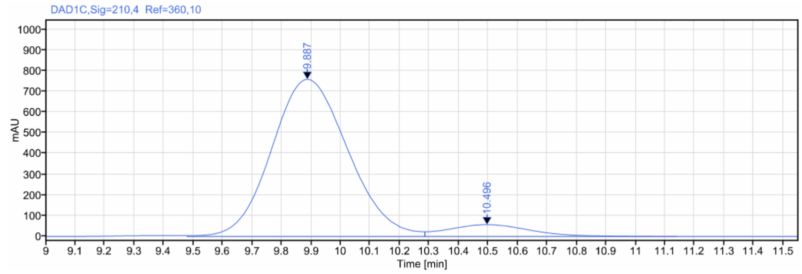

Signal: DAD1C,Sig=210,4 Ref=360,10

RT [min]	Type	Width [min]	Area	Height	Area%	Name
9.887	VV	0.81	13690.33	759.46	92.44	
10.496	VB	0.85	1119.60	57.40	7.56	
		Sum	14809.93			

Supplementary Fig. 19 SFC spectra of **5s**

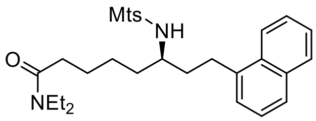

(*R*)-*N*,*N*-Diethyl-8-(naphthalen-1-yl)-6-((2,4,6-trimethylphenyl)sulfonamido)octanamide (5t)

Following the **General Procedure C**, the title compound was isolated as light yellow solid (26.6 mg, 51% yield). **^{1}H NMR (500 MHz, CDCl$_3$)** δ 7.83–7.80 (m, 1 H), 7.74–7.72 (m, 1 H), 7.67 (d, *J* = 8.5 Hz, 1 H), 7.47–7.41 (m, 2 H), 7.32 (t, *J* = 7.0 Hz, 1 H), 7.09 (d, *J* = 7.0 Hz, 1 H), 6.88 (s, 2 H), 4.66 (d, *J* = 9.0 Hz, 1 H), 3.41–3.32 (m, 3 H), 3.27 (q, *J* = 7.0 Hz, 2 H), 2.98–2.92 (m, 1 H), 2.86–2.80 (m, 1 H), 2.61 (s, 6 H), 2.26 (s, 3 H), 2.24–2.20 (m, 2 H), 1.84–1.77 (m, 1 H), 1.71–1.64 (m, 2 H), 1.63–1.54 (m, 2 H), 1.53–1.49 (m, 1 H), 1.43–1.37 (m, 1 H), 1.34–1.24 (m, 1 H), 1.15 (t, *J* = 7.0 Hz, 3 H), 1.10 (t, *J* = 7.0 Hz, 3 H). **^{13}C NMR (125 MHz, CDCl$_3$)** δ 171.9, 141.8, 138.5, 137.4, 135.2, 133.8, 131.9, 131.6, 128.7, 126.7, 125.7, 125.6, 125.45, 125.40, 123.4, 53.8, 41.9, 40.1, 36.0, 35.1, 32.8, 28.8, 25.2, 25.1, 23.1, 20.9, 14.3, 13.1. **HRMS** (ESI) m/z calcd. For $C_{31}H_{42}N_2NaO_3S$ $[M+Na]^+$: 545.2808, found: 545.2817.

The enantiomeric ratio was determined to be 92.1:7.9 by SFC analysis: OJ-3 column, MeOH/CO_2 = 15:85, 1.0 mL/min, 210 nm, t_{major} = 3.6 min, t_{minor} = 3.2 min.

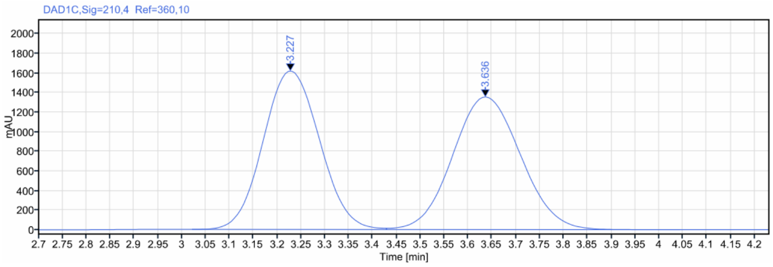

Signal: DAD1C,Sig=210,4 Ref=360,10

RT [min]	Type	Width [min]	Area	Height	Area%	Name
3.227	BV	0.41	13248.80	1615.67	49.69	
3.636	VB	0.61	13412.07	1352.11	50.31	
		Sum	26660.86			

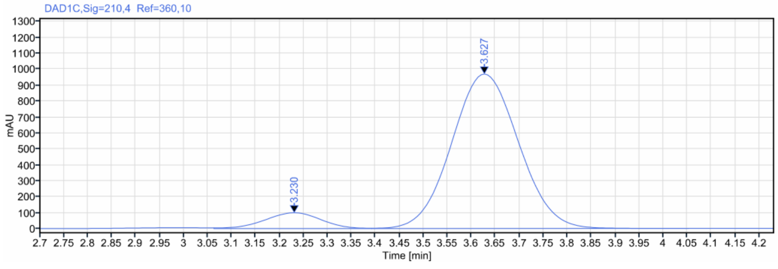

Signal: DAD1C,Sig=210,4 Ref=360,10

RT [min]	Type	Width [min]	Area	Height	Area%	Name
3.230	VV	0.33	809.52	99.33	7.90	
3.627	VB	0.66	9441.90	970.01	92.10	
		Sum	10251.43			

Supplementary Fig. 20 SFC spectra of **5t**

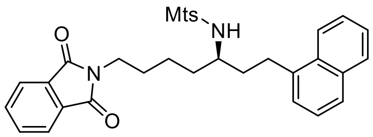

(*R*)-*N*-(7-(1,3-Dioxoisoindolin-2-yl)-1-(naphthalen-1-yl)heptan-3-yl)-2,4,6-trimethylbenzenesulfonamide (5u)

Following the **General Procedure C**, the title compound was isolated as yellow solid (37.9 mg, 67% yield). **^{1}H NMR (500 MHz, $CDCl_3$)** δ 7.85–7.81 (m, 3 H), 7.76–7.73 (m, 1 H), 7.72–7.67 (m, 3 H), 7.47–7.41 (m, 2 H), 7.34–7.31 (m, 1 H), 7.11 (d, J = 7.0 Hz, 1 H), 6.86 (s, 2 H), 4.59 (d, J = 8.5 Hz, 1 H), 3.60 (t, J = 7.0 Hz, 2 H), 3.38–3.32 (m, 1 H), 3.01–2.95 (m, 1 H), 2.89–2.83 (m, 1 H), 2.59 (s, 6 H), 2.24 (s, 3 H), 1.86–1.79 (m, 1 H), 1.75–1.67 (m, 1 H), 1.62–1.49 (m, 4 H), 1.36–1.22 (m, 2 H). **^{13}C NMR (125 MHz, $CDCl_3$)** δ 168.5, 141.9, 138.5, 137.4, 135.1, 133.9, 133.8, 132.1, 131.9, 131.6, 128.7, 126.7, 125.8, 125.6, 125.5, 125.4, 123.4, 123.2, 53.8, 37.3, 36.0, 34.4, 28.8, 28.2, 23.1, 22.3, 20.9. **HRMS** (ESI) m/z calcd. For $C_{34}H_{36}N_2NaO_4S$ $[M+Na]^+$: 591.2288, found: 591.2305.

The enantiomeric ratio was determined to be 93.8:6.2 by SFC analysis: IC-3 column, $MeOH/CO_2$ = 15:85, 1.0 mL/min, 210 nm, t_{major} = 64.4 min, t_{minor} = 67.2 min.

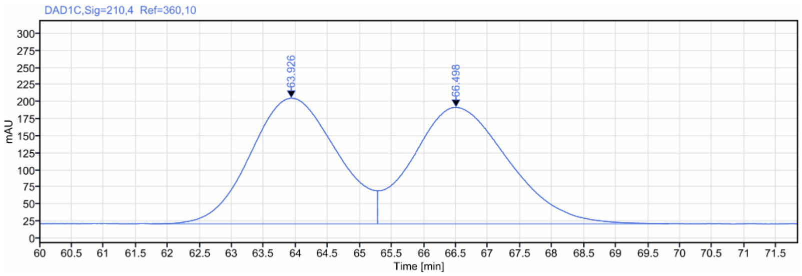

Signal: DAD1C,Sig=210,4 Ref=360,10

RT [min]	Type	Width [min]	Area	Height	Area%	Name
63.926	BV	3.52	17244.65	183.86	49.23	
66.498	VV	4.54	17784.69	170.37	50.77	
		Sum	35029.34			

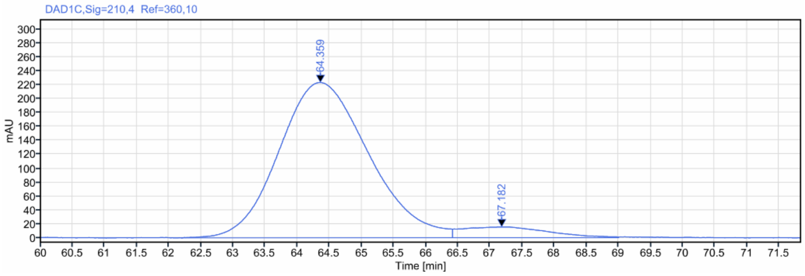

Signal: DAD1C,Sig=210,4 Ref=360,10

RT [min]	Type	Width [min]	Area	Height	Area%	Name
64.359	BV	4.22	21715.90	222.79	93.80	
67.182	VV	2.59	1434.83	15.39	6.20	
		Sum	23150.74			

Supplementary Fig. 21 SFC spectra of **5u**

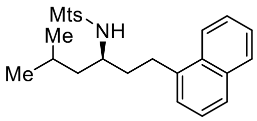

(*S*)-2,4,6-Trimethyl-*N*-(5-methyl-1-(naphthalen-1-yl)hexan-3-yl)benzenesulfonamide (5v)

Following the **General Procedure C**, but the reaction was conducted at 50 °C. The title compound was isolated as white solid (21.3 mg, 50% yield). **^{1}H NMR (500 MHz, $CDCl_3$)** δ 7.84–7.81 (m, 1 H), 7.78–7.75 (m, 1 H), 7.68 (d, J = 8.0 Hz, 1 H), 7.47–7.42 (m, 2 H), 7.33 (dd, J_1 = 8.0 Hz, J_2 = 7.0 Hz, 1 H), 7.11 (d, J = 7.0 Hz, 1 H), 6.89 (s, 2 H), 4.43 (d, J = 9.0 Hz, 1 H), 3.42–3.35 (m, 1 H), 3.02–2.96 (m, 1 H), 2.90–2.84 (m, 1 H), 2.61 (s, 6 H), 2.26 (s, 3 H), 1.86–1.79 (m, 1 H), 1.70–1.59 (m, 2 H), 1.41–1.29 (m, 2 H), 0.79 (d, J = 7.0 Hz, 3 H), 0.7 (d, J = 6.5 Hz, 3 H). **^{13}C NMR (125 MHz, $CDCl_3$)** δ 141.9, 138.6, 137.5, 135.1, 133.8, 131.9, 131.6, 128.7, 126.7, 125.8, 125.7, 125.44, 125.42, 123.5, 52.1, 44.9, 36.3, 28.7, 24.6, 23.1, 22.6, 22.2, 20.9. **HRMS** (ESI) m/z calcd. For $C_{26}H_{33}NNaO_2S$ $[M+Na]^+$: 446.2124, found: 446.2132.

The enantiomeric ratio was determined to be 95.5:4.5 by SFC analysis: IC-3 column, MeOH/CO_2 = 20:80, 1.0 mL/min, 210 nm, t_{major} = 4.3 min, t_{minor} = 5.0 min.

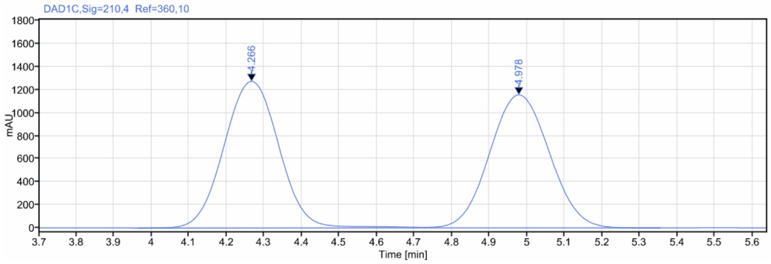

Signal: DAD1C,Sig=210,4 Ref=360,10

RT [min]	Type	Width [min]	Area	Height	Area%	Name
4.266	BV	0.76	12995.96	1274.54	50.38	
4.978	VB	0.63	12799.00	1156.12	49.62	
		Sum	25794.96			

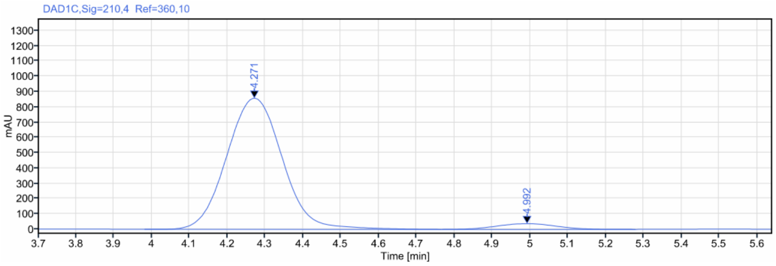

Signal: DAD1C,Sig=210,4 Ref=360,10

RT [min]	Type	Width [min]	Area	Height	Area%	Name
4.271	BB	0.78	8768.52	857.61	95.54	
4.992	BB	0.51	409.69	37.64	4.46	
		Sum	9178.21			

Supplementary Fig. 22 SFC spectra of **5v**

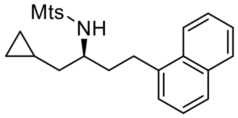

(*S*)-*N*-(1-Cyclopropyl-4-(naphthalen-1-yl)butan-2-yl)-2,4,6-trimethylbenzenesulfonamide (5w)

Following the **General Procedure C**, but the reaction was conducted at 50 °C. The title compound was isolated as white solid (24.7 mg, 59% yield). **^{1}H NMR (500 MHz, $CDCl_3$)** δ 7.84–7.79 (m, 2 H), 7.68 (d, *J* = 8.0 Hz, 1 H), 7.47–7.44 (m, 2 H), 7.33 (t, *J* = 7.0 Hz, 1 H), 7.12 (d, *J* = 7.0 Hz, 1 H), 6.91 (s, 2 H), 4.60 (d, *J* = 9.0 Hz, 1 H), 3.46–3.39 (m, 1 H), 3.06–2.99 (m, 1 H), 2.87–2.81 (m, 1 H), 2.64 (s, 6 H), 2.27 (s, 3 H), 2.01–1.93 (m, 1 H), 1.82–1.74 (m, 1 H), 1.44 (t, *J* = 6.5 Hz, 2 H), 0.62–0.54 (m, 1 H), 0.43–0.34 (m, 2 H), 0.03– -0.04 (m, 2 H). **^{13}C NMR (125 MHz, $CDCl_3$)** δ 142.0, 138.7, 137.5, 134.9, 133.8, 131.9, 131.6, 128.8, 126.7, 125.8, 125.6, 125.5, 125.4, 123.5, 54.6, 40.2, 35.7, 28.9, 23.2, 20.9, 7.1, 4.6, 4.2. **HRMS** (ESI) m/z calcd. For $C_{26}H_{31}NNaO_2S$ $[M+Na]^+$: 444.1968, found: 444.1978.

The enantiomeric ratio was determined to be 93.2:6.8 by SFC analysis: IC-3 column, MeOH/CO_2 = 20:80, 1.0 mL/min, 210 nm, t_{major} = 6.1 min, t_{minor} = 6.9 min.

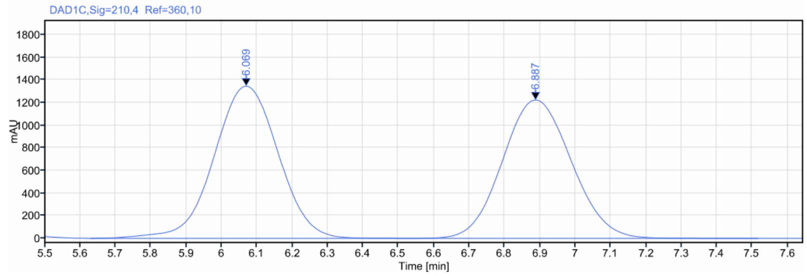

Signal: DAD1C,Sig=210,4 Ref=360,10

RT [min]	Type	Width [min]	Area	Height	Area%	Name
6.069	BB	0.90	16969.90	1344.19	50.59	
6.887	BV	0.99	16573.39	1222.46	49.41	
		Sum	33543.29			

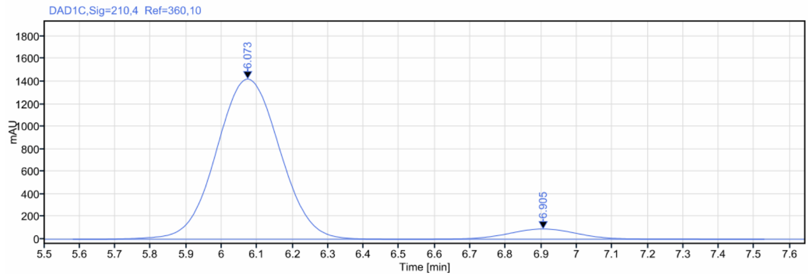

Signal: DAD1C,Sig=210,4 Ref=360,10

RT [min]	Type	Width [min]	Area	Height	Area%	Name
6.073	BV	0.93	17705.24	1421.59	93.19	
6.905	VB	1.02	1293.47	92.02	6.81	
		Sum	18998.71			

Supplementary Fig. 23 SFC spectra of **5w**

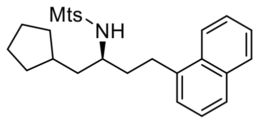

(*S*)-*N*-(1-Cyclopentyl-4-(naphthalen-1-yl)butan-2-yl)-2,4,6-trimethylbenzenesulfonamide (5x)

Following the **General Procedure C**, but the reaction was conducted at 50 °C. The title compound was isolated as white solid (33.1 mg, 74% yield). **^{1}H NMR (500 MHz, CDCl$_3$)** δ 7.84–7.77 (m, 2 H), 7.68 (d, *J* = 8.0 Hz, 1 H), 7.47–7.42 (m, 2 H), 7.33 (dd, J_1 = 8.0 Hz, J_2 = 7.0 Hz, 1 H), 7.12 (d, *J* = 6.5 Hz, 1 H), 6.90 (s, 2 H), 4.47 (d, *J* = 9.0 Hz, 1 H), 3.36–3.29 (m, 1 H), 3.04–2.98 (m, 1 H), 2.90–2.84 (m, 1 H), 2.61 (s, 6 H), 2.26 (s, 3 H), 1.90–1.83 (m, 1 H), 1.75–1.67 (m, 2 H), 1.66–1.60 (m, 1 H), 1.56–1.46 (m, 5 H), 1.45–1.35 (m, 2 H). **^{13}C NMR (125 MHz, CDCl$_3$)** δ 141.9, 138.6, 137.6, 135.0, 133.8, 131.9, 131.6, 128.7, 126.7, 125.75, 125.68, 125.44, 125.41, 123.5, 53.4, 42.0, 36.5, 36.4, 32.59, 32.58, 28.7, 24.94, 24.90, 23.1, 20.9. **HRMS** (ESI) m/z calcd. For $C_{28}H_{35}NNaO_2S$ [M+Na]$^+$: 472.2281, found: 472.2284.

The enantiomeric ratio was determined to be 95.3:4.7 by SFC analysis: IC-3 column, MeOH/CO_2 = 20:80, 1.0 mL/min, 210 nm, t_{major} = 6.7 min, t_{minor} = 8.1 min.

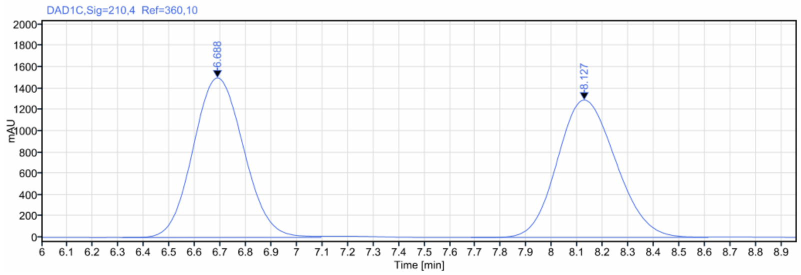

Signal: DAD1C,Sig=210,4 Ref=360,10

RT [min]	Type	Width [min]	Area	Height	Area%	Name
6.688	BV	0.78	20661.08	1497.75	50.14	
8.127	BV	0.93	20548.64	1290.27	49.86	
		Sum	41209.72			

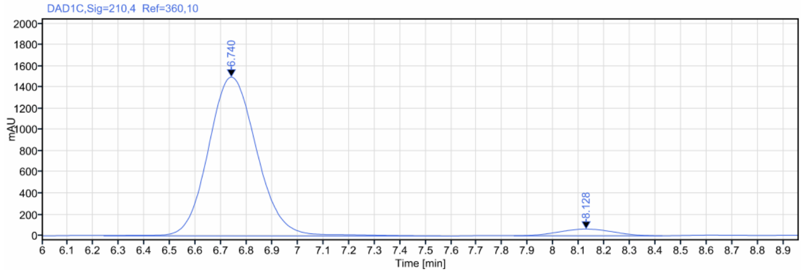

Signal: DAD1C,Sig=210,4 Ref=360,10

RT [min]	Type	Width [min]	Area	Height	Area%	Name
6.740	VB	1.35	19781.94	1499.32	95.31	
8.128	VB	0.58	972.56	64.87	4.69	
		Sum	20754.49			

Supplementary Fig. 24 SFC spectra of **5x**

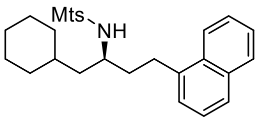

(*S*)-*N*-(1-Cyclohexyl-4-(naphthalen-1-yl)butan-2-yl)-2,4,6-trimethylbenzenesulfonamide (5y)

Following the **General Procedure C**, but the reaction was conducted at 50 °C. The title compound was isolated as white solid (34.2 mg, 74% yield). **^{1}H NMR (500 MHz, CDCl$_3$)** δ 7.84–7.81 (m, 2 H), 7.68 (d, *J* = 8.5 Hz, 1 H), 7.47–7.44 (m, 2 H), 7.34 (t, *J* = 8.0 Hz, 1 H), 7.14 (d, *J* = 7.0 Hz, 1 H), 6.90 (s, 2 H), 4.43 (d, *J* = 8.5 Hz, 1 H), 3.42–3.35 (m, 1 H), 3.05–2.99 (m, 1 H), 2.94–2.88 (m, 1 H), 2.61 (s, 6 H), 2.26 (s, 3 H), 1.89–1.82 (m, 1 H), 1.77–1.70 (m, 1 H), 1.61–1.53 (m, 3 H), 1.48–1.42 (m, 2 H), 1.39–1.27 (m, 2 H), 1.18–1.09 (m, 1 H), 1.08–1.00 (m, 2 H), 0.99–0.90 (m, 1 H), 0.83–0.75 (m, 1 H), 0.74–0.66 (m, 1 H). **^{13}C NMR (125 MHz, CDCl$_3$)** δ 141.9, 138.6, 137.6, 135.0, 133.8, 132.0, 131.6, 128.7, 126.7, 125.8, 125.44, 125.42, 123.5, 51.4, 43.4, 36.7, 33.9, 33.5, 32.9, 28.8, 26.4, 26.2, 25.9, 23.1, 20.9. **HRMS** (ESI) m/z calcd. For $C_{29}H_{37}NNaO_2S$ $[M+Na]^+$: 486.2437, found: 486.2442.

The enantiomeric ratio was determined to be 95.3:4.7 by SFC analysis: IC-3 column, MeOH/CO_2 = 20:80, 1.0 mL/min, 210 nm, t_{major} = 6.8 min, t_{minor} = 9.1 min.

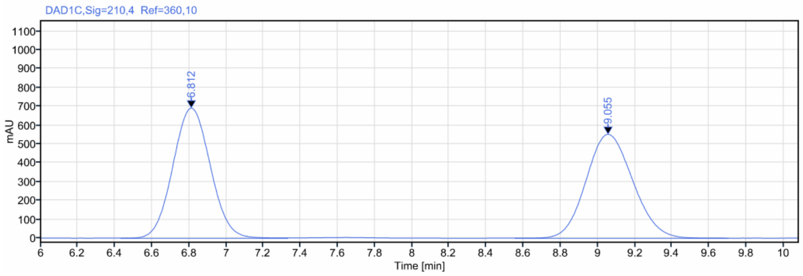

Signal: DAD1C,Sig=210,4 Ref=360,10

RT [min]	Type	Width [min]	Area	Height	Area%	Name
6.812	VV	0.90	9710.53	691.37	50.08	
9.055	VB	1.15	9678.66	552.02	49.92	
		Sum	19389.19			

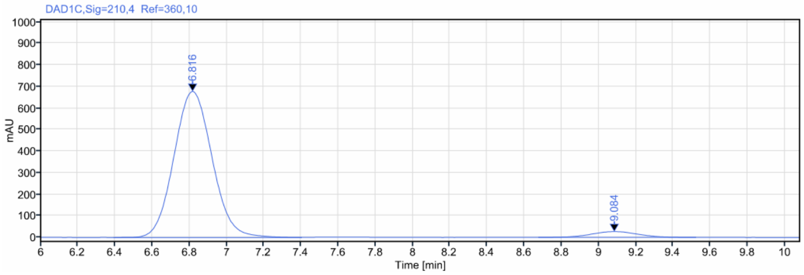

Signal: DAD1C,Sig=210,4 Ref=360,10

RT [min]	Type	Width [min]	Area	Height	Area%	Name
6.816	BV	1.01	9668.55	678.11	95.29	
9.084	VV	0.85	478.27	27.11	4.71	
		Sum	10146.82			

Supplementary Fig. 25 SFC spectra of **5y**

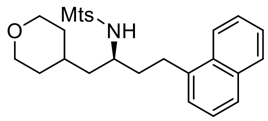

(*S*)-2,4,6-Trimethyl-*N*-(4-(naphthalen-1-yl)-1-(tetrahydro-2*H*-pyran-4-yl)butan-2-yl)benzenesulfonamide (5z)

Following the **General Procedure C**, the title compound was isolated as white solid (41.8 mg, 90% yield). **^{1}H NMR (500 MHz, $CDCl_3$)** δ 7.84–7.82 (m, 1 H), 7.79–7.77 (m, 1 H), 7.69 (d, *J* = 8.0 Hz, 1 H), 7.48–7.42 (m, 2 H), 7.34 (t, *J* = 7.0 Hz, 1 H), 7.13 (d, *J* = 7.0 Hz, 1 H), 6.89 (s, 2 H), 4.53 (d, *J* = 9.5 Hz, 1 H), 3.86–3.78 (m, 2 H), 3.41–3.34 (m, 1 H), 3.21–3.16 (m, 1 H), 3.09–3.04 (m, 1 H), 3.03–2.98 (m, 1 H), 2.95–2.89 (m, 1 H), 2.58 (s, 6 H), 2.27 (s, 3 H), 1.87–1.72 (m, 2 H), 1.46–1.39 (m, 2 H), 1.37–1.32 (m, 3 H), 1.22–1.14 (m, 1 H), 1.10–1.02 (m, 2 H). **^{13}C NMR (125 MHz, $CDCl_3$)** δ 142.0, 138.6, 137.2, 135.0, 133.8, 132.0, 131.5, 128.8, 126.8, 125.84, 125.81, 125.5, 125.4, 123.4, 67.8, 67.7, 50.7, 42.9, 36.9, 33.2, 32.5, 31.3, 28.9, 23.1, 20.9. **HRMS** (ESI) m/z calcd. For $C_{28}H_{35}NNaO_3S$ $[M+Na]^+$: 488.2230, found: 488.2236.

The enantiomeric ratio was determined to be 94.5:5.5 by SFC analysis: IC-3 column, MeOH/CO_2 = 20:80, 1.0 mL/min, 210 nm, t_{major} = 11.7 min, t_{minor} = 12.9 min.

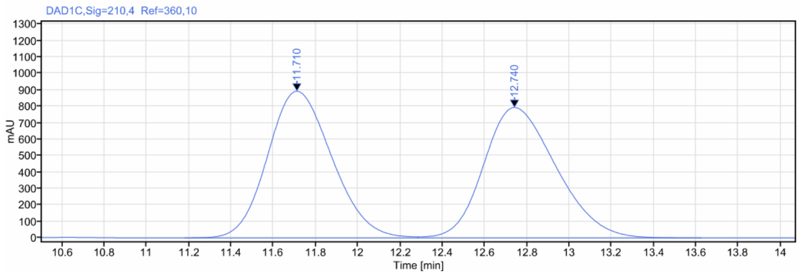

Signal: DAD1C,Sig=210,4 Ref=360,10

RT [min]	Type	Width [min]	Area	Height	Area%	Name
11.710	VV	1.10	18976.63	893.71	50.04	
12.740	VV	1.34	18946.36	794.25	49.96	
		Sum	37922.99			

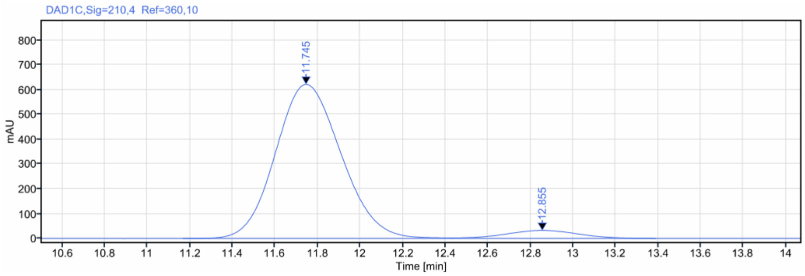

Signal: DAD1C,Sig=210,4 Ref=360,10

RT [min]	Type	Width [min]	Area	Height	Area%	Name
11.745	BV	1.27	13346.54	622.02	94.49	
12.855	VV	0.96	778.49	32.59	5.51	
		Sum	14125.02			

Supplementary Fig. 26 SFC spectra of **5z**

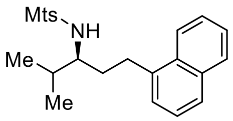

(*S*)-2,4,6-Trimethyl-*N*-(4-methyl-1-(naphthalen-1-yl)pentan-3-yl)benzenesulfonamide (5aa)

Following the **General Procedure C**, but the reaction was conducted at 80 °C. The title compound was isolated as white solid (23.7 mg, 58% yield). **^{1}H NMR (500 MHz, $CDCl_3$)** δ 7.85–7.81 (m, 1 H), 7.76–7.74 (m, 1 H), 7.69 (d, J = 8.0 Hz, 1 H), 7.48–7.43 (m, 2 H), 7.34 (dd, J_1 = 8.0 Hz, J_2 = 7.0 Hz, 1 H), 7.13 (d, J = 7.0 Hz, 1 H), 6.91 (s, 2 H), 4.50 (d, J = 9.0 Hz, 1 H), 3.29–3.23 (m, 1 H), 3.01–2.95 (m, 1 H), 2.85–2.77 (m, 1 H), 2.64 (s, 6 H), 2.27 (s, 3 H), 1.91–1.81 (m, 2 H), 1.67–1.60 (m, 1 H), 0.83 (t, J = 6.5 Hz, 6 H). **^{13}C NMR (125 MHz, $CDCl_3$)** δ 141.8, 138.5, 137.6, 135.3, 133.8, 132.0, 131.6, 128.8, 126.7, 125.8, 125.6, 125.5, 125.4, 123.4, 59.2, 32.9, 31.5, 29.3, 23.2, 20.9, 18.2, 17.7. **HRMS** (ESI) m/z calcd. For $C_{25}H_{31}NNaO_2S$ $[M+Na]^+$: 432.1968, found: 432.1977.

The enantiomeric ratio was determined to be 91.9:8.1 by SFC analysis: IC-3 column, MeOH/CO_2 = 15:85, 1.0 mL/min, 210 nm, t_{major} = 7.1 min, t_{minor} = 8.0 min.

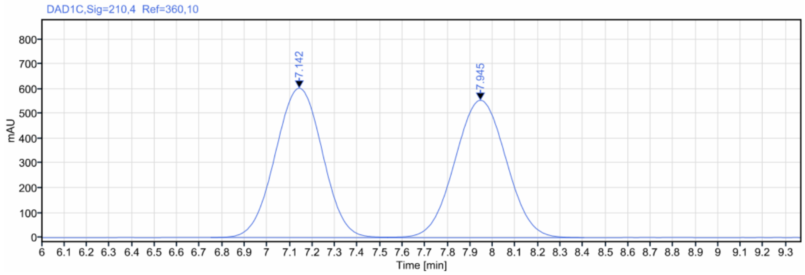

Signal: DAD1C,Sig=210,4 Ref=360,10

RT [min]	Type	Width [min]	Area	Height	Area%	Name
7.142	VV	0.79	8803.50	603.55	49.97	
7.945	VB	0.87	8813.30	554.70	50.03	
		Sum	17616.81			

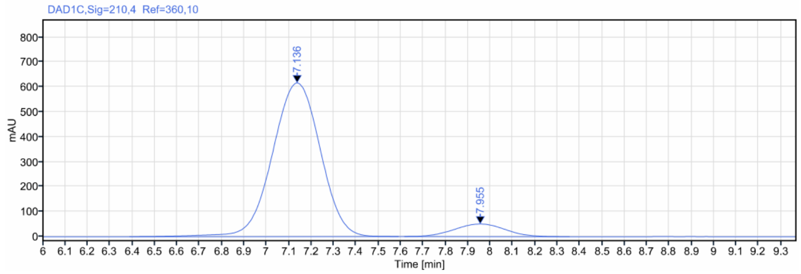

Signal: DAD1C,Sig=210,4 Ref=360,10

RT [min]	Type	Width [min]	Area	Height	Area%	Name
7.136	VV	1.20	9177.66	616.31	91.92	
7.955	BB	0.74	807.23	50.85	8.08	
		Sum	9984.89			

Supplementary Fig. 27 SFC spectra of **5aa**

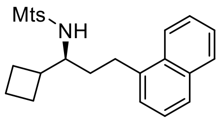

(*S*)-*N*-(1-Cyclobutyl-3-(naphthalen-1-yl)propyl)-2,4,6-trimethylbenzenesulfonamide (5ab)

Following the **General Procedure C**, but the reaction was conducted at 50 °C. The title compound was isolated as white solid (39.6 mg, 94% yield). **^{1}H NMR (500 MHz, CDCl$_3$)** δ 7.83–7.81 (m, 1 H), 7.77–7.75 (m, 1 H), 7.67 (d, *J* = 8.5 Hz, 1 H), 7.46–7.42 (m, 2 H), 7.33 (t, *J* = 7.5 Hz, 1 H), 7.12 (d, *J* = 7.0 Hz, 1 H), 6.92 (s, 2 H), 4.46 (d, *J* = 8.5 Hz, 1 H), 3.35–3.29 (m, 1 H), 2.99–2.93 (m, 1 H), 2.87–2.79 (m, 1 H), 2.66 (s, 6 H), 2.47–2.38 (m, 1 H), 2.27 (s, 3 H), 1.98–1.90 (m, 1 H), 1.89–1.71 (m, 3 H), 1.76–1.66 (m, 2 H), 1.64–1.54 (m, 2 H). **^{13}C NMR (125 MHz, CDCl$_3$)** δ 141.9, 138.5, 137.8, 135.4, 133.8, 132.0, 131.6, 128.7, 126.6, 125.7, 125.53, 125.49, 125.4, 123.5, 58.9, 39.9, 33.7, 28.4, 25.6, 25.3, 23.2, 20.9, 17.4. **HRMS** (ESI) m/z calcd. For $C_{26}H_{31}NNaO_2S$ [M+Na]$^+$: 444.1968, found: 444.1981.

The enantiomeric ratio was determined to be 93.1:6.9 by SFC analysis: IC-3 column, MeOH/CO_2 = 15:85, 1.0 mL/min, 210 nm, t_{major} = 10.4 min, t_{minor} = 11.0 min.

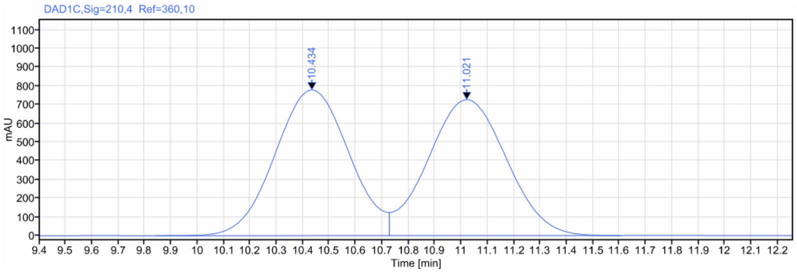

Signal: DAD1C,Sig=210,4 Ref=360,10

RT [min]	Type	Width [min]	Area	Height	Area%	Name
10.434	BV	0.89	15191.13	778.75	50.25	
11.021	VB	0.88	15038.86	726.37	49.75	
		Sum	30229.99			

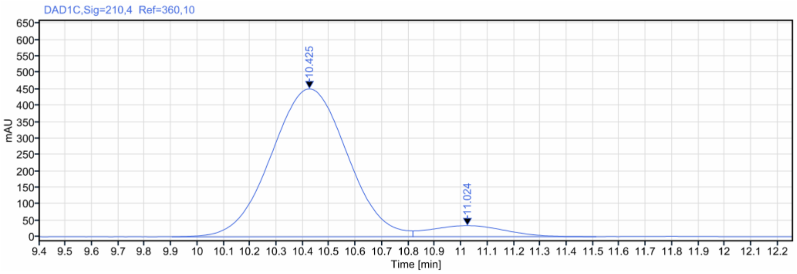

Signal: DAD1C,Sig=210,4 Ref=360,10

RT [min]	Type	Width [min]	Area	Height	Area%	Name
10.425	VV	0.91	9029.45	451.01	93.11	
11.024	VB	0.69	668.62	33.86	6.89	
		Sum	9698.07			

Supplementary Fig. 28 SFC spectra of **5ab**

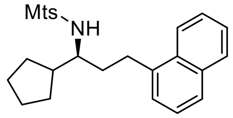

(*S*)-*N*-(1-Cyclopentyl-3-(naphthalen-1-yl)propyl)-2,4,6-trimethylbenzenesulfonamide (5ac)

Following the **General Procedure C**, but the reaction was conducted at 80 °C. The title compound was isolated as light yellow solid (19.9 mg, 46% yield). **^{1}H NMR (500 MHz, CDCl$_3$)** δ 7.84–7.81 (m, 1 H), 7.74–7.72 (m, 1 H), 7.68 (d, *J* = 8.0 Hz, 1 H), 7.47–7.42 (m, 2 H), 7.33 (t, *J* = 7.5 Hz, 1 H), 7.10 (d, *J* = 7.0 Hz, 1 H), 6.91 (s, 2 H), 4.50 (d, *J* = 8.5 Hz, 1 H), 3.39–3.33 (m, 1 H), 2.98–2.91 (m, 1 H), 2.88–2.82 (m, 1 H), 2.65 (s, 6 H), 2.26 (s, 3 H), 2.12–2.03 (m, 1 H), 1.93–1.86 (m, 1 H), 1.75–1.65 (m, 3 H), 1.63–1.49 (m, 4 H), 1.29–1.20 (m, 1 H), 1.19–1.12 (m, 1 H). **^{13}C NMR (125 MHz, CDCl$_3$)** δ 141.8, 138.4, 137.8, 135.5, 133.8, 132.0, 131.6, 128.7, 126.6, 125.7, 125.54, 125.48, 125.42, 123.4, 58.1, 44.1, 35.1, 29.2, 28.9, 28.6, 25.5, 25.2, 23.2, 20.9. **HRMS** (ESI) m/z calcd. For $C_{27}H_{33}NNaO_2S$ [M+Na]$^+$: 458.2124, found: 458.2141.

The enantiomeric ratio was determined to be 94.1:5.9 by SFC analysis: IC-3 column, MeOH/CO_2 = 20:80, 1.0 mL/min, 210 nm, t_{major} = 6.8 min, t_{minor} = 7.9 min.

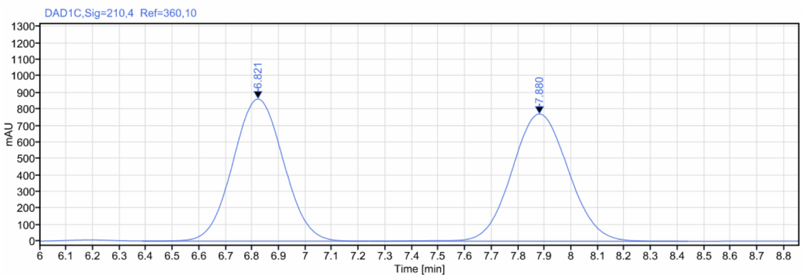

Signal: DAD1C,Sig=210,4 Ref=360,10

RT [min]	Type	Width [min]	Area	Height	Area%	Name
6.821	VB	0.85	11437.36	862.32	49.96	
7.880	BV	1.20	11457.53	772.04	50.04	
		Sum	22894.89			

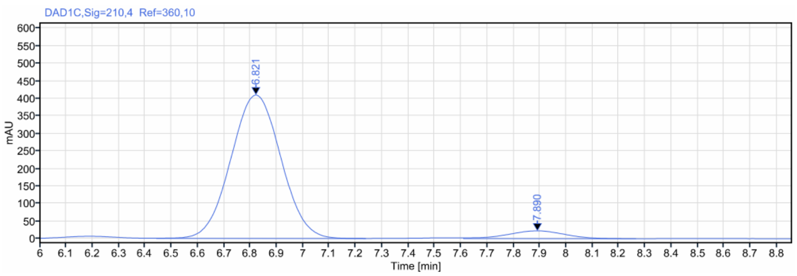

Signal: DAD1C,Sig=210,4 Ref=360,10

RT [min]	Type	Width [min]	Area	Height	Area%	Name
6.821	VB	0.80	5421.52	409.71	94.15	
7.890	VB	0.65	337.17	22.40	5.85	
		Sum	5758.69			

Supplementary Fig. 29 SFC spectra of **5ac**

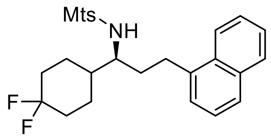

(*S*)-*N*-(1-(4,4-Difluorocyclohexyl)-3-(naphthalen-1-yl)propyl)-2,4,6-trimethylbenzenesulfonamide (5ad)

Following the **General Procedure C**, but (*R*)-BI-DIME (12 mol%) was used and the reaction was conducted at 80 ºC. The title compound was isolated as white solid (24.8 mg, 51% yield). **^{1}H NMR (500 MHz, CDCl$_3$)** δ 7.86–7.80 (m, 1 H), 7.71–7.68 (m, 2 H), 7.48–7.43 (m, 2 H), 7.33 (t, J = 7.0 Hz, 1 H), 7.09 (d, J = 7.0 Hz, 1 H), 6.93 (s, 2 H), 4.61 (d, J = 9.5 Hz, 1 H), 3.35–3.30 (m, 1 H), 2.98–2.92 (m, 1 H), 2.79–2.73 (m, 1 H), 2.64 (s, 6 H), 2.27 (s, 3 H), 2.10–2.01 (m, 2 H), 1.90–1.83 (m, 1 H), 1.69–1.51 (m, 6 H), 1.37–1.24 (m, 2 H). **^{13}C NMR (125 MHz, CDCl$_3$)** δ 142.2, 138.5, 137.0, 135.1, 133.8, 132.0, 131.5, 128.8, 126.9, 125.9, 125.52, 125.51, 125.4, 125.0, 123.2, 123.0 (dd, J_1 = 241.0 Hz, J_2 = 237.9 Hz), 57.6 (d, J = 2.3 Hz), 40.3, 33.4 (dt, J_1 = 24.1 Hz, J_2 = 5.0 Hz), 32.9, 29.2, 24.5 (dd, J_1 = 9.9 Hz, J_2 = 3.5 Hz), 23.2, 20.9. **^{19}F NMR (470 MHz, CDCl$_3$)** δ -91.7 (d, J = 235.5 Hz), -102.6 (d, J = 235.5 Hz). **HRMS** (ESI) m/z calcd. For $C_{28}H_{33}F_2NNaO_2S$ $[M+Na]^+$: 508.2092, found: 508.2105.

The enantiomeric ratio was determined to be 91.5:8.5 by SFC analysis: IC-3 column, MeOH/CO_2 = 20:80, 1.0 mL/min, 210 nm, t_{major} = 4.0 min, t_{minor} = 4.4 min.

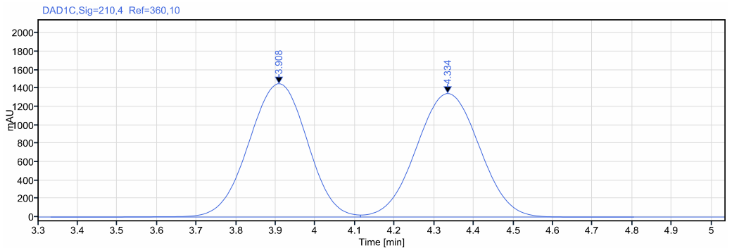

Signal: DAD1C,Sig=210,4 Ref=360,10

RT [min]	Type	Width [min]	Area	Height	Area%	Name
3.908	BV	0.78	14936.19	1450.97	50.08	
4.334	VB	0.69	14891.17	1344.63	49.92	
		Sum	29827.36			

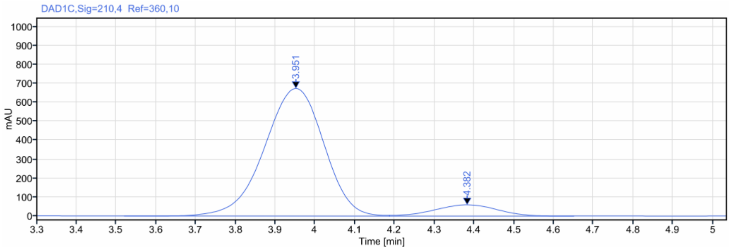

Signal: DAD1C,Sig=210,4 Ref=360,10

RT [min]	Type	Width [min]	Area	Height	Area%	Name
3.951	BV	0.67	7009.47	673.46	91.47	
4.382	VV	0.46	654.06	59.45	8.53	
		Sum	7663.53			

Supplementary Fig. 30 SFC spectra of **5ad**

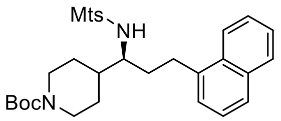

tert-Butyl (S)-4-(3-(naphthalen-1-yl)-1-((2,4,6-trimethylphenyl)sulfonamido)propyl)piperidine-1-carboxylate (5ae)

Following the **General Procedure C**, but (*R*)-BI-DIME (12 mol%) was used and the reaction was conducted at 80 ºC. The title compound was isolated as light yellow solid (29.3 mg, 53% yield). **^{1}H NMR (500 MHz, CDCl$_3$)** δ 7.84–7.82 (m, 1 H), 7.69 (d, *J* = 8.0 Hz, 2 H), 7.48–7.42 (m, 2 H), 7.33 (dd, J_1 = 8.0 Hz, J_2 = 7.0 Hz, 1 H), 7.09 (d, *J* = 7.0 Hz, 1 H), 6.92 (s, 2 H), 4.57 (d, *J* = 9.0 Hz, 1 H), 4.14–4.10 (m, 2 H), 3.32–3.27 (m, 1 H), 2.96–2.90 (m, 1 H), 2.80–2.74 (m, 1 H), 2.64–2.56 (m, 8 H), 2.27 (s, 3 H), 1.91–1.84 (m, 1 H), 1.65–1.56 (m, 3 H), 1.50–1.48 (m, 1 H), 1.43 (s, 9 H), 1.30–1.08 (m, 2 H). **^{13}C NMR (125 MHz, CDCl$_3$)** δ 154.6, 142.1, 138.4, 137.1, 135.2, 133.8, 132.0, 131.5, 128.8, 126.9, 125.8, 125.50, 125.48, 125.44, 123.2, 79.4, 58.1, 43.6, 40.6, 32.7, 29.1, 28.4, 27.9, 23.2, 20.9. **HRMS** (ESI) m/z calcd. For $C_{32}H_{42}N_2NaO_4S$ [M+Na]$^+$: 573.2757, found: 573.2762.

The enantiomeric ratio was determined to be 91.7:8.3 by SFC analysis: IC-3 column, MeOH/CO_2 = 20:80, 1.0 mL/min, 210 nm, t_{major} = 13.0 min, t_{minor} = 18.6 min.

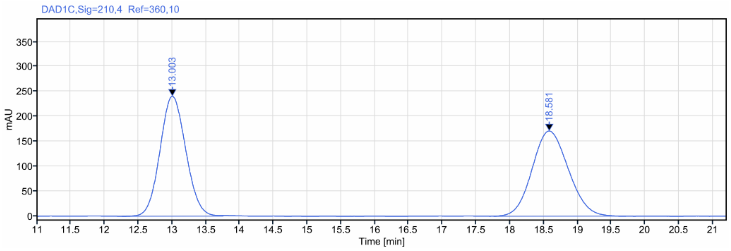

Signal: DAD1C,Sig=210,4 Ref=360,10

RT [min]	Type	Width [min]	Area	Height	Area%	Name
13.003	BV	1.50	6388.35	240.66	50.04	
18.581	VV	1.95	6377.93	170.92	49.96	
		Sum	12766.28			

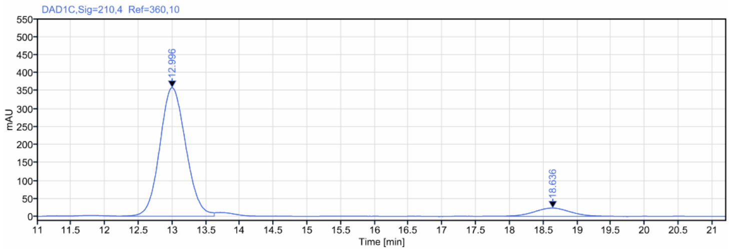

Signal: DAD1C,Sig=210,4 Ref=360,10

RT [min]	Type	Width [min]	Area	Height	Area%	Name
12.996	BV	1.38	9559.60	357.49	91.67	
18.636	VV	1.55	869.13	23.14	8.33	
		Sum	10428.73			

Supplementary Fig. 31 SFC spectra of **5ae**

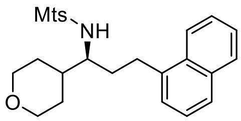

(*S*)-2,4,6-trimethyl-*N*-(3-(naphthalen-1-yl)-1-(tetrahydro-2*H*-pyran-4-yl)propyl)benzenesulfonamide (5af)

Following the **General Procedure C**, but (*R*)-BI-DIME (12 mol%) was used and the reaction was conducted at 80 ºC. The title compound was isolated as white solid (22.9 mg, 51% yield). **^{1}H NMR (500 MHz, CDCl$_3$)** δ 7.85–7.80 (m, 1 H), 7.72–7.68 (m, 2 H), 7.48–7.42 (m, 2 H), 7.33 (dd, J_1 = 8.0 Hz, J_2 = 7.5 Hz, 1 H), 7.10 (d, J = 7.0 Hz, 1 H), 6.92 (s, 2 H), 4.60 (d, J = 9.0 Hz, 1 H), 3.95–3.91 (m, 2 H), 3.30–3.24 (m, 3 H), 2.99–2.93 (m, 1 H), 2.82–2.76 (m, 1 H), 2.65 (s, 6 H), 2.27 (s, 3 H), 1.94–1.87 (m, 1 H), 1.78–1.71 (m, 1 H), 1.70–1.63 (m, 1 H), 1.51–1.49 (m, 1 H), 1.43–1.24 (m, 3 H). **^{13}C NMR (125 MHz, CDCl$_3$)** δ 142.0, 138.4, 137.2, 135.3, 133.8, 132.0, 131.5, 128.8, 126.8, 125.8, 125.5, 125.4, 123.3, 67.8, 67.7, 58.3, 39.5, 32.7, 29.0, 28.8, 28.6, 23.3, 20.9. **HRMS** (ESI) m/z calcd. For $C_{27}H_{33}NNaO_3S$ [M+Na]$^+$: 474.2073, found: 474.2085.

The enantiomeric ratio was determined to be 91.7:8.3 by SFC analysis: IC-3 column, MeOH/CO_2 = 10:90, 1.0 mL/min, 210 nm, t_{major} = 66.3 min, t_{minor} = 63.5 min.

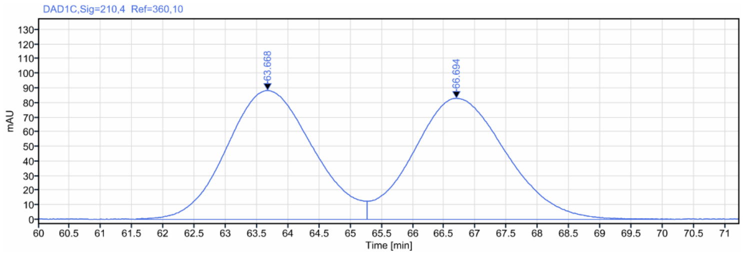

Signal: DAD1C,Sig=210,4 Ref=360,10

RT [min]	Type	Width [min]	Area	Height	Area%	Name
63.668	BV	3.73	8477.25	88.15	49.64	
66.694	VBA	4.93	8598.61	82.79	50.36	
		Sum	17075.86			

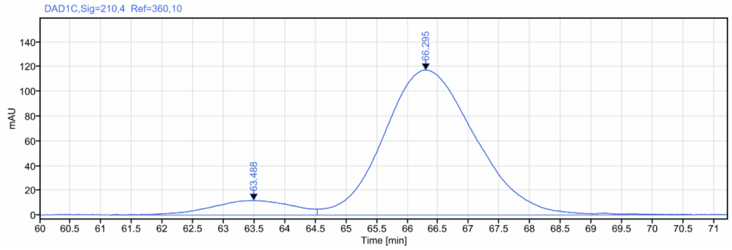

Signal: DAD1C,Sig=210,4 Ref=360,10

RT [min]	Type	Width [min]	Area	Height	Area%	Name
63.488	BV	3.37	1108.24	11.71	8.34	
66.295	VBA	6.15	12176.69	117.08	91.66	
		Sum	13284.94			

Supplementary Fig. 32 SFC spectra of **5af**

2.6 Mechanistic Studies

2.6.1 Control Experiments

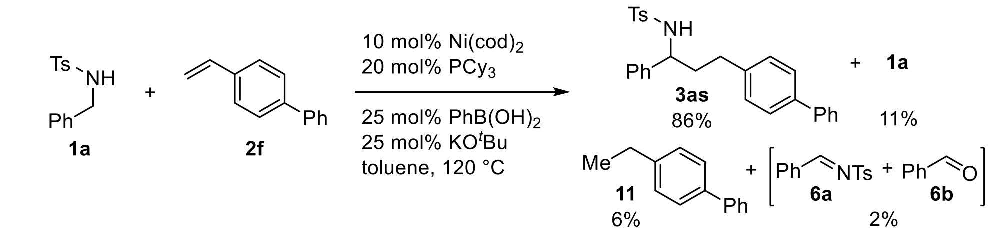

In a nitrogen-filled glovebox, a 4 mL oven-dried vial was charged with *N*-tosyl amine **1a** (0.2 mmol, 52.2 mg, 1.0 equiv.), olefin **2f** (0.4 mmol, 72.1 mg, 2.0 equiv.), $Ni(cod)_2$ (0.02 mmol, 5.5 mg, 10 mol%), PCy_3 (0.04 mmol, 11.2 mg, 20 mol%), $PhB(OH)_2$ (0.05 mmol, 6.1 mg, 25 mol%) and KO^tBu (0.05 mmol, 5.6 mg, 25 mol%). Toluene (0.3 mL) was added. The vial was equipped with a magnetic stir bar, sealed, and the reaction mixture was stirred at 120 °C for 20 h. Then the reaction mixture was cooled to room temperature. The reaction mixture was filtered with silica gel and concentrated under reduced pressure. The desired product **3as** (86%), *N*-tosyl amine **1a** (11%), alkane **11** (6%) and *N*-tosyl imine **6a** (2%, along with **6b**) were determined by ^{1}H NMR spectroscopy (1,1,2,2-tetrachloroethane as the internal standard). The alkane **11** was also confirmed by GC-MS.

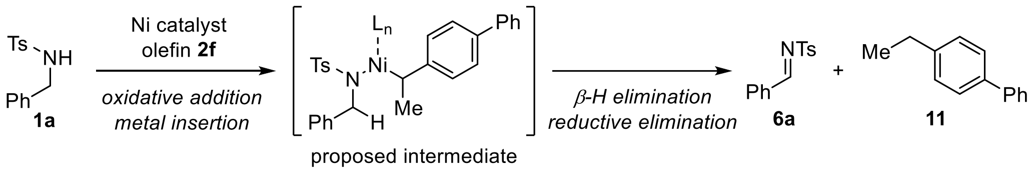

According to reported literature and our previous work,[20,32] we proposed that a trace amount of *N*-tosyl imine was generated via nickel-catalyzed sequential hydrogen transfer of *N*-tosyl amines with olefins in the initial step.

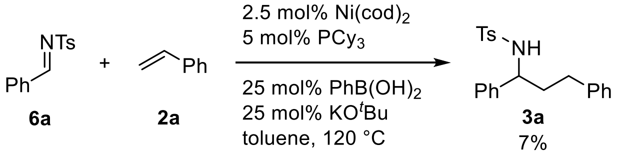

In a nitrogen-filled glovebox, a 4 mL oven-dried vial was charged with *N*-tosyl imine **6a** (0.2 mmol, 51.8 mg, 1.0 equiv.), styrene **2a** (0.4 mmol, 46 μL, 2.0 equiv.), $Ni(cod)_2$ (0.005 mmol, 1.4 mg, 2.5 mol%), PCy_3 (0.01 mmol, 2.8 mg, 5 mol%), $PhB(OH)_2$ (0.05 mmol, 6.1 mg, 25 mol%) and KO^tBu (0.05 mmol, 5.6 mg, 25 mol%). Toluene (0.3 mL) was added. The vial was equipped with a magnetic stir bar, sealed, and the reaction mixture was stirred at 120 °C for 20 h. Then the reaction mixture was cooled to room temperature. The reaction mixture was filtered with silica gel and concentrated under reduced pressure. The desired product **3a** (7%) was determined by ^{1}H NMR spectroscopy (1,1,2,2-tetrachloroethane as the internal standard).

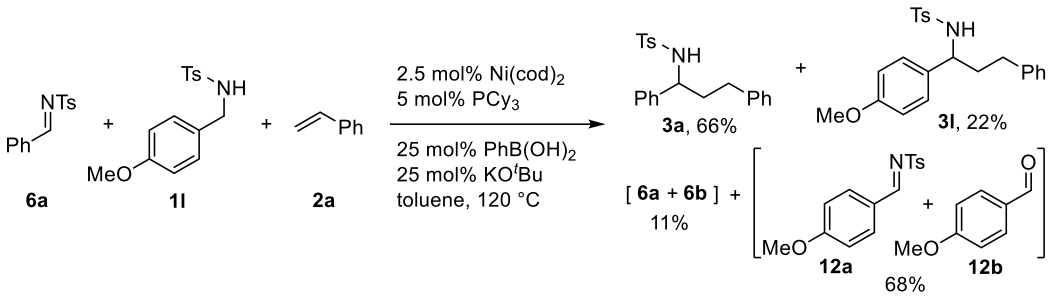

In a nitrogen-filled glovebox, a 4 mL oven-dried vial was charged with *N*-tosyl imine **6a** (0.20 mmol, 51.8 mg, 1.0 equiv.), *N*-tosyl amine **1l** (0.2 mmol, 58.2 mg, 1.0 equiv.), styrene **2a** (0.4 mmol, 46 μL, 2.0 equiv.), Ni(cod)$_2$ (0.005 mmol, 1.4 mg, 2.5 mol%), PCy$_3$ (0.01 mmol, 2.8 mg, 5 mol%), PhB(OH)$_2$ (0.05 mmol, 6.1 mg, 25 mol%) and KO^{t}Bu (0.05 mmol, 5.6 mg, 25 mol%). Toluene (0.3 mL) was added. The vial was equipped with a magnetic stir bar, sealed, and the reaction mixture was stirred at 120 °C for 20 h. Then the reaction mixture was cooled to room temperature. The reaction mixture was filtered with silica gel and concentrated under reduced pressure. The desired product **3a** and **3l** (66% and 22% respectively), *N*-tosyl imine **6a** (11%, along with **6b**) and *N*-tosyl imine **12a** (68%, along with **12b**) were determined by ^{1}H NMR spectroscopy (1,1,2,2-tetrachloroethane as the internal standard).

2.6.2 Deuterium Labelling Experiments

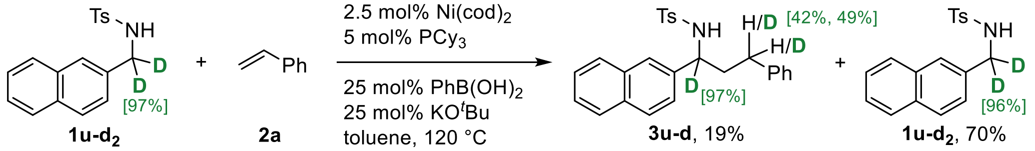

In a nitrogen-filled glovebox, a 4 mL oven-dried vial was charged with deuterium-labelled *N*-tosyl amine **1u-*d*2** (0.2 mmol, 62.6 mg, 1.0 equiv.), styrene **2a** (0.4 mmol, 46 μL, 2.0 equiv.), $Ni(cod)_2$ (0.005 mmol, 1.4 mg, 2.5 mol%), PCy_3 (0.01 mmol, 2.8 mg, 5 mol%), $PhB(OH)_2$ (0.05 mmol, 6.1 mg, 25 mol%) and KO^tBu (0.05 mmol, 5.6 mg, 25 mol%). Toluene (0.3 mL) was added. The vial was equipped with a magnetic stir bar, sealed, and the reaction mixture was stirred at 120 °C for 20 h. Then the reaction mixture was cooled to room temperature and concentrated under reduced pressure. Purification by column chromatography afforded the desired product **3u-d** (15.8 mg, 19%) and recovered *N*-tosyl amine **1u-d2** (43.9 mg, 70% recovery of starting material).

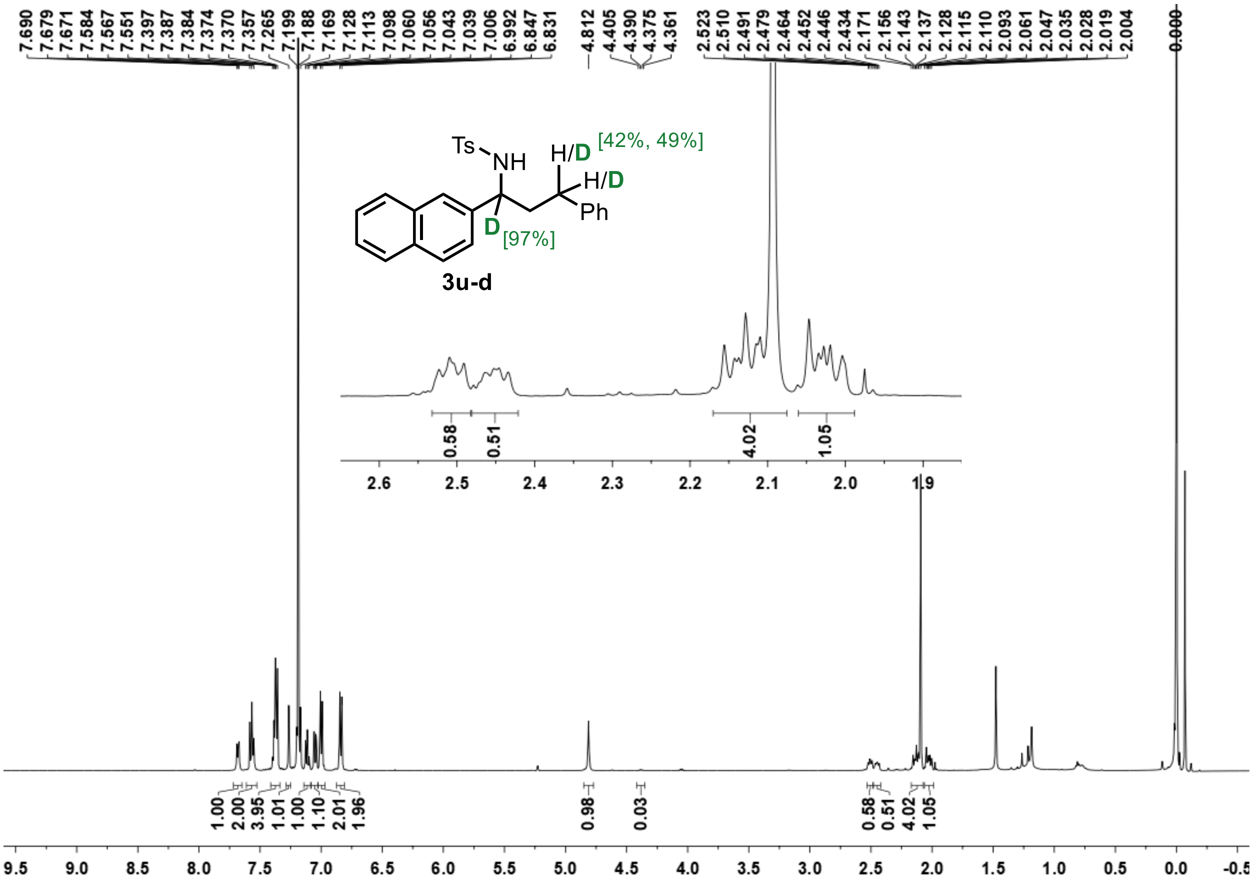

Supplementary Fig. 33 ^{1}H NMR (500 MHz, $CDCl_3$) of deuterium-labelled product **3u-d**

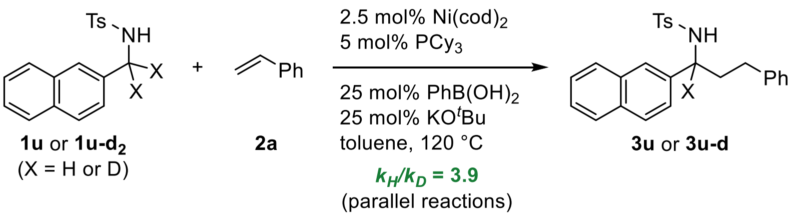

In a nitrogen-filled glovebox, a 4 mL oven-dried vial was charged with *N*-tosyl amine **1u** or **1u-d$_2$** (0.2 mmol, 1.0 equiv.), styrene **2a** (0.4 mmol, 46 μL, 2.0 equiv.), Ni(cod)$_2$ (0.005 mmol, 1.4 mg, 2.5 mol%), PCy$_3$ (0.01 mmol, 2.8 mg, 5 mol%), PhB(OH)$_2$ (0.05 mmol, 6.1 mg, 25 mol%) and KO^{t}Bu (0.05 mmol, 5.6 mg, 25 mol%). Toluene (0.3 mL) was added. The vial was equipped with a magnetic stir bar, sealed, and the reaction mixture was stirred at 120 °C for the specific time. All the reactions were quenched at low temperature with Dewar flask containing liquid nitrogen, filtered with silica gel and concentrated under reduced pressure. Yields were determined by ^{1}H NMR spectroscopy (1,1,2,2-tetrachloroethane as the internal standard). A primary kinetic isotope effect (k_H/k_D = 3.9) was obtained by parallel reactions.

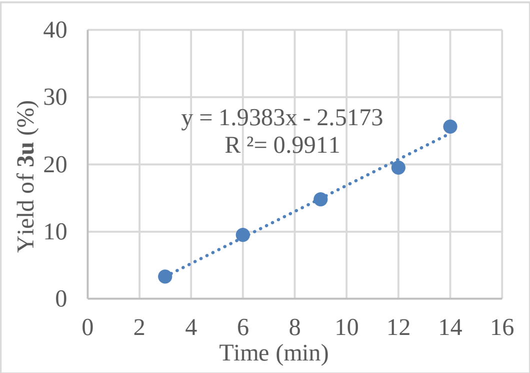

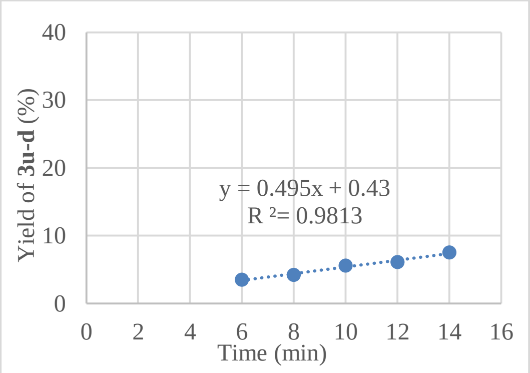

entry	time (min)	**3u** (%)
1	3	3.3
2	6	9.5
3	9	14.8
4	12	19.5
5	14	25.6

entry	time (min)	**3u-d** (%)
1	6	3.5
2	8	4.2
3	10	5.6
4	12	6.1
5	14	7.5

Supplementary Fig. 34 Deuterium labelling experiment

2.6.3 Role of Pivaldehyde

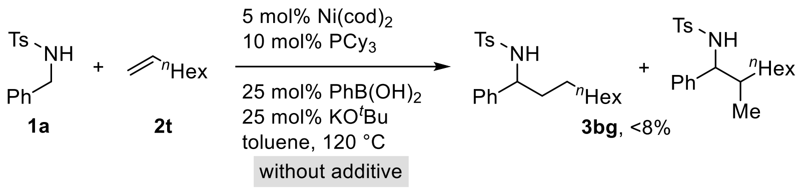

In a nitrogen-filled glovebox, a 4 mL oven-dried vial was charged with *N*-tosyl amine **1a** (0.2 mmol, 52.2 mg, 1.0 equiv.), olefin **2t** (0.4 mmol, 63 μL, 2.0 equiv.), Ni(cod)$_2$ (0.01 mmol, 2.8 mg, 5 mol%), PCy$_3$ (0.02 mmol, 5.6 mg, 10 mol%), PhB(OH)$_2$ (0.05 mmol, 6.1 mg, 25 mol%) and KO^{t}Bu (0.05 mmol, 5.6 mg, 25 mol%). Toluene (0.3 mL) was added. The vial was equipped with a magnetic stir bar, sealed, and the reaction mixture was stirred at 120 °C for 20 h. Then the reaction mixture was cooled to room temperature. The reaction mixture was filtered with silica gel and concentrated under reduced pressure. The desired products **3bg** (<8%) were determined by ^{1}H NMR spectroscopy (1,1,2,2-tetrachloroethane as the internal standard).

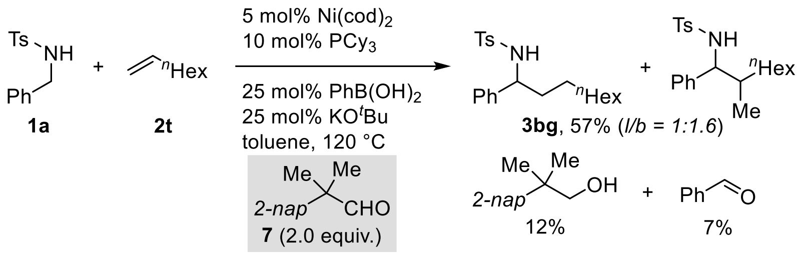

In a nitrogen-filled glovebox, a 4 mL oven-dried vial was charged with *N*-tosyl amine **1a** (0.2 mmol, 52.2 mg, 1.0 equiv.), olefin **2t** (0.4 mmol, 63 μL, 2.0 equiv.), Ni(cod)$_2$ (0.01 mmol, 2.8 mg, 5 mol%), PCy$_3$ (0.02 mmol, 5.6 mg, 10 mol%), PhB(OH)$_2$ (0.05 mmol, 6.1 mg, 25 mol%), KO^{t}Bu (0.05 mmol, 5.6 mg, 25 mol%) and *α,α,α*-trisubstituted aldehyde **7** (0.4 mmol, 79.2 mg, 2.0 equiv.). Toluene (0.3 mL) was added. The vial was equipped with a magnetic stir bar, sealed, and the reaction mixture was stirred at 120 °C for 20 h. Then the reaction mixture was cooled to room temperature. The reaction mixture was filtered with silica gel and concentrated under reduced pressure. The desired products **3bg** (57%, *l/b = 1:1.6*) and alcohol **7'** (12%, derived from *α,α,α*-trisubstituted aldehyde **7**) were determined by ^{1}H NMR spectroscopy (1,1,2,2-tetrachloroethane as the internal standard).

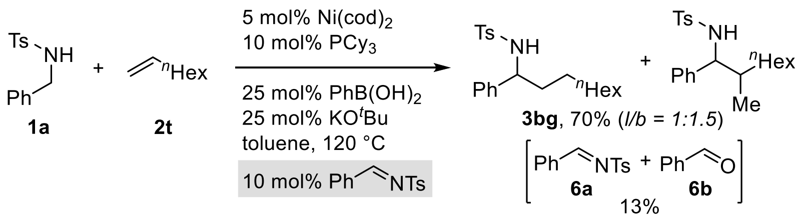

In a nitrogen-filled glovebox, a 4 mL oven-dried vial was charged with *N*-tosyl amine **1a** (0.2 mmol, 52.2 mg, 1.0 equiv.), *N*-tosyl imine **6a** (0.02 mmol, 5.2 mg, 10 mol%), olefin **2t** (0.4 mmol, 63 μL, 2.0 equiv.), Ni(cod)$_2$ (0.01 mmol, 2.8 mg, 5 mol%), PCy$_3$ (0.02 mmol, 5.6 mg, 10 mol%), PhB(OH)$_2$ (0.05 mmol, 6.1 mg, 25 mol%) and

KO^{t}Bu (0.05 mmol, 5.6 mg, 25 mol%). Toluene (0.3 mL) was added. The vial was equipped with a magnetic stir bar, sealed, and the reaction mixture was stirred at 120 °C for 20 h. Then the reaction mixture was cooled to room temperature. The reaction mixture was filtered with silica gel and concentrated under reduced pressure. The desired products **3bg** (70%, *l/b = 1:1.2*) and *N*-tosyl imine **6a** (13%, along with **6b**) were determined by ^{1}H NMR spectroscopy (1,1,2,2-tetrachloroethane as the internal standard).

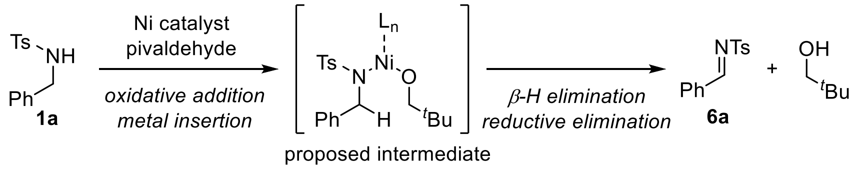

According to the above control experiments, we proposed that the *α,α,α*-trisubstituted aldehyde (pivaldehyde) might accept hydride from the Ni–H species generated in the initial step (*N*-tosyl amine dehydration), delivering a catalytic amount of *N*-tosyl imine.

2.6.4 Effects of KO^{t}Bu and $PhB(OH)_2$

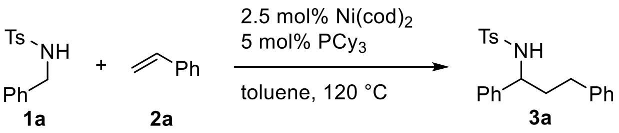

entry	KO^{t}Bu	additive	**3a** (%)
1	25 mol%	25 mol% $PhB(OH)_2$	quant.
2	25 mol%	none	7
3	0	25 mol% $PhB(OH)_2$	0
4	25 mol%	25 mol% $TsNH_2$	0
5	0	25 mol% $TsNH_2$	0
6	25 mol%	25 mol% PhOH	2
7	0	25 mol% PhOH	0

In a nitrogen-filled glovebox, a 4 mL oven-dried vial was charged with *N*-tosyl amine **1a** (0.2 mmol, 52.2 mg, 1.0 equiv.), styrene **2a** (0.4 mmol, 46 μL, 2.0 equiv.), $Ni(cod)_2$ (0.005 mmol, 1.4 mg, 2.5 mol%), PCy_3 (0.01 mmol, 2.8 mg, 5 mol%) (*with or without KO^{t}Bu (25 mol%)/additive (25 mol%)*). Toluene (0.3 mL) was added. The vial was equipped with a magnetic stir bar, sealed, and the reaction mixture was stirred at 120 °C for 20 h. Then the reaction mixture was cooled to room temperature. The reaction mixture was filtered with silica gel and concentrated under reduced pressure. Yields were determined by ^{1}H NMR spectroscopy (1,1,2,2-tetrachloroethane as the internal standard). Control experiments shown that both KO^{t}Bu and $PhB(OH)_2$ are crucial for this transformation. In previous reports, active hydrogen compounds (e.g. $TsNH_2$, phenol) are assumed to promote opening of the five-membered nickellacycle intermediate via protonation. We subjected them into the model reactions and found that neither $TsNH_2$ nor phenol promoted the transformation.

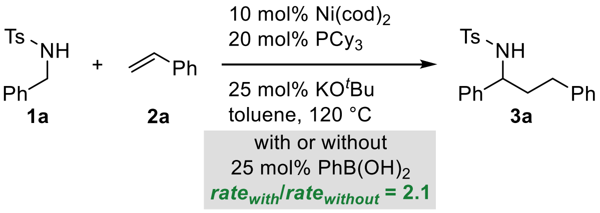

In a nitrogen-filled glovebox, a 4 mL oven-dried vial was charged with *N*-tosyl amine **1a** (0.2 mmol, 52.2 mg, 1.0 equiv.), styrene **2a** (0.4 mmol, 46 μL, 2.0 equiv.), $Ni(cod)_2$ (0.02 mmol, 5.5 mg, 10 mol%), PCy_3 (0.04 mmol, 11.2 mg, 20 mol%) and KO^{t}Bu (0.05 mmol, 5.6 mg, 25 mol%) (*with or without $PhB(OH)_2$ (25 mol%)*). Toluene (0.3 mL) was added. The vial was equipped with a magnetic stir bar, sealed, and the reaction mixture was stirred at 120 °C for the specific time. All the reactions were quenched at low temperature with Dewar flask containing liquid nitrogen, filtered with silica gel and concentrated under reduced pressure. Yields were determined by ^{1}H NMR spectroscopy (1,1,2,2-tetrachloroethane as the internal standard). The initial rate of this reaction was enhanced dramatically in the presence of $PhB(OH)_2$ ($rate_w/rate_{w/o}$ = 2.1). This result revealed that $PhB(OH)_2$ was crucial for reducing the global energetic barrier.

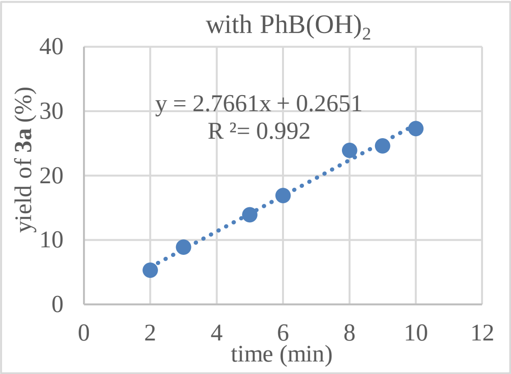

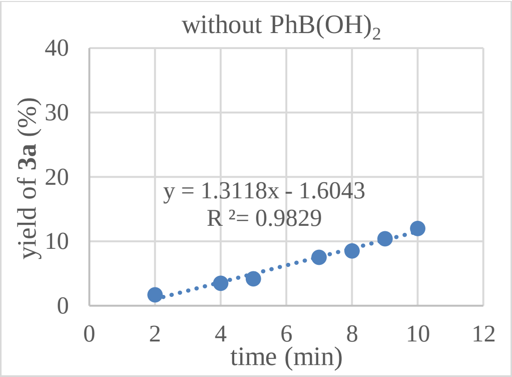

entry	time (min)	**3a** (%)
1	2	5.3
2	3	8.9
3	5	13.9
4	6	16.9
5	8	23.9
6	9	24.6
7	10	27.3

entry	time (min)	**3a** (%)
1	2	1.7
2	4	3.5
3	5	4.2
4	7	7.5
5	8	8.5
6	9	10.4
7	10	12.0

Supplementary Fig. 35 Effect of $PhB(OH)_2$

2.6.5 ^{11}B NMR Study

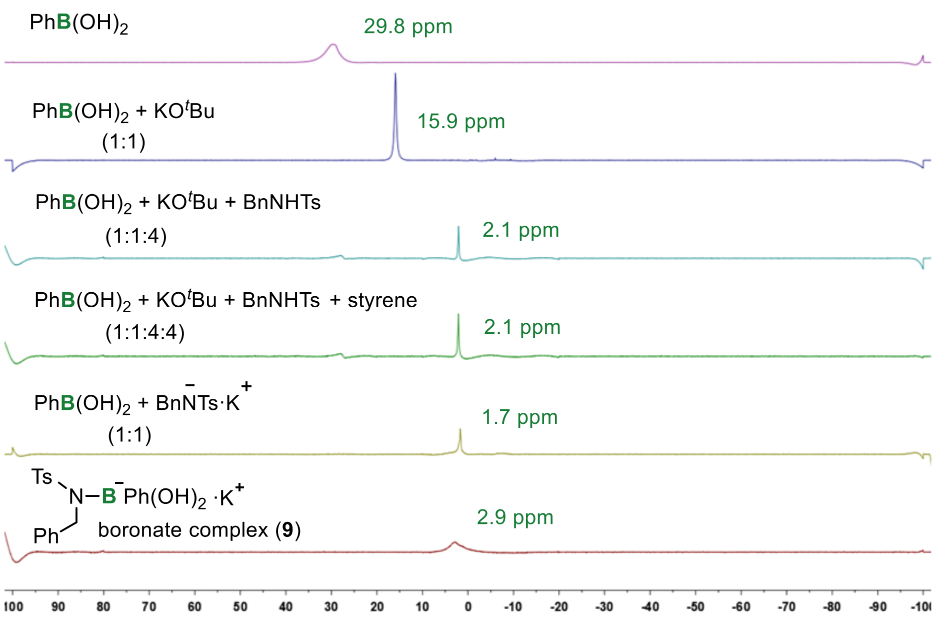

Supplementary Fig. 36 ^{11}B NMR study with KO^{t}Bu

In a nitrogen-filled glovebox, a 4 mL oven-dried vial was charged with PhB(OH)$_2$ (0.05 mmol, 6.1 mg), KO^{t}Bu (0.05 mmol, 6.1 mg), *N*-tosyl amine **1a** (0.2 mmol, 52.2 mg) and styrene **2a** (0.2 mmol, 23 μL). Toluene-d_8 (0.4 mL) was added. The vial was equipped with a magnetic stir bar, sealed, and the reaction mixture was stirred at 120 °C for 30 min. Then the reaction mixture was cooled to room temperature and monitored by ^{11}B NMR spectroscopy. A new peak appeared in the upfield, implying the formation of a borate. Notably, this peak still appeared in the absence of an alkene and when PhB(OH)$_2$ was treated with potassium sulfonamide (**8**) directly. Moreover, the boronate complex (**9**) bearing a B–N bond was isolated and further characterized by NMR spectroscopy and elementary analysis.

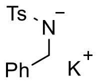

Potassium benzyl(tosyl)amide (8)

In a nitrogen-filled glovebox, a 50 mL round-bottom flask was charged with *N*-tosyl amine **1a** (5 mmol, 1.305 g, 1.0 equiv.) and KO^{t}Bu (5 mmol, 0.561 g, 1.0 equiv.). Dry THF (20 mL) was added. The flask was equipped with a magnetic stir bar, sealed, and the reaction mixture was stirred at room temperature for 2 h. Then the solvent was evaporated. The residue was washed with Et$_2$O and brought to dryness under reduced pressure, affording the title potassium salt (white solid, 1.481 g, 99%). **^{1}H NMR (500 MHz, CDCl$_3$)** δ 7.53 (d, *J* = 8.0 Hz, 2 H), 7.05–6.98 (m, 5 H), 6.85 (d, *J* = 8.0 Hz, 2 H), 3.73 (s, 2 H), 2.23 (s, 3 H). **^{13}C NMR (125 MHz, CDCl$_3$)** δ 143.2, 141.3, 139.8, 129.1, 128.1, 127.6, 126.8, 125.9, 49.5, 21.2.

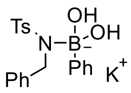

Potassium ((*N*-benzyl-4-methylphenyl)sulfonamido)dihydroxy(phenyl)borate (9)

In a nitrogen-filled glovebox, a 15 mL oven-dried sealed tube was charged with BnNKTs **8** (1 mmol, 0.299 g, 1.0 equiv.) and $PhB(OH)_2$ (1 mmol, 0.122 g, 1.0 equiv.). Toluene (5 mL) was added. The flask was equipped with a magnetic stir bar, sealed, and the reaction mixture was stirred at 120 °C for 1 h. Then the tube was transferred into glovebox again. Dry pentane was added to precipitate the product out. Collected the precipitation, which was washed again with pentane and brought to dryness under reduced pressure, affording the title boronate complex (white solid, 0.413 g, 98%). **^{1}H NMR (500 MHz, CD_3OD)** δ 7.72 (d, J = 8.5 Hz, 2 H), 7.51 (d, J = 6.5 Hz, 2 H), 7.34 (d, J = 8.0 Hz, 2 H), 7.26–7.13 (m, 7 H), 7.05–7.02 (m, 1 H), 4.03 (s, 2 H), 2.42 (s, 3 H). **^{13}C NMR (125 MHz, CD_3OD)** δ 144.6, 139.1, 138.7, 134.2, 130.7, 129.4, 128.9, 128.4, 128.1, 127.4, 126.0, 47.9, 21.4. **^{11}B NMR (160 MHz, CD_3OD)** δ 6.0. **Elementary analysis**: calcd. For [$C_{20}H_{21}BKNO_4S$] B: 2.57%, S: 7.61%; found: B: 2.73%, S: 7.50%.

According to the above method, the interactions between $PhB(OH)_2$, LiO^{t}Bu, and *N*-Ts benzylamine were investigated. LiO^{t}Bu behaves similarly to KO^{t}Bu that a lithium boronate complex formed (Figure S5). Following the synthetic procedure of potassium boronate complex (**9**), the corresponding lithium boronate complex (**13**) was synthesized in toluene-d_8, and characterized by ^{11}B NMR and ^{1}H NMR spectroscopy.

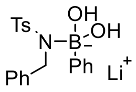

Lithium ((*N*-benzyl-4-methylphenyl)sulfonamido)dihydroxy(phenyl)borate (13)

^{1}H NMR (500 MHz, toluene-d_8) δ 7.64–7.62 (m, 3 H), 7.33–7.23 (m, 2 H), 6.98–6.91 (m, 6 H), 6.78–6.77 (m, 3 H), 3.75 (s, 2 H), 1.95 (s, 3 H). **^{11}B NMR (160 MHz, toluene-d_8)** δ 2.3.

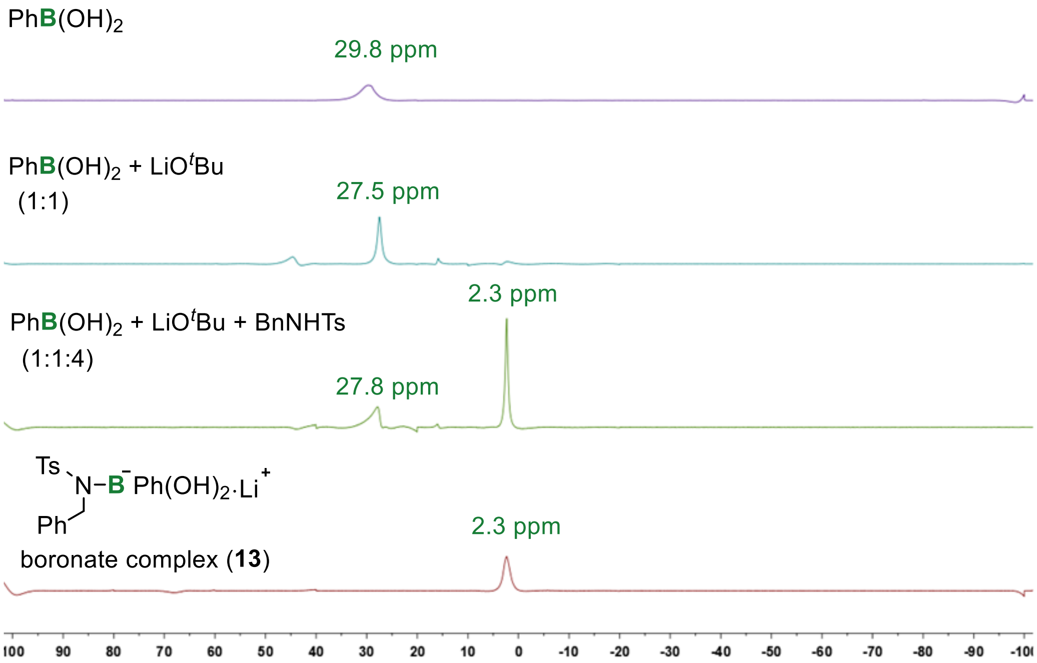

Supplementary Fig. 37 ^{11}B NMR study with LiO^{t}Bu

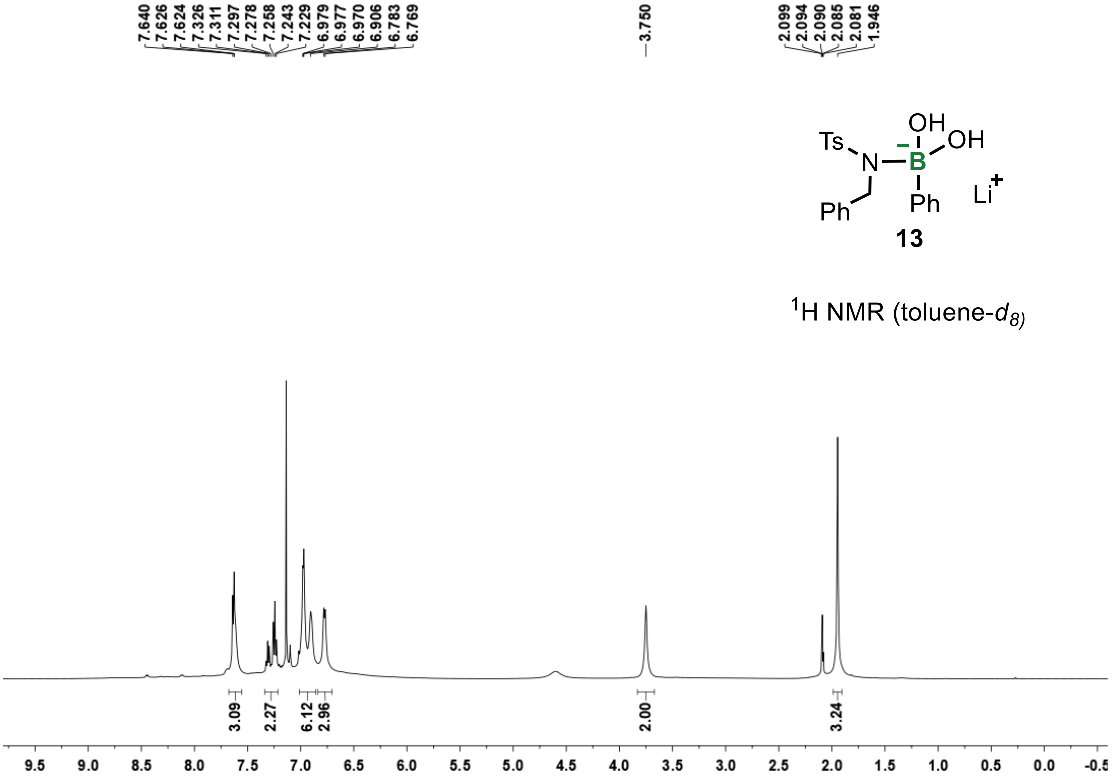

Supplementary Fig. 38 ^{1}H NMR (500 MHz, toluene-d_8) of lithium boronate complex **13**

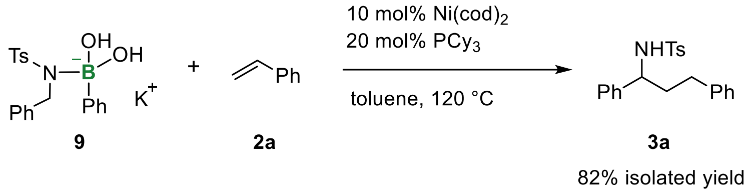

Based on the above results, a control experiment using potassium boronate complex (**9**) as the starting material without any additive was conducted. In a nitrogen-filled glovebox, a 4 mL oven-dried vial was charged with **9** (0.2 mmol, 84.2 mg, 1.0 equiv.), styrene **2a** (0.4 mmol, 46 μL, 2.0 equiv.), $Ni(cod)_2$ (0.02 mmol, 5.6 mg, 10 mol%), PCy_3 (0.04 mmol, 11.2 mg, 20 mol%). Toluene (0.3 mL) was added. The vial was equipped with a magnetic stir bar, sealed, and the reaction mixture was stirred at 120 °C for 20 h. The reaction mixture was cooled to room temperature and concentrated under reduced pressure. Purification by column chromatography afforded the desired product **3a** (59.9 mg, 82% yield).

2.7 NMR Spectra

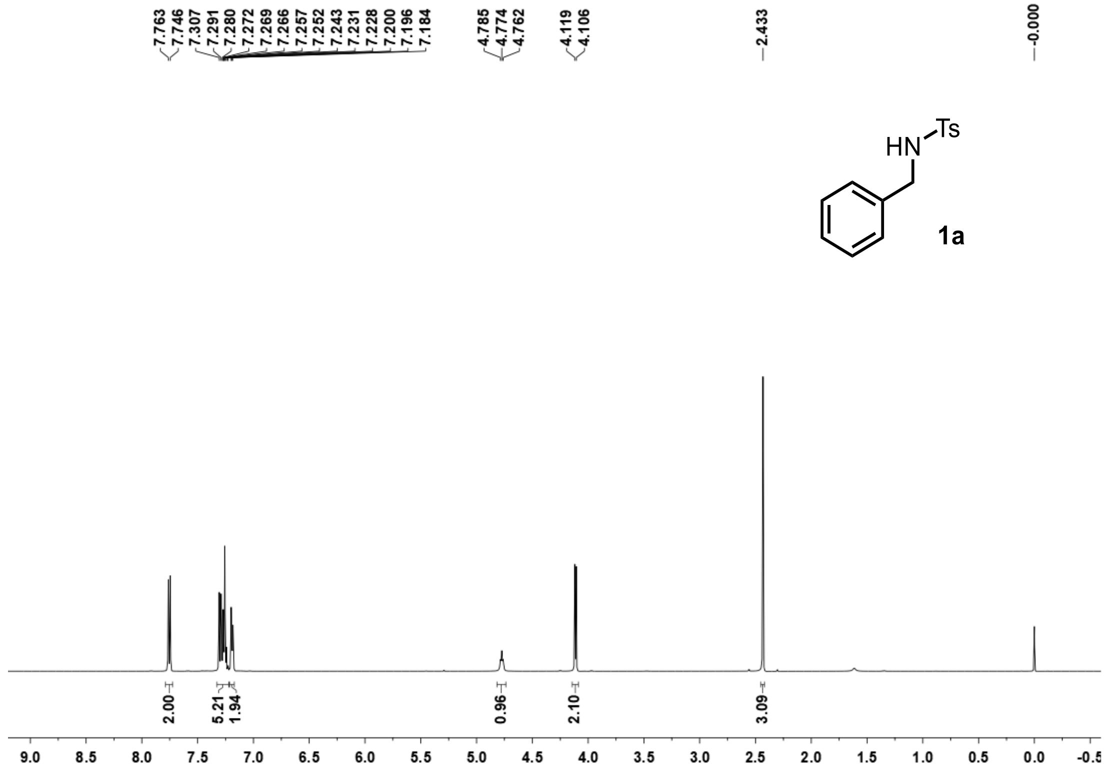

Supplementary Fig. 39 ^{1}H NMR (500 MHz, $CDCl_3$) of **1a**

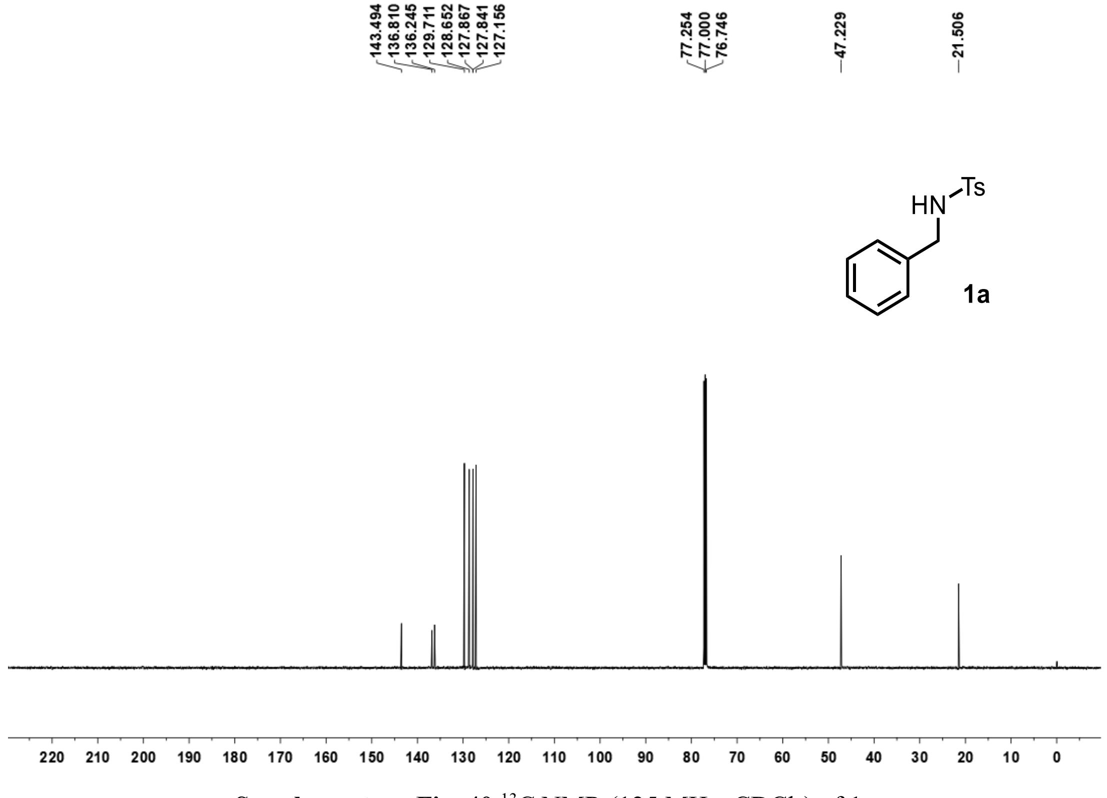

Supplementary Fig. 40 ^{13}C NMR (125 MHz, $CDCl_3$) of **1a**

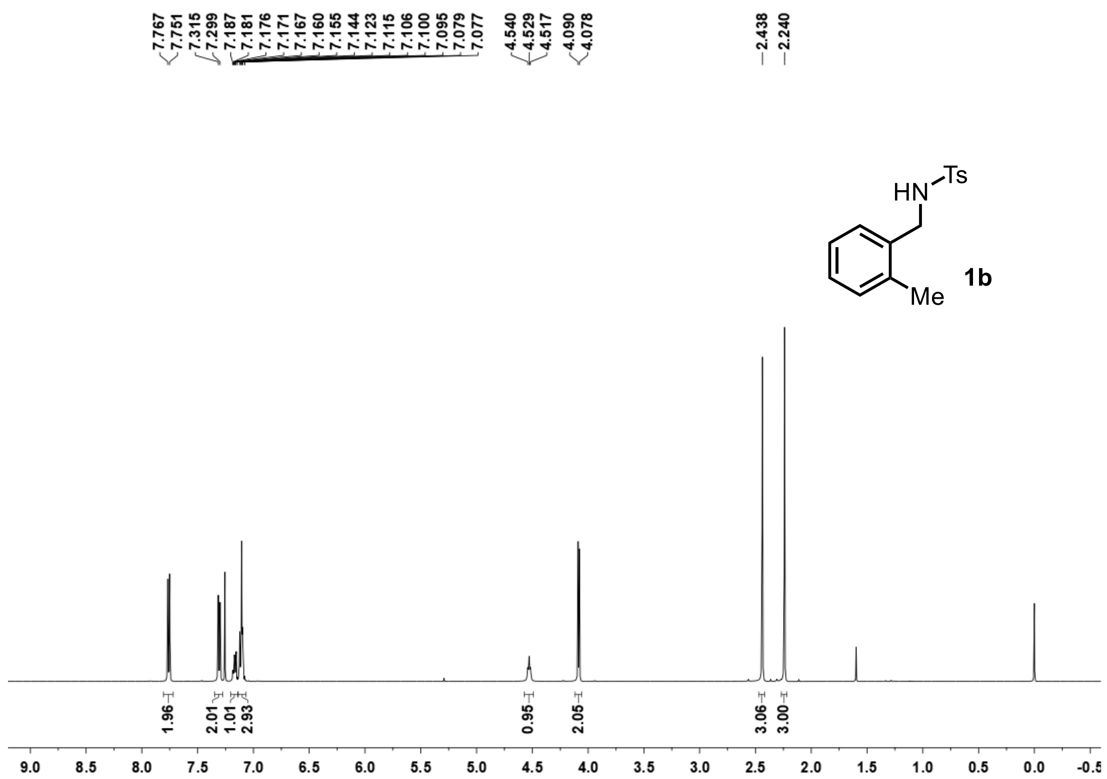

Supplementary Fig. 41 ^{1}H NMR (500 MHz, $CDCl_3$) of **1b**

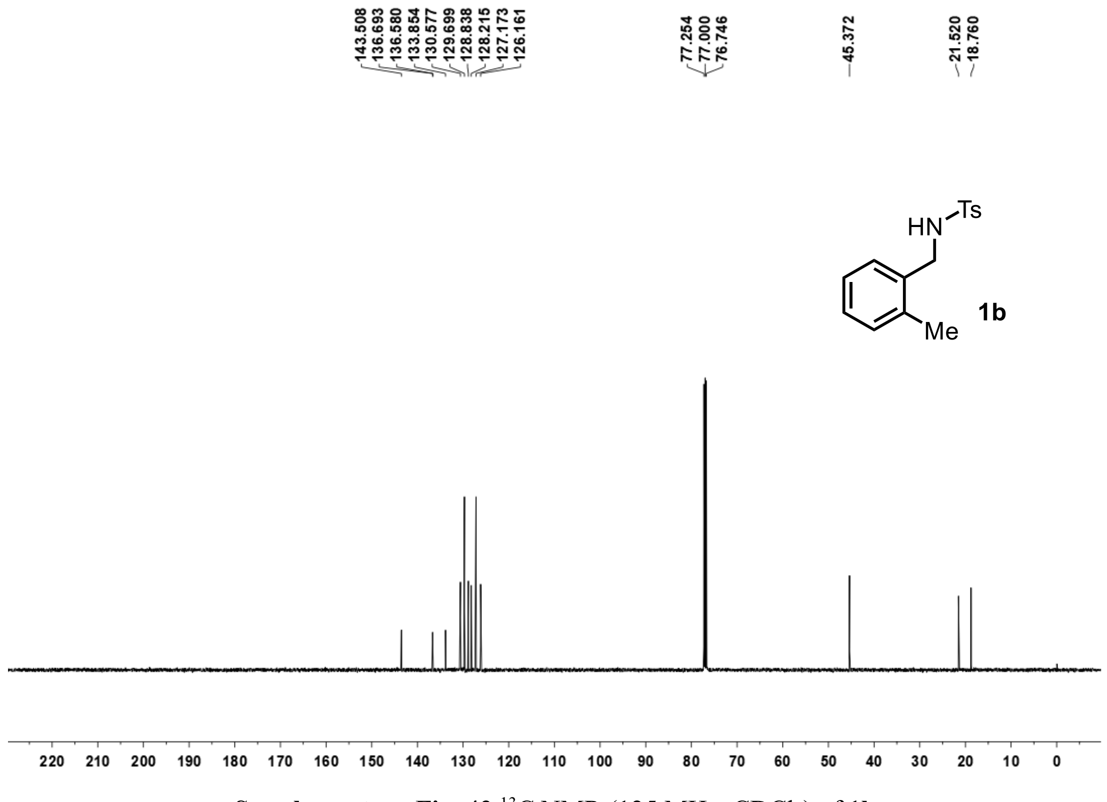

Supplementary Fig. 42 ^{13}C NMR (125 MHz, $CDCl_3$) of **1b**

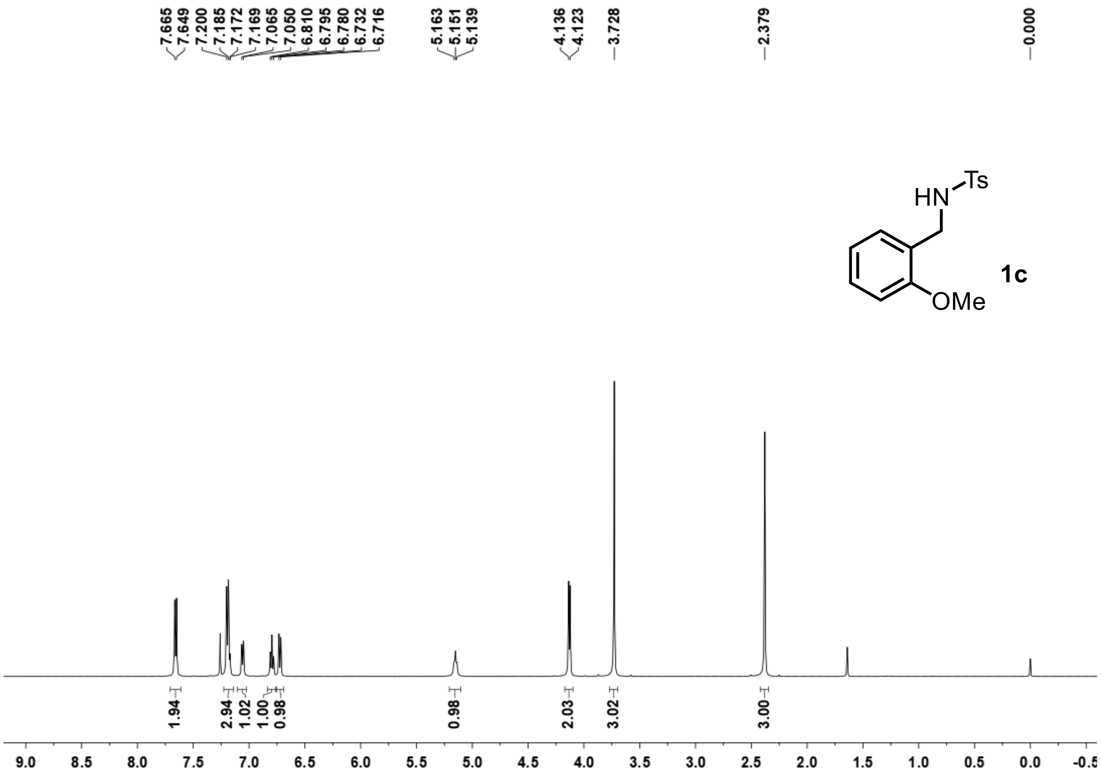

Supplementary Fig. 43 ^{1}H NMR (500 MHz, $CDCl_3$) of **1c**

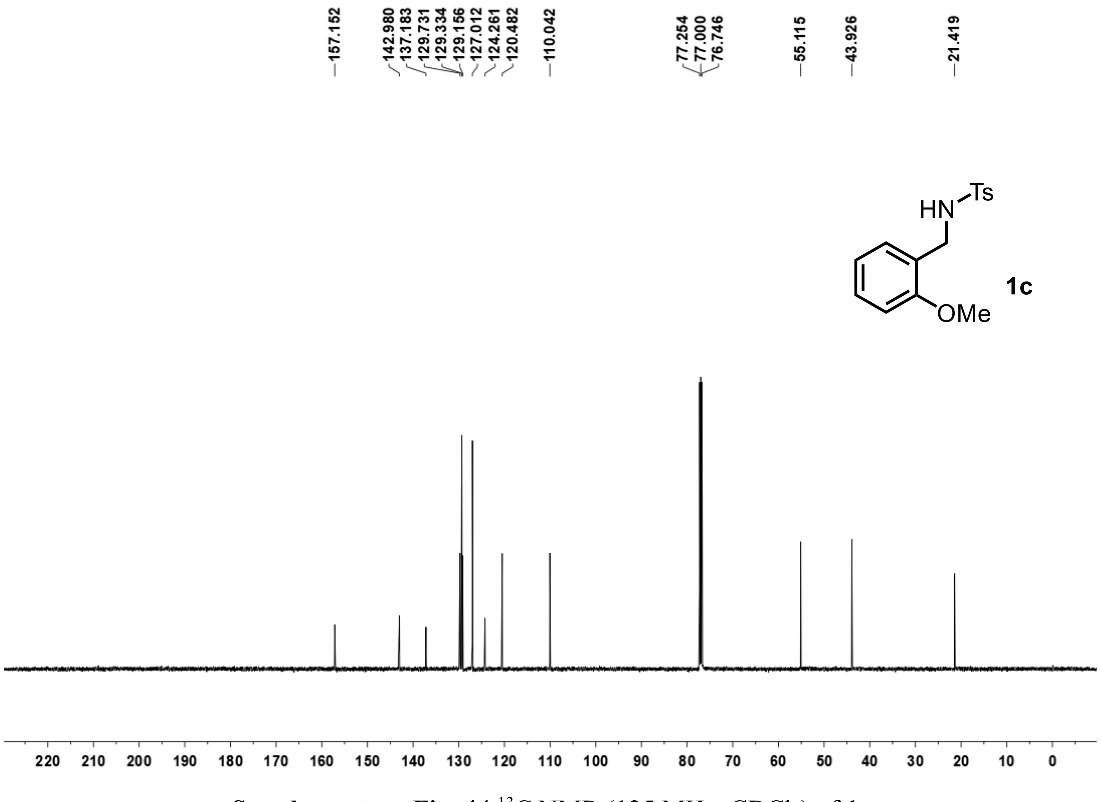

Supplementary Fig. 44 ^{13}C NMR (125 MHz, $CDCl_3$) of **1c**

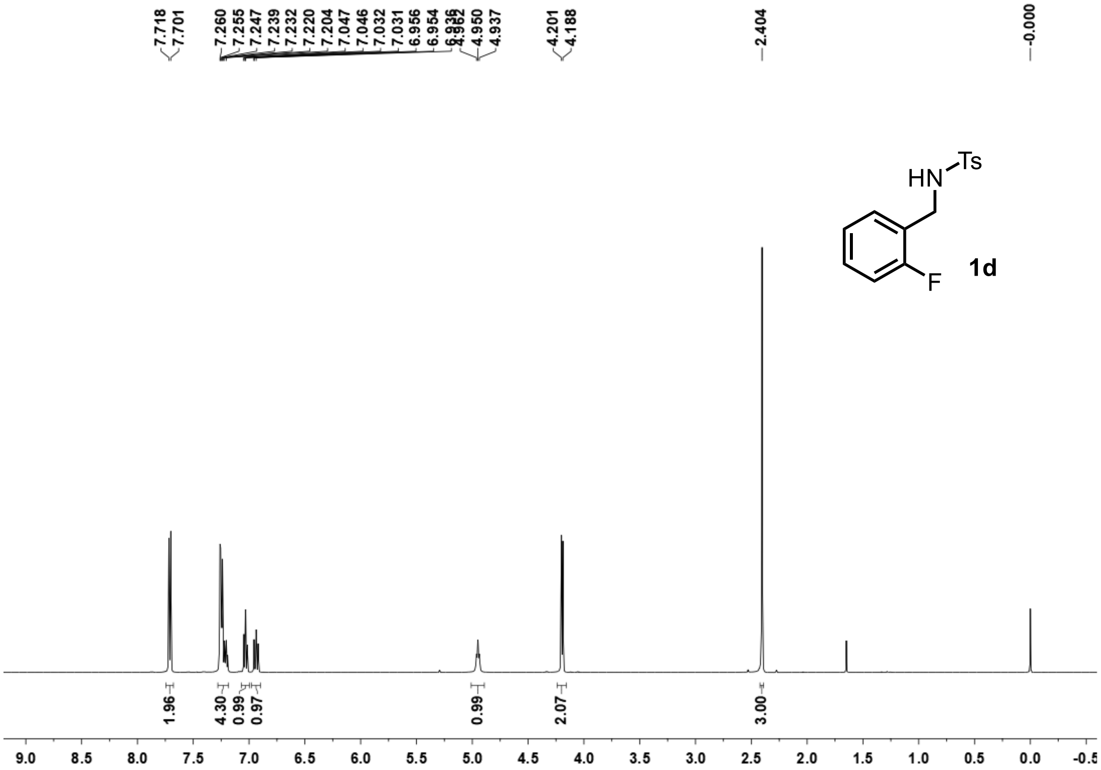

Supplementary Fig. 45 ^{1}H NMR (500 MHz, $CDCl_3$) of **1d**

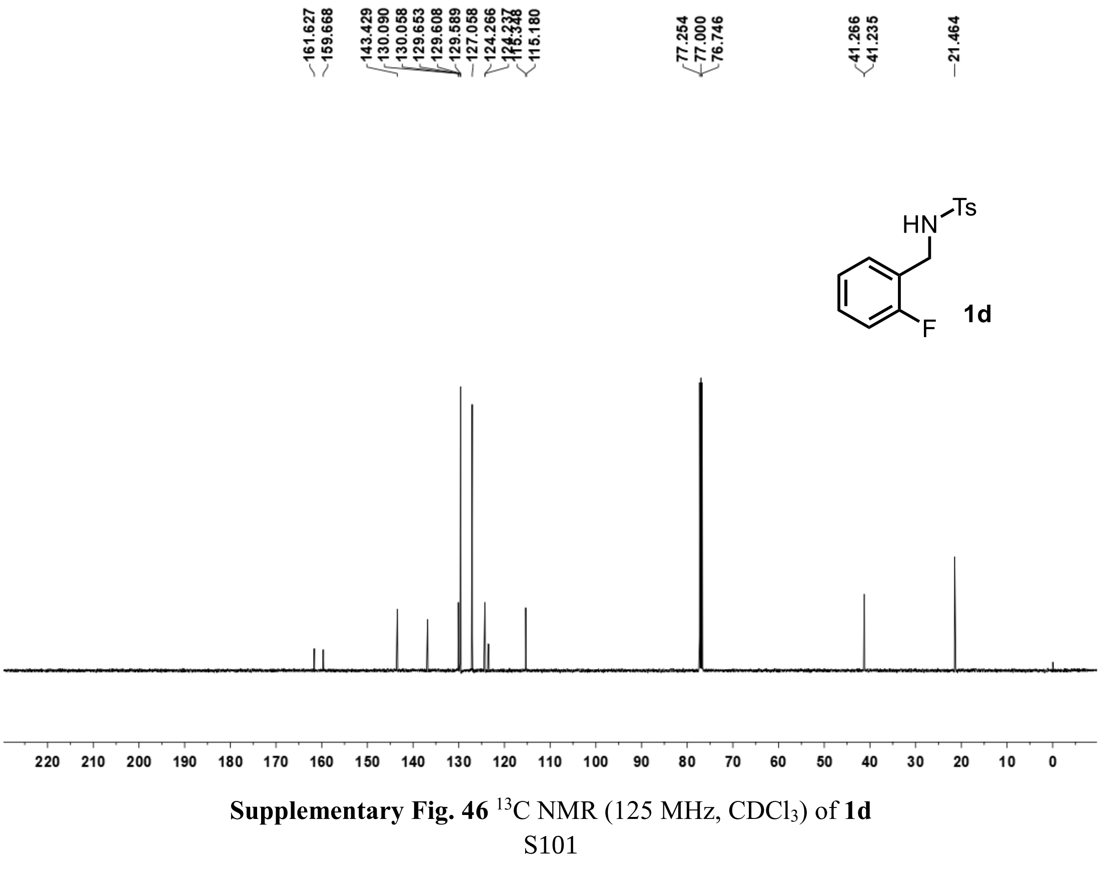

Supplementary Fig. 46 ^{13}C NMR (125 MHz, $CDCl_3$) of **1d**

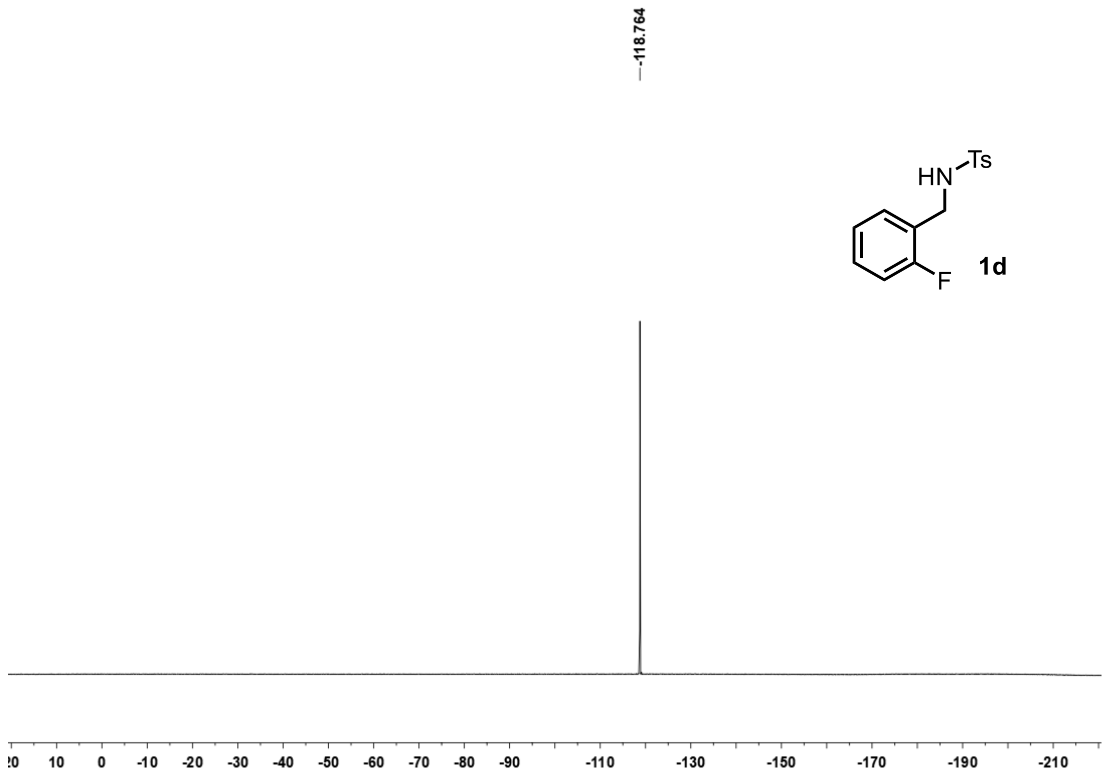

Supplementary Fig. 47 ^{19}F NMR (470 MHz, $CDCl_3$) of **1d**

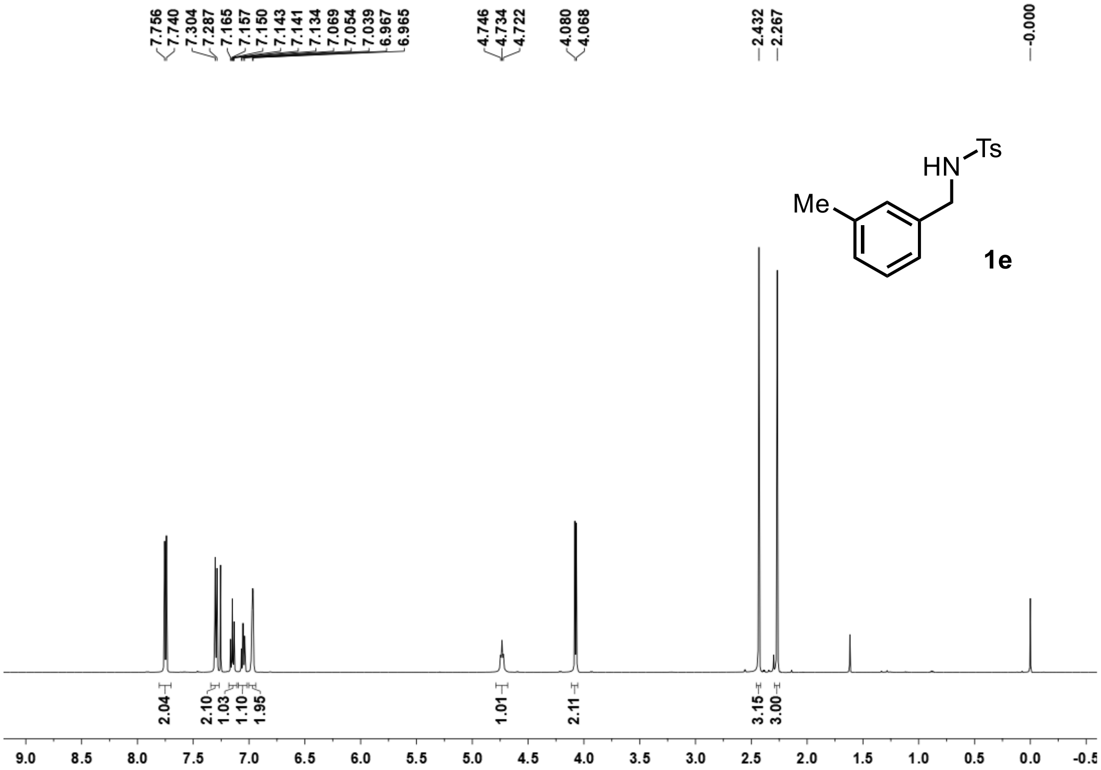

Supplementary Fig. 48 ^{1}H NMR (500 MHz, $CDCl_3$) of **1e**

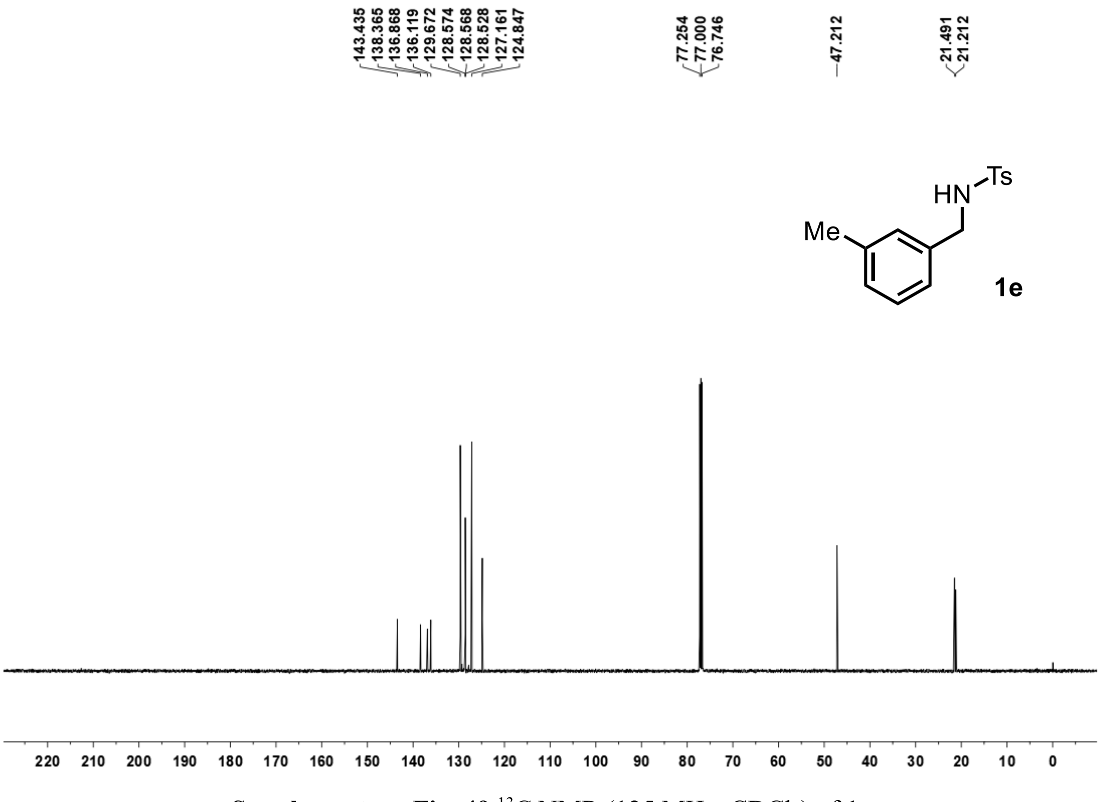

Supplementary Fig. 49 ^{13}C NMR (125 MHz, $CDCl_3$) of **1e**

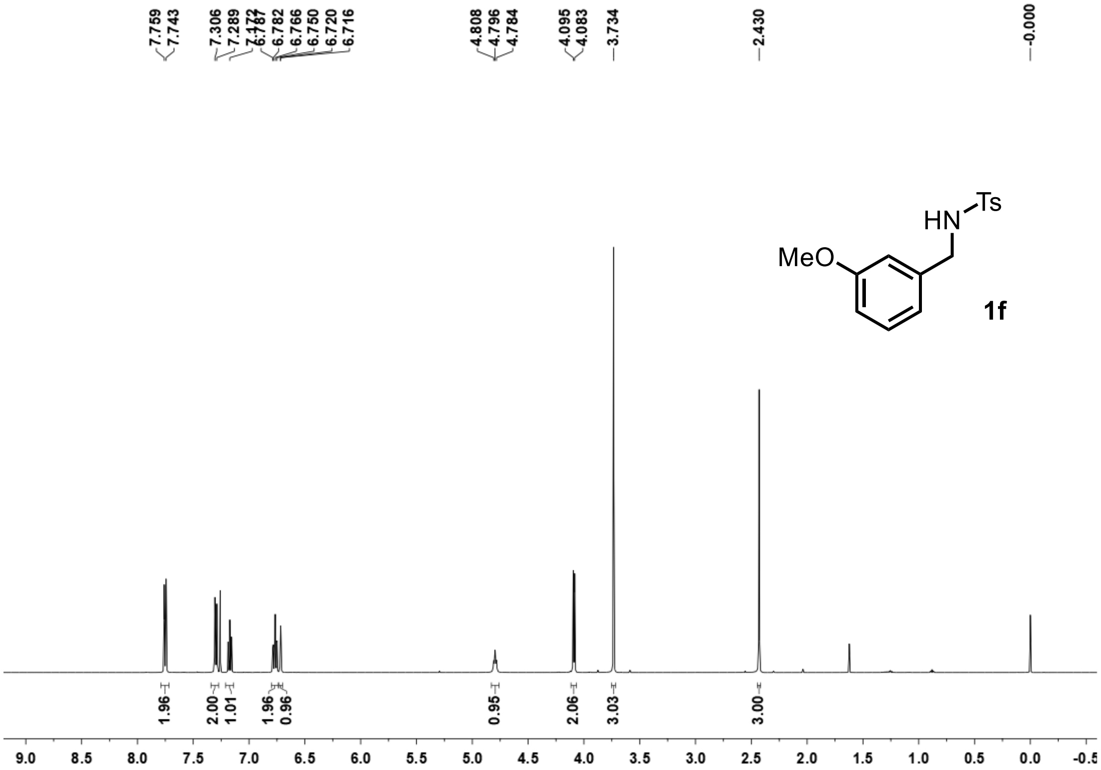

Supplementary Fig. 50 ^{1}H NMR (500 MHz, $CDCl_3$) of **1f**

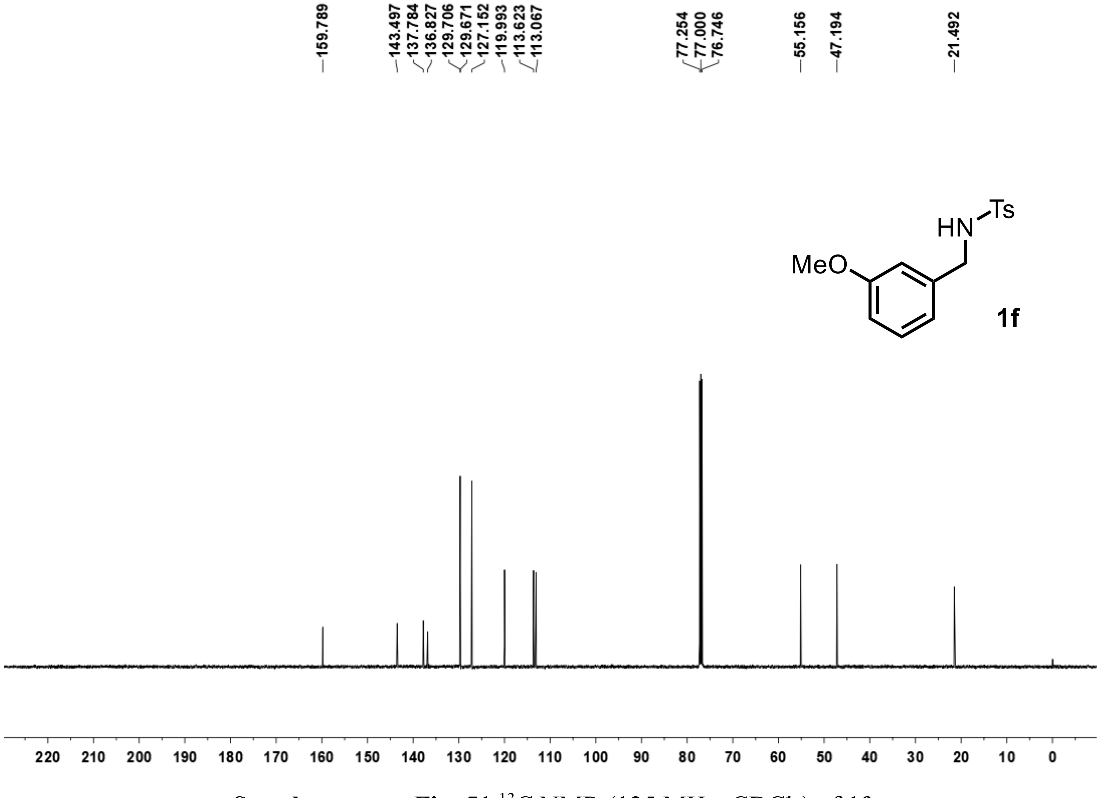

Supplementary Fig. 51 ^{13}C NMR (125 MHz, $CDCl_3$) of **1f**

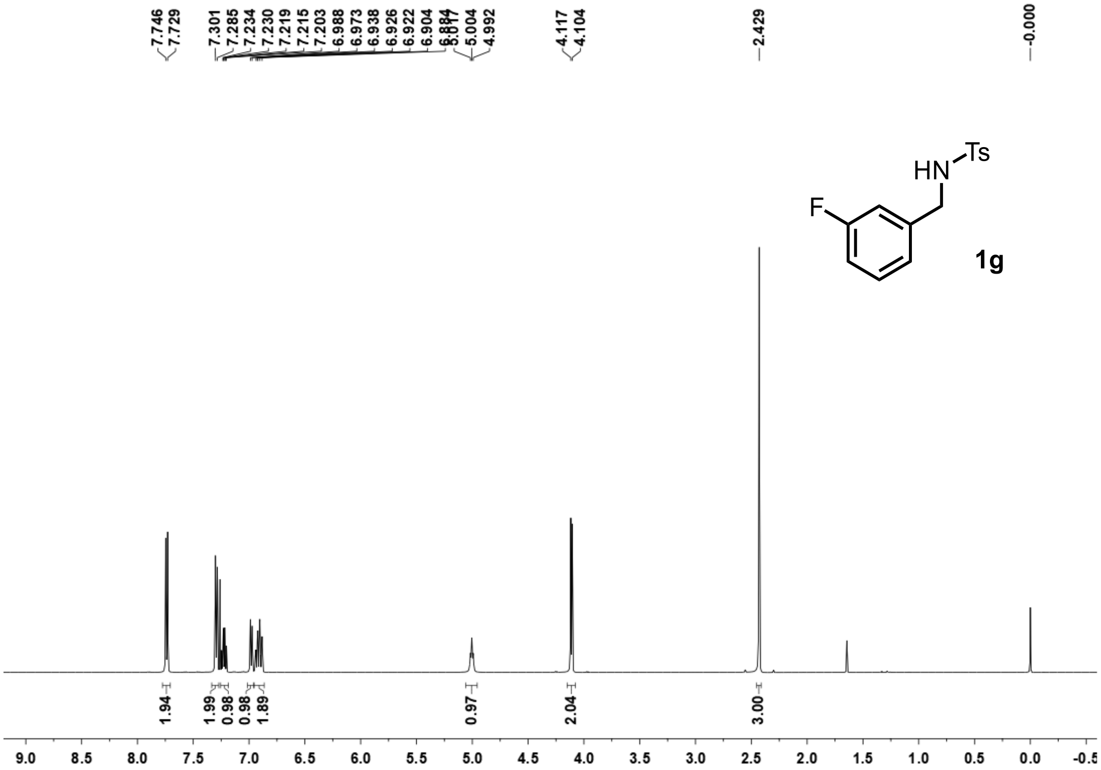

Supplementary Fig. 52 ^{1}H NMR (500 MHz, $CDCl_3$) of **1g**

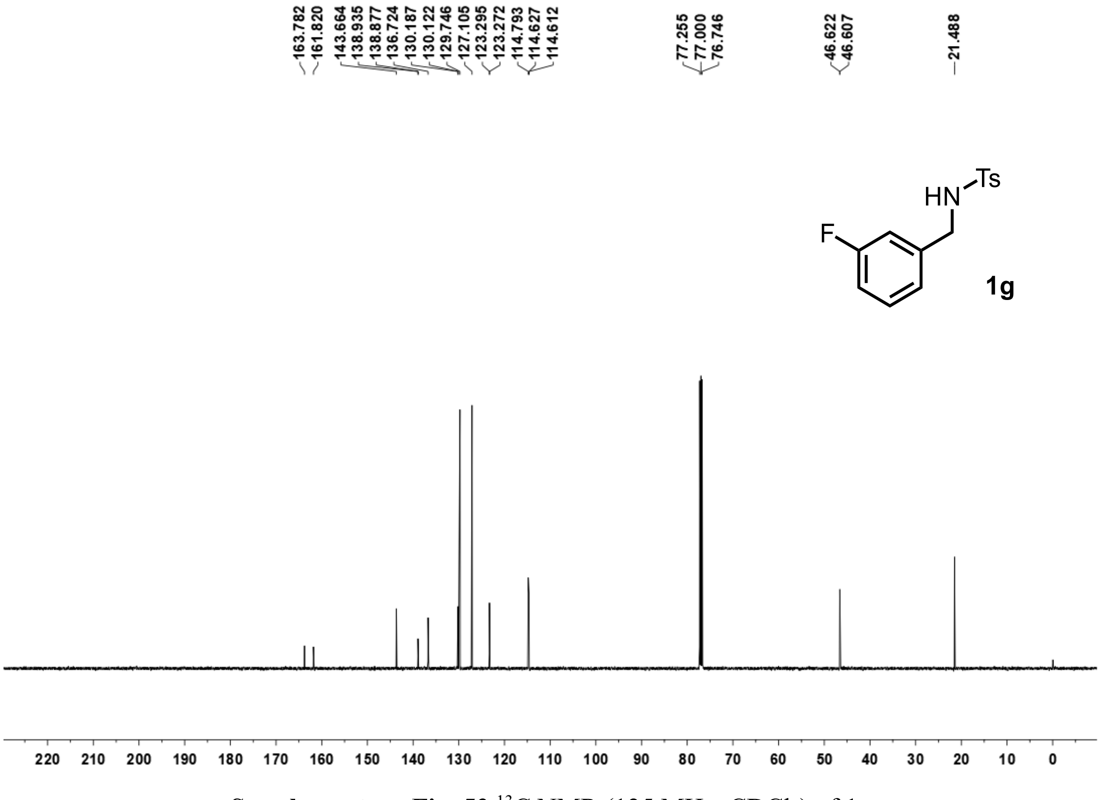

Supplementary Fig. 53 ^{13}C NMR (125 MHz, $CDCl_3$) of **1g**

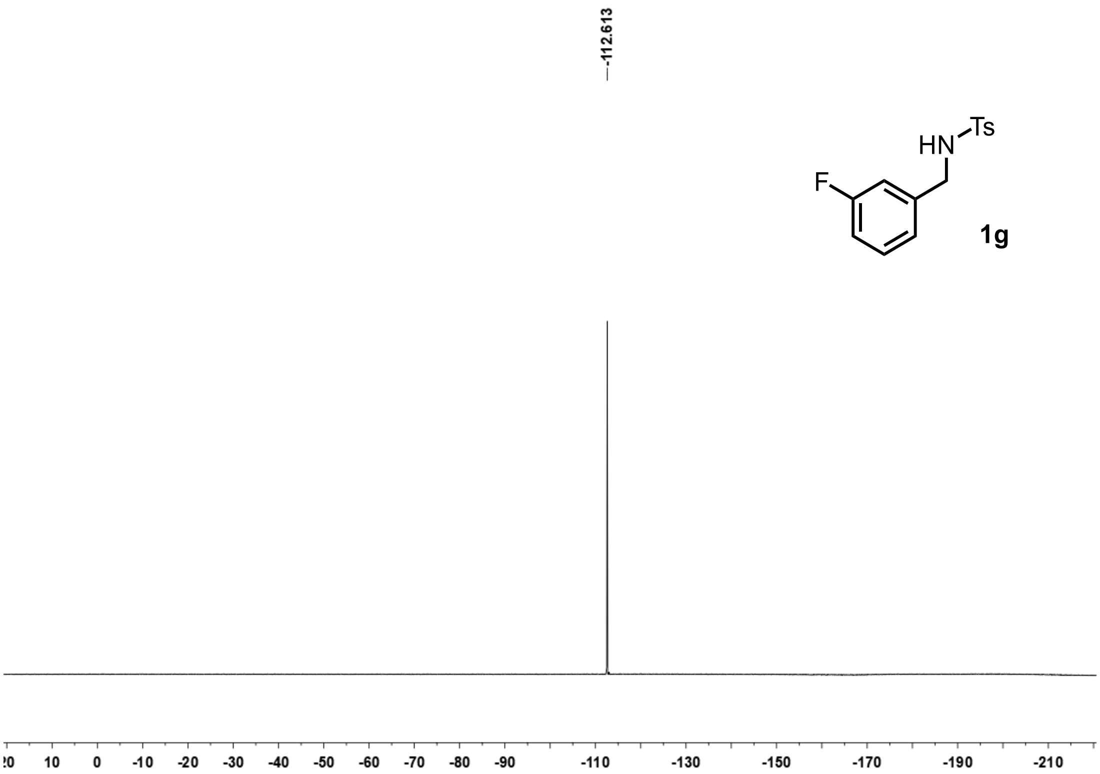

Supplementary Fig. 54 ^{19}F NMR (470 MHz, $CDCl_3$) of **1g**

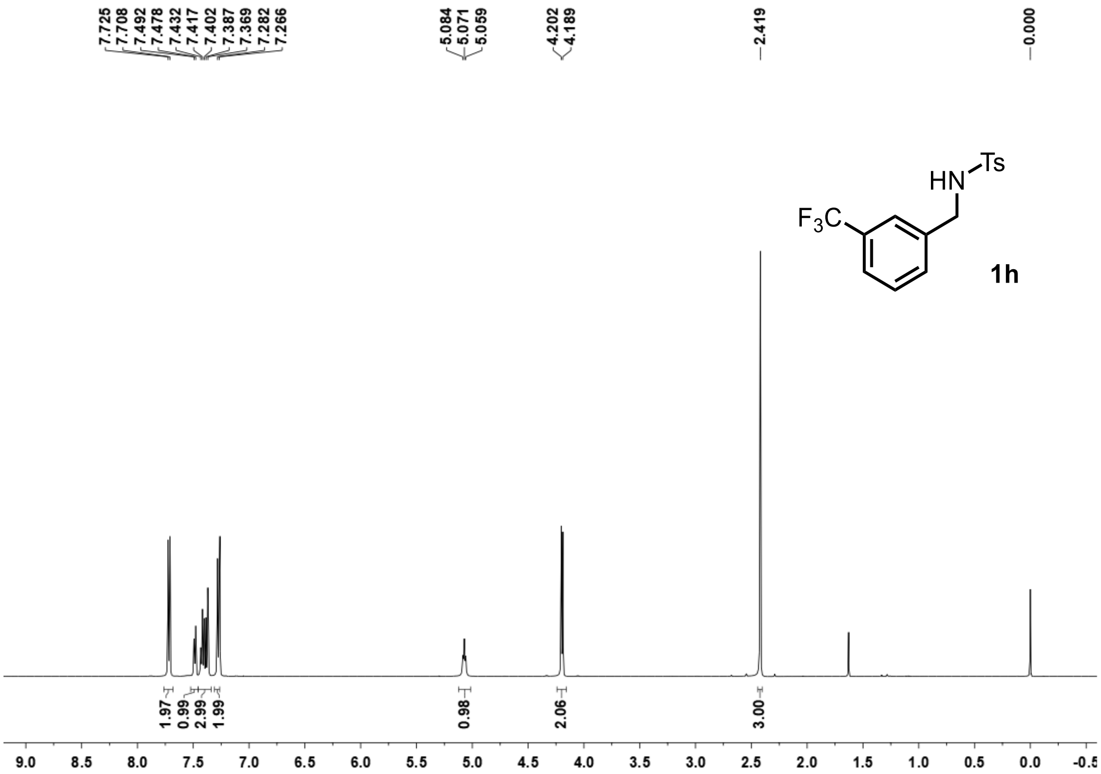

Supplementary Fig. 55 ^{1}H NMR (500 MHz, $CDCl_3$) of **1h**

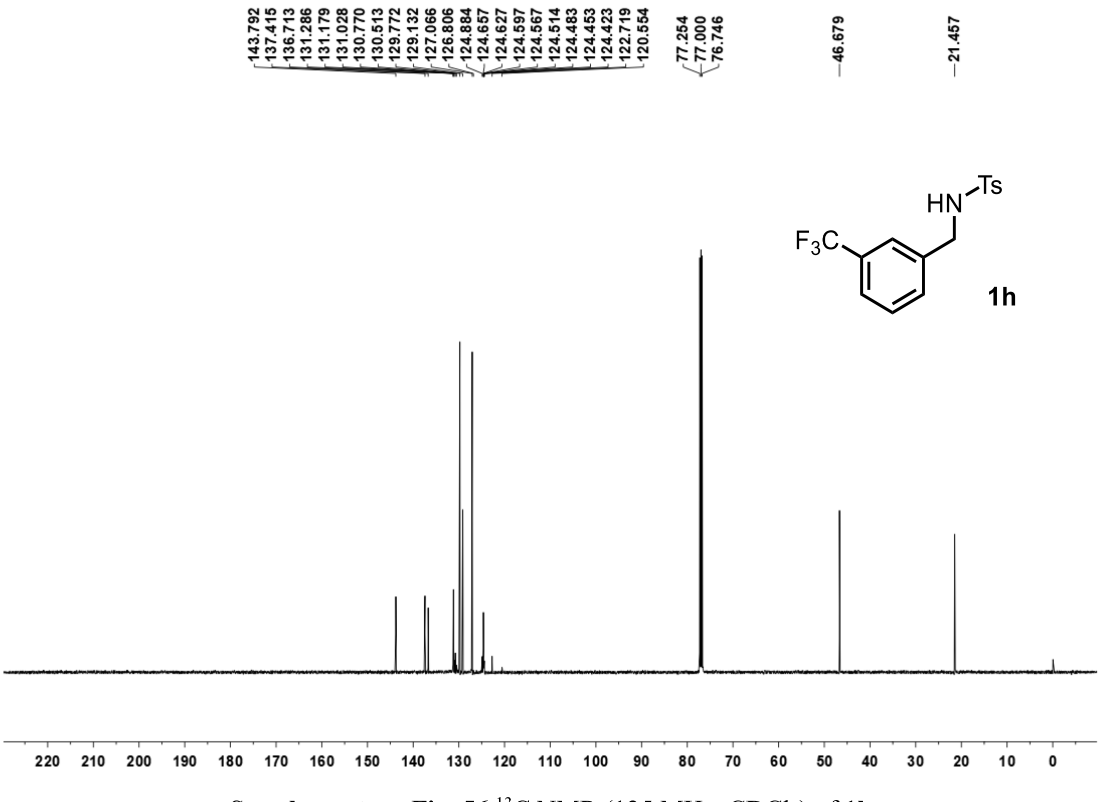

Supplementary Fig. 56 ^{13}C NMR (125 MHz, $CDCl_3$) of **1h**

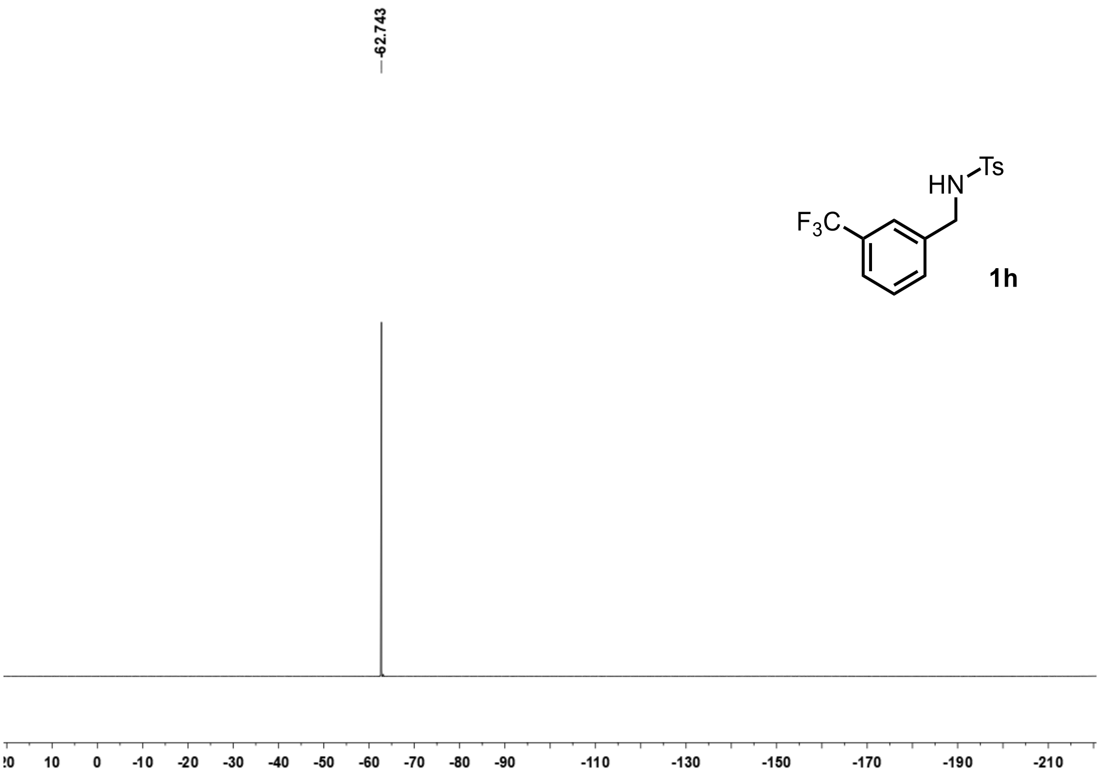

Supplementary Fig. 57 ^{19}F NMR (470 MHz, $CDCl_3$) of **1h**

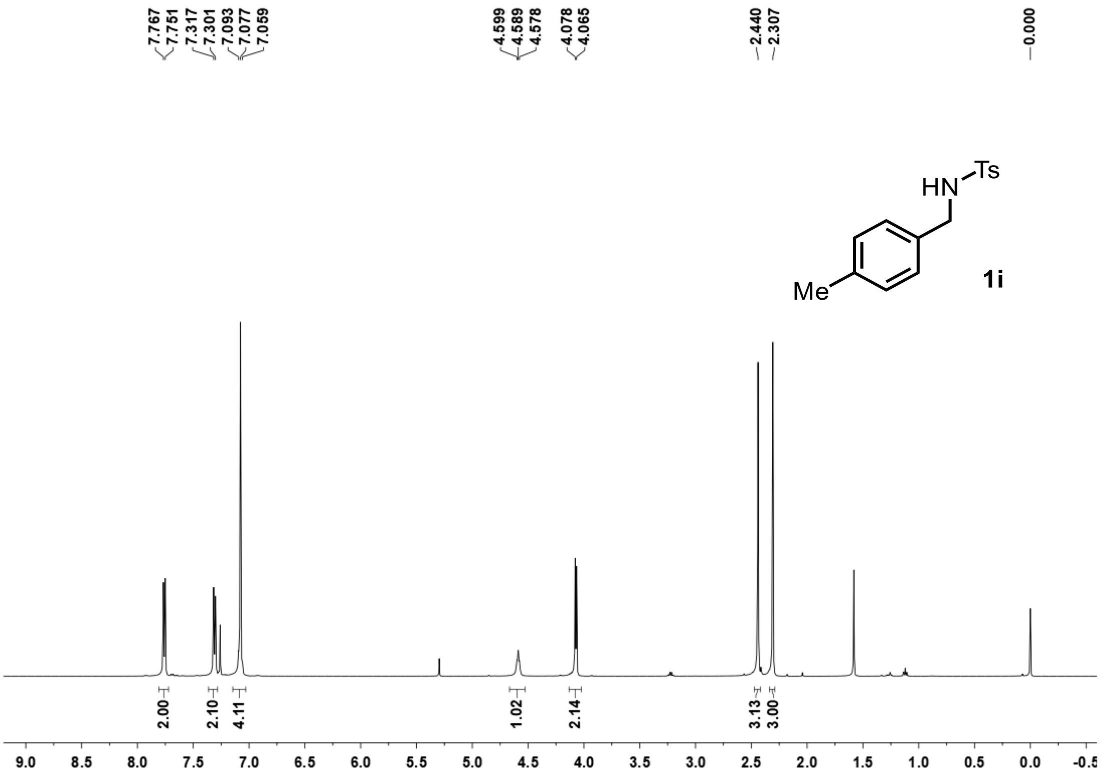

1i

Supplementary Fig. 58 ^{1}H NMR (500 MHz, $CDCl_3$) of **1i**

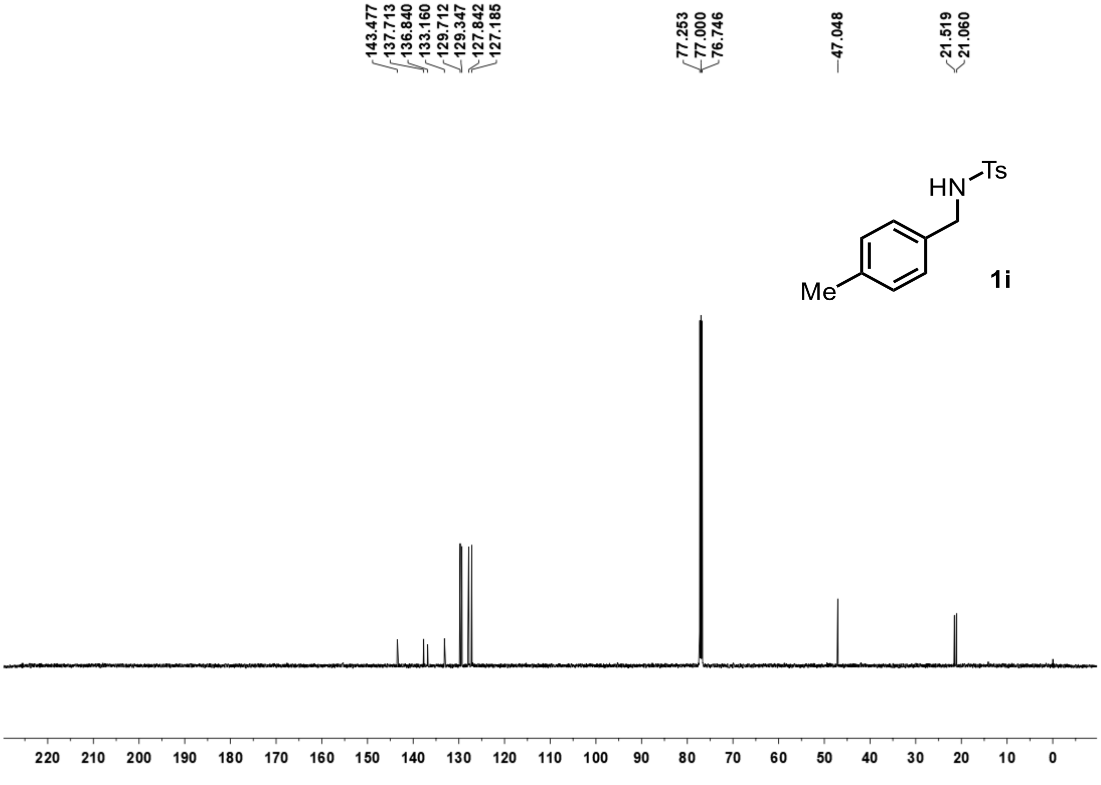

1i

Supplementary Fig. 59 ^{13}C NMR (125 MHz, $CDCl_3$) of **1i**

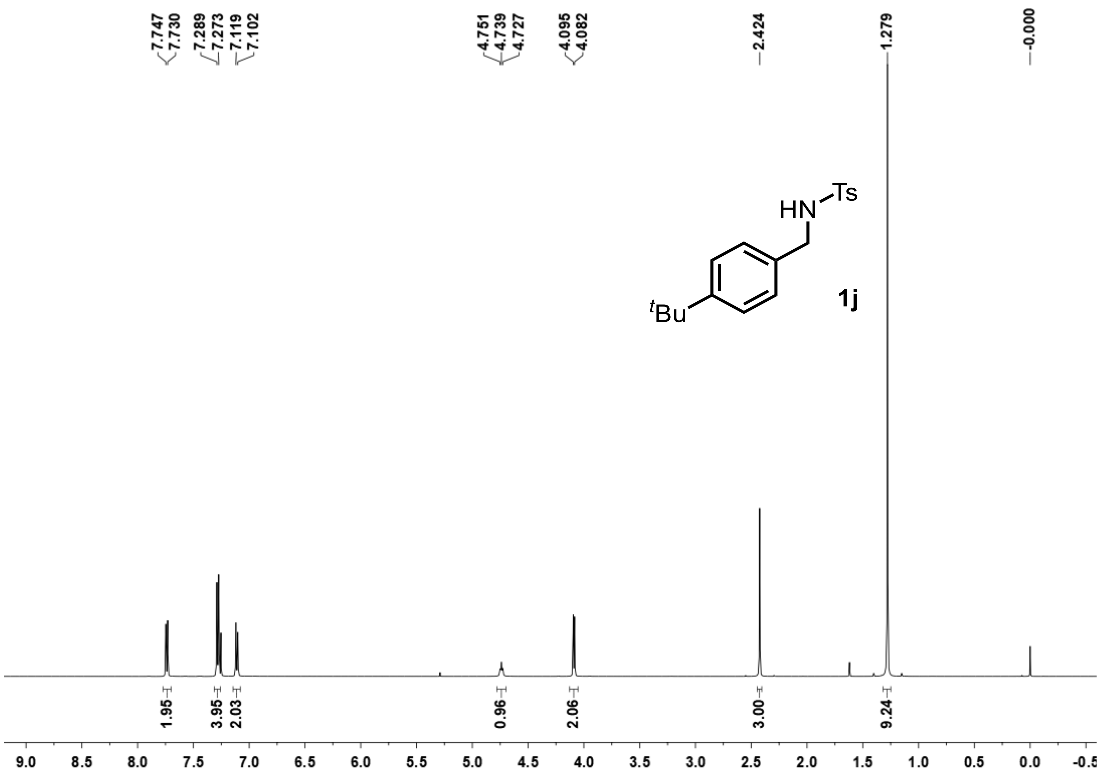

Supplementary Fig. 60 ^{1}H NMR (500 MHz, $CDCl_3$) of **1j**

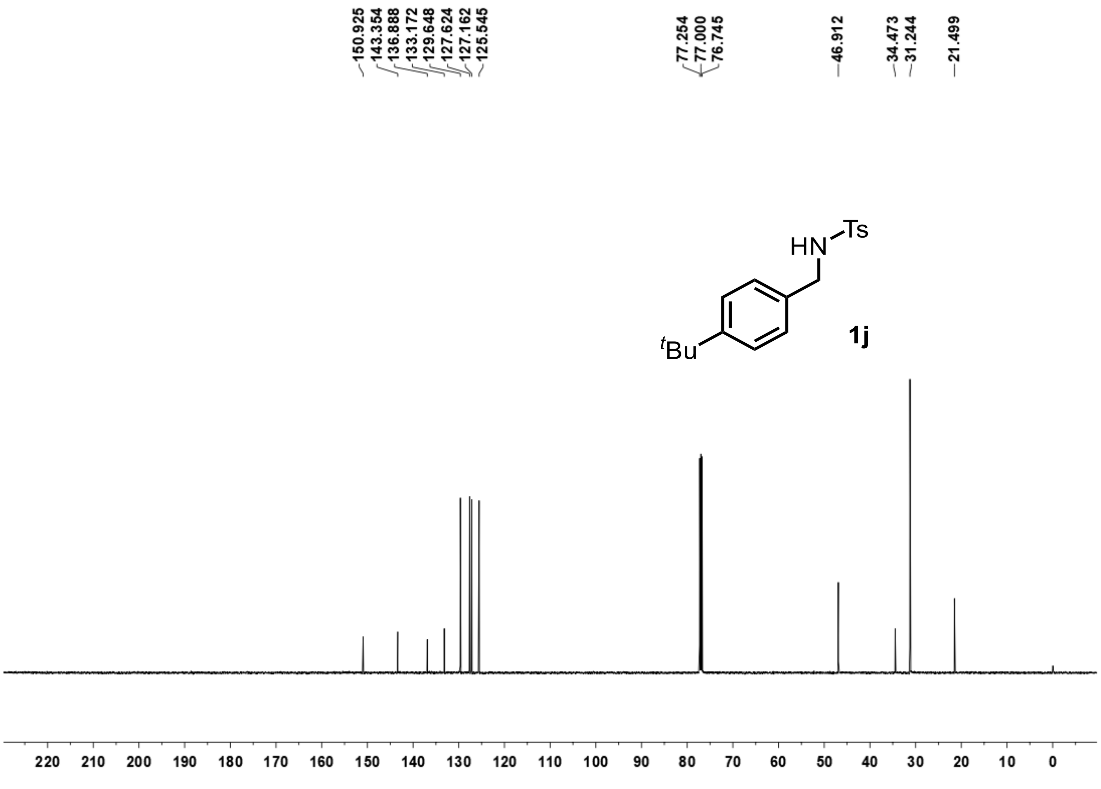

Supplementary Fig. 61 ^{13}C NMR (125 MHz, $CDCl_3$) of **1j**

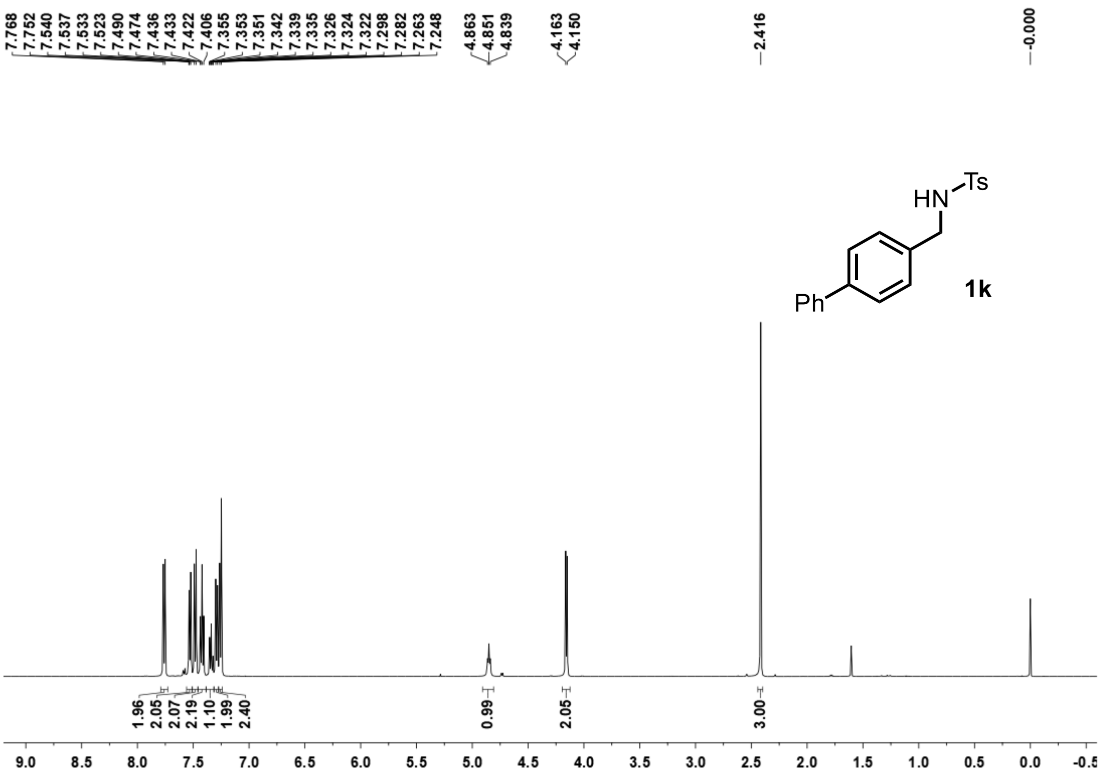

Supplementary Fig. 62 ^{1}H NMR (500 MHz, $CDCl_3$) of **1k**

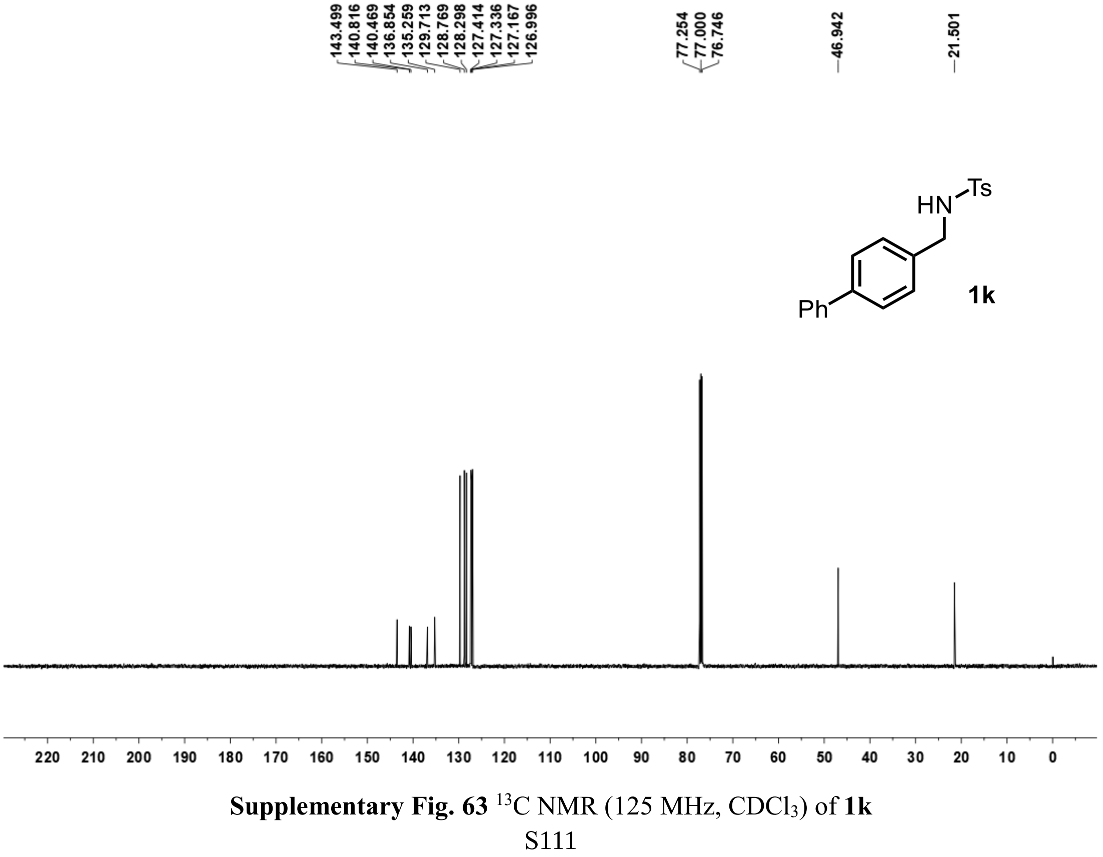

Supplementary Fig. 63 ^{13}C NMR (125 MHz, $CDCl_3$) of **1k**

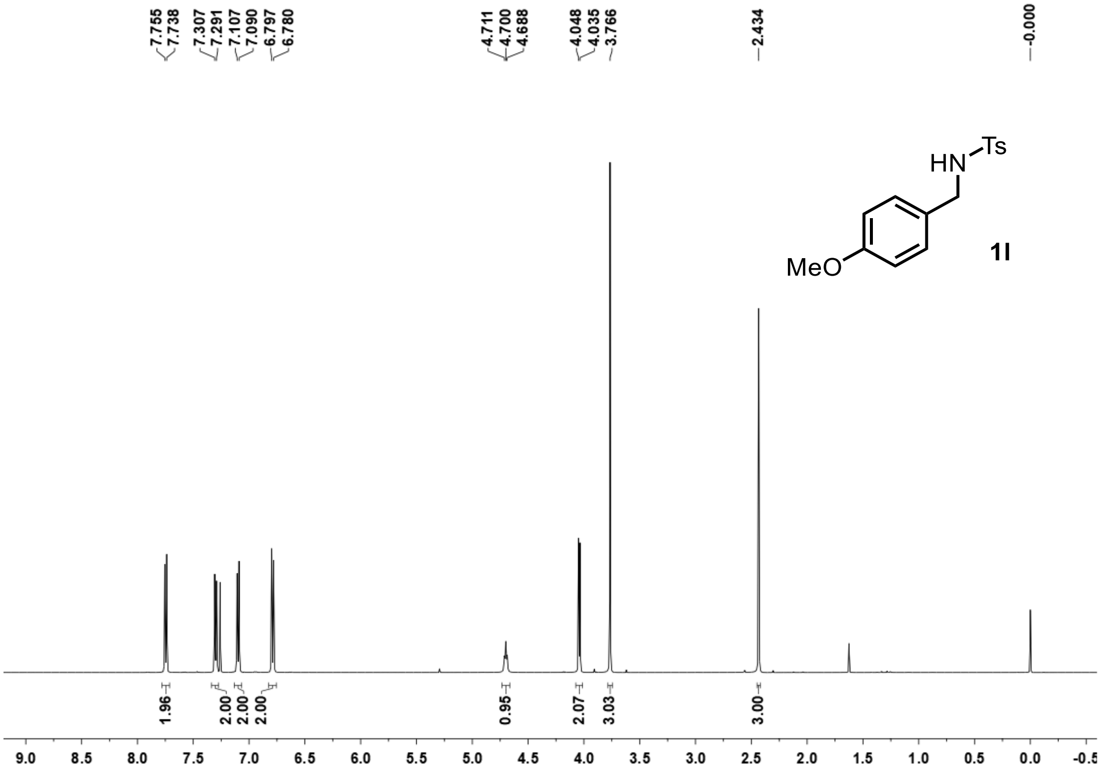

Supplementary Fig. 64 ^{1}H NMR (500 MHz, $CDCl_3$) of **1l**

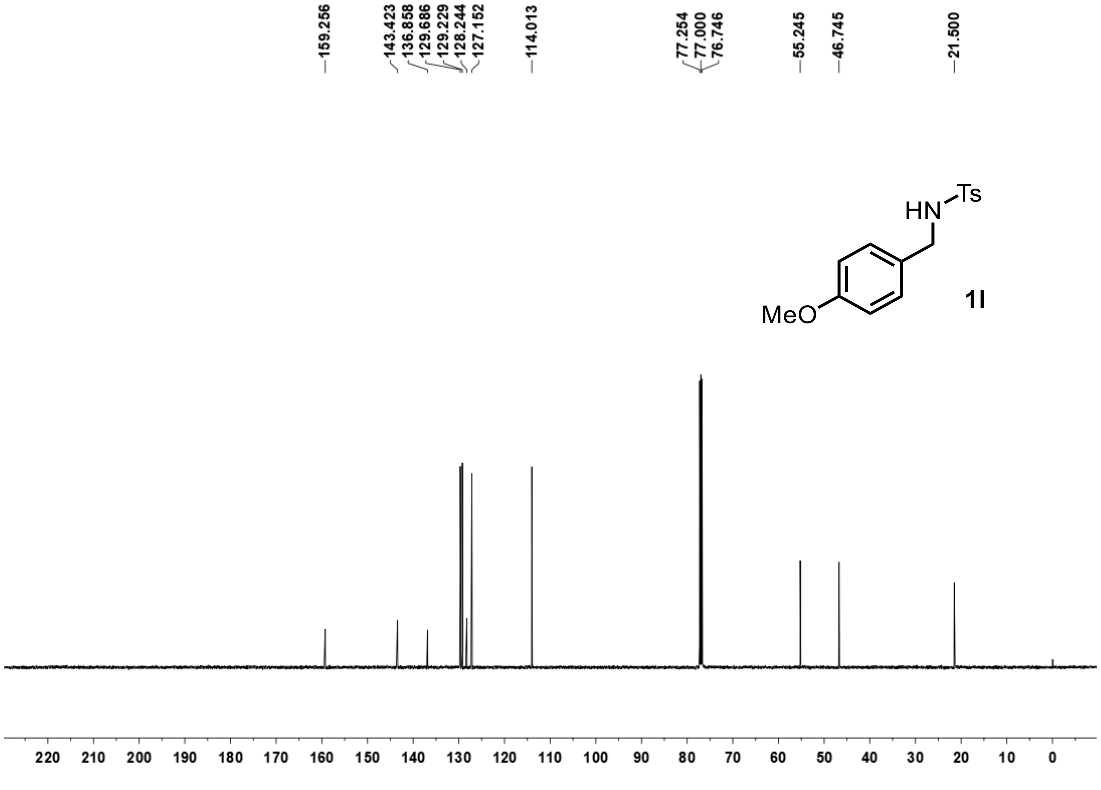

Supplementary Fig. 65 ^{13}C NMR (125 MHz, $CDCl_3$) of **1l**

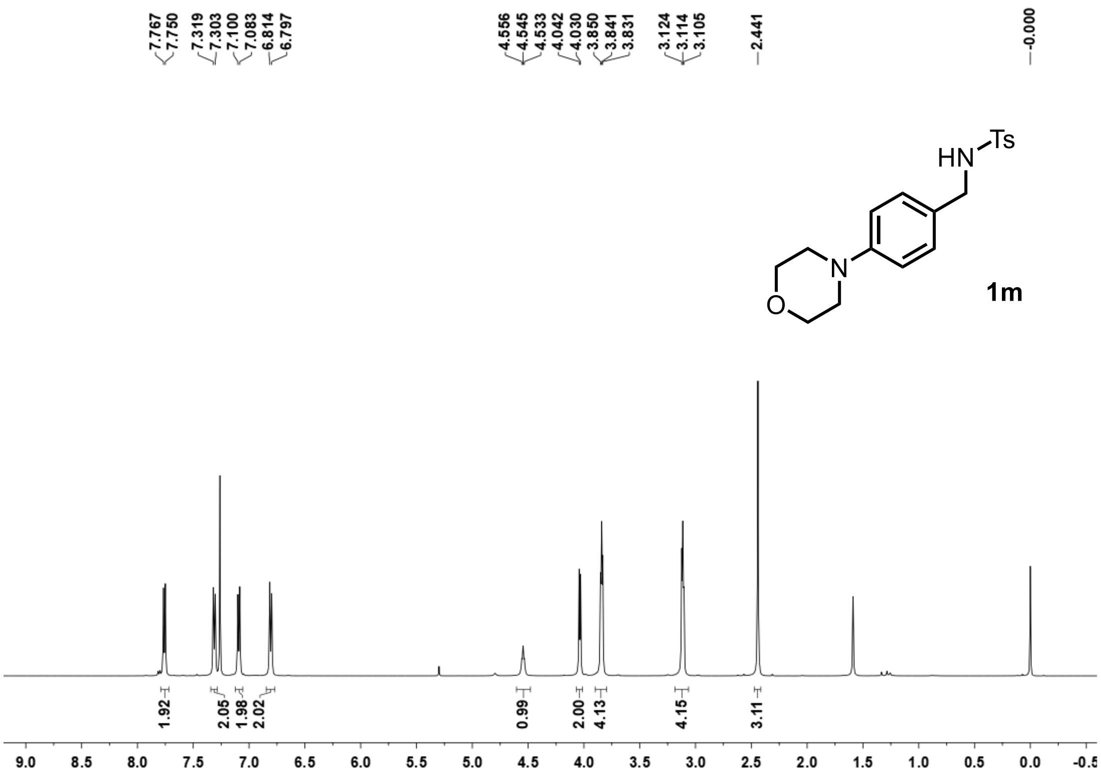

Supplementary Fig. 66 ^{1}H NMR (500 MHz, $CDCl_3$) of **1m**

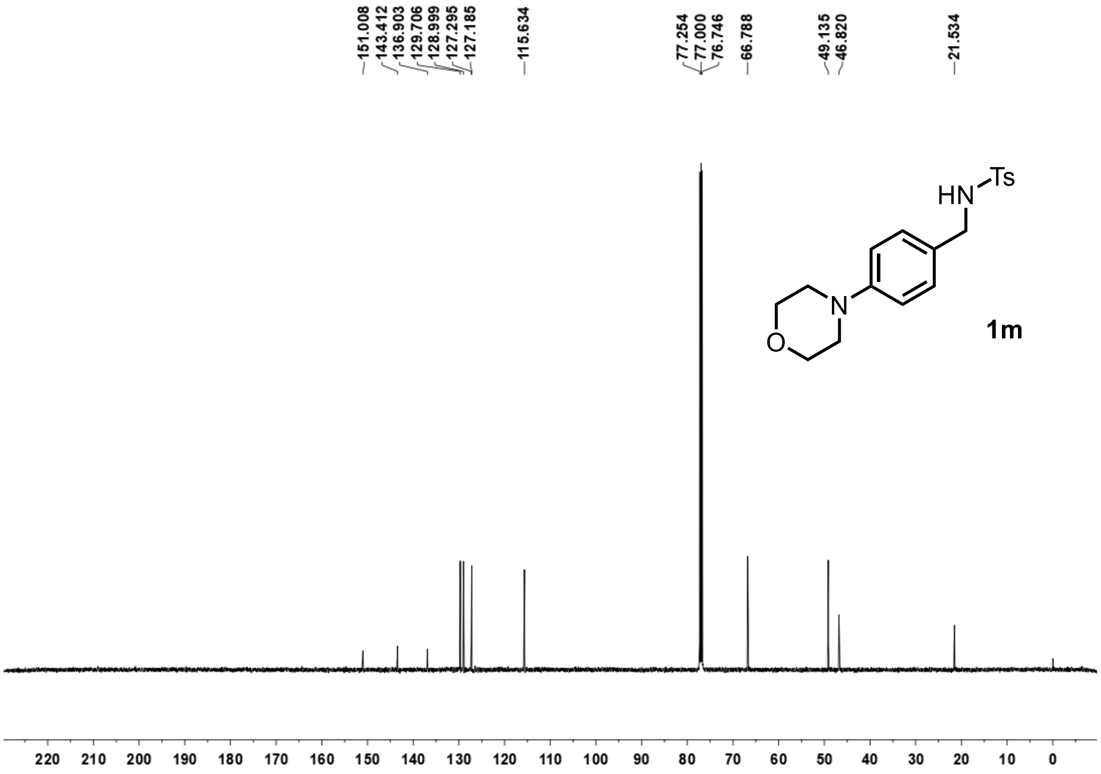

Supplementary Fig. 67 ^{13}C NMR (125 MHz, $CDCl_3$) of **1m**

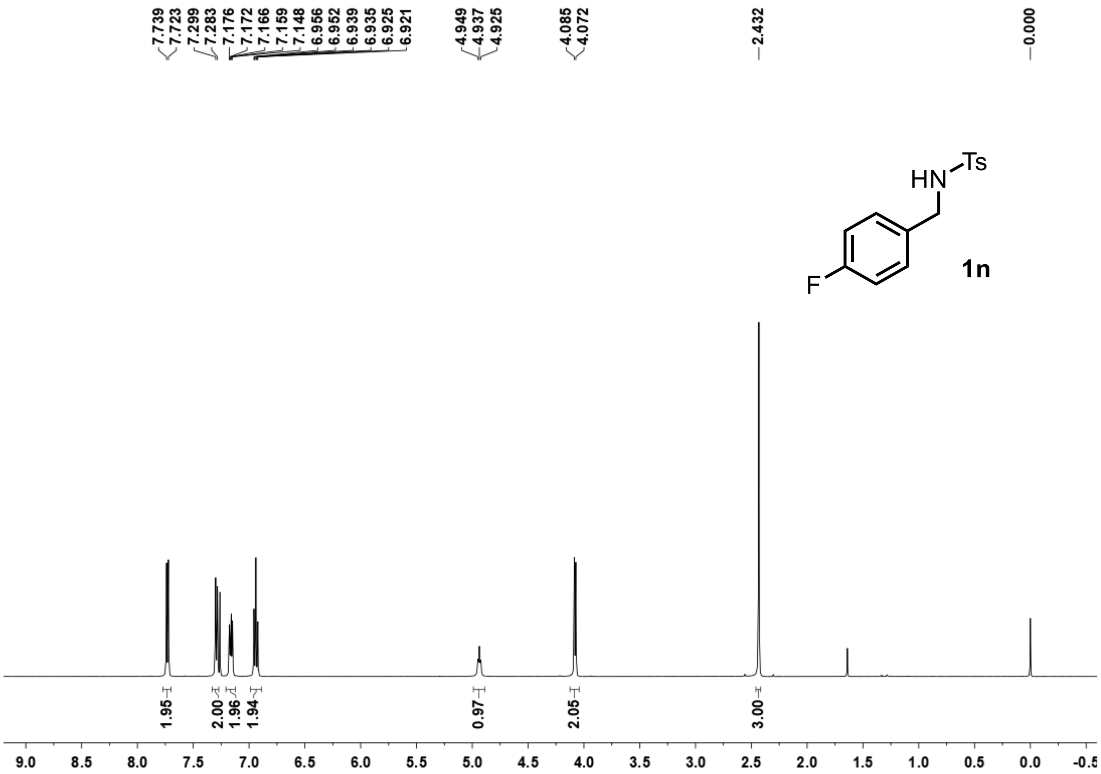

Supplementary Fig. 68 ^{1}H NMR (500 MHz, $CDCl_3$) of **1n**

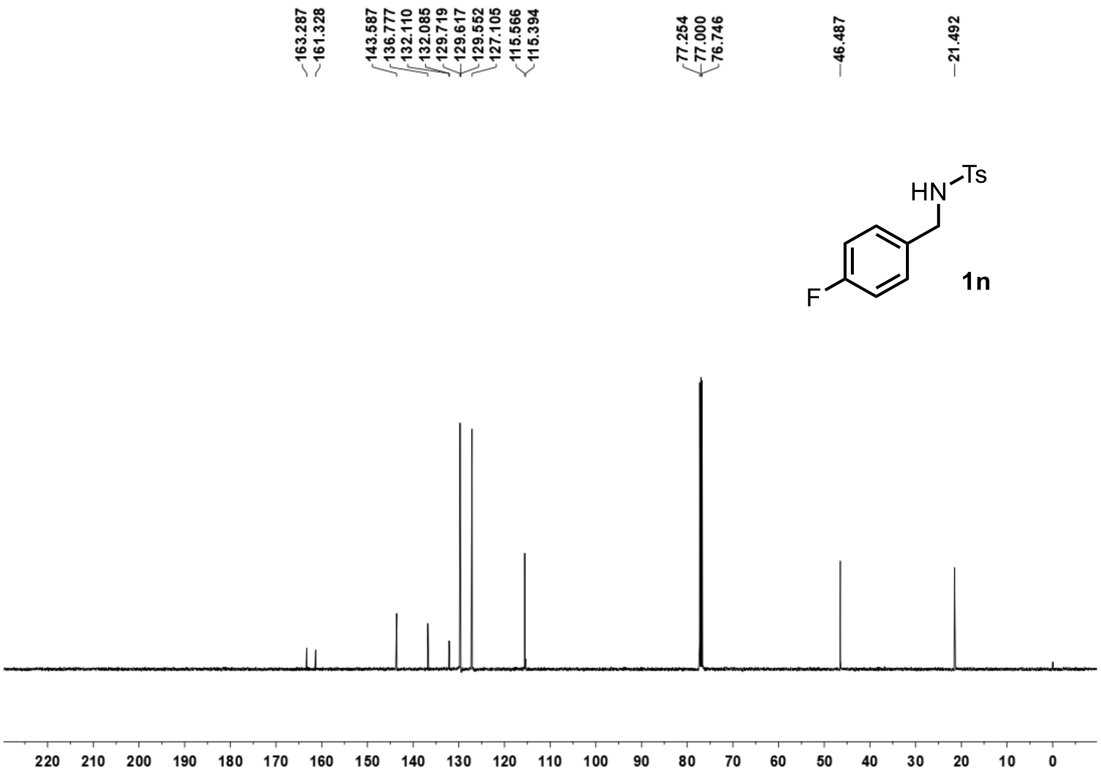

Supplementary Fig. 69 ^{13}C NMR (125 MHz, $CDCl_3$) of **1n**

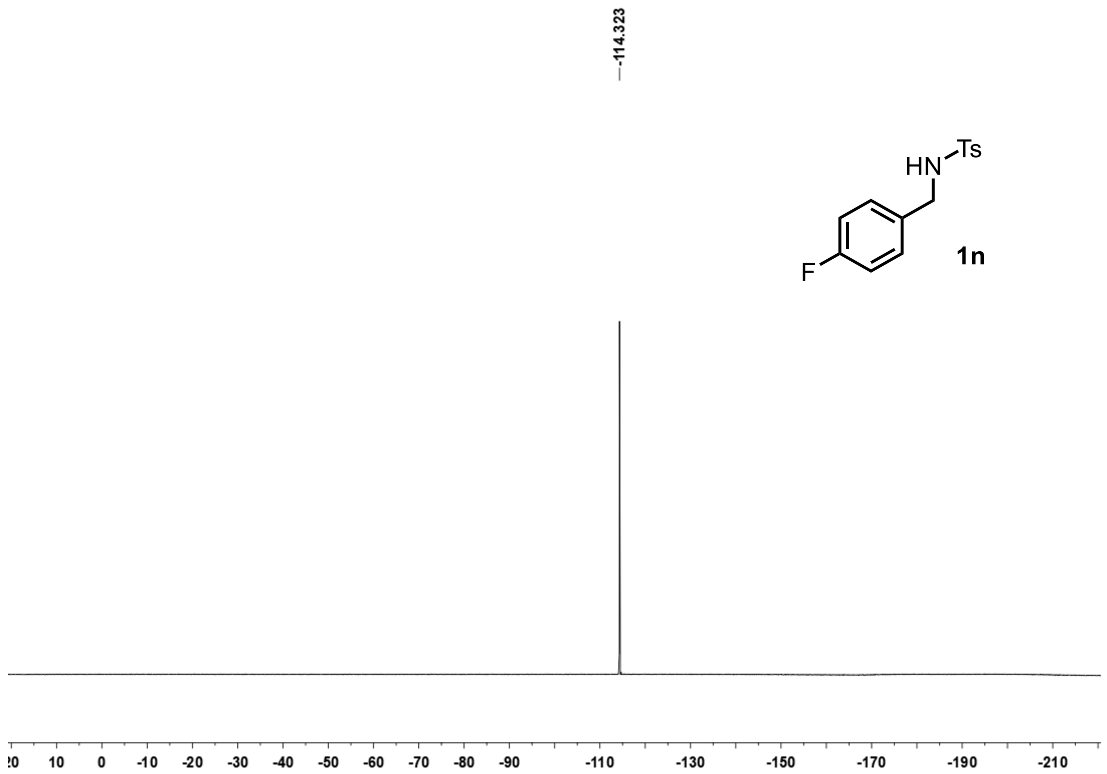

Supplementary Fig. 70 ^{19}F NMR (470 MHz, $CDCl_3$) of **1n**

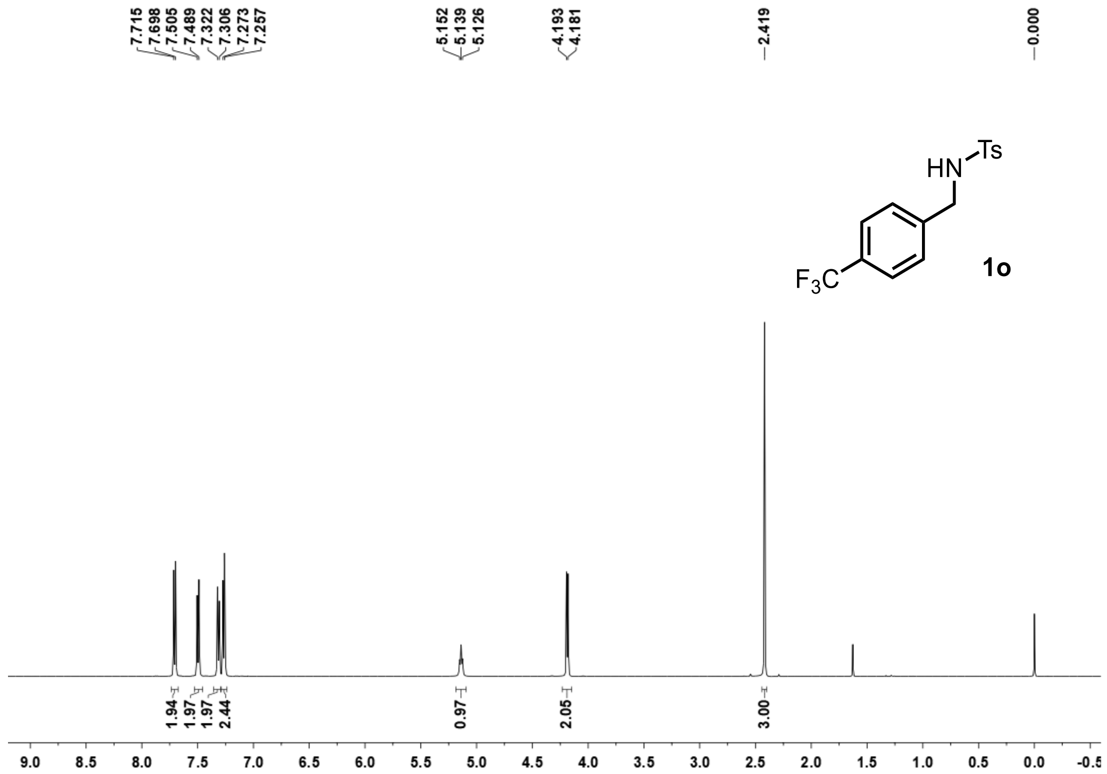

Supplementary Fig. 71 ^{1}H NMR (500 MHz, $CDCl_3$) of **1o**

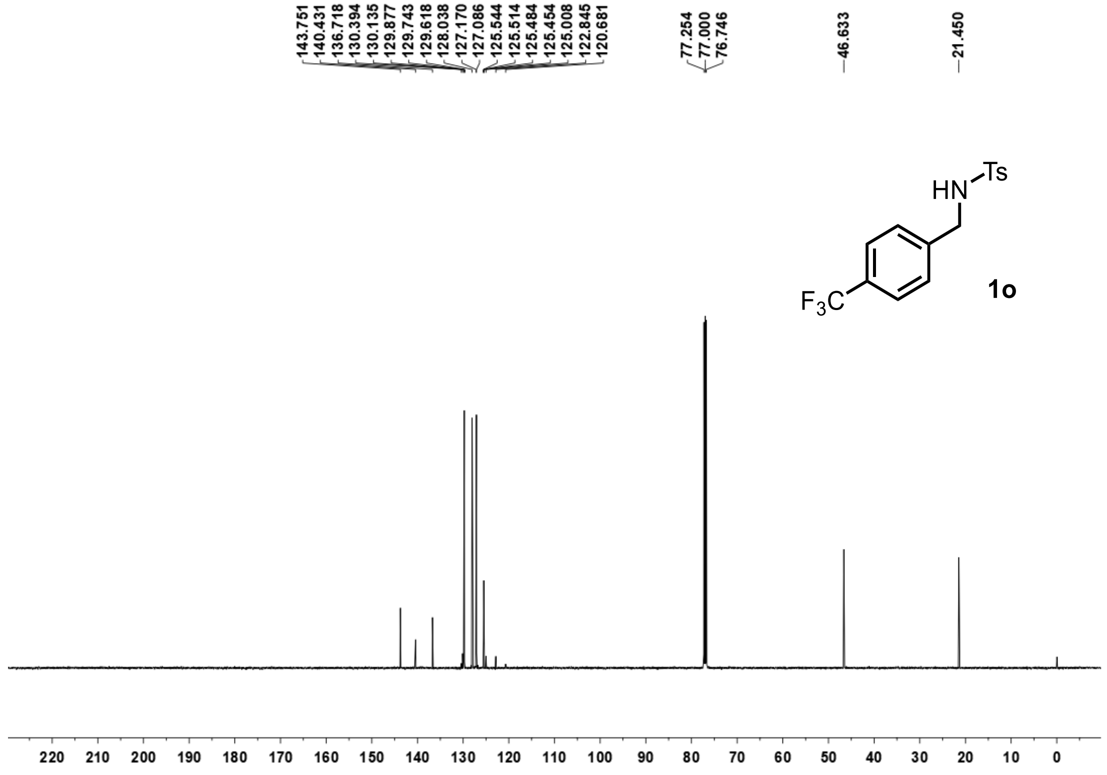

Supplementary Fig. 72 ^{13}C NMR (125 MHz, $CDCl_3$) of **1o**

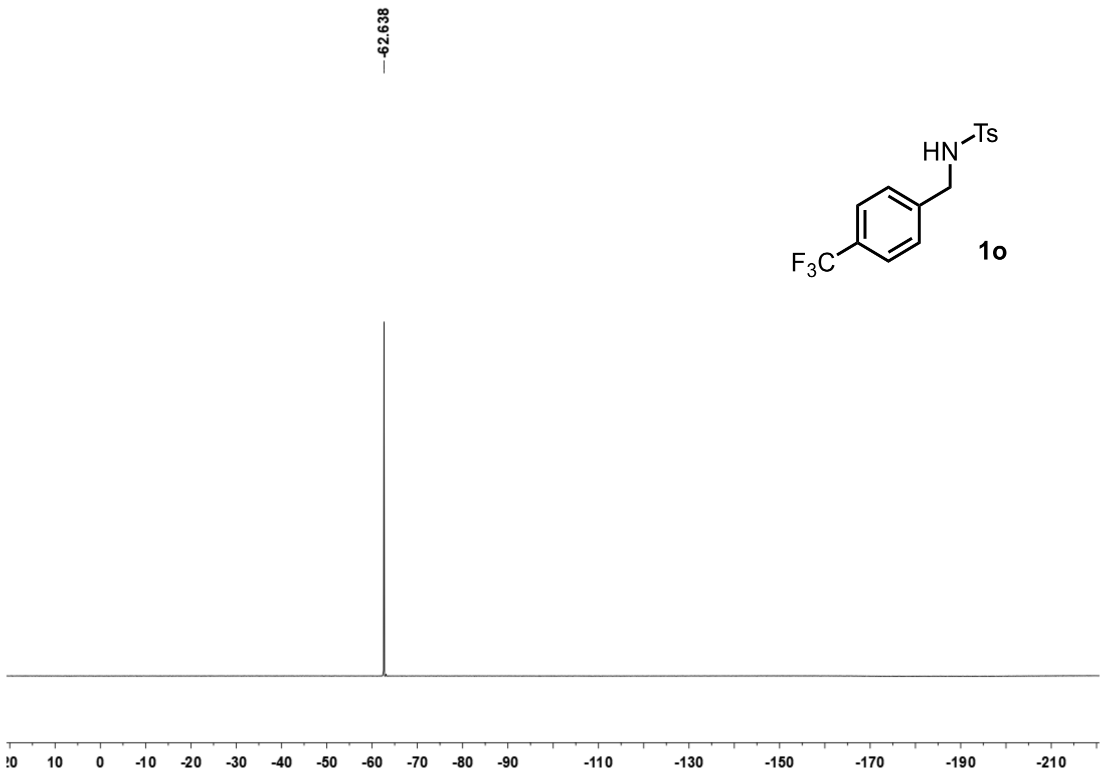

Supplementary Fig. 73 ^{19}F NMR (470 MHz, $CDCl_3$) of **1o**

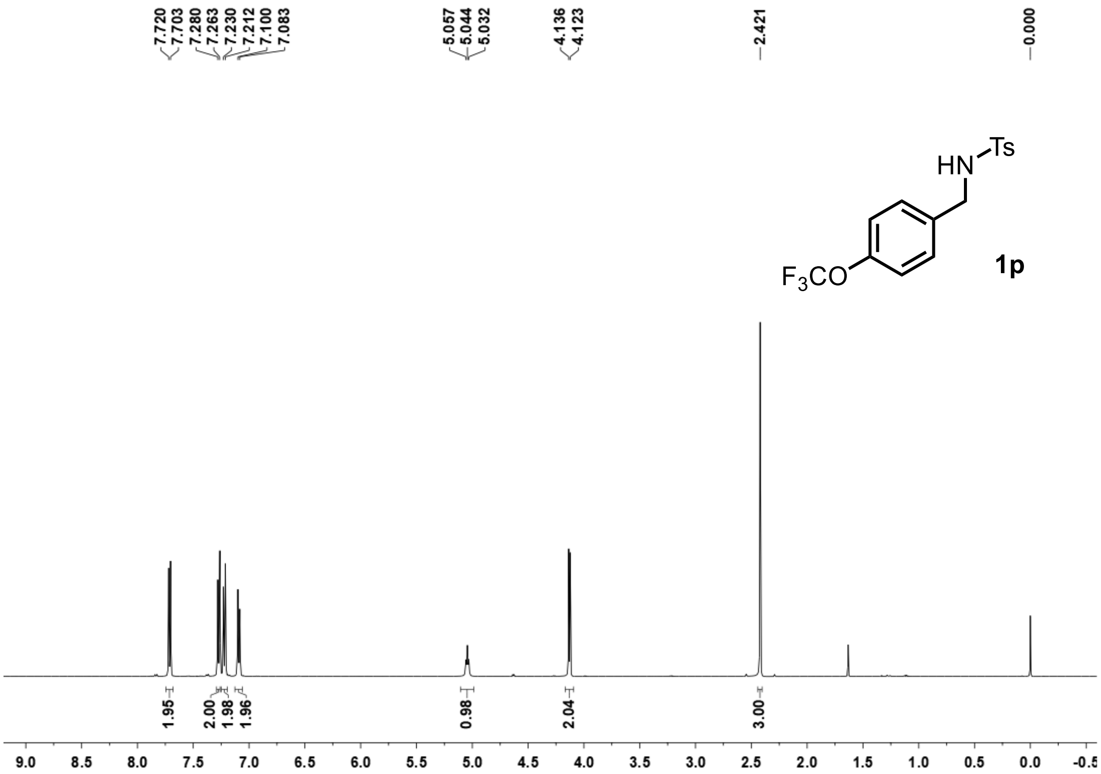

Supplementary Fig. 74 ^{1}H NMR (500 MHz, $CDCl_3$) of **1p**

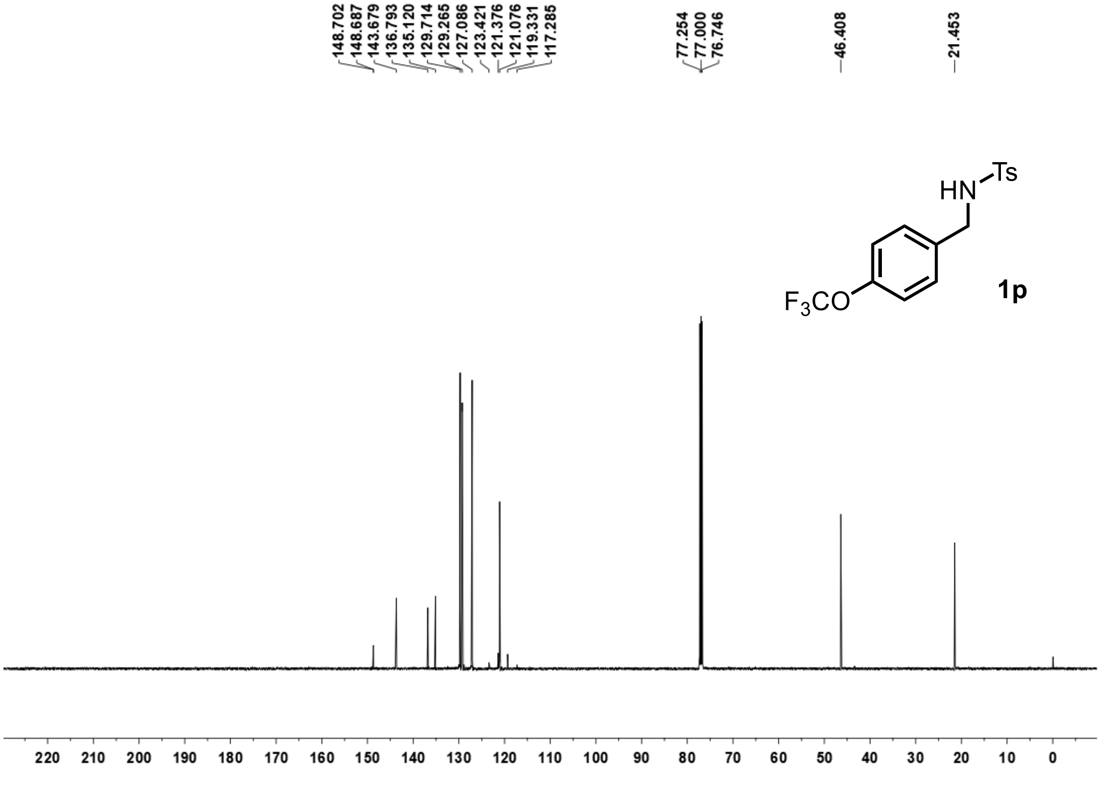

Supplementary Fig. 75 ^{13}C NMR (125 MHz, $CDCl_3$) of **1p**

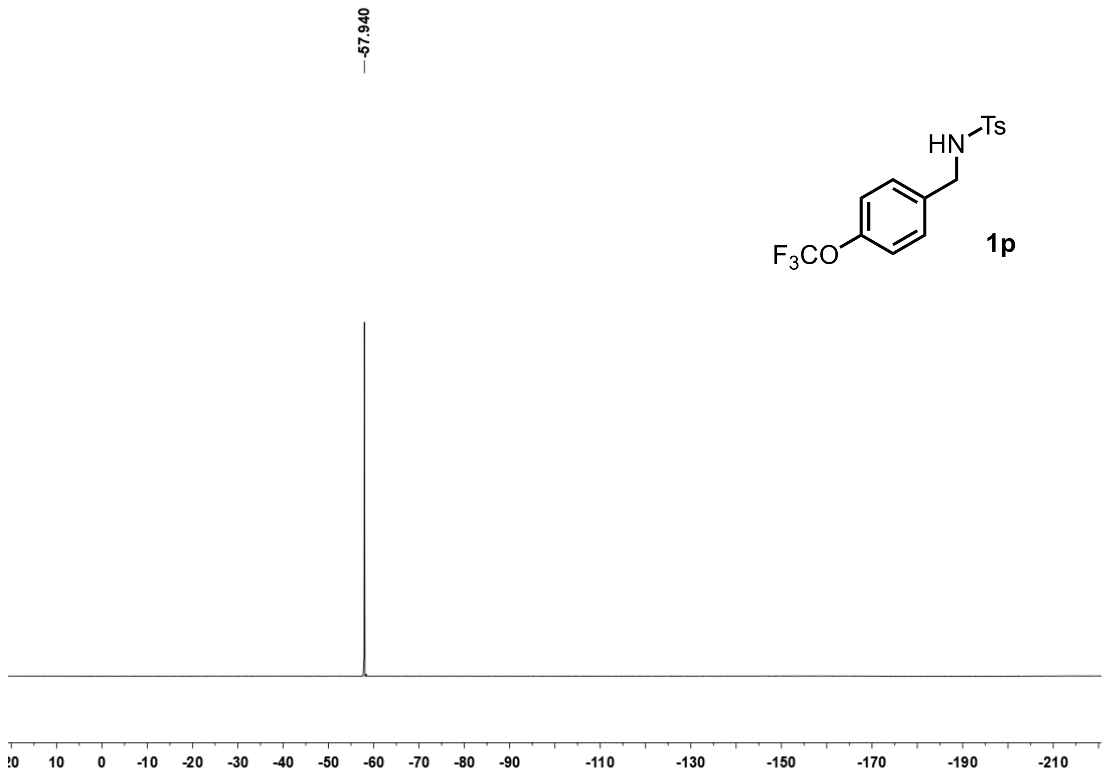

Supplementary Fig. 76 ^{19}F NMR (470 MHz, $CDCl_3$) of **1p**

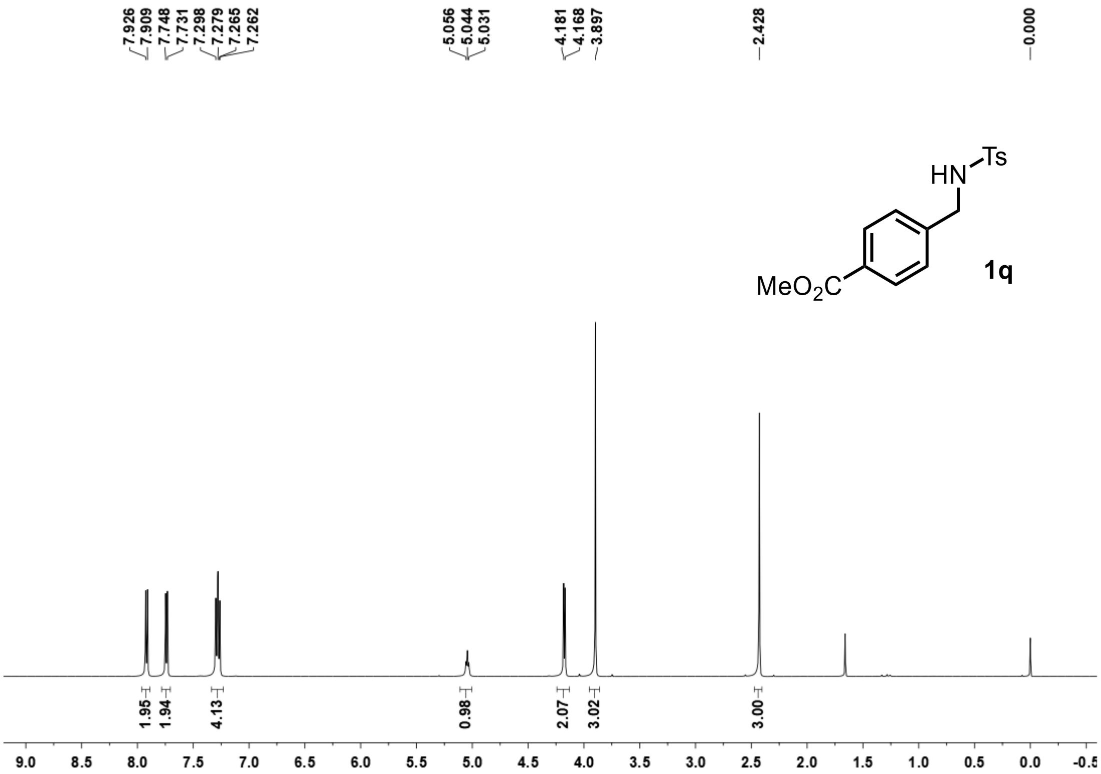

Supplementary Fig. 77 ^{1}H NMR (500 MHz, CDCl$_3$) of **1q**

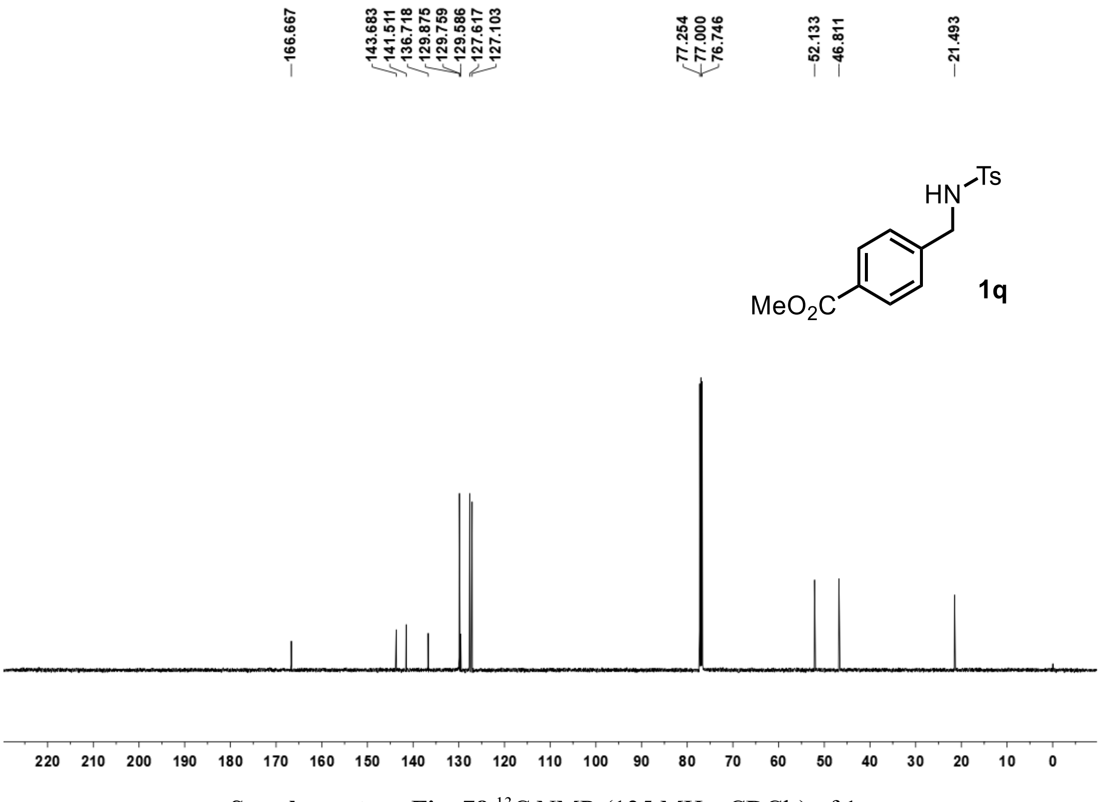

Supplementary Fig. 78 ^{13}C NMR (125 MHz, CDCl$_3$) of **1q**

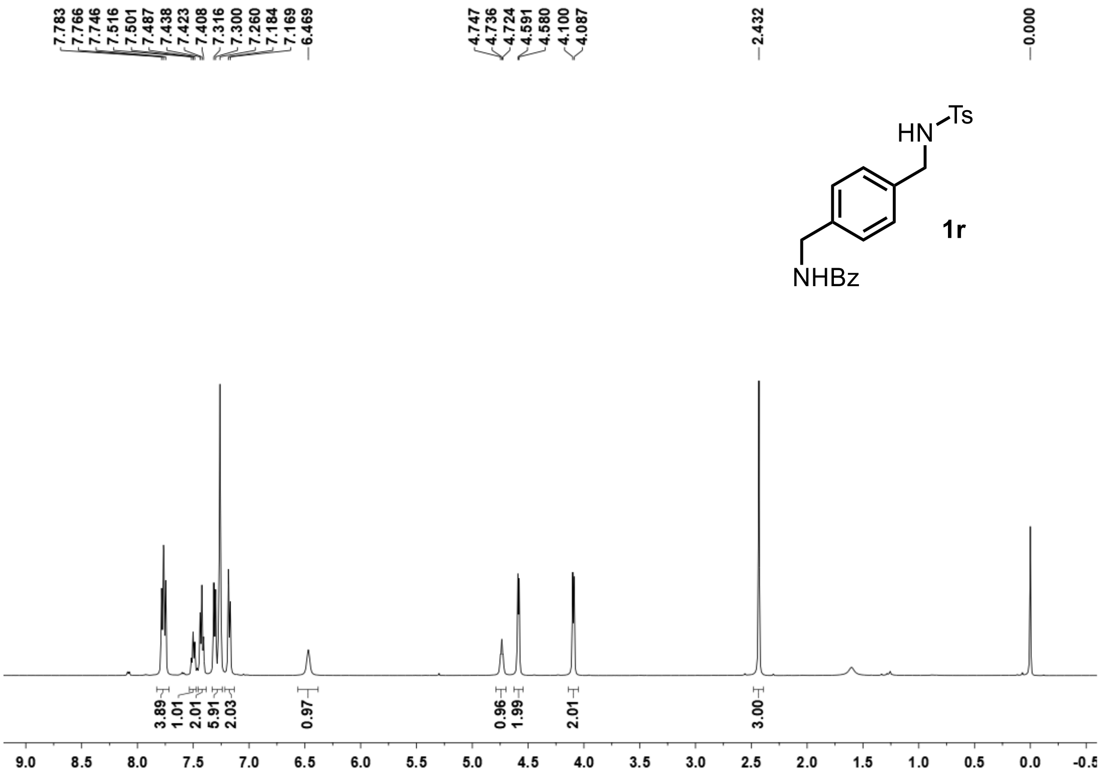

Supplementary Fig. 79 ^{1}H NMR (500 MHz, $CDCl_3$) of **1r**

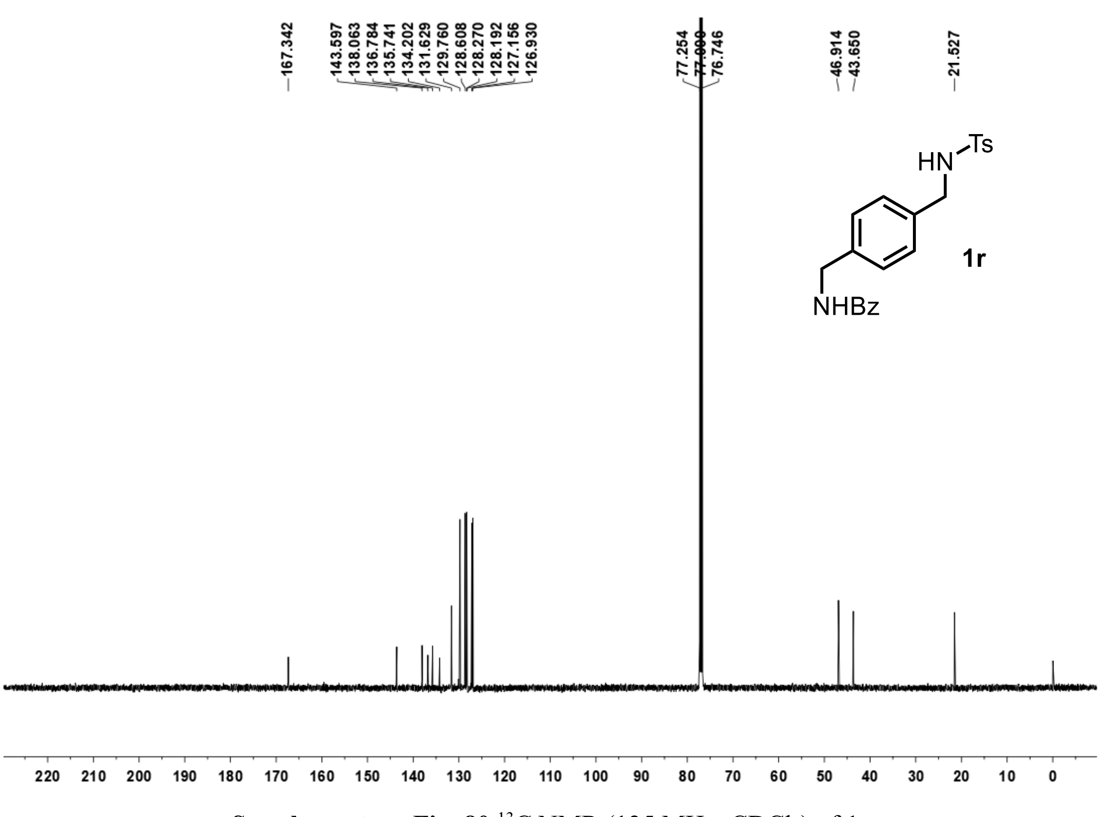

Supplementary Fig. 80 ^{13}C NMR (125 MHz, $CDCl_3$) of **1r**

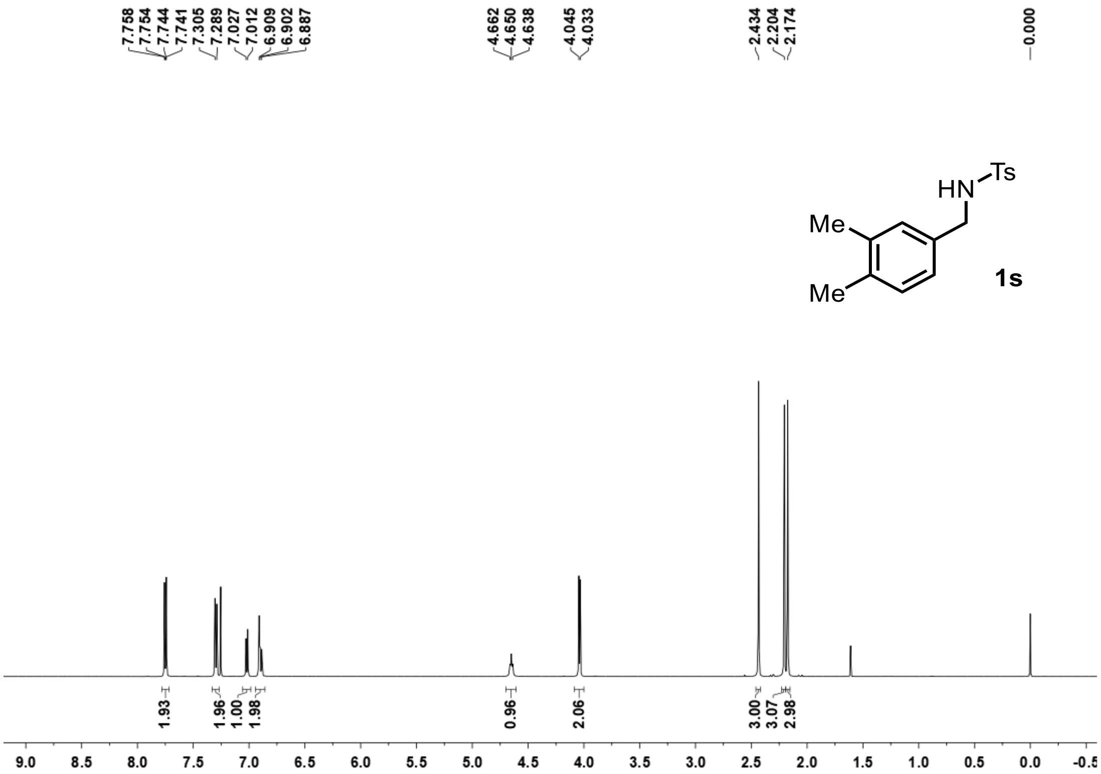

Supplementary Fig. 81 ^{1}H NMR (500 MHz, $CDCl_3$) of **1s**

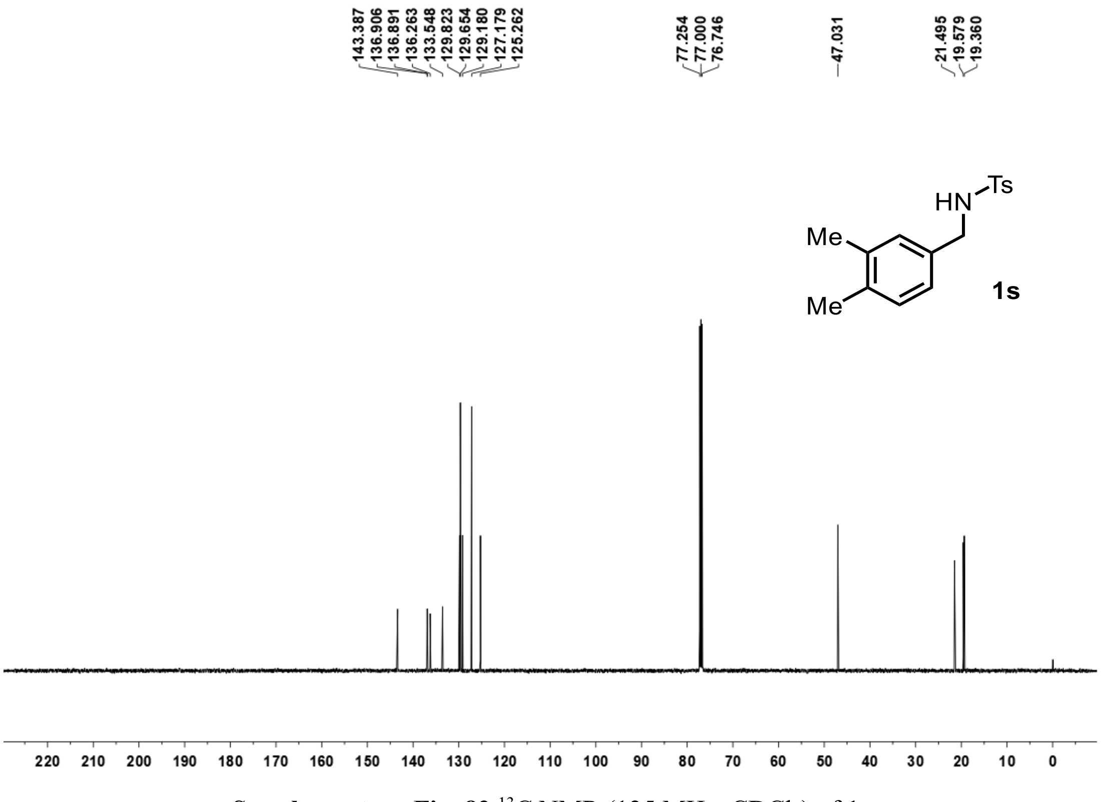

Supplementary Fig. 82 ^{13}C NMR (125 MHz, $CDCl_3$) of **1s**

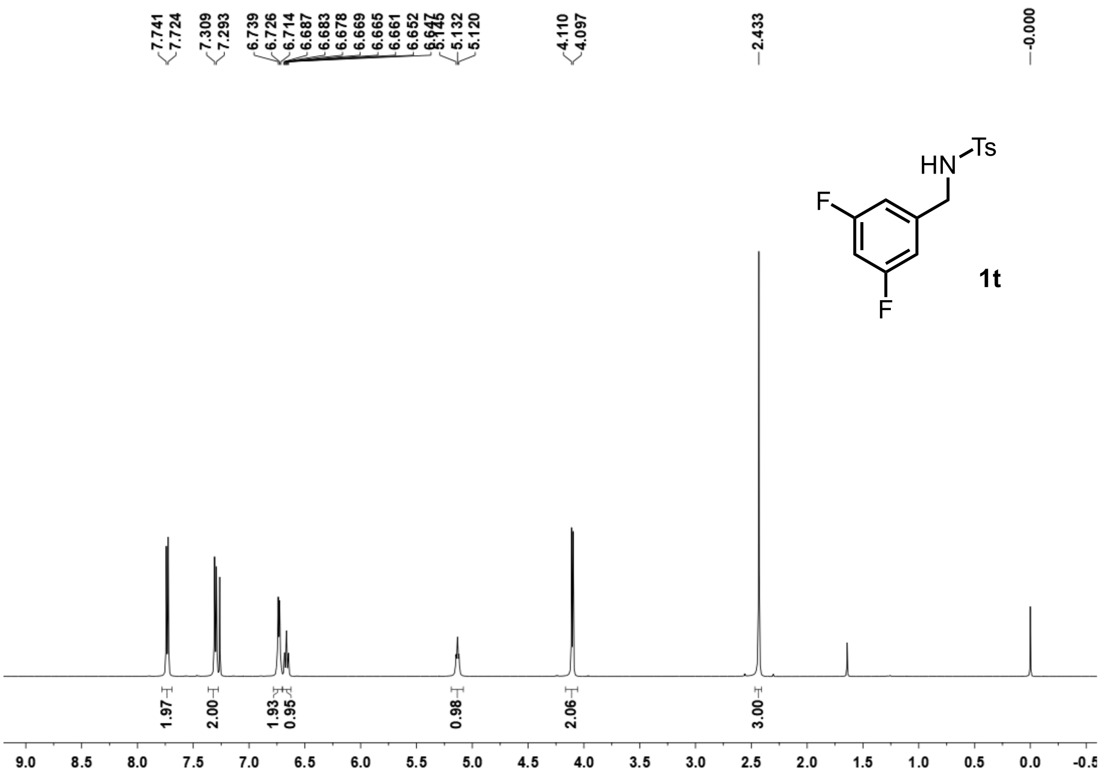

Supplementary Fig. 83 ^{1}H NMR (500 MHz, $CDCl_3$) of **1t**

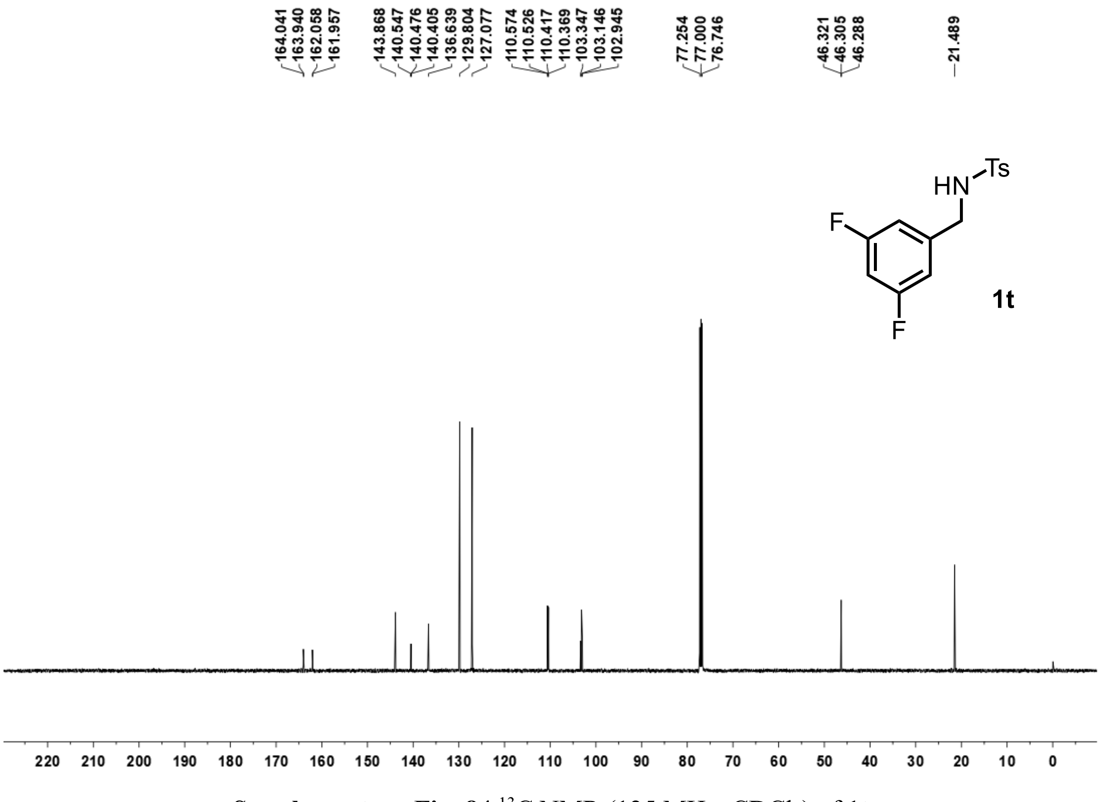

Supplementary Fig. 84 ^{13}C NMR (125 MHz, $CDCl_3$) of **1t**

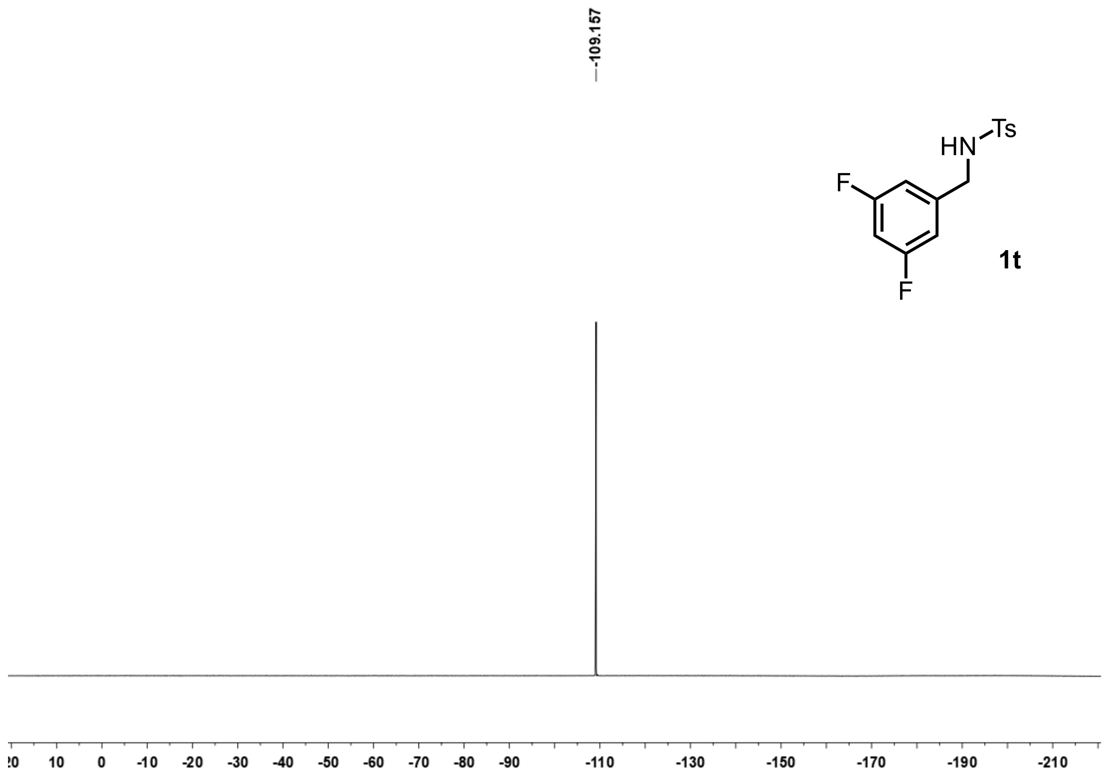

Supplementary Fig. 85 ^{19}F NMR (470 MHz, $CDCl_3$) of **1t**

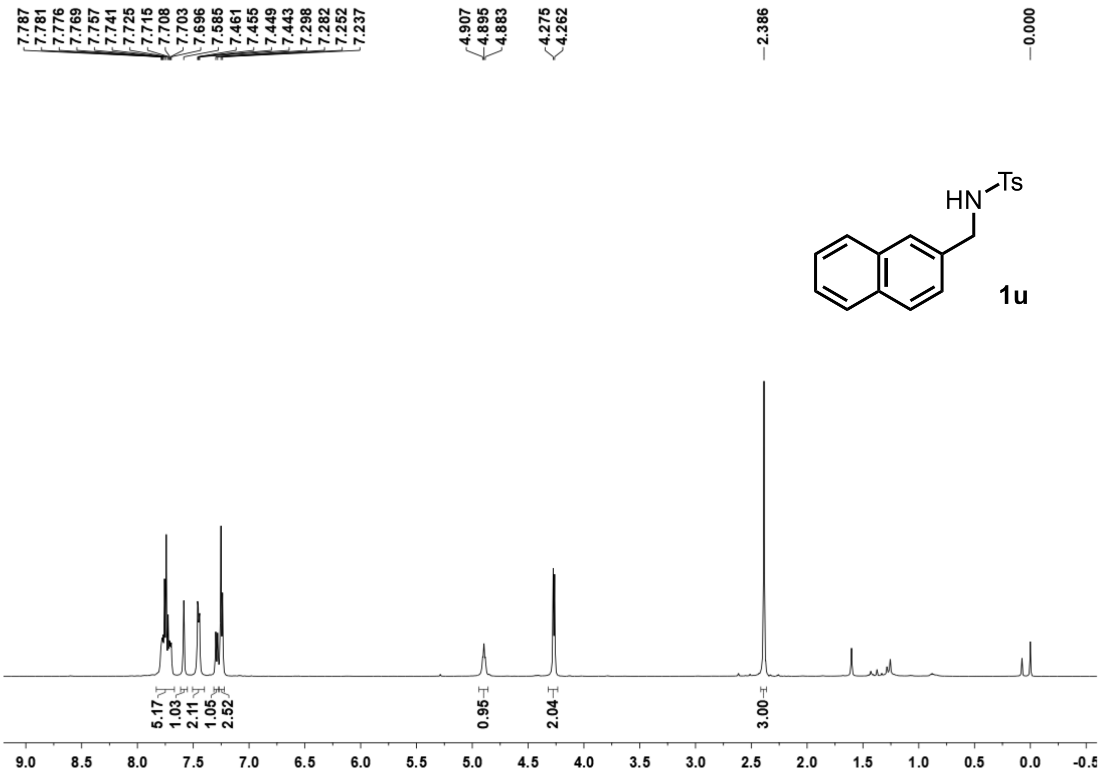

Supplementary Fig. 86 ^{1}H NMR (500 MHz, $CDCl_3$) of **1u**

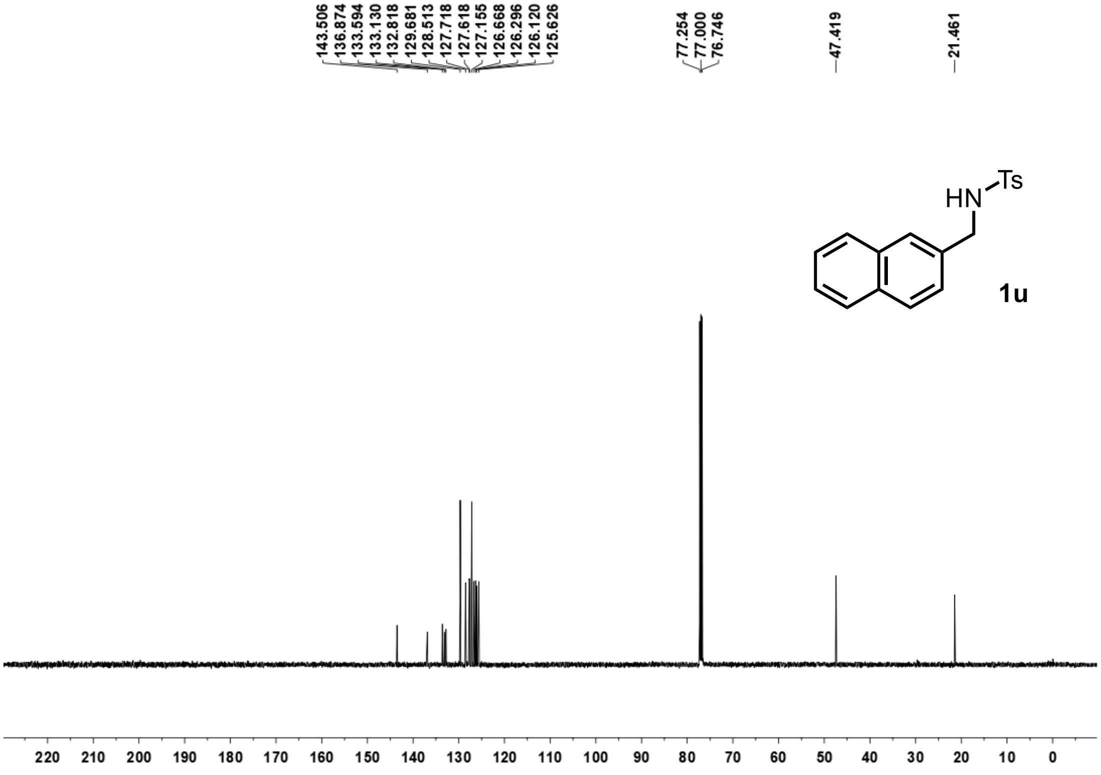

Supplementary Fig. 87 ^{13}C NMR (125 MHz, $CDCl_3$) of **1u**

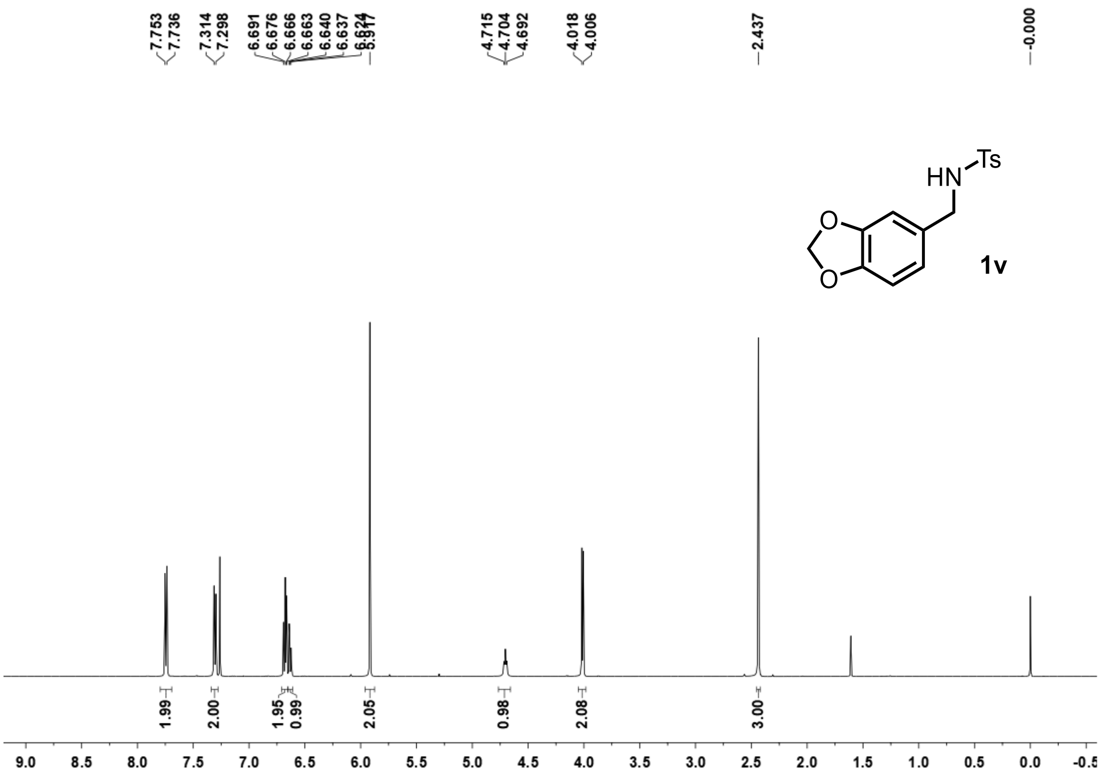

Supplementary Fig. 88 ^{1}H NMR (500 MHz, $CDCl_3$) of **1v**

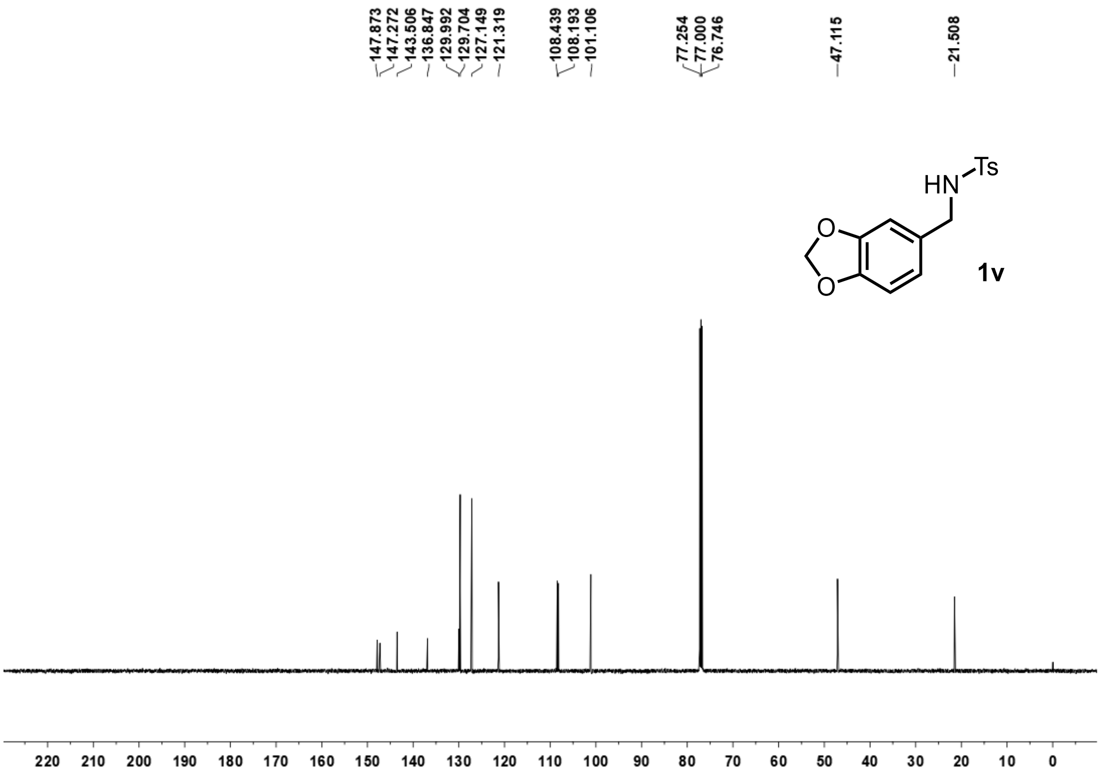

Supplementary Fig. 89 ^{13}C NMR (125 MHz, $CDCl_3$) of **1v**

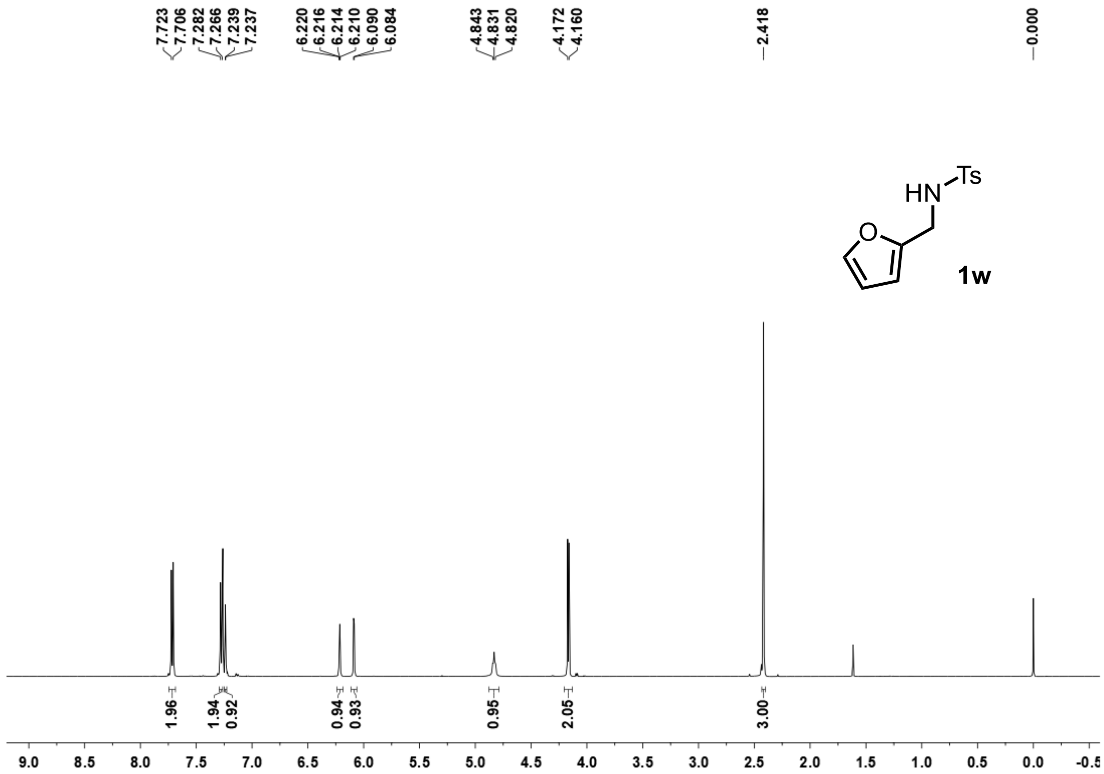

Supplementary Fig. 90 ^{1}H NMR (500 MHz, $CDCl_3$) of **1w**

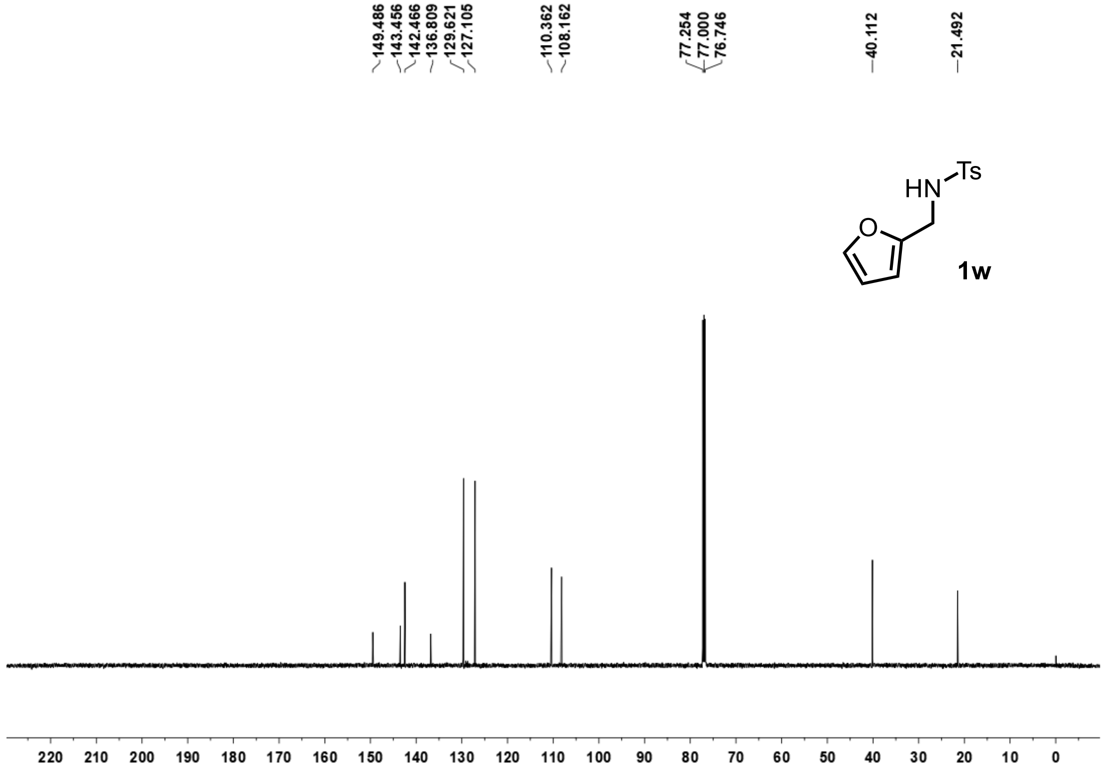

Supplementary Fig. 91 ^{13}C NMR (125 MHz, $CDCl_3$) of **1w**

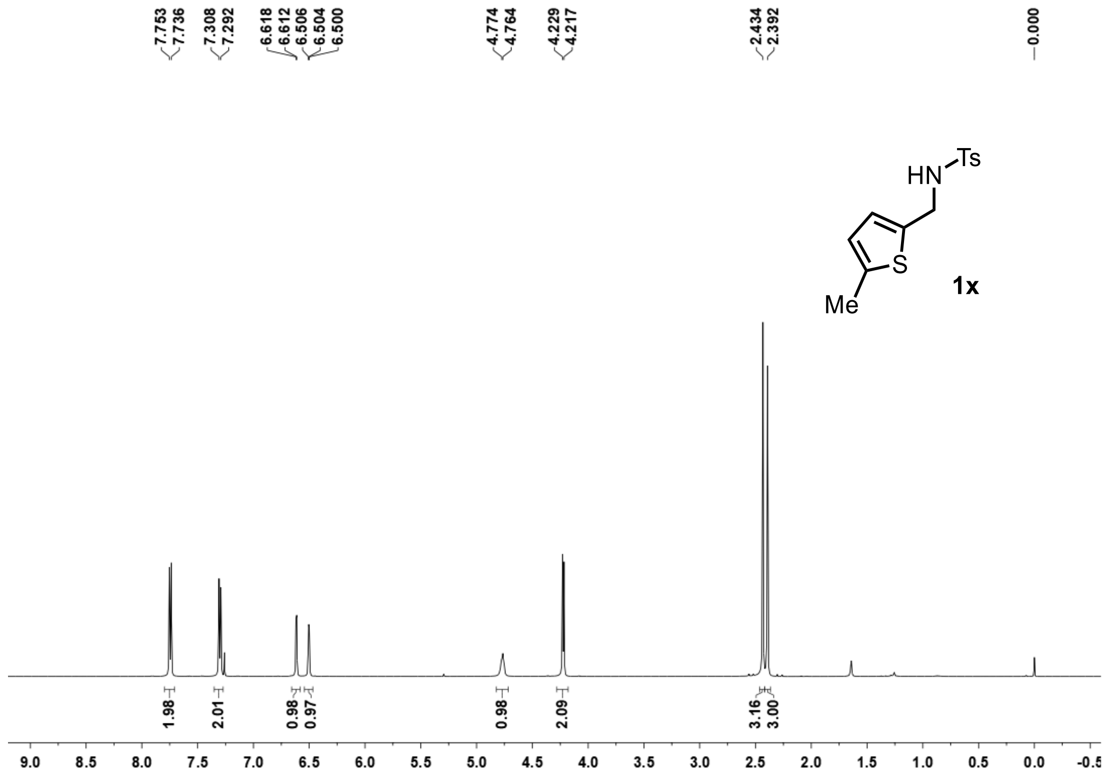

Supplementary Fig. 92 ^{1}H NMR (500 MHz, $CDCl_3$) of **1x**

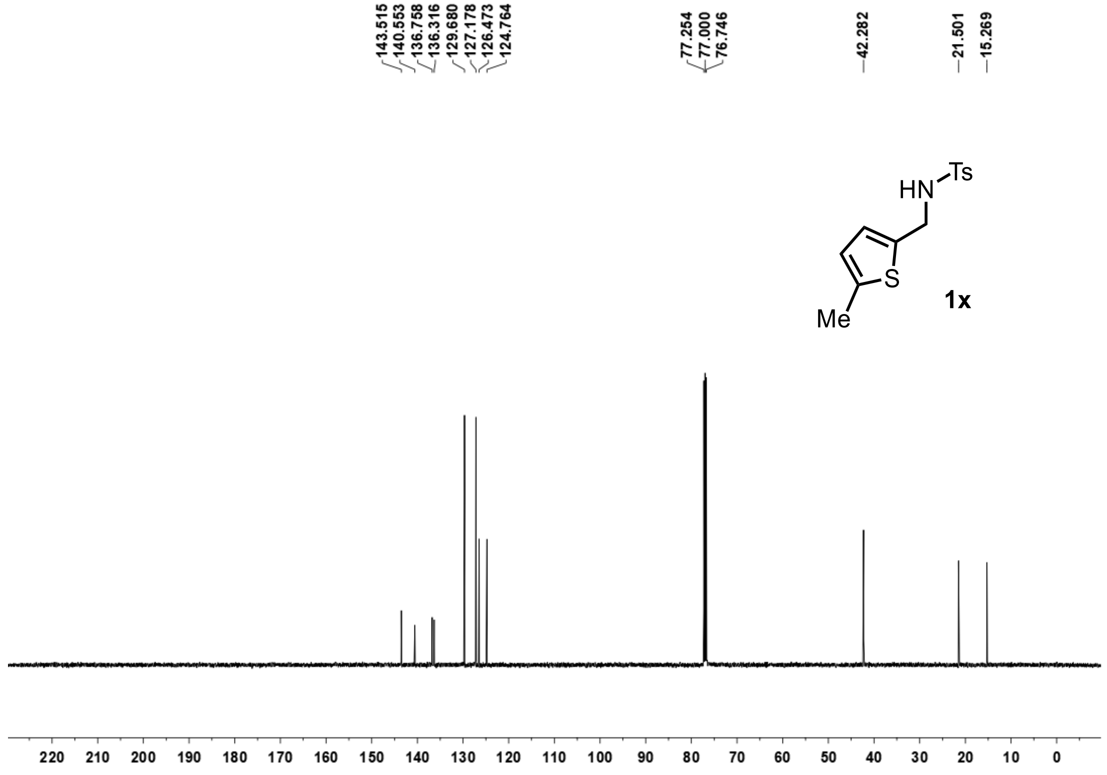

Supplementary Fig. 93 ^{13}C NMR (125 MHz, $CDCl_3$) of **1x**

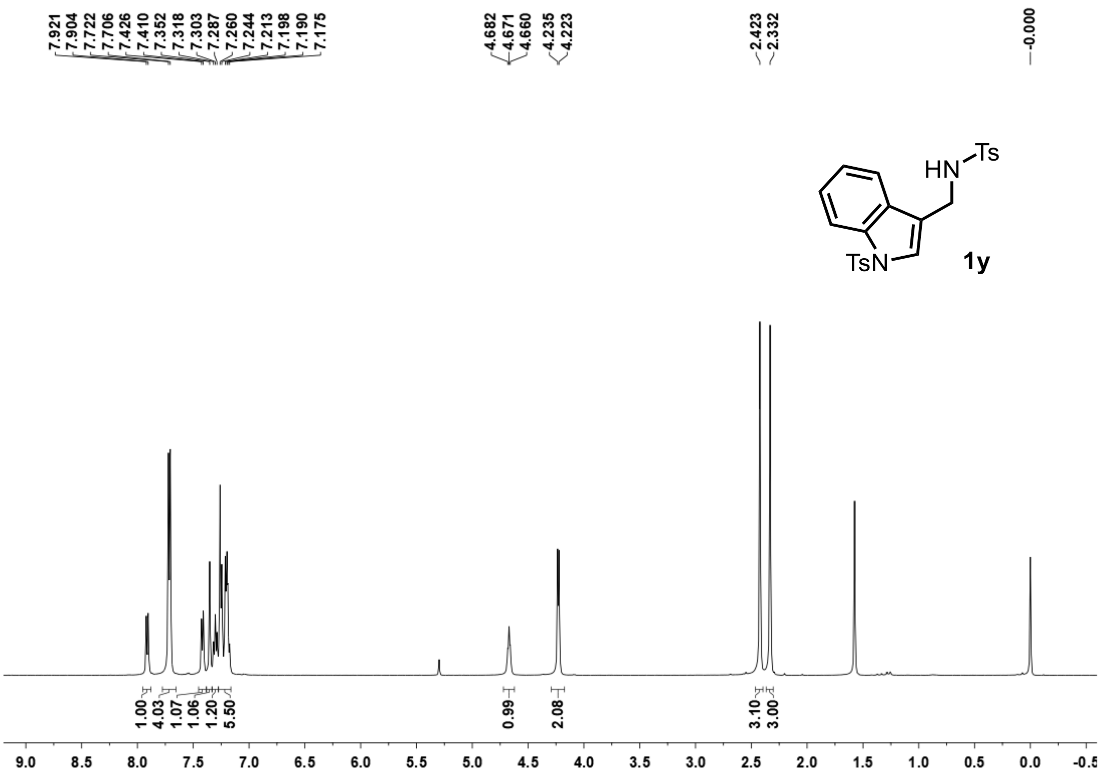

Supplementary Fig. 94 ^{1}H NMR (500 MHz, $CDCl_3$) of **1y**

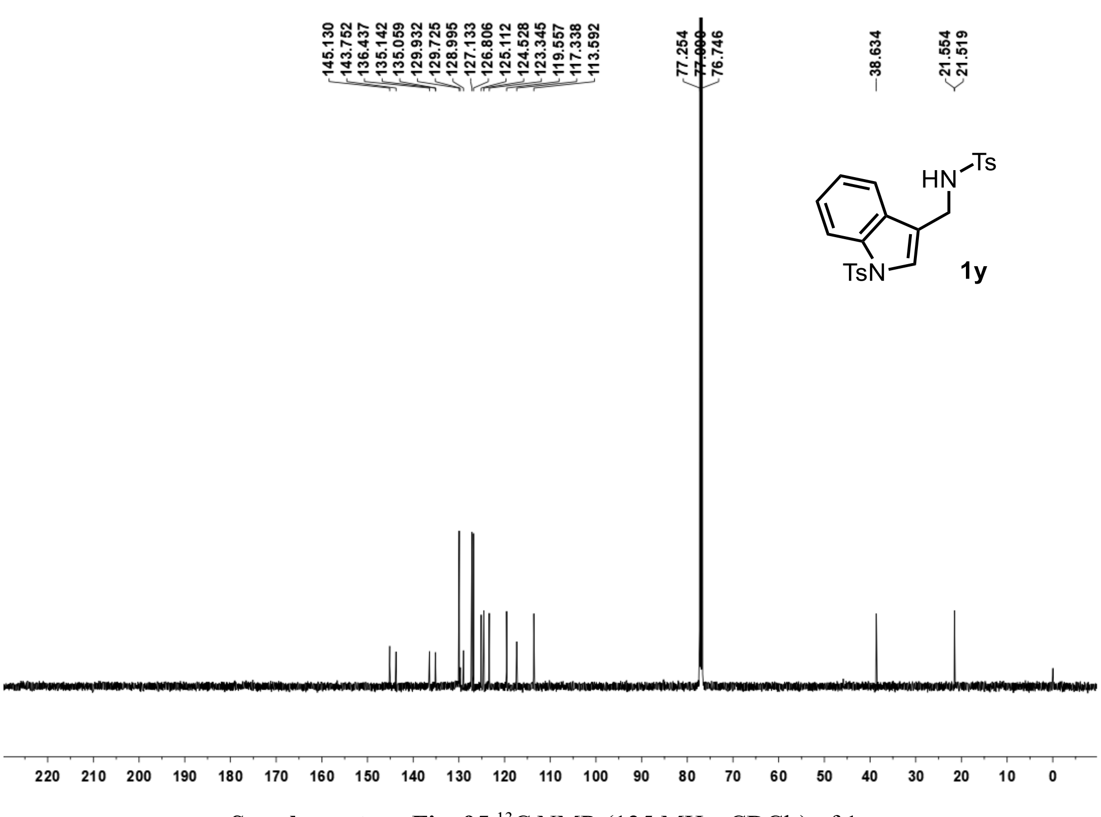

Supplementary Fig. 95 ^{13}C NMR (125 MHz, $CDCl_3$) of **1y**

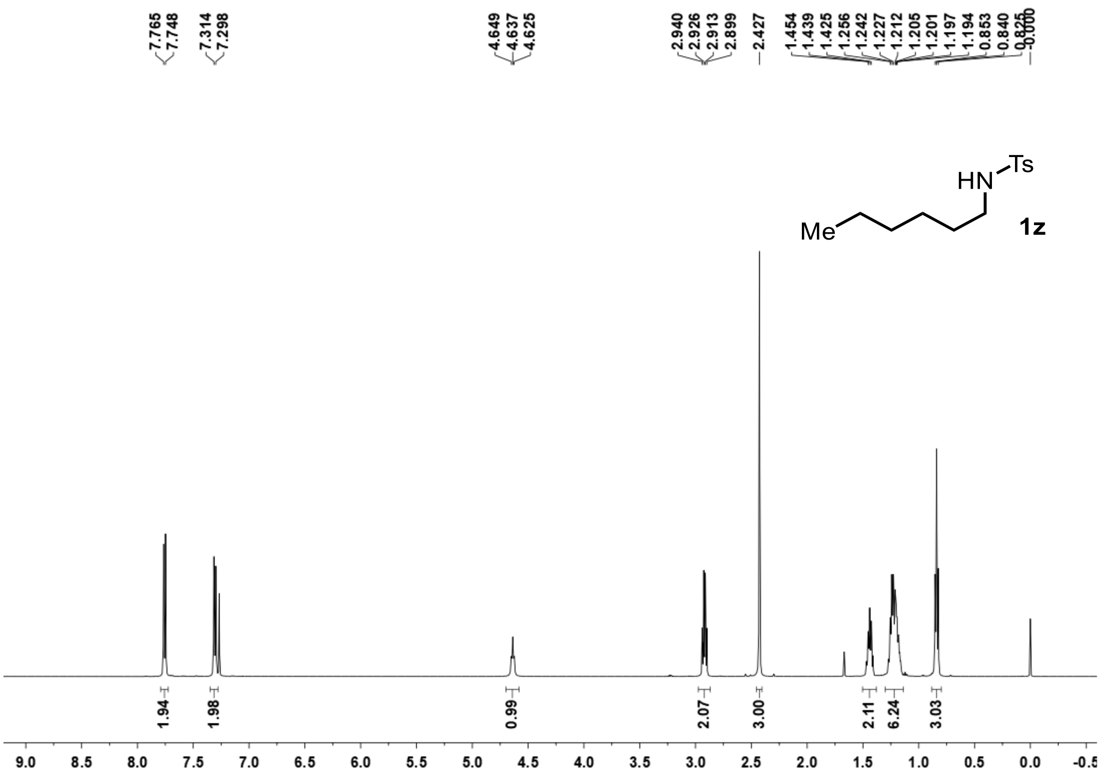

Supplementary Fig. 96 ^{1}H NMR (500 MHz, $CDCl_3$) of **1z**

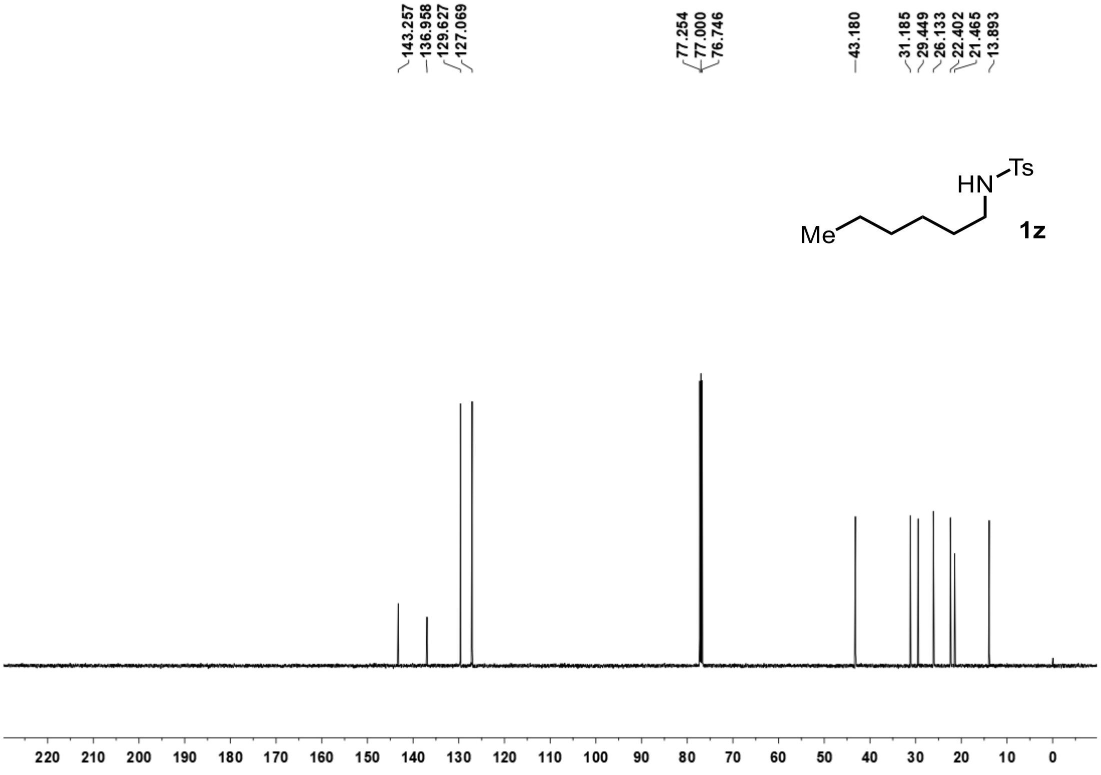

Supplementary Fig. 97 ^{13}C NMR (125 MHz, $CDCl_3$) of **1z**

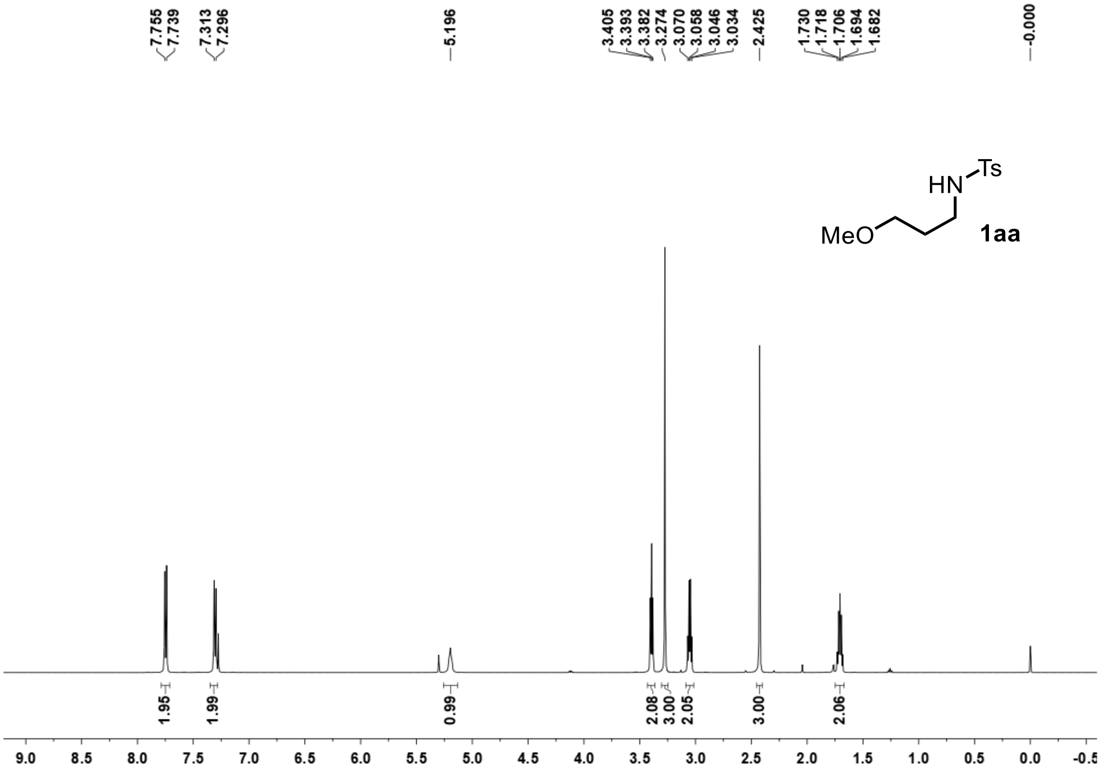

Supplementary Fig. 98 ^{1}H NMR (500 MHz, $CDCl_3$) of **1aa**

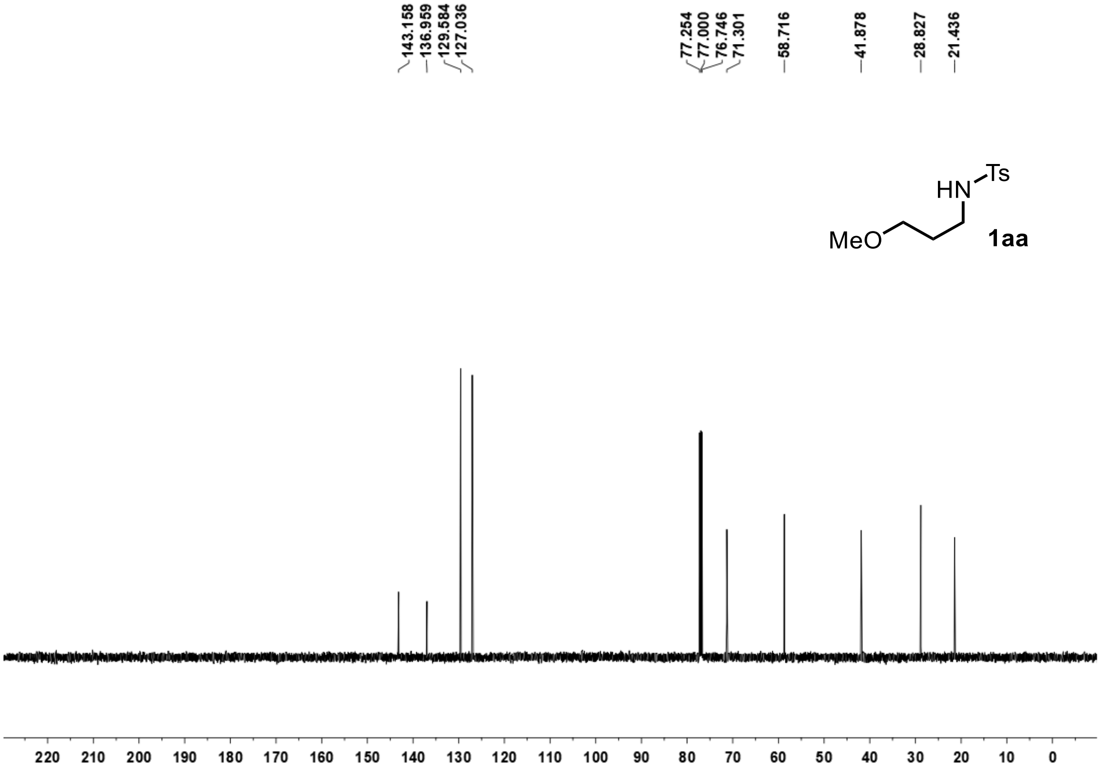

Supplementary Fig. 99 ^{13}C NMR (125 MHz, $CDCl_3$) of **1aa**

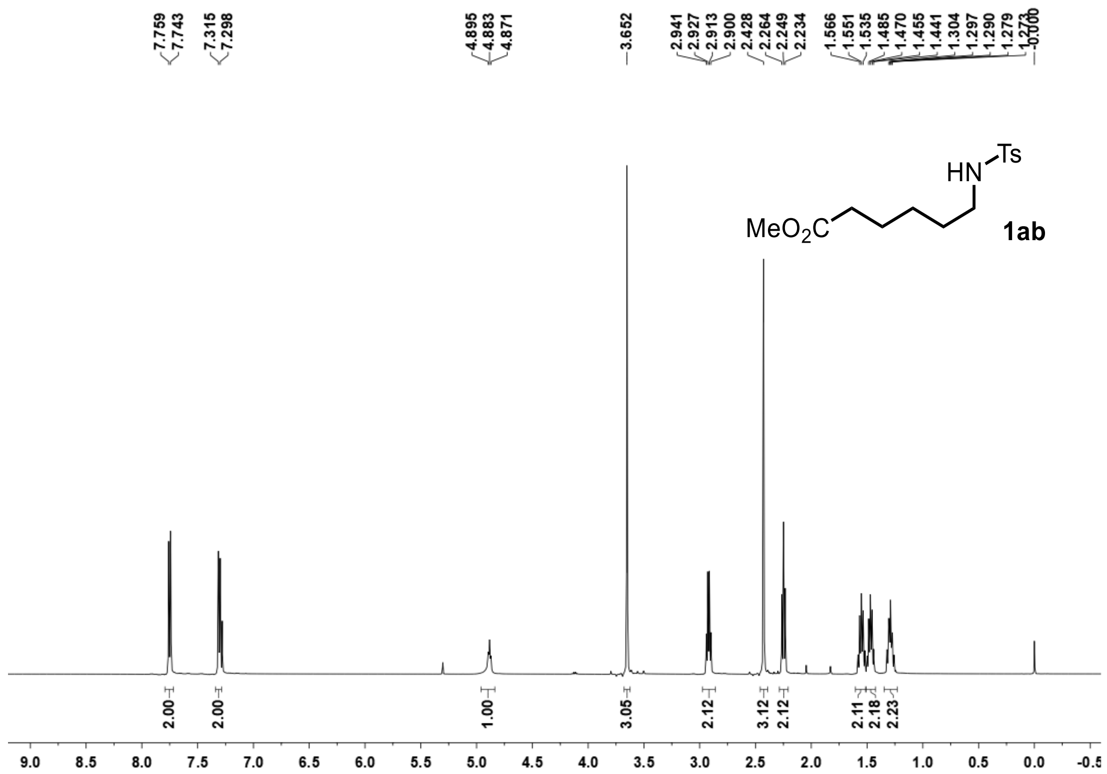

Supplementary Fig. 100 ^{1}H NMR (500 MHz, $CDCl_3$) of **1ab**

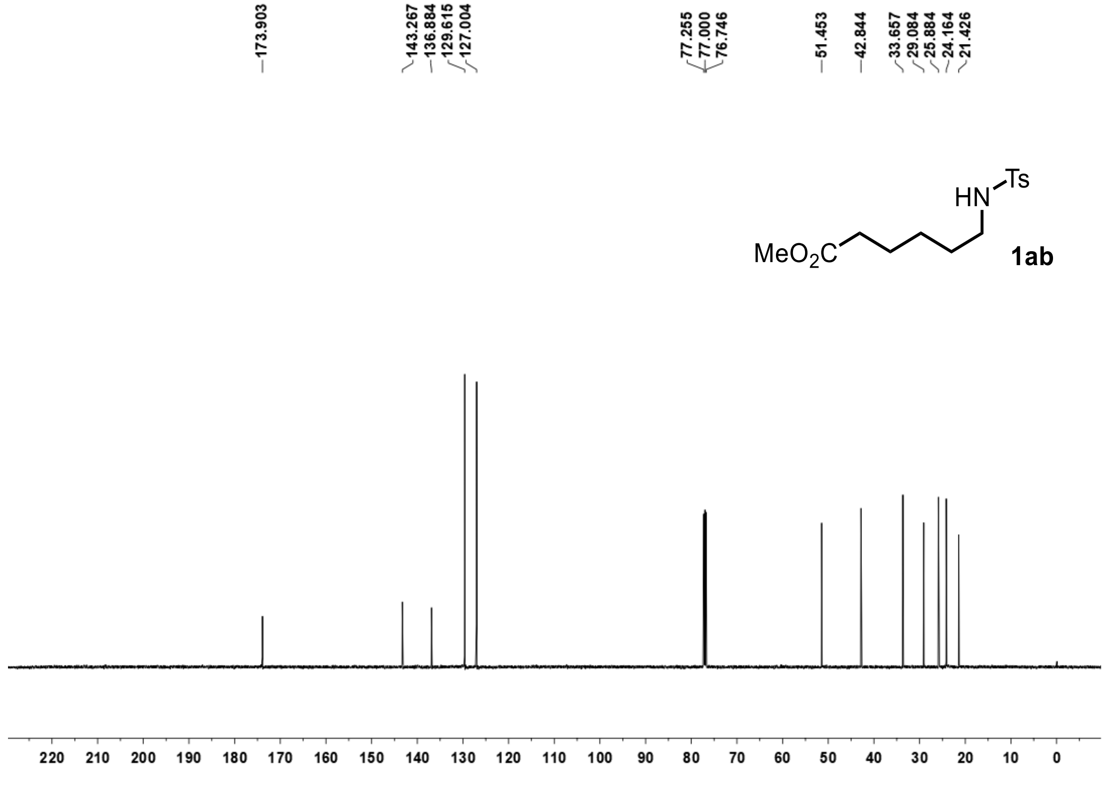

Supplementary Fig. 101 ^{13}C NMR (125 MHz, $CDCl_3$) of **1ab**

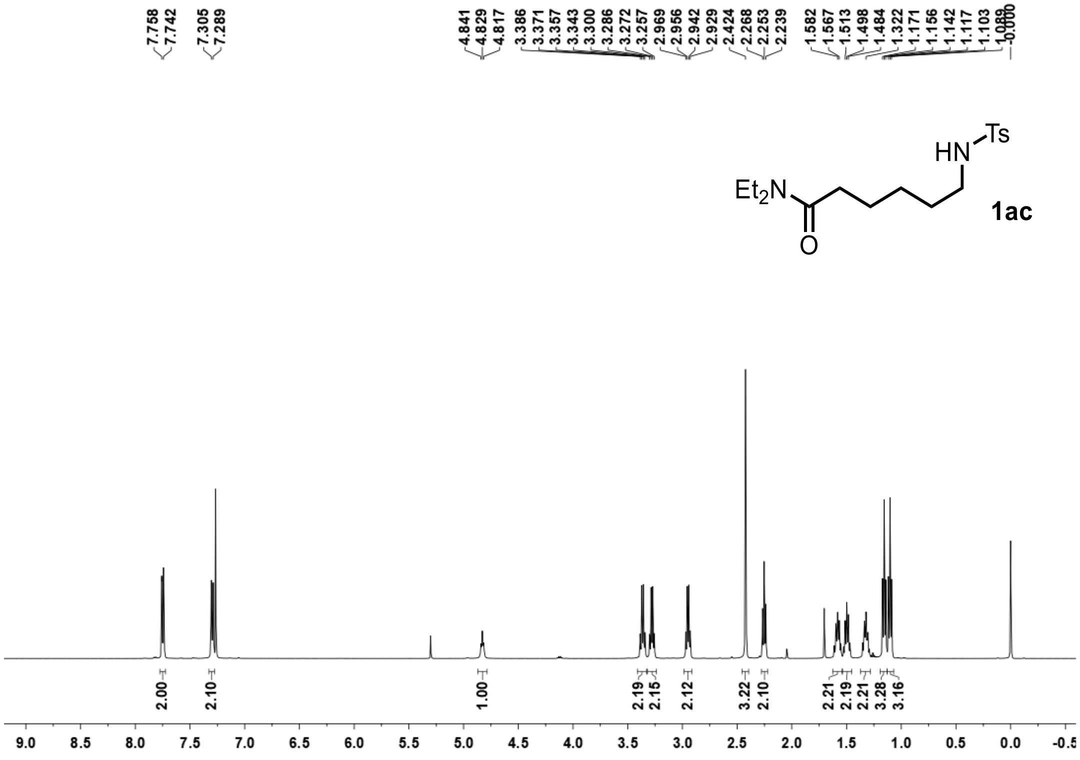

Supplementary Fig. 102 ^{1}H NMR (500 MHz, $CDCl_3$) of **1ac**

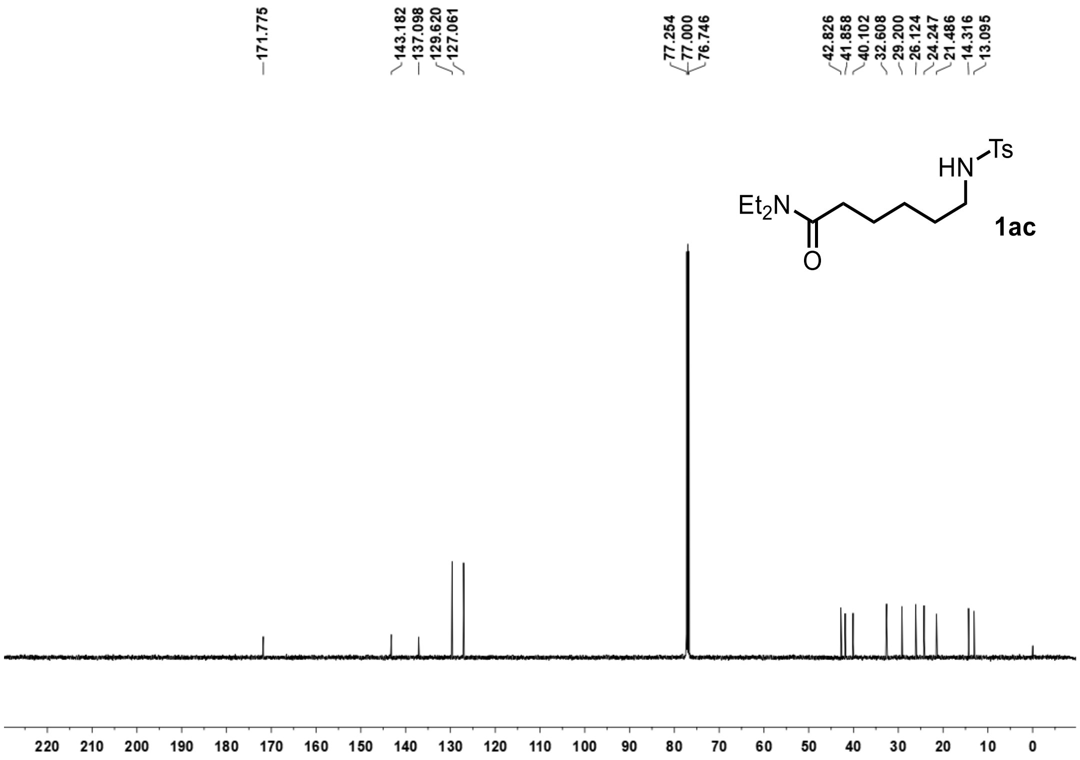

Supplementary Fig. 103 ^{13}C NMR (125 MHz, $CDCl_3$) of **1ac**

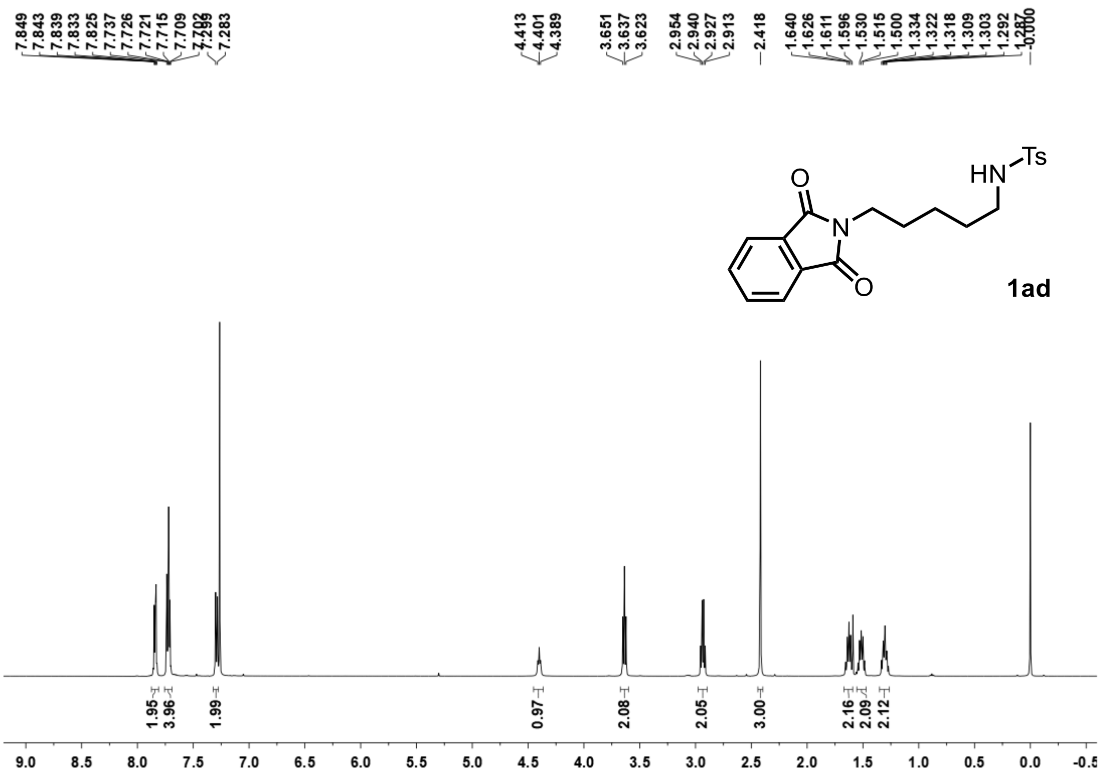

Supplementary Fig. 104 ^{1}H NMR (500 MHz, $CDCl_3$) of **1ad**

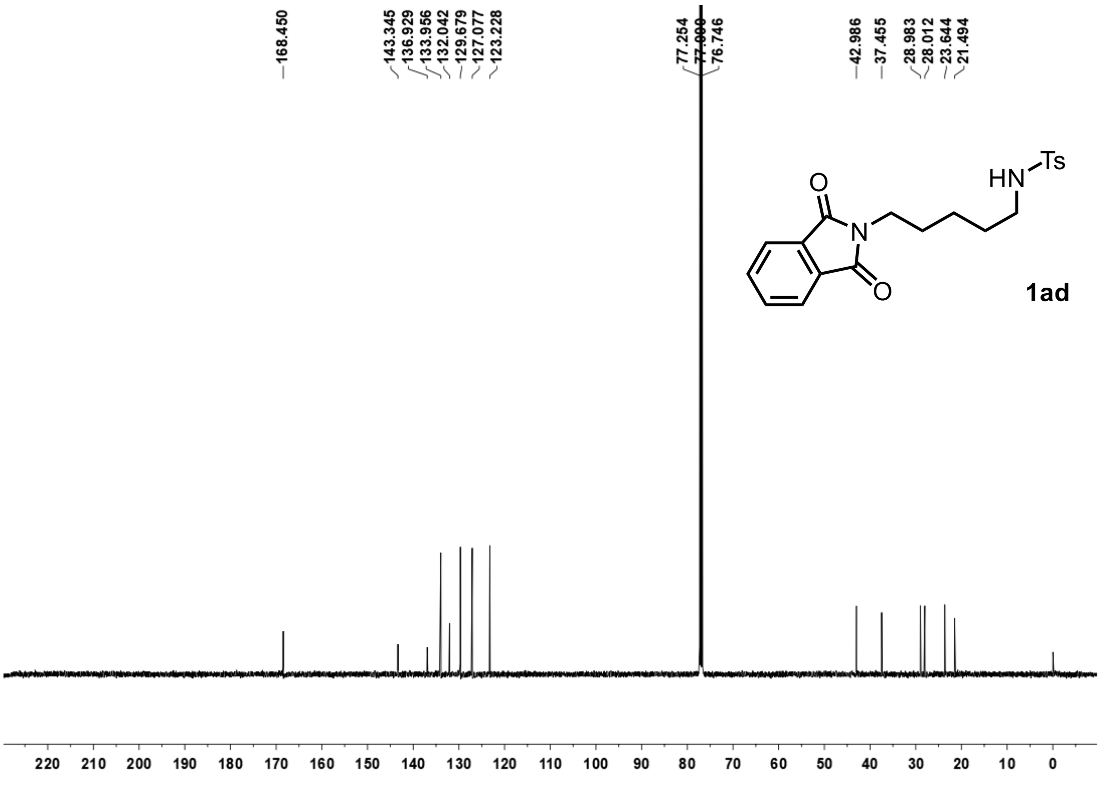

Supplementary Fig. 105 ^{13}C NMR (125 MHz, $CDCl_3$) of **1ad**

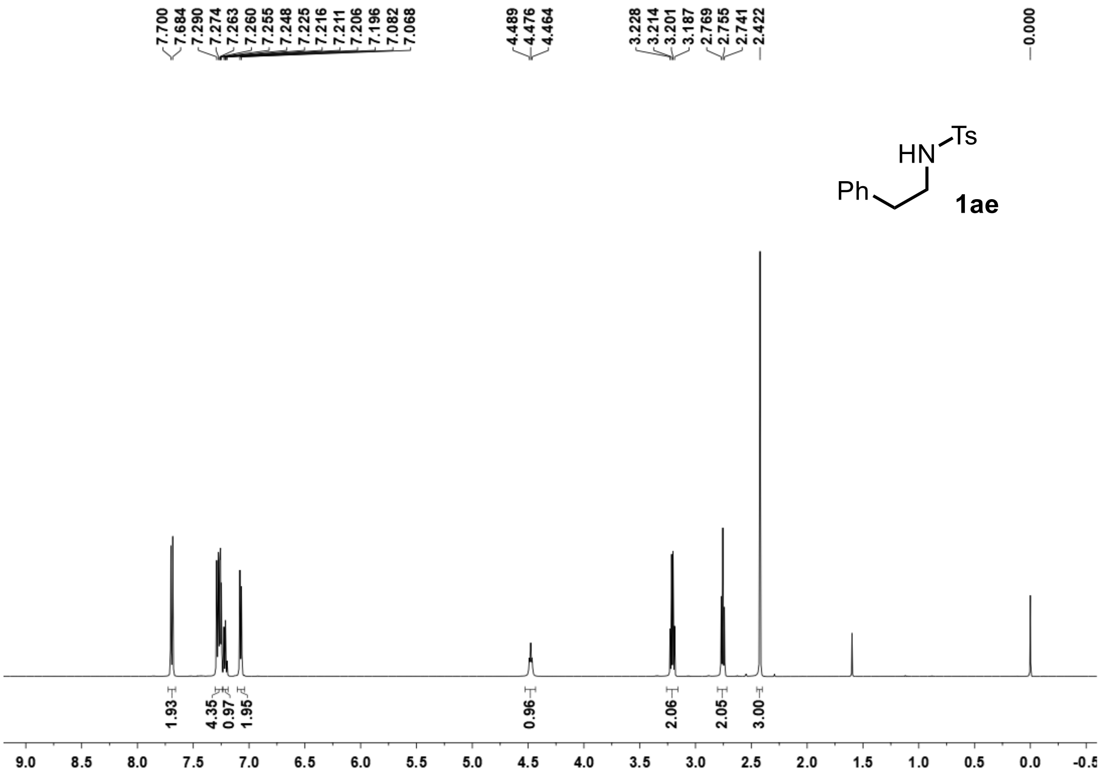

Supplementary Fig. 106 ^{1}H NMR (500 MHz, $CDCl_3$) of **1ae**

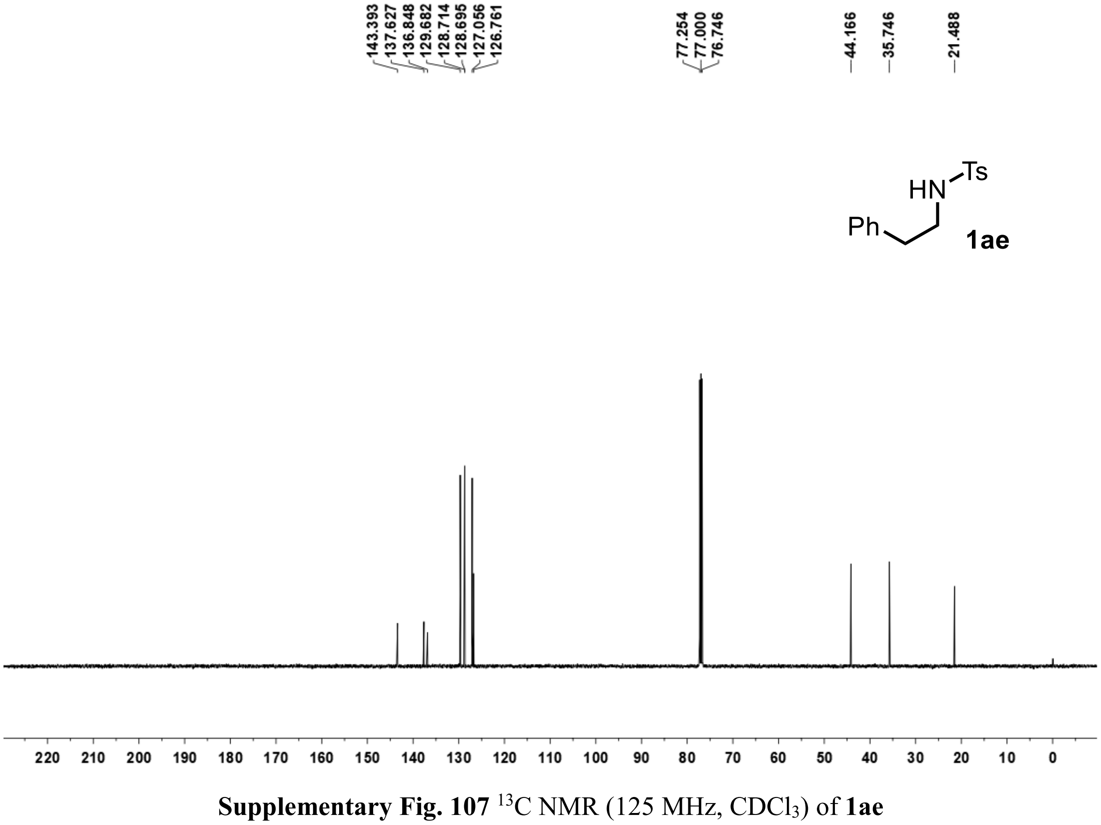

Supplementary Fig. 107 ^{13}C NMR (125 MHz, $CDCl_3$) of **1ae**

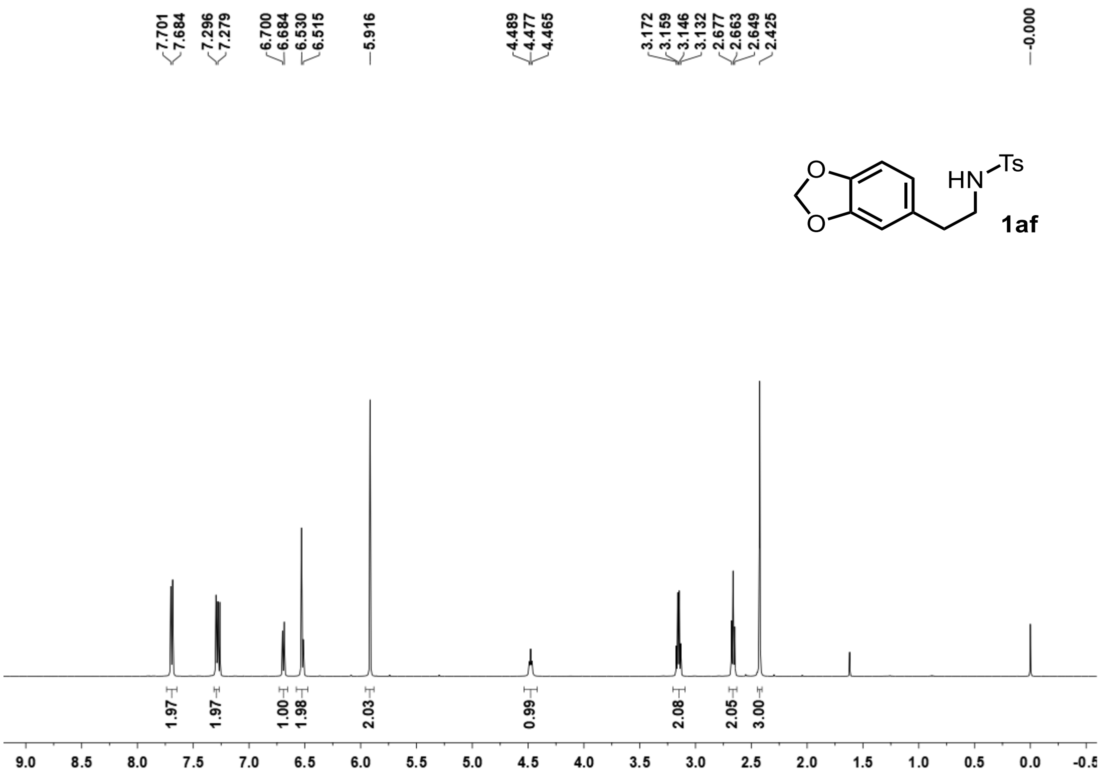

Supplementary Fig. 108 ^{1}H NMR (500 MHz, $CDCl_3$) of **1af**

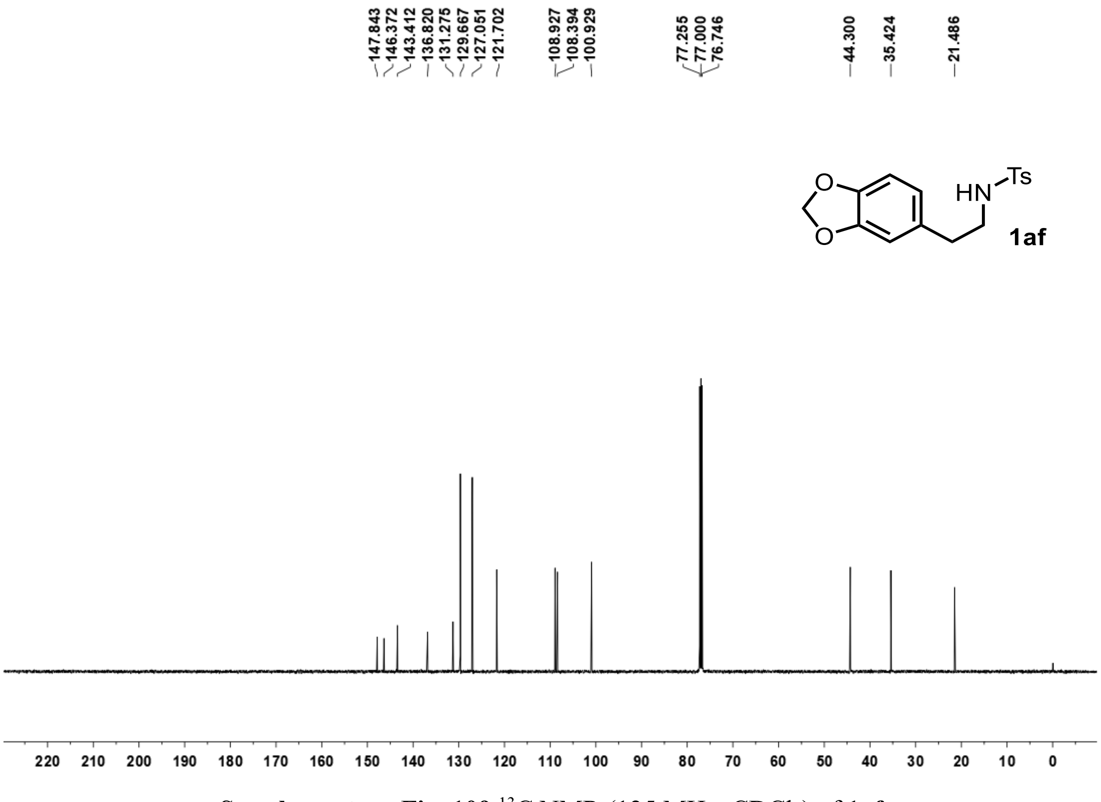

Supplementary Fig. 109 ^{13}C NMR (125 MHz, $CDCl_3$) of **1af**

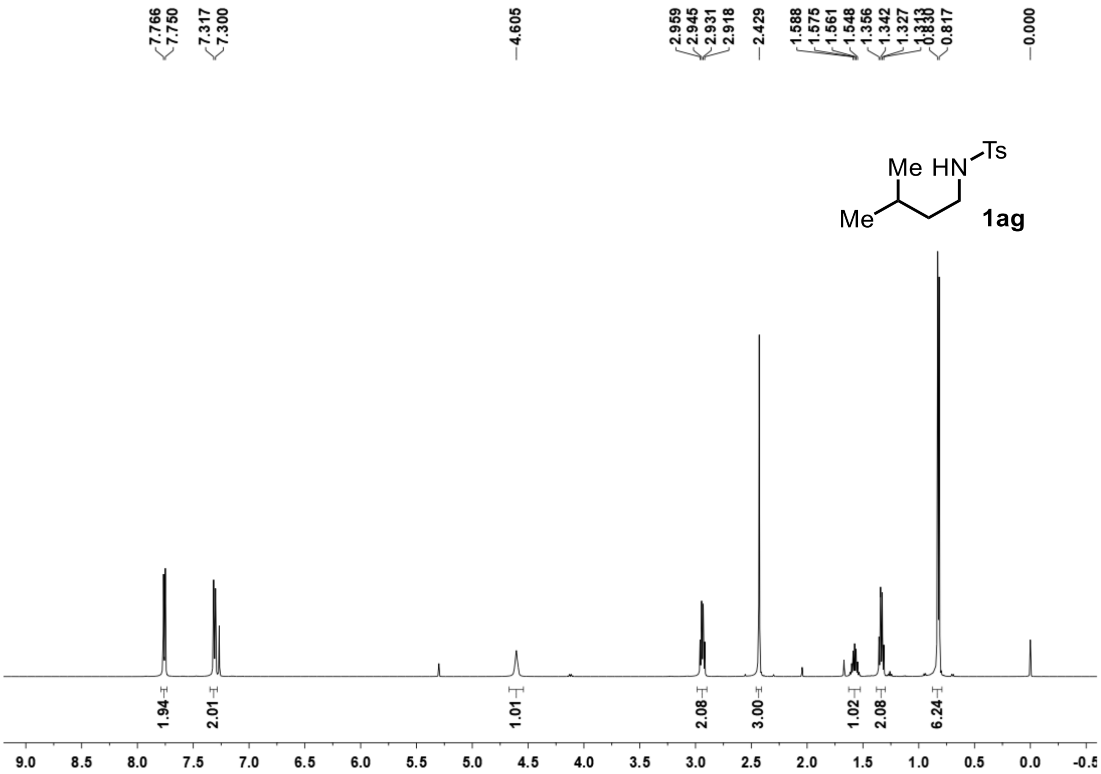

Supplementary Fig. 110 ^{1}H NMR (500 MHz, $CDCl_3$) of **1ag**

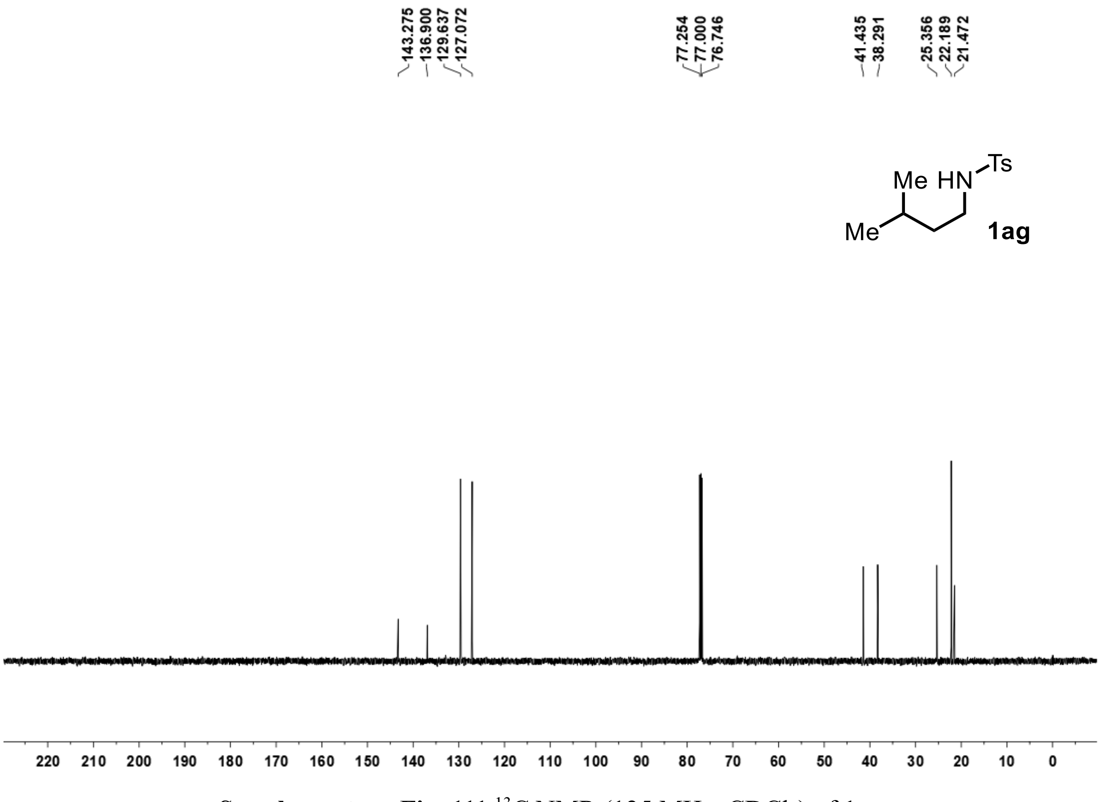

Supplementary Fig. 111 ^{13}C NMR (125 MHz, $CDCl_3$) of **1ag**

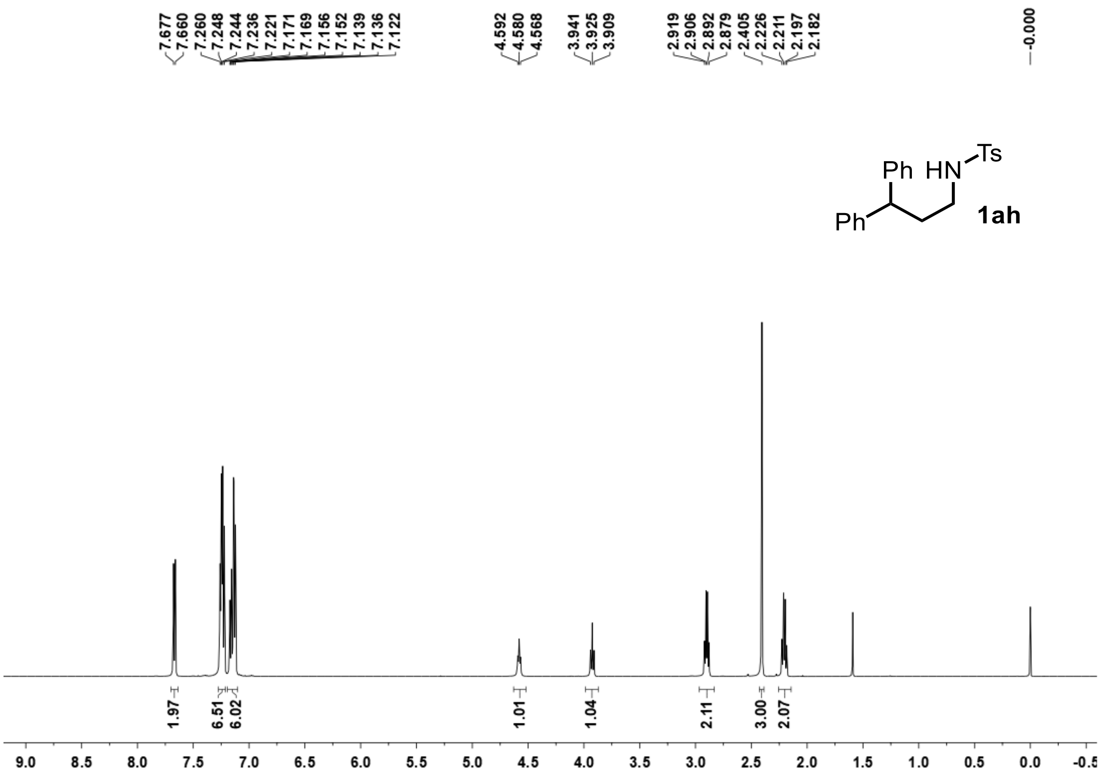

Supplementary Fig. 112 ^{1}H NMR (500 MHz, $CDCl_3$) of **1ah**

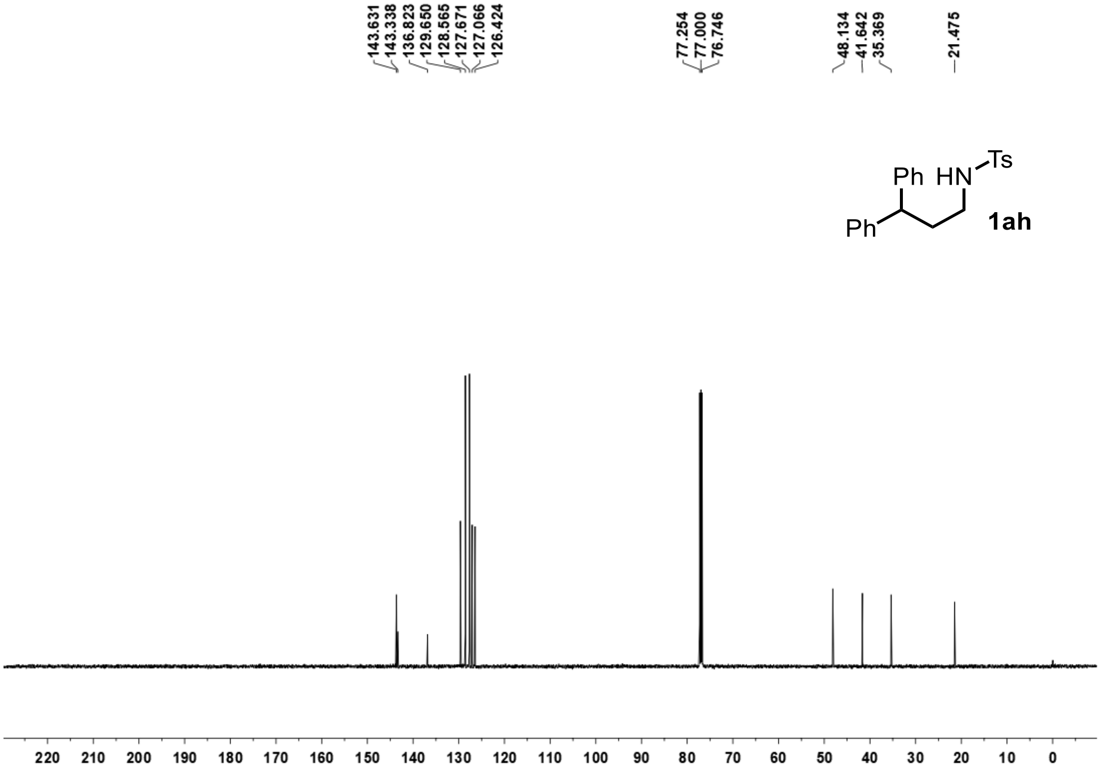

Supplementary Fig. 113 ^{13}C NMR (125 MHz, $CDCl_3$) of **1ah**

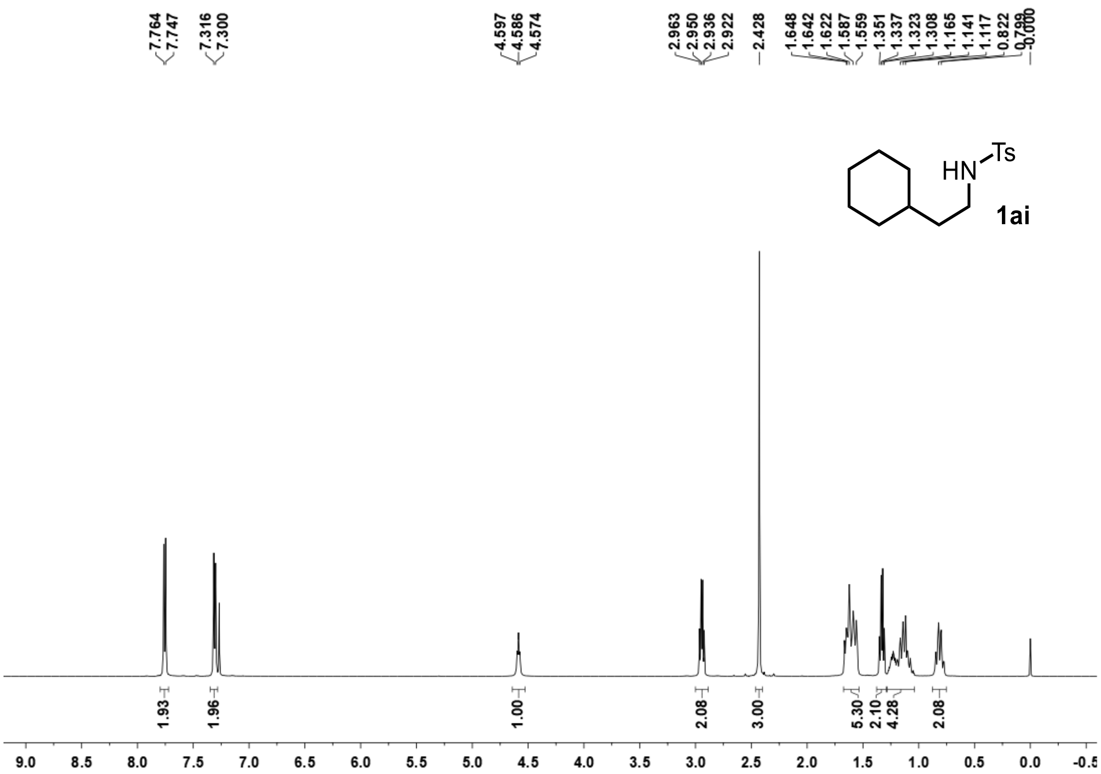

Supplementary Fig. 114 ^{1}H NMR (500 MHz, $CDCl_3$) of **1ai**

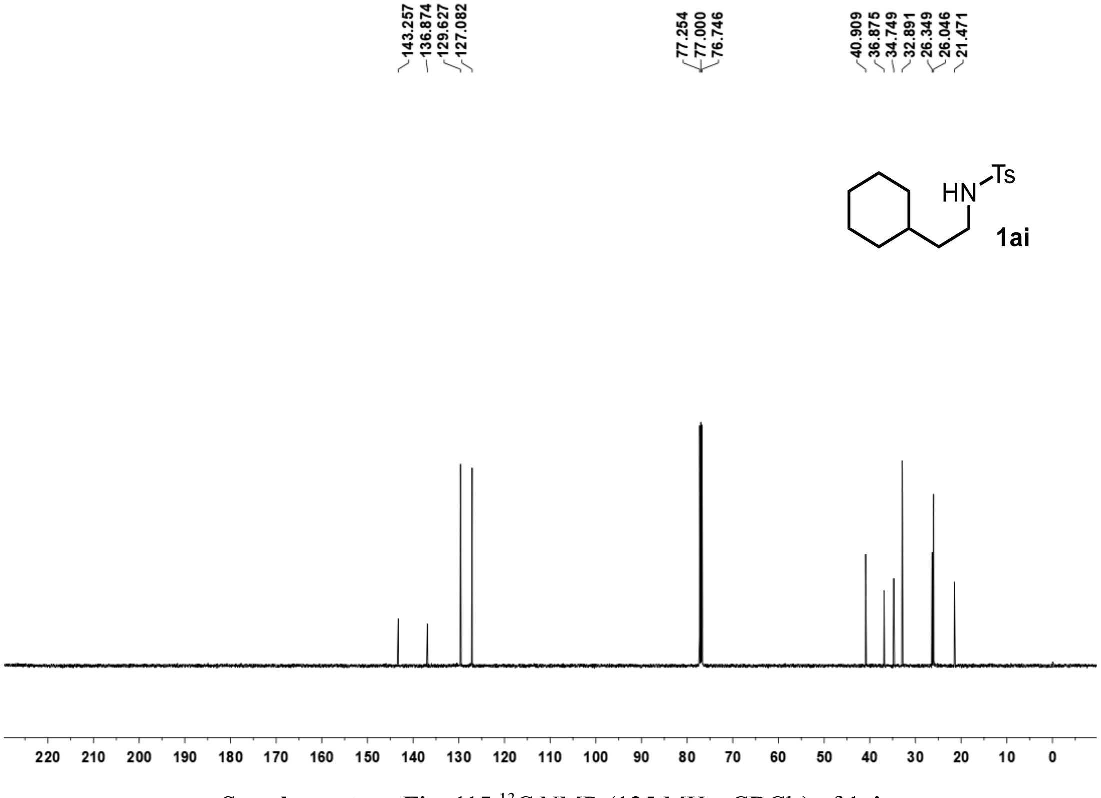

Supplementary Fig. 115 ^{13}C NMR (125 MHz, $CDCl_3$) of **1ai**

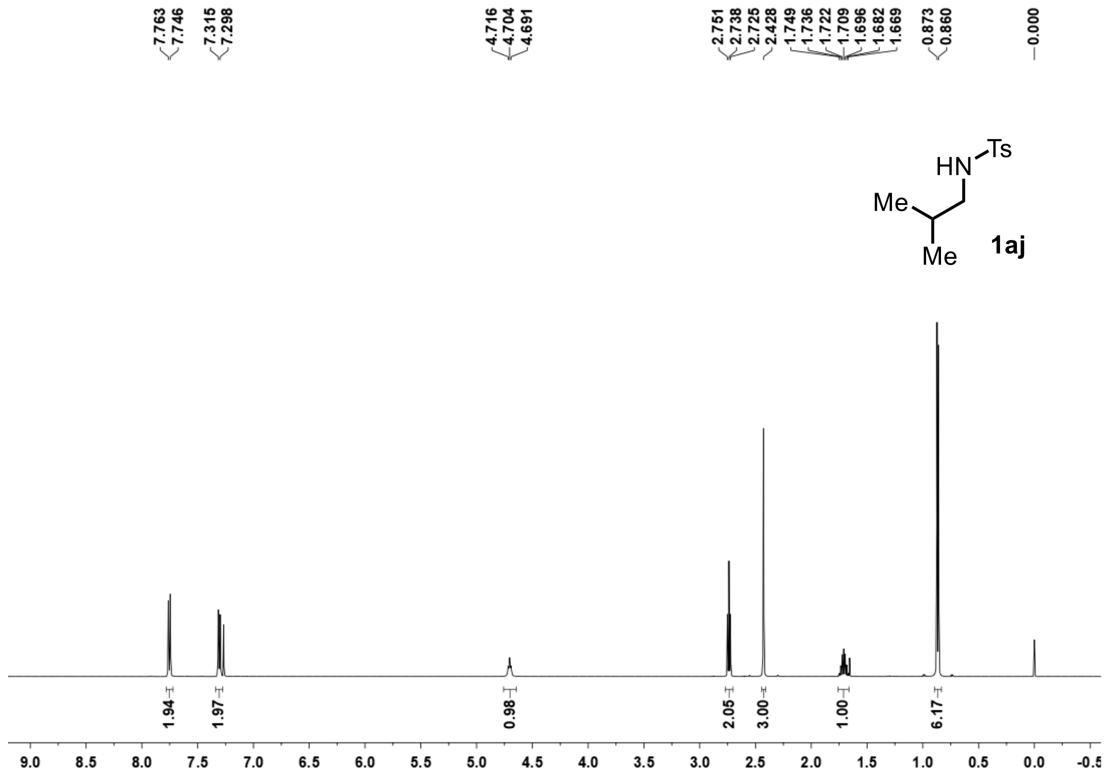

Supplementary Fig. 116 ^{1}H NMR (500 MHz, $CDCl_3$) of **1aj**

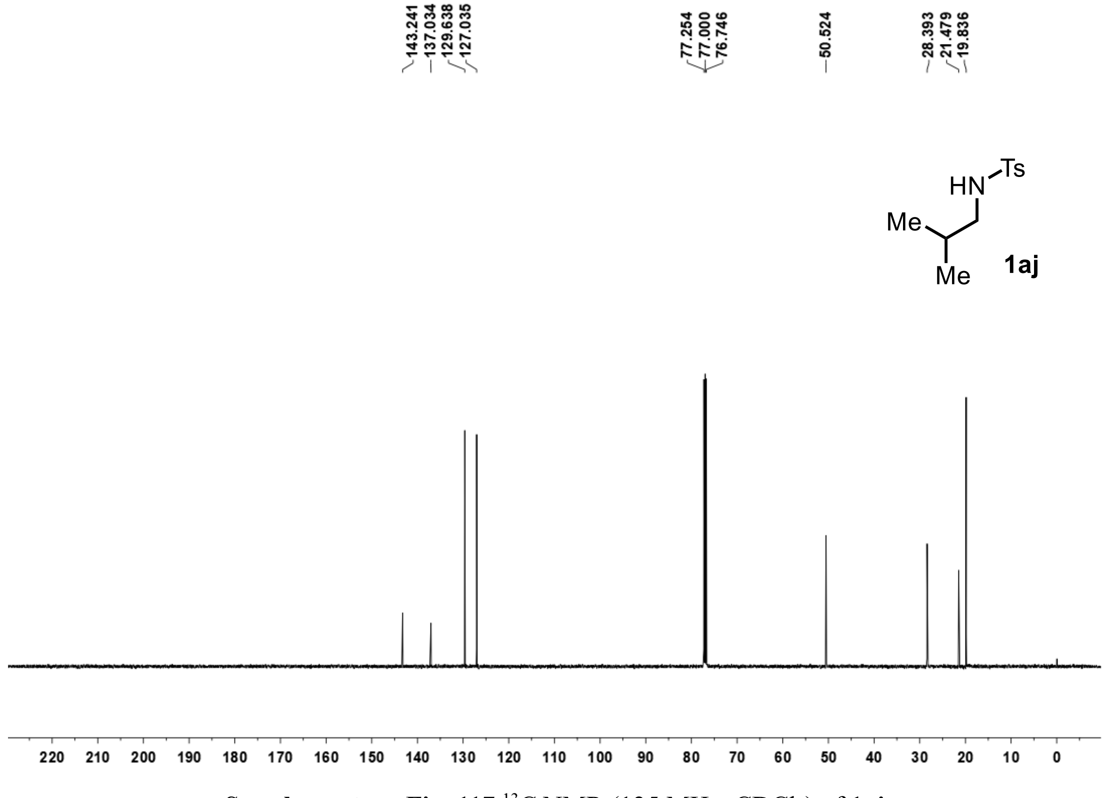

Supplementary Fig. 117 ^{13}C NMR (125 MHz, $CDCl_3$) of **1aj**

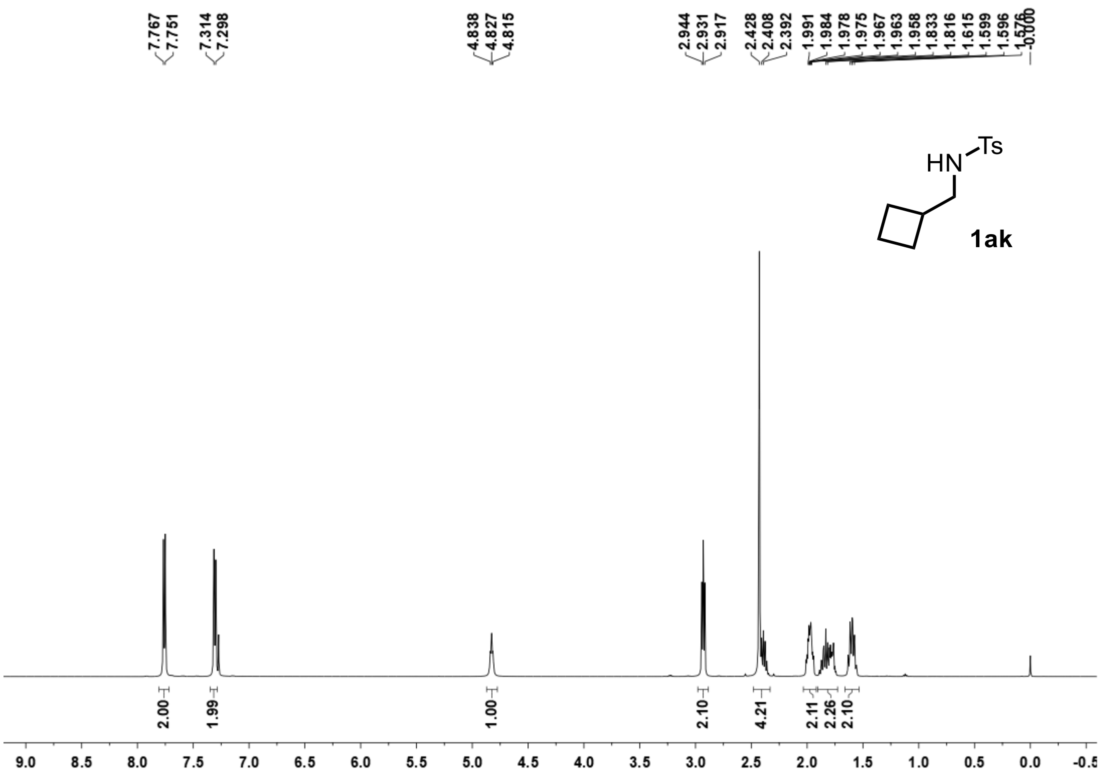

Supplementary Fig. 118 ^{1}H NMR (500 MHz, $CDCl_3$) of **1ak**

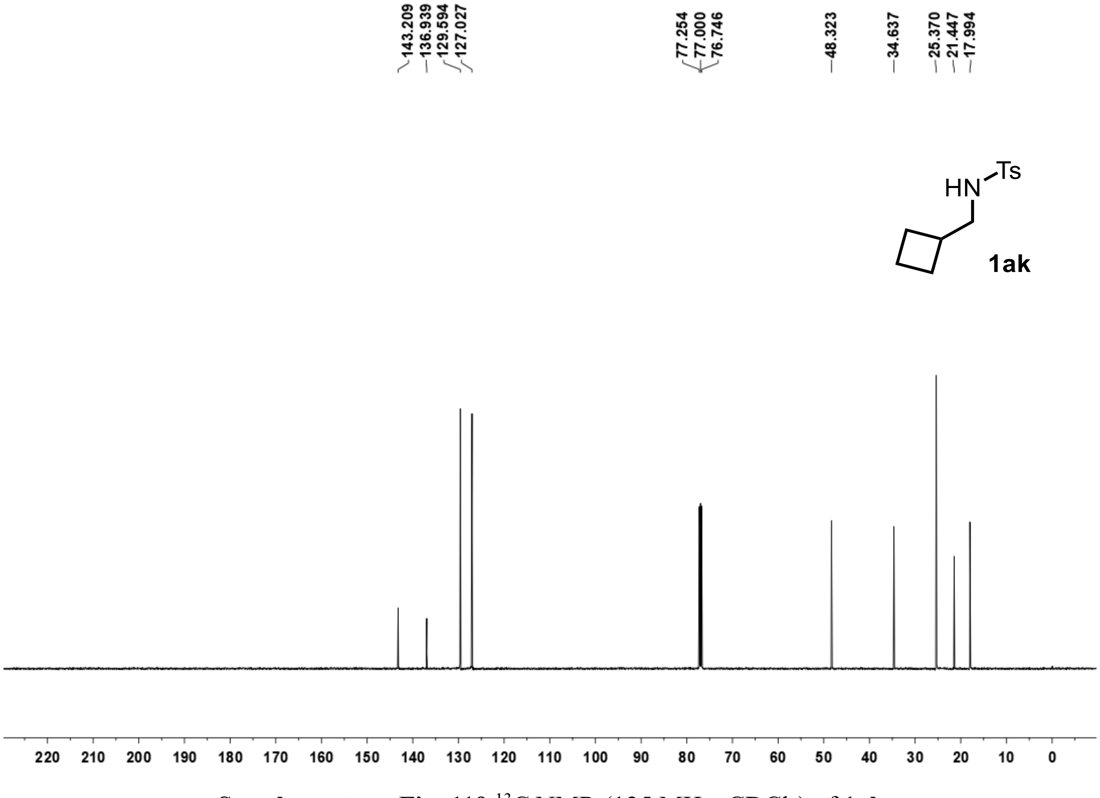

Supplementary Fig. 119 ^{13}C NMR (125 MHz, $CDCl_3$) of **1ak**

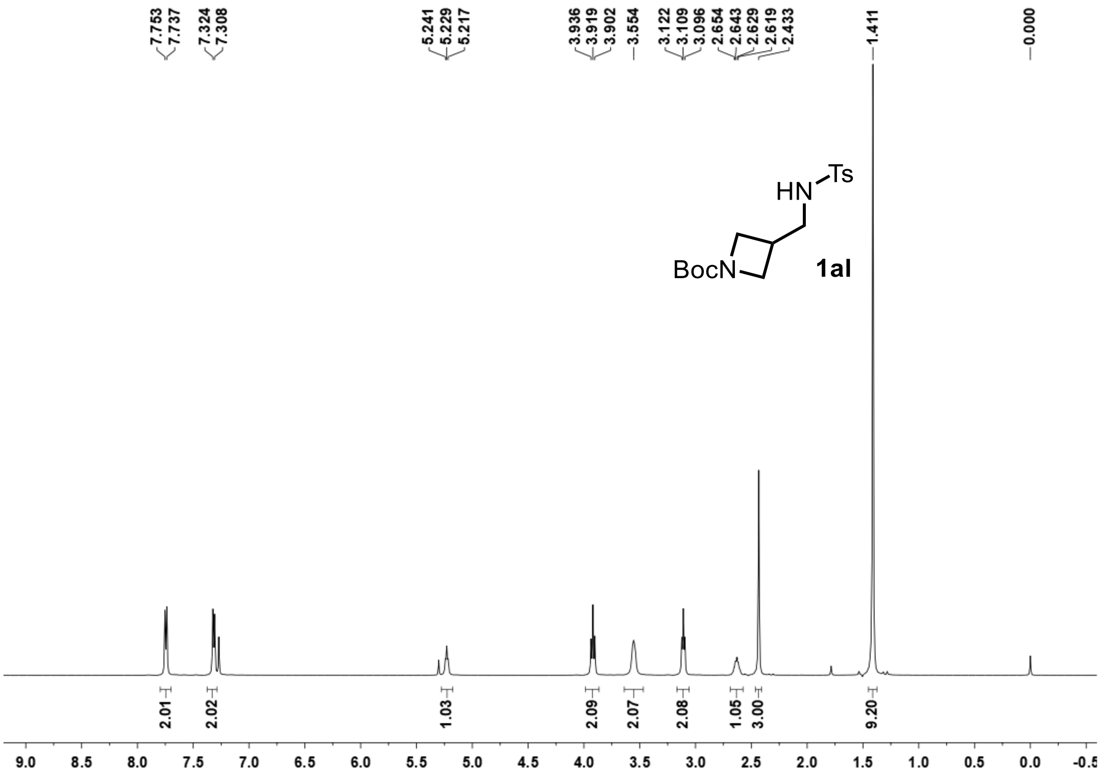

Supplementary Fig. 120 ^{1}H NMR (500 MHz, $CDCl_3$) of **1al**

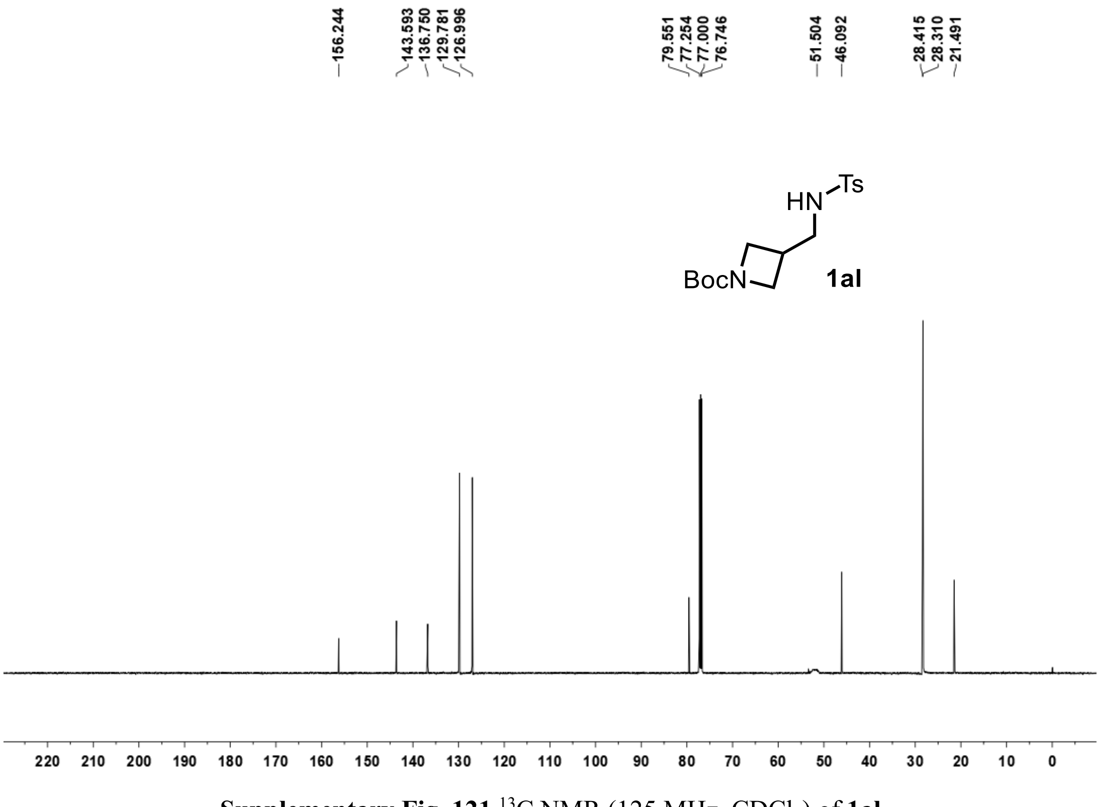

Supplementary Fig. 121 ^{13}C NMR (125 MHz, $CDCl_3$) of **1al**

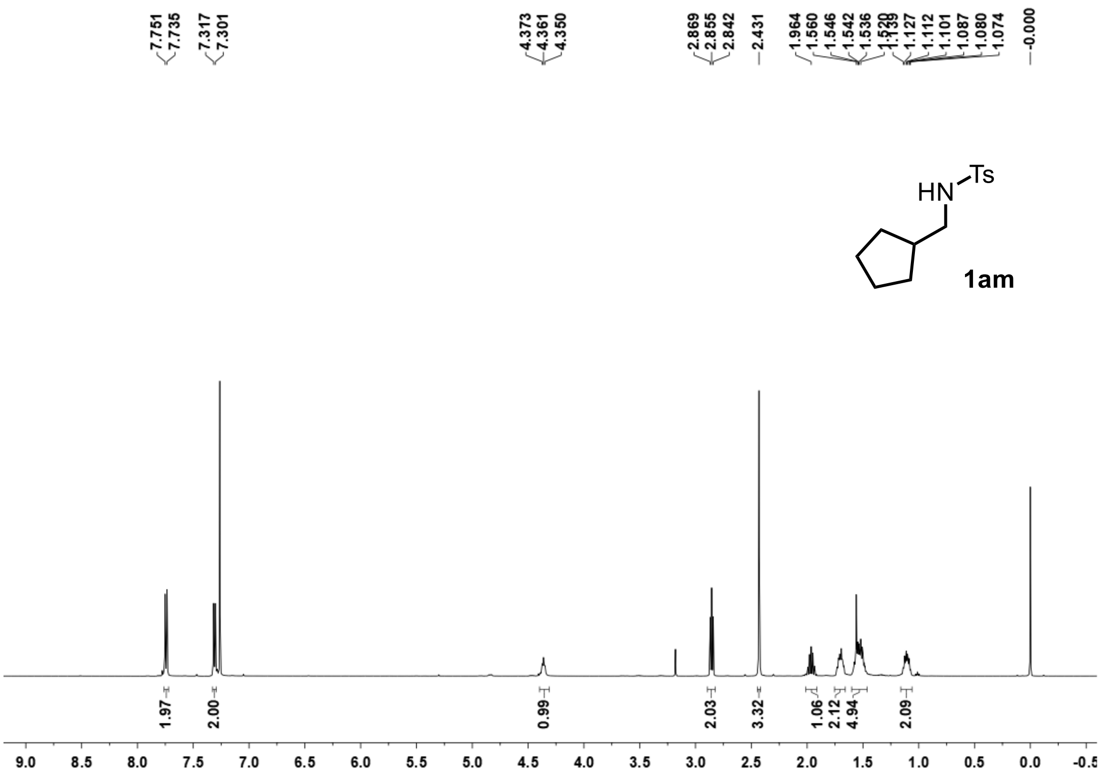

Supplementary Fig. 122 ^{1}H NMR (500 MHz, $CDCl_3$) of **1am**

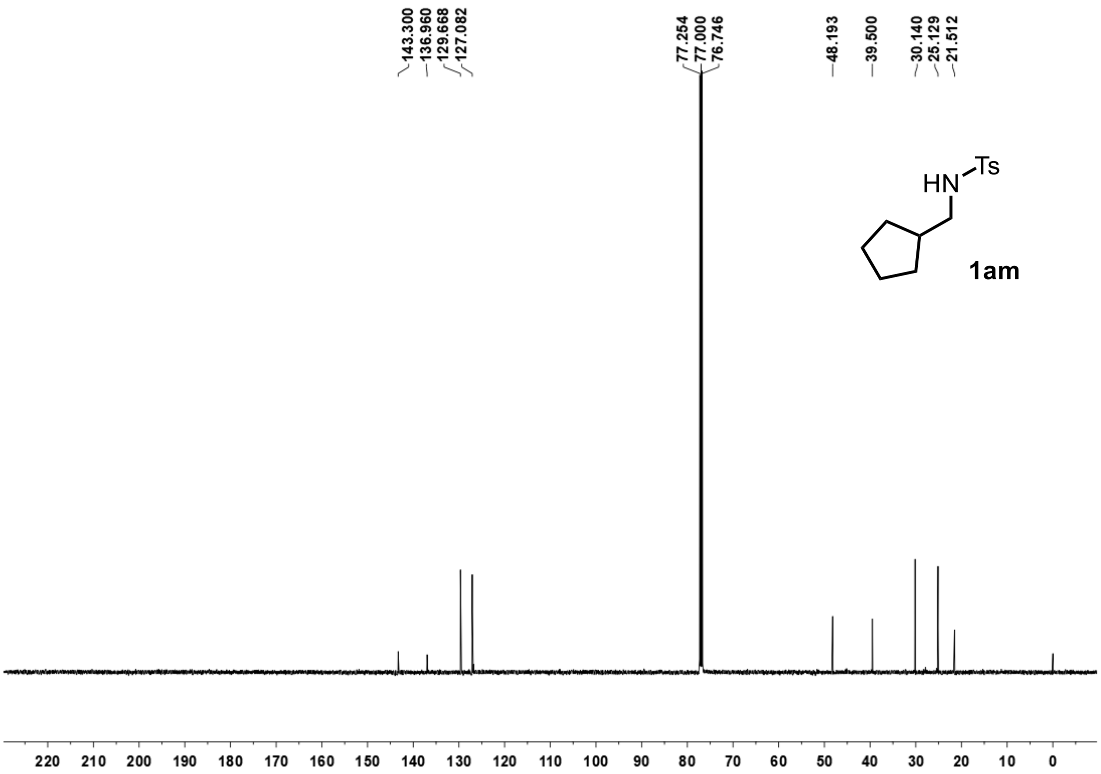

Supplementary Fig. 123 ^{13}C NMR (125 MHz, $CDCl_3$) of **1am**

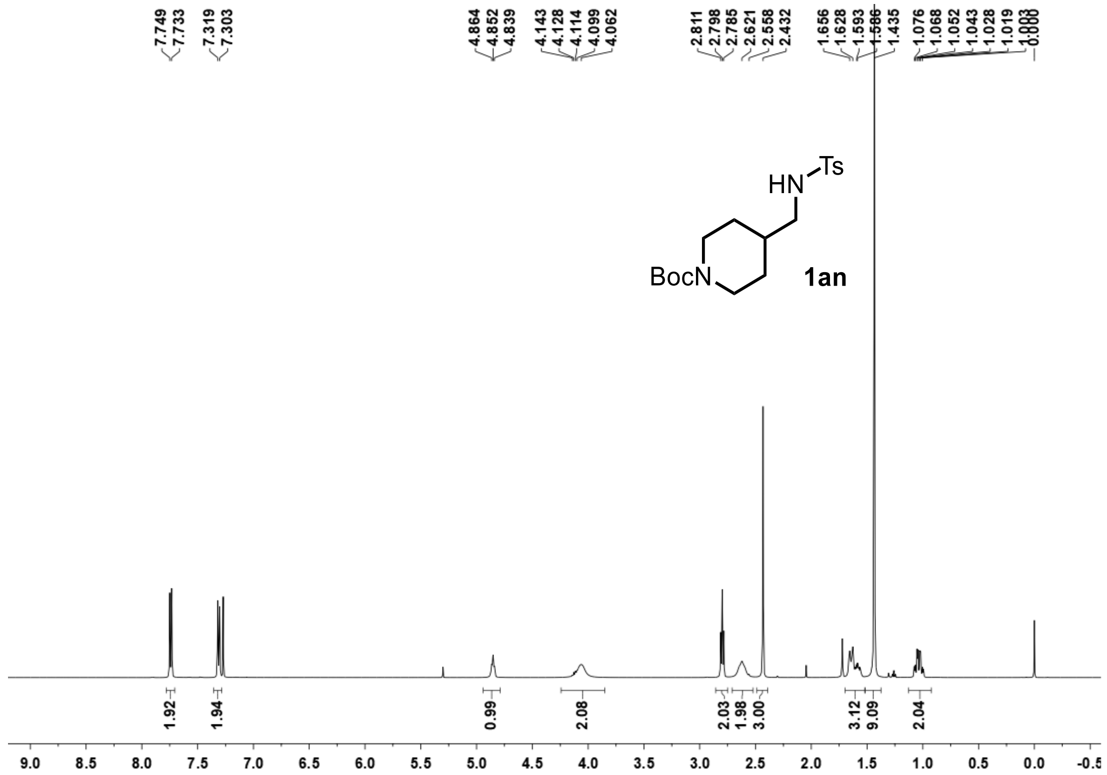

Supplementary Fig. 124 ^{1}H NMR (500 MHz, $CDCl_3$) of **1an**

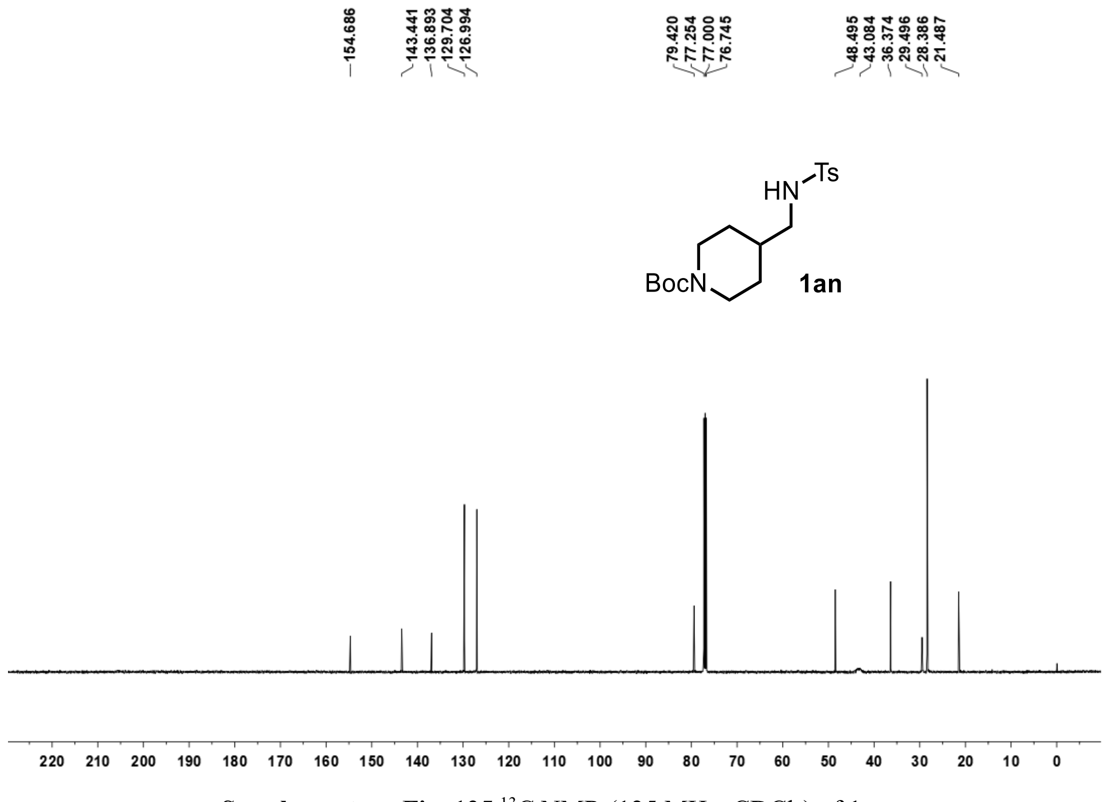

Supplementary Fig. 125 ^{13}C NMR (125 MHz, $CDCl_3$) of **1an**

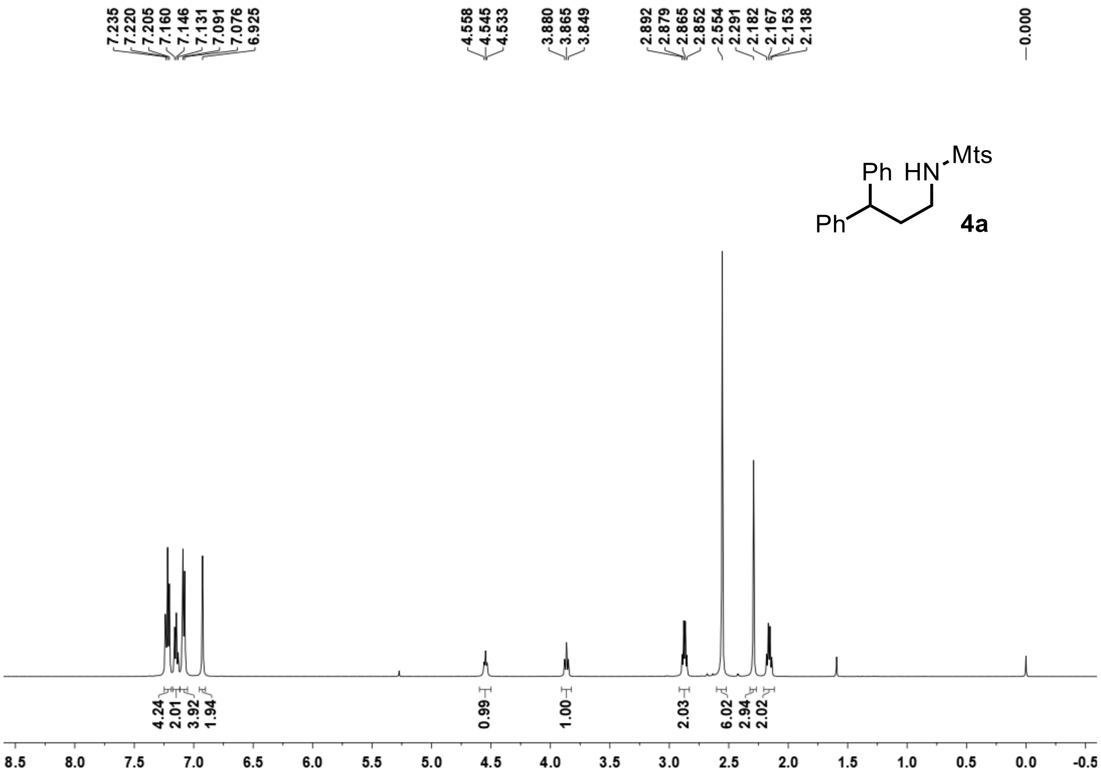

Supplementary Fig. 126 ^{1}H NMR (500 MHz, $CDCl_3$) of **4a**

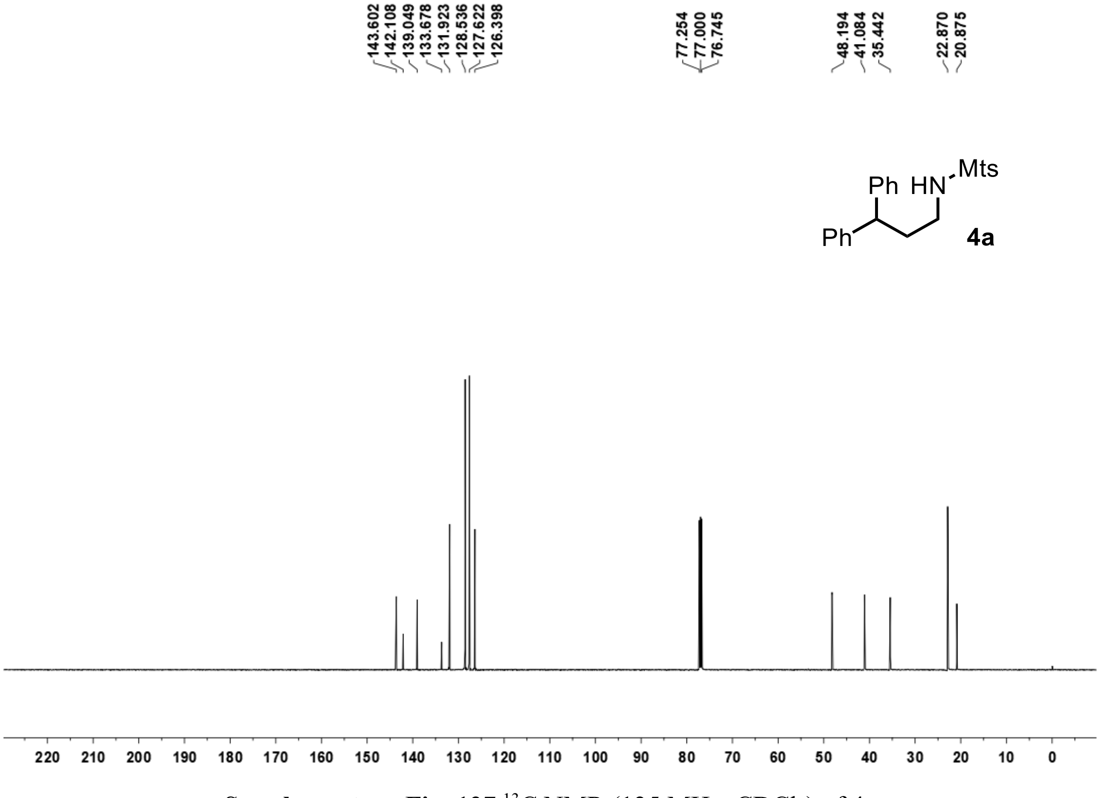

Supplementary Fig. 127 ^{13}C NMR (125 MHz, $CDCl_3$) of **4a**

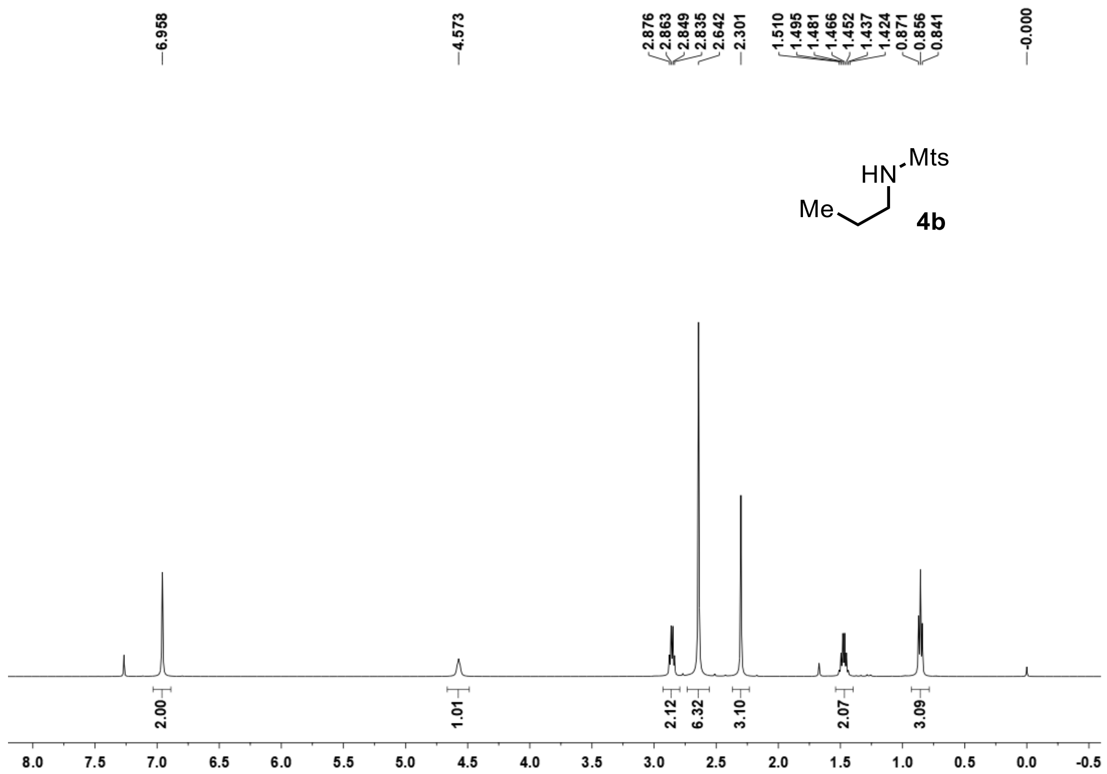

Supplementary Fig. 128 ^{1}H NMR (500 MHz, $CDCl_3$) of **4b**

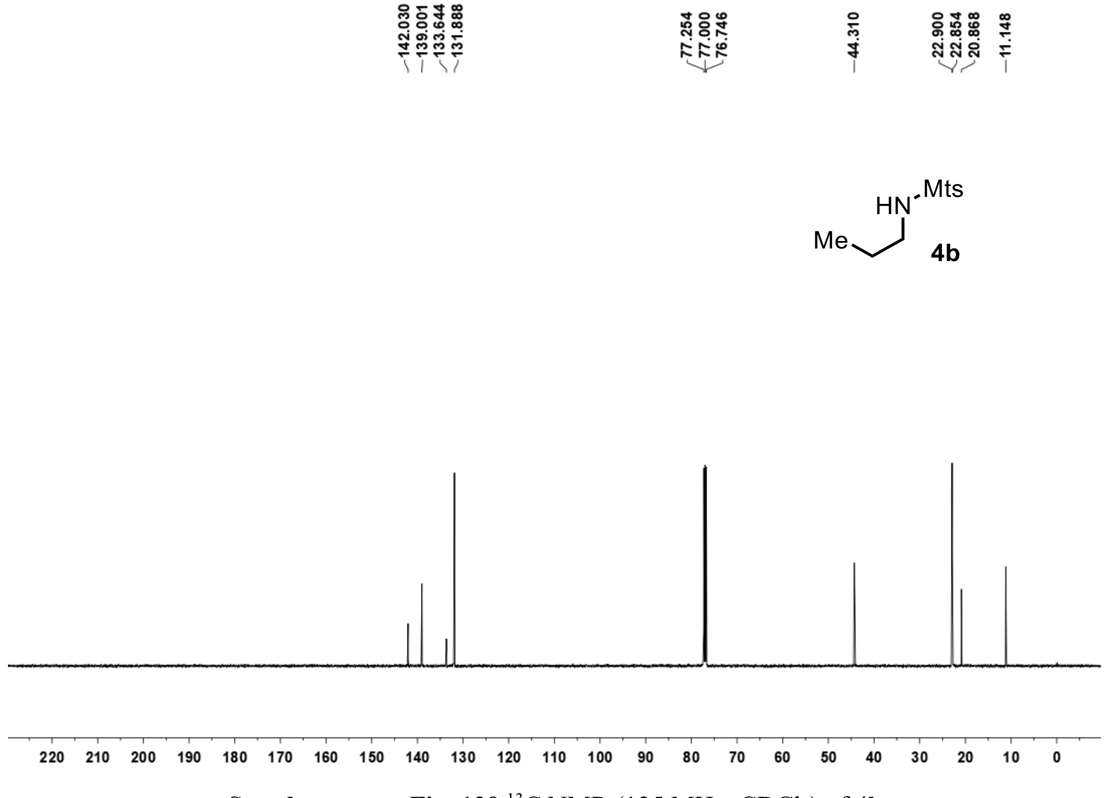

Supplementary Fig. 129 ^{13}C NMR (125 MHz, $CDCl_3$) of **4b**

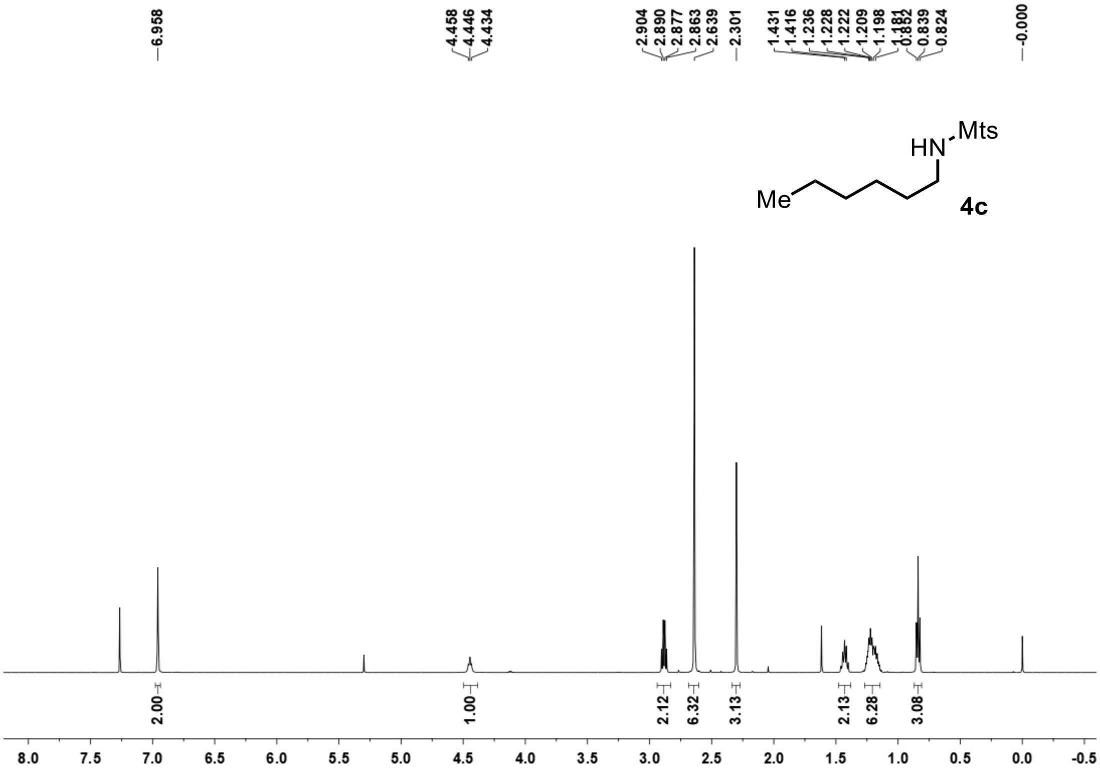

Supplementary Fig. 130 ^{1}H NMR (500 MHz, $CDCl_3$) of **4c**

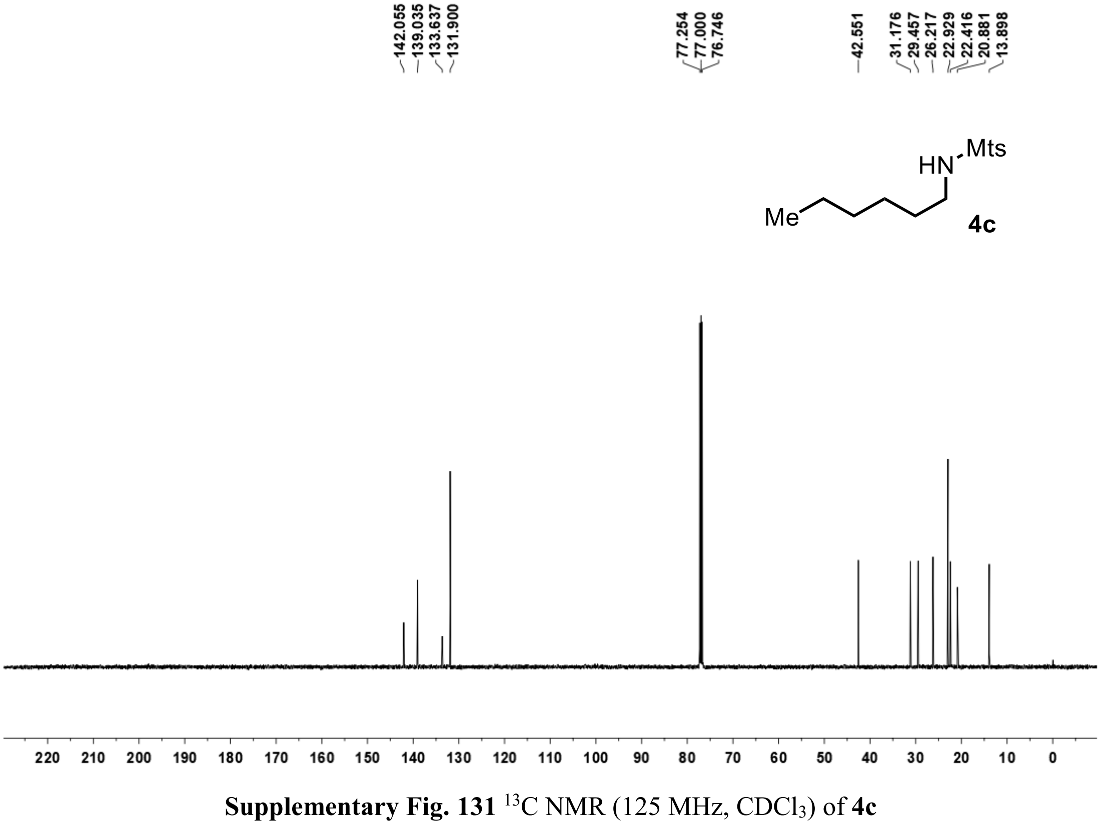

Supplementary Fig. 131 ^{13}C NMR (125 MHz, $CDCl_3$) of **4c**

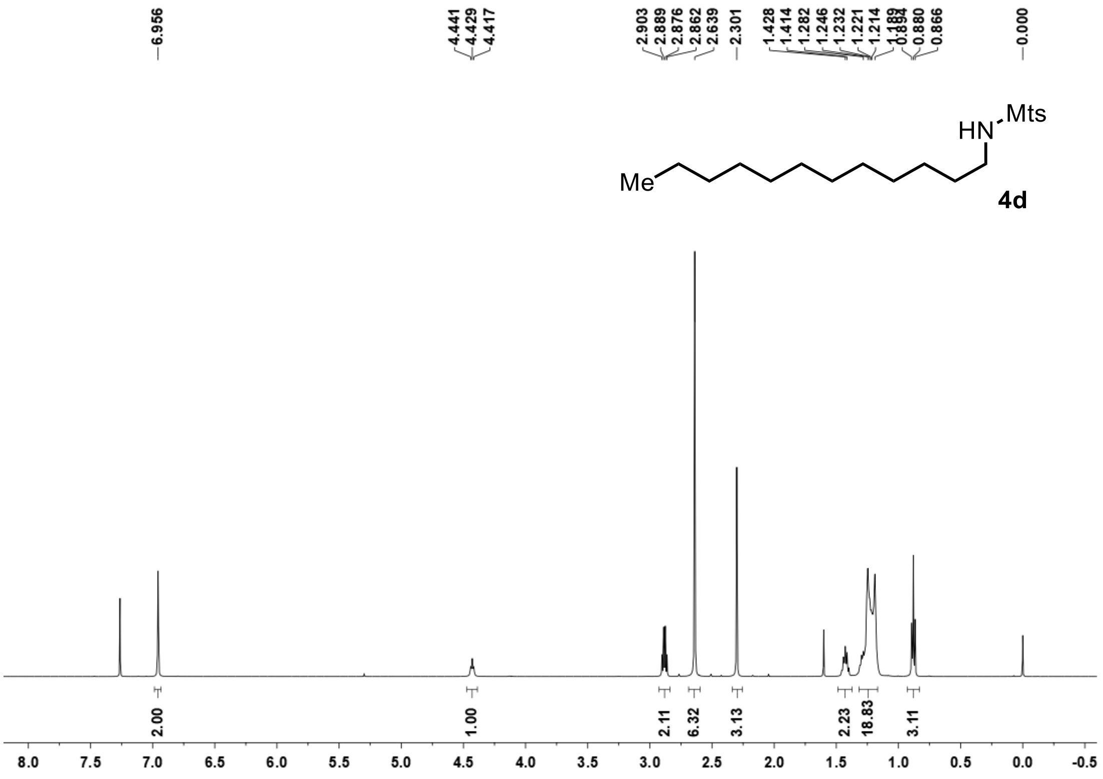

Supplementary Fig. 132 ^{1}H NMR (500 MHz, $CDCl_3$) of **4d**

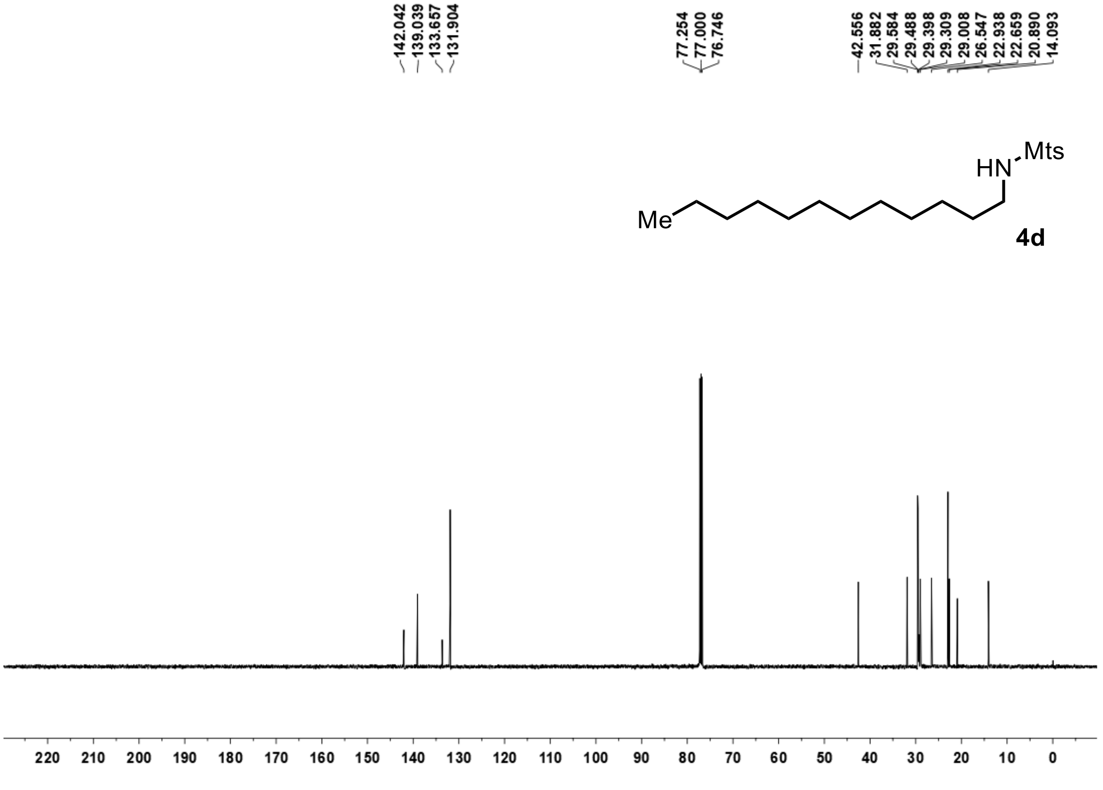

Supplementary Fig. 133 ^{13}C NMR (125 MHz, $CDCl_3$) of **4d**

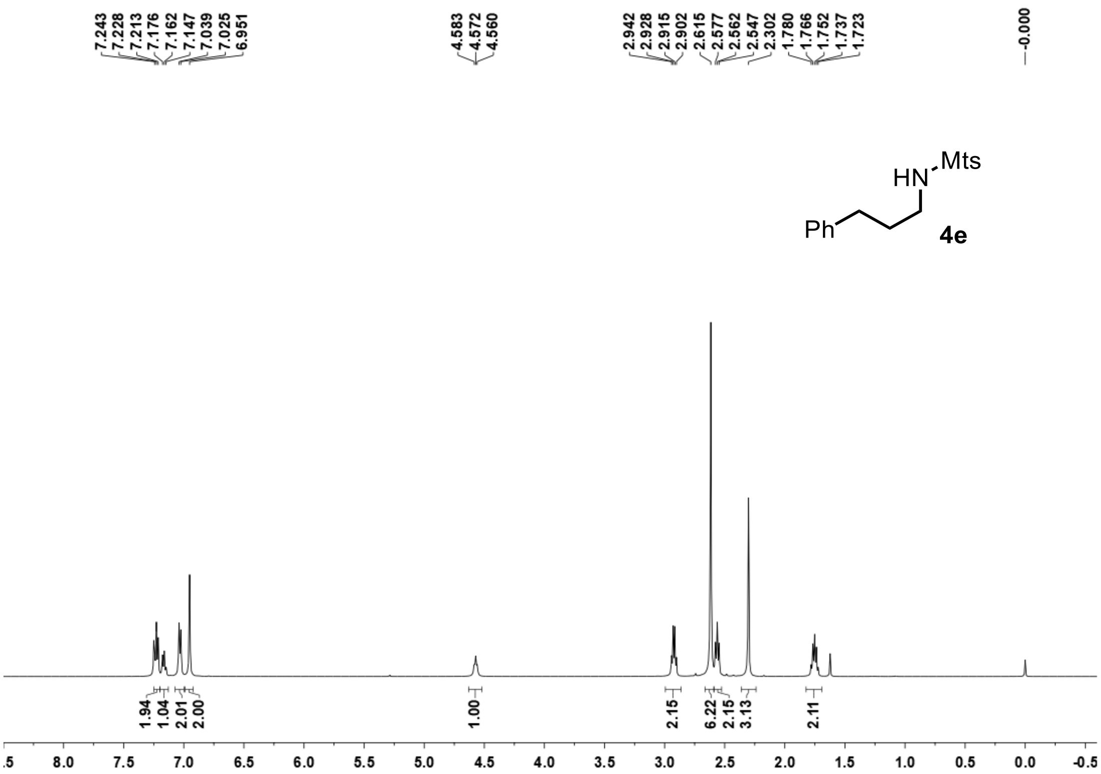

Supplementary Fig. 134 ^{1}H NMR (500 MHz, $CDCl_3$) of **4e**

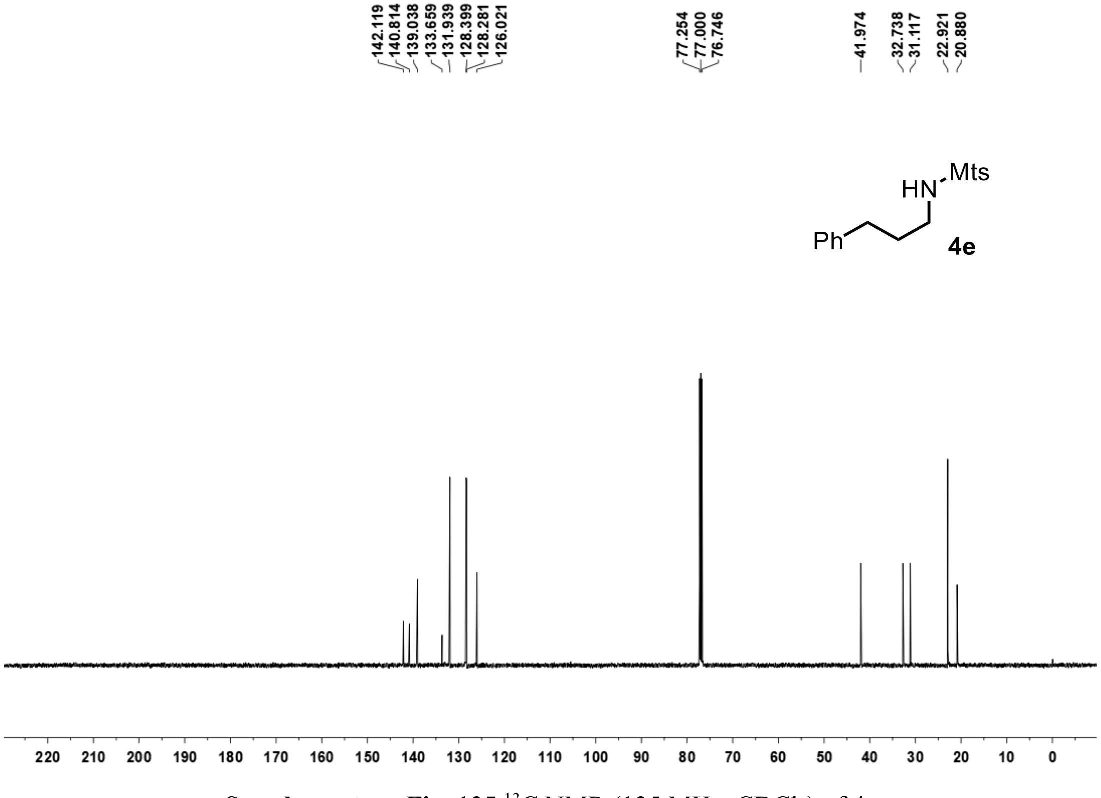

Supplementary Fig. 135 ^{13}C NMR (125 MHz, $CDCl_3$) of **4e**

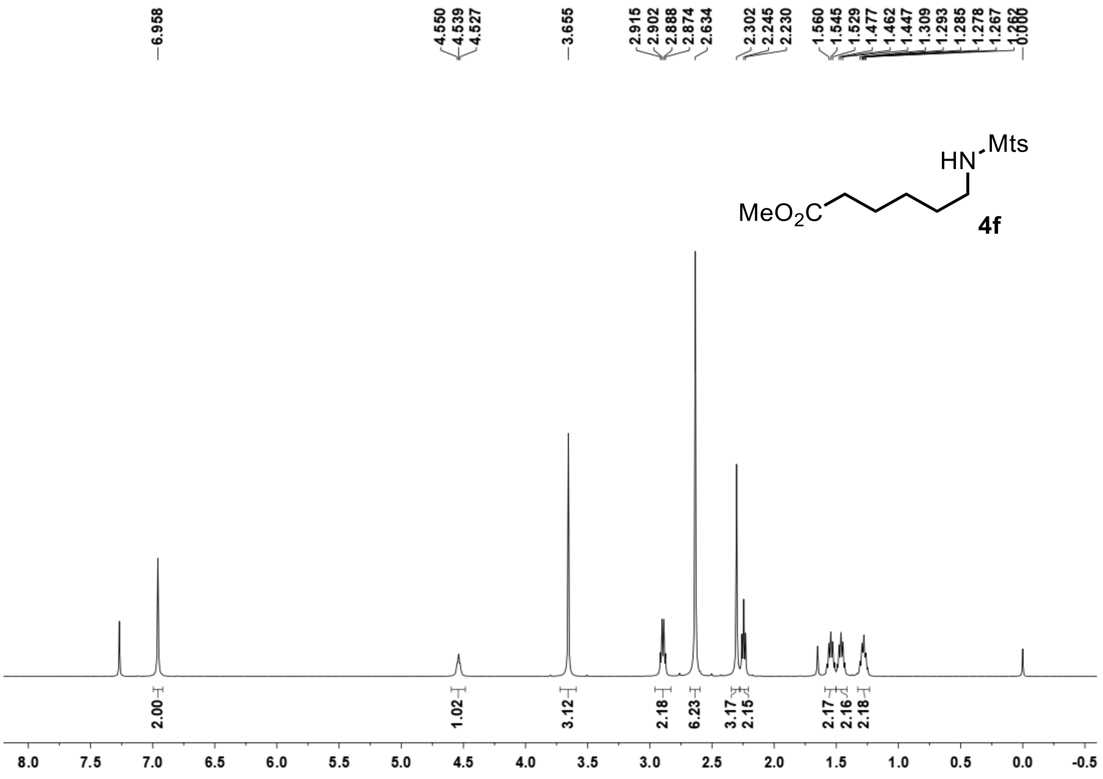

Supplementary Fig. 136 ^{1}H NMR (500 MHz, $CDCl_3$) of **4f**

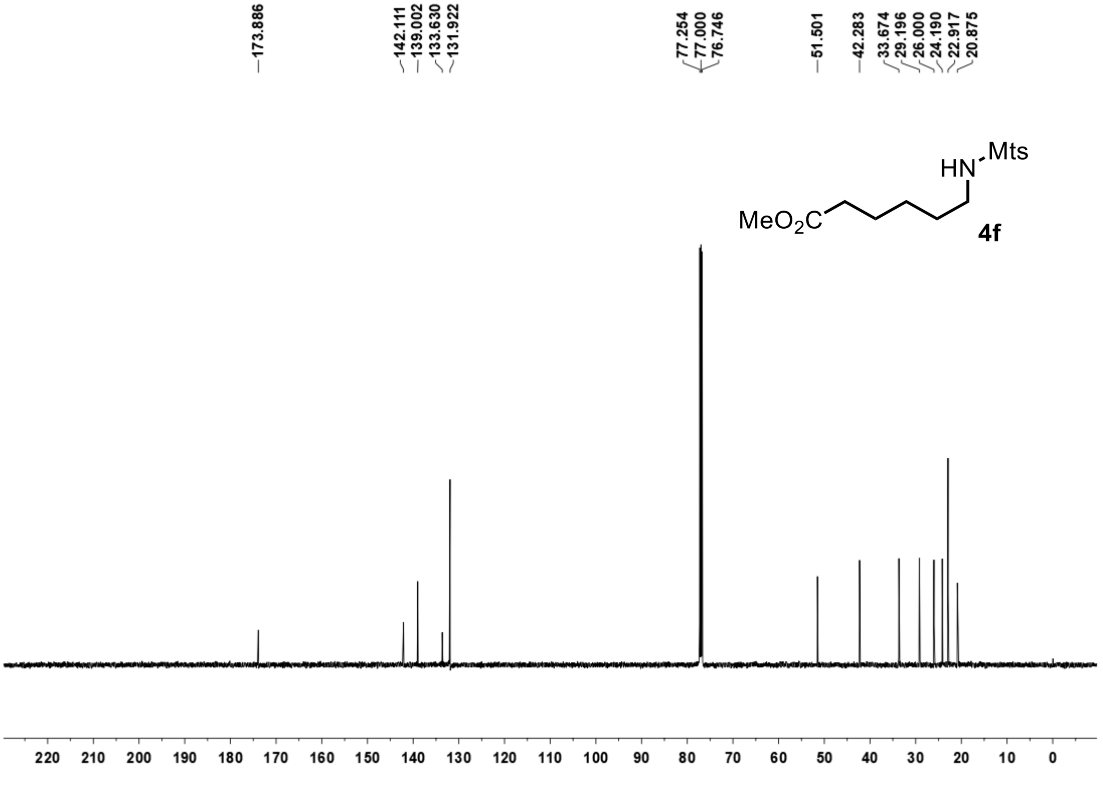

Supplementary Fig. 137 ^{13}C NMR (125 MHz, $CDCl_3$) of **4f**

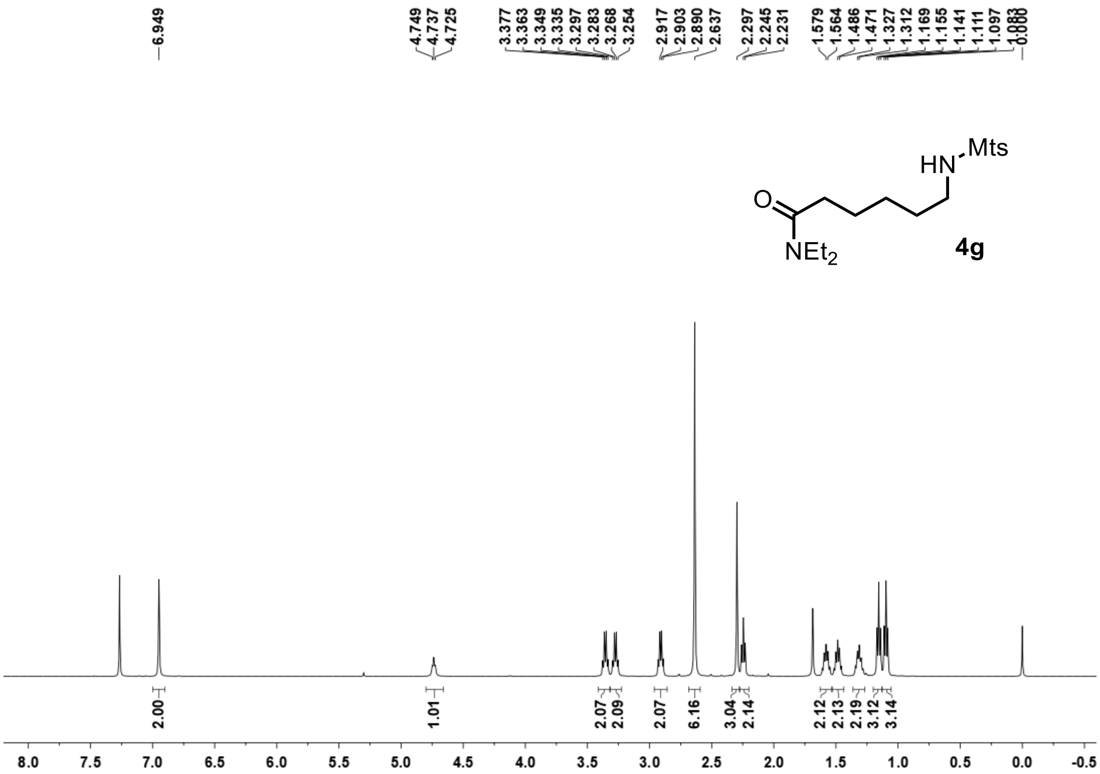

Supplementary Fig. 138 ^{1}H NMR (500 MHz, $CDCl_3$) of **4g**

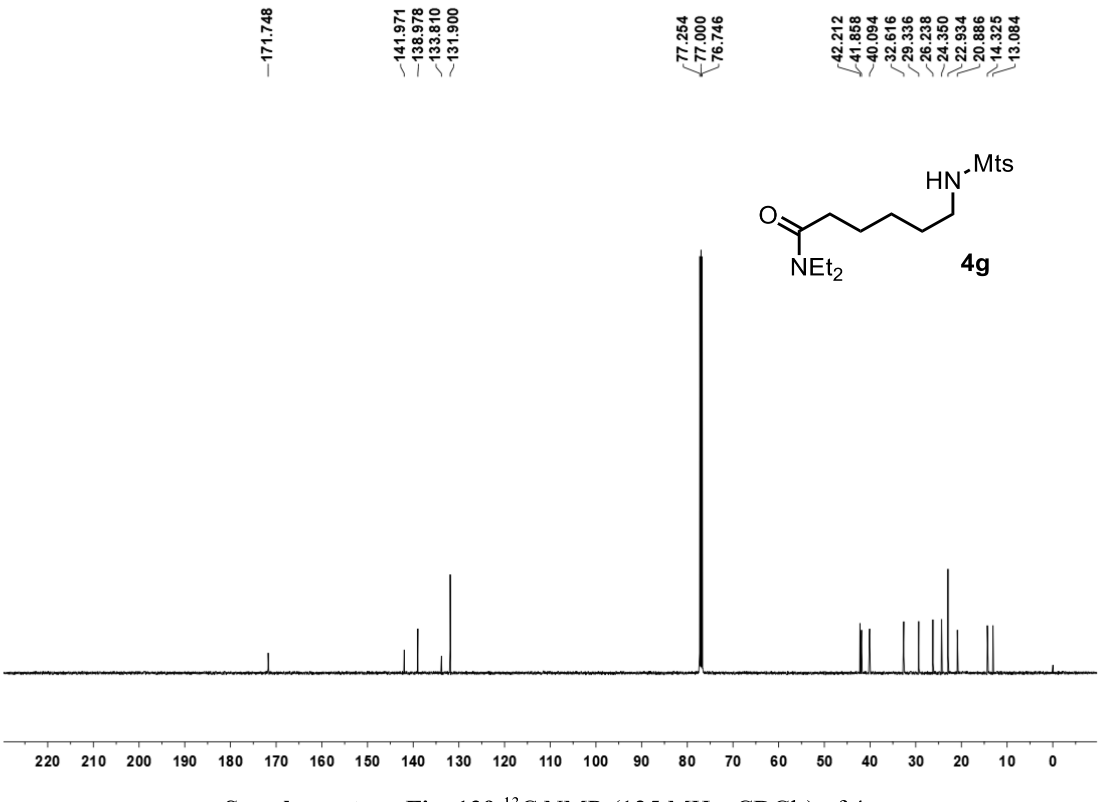

Supplementary Fig. 139 ^{13}C NMR (125 MHz, $CDCl_3$) of **4g**

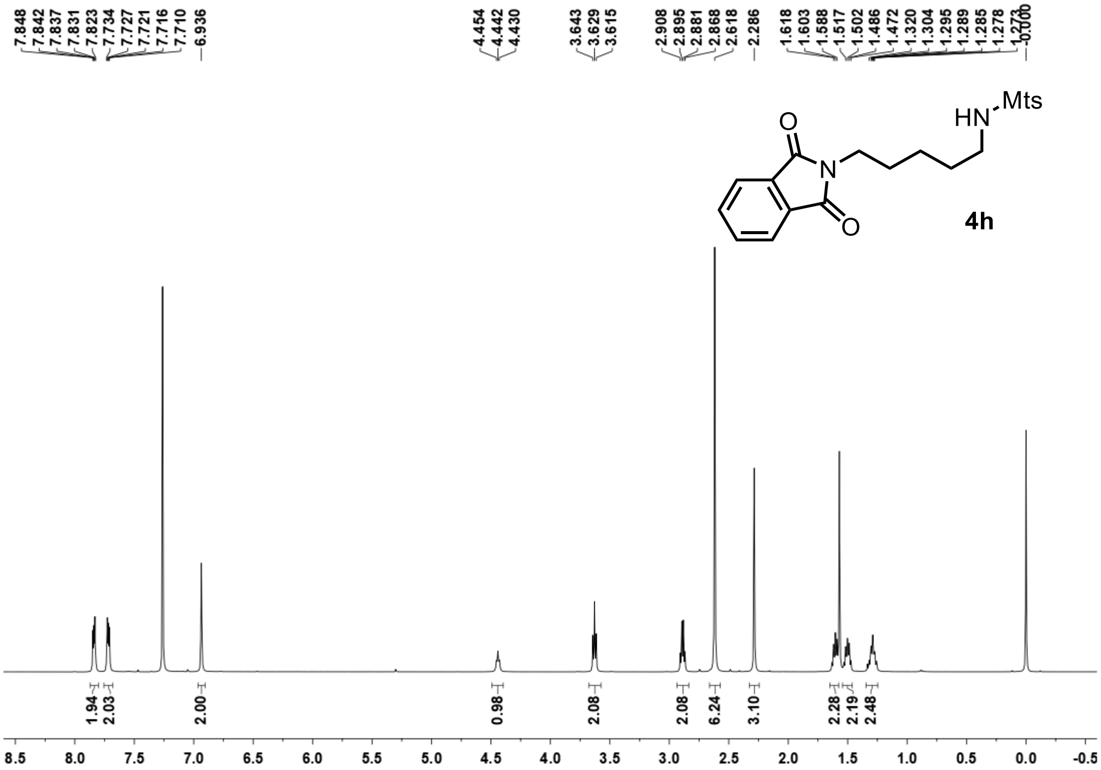

Supplementary Fig. 140 ^{1}H NMR (500 MHz, $CDCl_3$) of **4h**

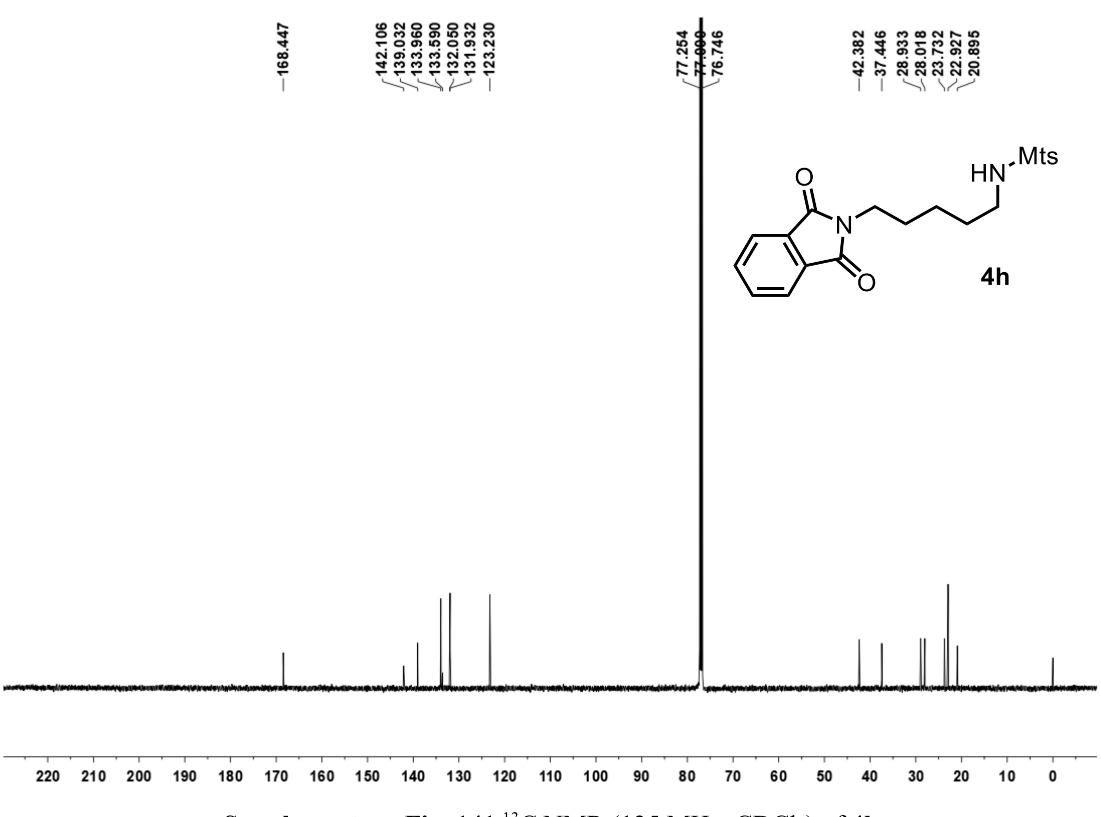

Supplementary Fig. 141 ^{13}C NMR (125 MHz, $CDCl_3$) of **4h**

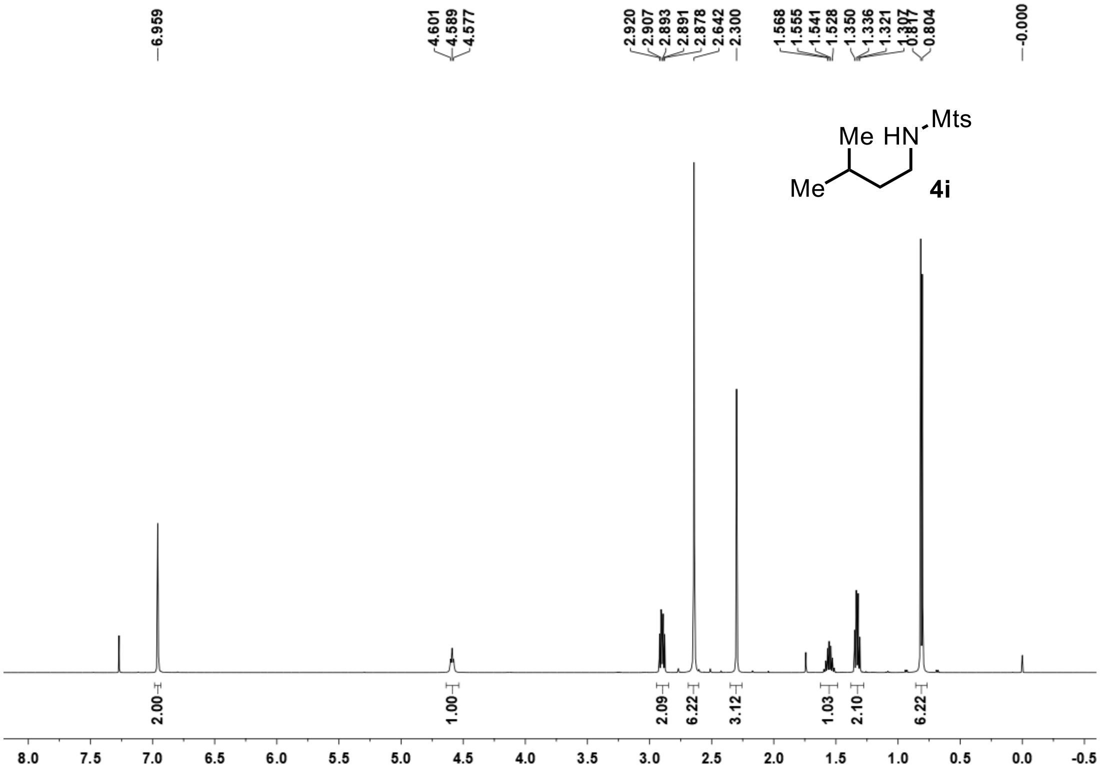

Supplementary Fig. 142 ^{1}H NMR (500 MHz, $CDCl_3$) of **4i**

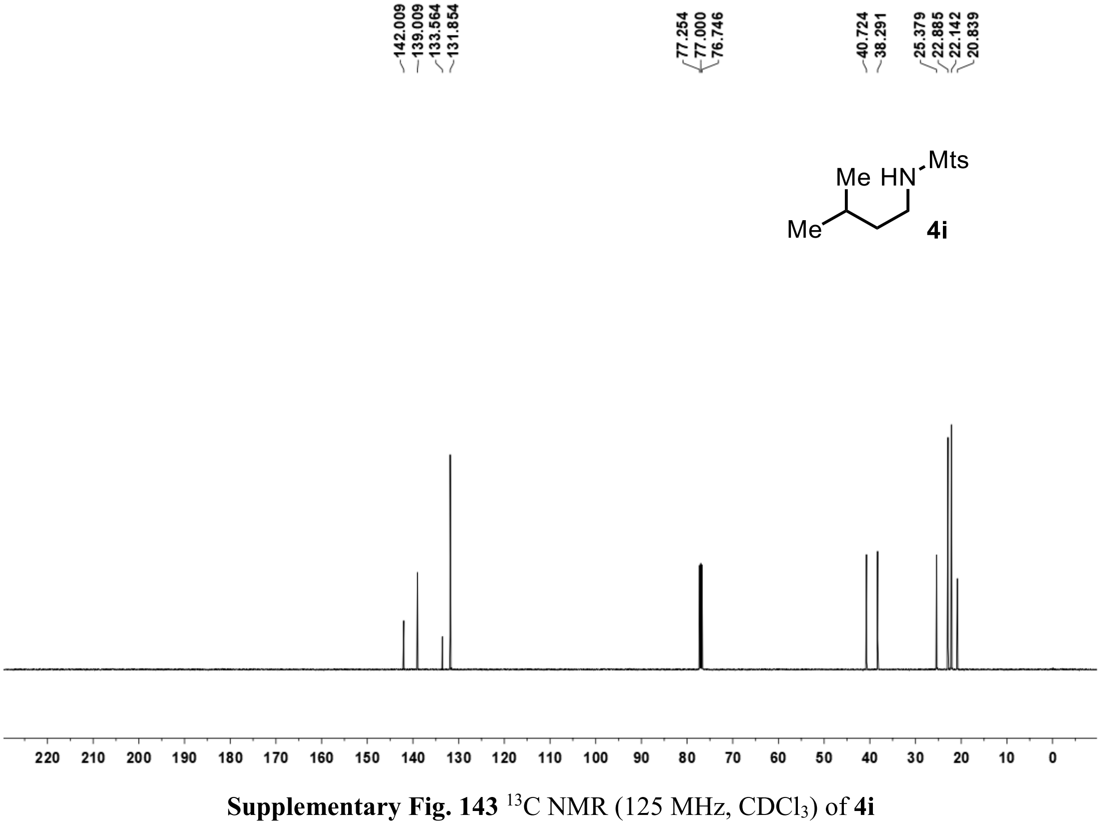

Supplementary Fig. 143 ^{13}C NMR (125 MHz, $CDCl_3$) of **4i**

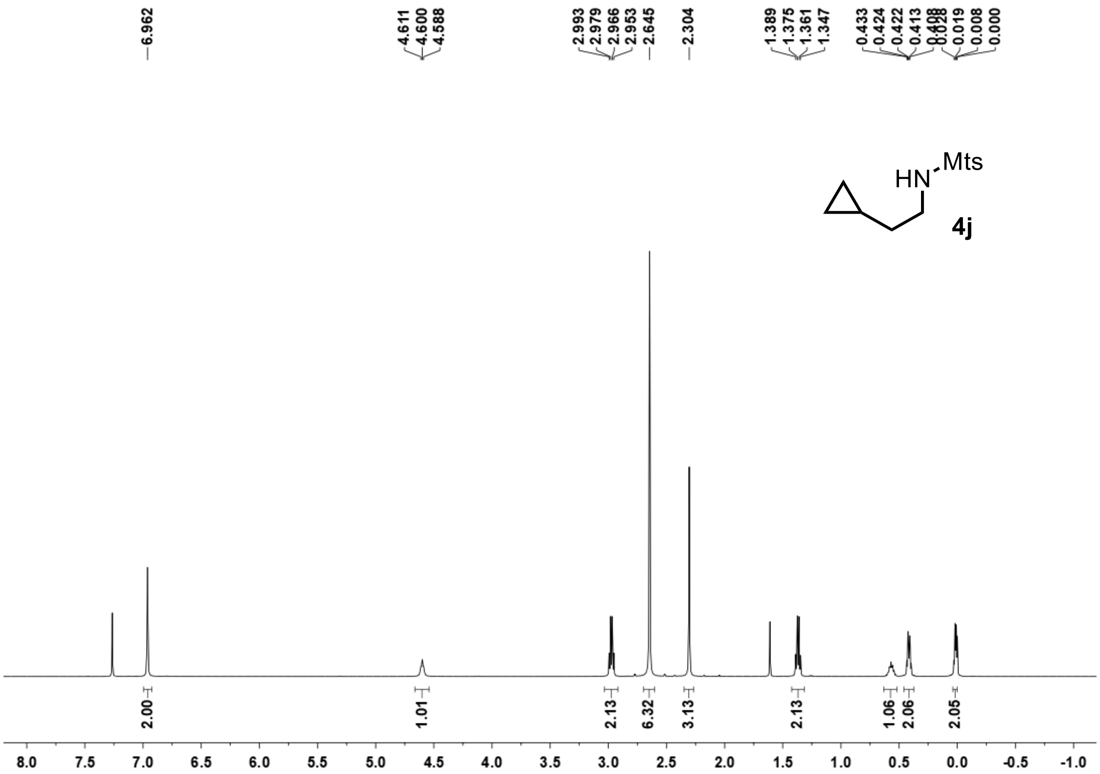

Supplementary Fig. 144 ^{1}H NMR (500 MHz, $CDCl_3$) of **4j**

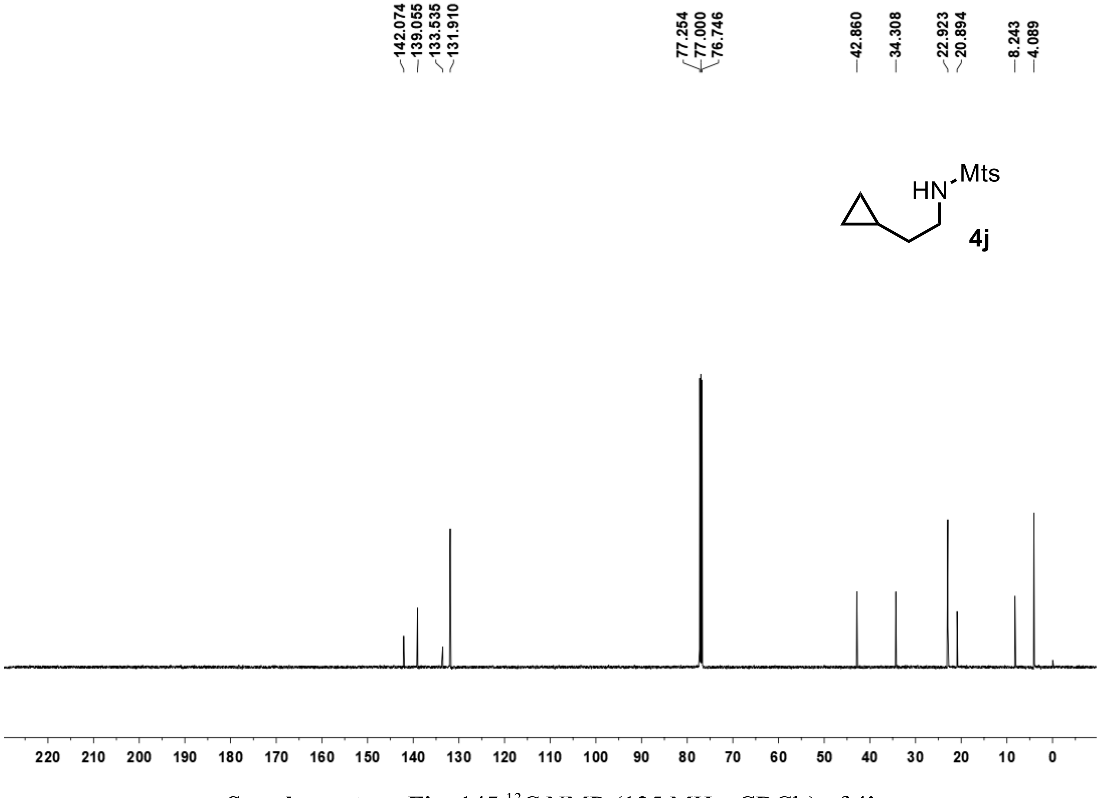

Supplementary Fig. 145 ^{13}C NMR (125 MHz, $CDCl_3$) of **4j**

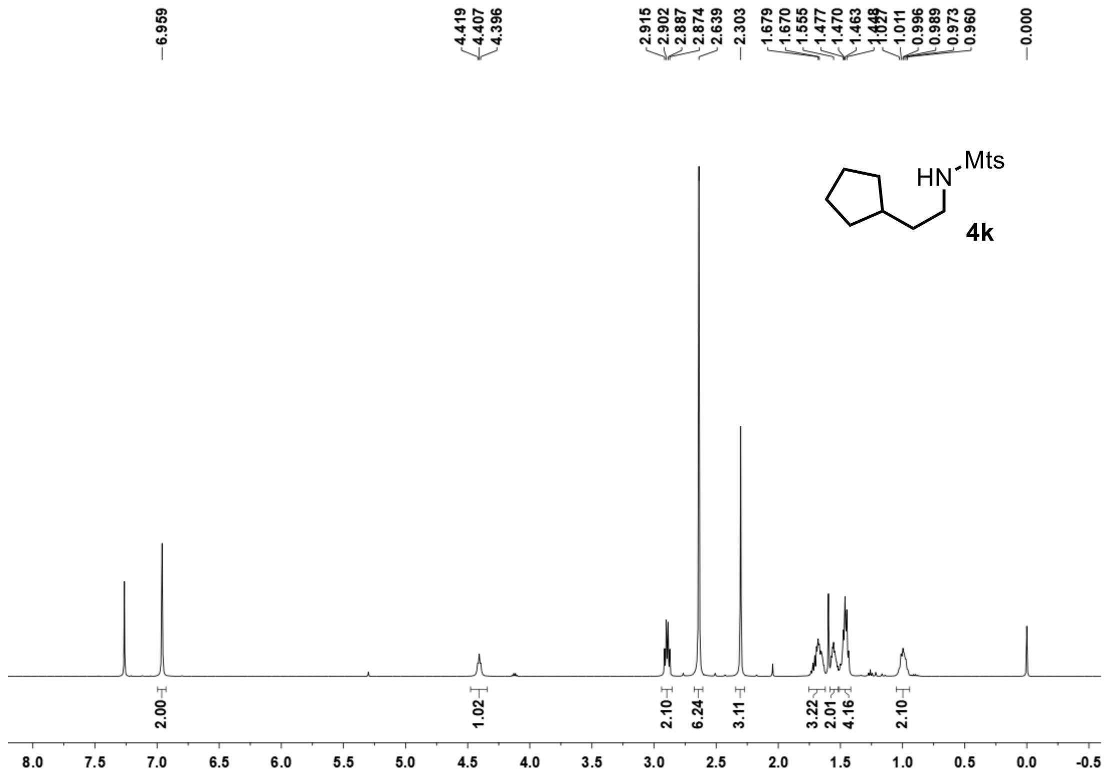

Supplementary Fig. 146 ^{1}H NMR (500 MHz, $CDCl_3$) of **4k**

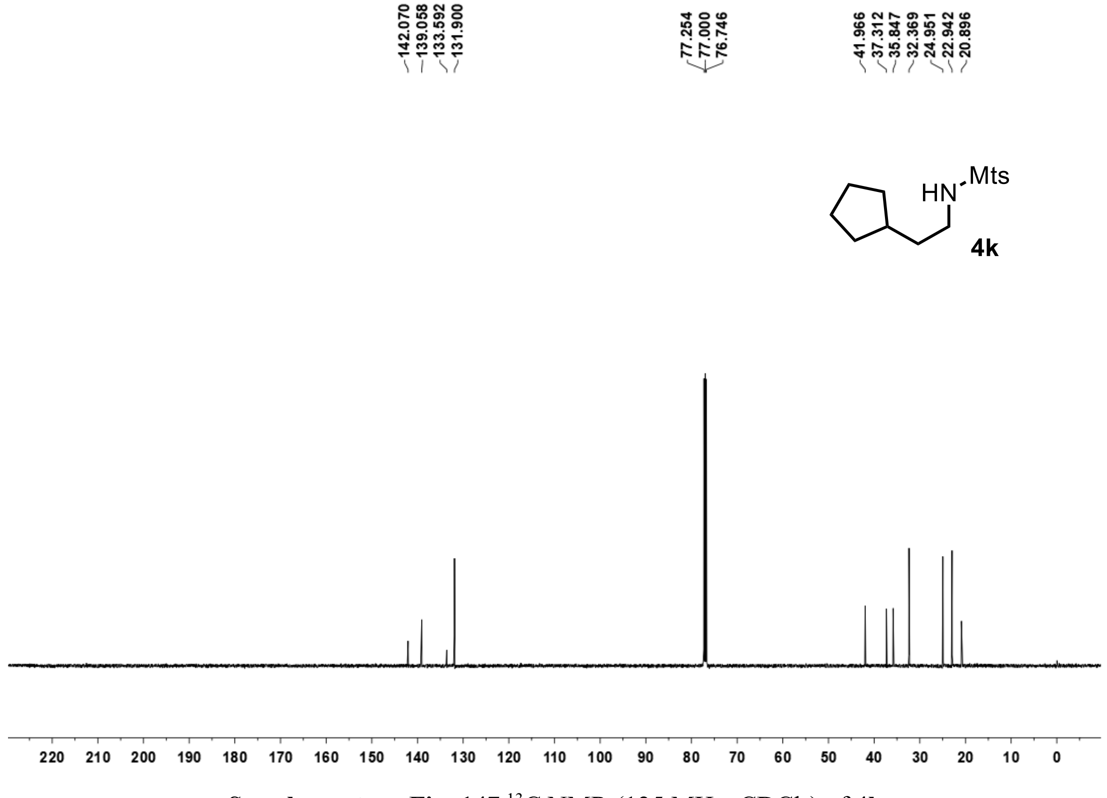

Supplementary Fig. 147 ^{13}C NMR (125 MHz, $CDCl_3$) of **4k**

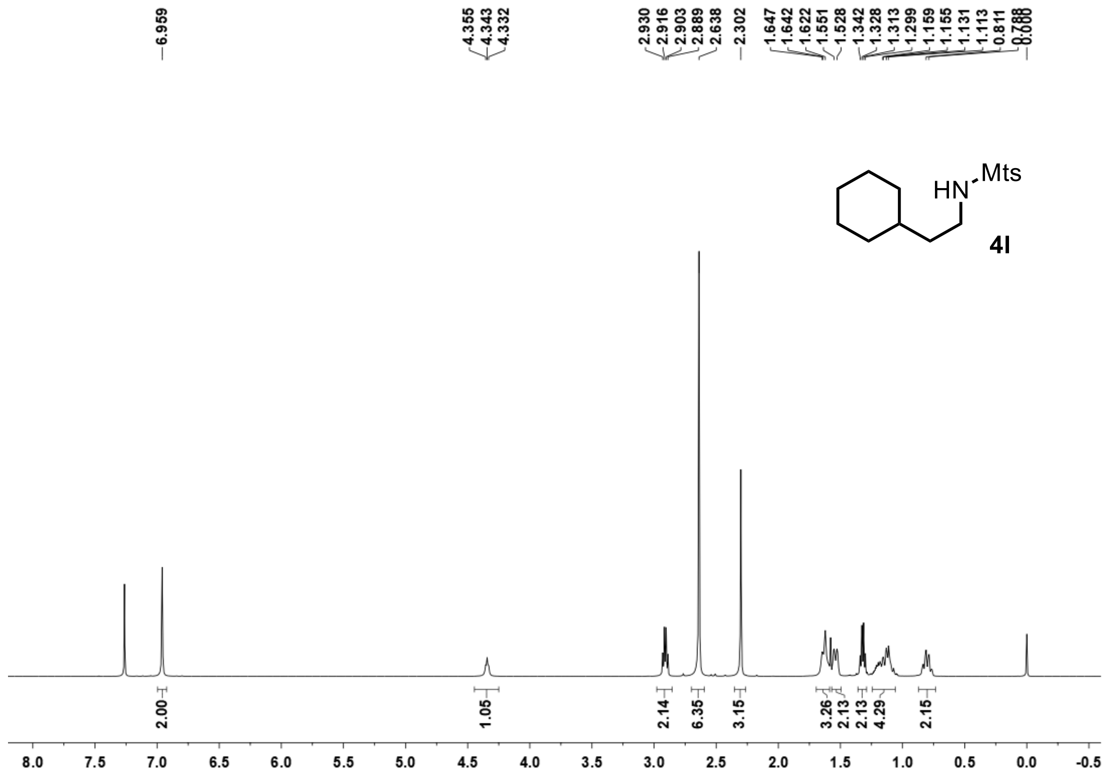

Supplementary Fig. 148 ^{1}H NMR (500 MHz, $CDCl_3$) of **4l**

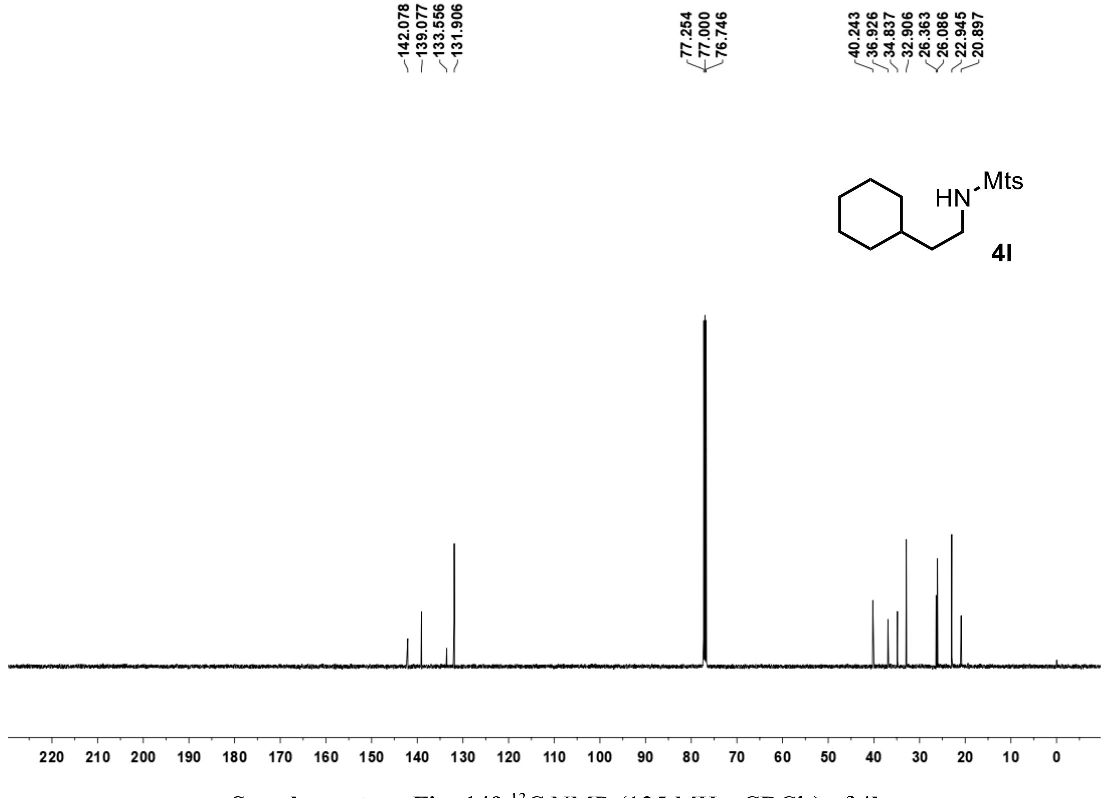

Supplementary Fig. 149 ^{13}C NMR (125 MHz, $CDCl_3$) of **4l**

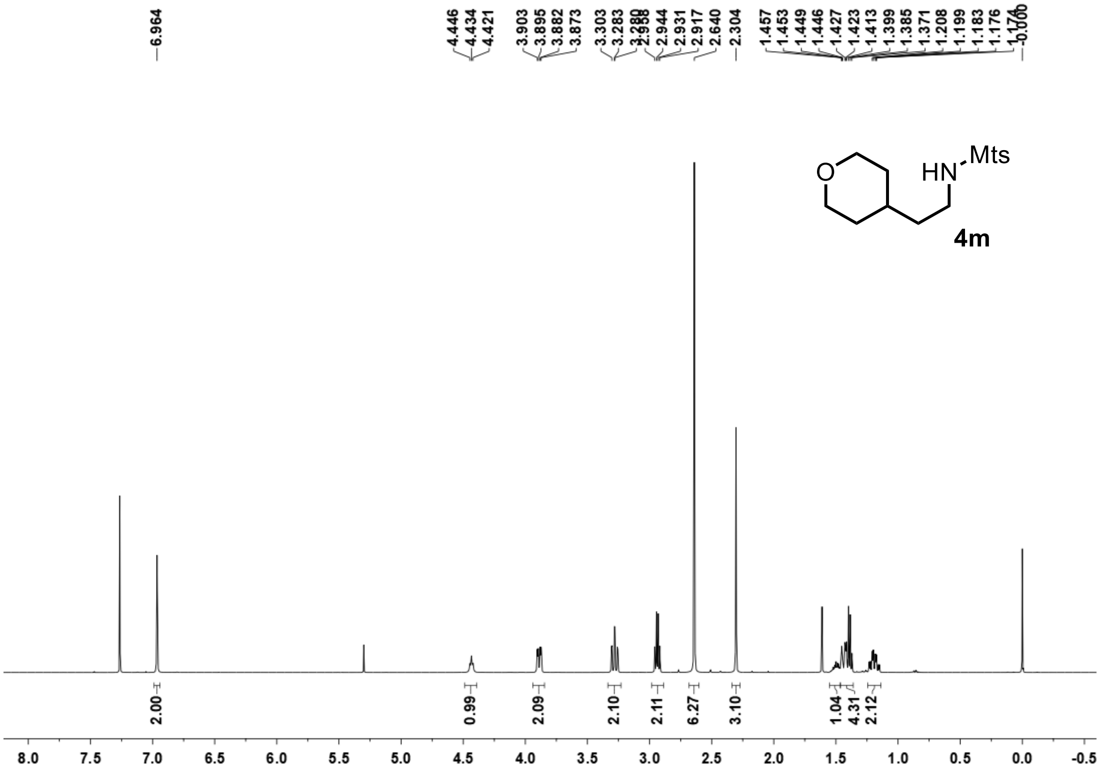

Supplementary Fig. 150 ^{1}H NMR (500 MHz, $CDCl_3$) of **4m**

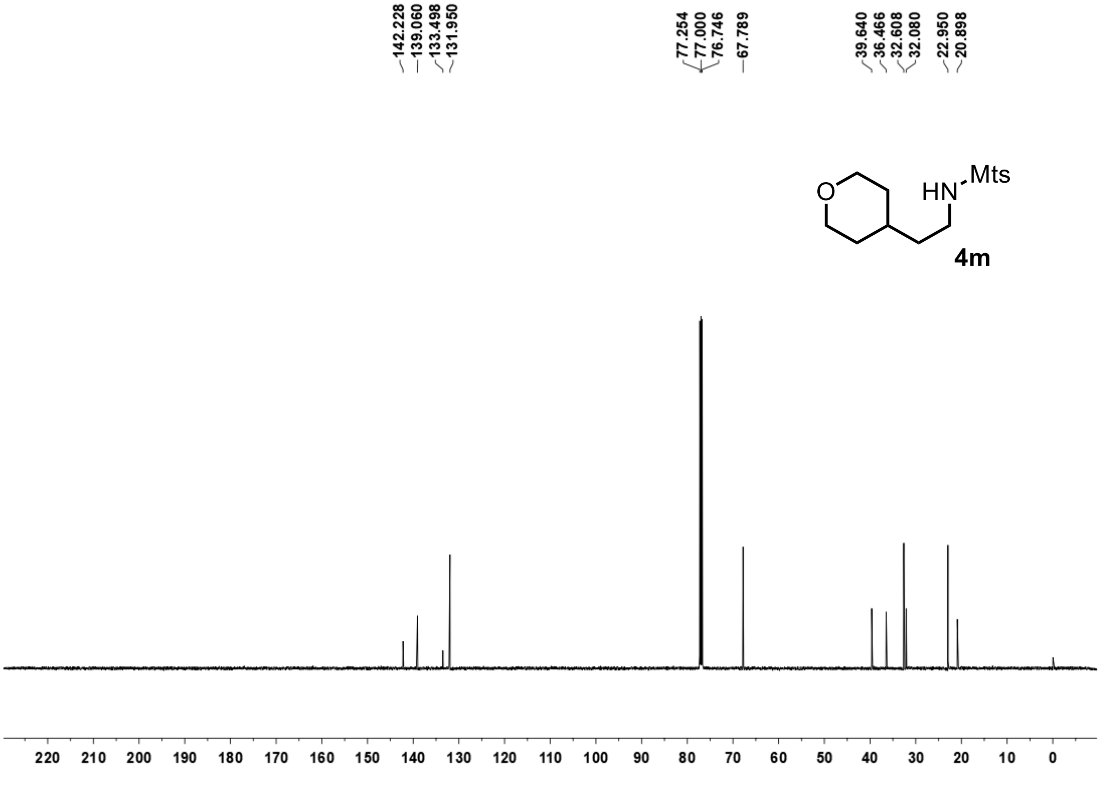

Supplementary Fig. 151 ^{13}C NMR (125 MHz, $CDCl_3$) of **4m**

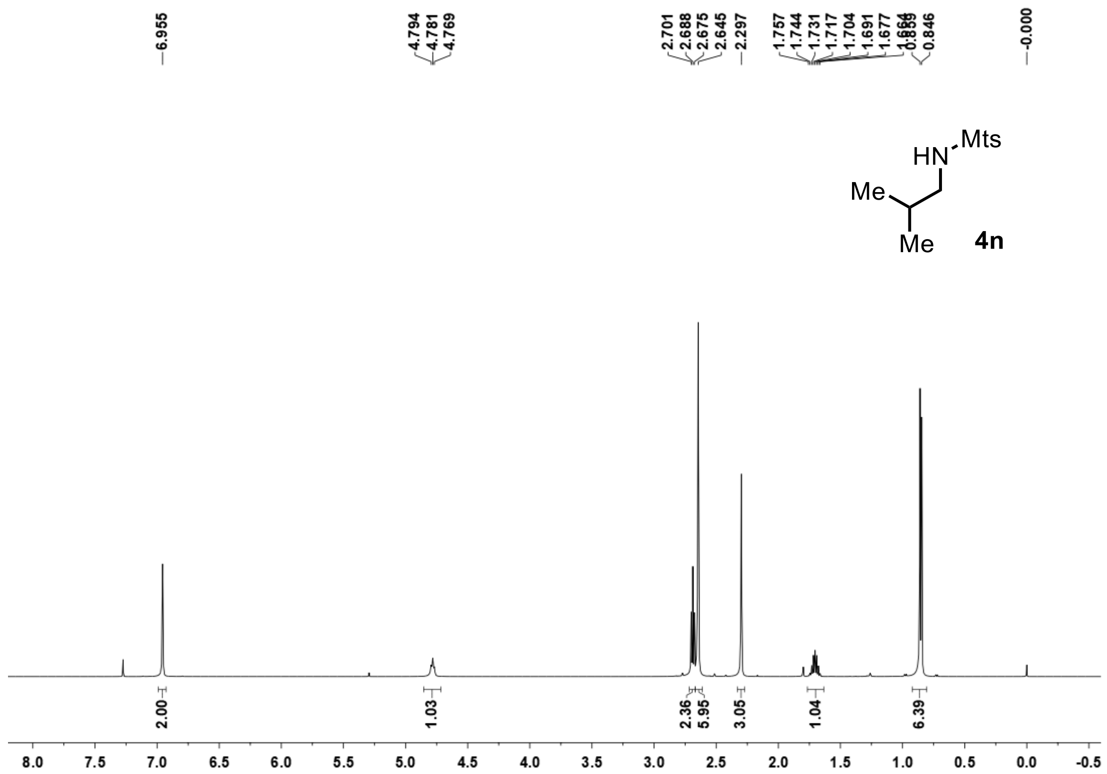

Supplementary Fig. 152 ^{1}H NMR (500 MHz, $CDCl_3$) of **4n**

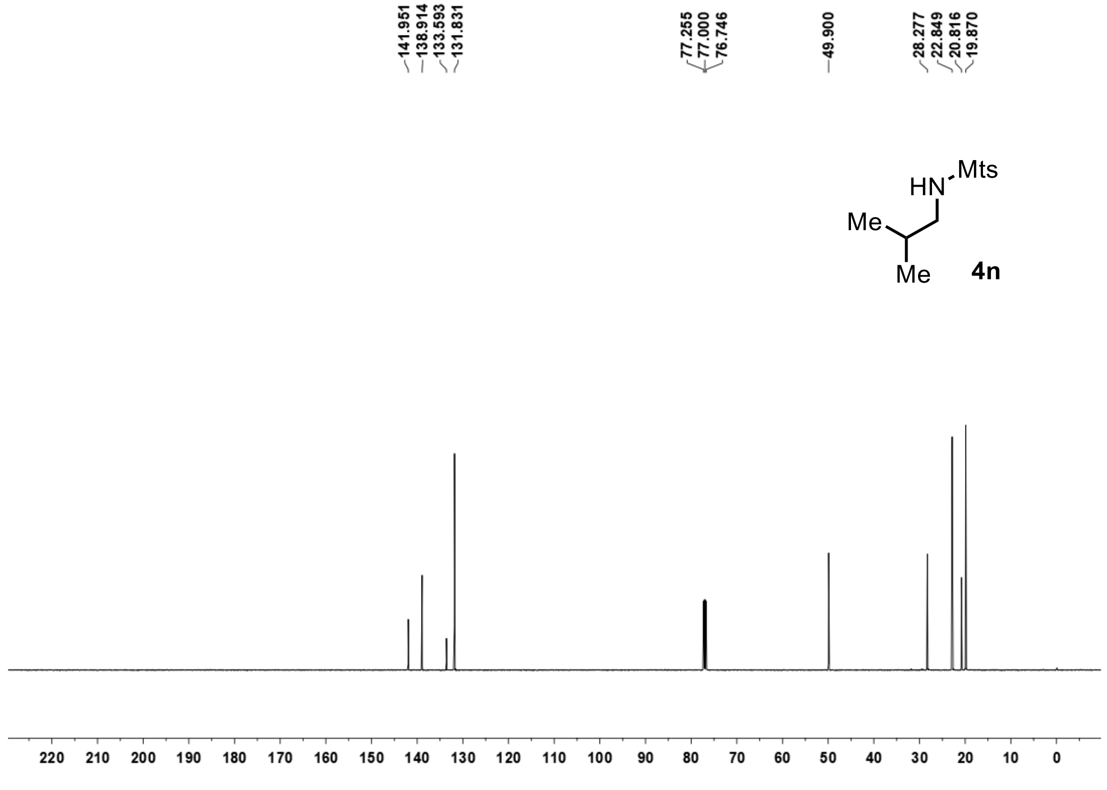

Supplementary Fig. 153 ^{13}C NMR (125 MHz, $CDCl_3$) of **4n**

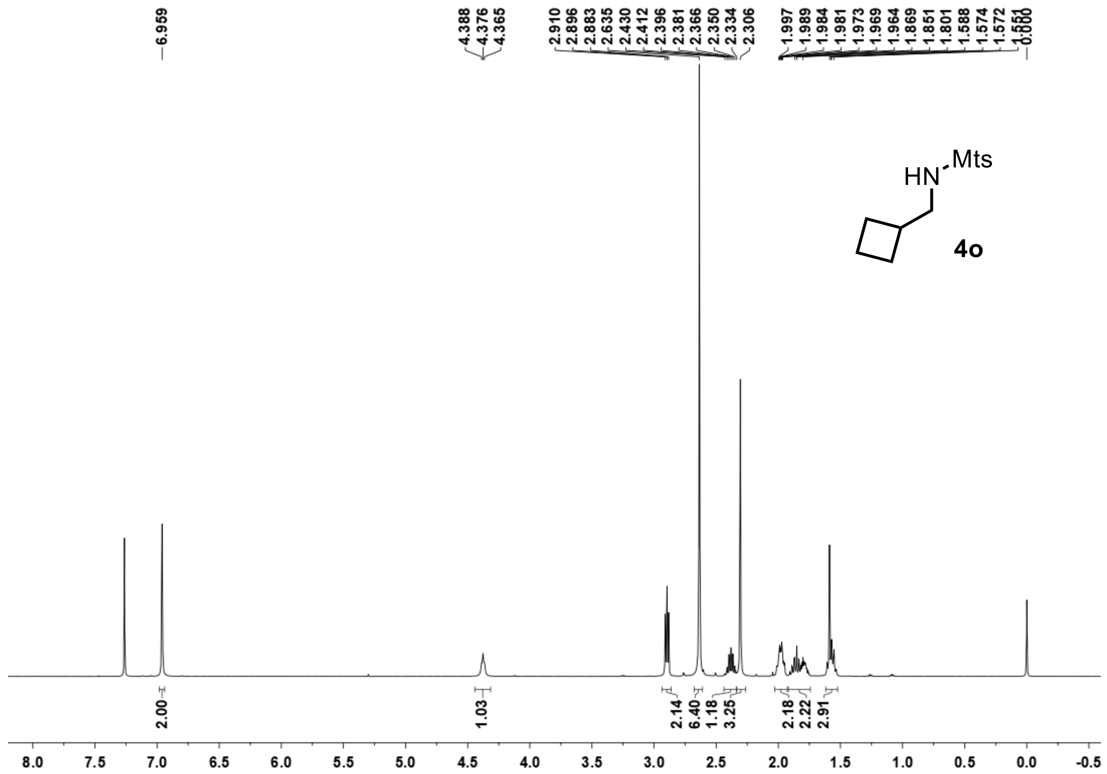

Supplementary Fig. 154 ^{1}H NMR (500 MHz, $CDCl_3$) of **4o**

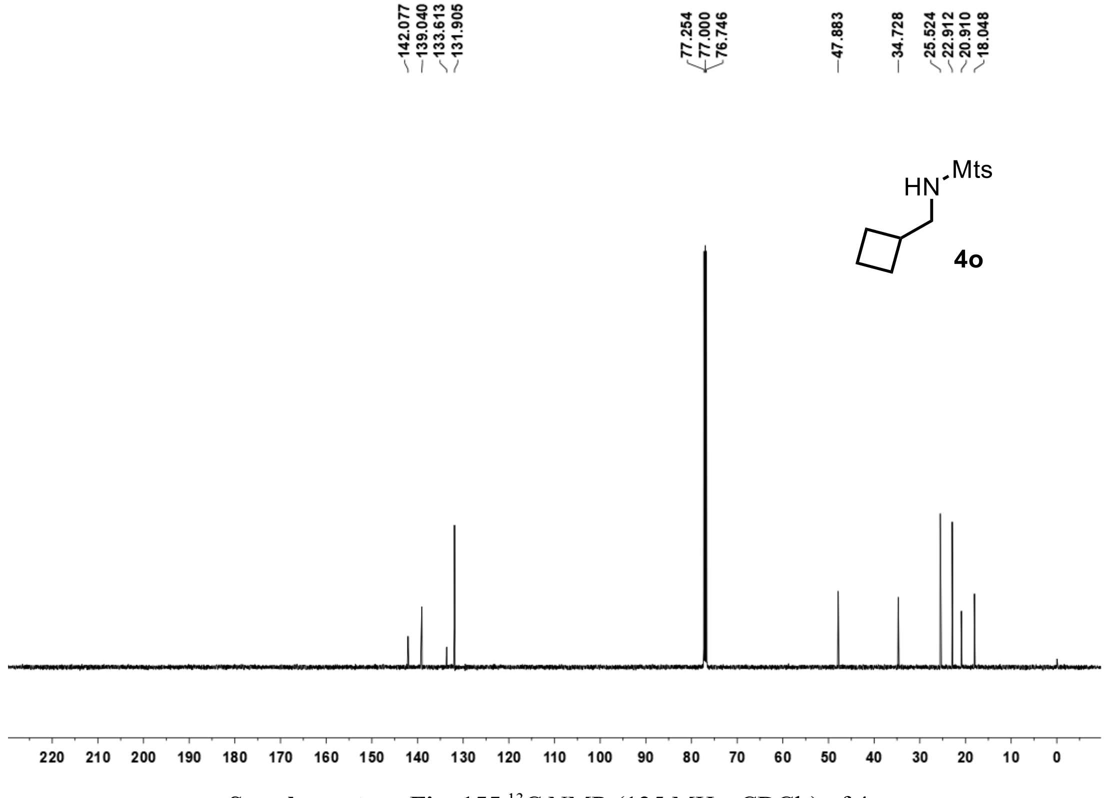

Supplementary Fig. 155 ^{13}C NMR (125 MHz, $CDCl_3$) of **4o**

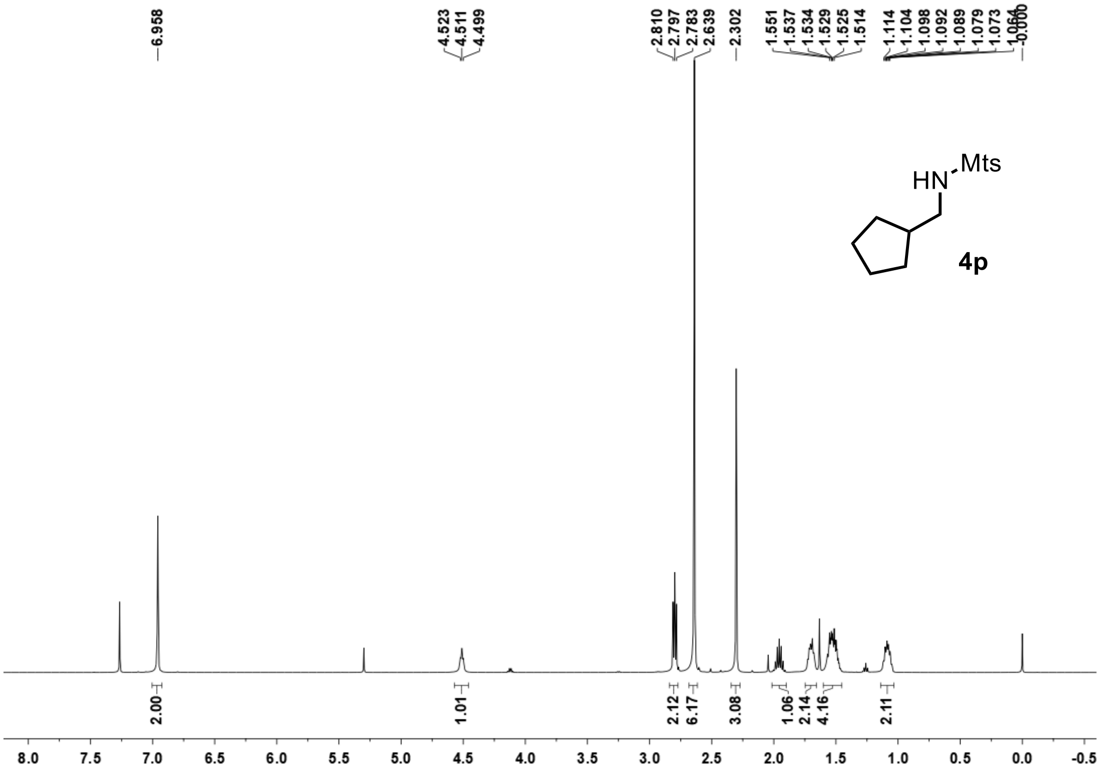

Supplementary Fig. 156 ^{1}H NMR (500 MHz, $CDCl_3$) of **4p**

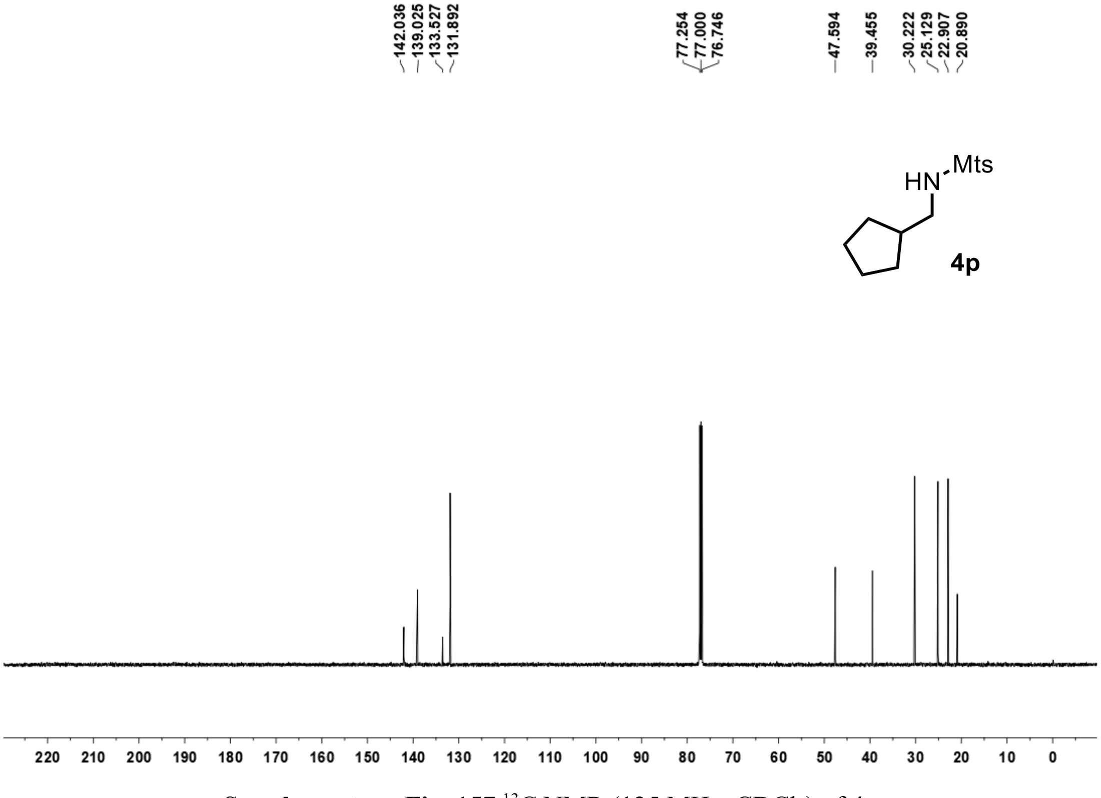

Supplementary Fig. 157 ^{13}C NMR (125 MHz, $CDCl_3$) of **4p**

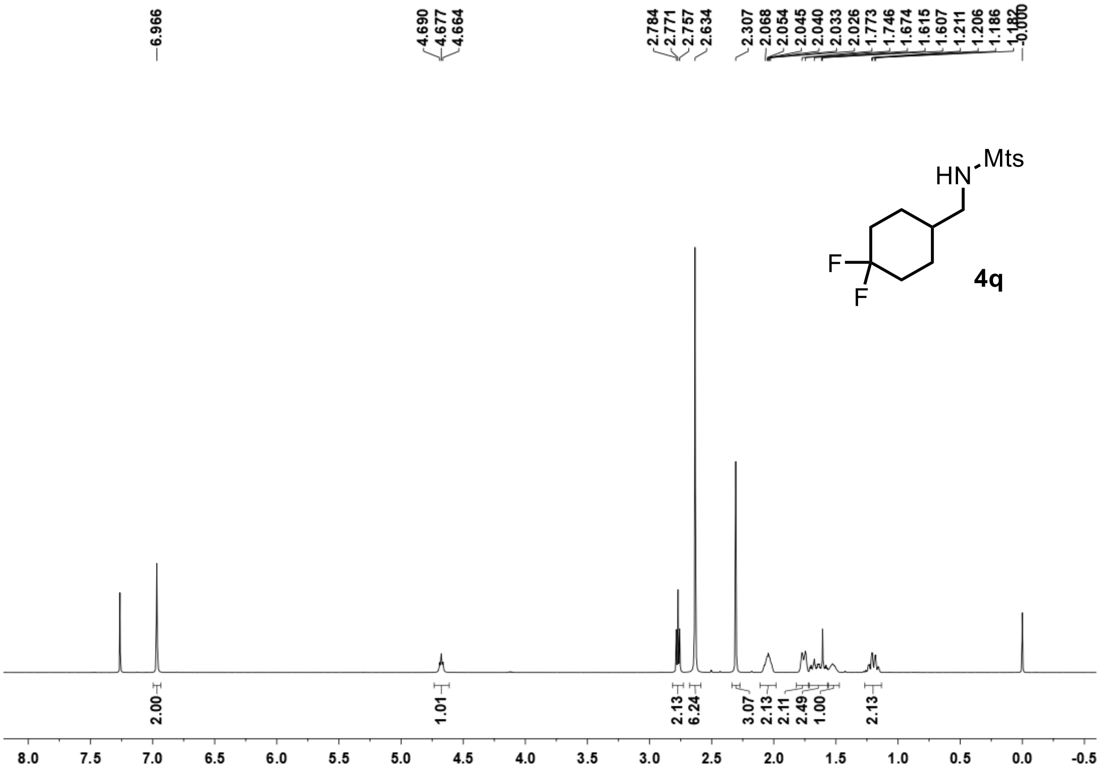

Supplementary Fig. 158 ^{1}H NMR (500 MHz, $CDCl_3$) of **4q**

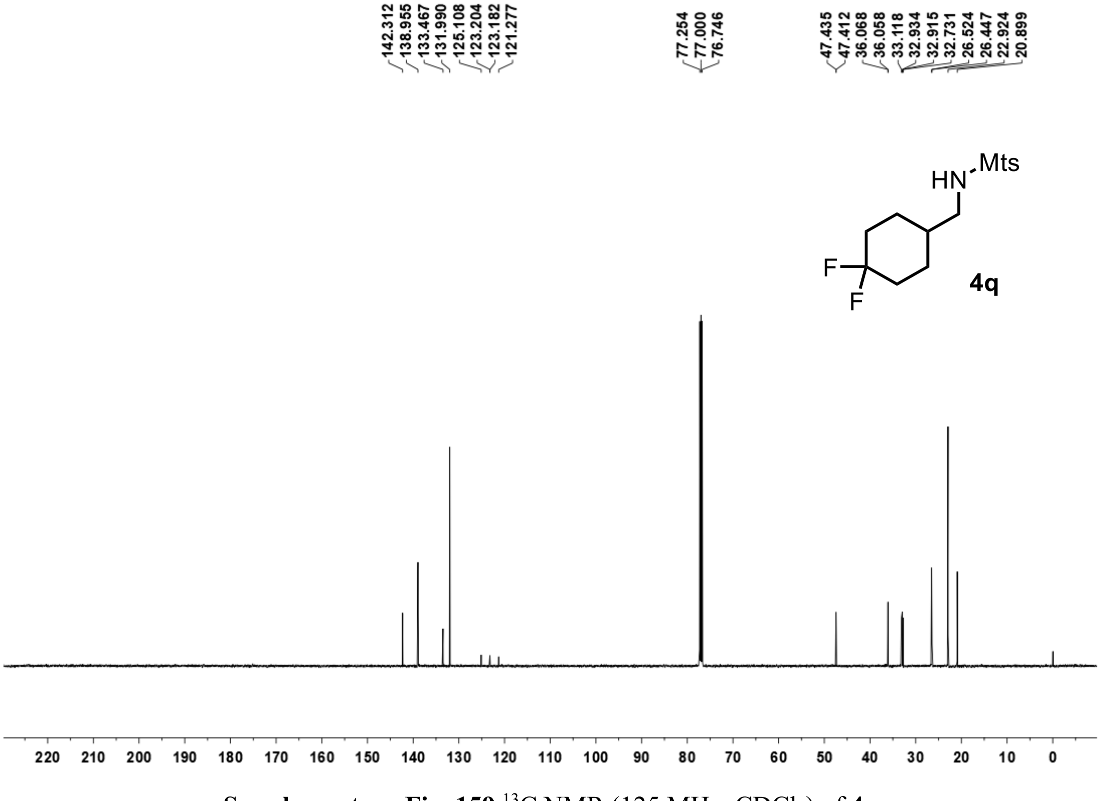

Supplementary Fig. 159 ^{13}C NMR (125 MHz, $CDCl_3$) of **4q**

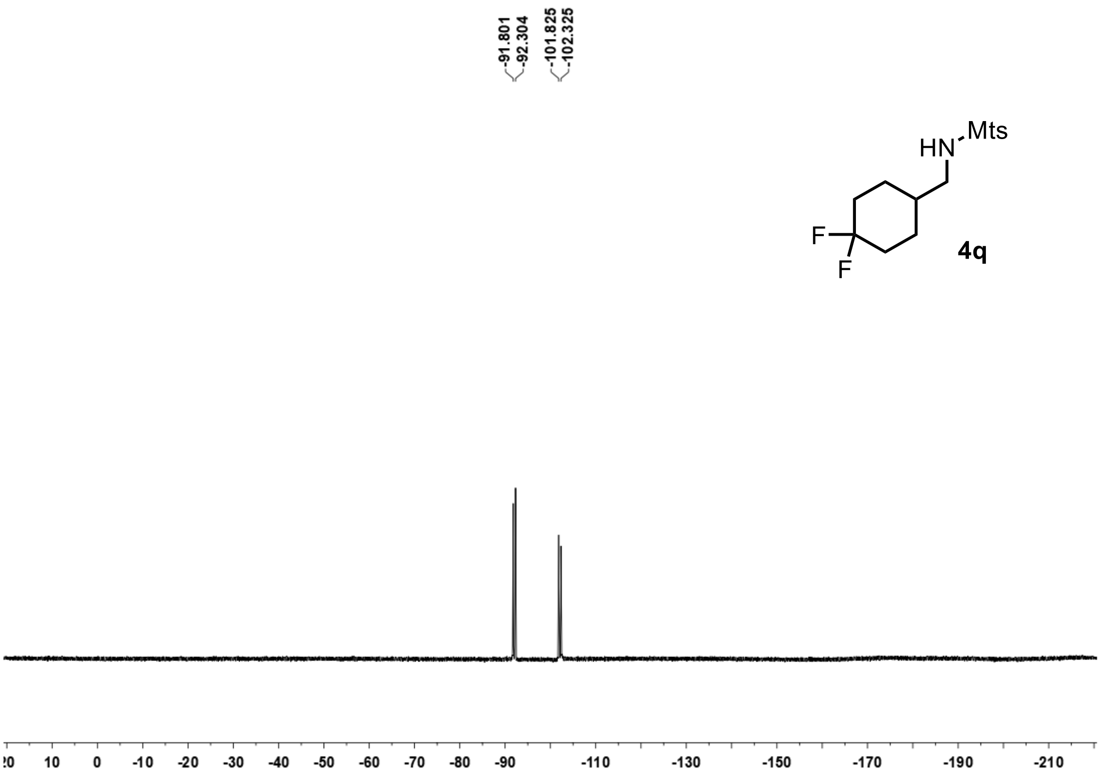

Supplementary Fig. 160 ^{19}F NMR (470 MHz, $CDCl_3$) of **4q**

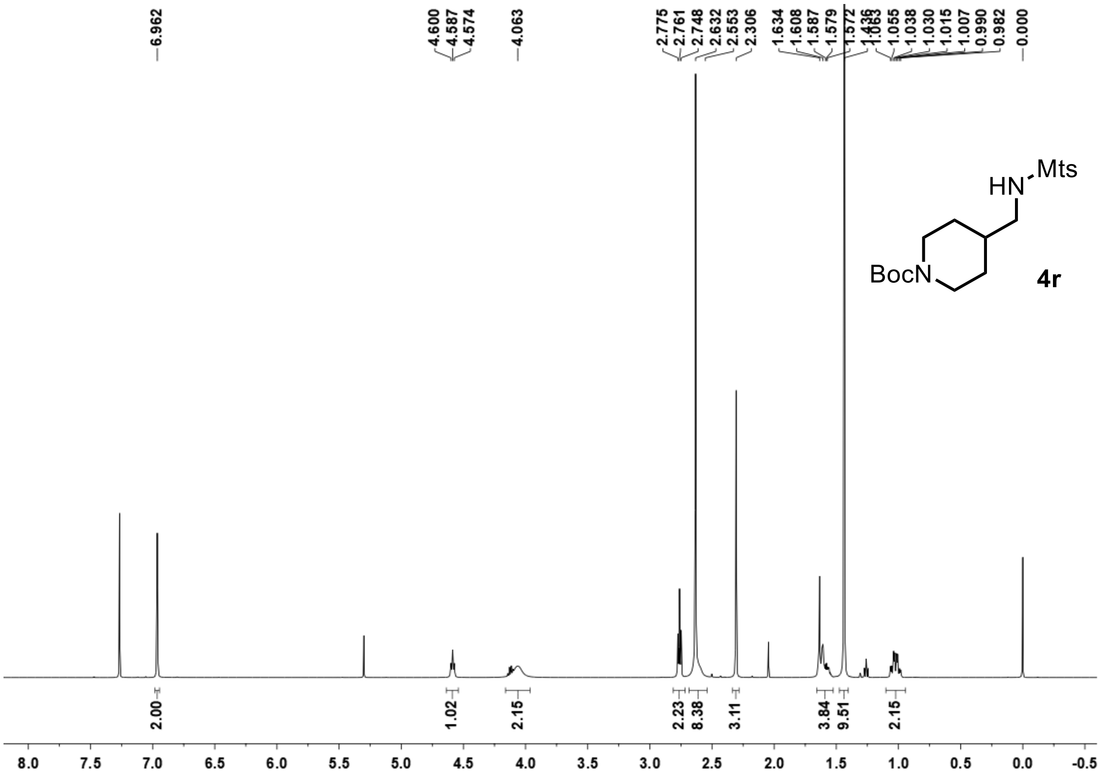

Supplementary Fig. 161 ^{1}H NMR (500 MHz, $CDCl_3$) of **4r**

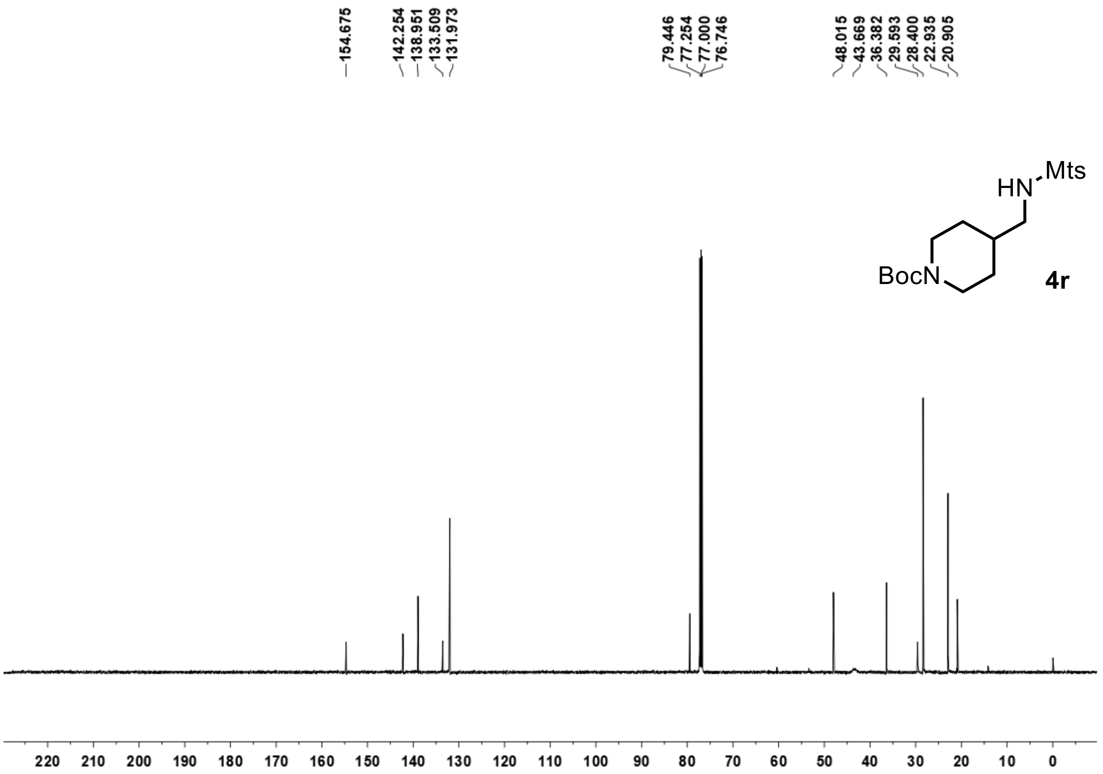

Supplementary Fig. 162 ^{13}C NMR (125 MHz, $CDCl_3$) of **4r**

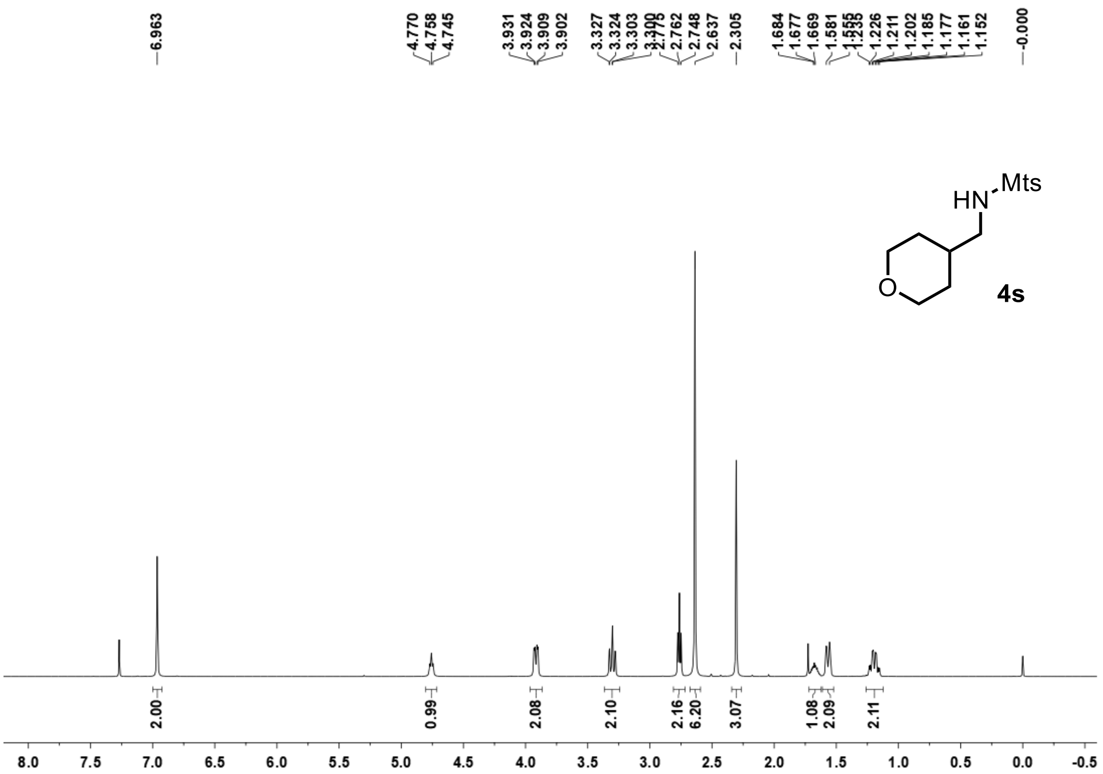

Supplementary Fig. 163 ^{1}H NMR (500 MHz, $CDCl_3$) of **4s**

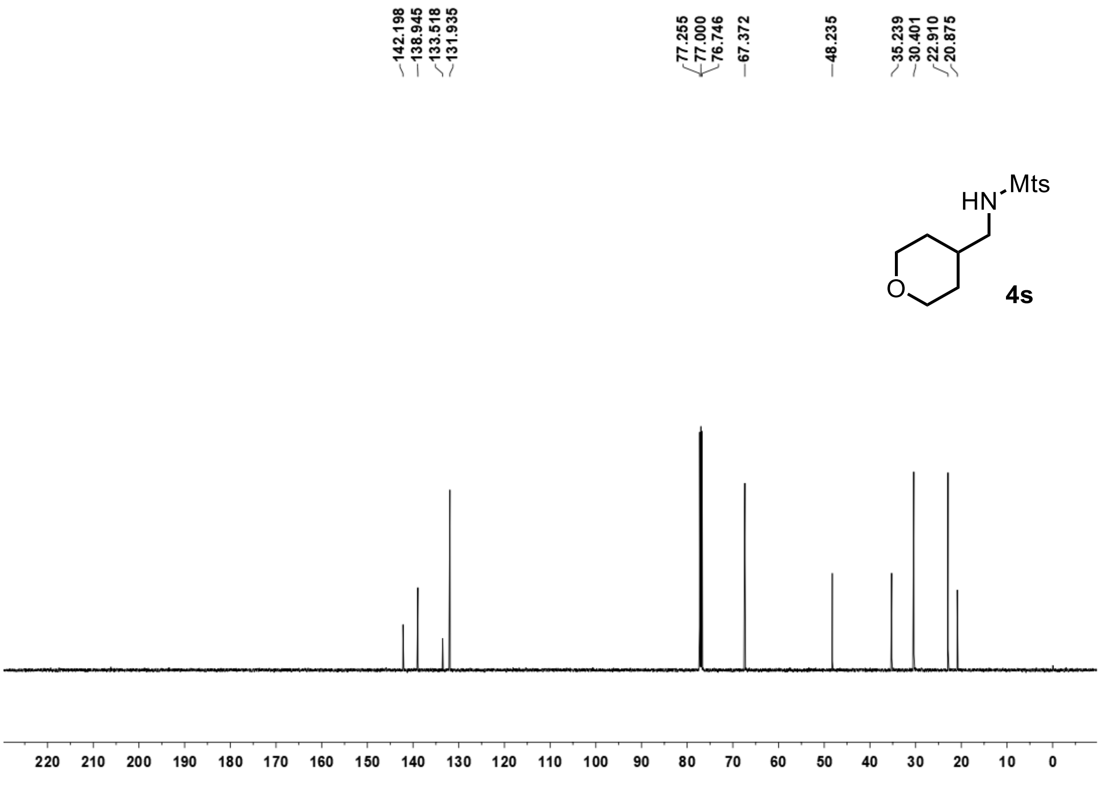

Supplementary Fig. 164 ^{13}C NMR (125 MHz, $CDCl_3$) of **4s**

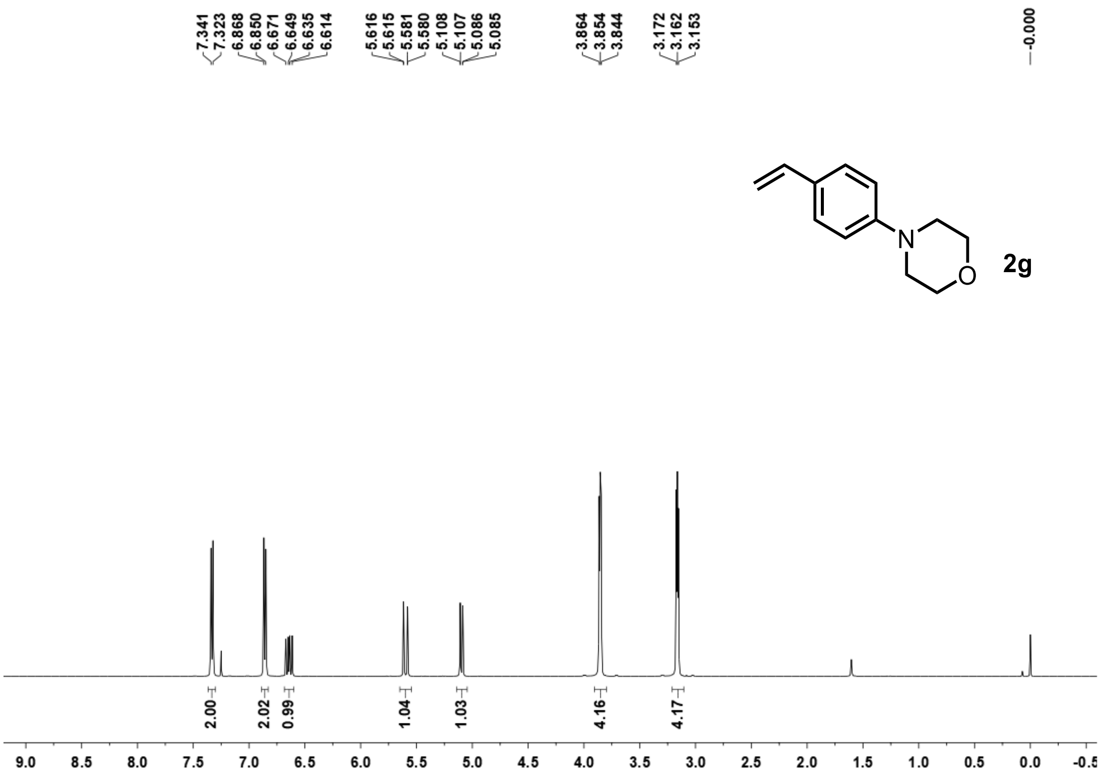

Supplementary Fig. 165 ^{1}H NMR (500 MHz, $CDCl_3$) of **2g**

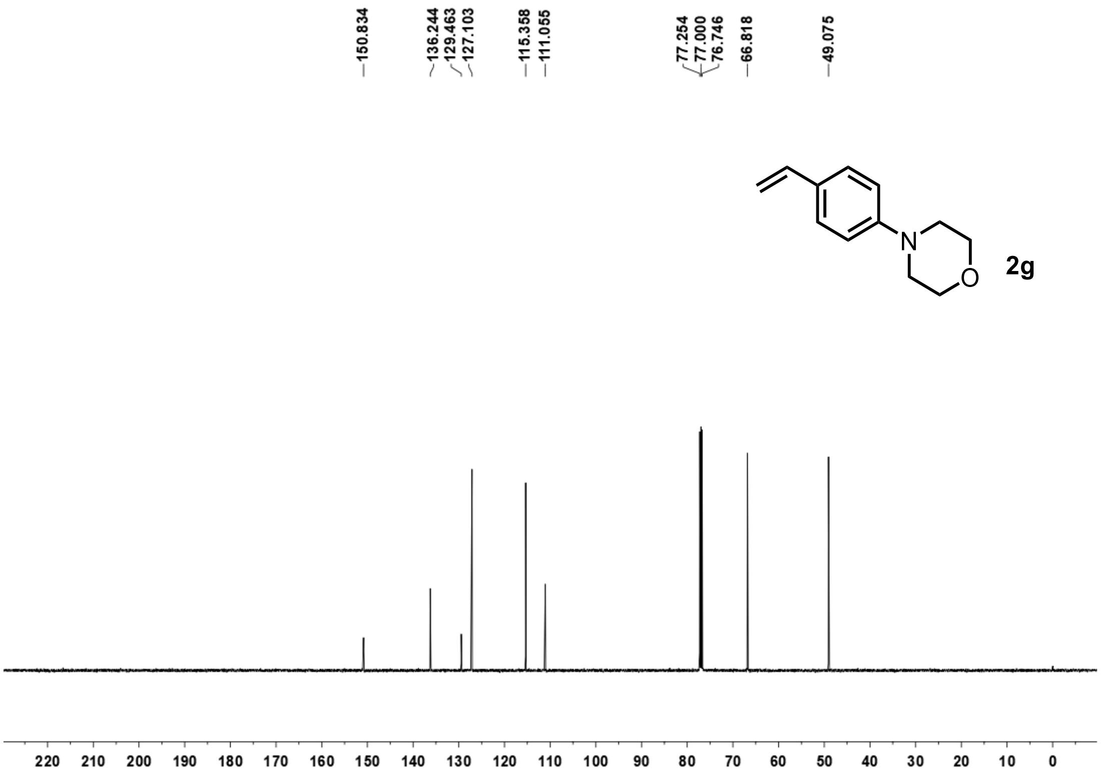

Supplementary Fig. 166 ^{13}C NMR (125 MHz, $CDCl_3$) of **2g**

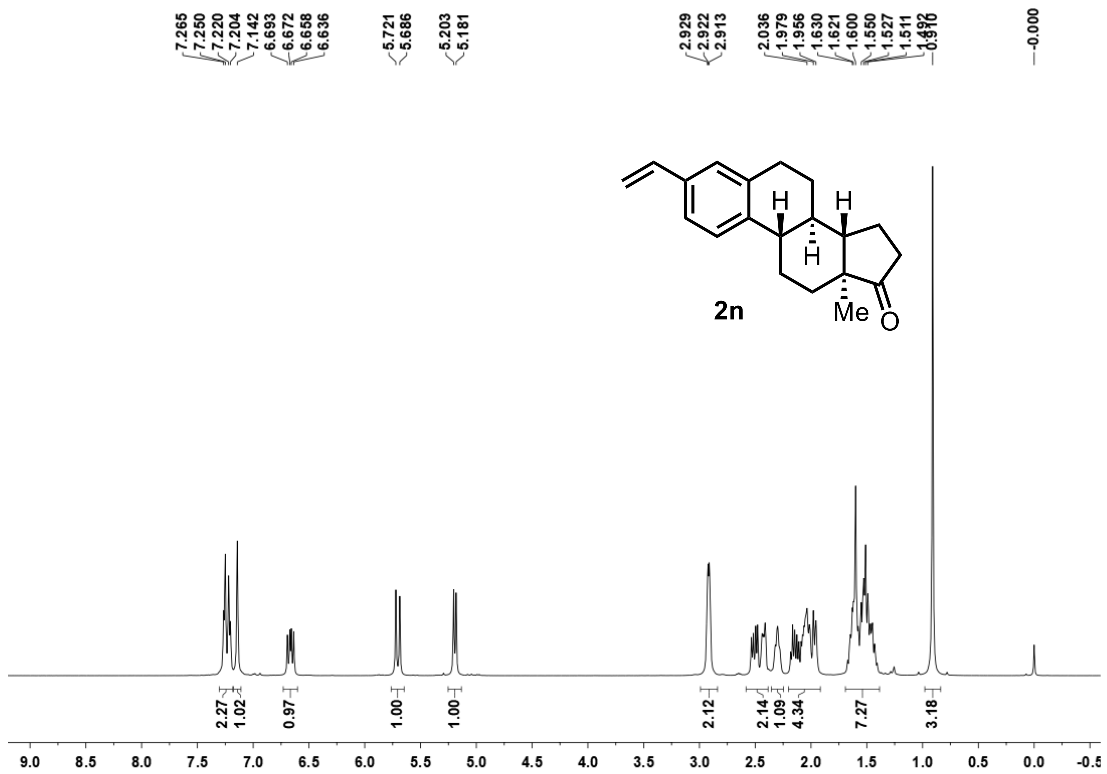

Supplementary Fig. 167 ^{1}H NMR (500 MHz, $CDCl_3$) of **2n**

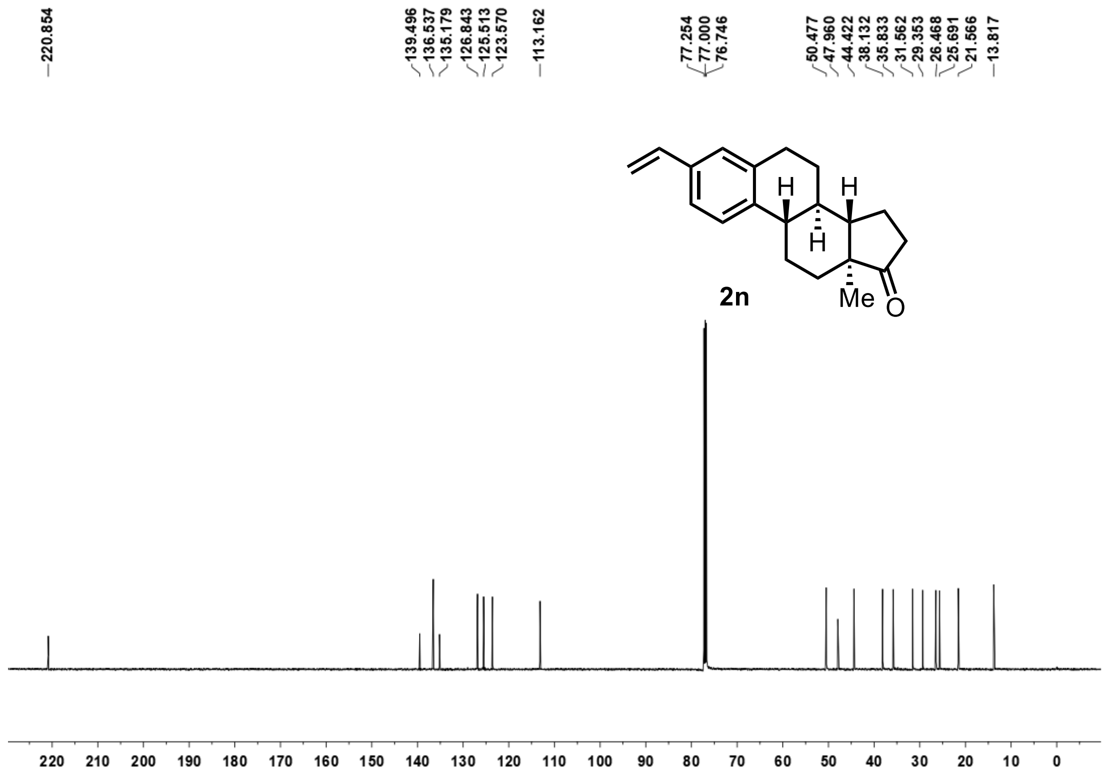

Supplementary Fig. 168 ^{13}C NMR (125 MHz, $CDCl_3$) of **2n**

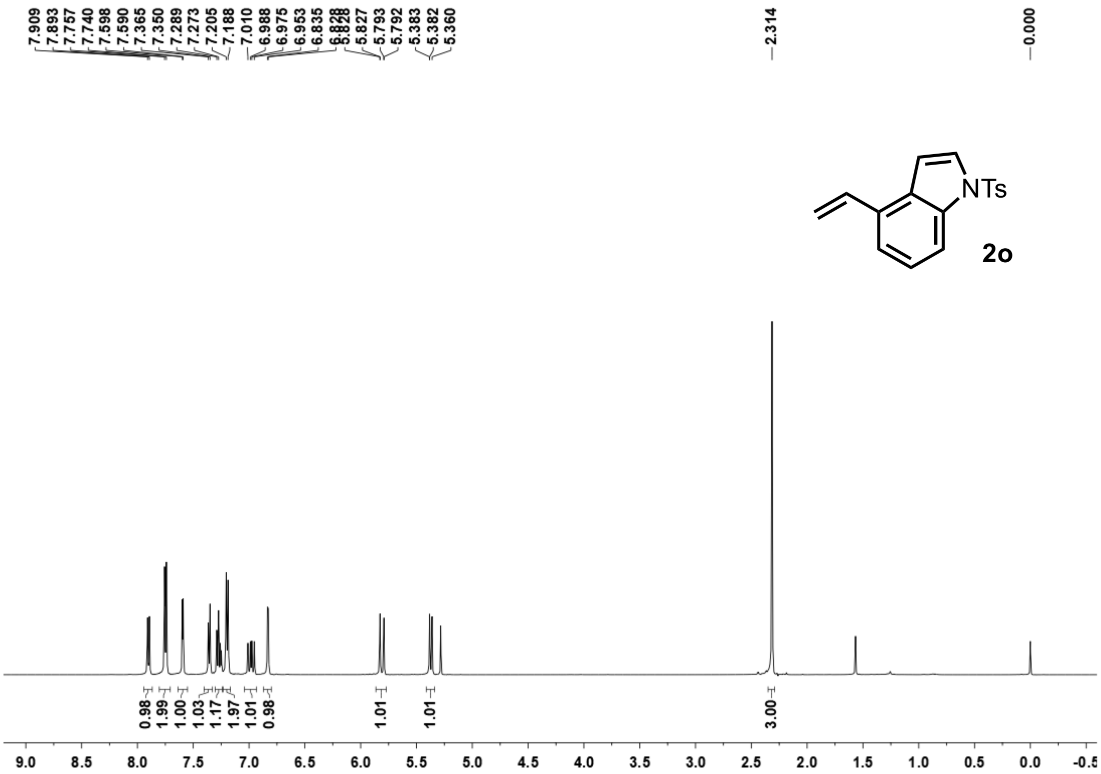

Supplementary Fig. 169 ^{1}H NMR (500 MHz, $CDCl_3$) of **2o**

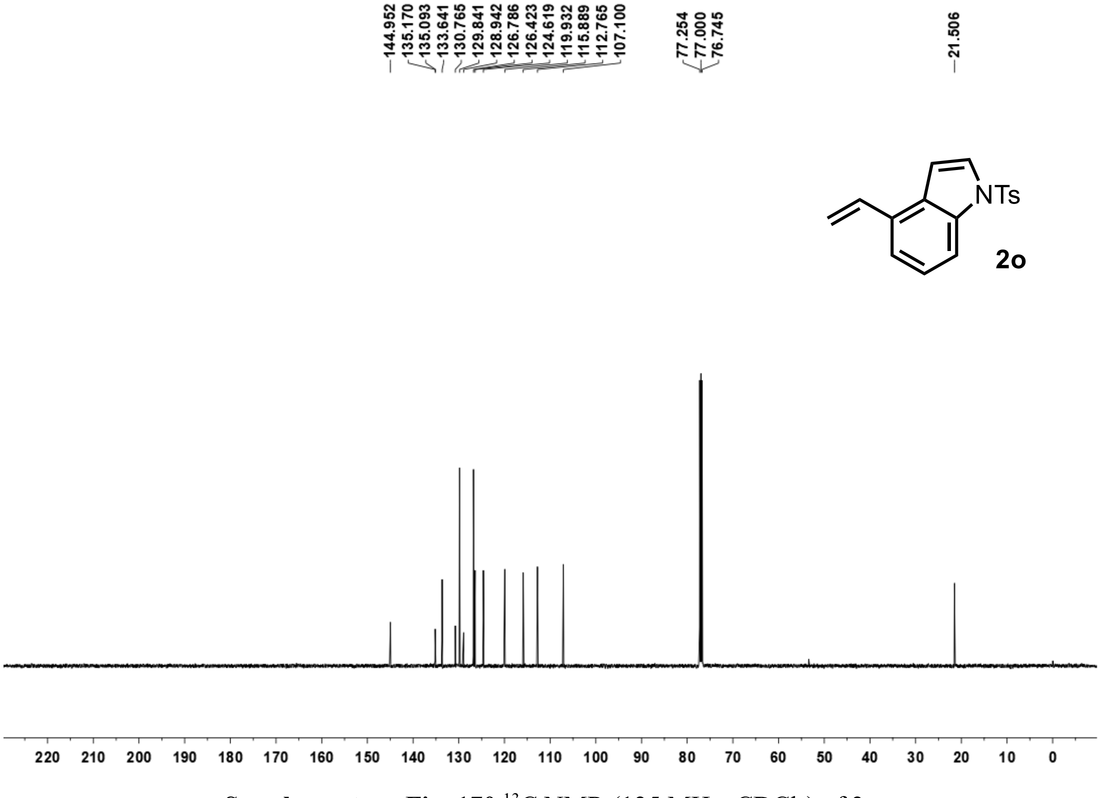

Supplementary Fig. 170 ^{13}C NMR (125 MHz, $CDCl_3$) of **2o**

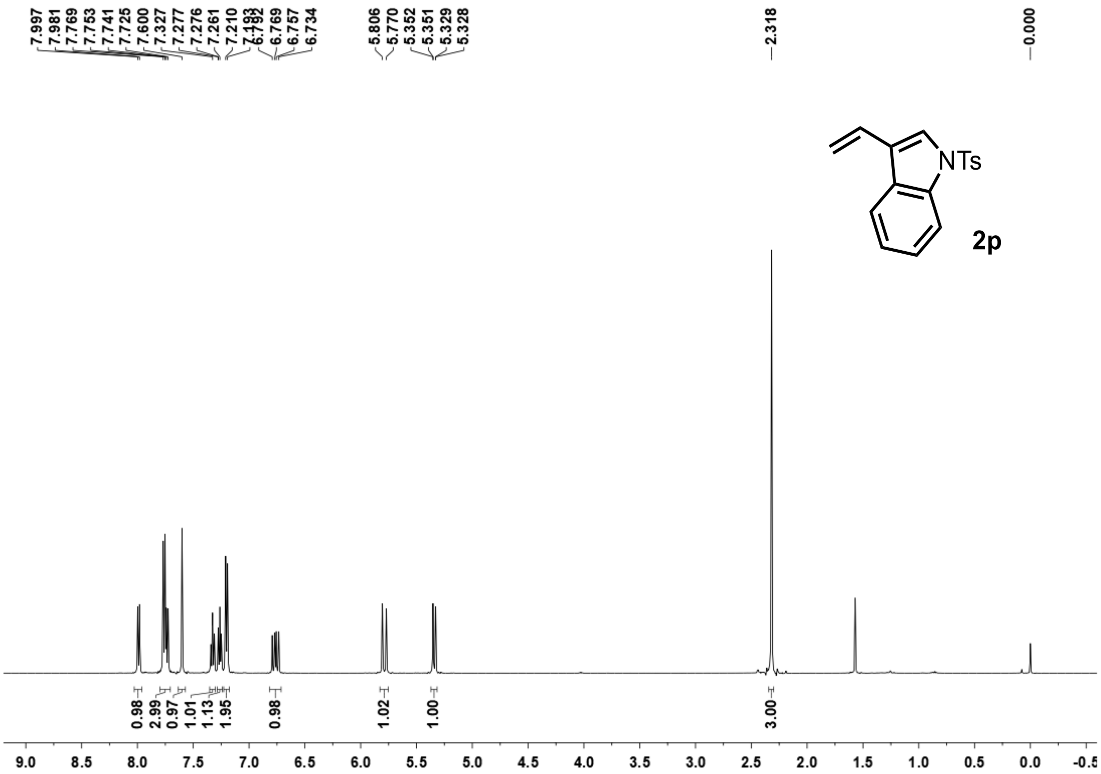

Supplementary Fig. 171 ^{1}H NMR (500 MHz, $CDCl_3$) of **2p**

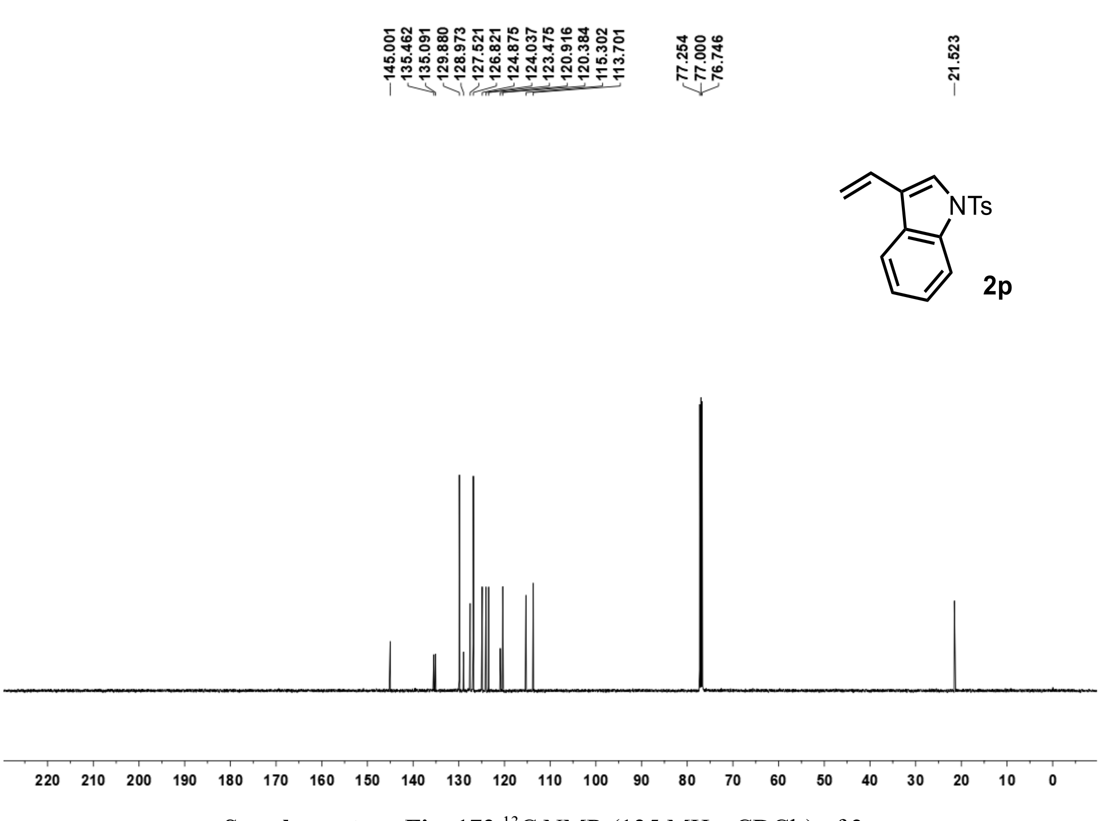

Supplementary Fig. 172 ^{13}C NMR (125 MHz, $CDCl_3$) of **2p**

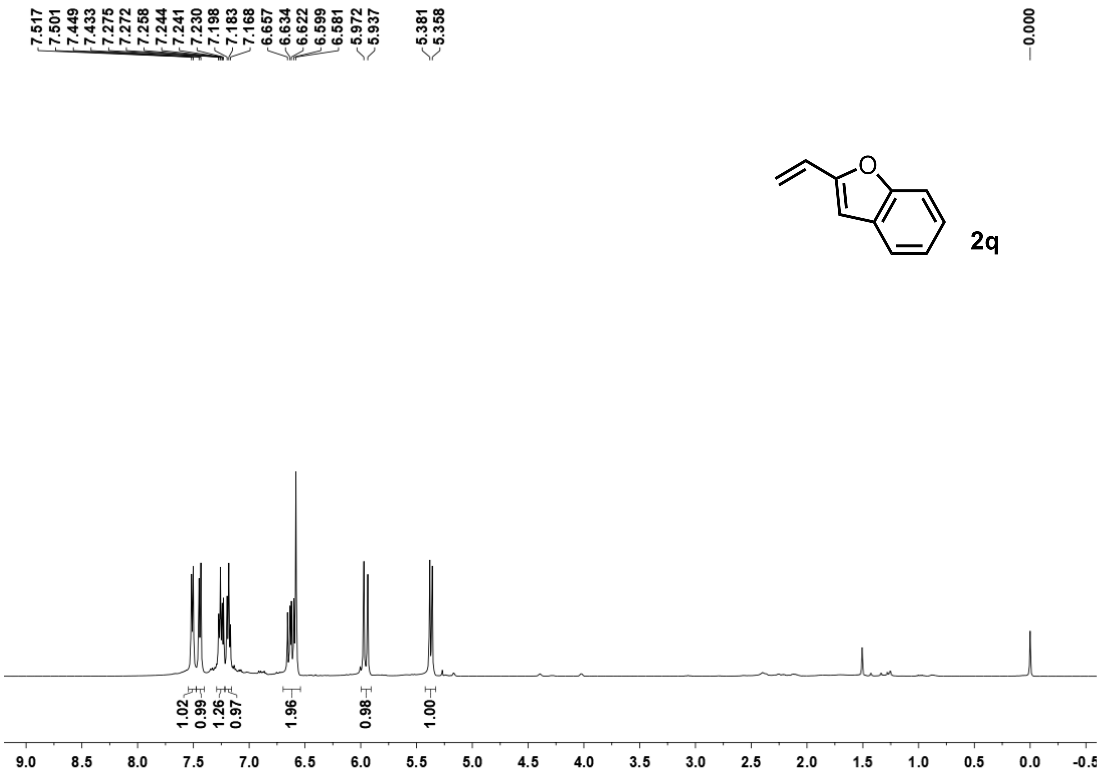

Supplementary Fig. 173 ^{1}H NMR (500 MHz, $CDCl_3$) of **2q**

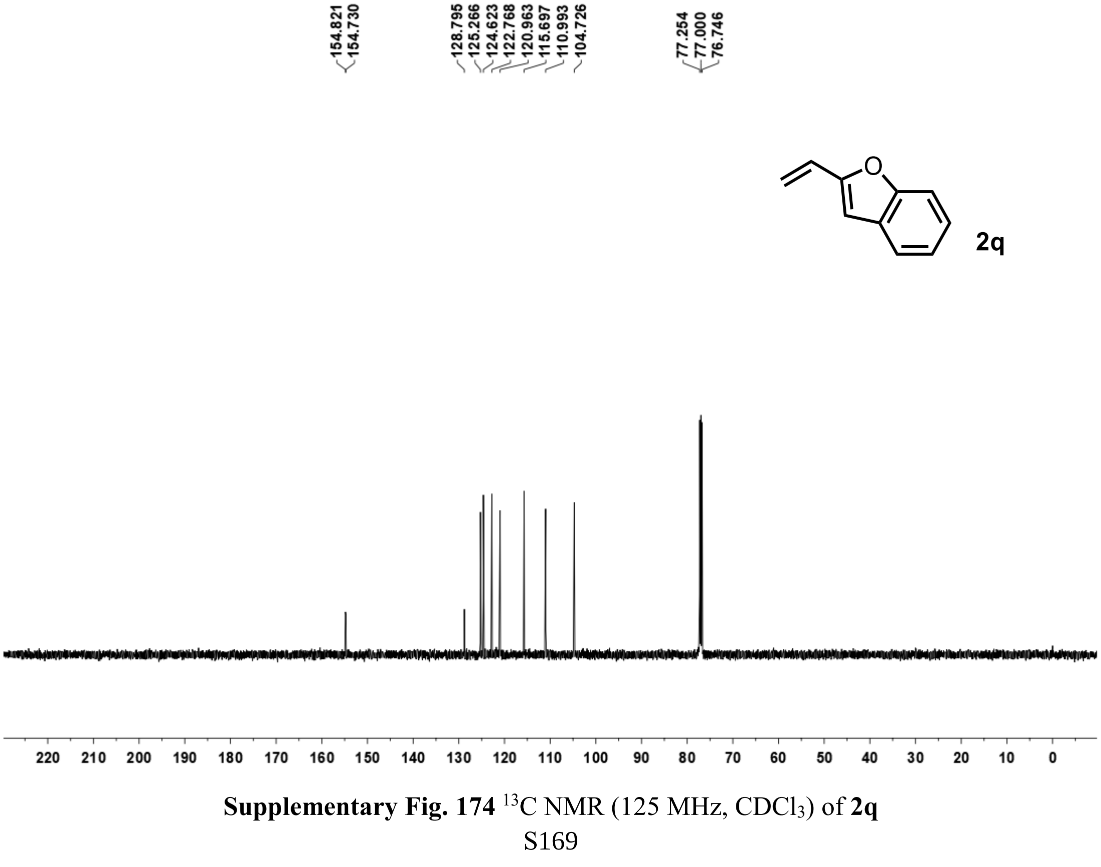

Supplementary Fig. 174 ^{13}C NMR (125 MHz, $CDCl_3$) of **2q**

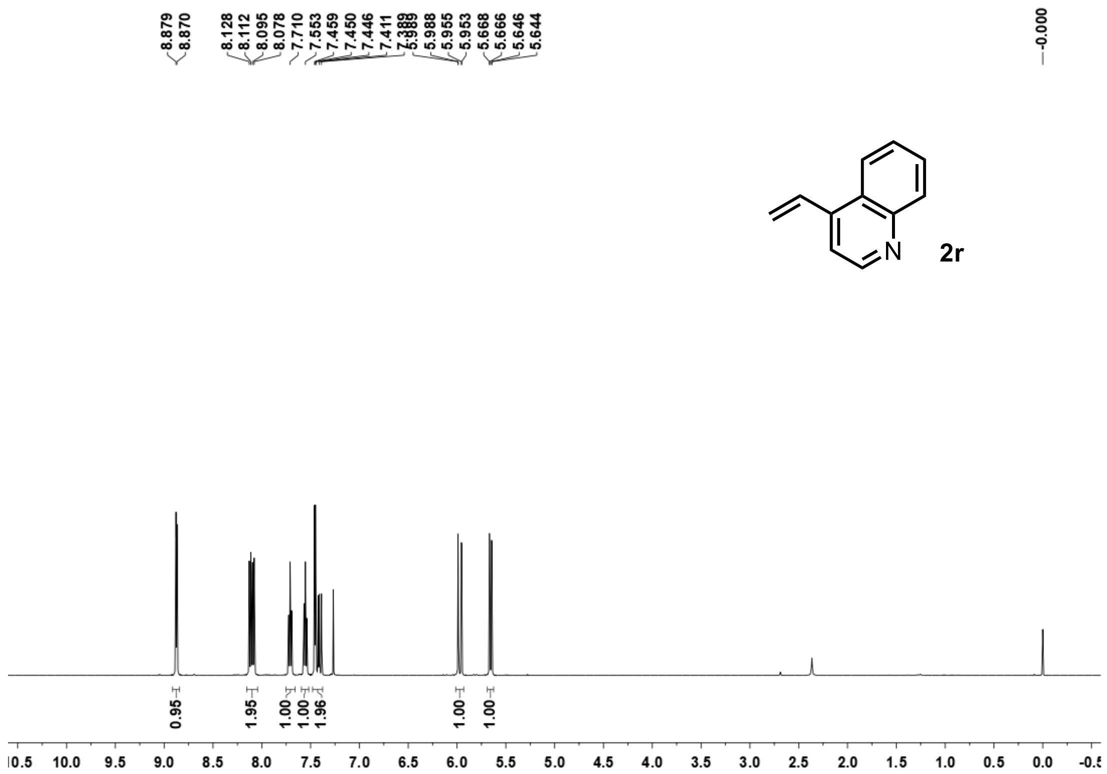

Supplementary Fig. 175 ^{1}H NMR (500 MHz, $CDCl_3$) of **2r**

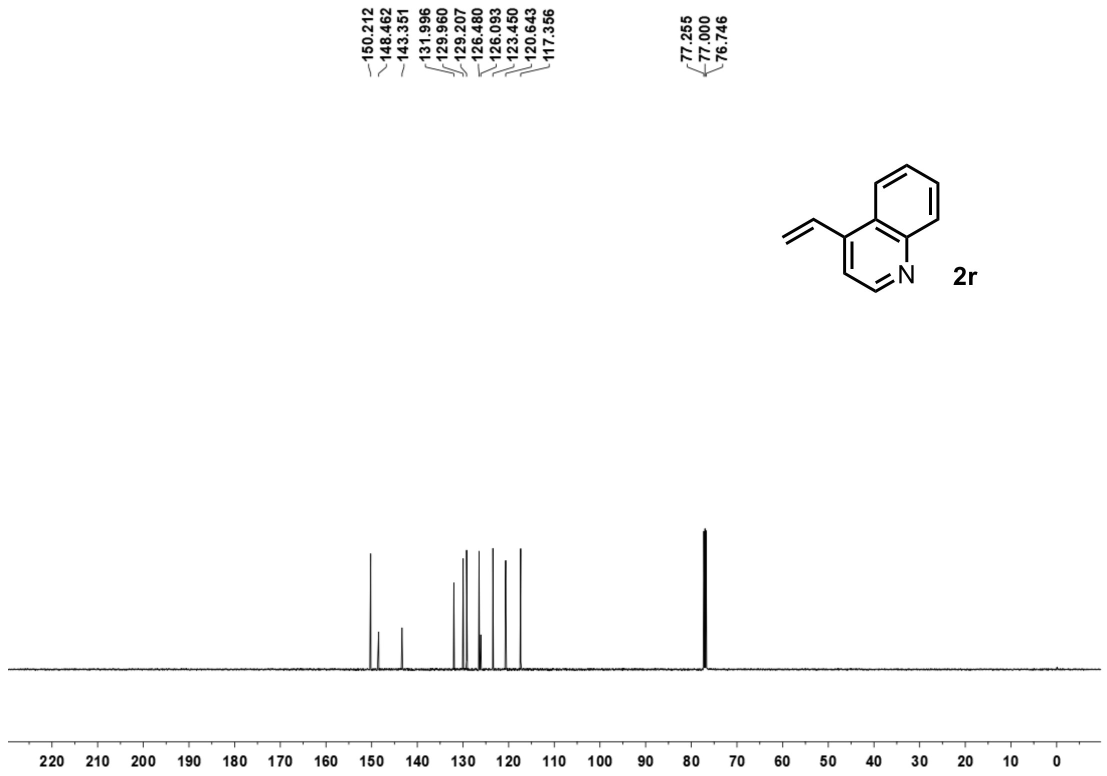

Supplementary Fig. 176 ^{13}C NMR (125 MHz, $CDCl_3$) of **2r**

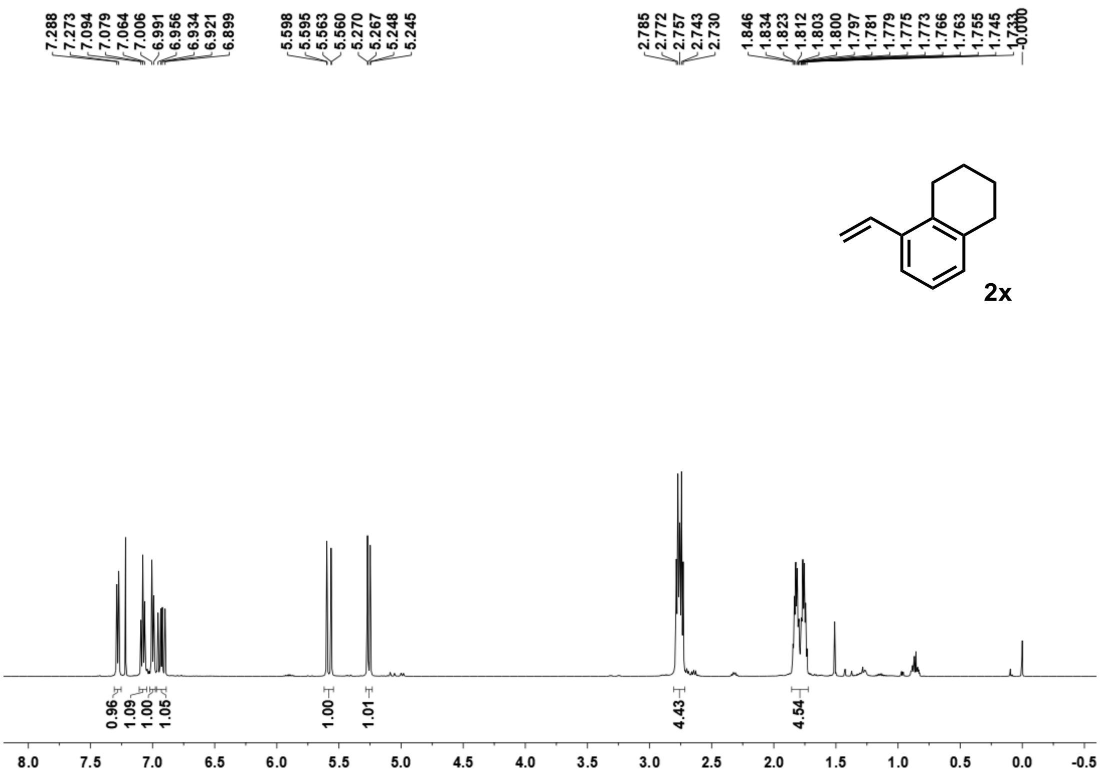

Supplementary Fig. 177 ^{1}H NMR (500 MHz, $CDCl_3$) of **2x**

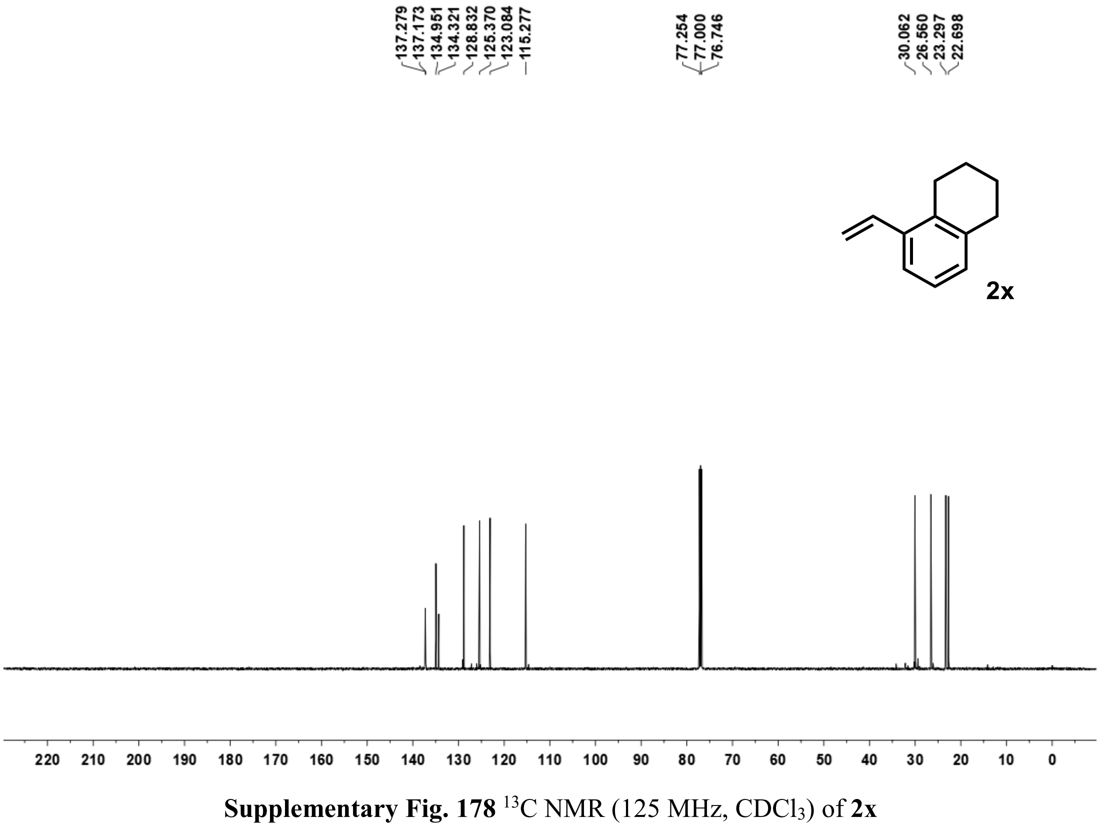

Supplementary Fig. 178 ^{13}C NMR (125 MHz, $CDCl_3$) of **2x**

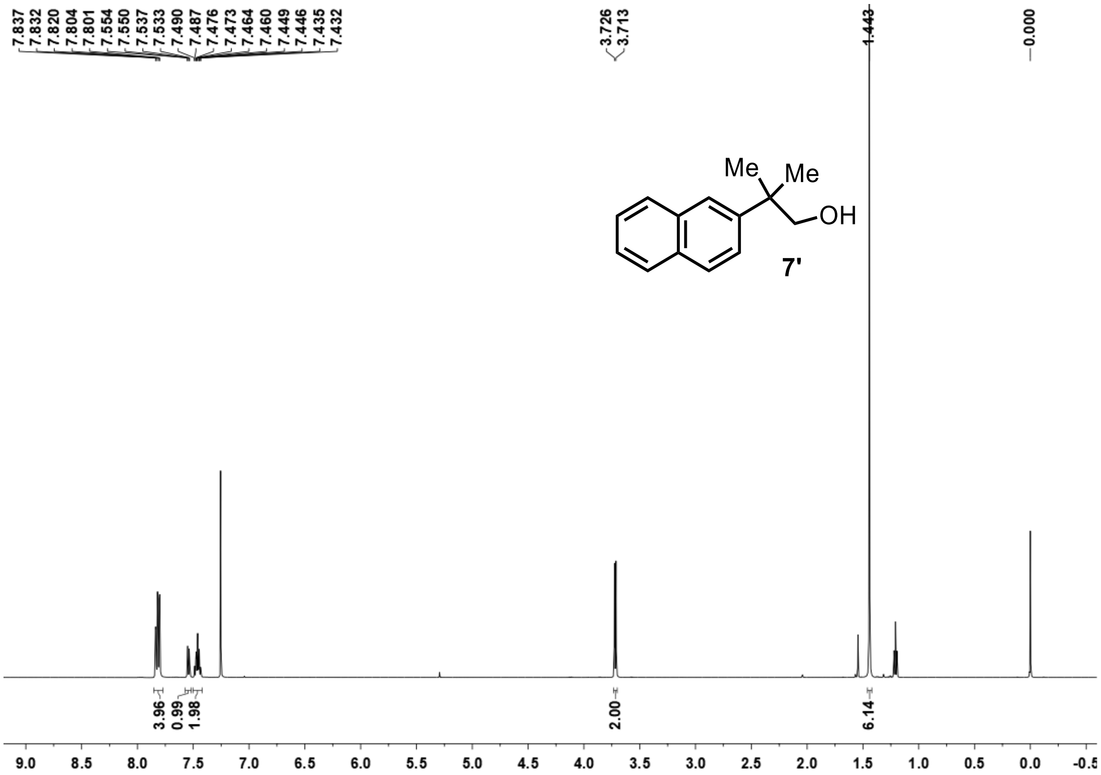

Supplementary Fig. 179 ^{1}H NMR (500 MHz, $CDCl_3$) of **7'**

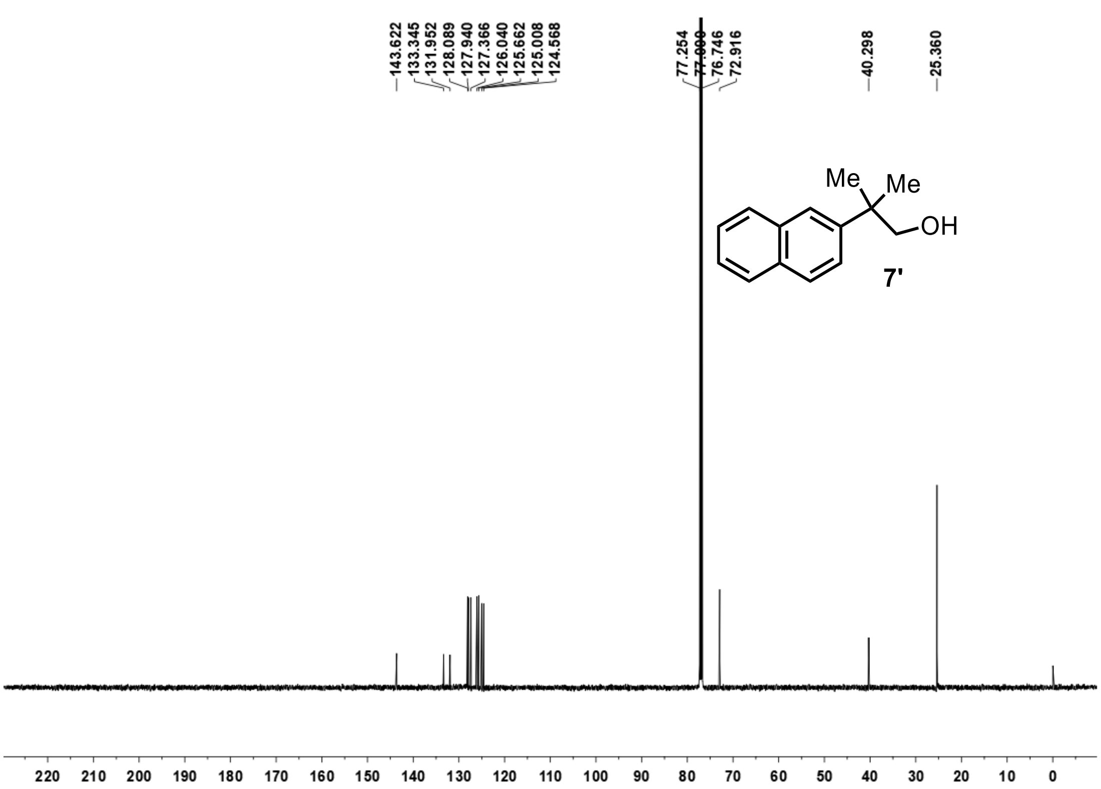

Supplementary Fig. 180 ^{13}C NMR (125 MHz, $CDCl_3$) of **7'**

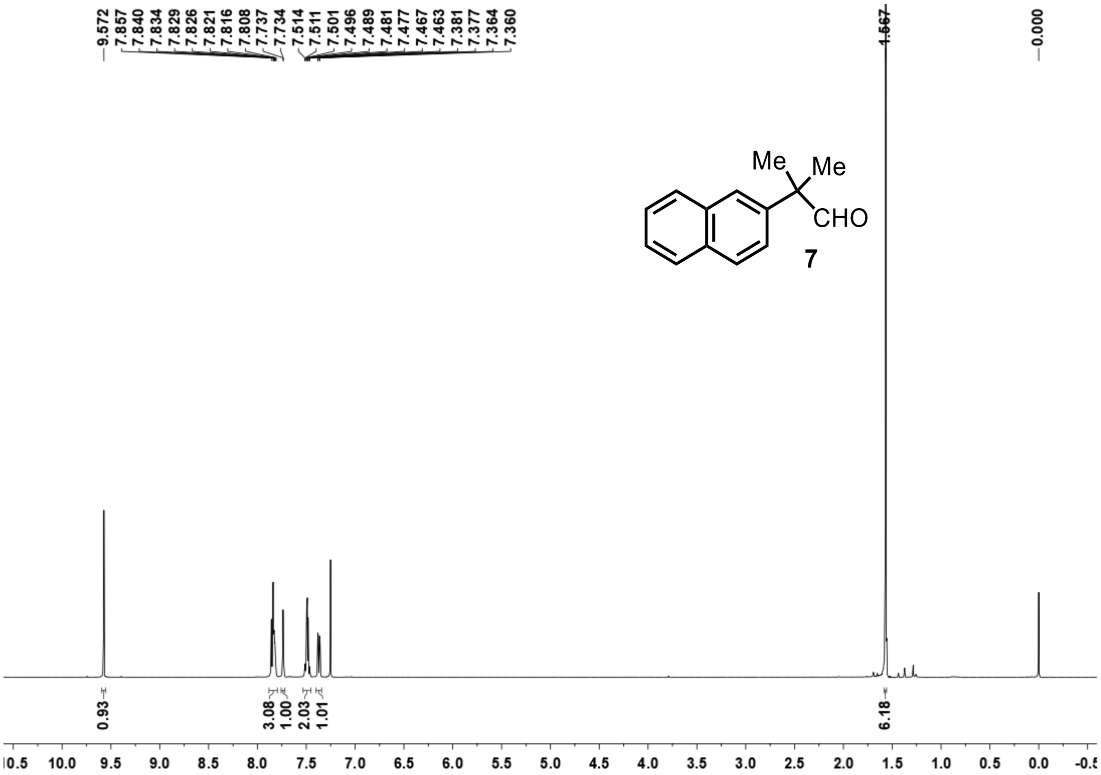

Supplementary Fig. 181 ^{1}H NMR (500 MHz, $CDCl_3$) of **7**

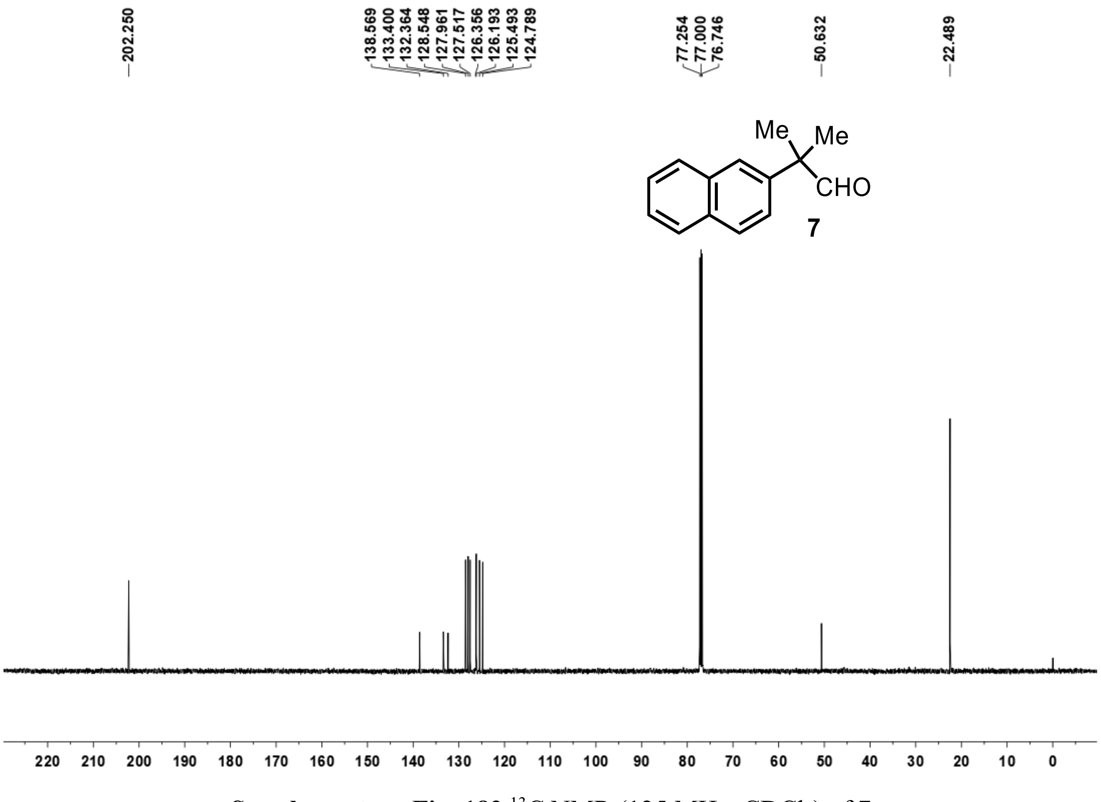

Supplementary Fig. 182 ^{13}C NMR (125 MHz, $CDCl_3$) of **7**

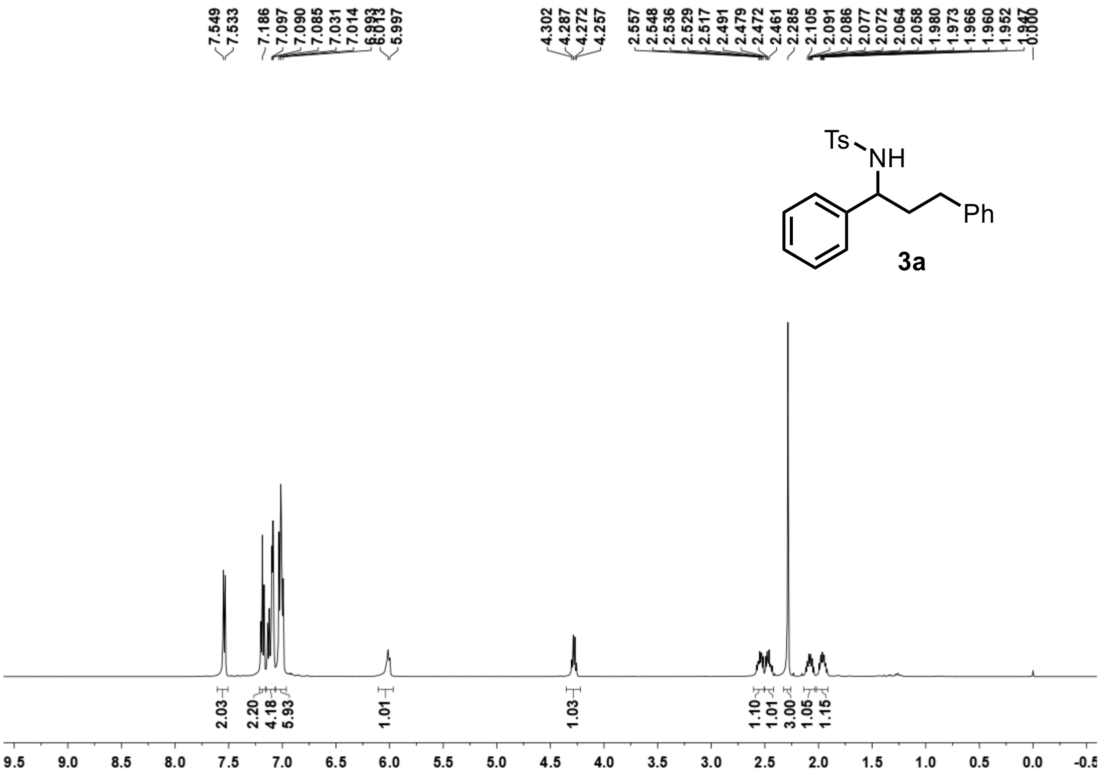

Supplementary Fig. 183 ^{1}H NMR (500 MHz, $CDCl_3$) of **3a**

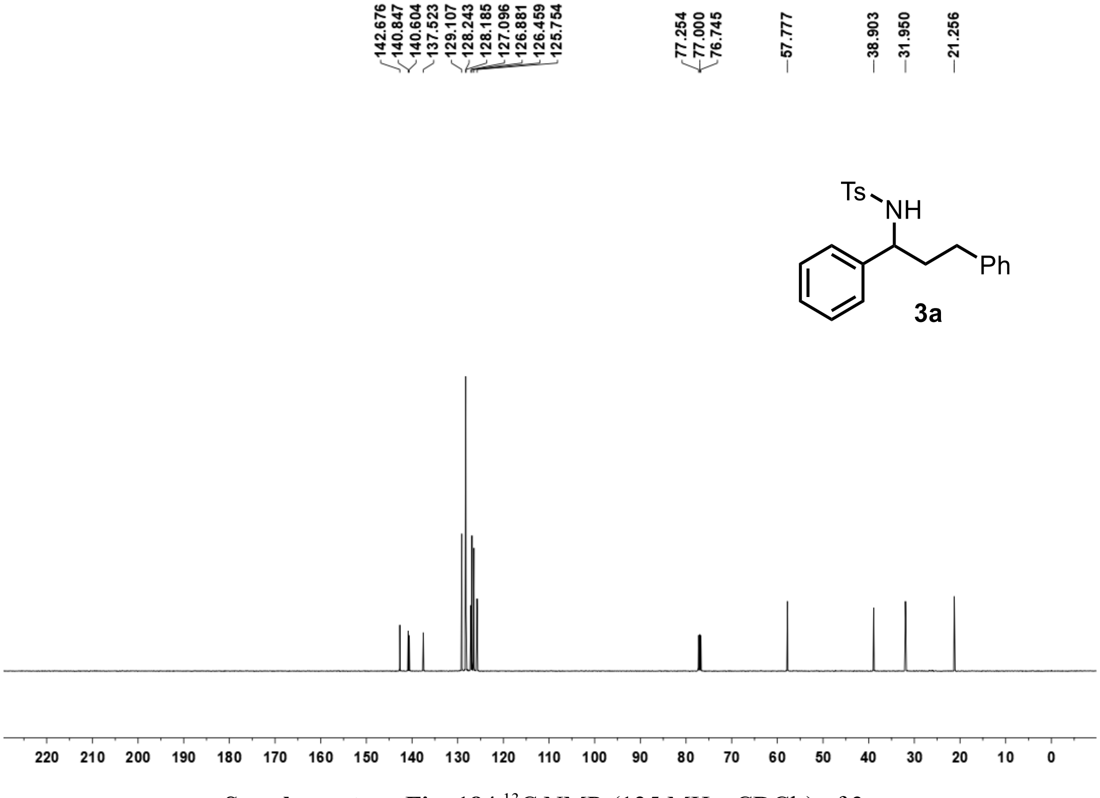

Supplementary Fig. 184 ^{13}C NMR (125 MHz, $CDCl_3$) of **3a**

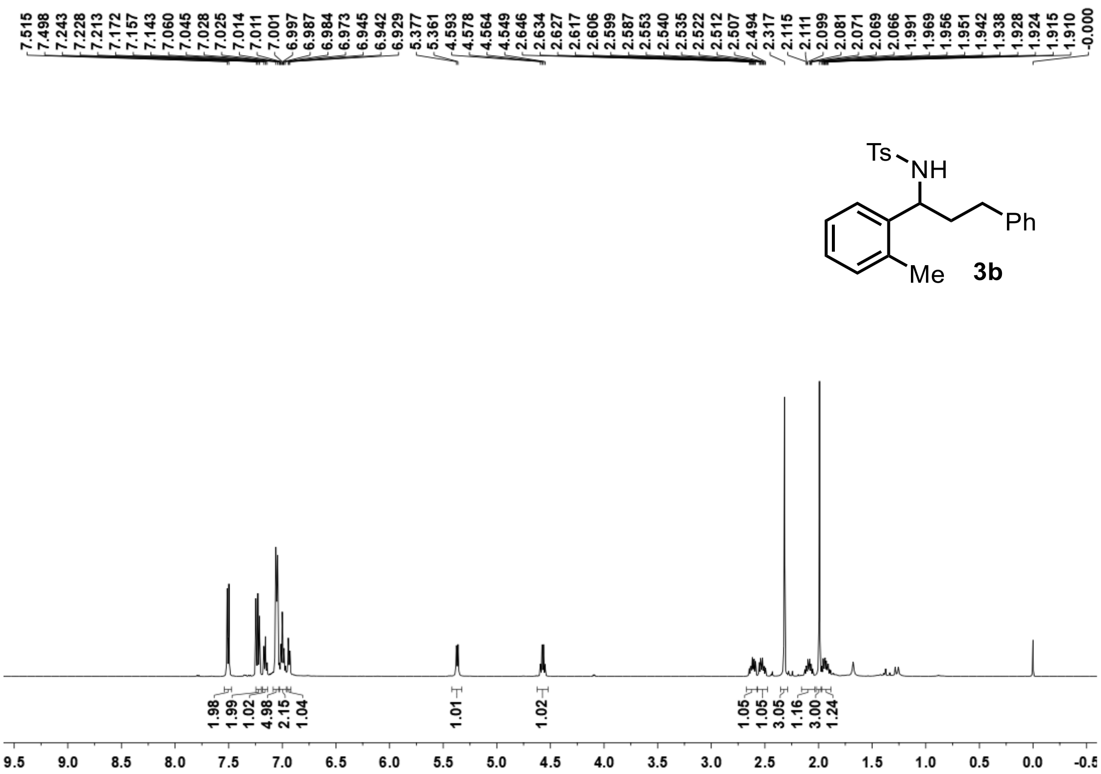

Supplementary Fig. 185 ^{1}H NMR (500 MHz, $CDCl_3$): of **3b**

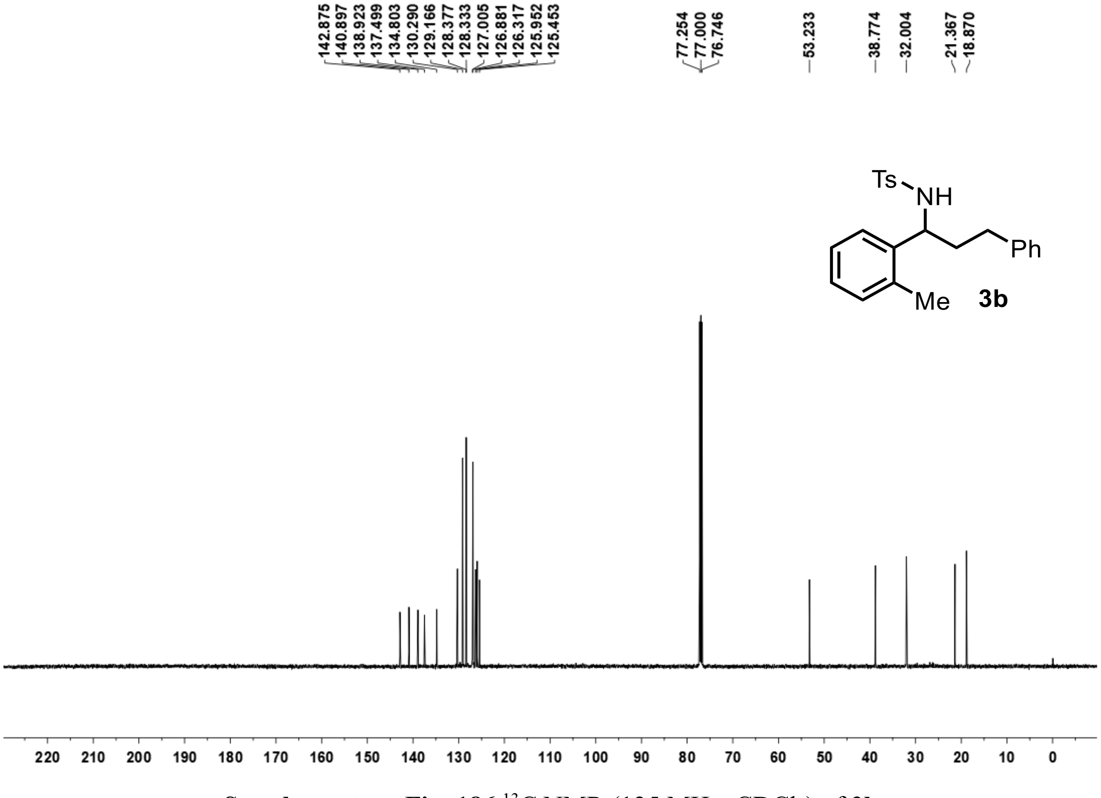

Supplementary Fig. 186 ^{13}C NMR (125 MHz, $CDCl_3$) of **3b**

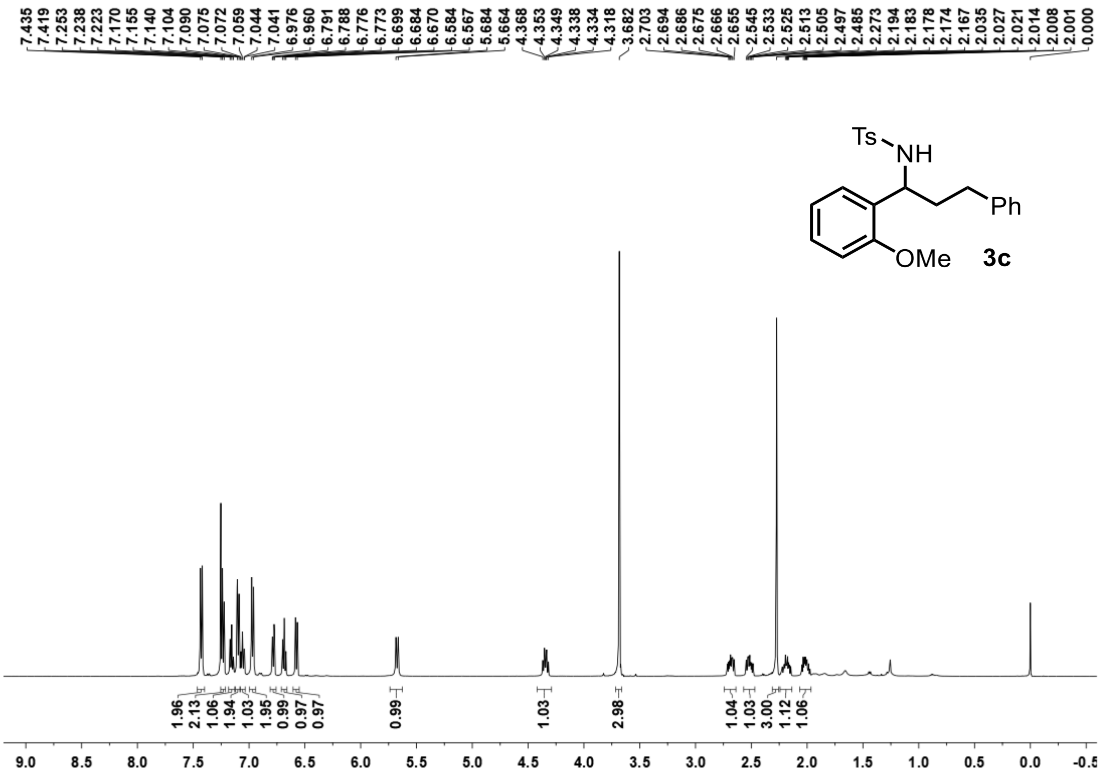

Supplementary Fig. 187 ^{1}H NMR (500 MHz, $CDCl_3$) of **3c**

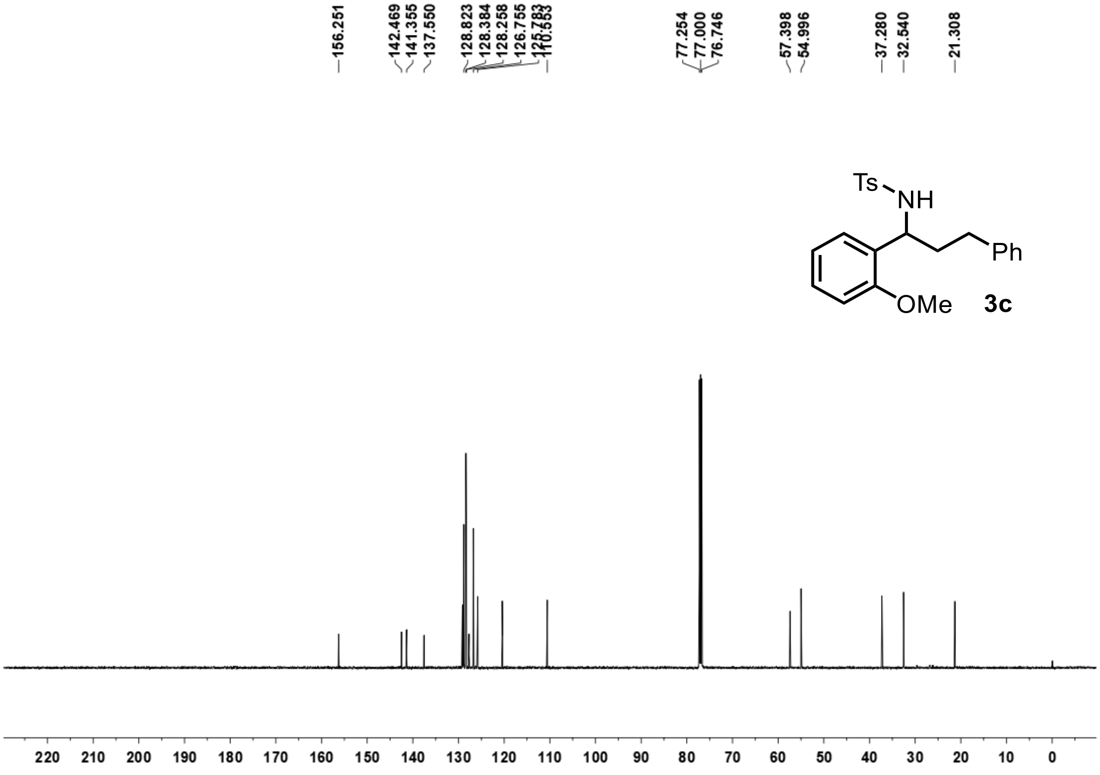

Supplementary Fig. 188 ^{13}C NMR (125 MHz, $CDCl_3$) of **3c**

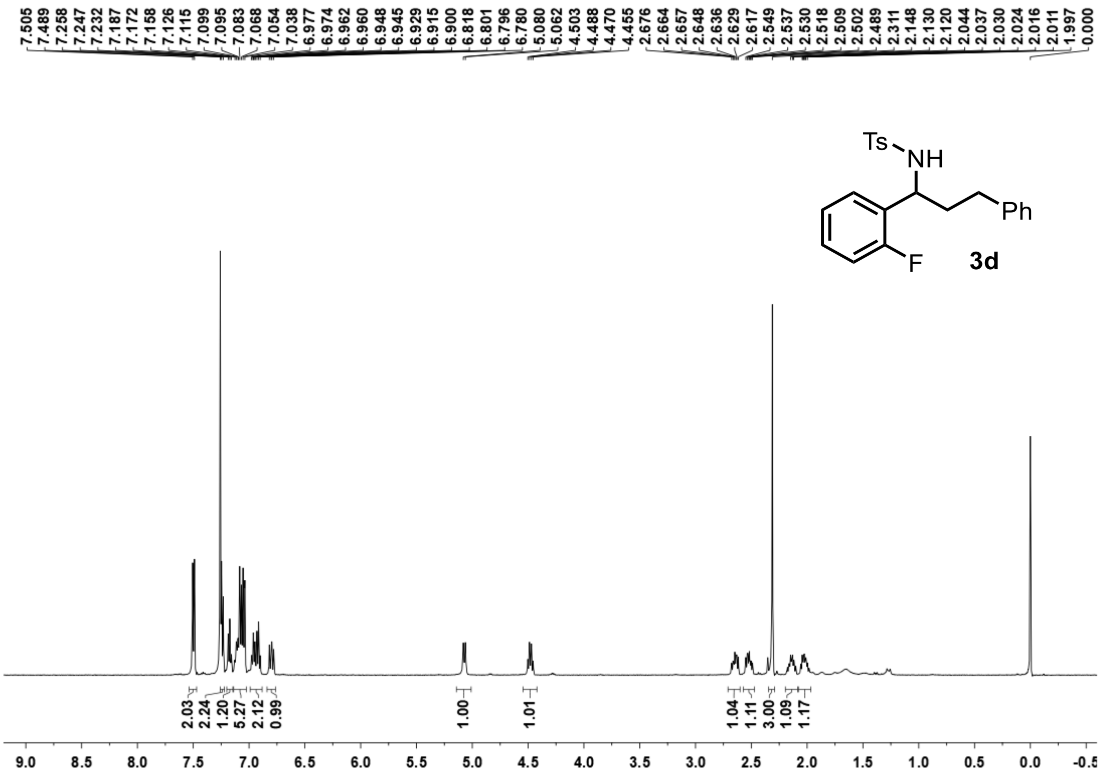

Supplementary Fig. 189 ^{1}H NMR (500 MHz, $CDCl_3$) of **3d**

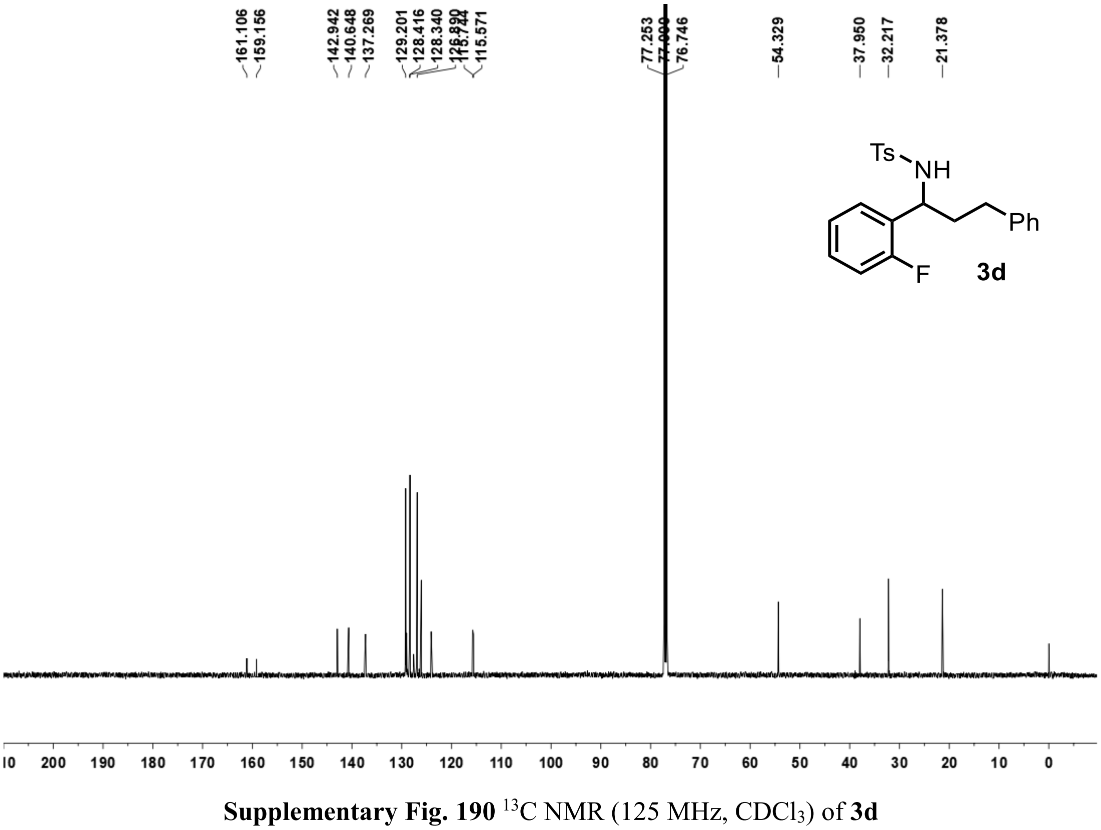

Supplementary Fig. 190 ^{13}C NMR (125 MHz, $CDCl_3$) of **3d**

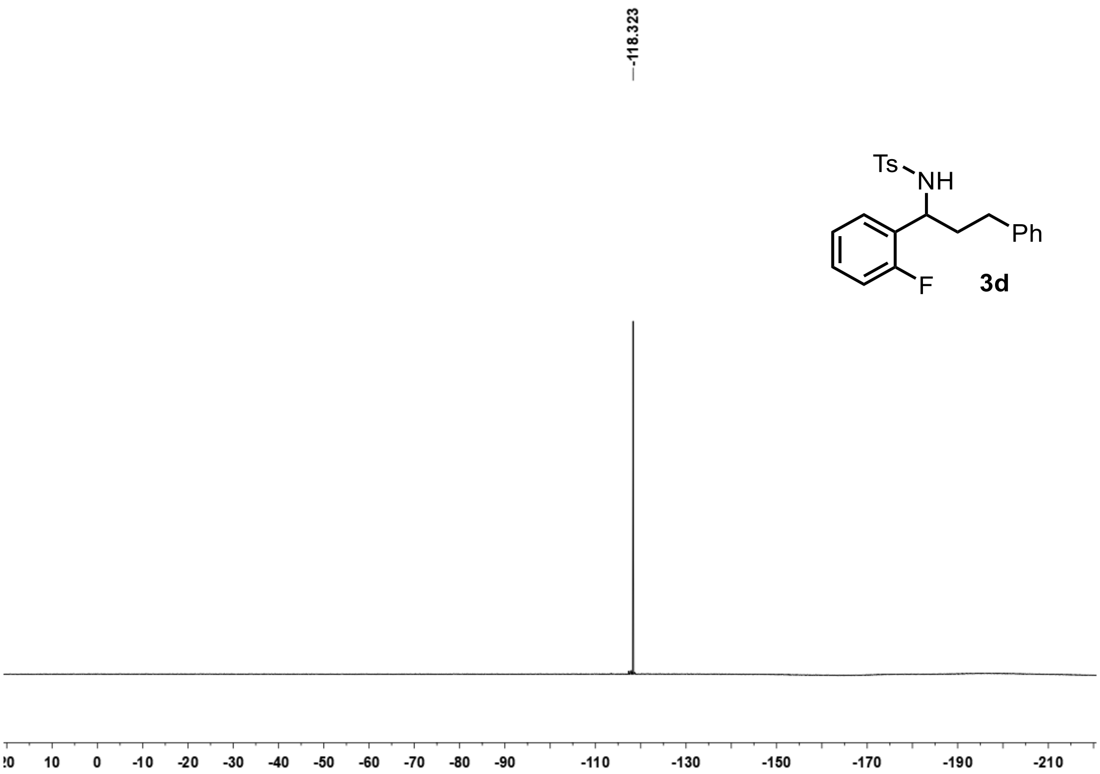

Supplementary Fig. 191 ^{19}F NMR (470 MHz, $CDCl_3$) of **3d**

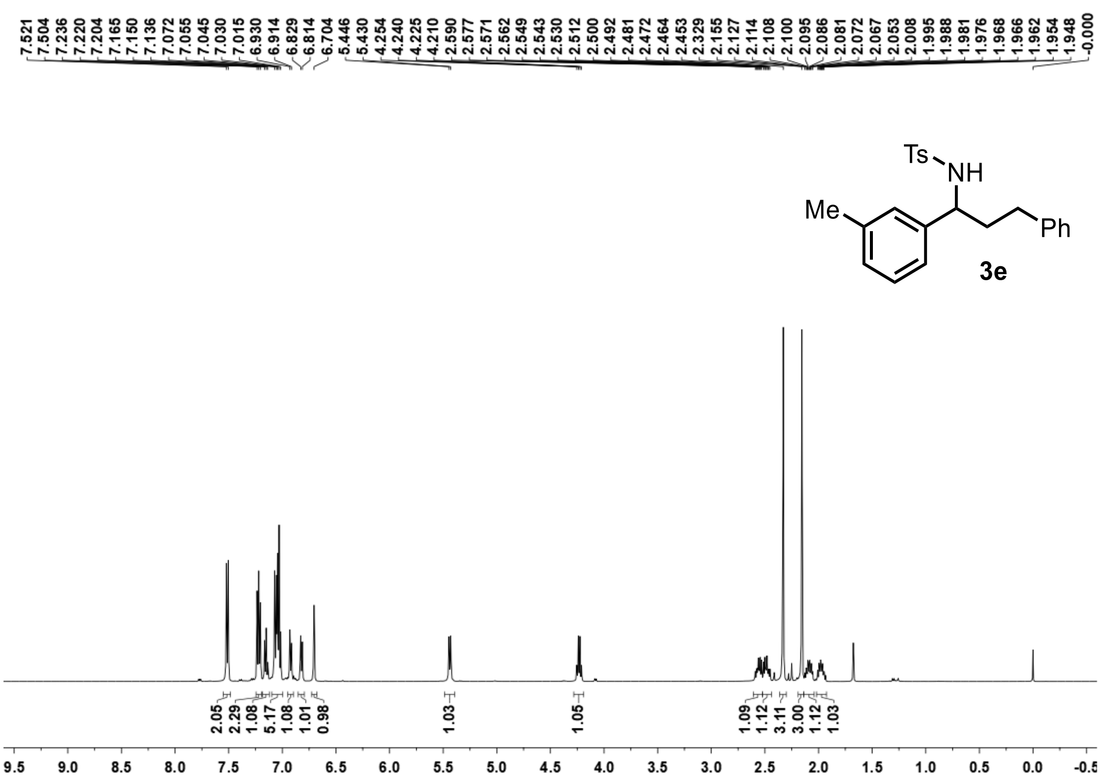

Supplementary Fig. 192 ^{1}H NMR (500 MHz, $CDCl_3$) of **3e**

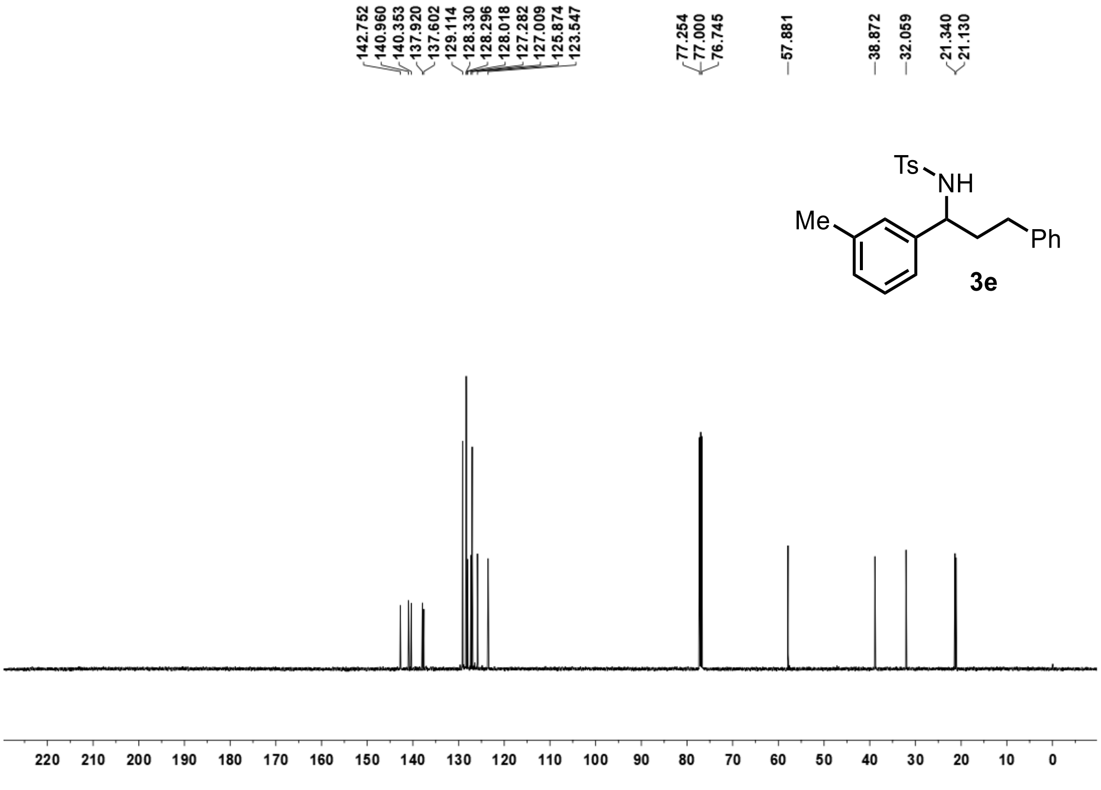

Supplementary Fig. 193 ^{13}C NMR (125 MHz, $CDCl_3$) of **3e**

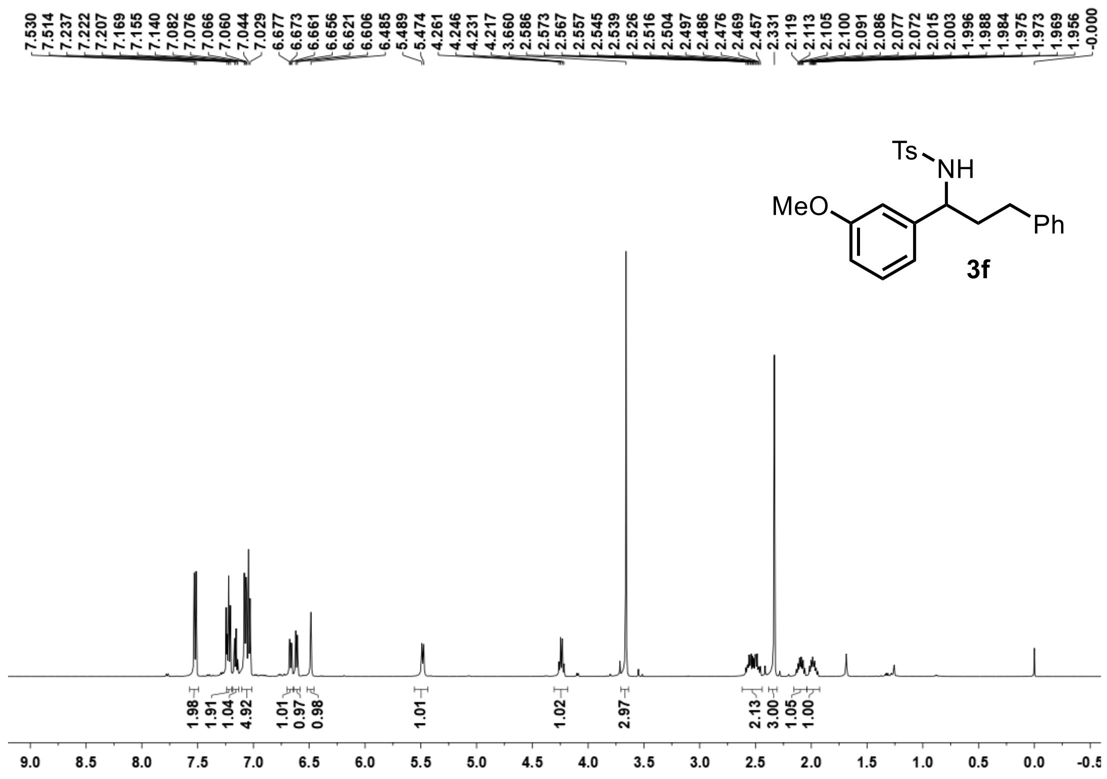

Supplementary Fig. 194 ^{1}H NMR (500 MHz, $CDCl_3$) of **3f**

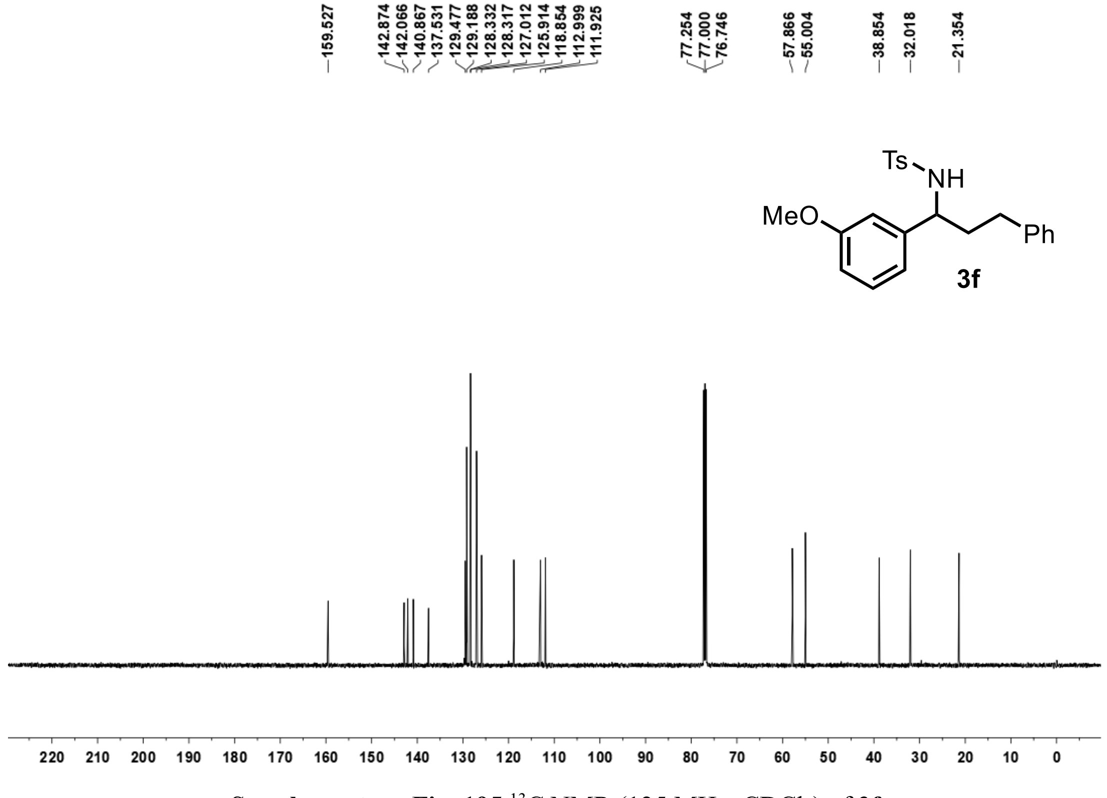

Supplementary Fig. 195 ^{13}C NMR (125 MHz, $CDCl_3$) of **3f**

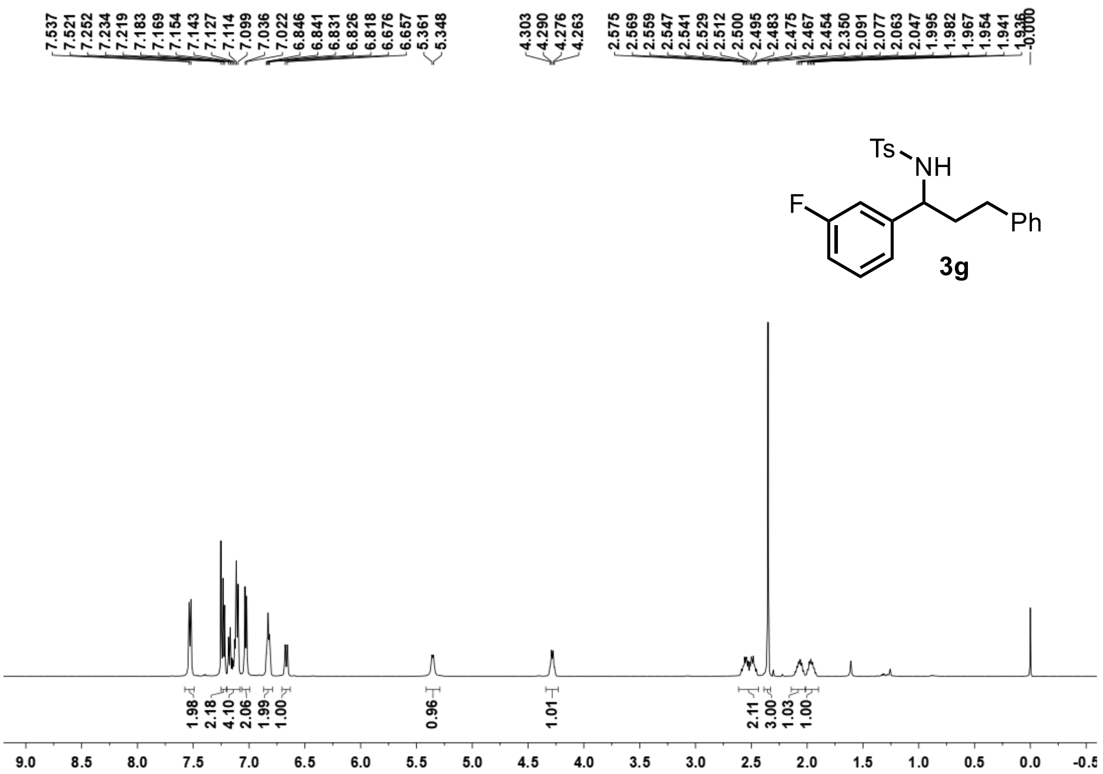

Supplementary Fig. 196 ^{1}H NMR (500 MHz, $CDCl_3$) of **3g**

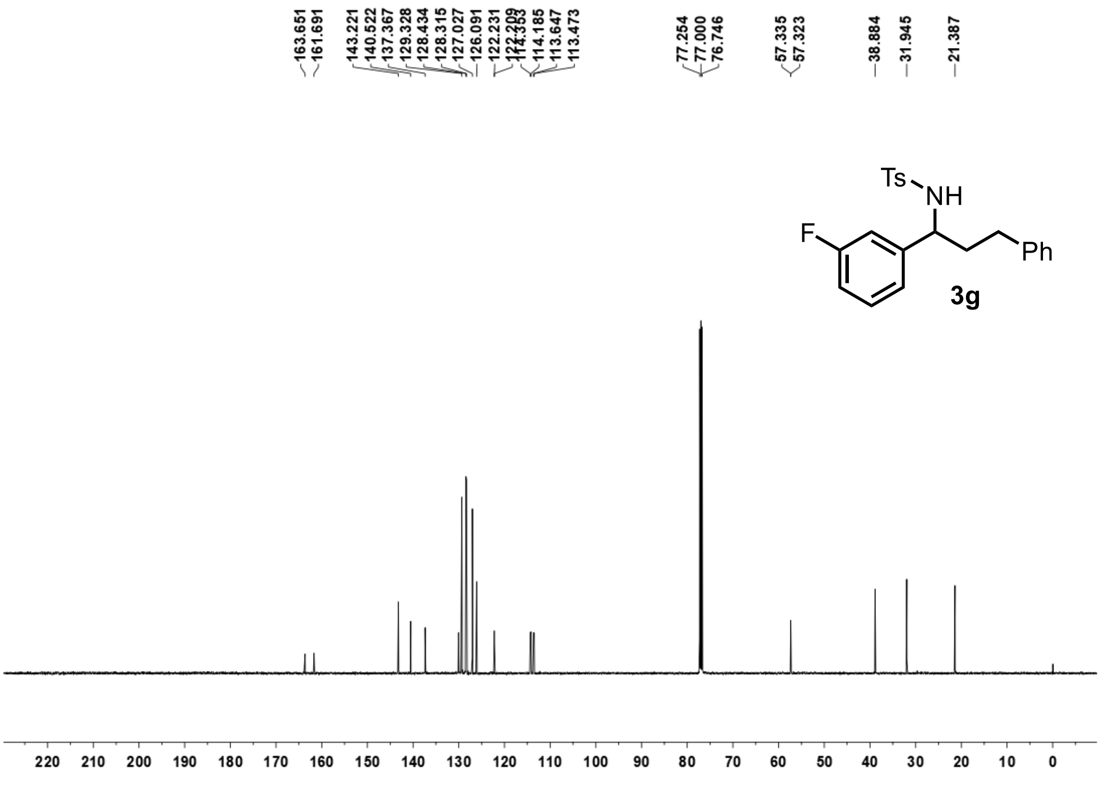

Supplementary Fig. 197 ^{13}C NMR (125 MHz, $CDCl_3$) of **3g**

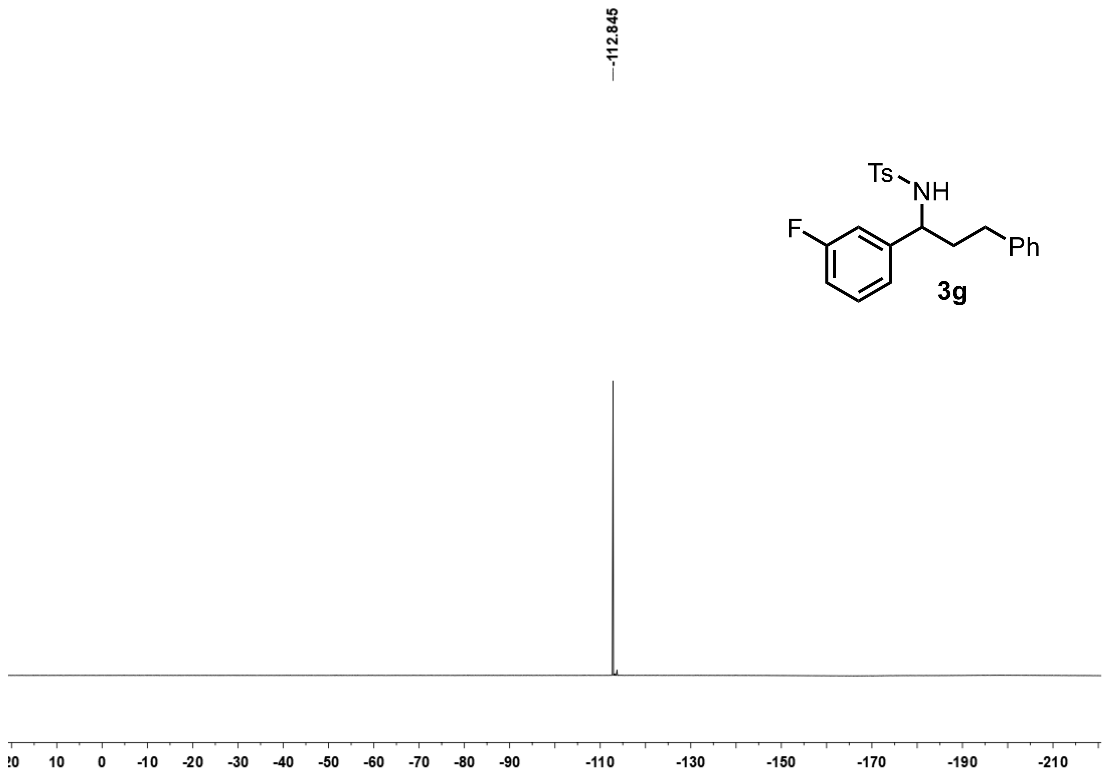

Supplementary Fig. 198 ^{19}F NMR (470 MHz, $CDCl_3$) of **3g**

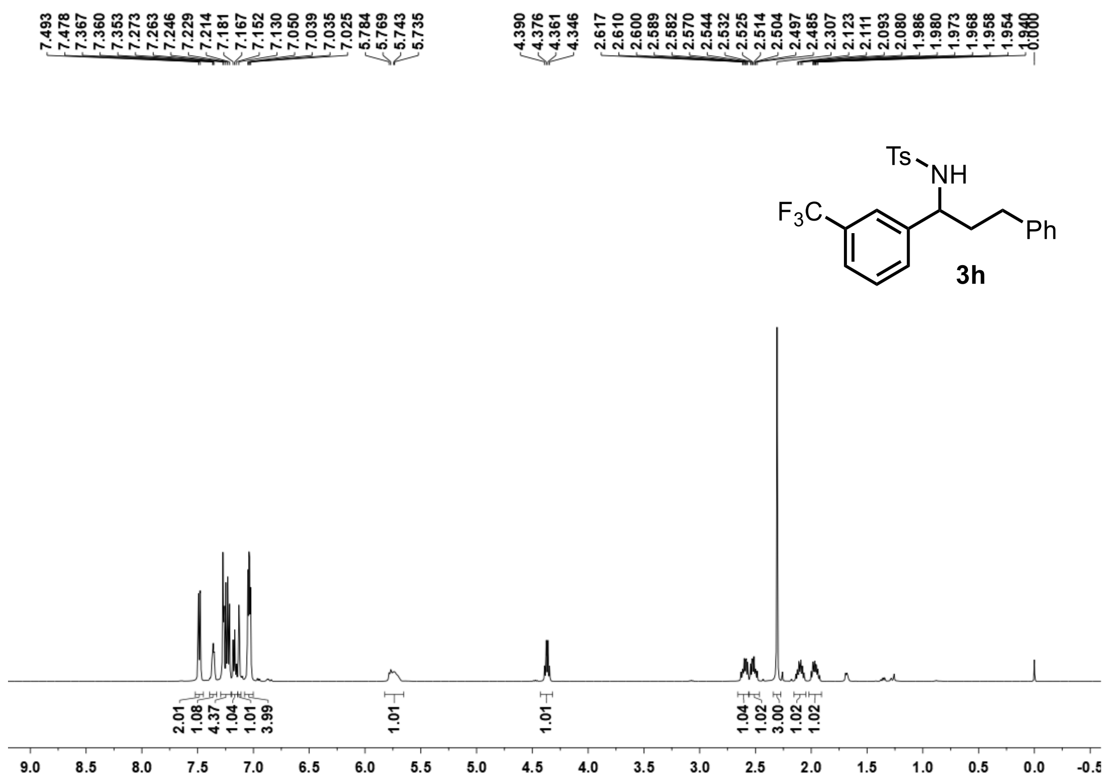

Supplementary Fig. 199 ^{1}H NMR (500 MHz, $CDCl_3$) of **3h**

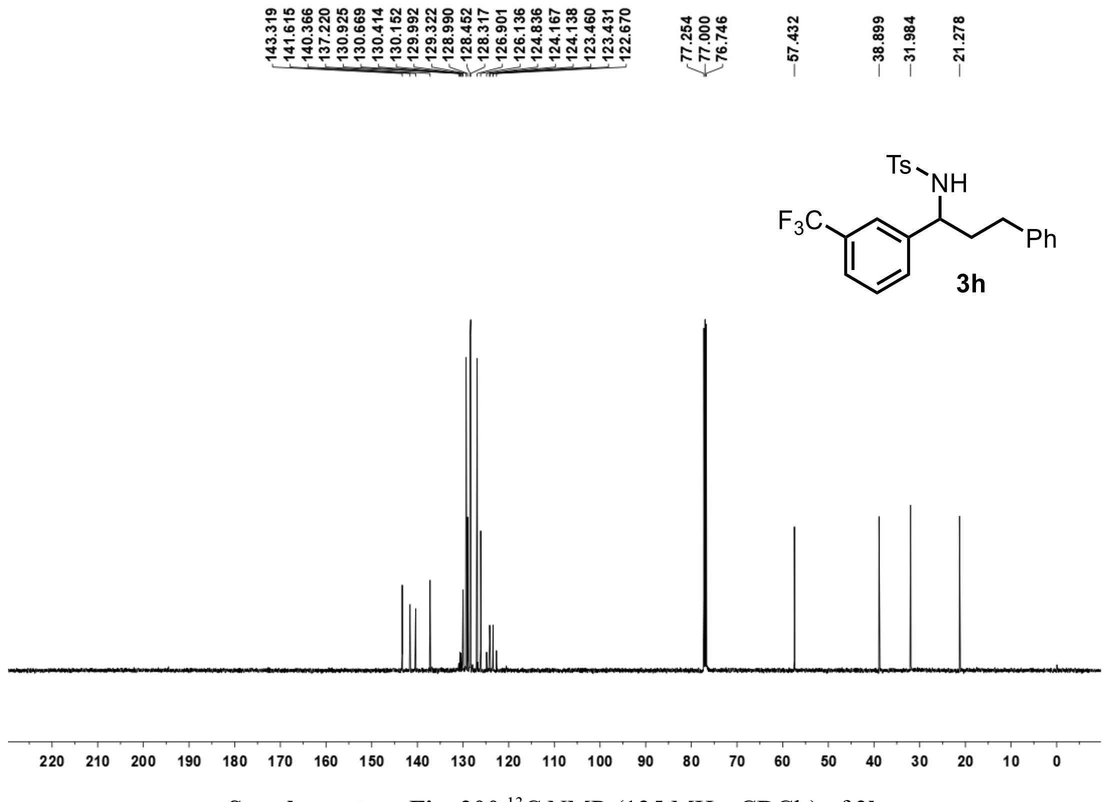

Supplementary Fig. 200 ^{13}C NMR (125 MHz, $CDCl_3$) of **3h**

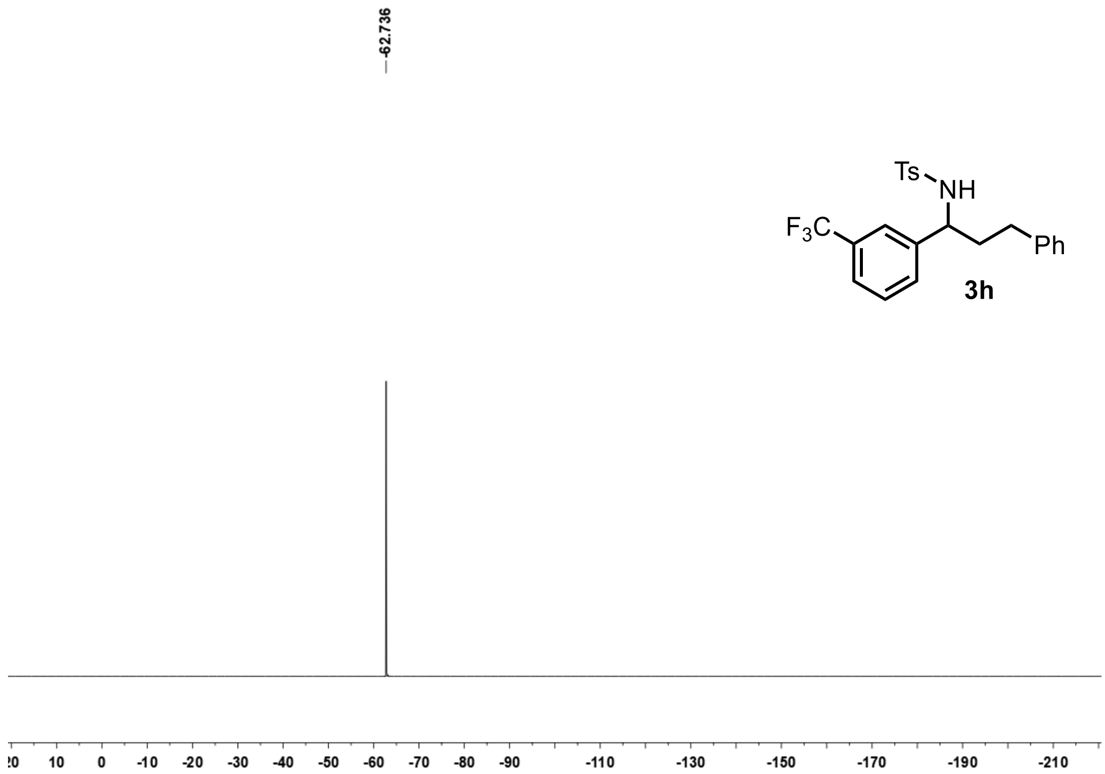

Supplementary Fig. 201 ^{19}F NMR (470 MHz, $CDCl_3$) of **3h**

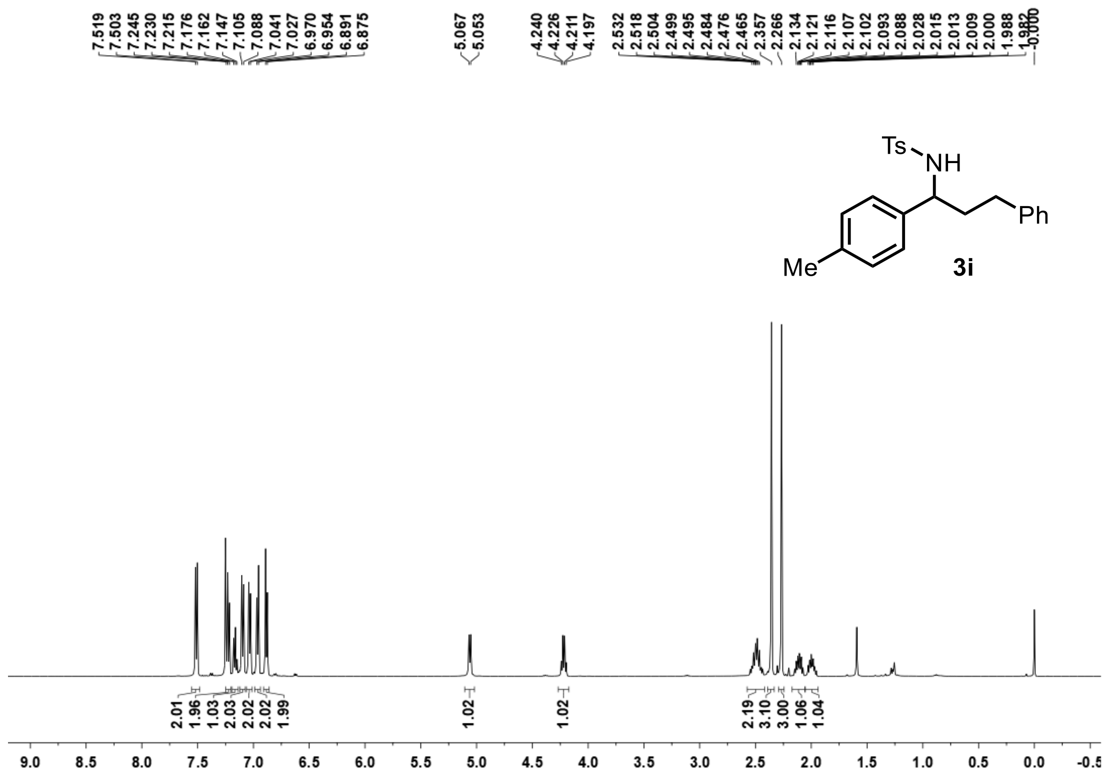

Supplementary Fig. 202 ^{1}H NMR (500 MHz, $CDCl_3$) of **3i**

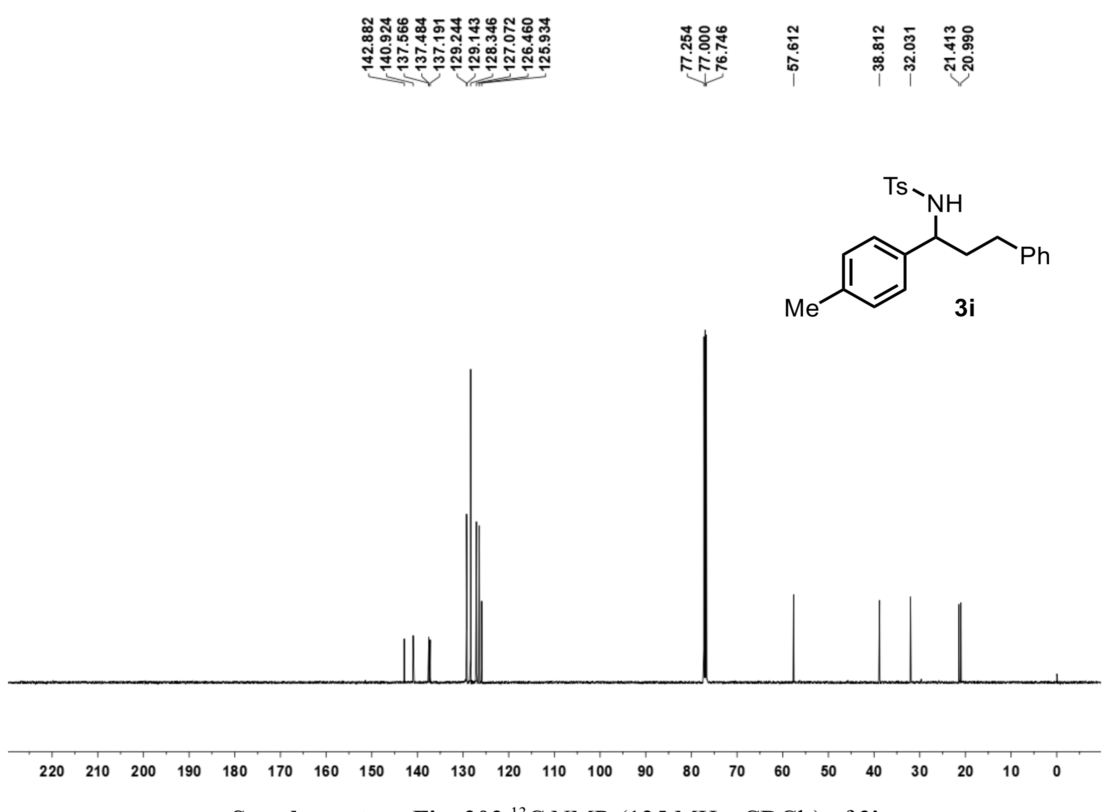

Supplementary Fig. 203 ^{13}C NMR (125 MHz, $CDCl_3$) of **3i**

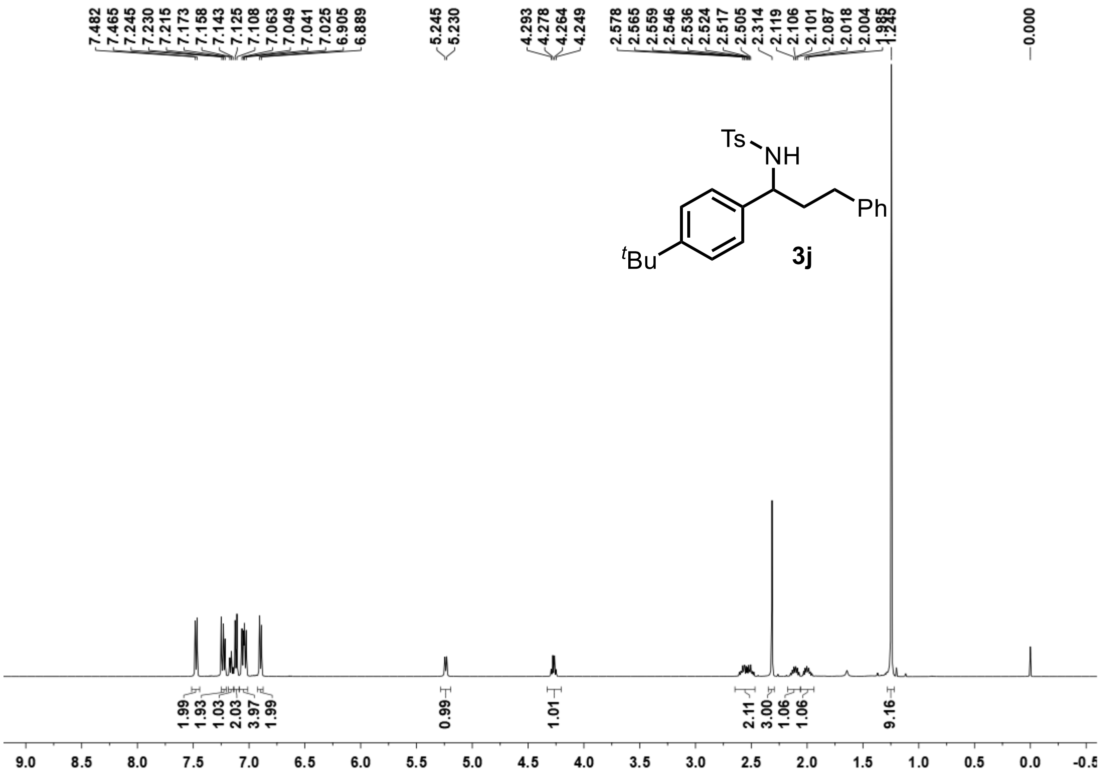

Supplementary Fig. 204 ^{1}H NMR (500 MHz, $CDCl_3$) of **3j**

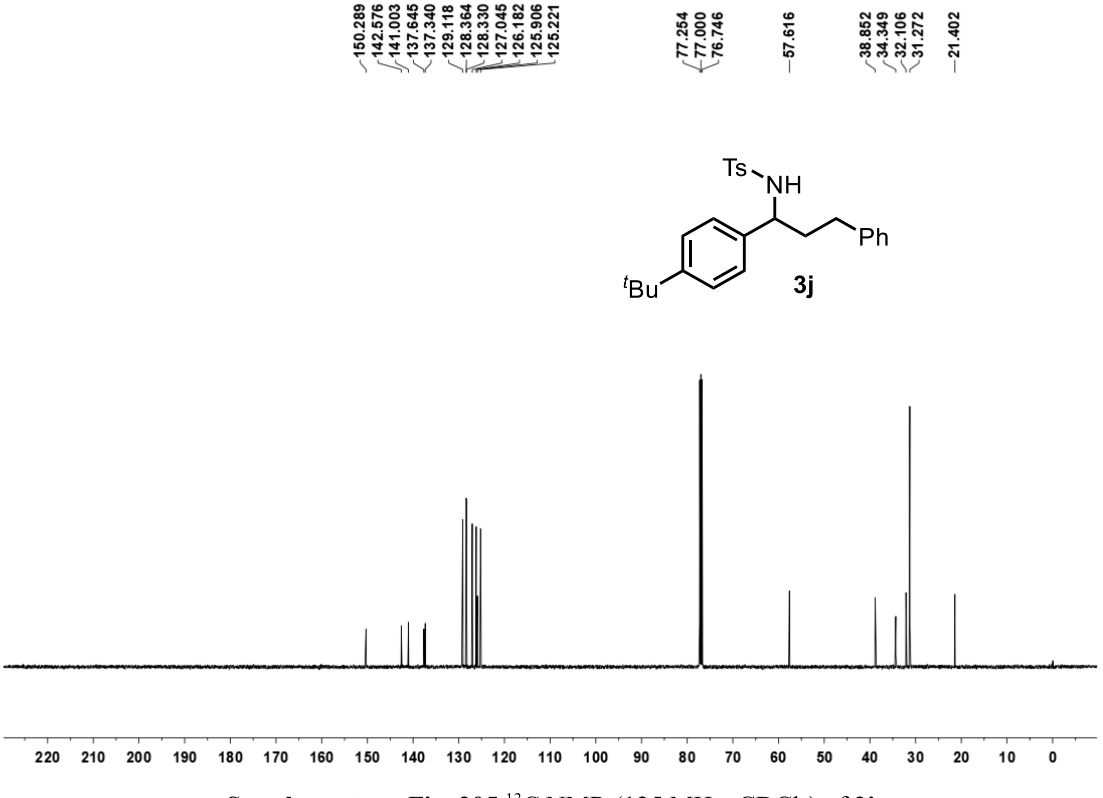

Supplementary Fig. 205 ^{13}C NMR (125 MHz, $CDCl_3$) of **3j**

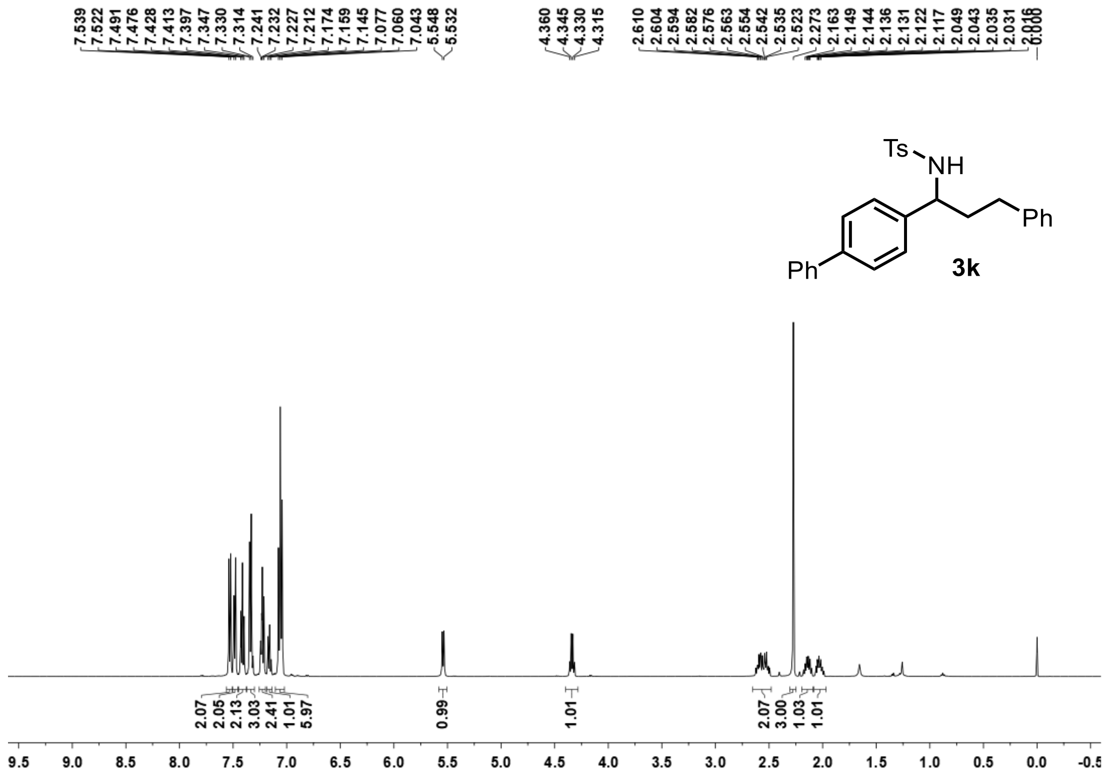

Supplementary Fig. 206 ^{1}H NMR (500 MHz, $CDCl_3$) of **3k**

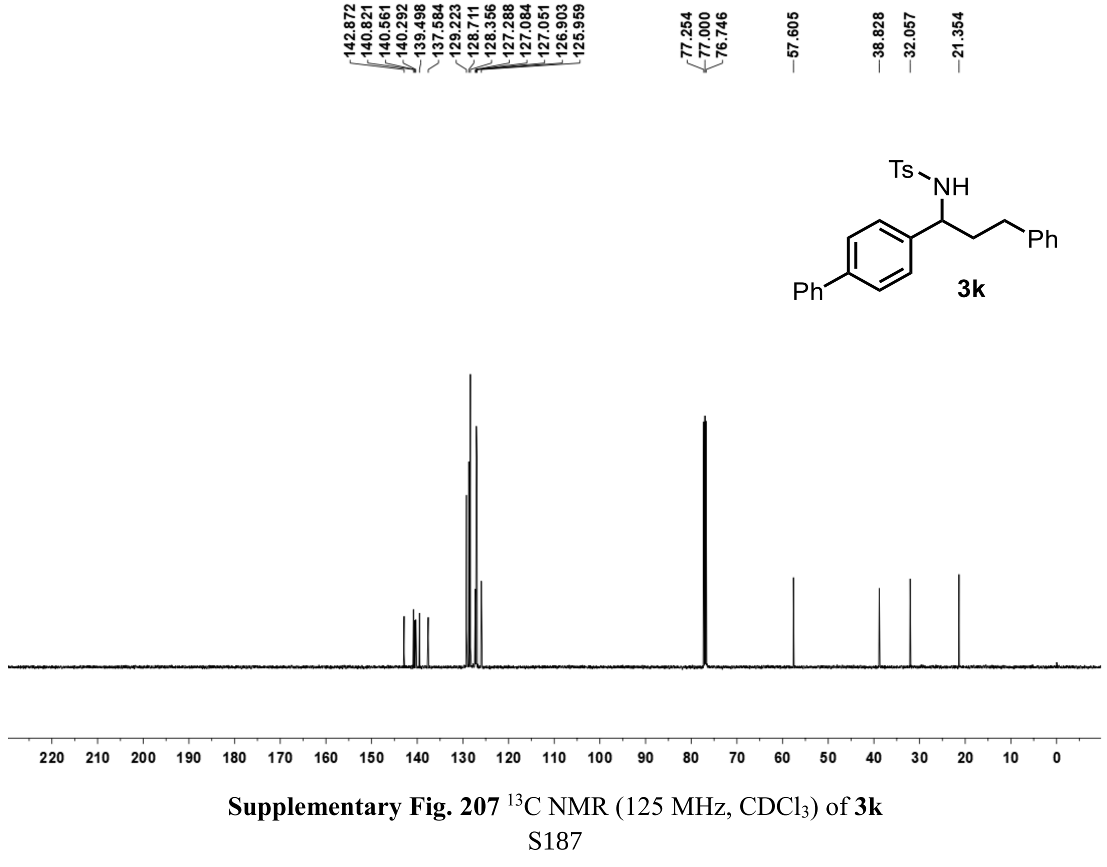

Supplementary Fig. 207 ^{13}C NMR (125 MHz, $CDCl_3$) of **3k**

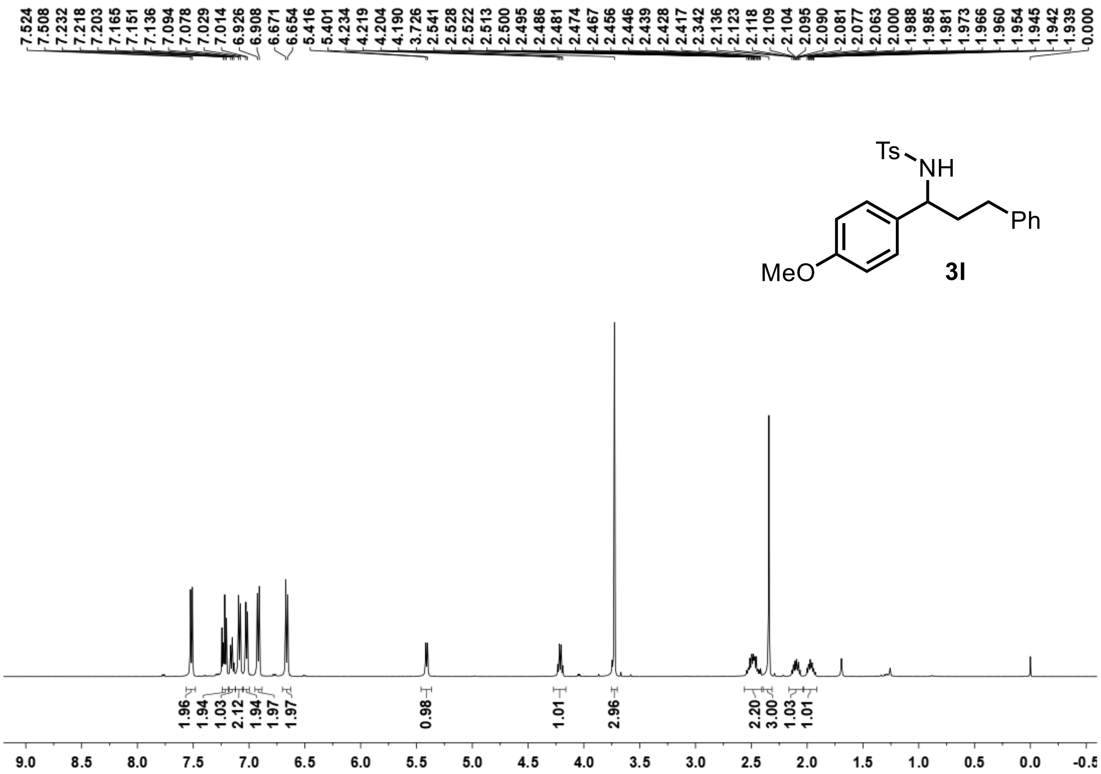

Supplementary Fig. 208 ^{1}H NMR (500 MHz, $CDCl_3$) of **3l**

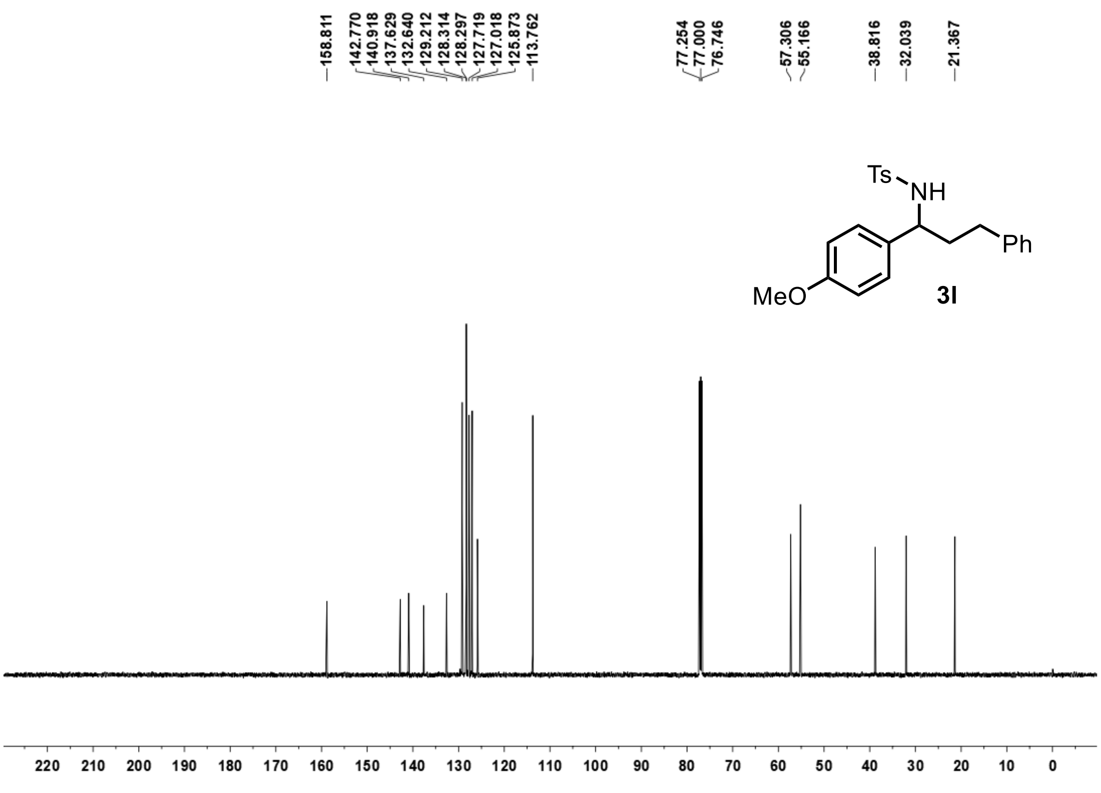

Supplementary Fig. 209 ^{13}C NMR (125 MHz, $CDCl_3$) of **3l**

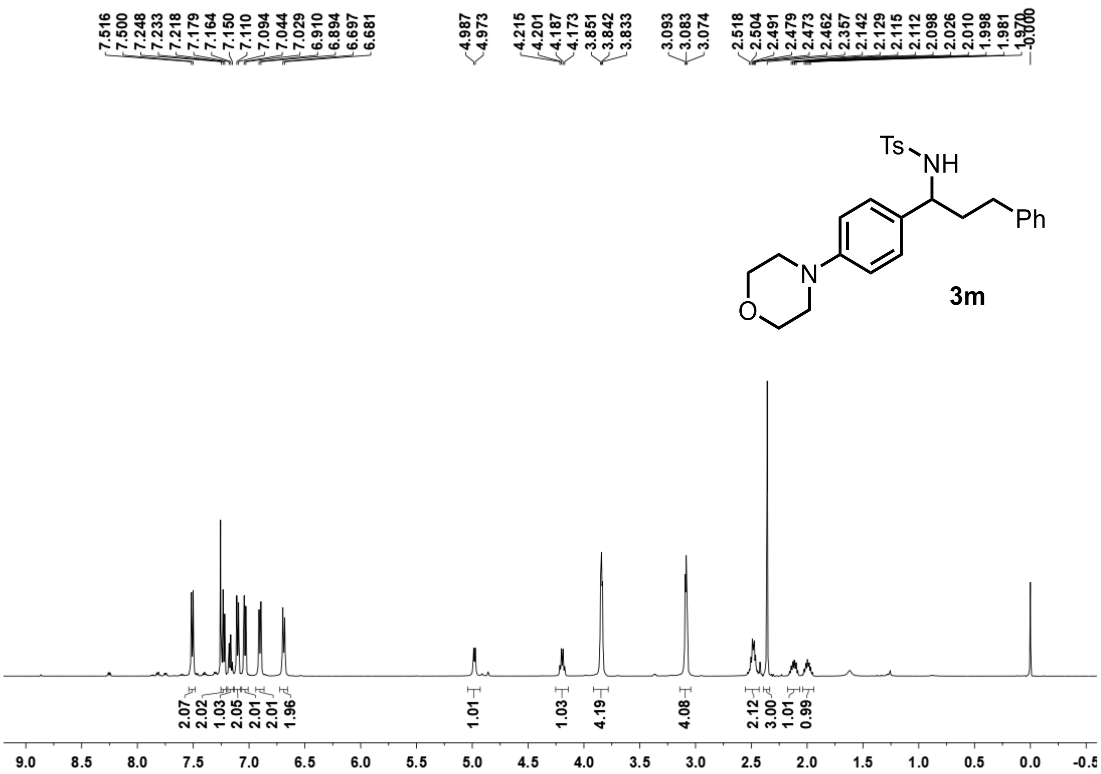

Supplementary Fig. 210 ^{1}H NMR (500 MHz, $CDCl_3$) of **3m**

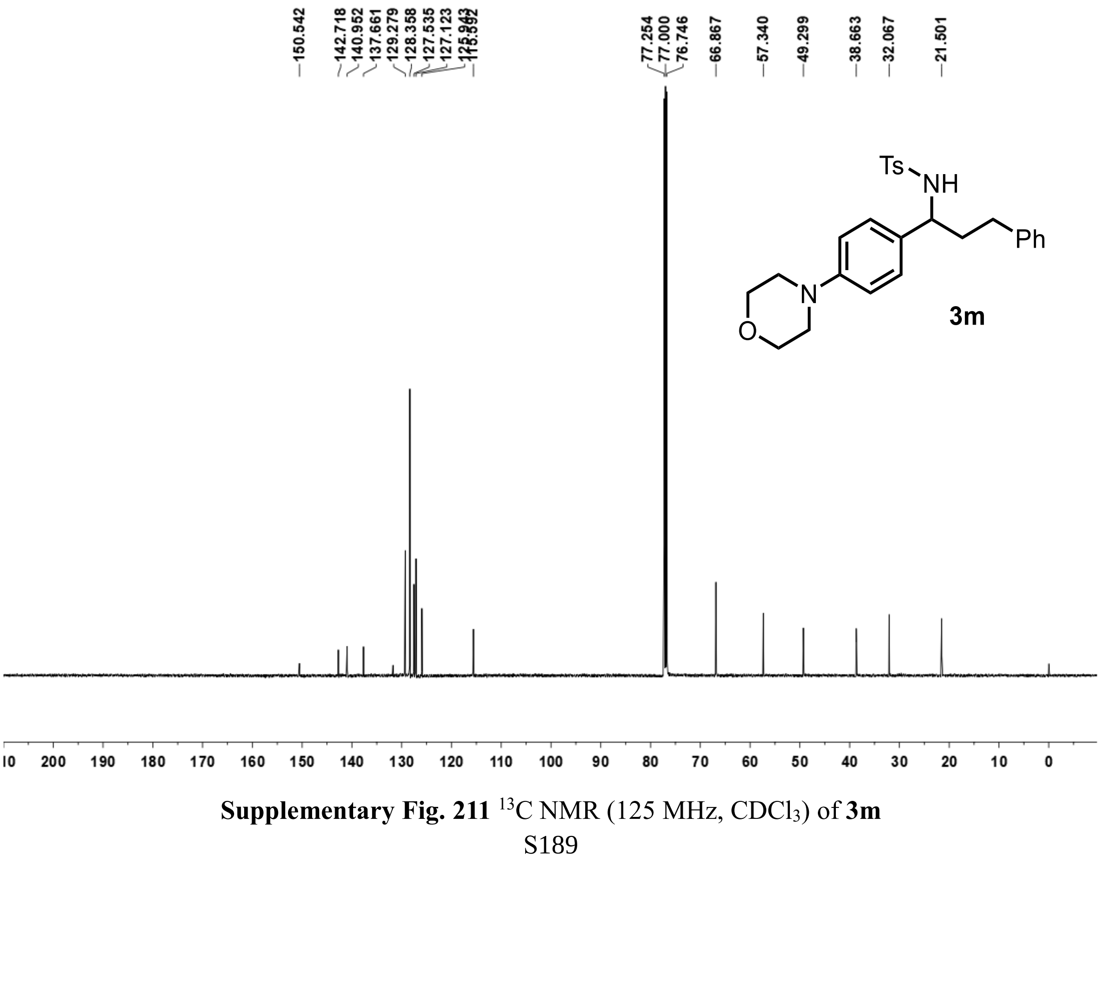

Supplementary Fig. 211 ^{13}C NMR (125 MHz, $CDCl_3$) of **3m**

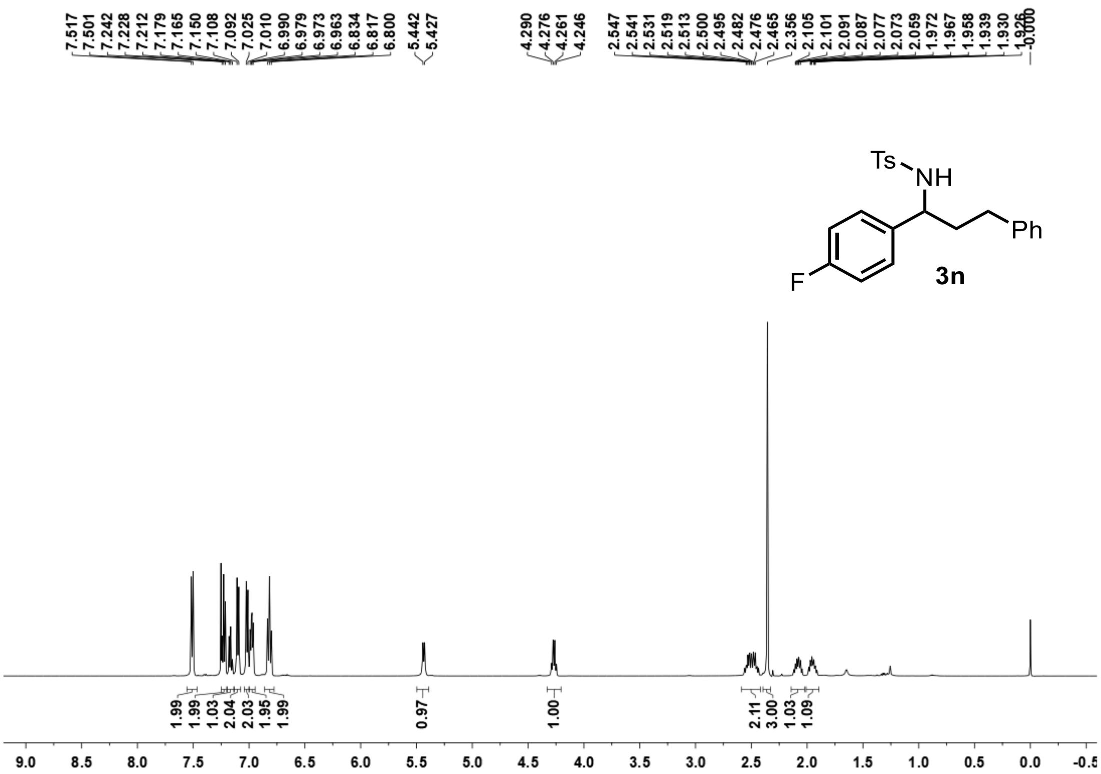

Supplementary Fig. 212 ^{1}H NMR (500 MHz, $CDCl_3$) of **3n**

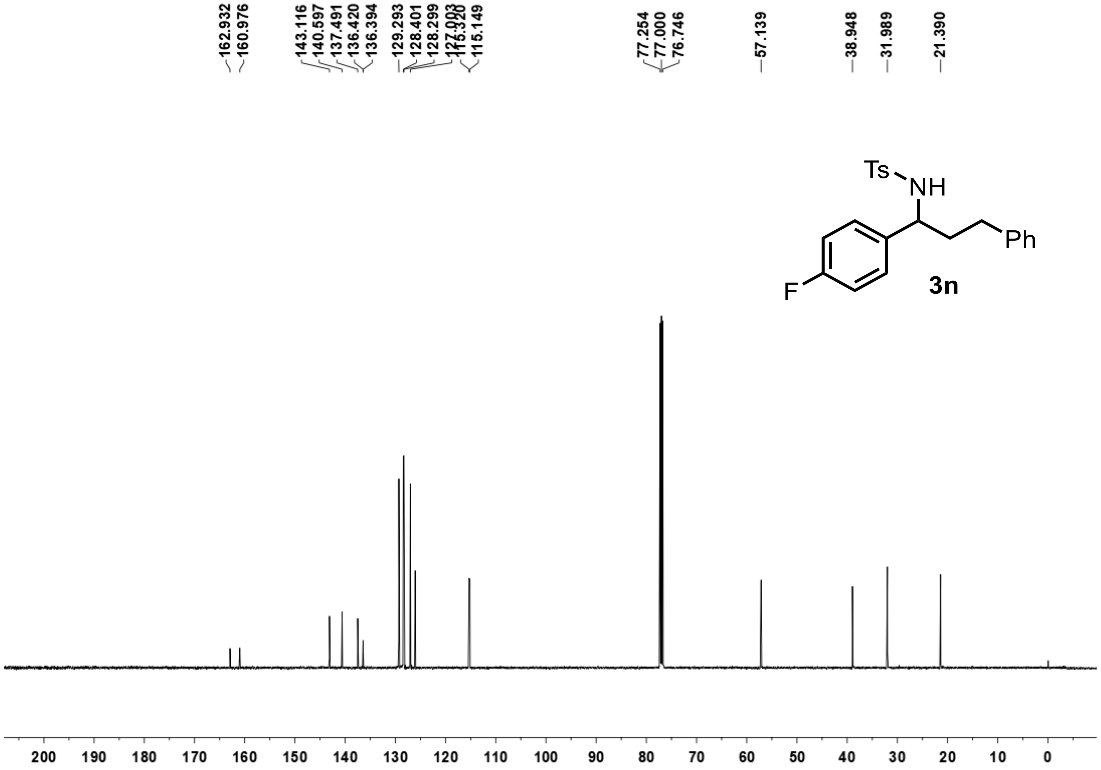

Supplementary Fig. 213 ^{13}C NMR (125 MHz, $CDCl_3$) of **3n**

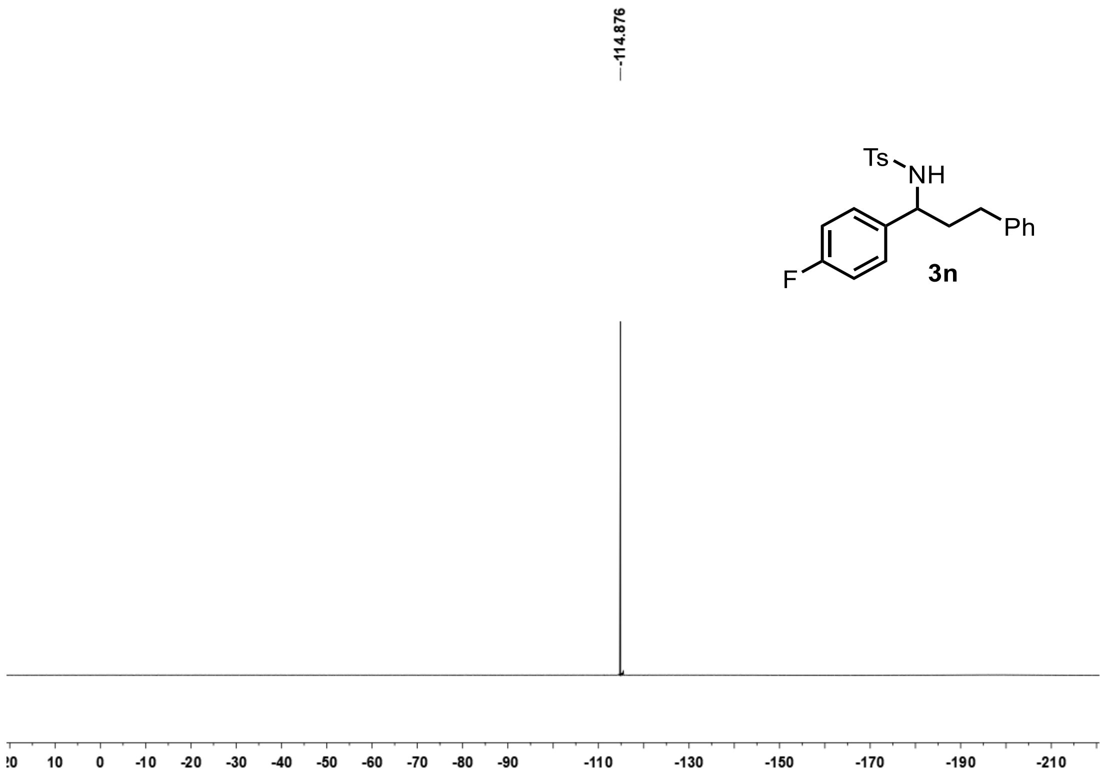

Supplementary Fig. 214 ^{19}F NMR (470 MHz, $CDCl_3$) of **3n**

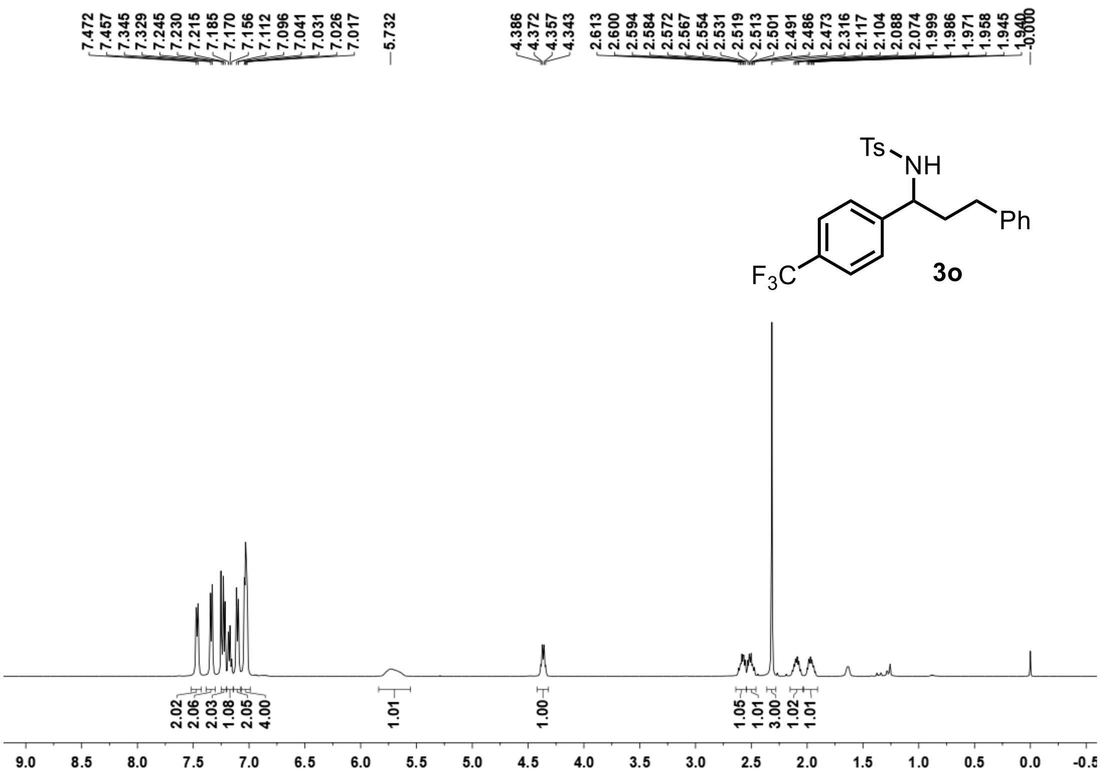

Supplementary Fig. 215 ^{1}H NMR (500 MHz, $CDCl_3$) of **3o**

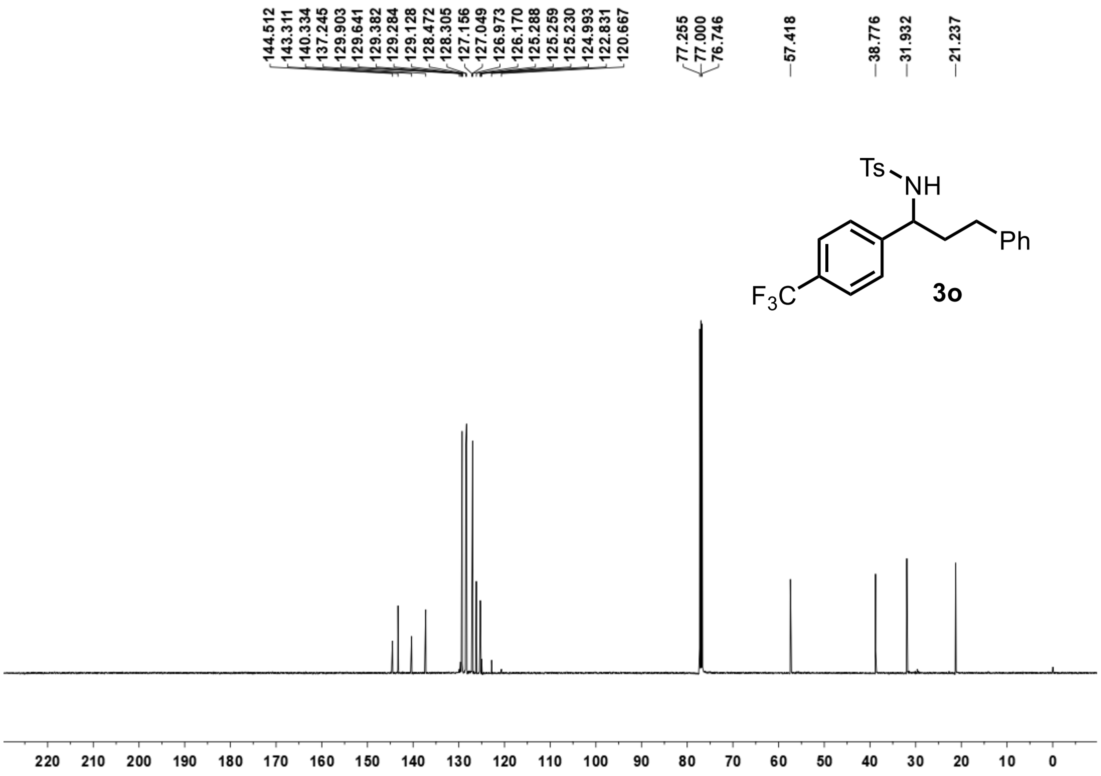

Supplementary Fig. 216 ^{13}C NMR (125 MHz, $CDCl_3$) of **3o**

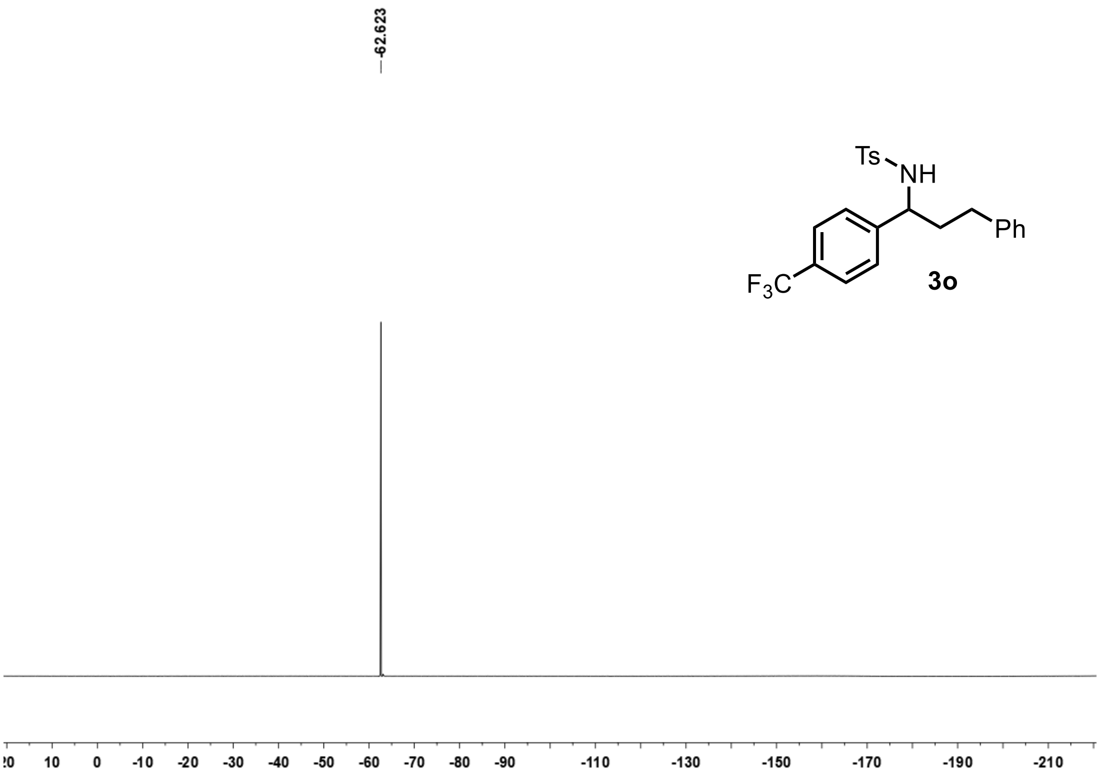

Supplementary Fig. 217 ^{19}F NMR (470 MHz, $CDCl_3$) of **3o**

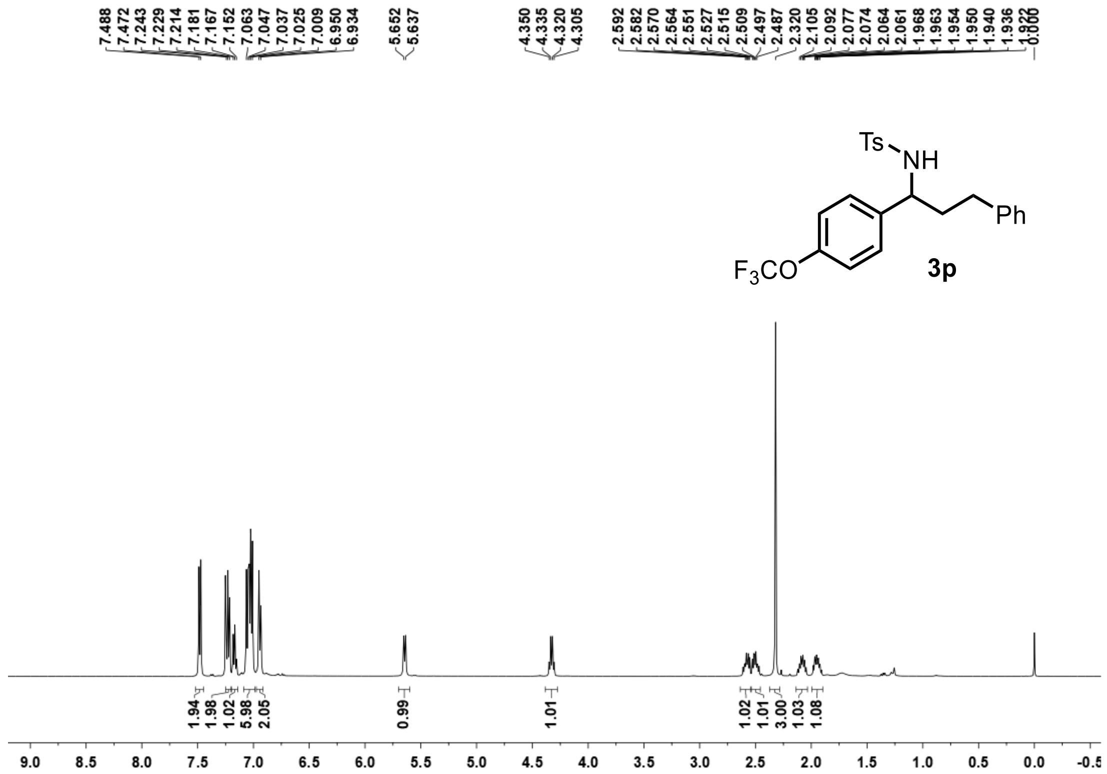

Supplementary Fig. 218 ^{1}H NMR (500 MHz, $CDCl_3$) of **3p**

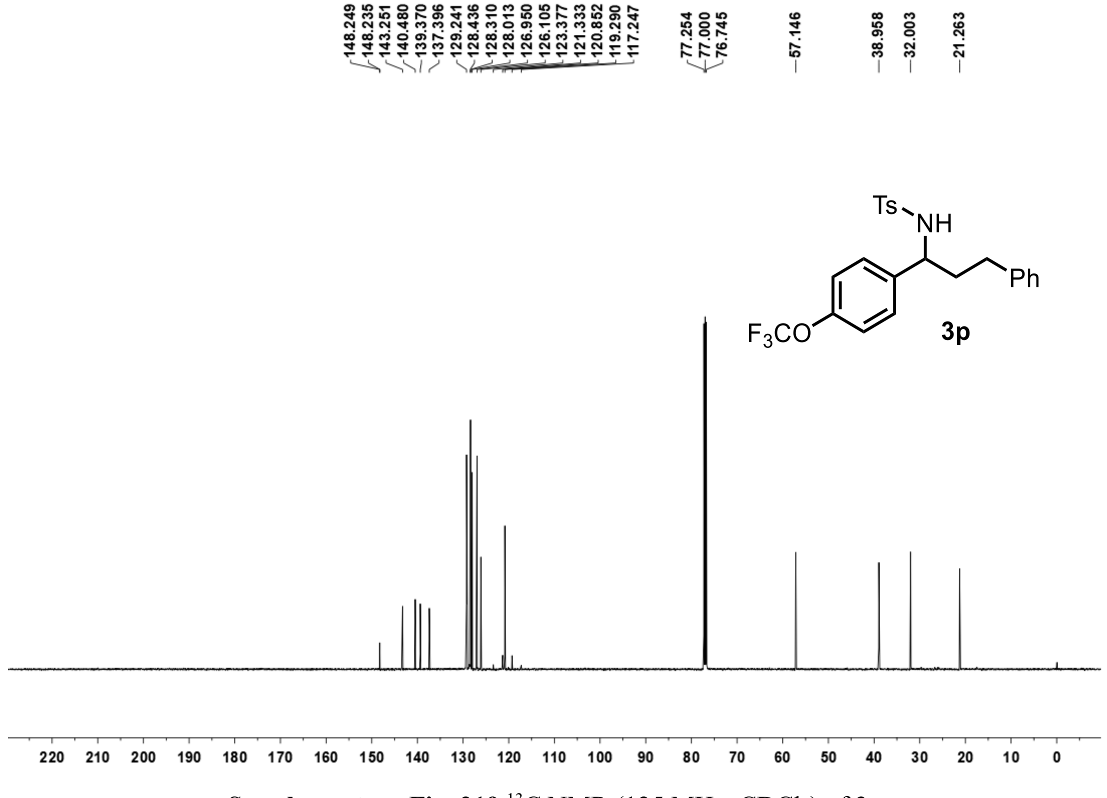

Supplementary Fig. 219 ^{13}C NMR (125 MHz, $CDCl_3$) of **3p**

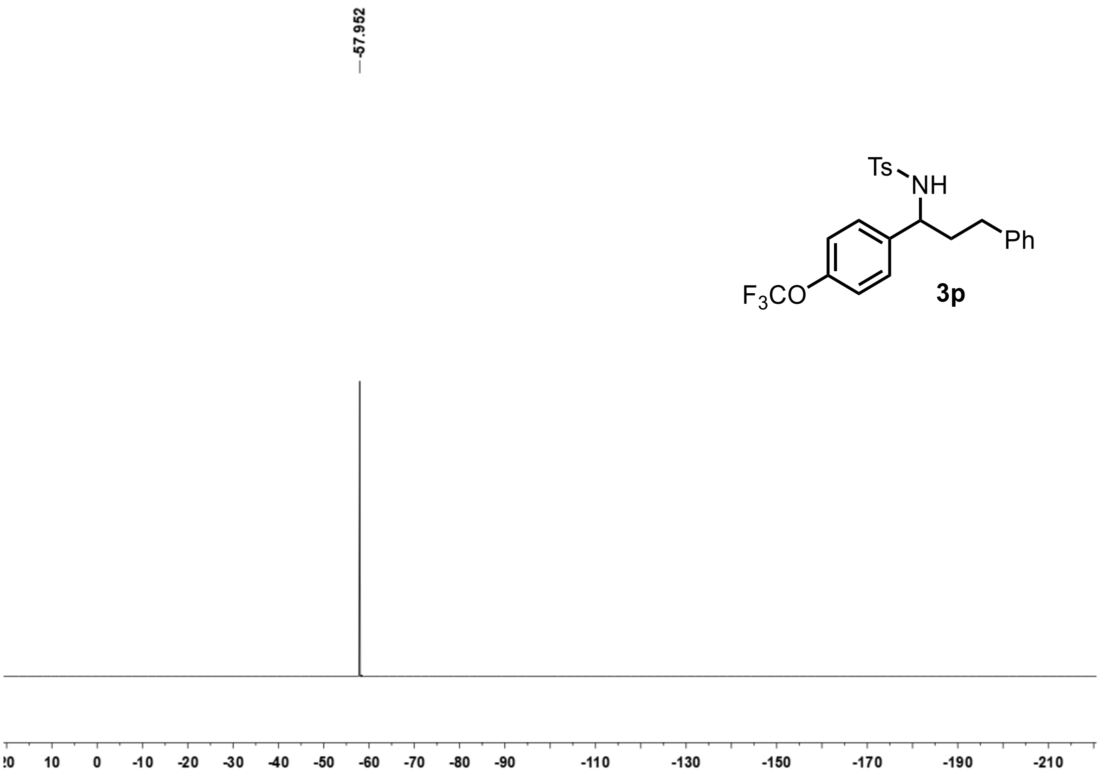

Supplementary Fig. 220 ^{19}F NMR (470 MHz, $CDCl_3$) of **3p**

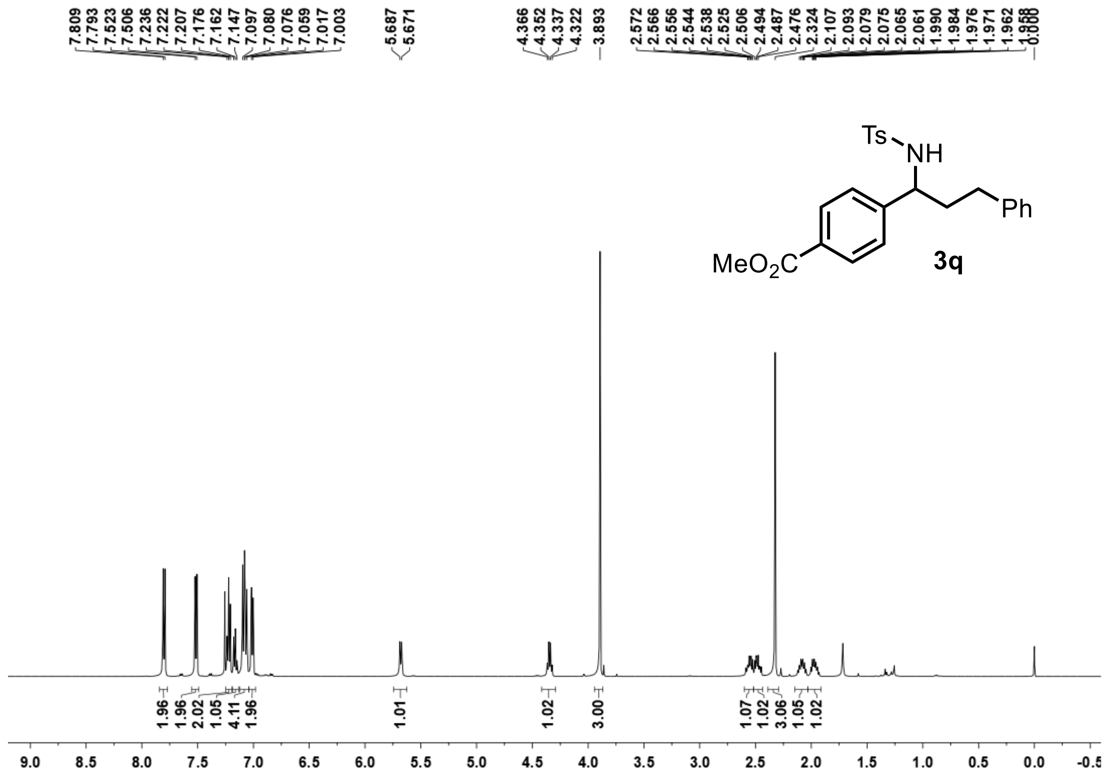

Supplementary Fig. 221 ^{1}H NMR (500 MHz, $CDCl_3$) of **3q**

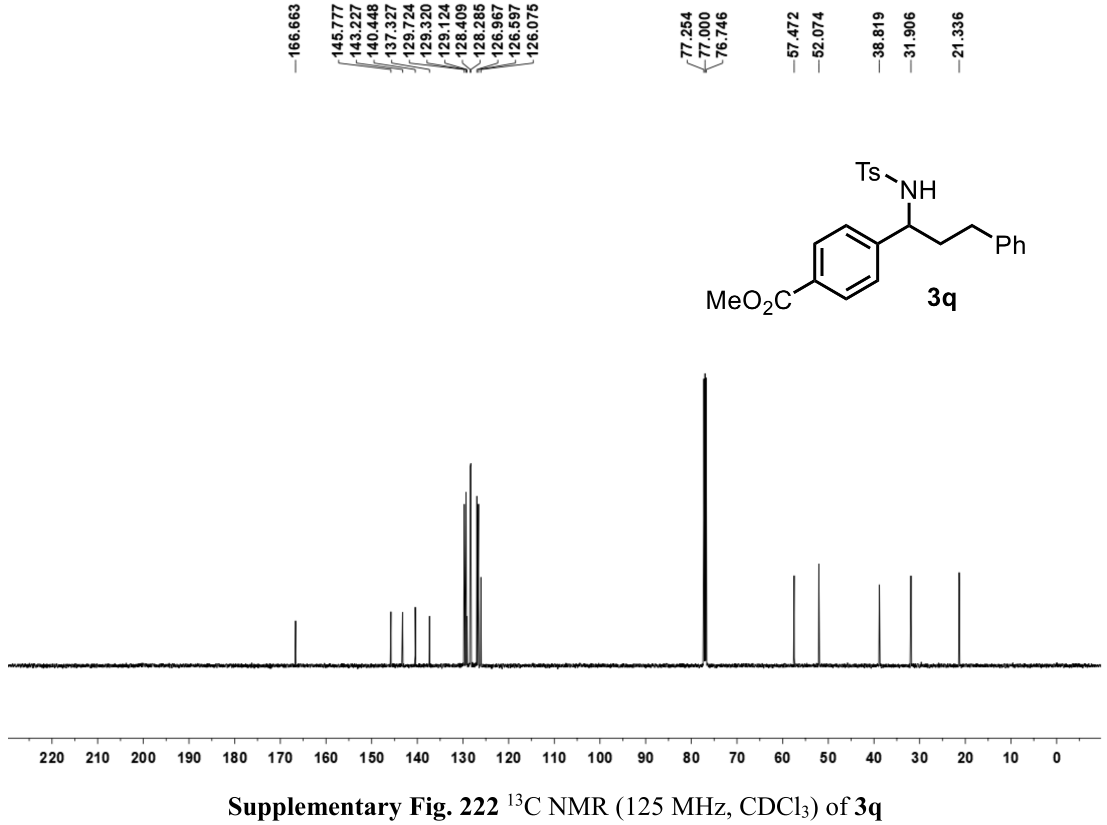

Supplementary Fig. 222 ^{13}C NMR (125 MHz, $CDCl_3$) of **3q**

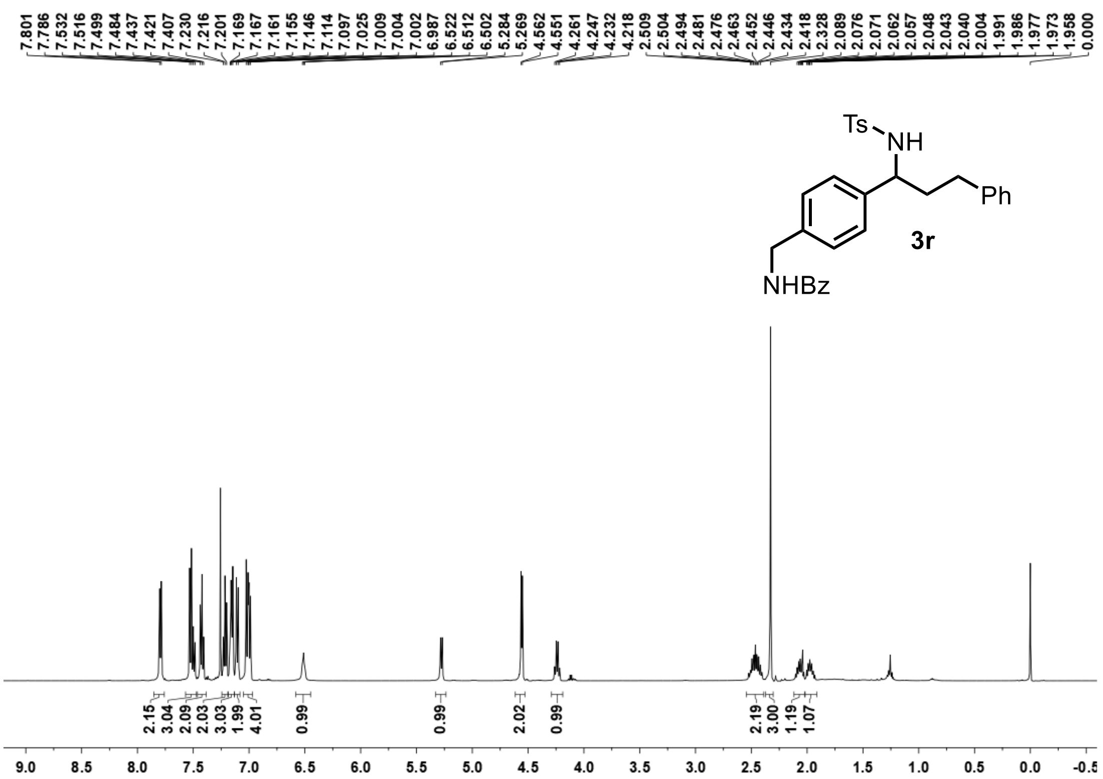

Supplementary Fig. 223 ^{1}H NMR (500 MHz, $CDCl_3$) of **3r**

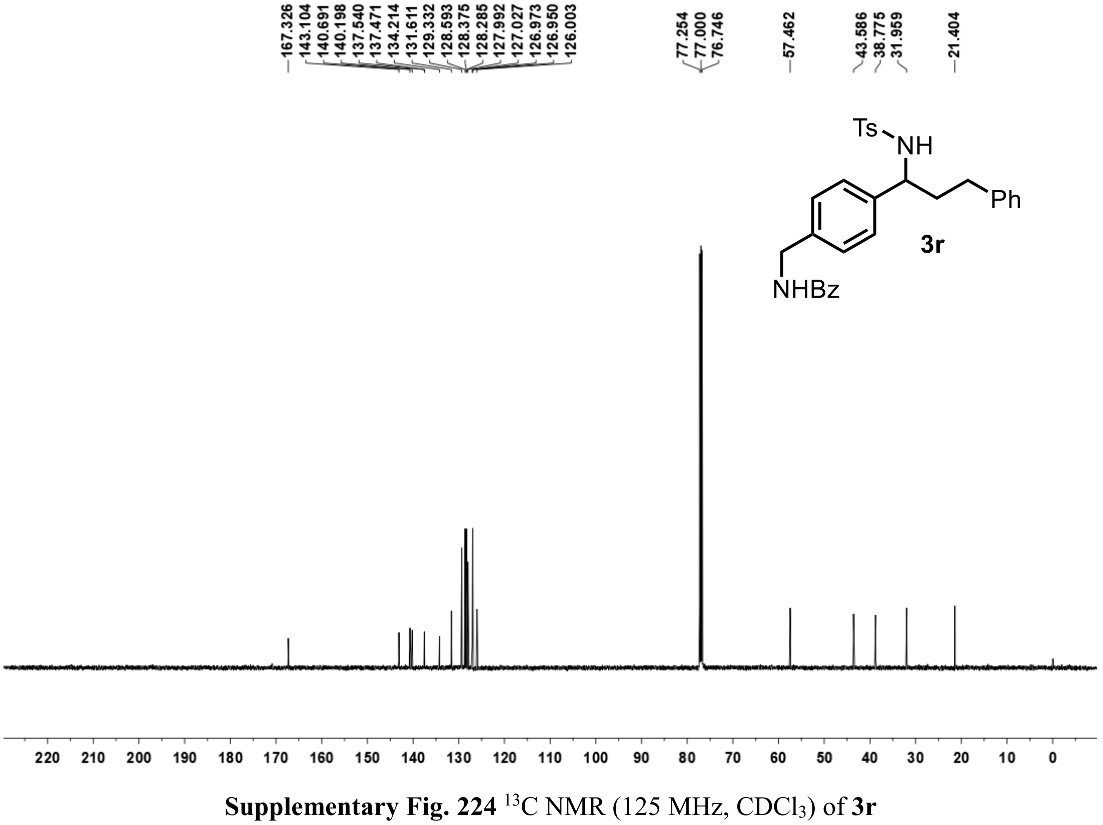

Supplementary Fig. 224 ^{13}C NMR (125 MHz, $CDCl_3$) of **3r**

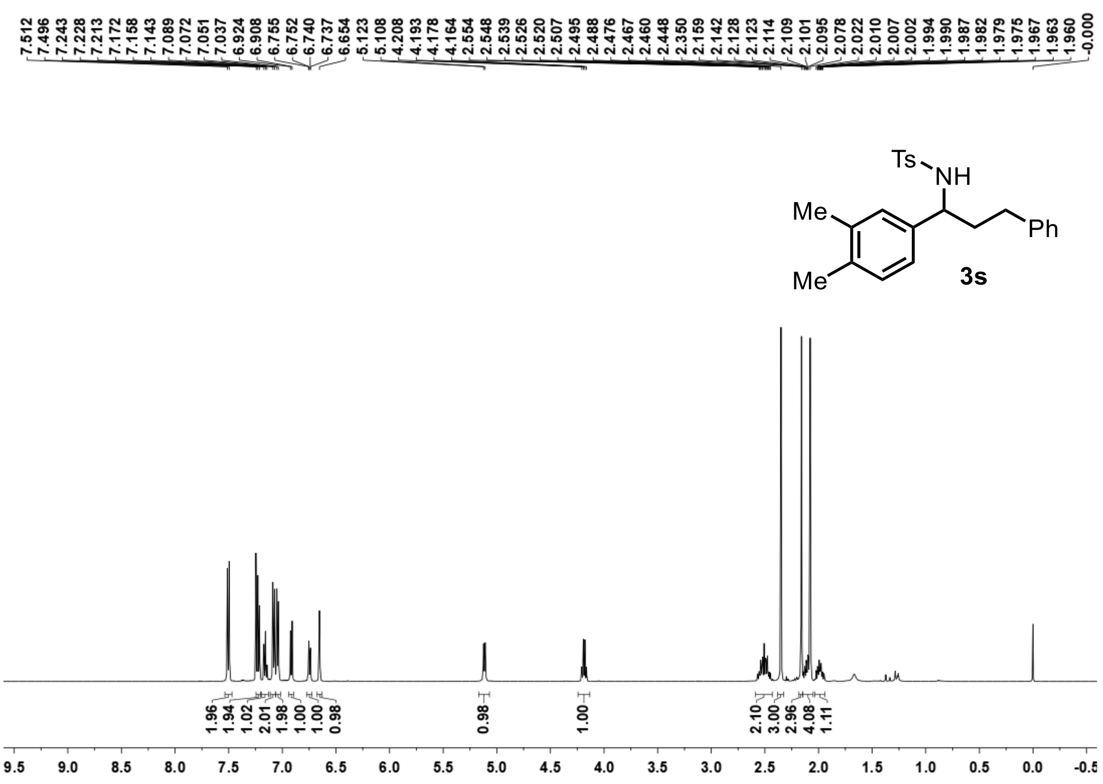

Supplementary Fig. 225 ^{1}H NMR (500 MHz, $CDCl_3$) of **3s**

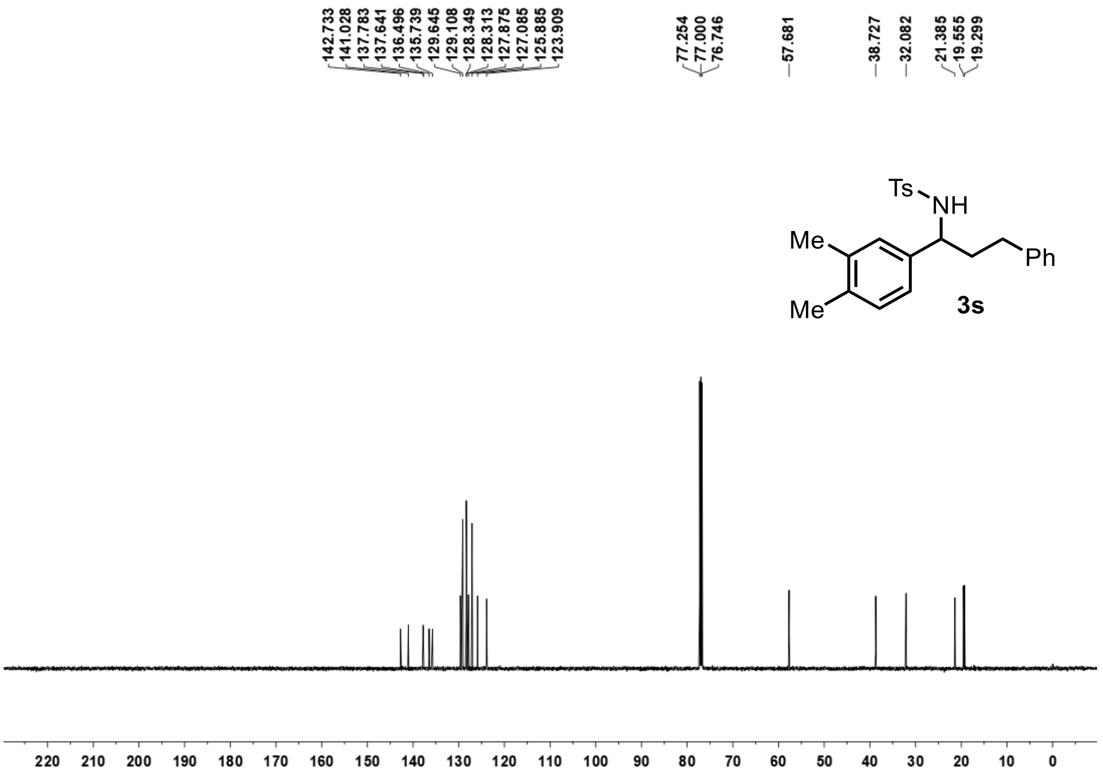

Supplementary Fig. 226 ^{13}C NMR (125 MHz, $CDCl_3$) of **3s**

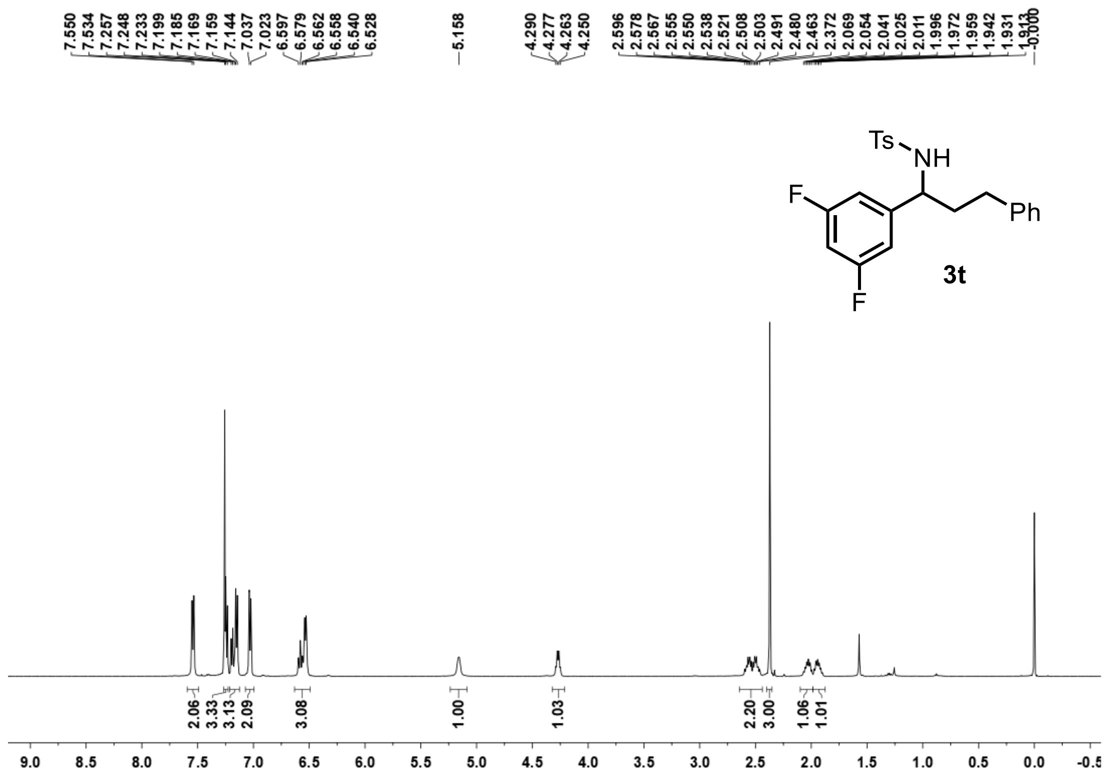

Supplementary Fig. 227 ^{1}H NMR (500 MHz, $CDCl_3$) of **3t**

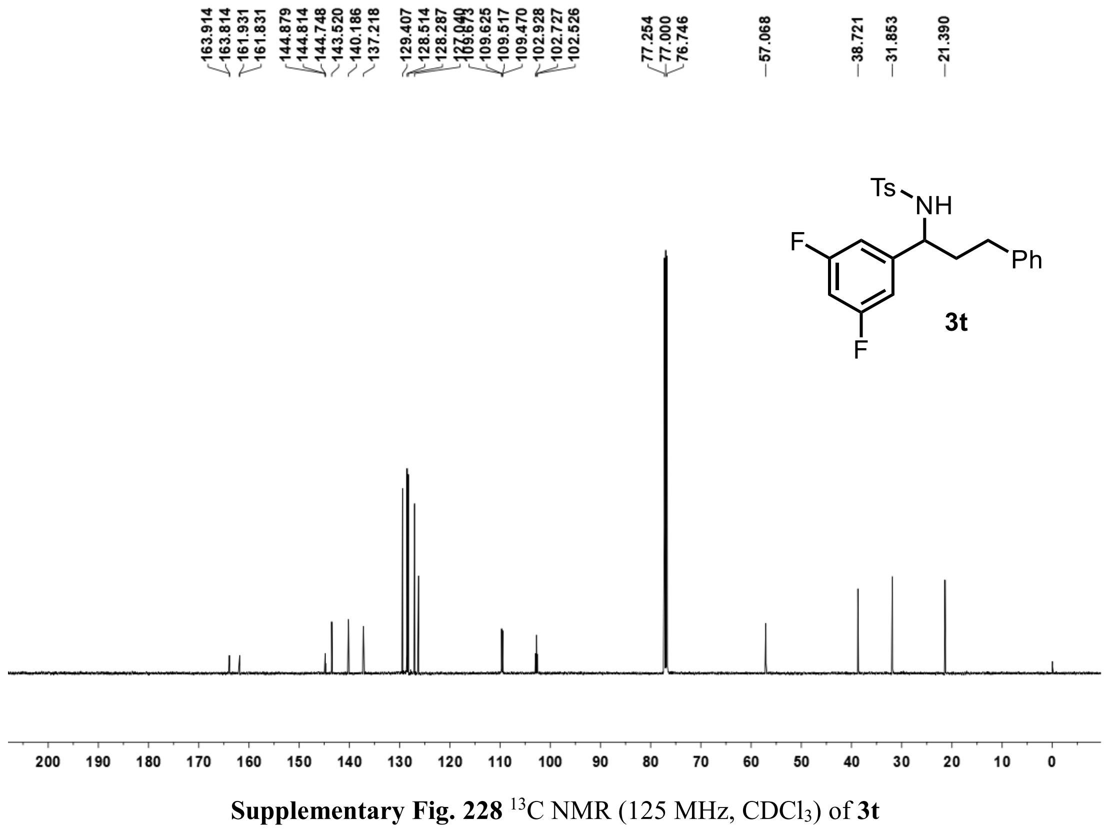

Supplementary Fig. 228 ^{13}C NMR (125 MHz, $CDCl_3$) of **3t**

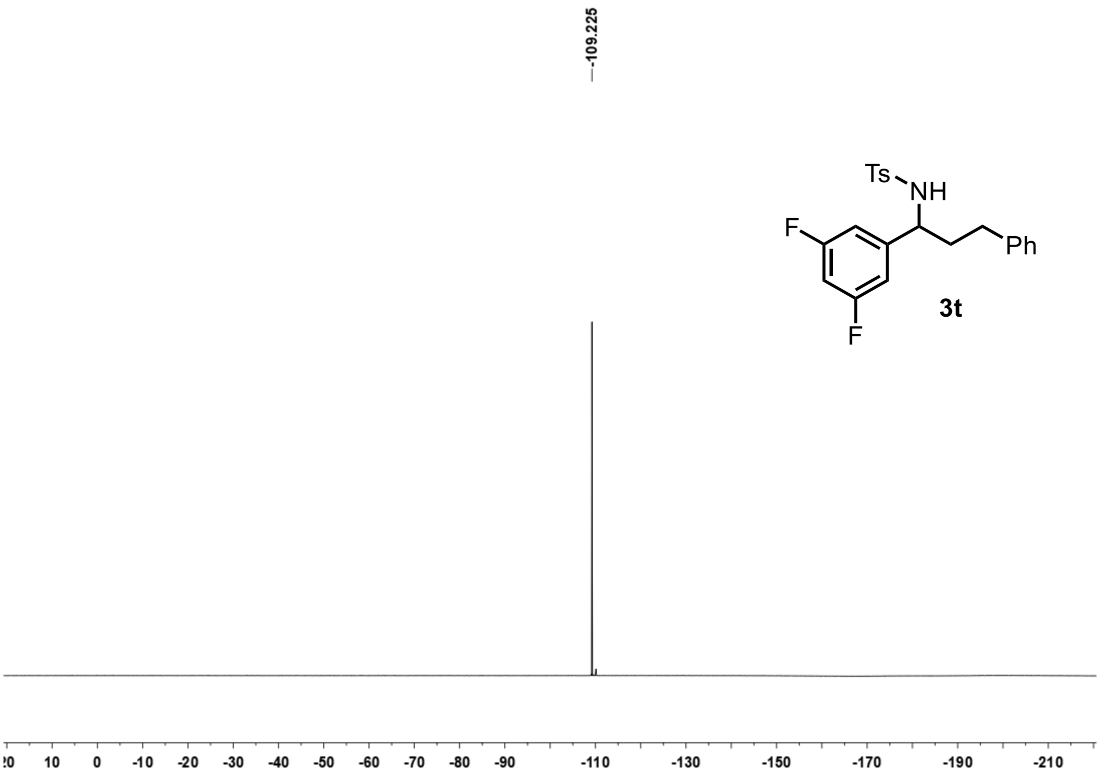

Supplementary Fig. 229 ^{19}F NMR (470 MHz, $CDCl_3$) of **3t**

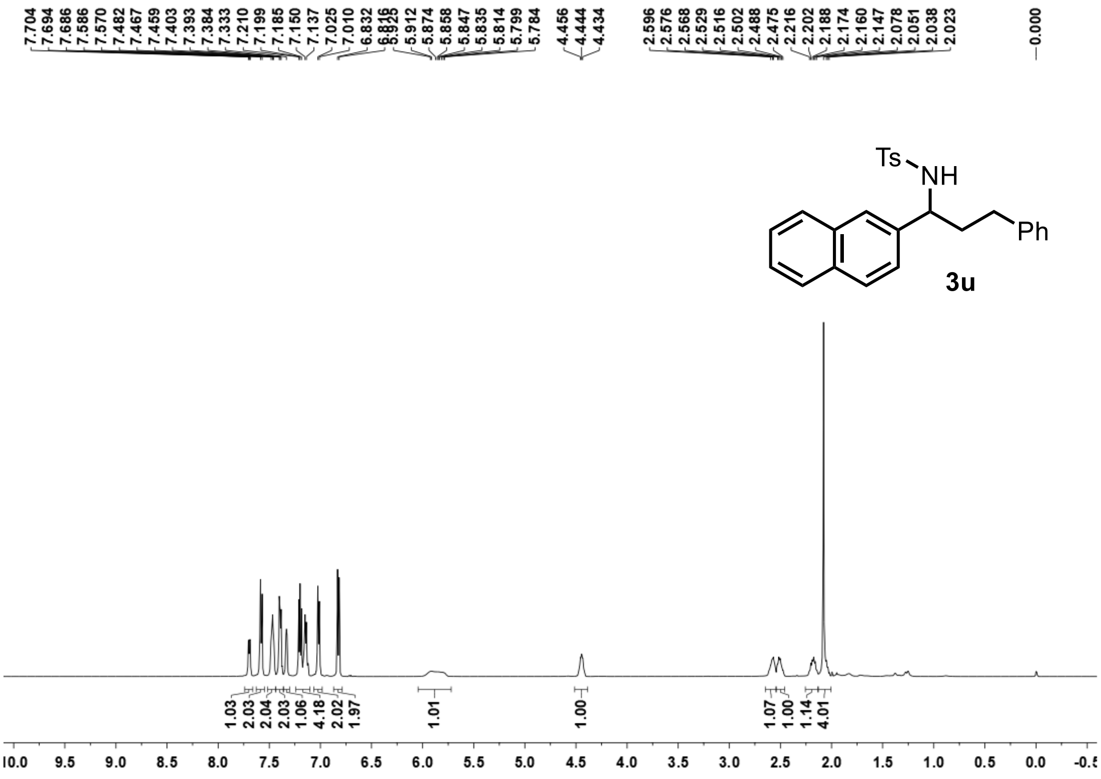

Supplementary Fig. 230 ^{1}H NMR (500 MHz, $CDCl_3$) of **3u**

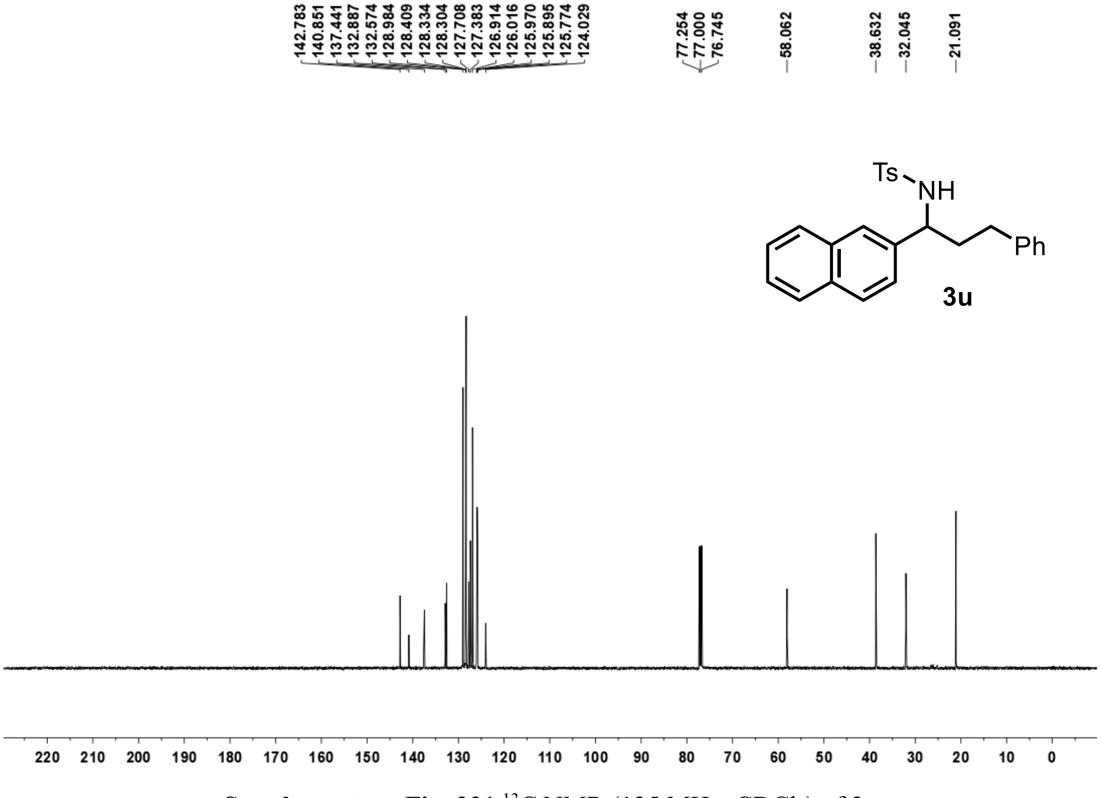

Supplementary Fig. 231 ^{13}C NMR (125 MHz, $CDCl_3$) of **3u**

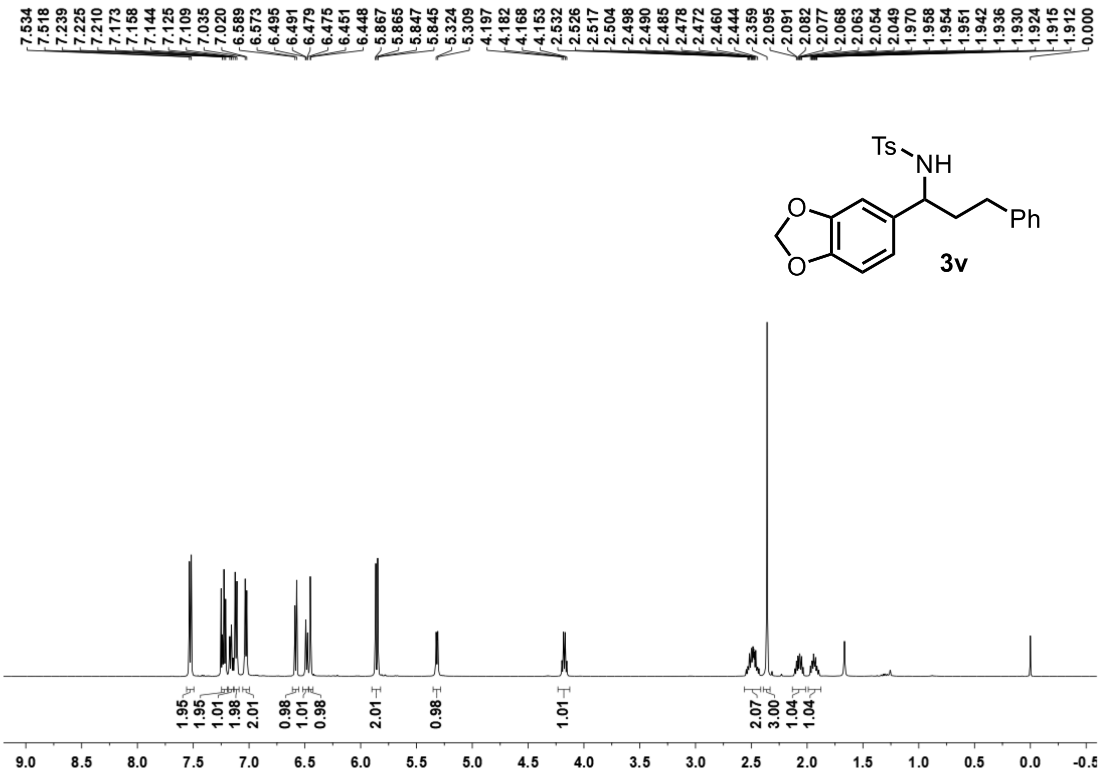

Supplementary Fig. 232 ^{1}H NMR (500 MHz, $CDCl_3$) of **3v**

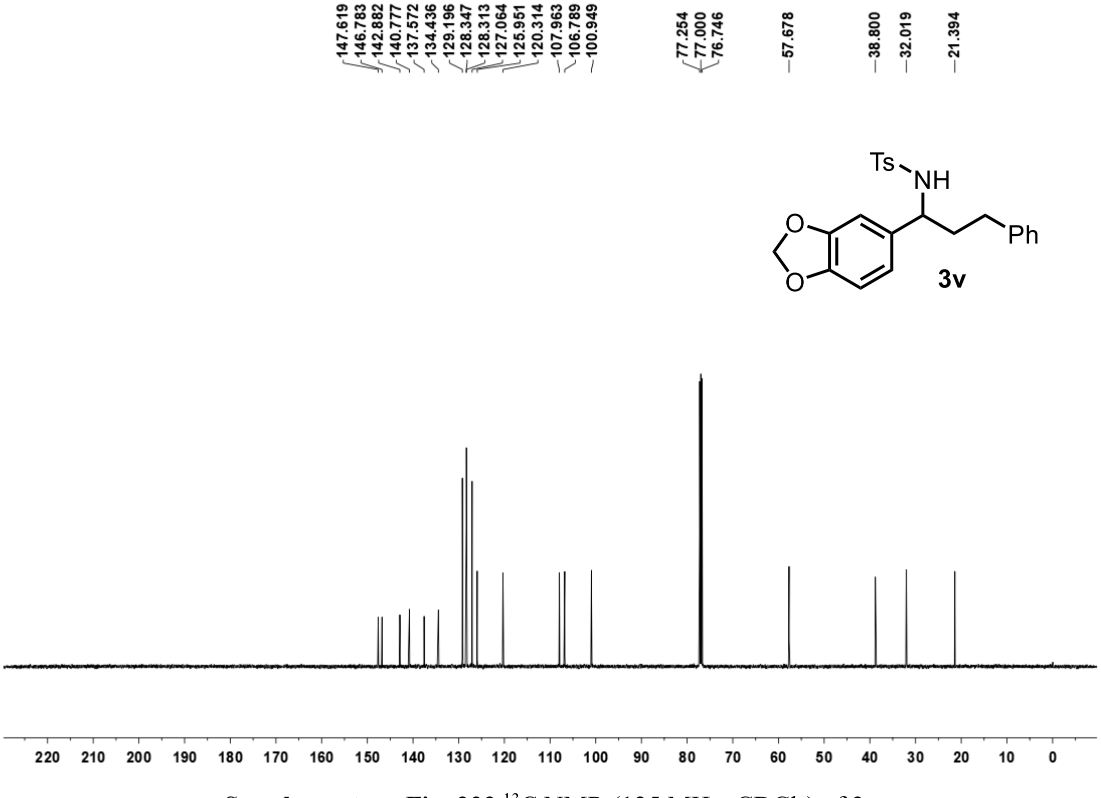

Supplementary Fig. 233 ^{13}C NMR (125 MHz, $CDCl_3$) of **3v**

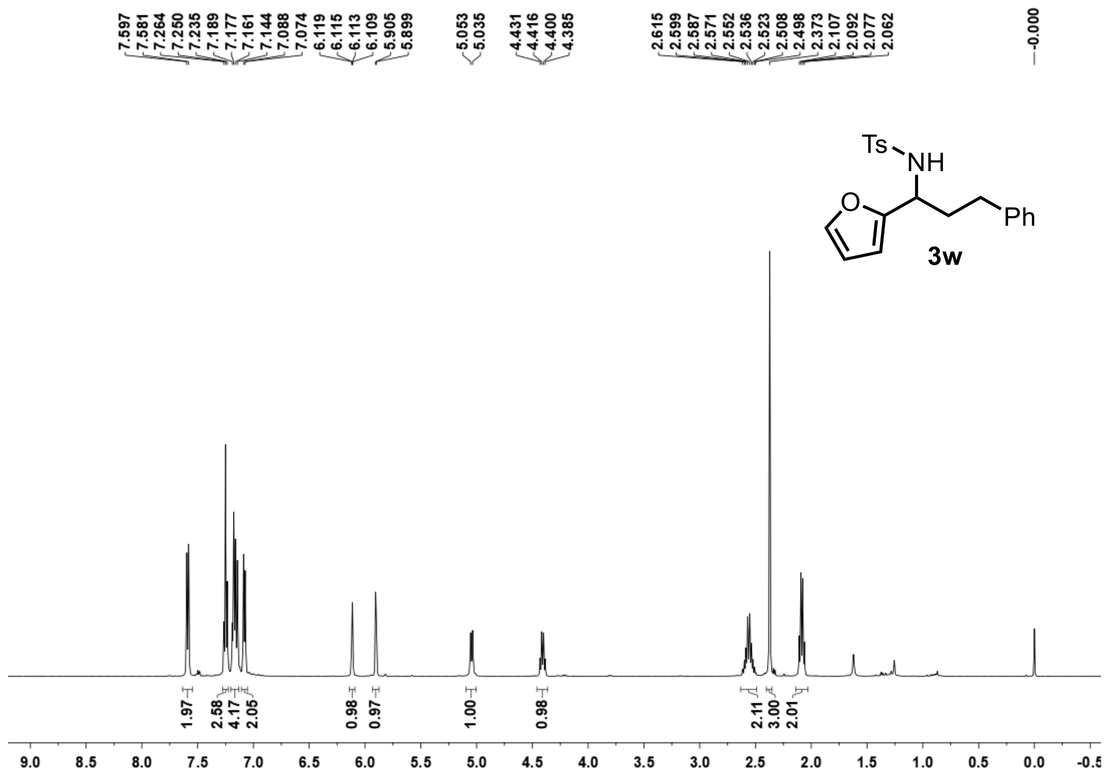

Supplementary Fig. 234 ^{1}H NMR (500 MHz, $CDCl_3$) of **3w**

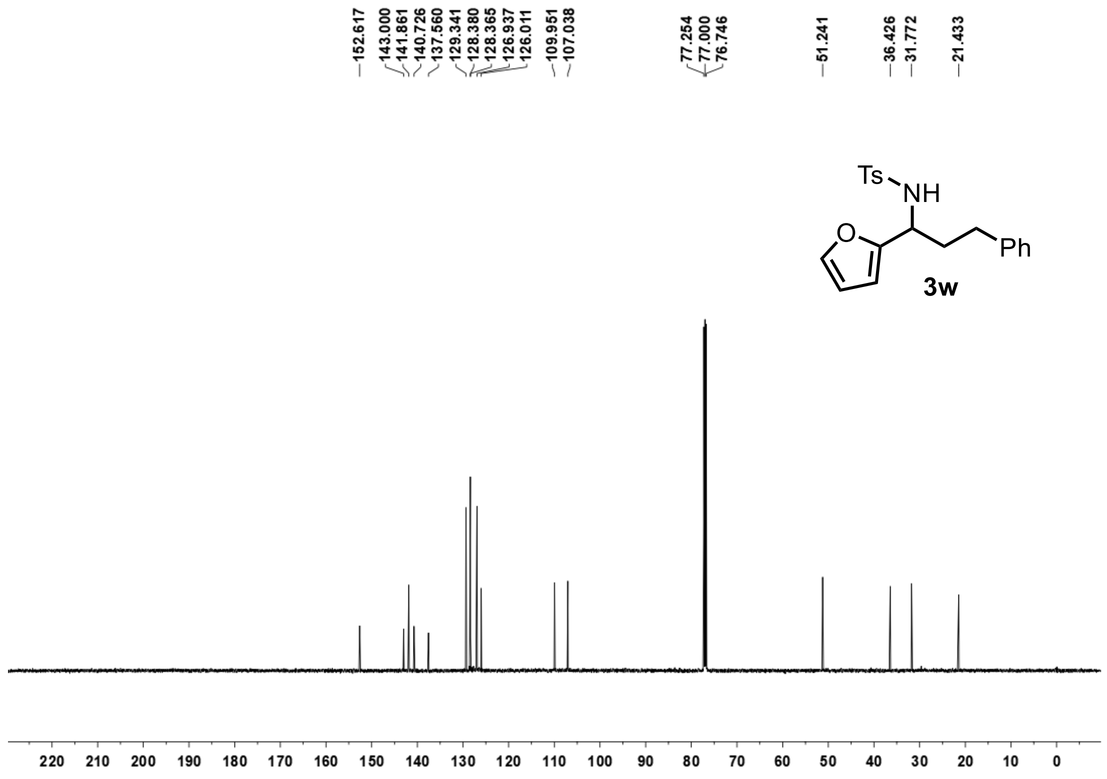

Supplementary Fig. 235 ^{13}C NMR (125 MHz, $CDCl_3$) of **3w**

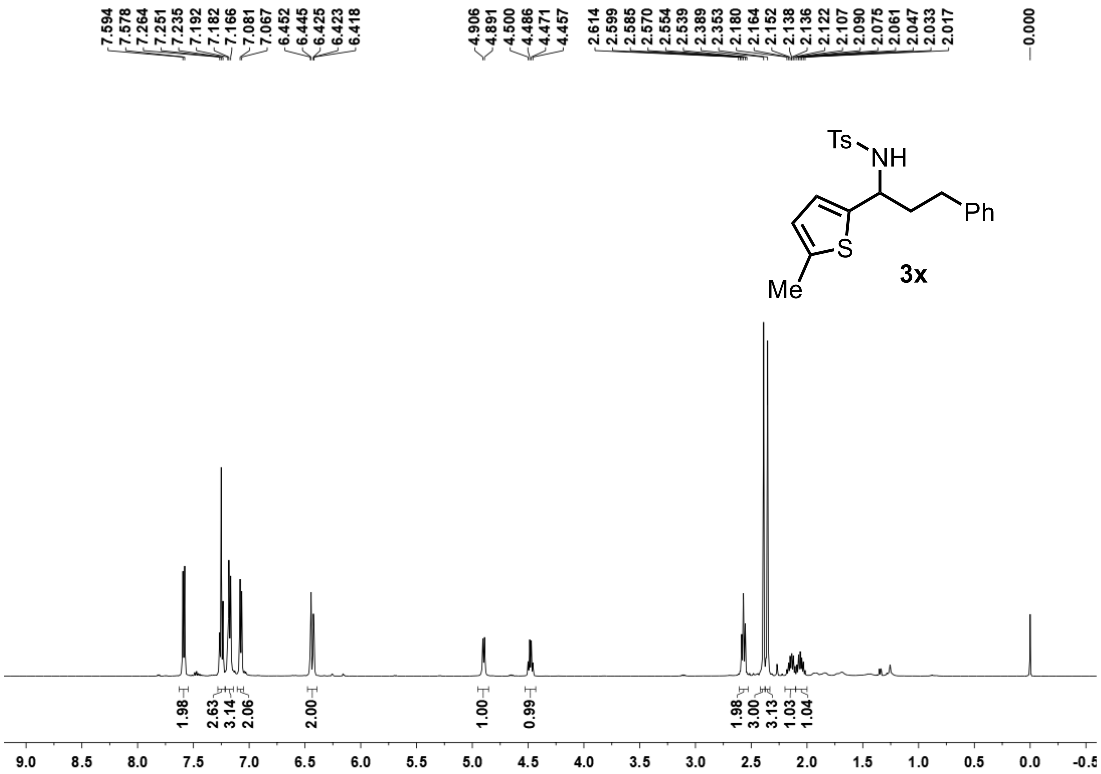

Supplementary Fig. 236 ^{1}H NMR (500 MHz, $CDCl_3$) of **3x**

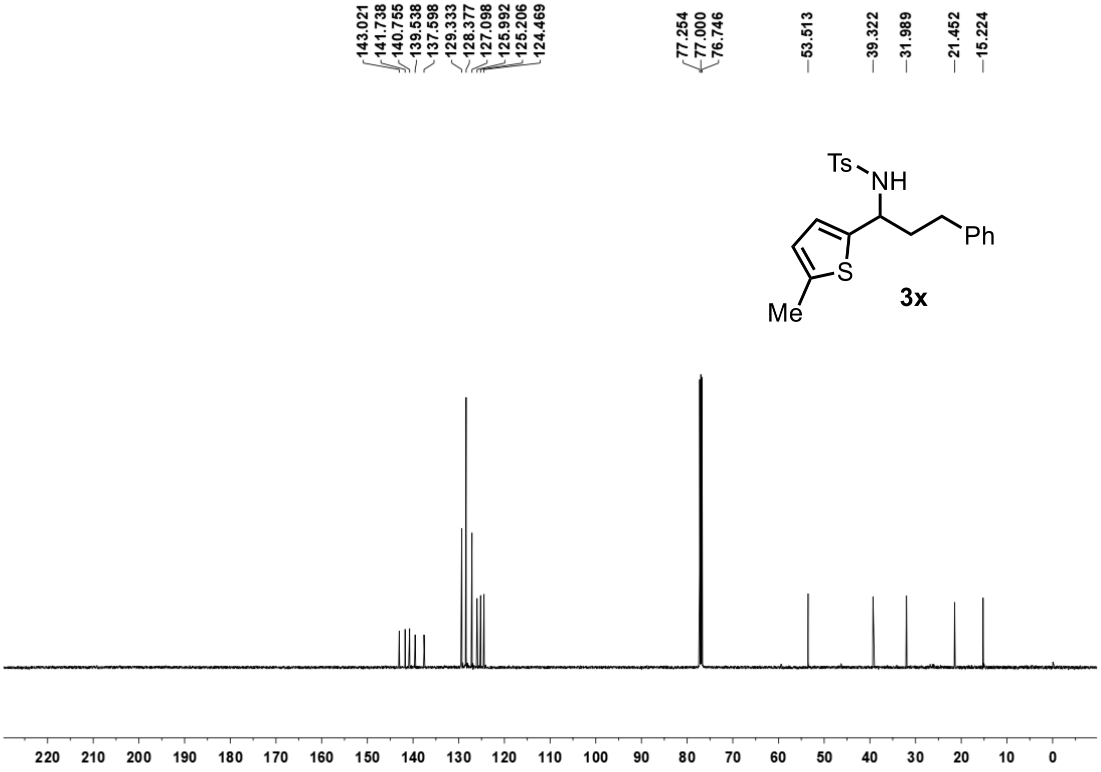

Supplementary Fig. 237 ^{13}C NMR (125 MHz, $CDCl_3$) of **3x**

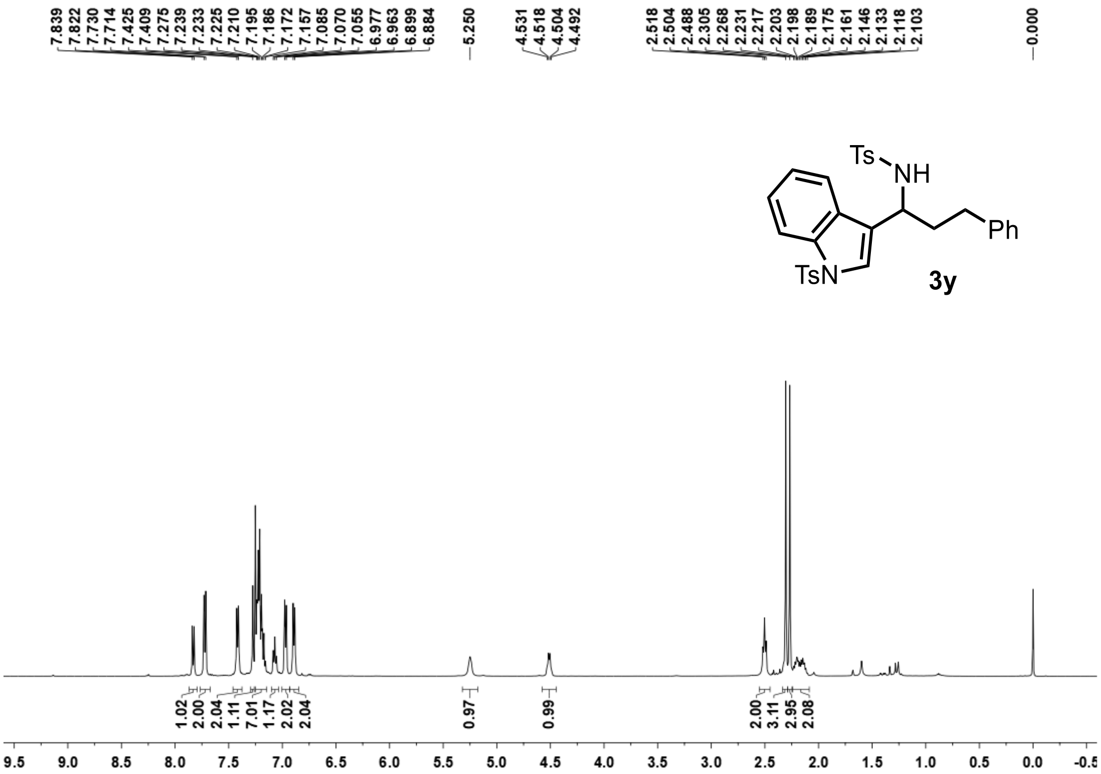

Supplementary Fig. 238 ^{1}H NMR (500 MHz, $CDCl_3$) of **3y**

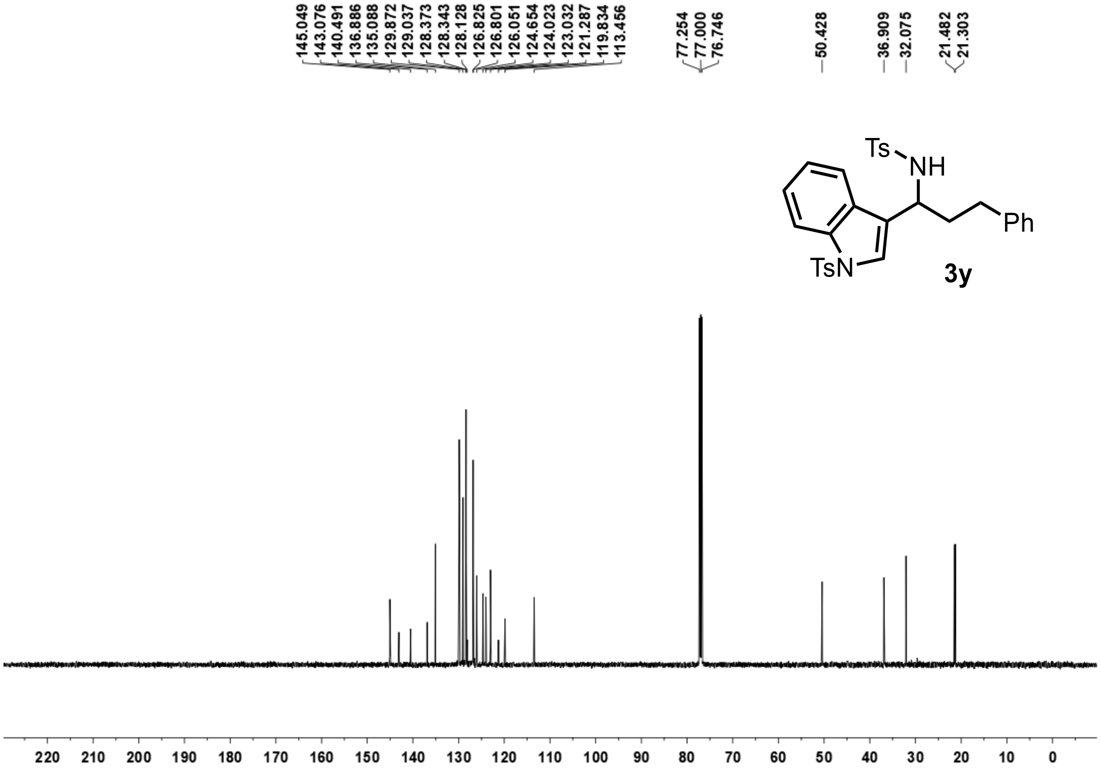

Supplementary Fig. 239 ^{13}C NMR (125 MHz, $CDCl_3$) of **3y**

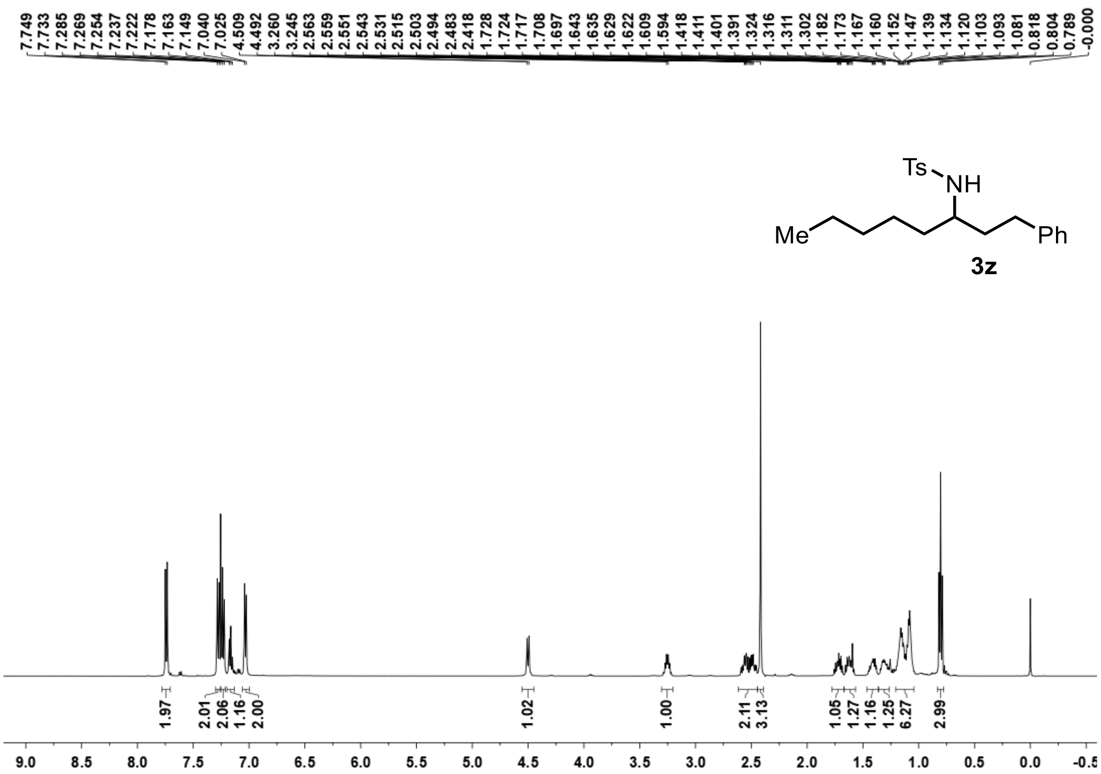

Supplementary Fig. 240 ^{1}H NMR (500 MHz, $CDCl_3$) of **3z**

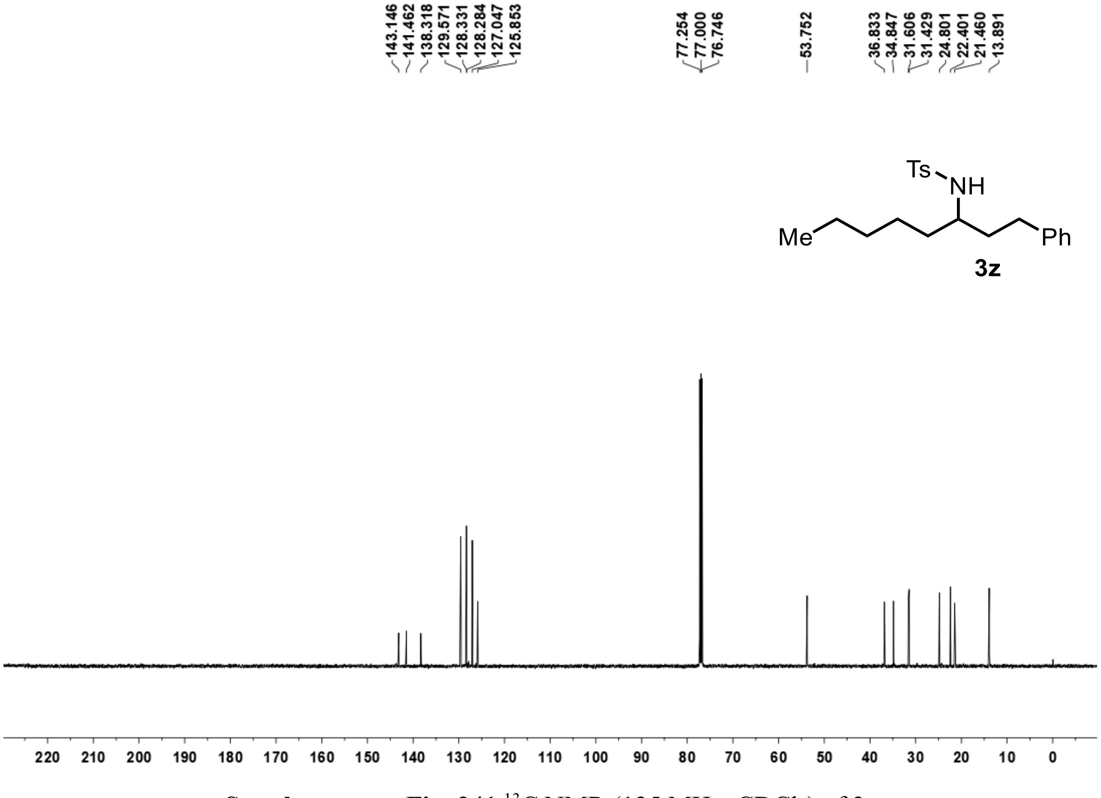

Supplementary Fig. 241 ^{13}C NMR (125 MHz, $CDCl_3$) of **3z**

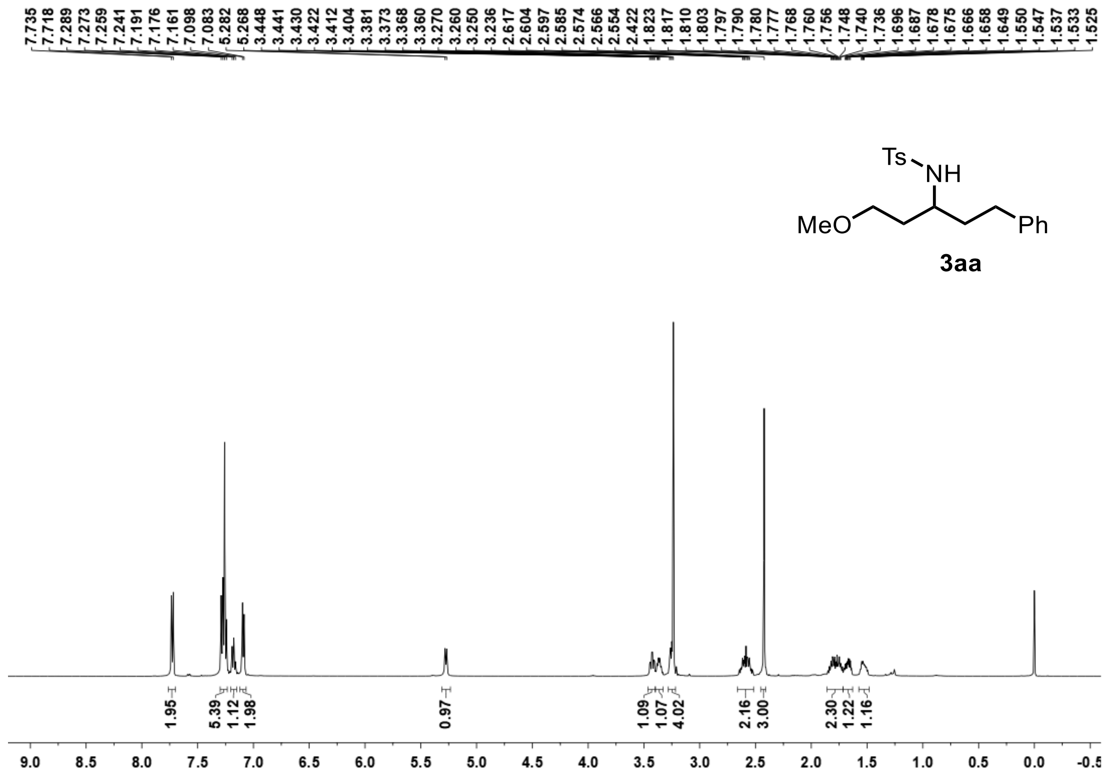

Supplementary Fig. 242 ^{1}H NMR (500 MHz, $CDCl_3$) of **3aa**

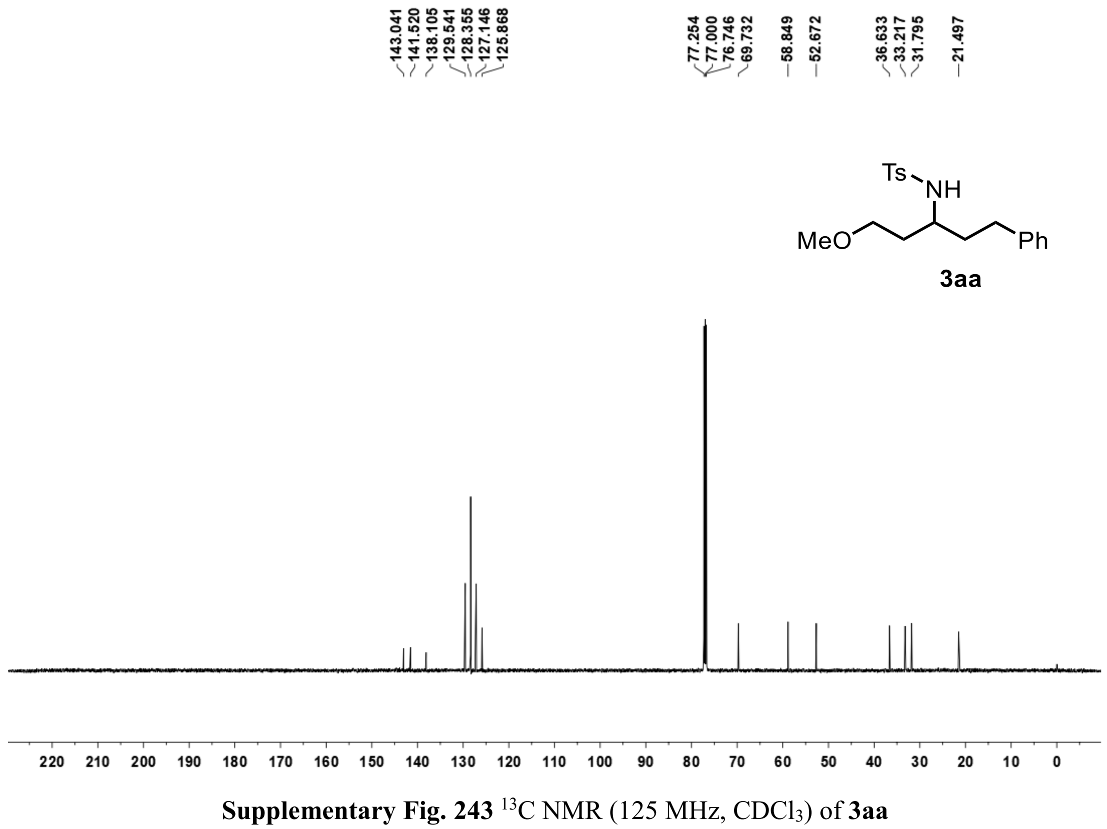

Supplementary Fig. 243 ^{13}C NMR (125 MHz, $CDCl_3$) of **3aa**

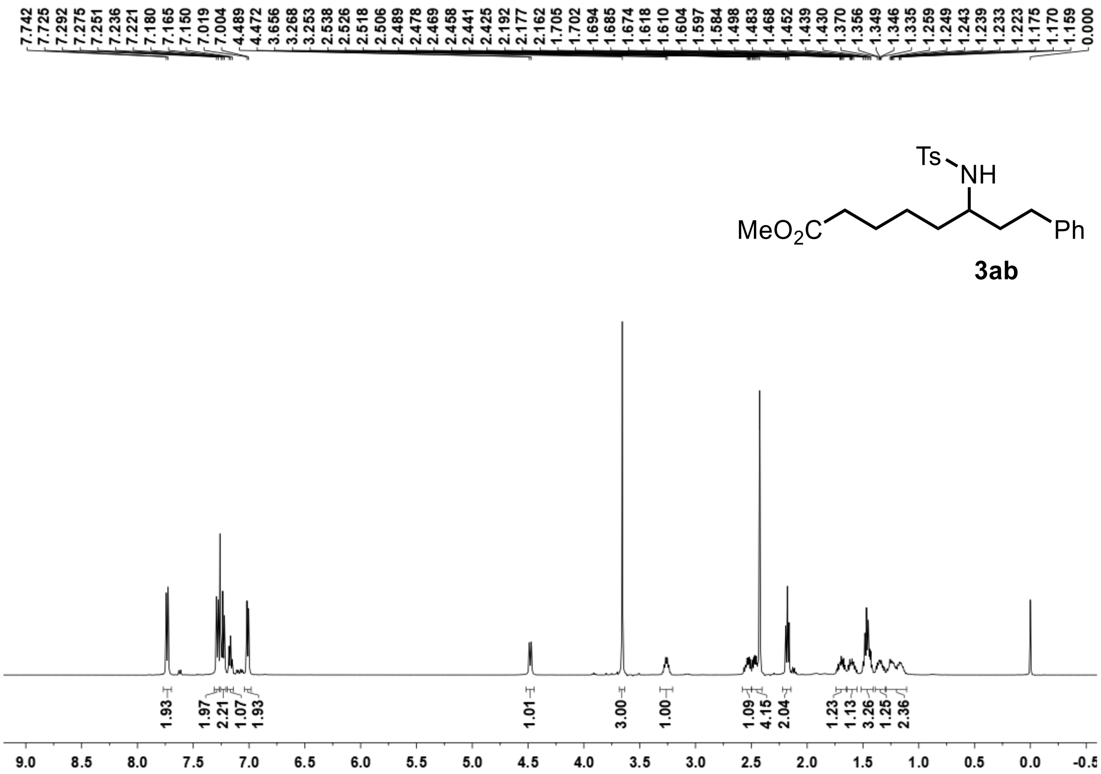

Supplementary Fig. 244 ^{1}H NMR (500 MHz, $CDCl_3$) of **3ab**

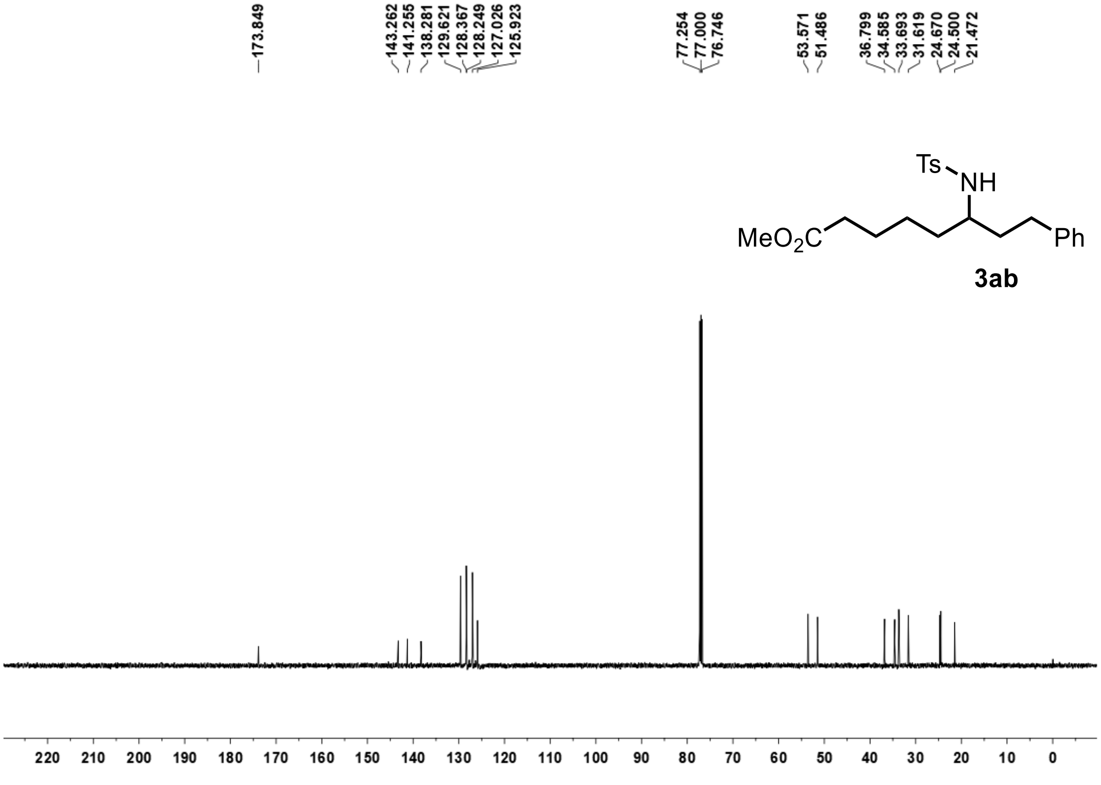

Supplementary Fig. 245 ^{13}C NMR (125 MHz, $CDCl_3$) of **3ab**

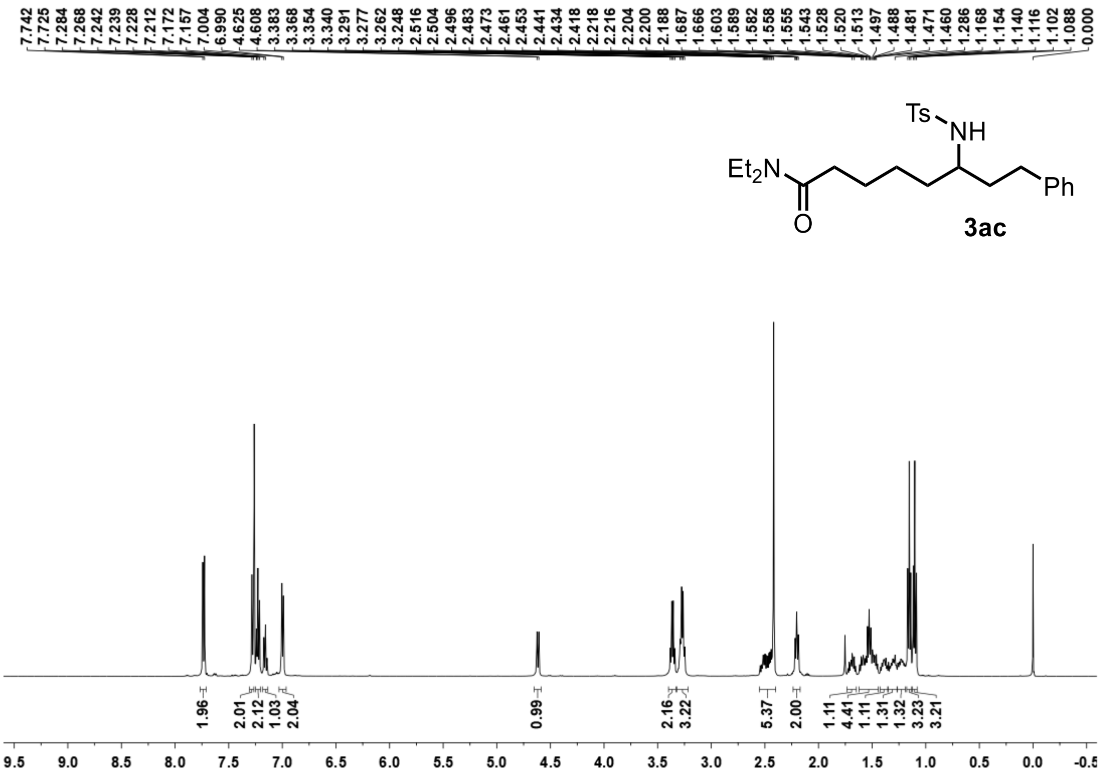

Supplementary Fig. 246 ^{1}H NMR (500 MHz, $CDCl_3$) of **3ac**

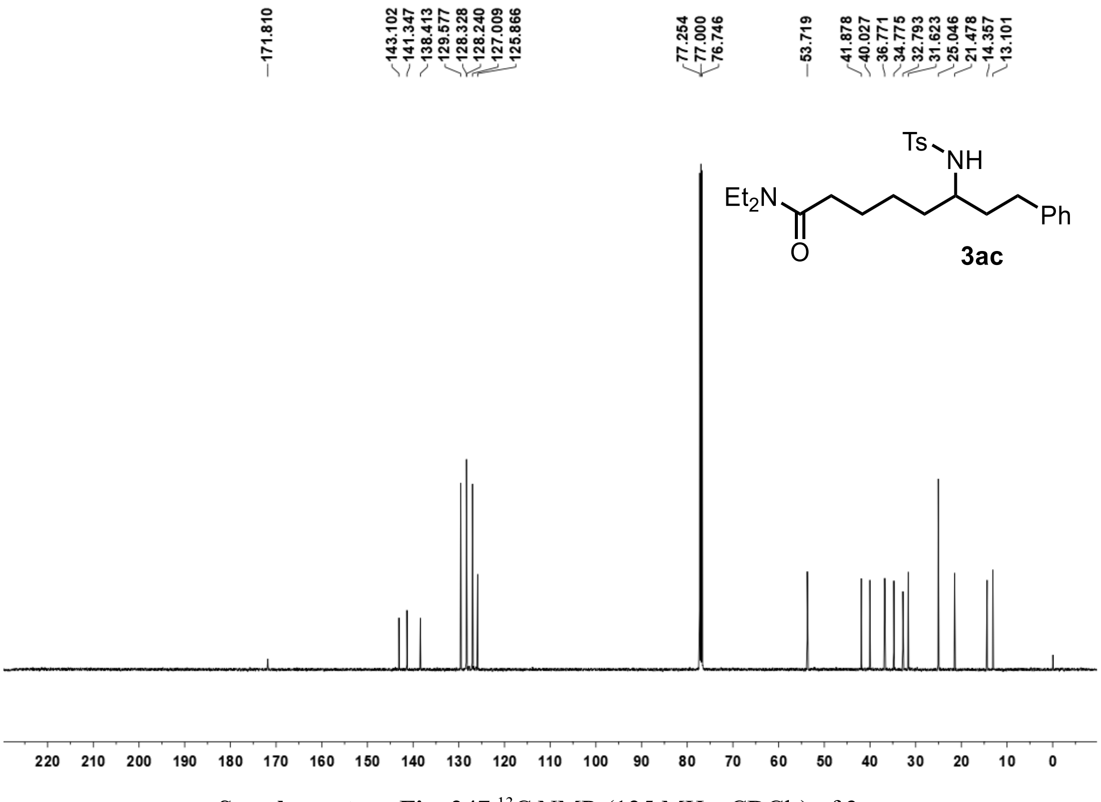

Supplementary Fig. 247 ^{13}C NMR (125 MHz, $CDCl_3$) of **3ac**

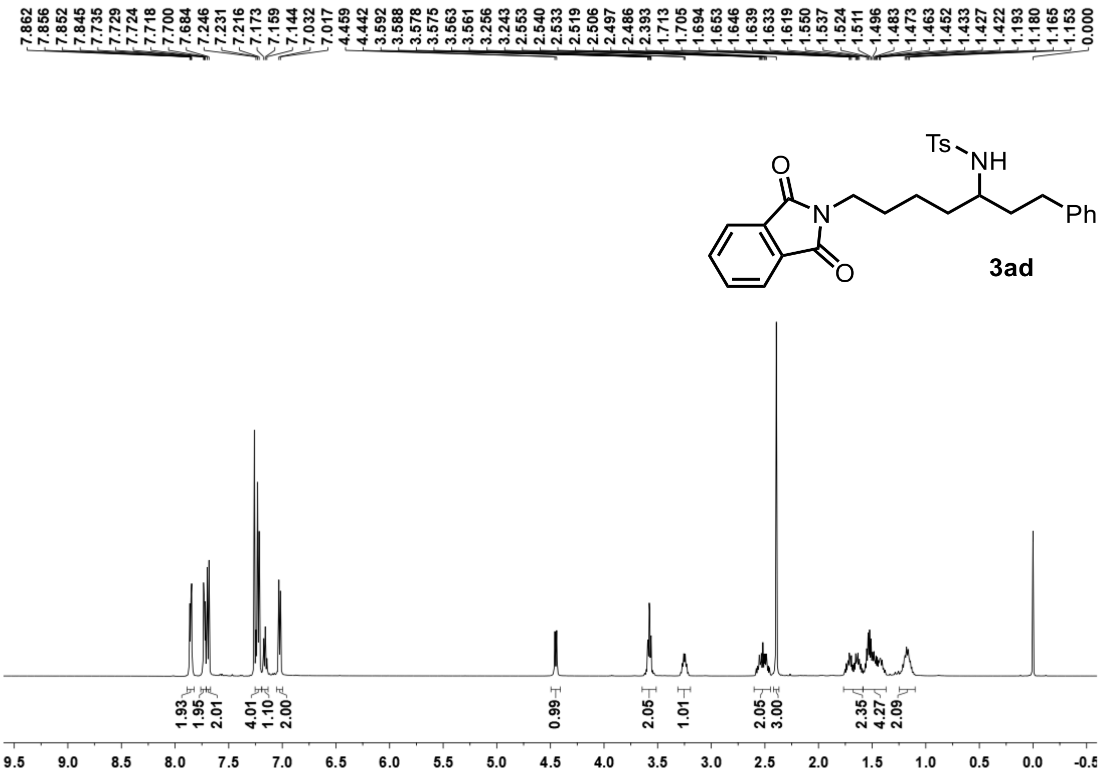

Supplementary Fig. 248 ^{1}H NMR (500 MHz, $CDCl_3$) of **3ad**

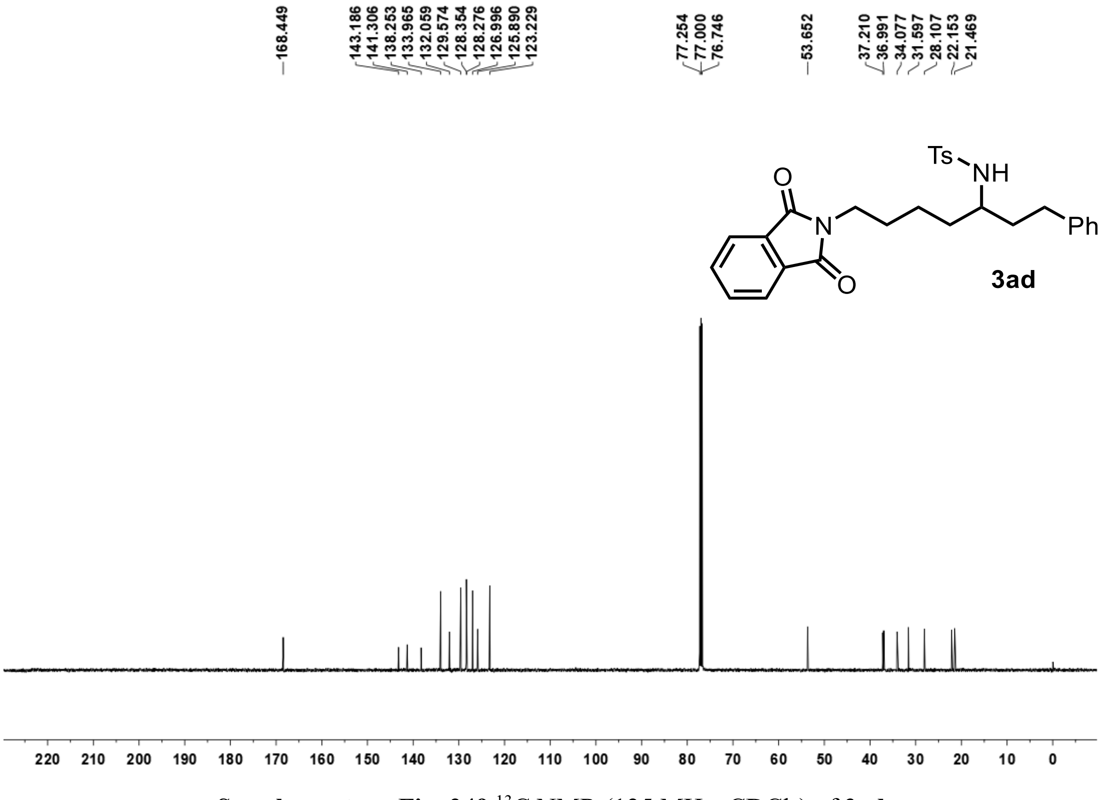

Supplementary Fig. 249 ^{13}C NMR (125 MHz, $CDCl_3$) of **3ad**

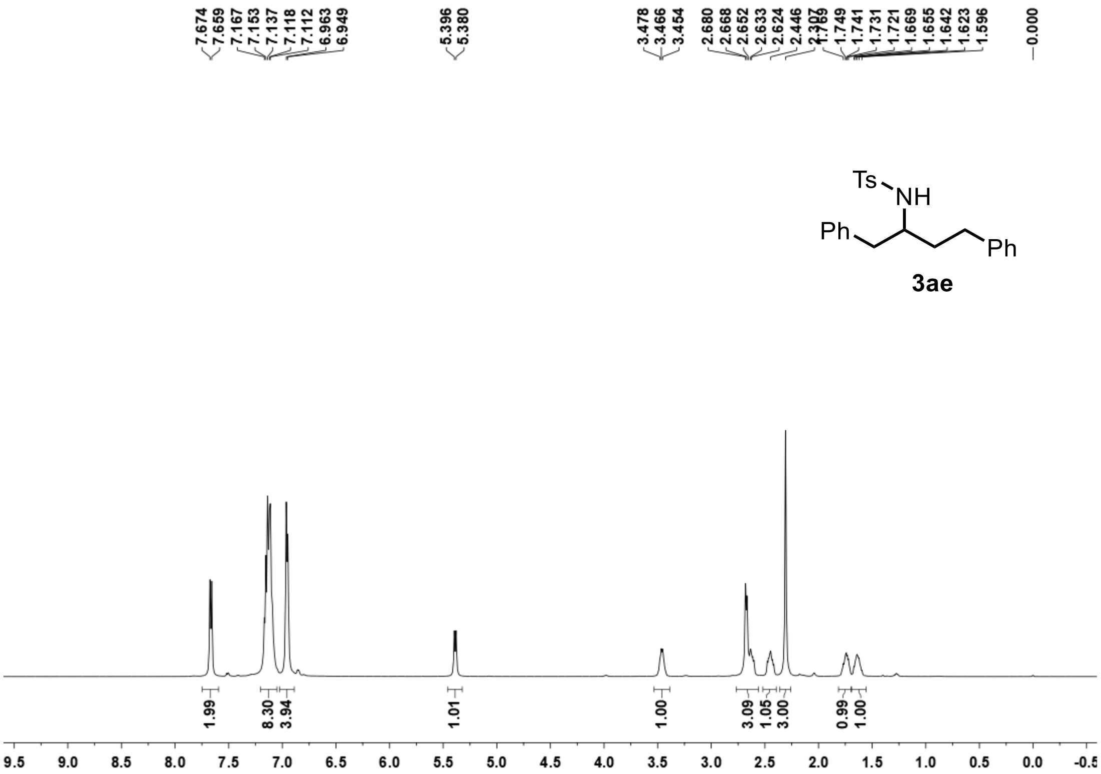

Supplementary Fig. 250 ^{1}H NMR (500 MHz, $CDCl_3$) of **3ae**

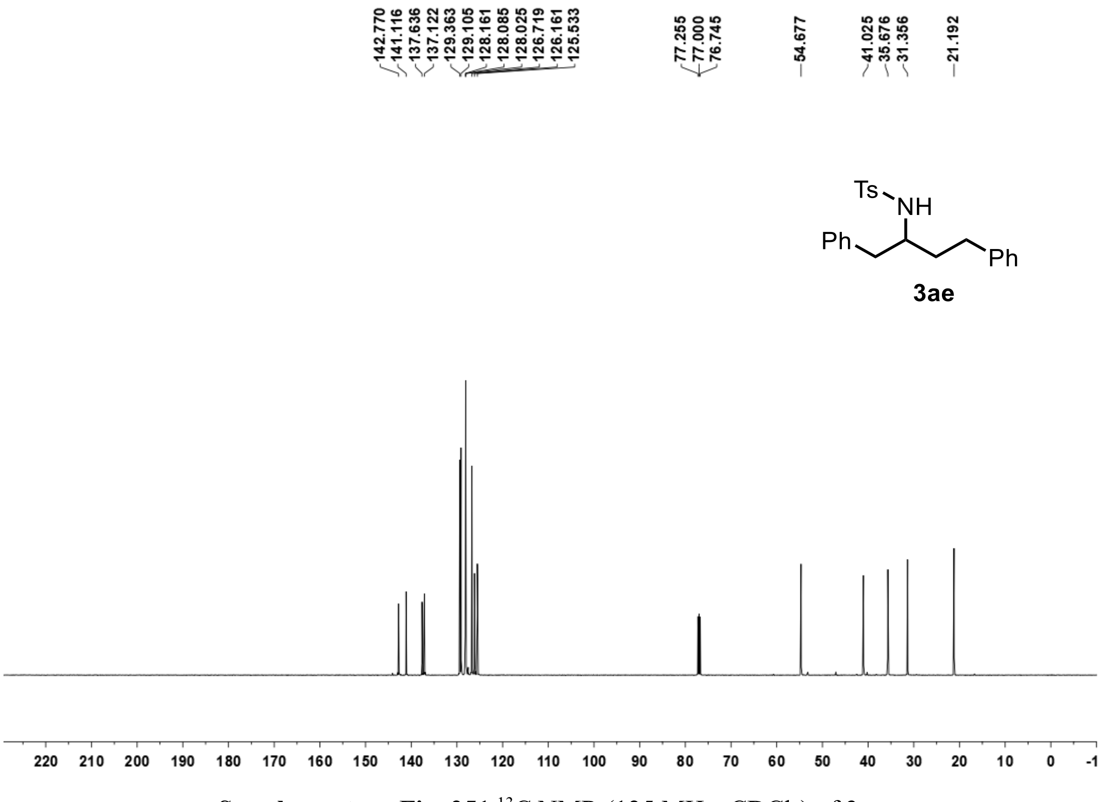

Supplementary Fig. 251 ^{13}C NMR (125 MHz, $CDCl_3$) of **3ae**

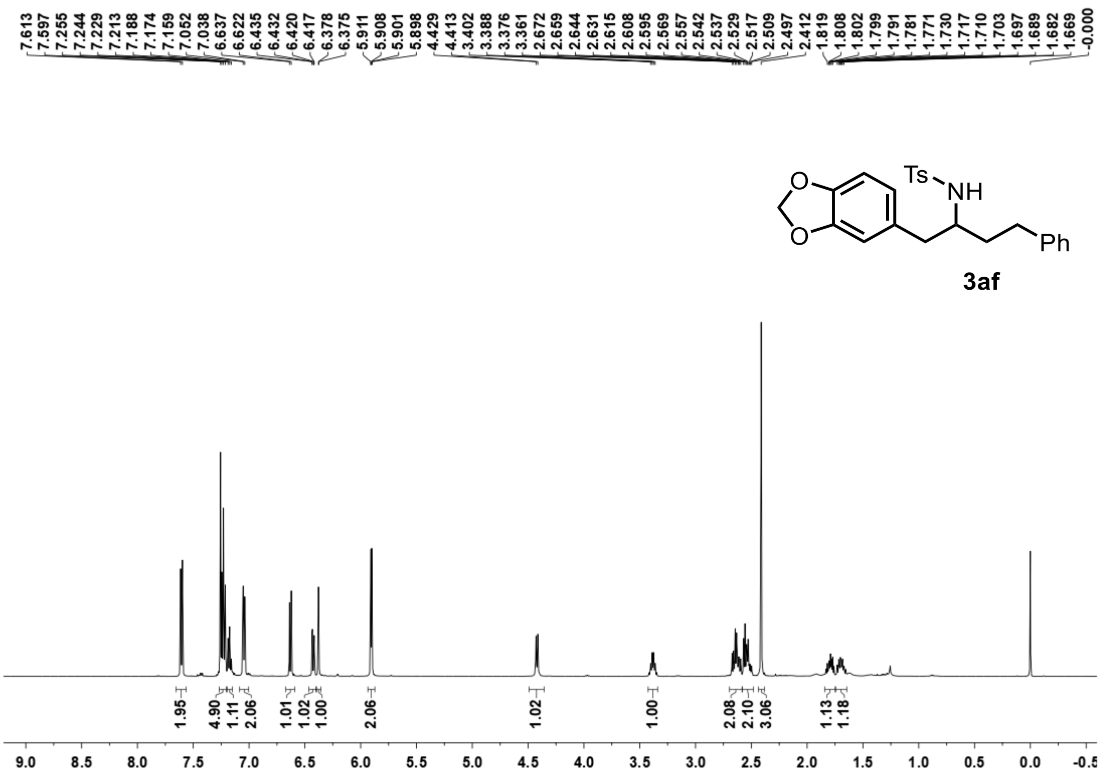

Supplementary Fig. 252 ^{1}H NMR (500 MHz, $CDCl_3$) of **3af**

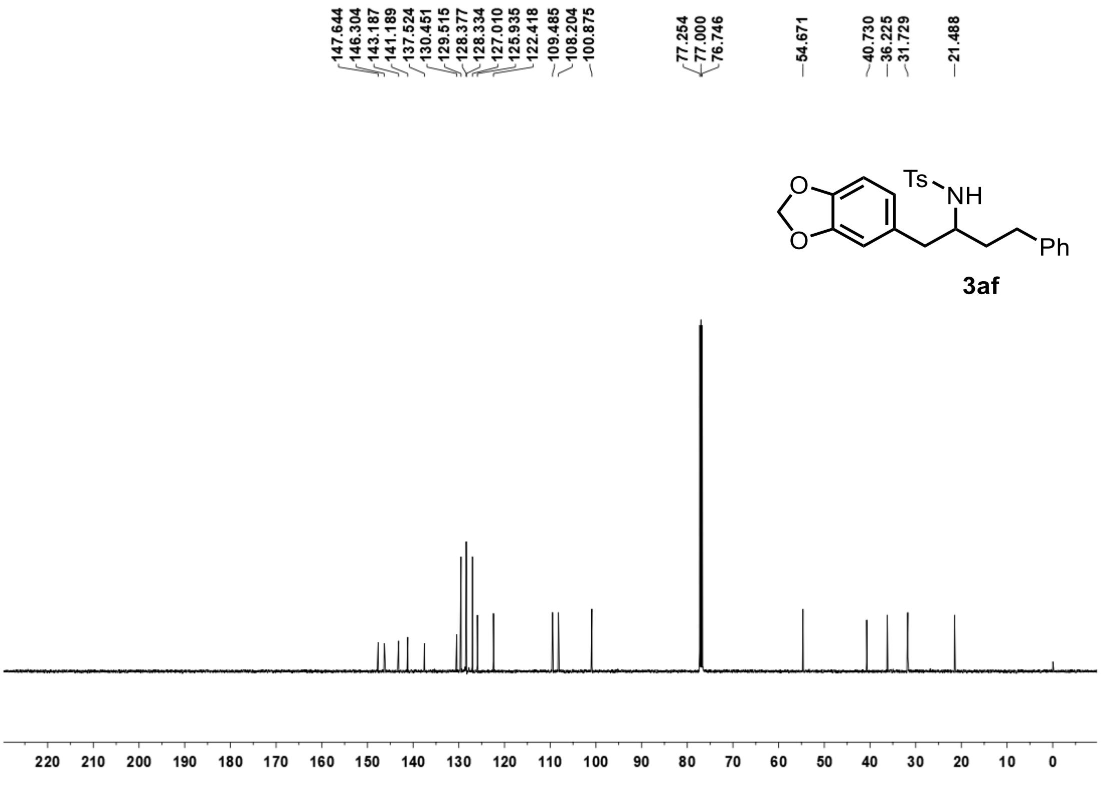

Supplementary Fig. 253 ^{13}C NMR (125 MHz, $CDCl_3$) of **3af**

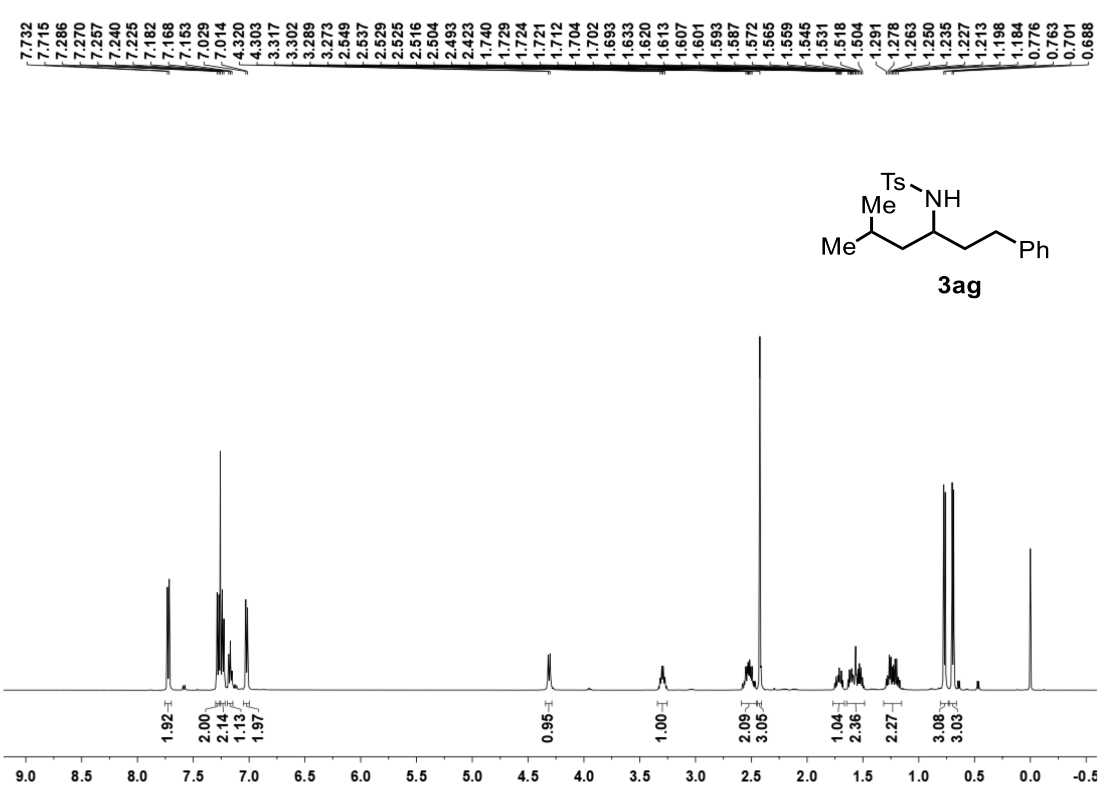

Supplementary Fig. 254 ^{1}H NMR (500 MHz, $CDCl_3$) of **3ag**

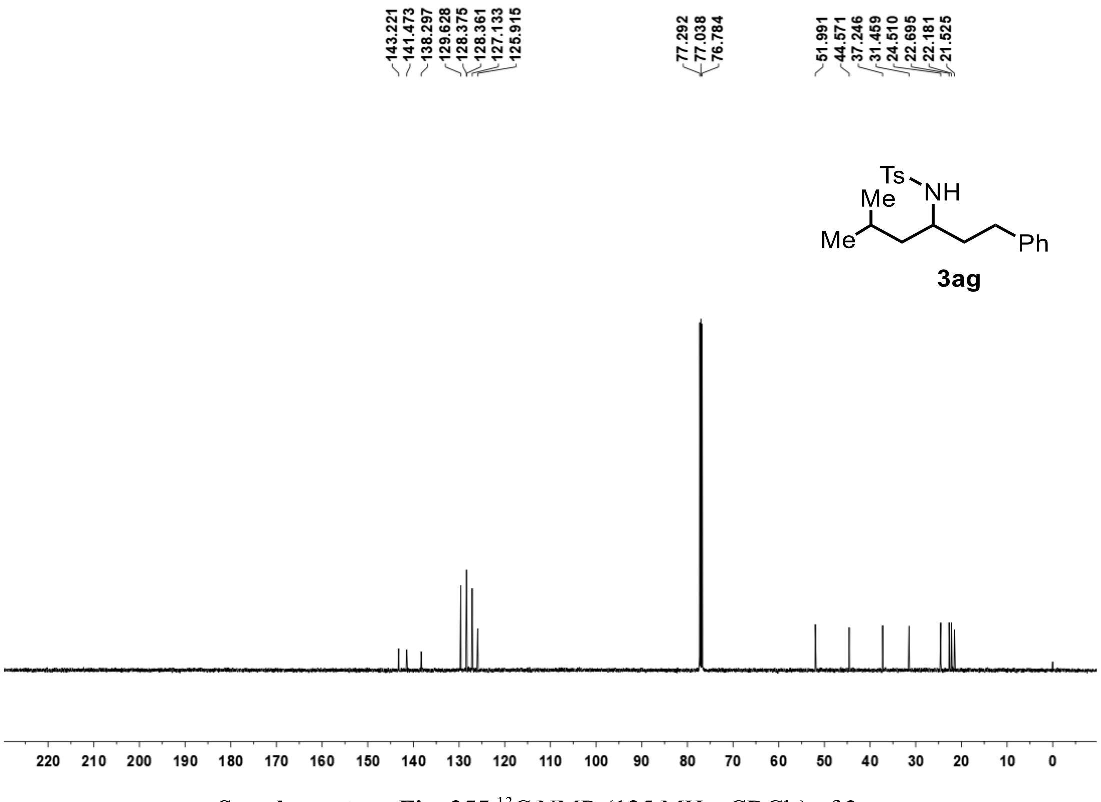

Supplementary Fig. 255 ^{13}C NMR (125 MHz, $CDCl_3$) of **3ag**

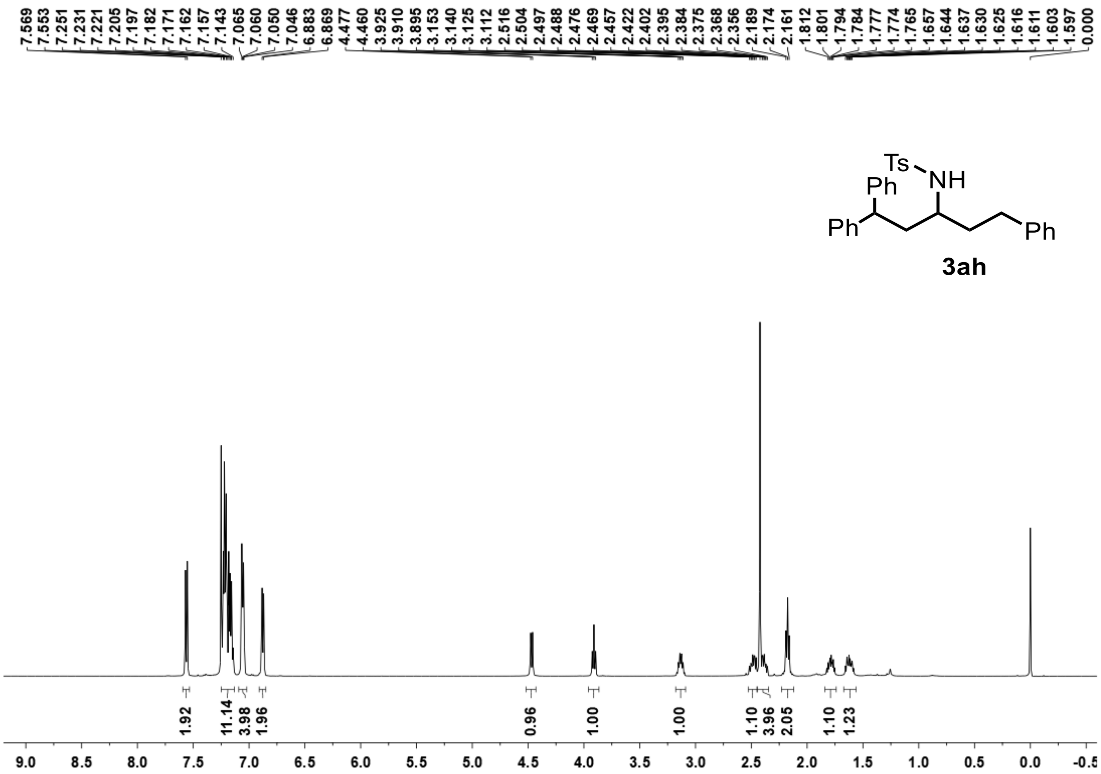

Supplementary Fig. 256 ^{1}H NMR (500 MHz, $CDCl_3$) of **3ah**

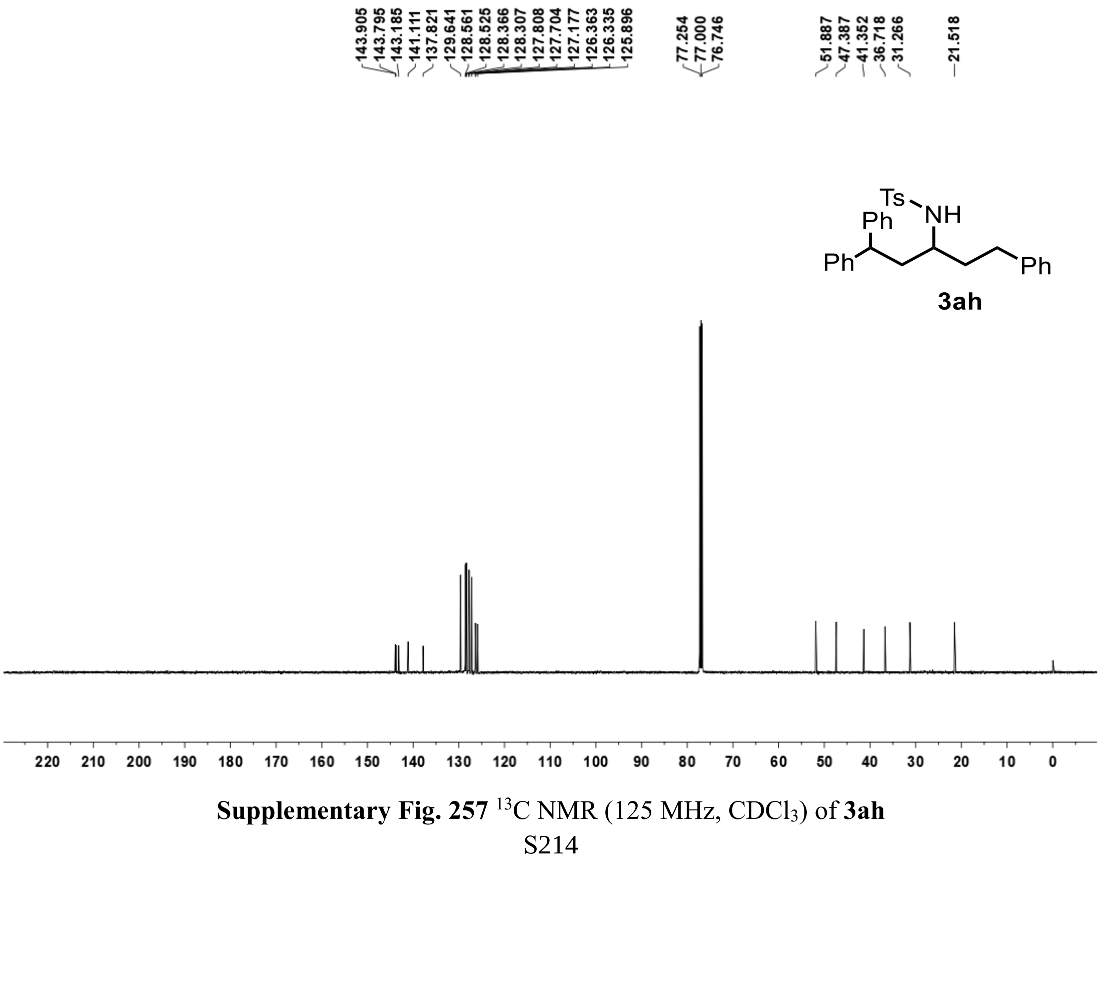

Supplementary Fig. 257 ^{13}C NMR (125 MHz, $CDCl_3$) of **3ah**

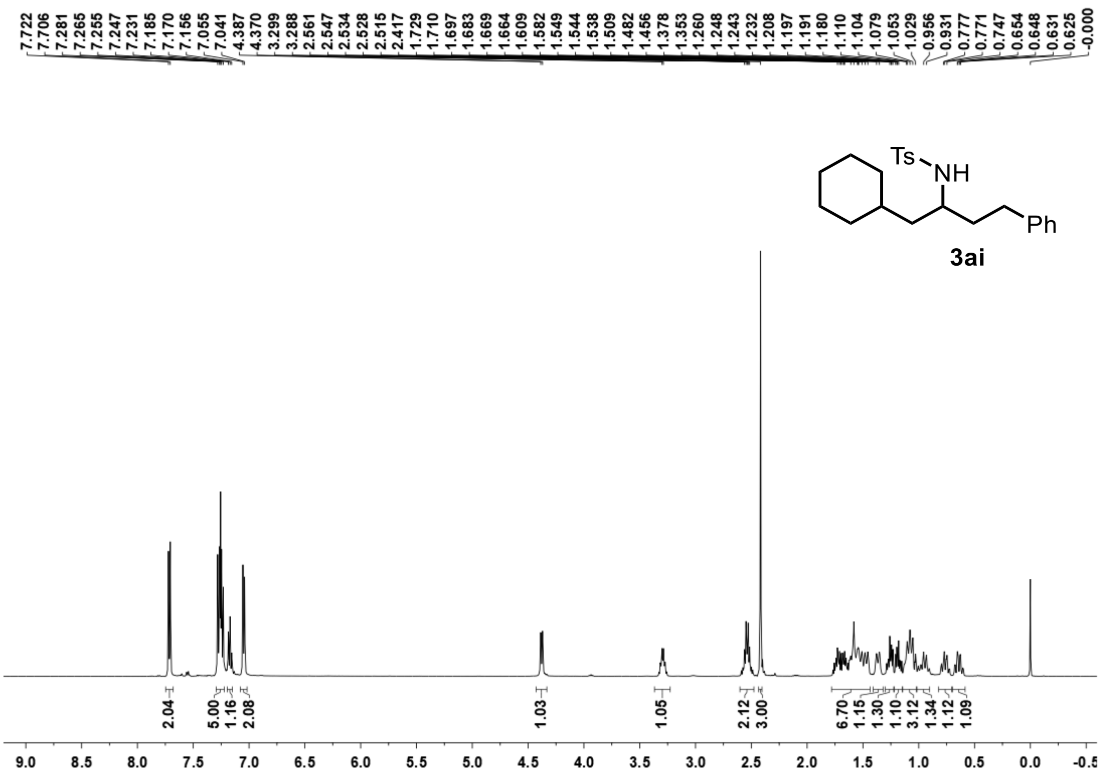

Supplementary Fig. 258 ^{1}H NMR (500 MHz, $CDCl_3$) of **3ai**

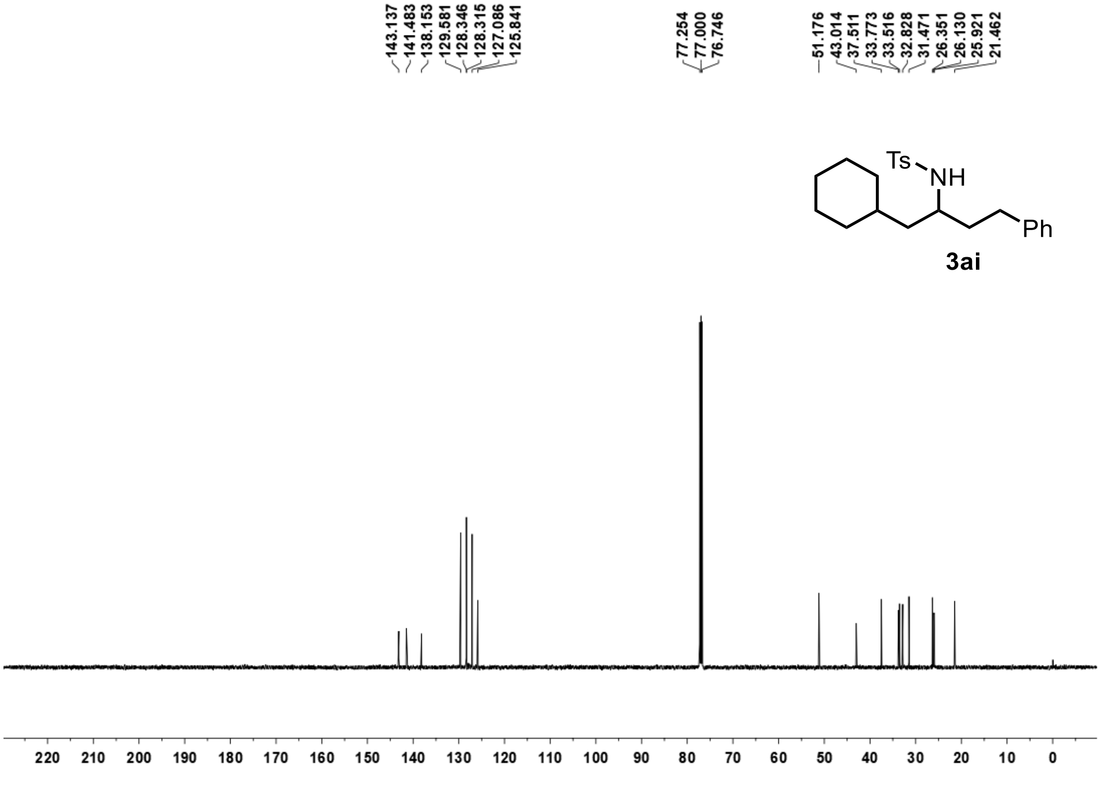

Supplementary Fig. 259 ^{13}C NMR (125 MHz, $CDCl_3$) of **3ai**

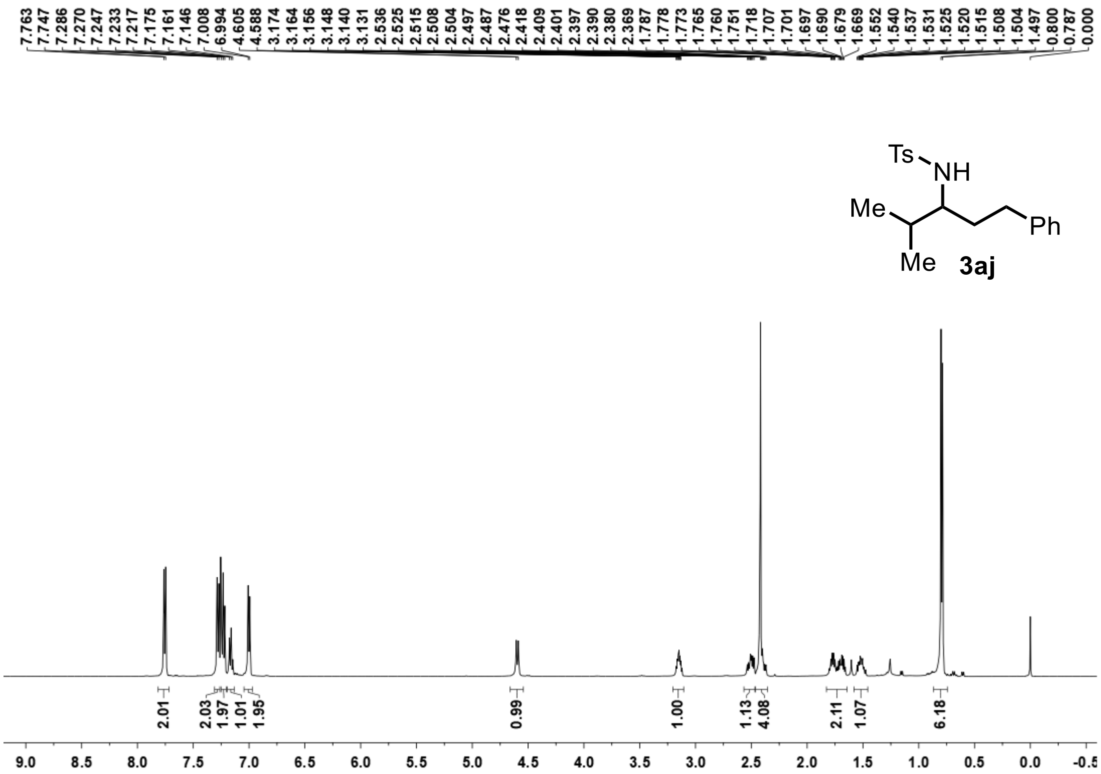

Supplementary Fig. 260 ^{1}H NMR (500 MHz, $CDCl_3$) of **3aj**

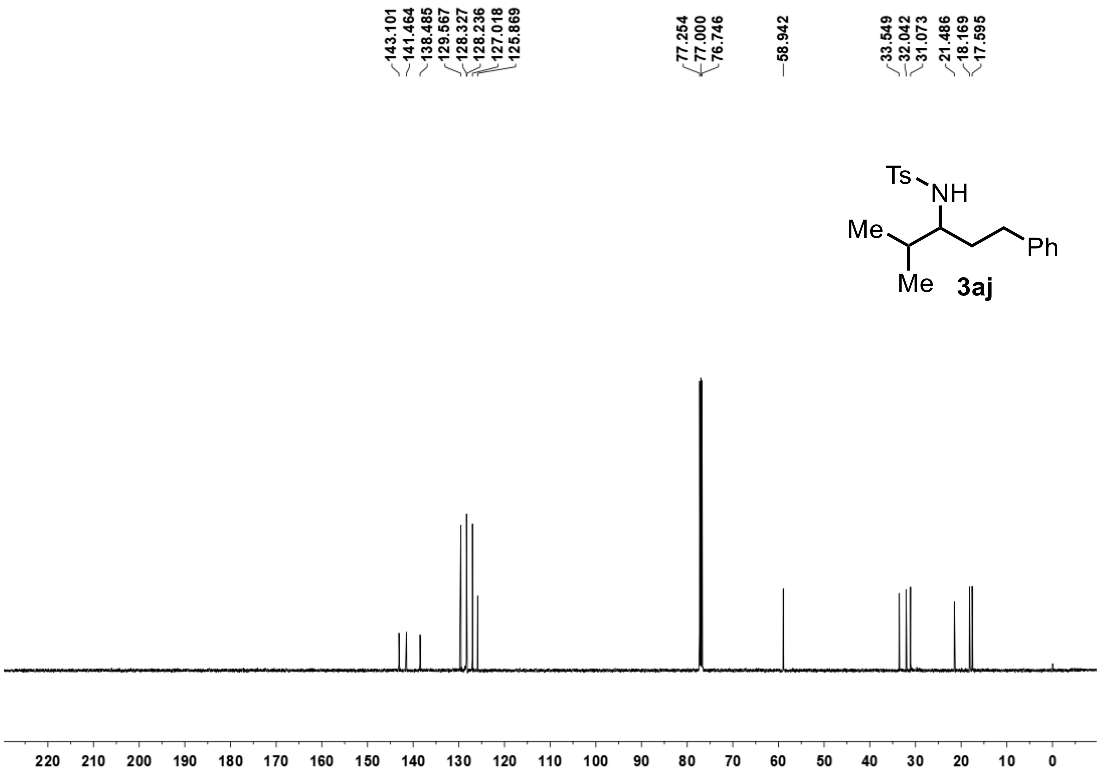

Supplementary Fig. 261 ^{13}C NMR (125 MHz, $CDCl_3$) of **3aj**

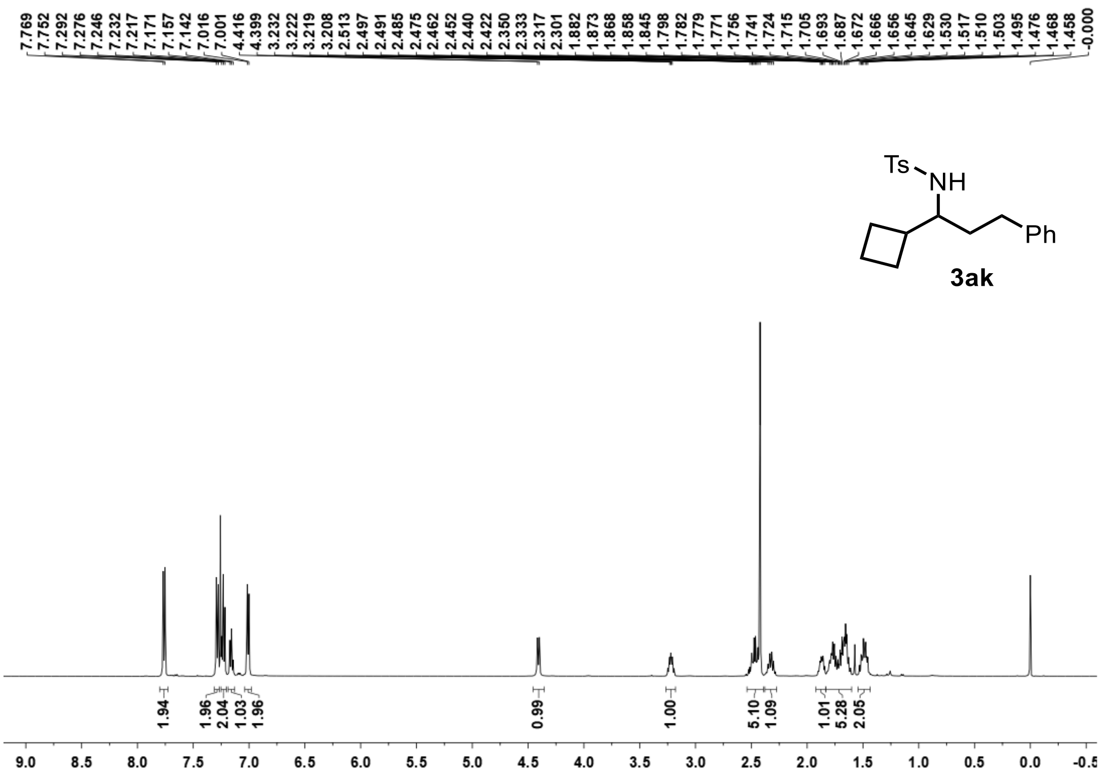

Supplementary Fig. 262 ^{1}H NMR (500 MHz, $CDCl_3$) of **3ak**

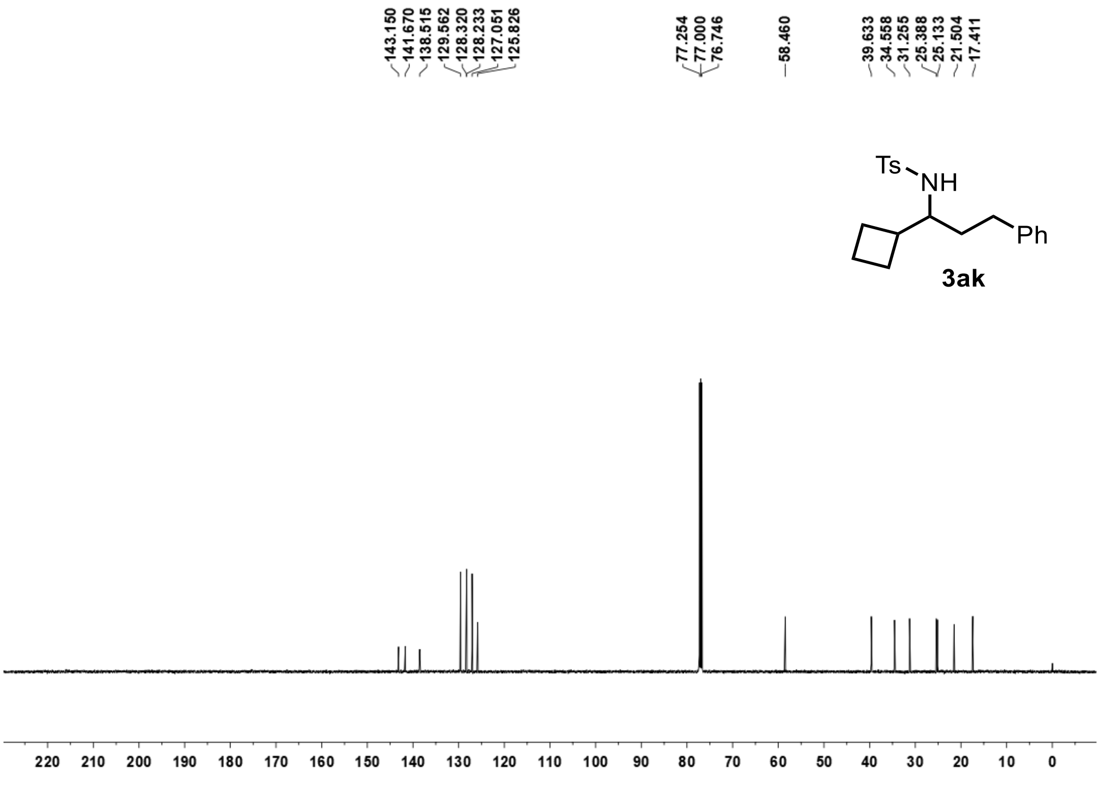

Supplementary Fig. 263 ^{13}C NMR (125 MHz, $CDCl_3$) of **3ak**

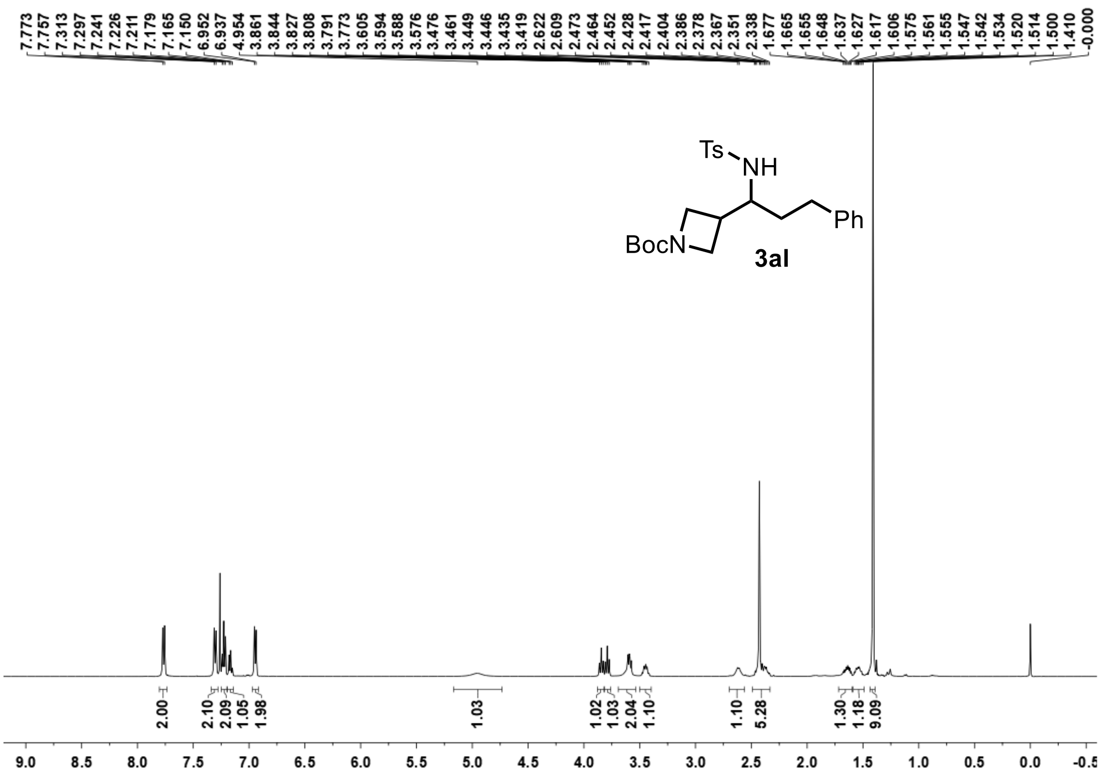

Supplementary Fig. 264 ^{1}H NMR (500 MHz, $CDCl_3$) of **3al**

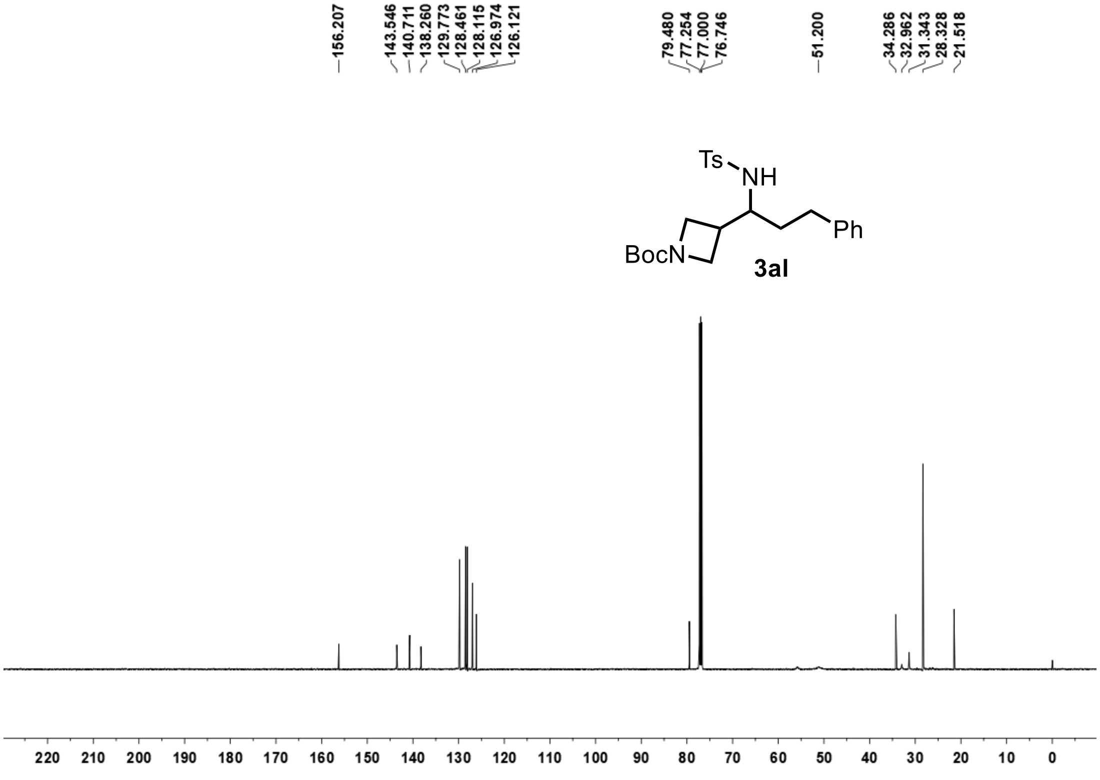

Supplementary Fig. 265 ^{13}C NMR (125 MHz, $CDCl_3$) of **3al**

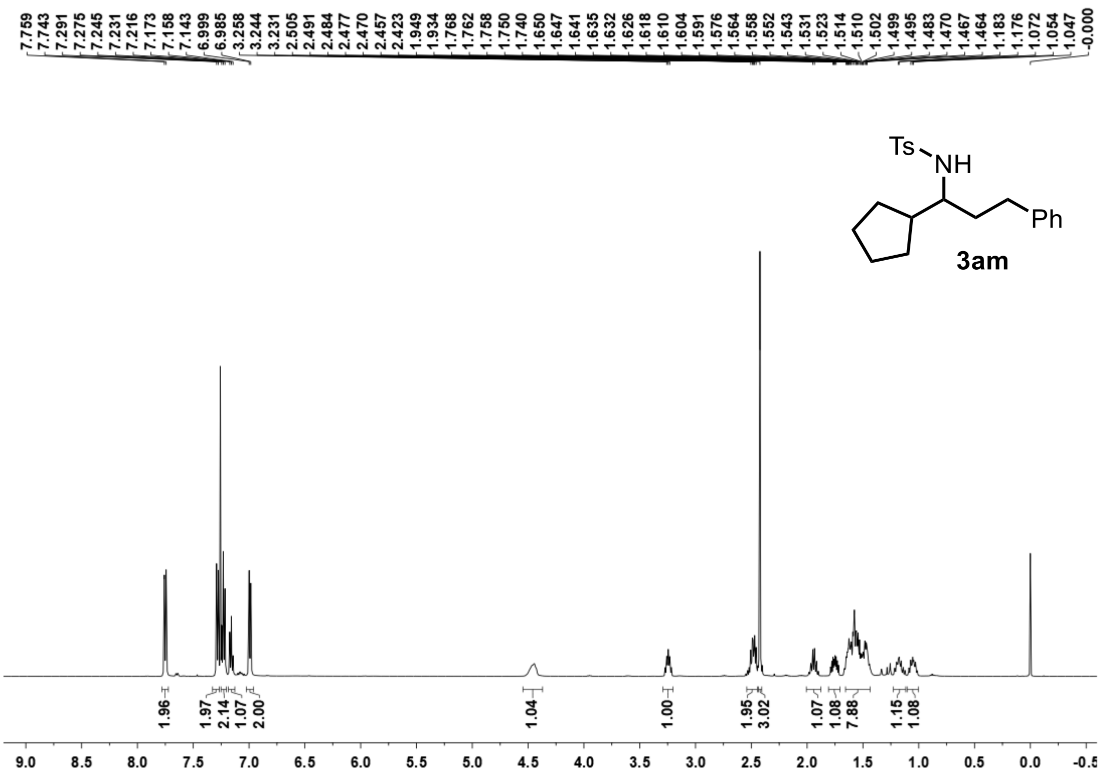

Supplementary Fig. 266 ^{1}H NMR (500 MHz, $CDCl_3$) of **3am**

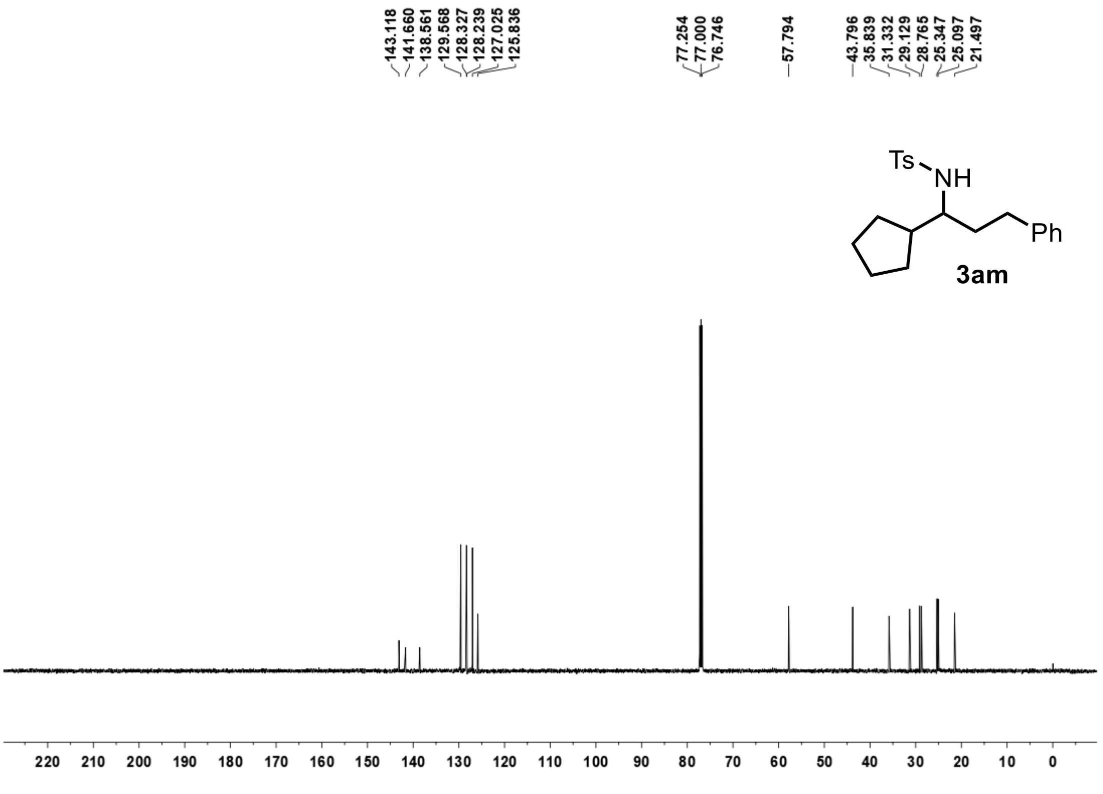

Supplementary Fig. 267 ^{13}C NMR (125 MHz, $CDCl_3$) of **3am**

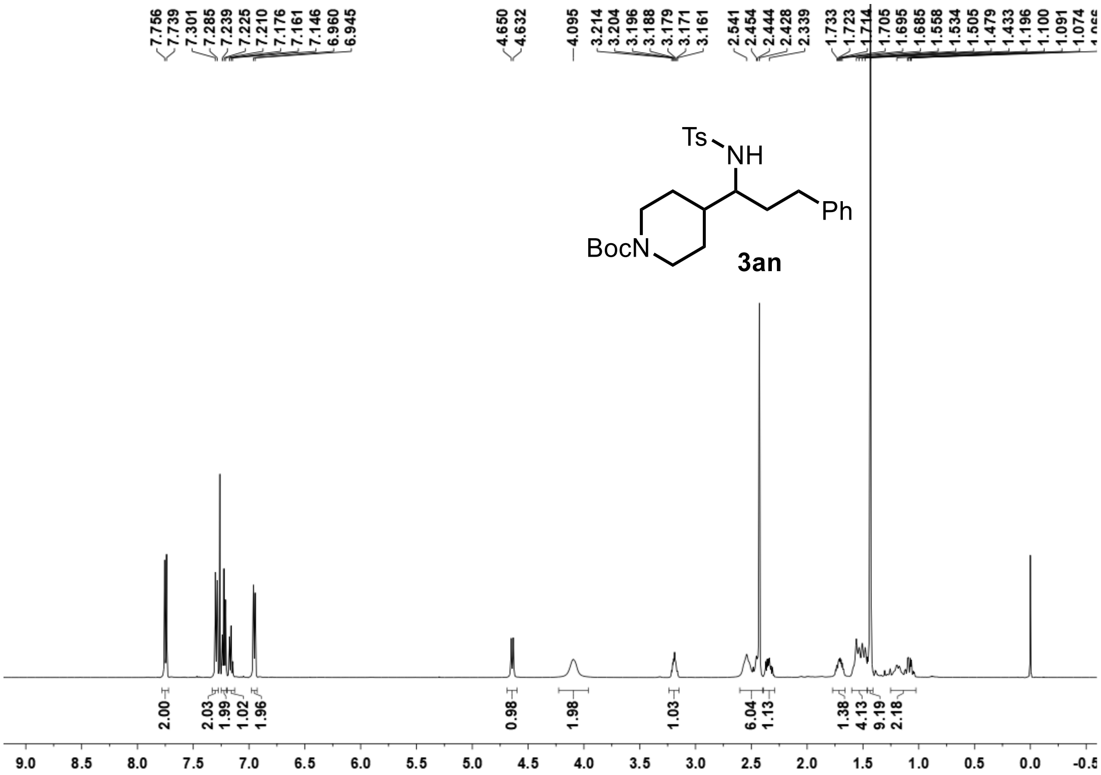

Supplementary Fig. 268 ^{1}H NMR (500 MHz, $CDCl_3$) of **3an**

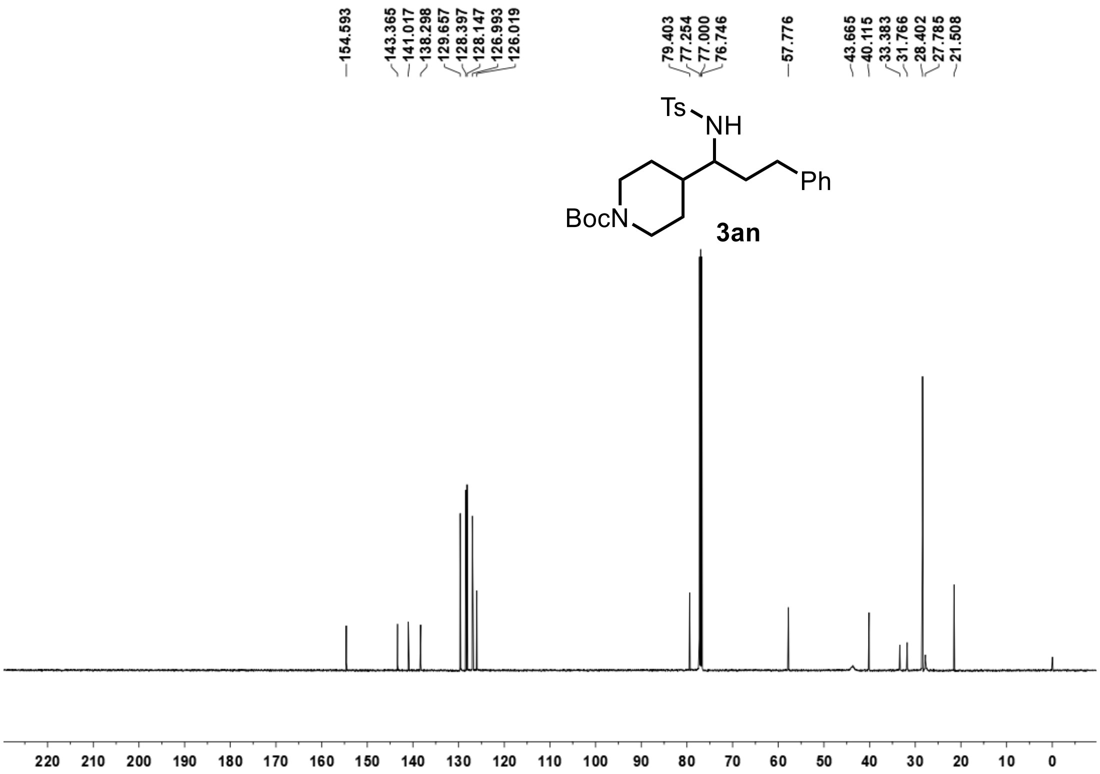

Supplementary Fig. 269 ^{13}C NMR (125 MHz, $CDCl_3$) of **3an**

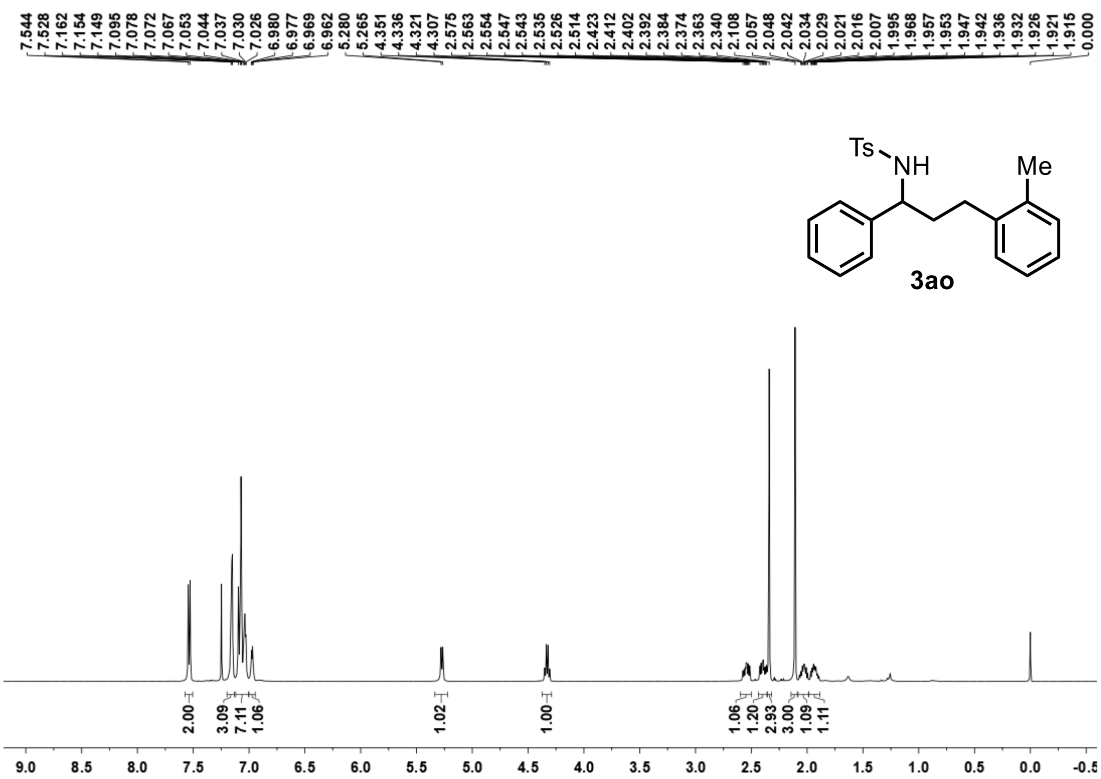

Supplementary Fig. 270 ^{1}H NMR (500 MHz, $CDCl_3$) of **3ao**

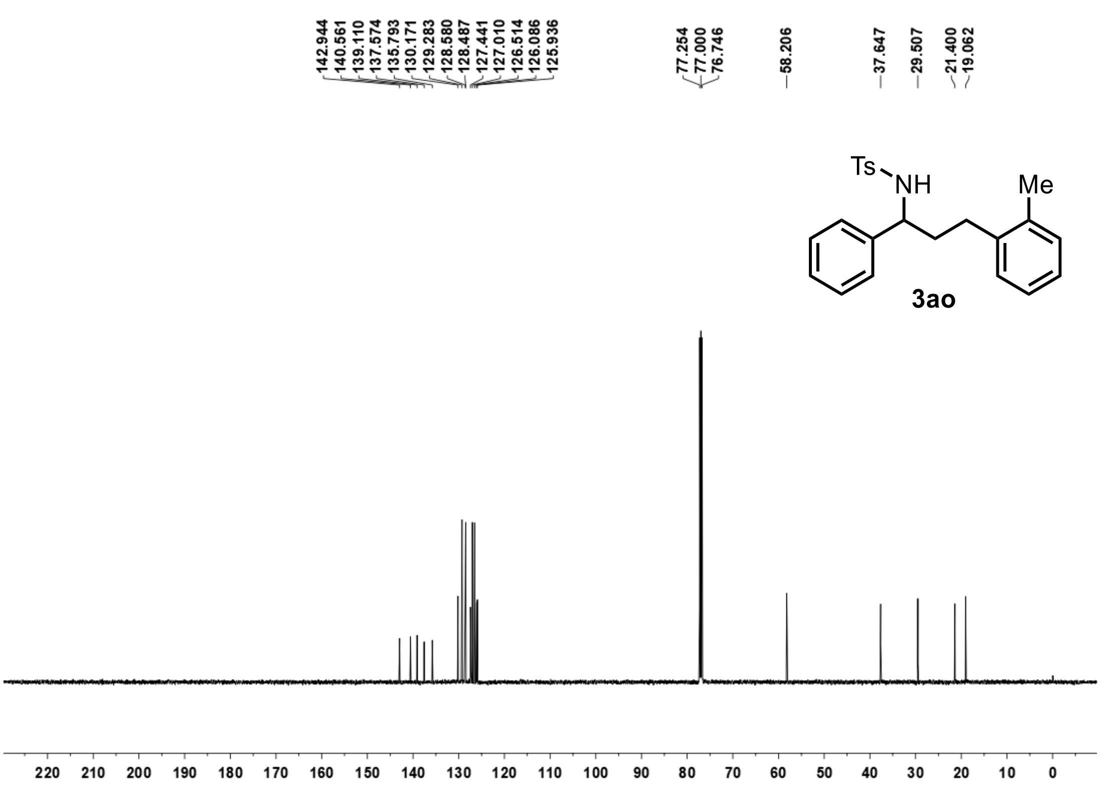

Supplementary Fig. 271 ^{13}C NMR (125 MHz, $CDCl_3$) of **3ao**

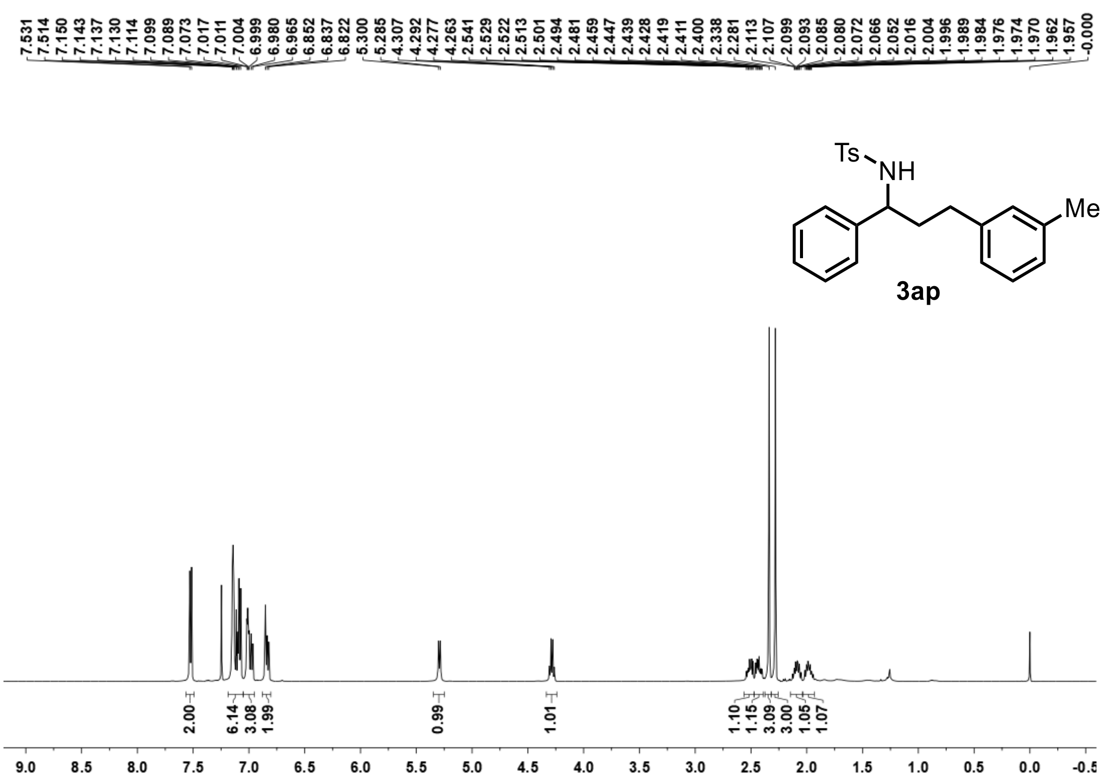

Supplementary Fig. 272 ^{1}H NMR (500 MHz, $CDCl_3$) of **3ap**

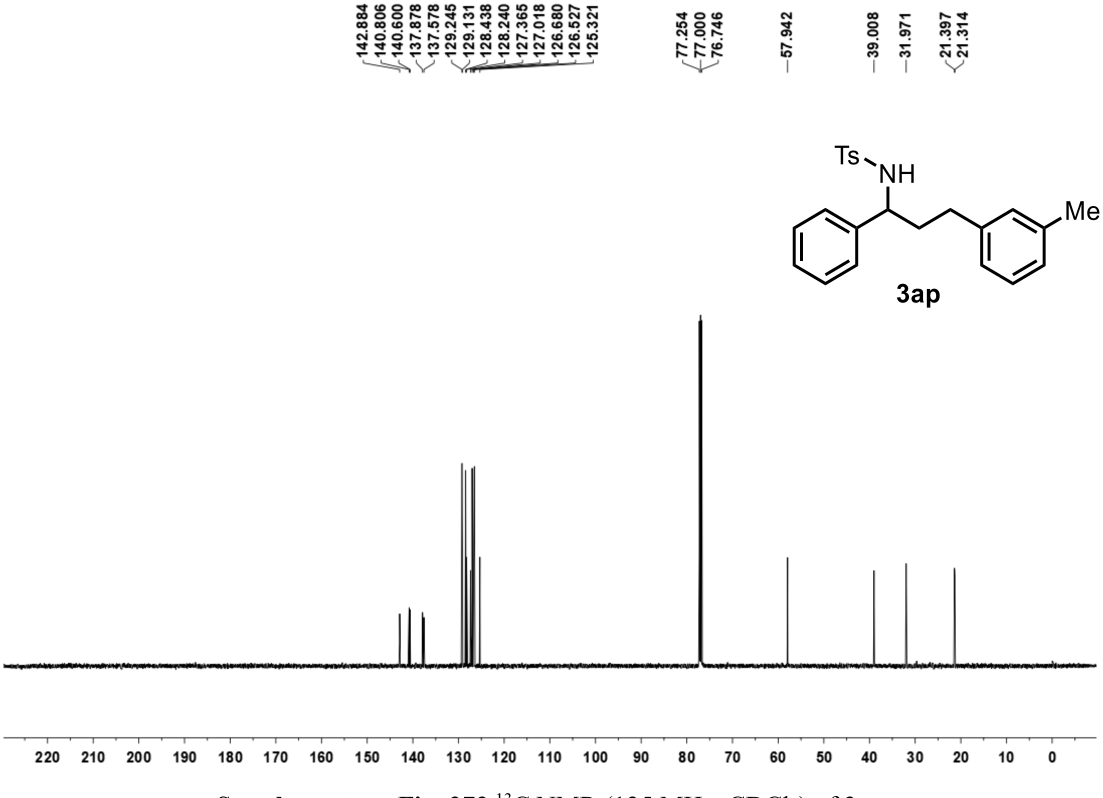

Supplementary Fig. 273 ^{13}C NMR (125 MHz, $CDCl_3$) of **3ap**

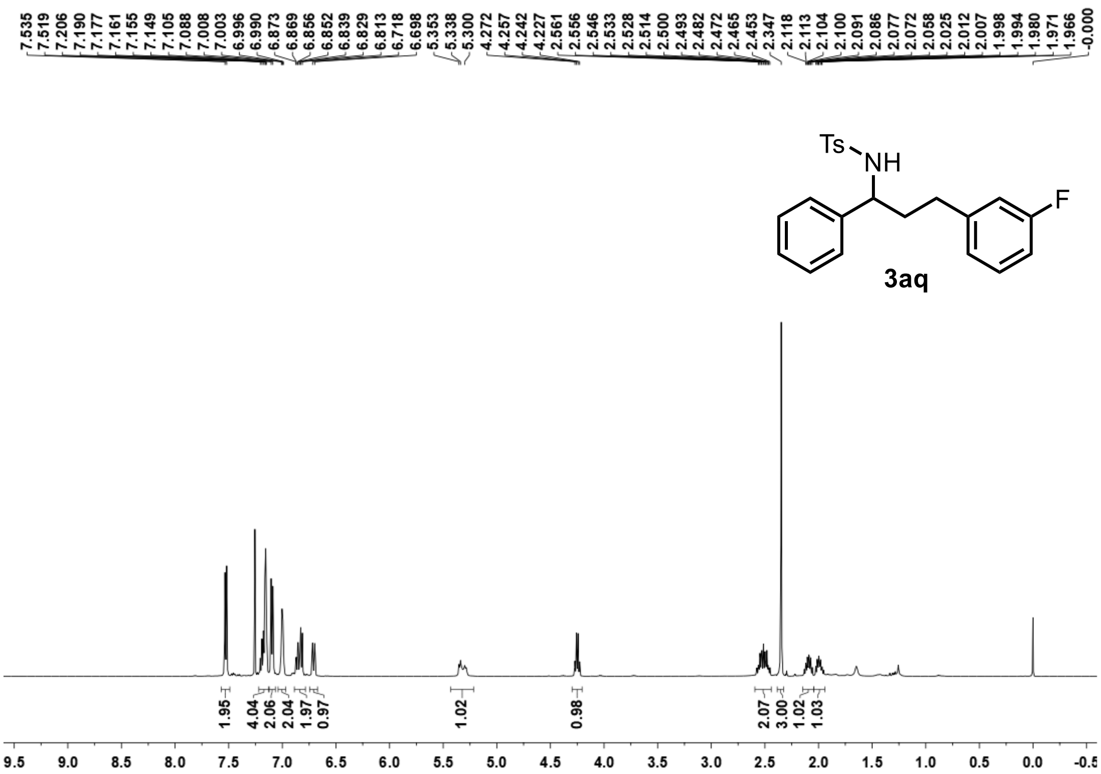

Supplementary Fig. 274 ^{1}H NMR (500 MHz, $CDCl_3$) of **3aq**

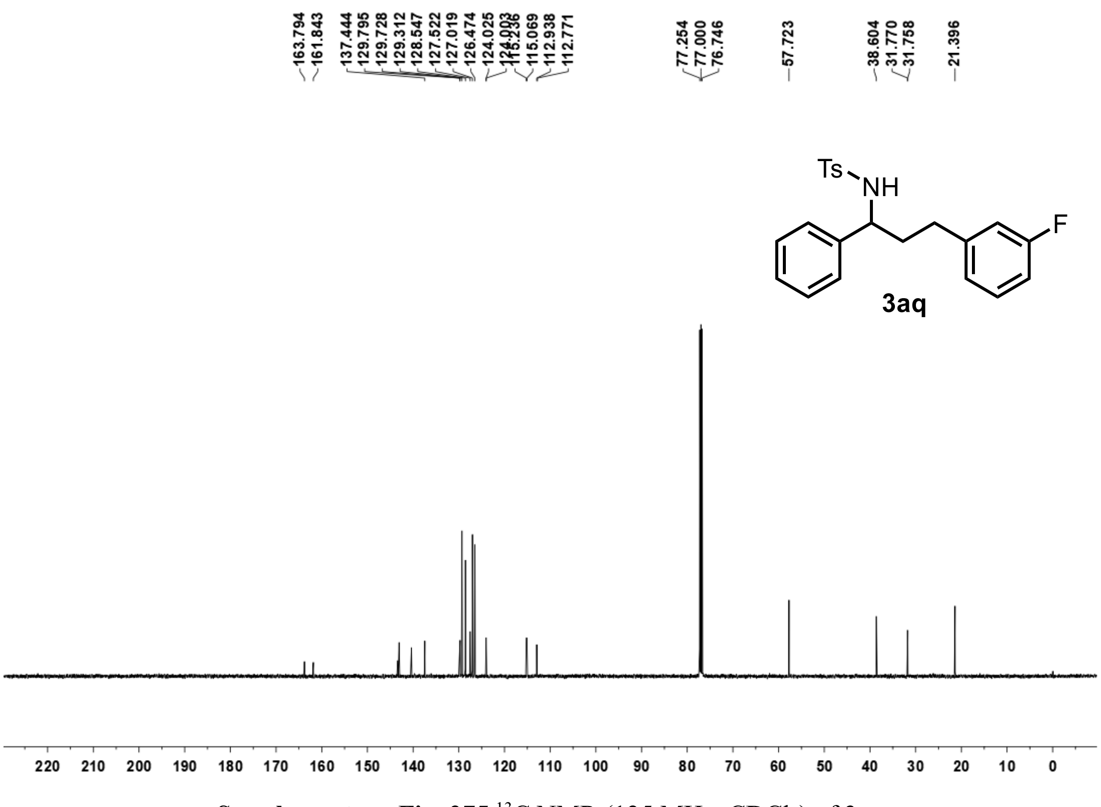

Supplementary Fig. 275 ^{13}C NMR (125 MHz, $CDCl_3$) of **3aq**

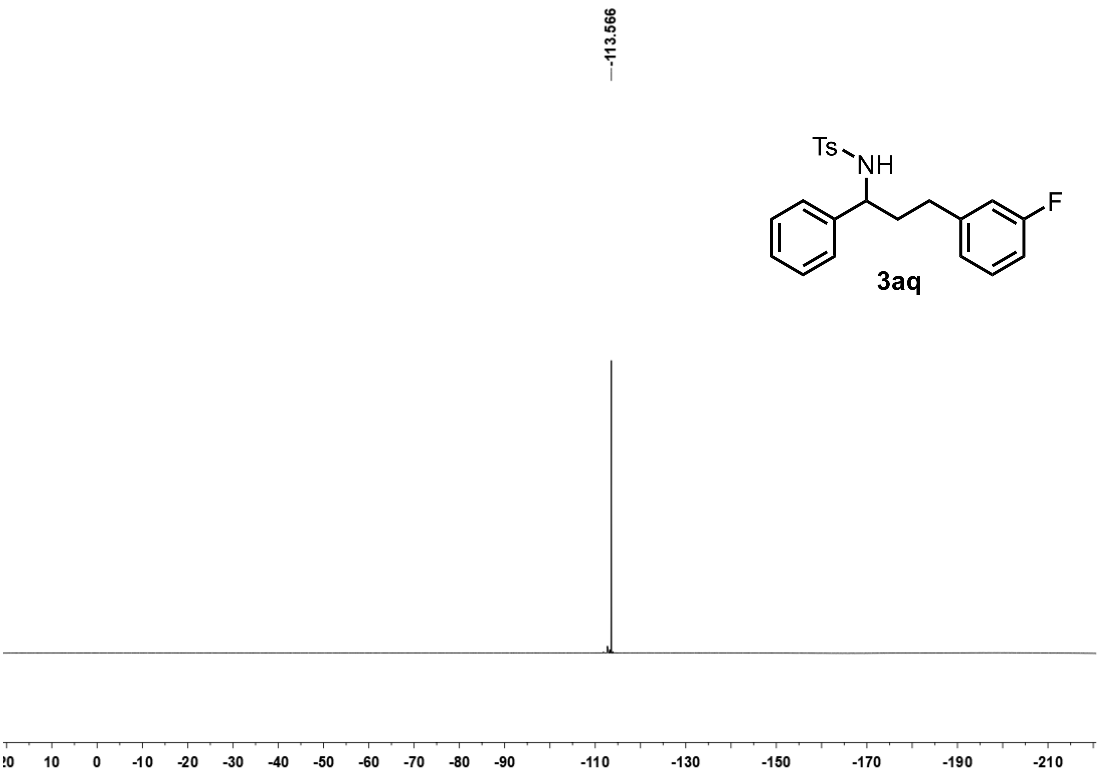

Supplementary Fig. 276 ^{19}F NMR (470 MHz, $CDCl_3$) of **3aq**

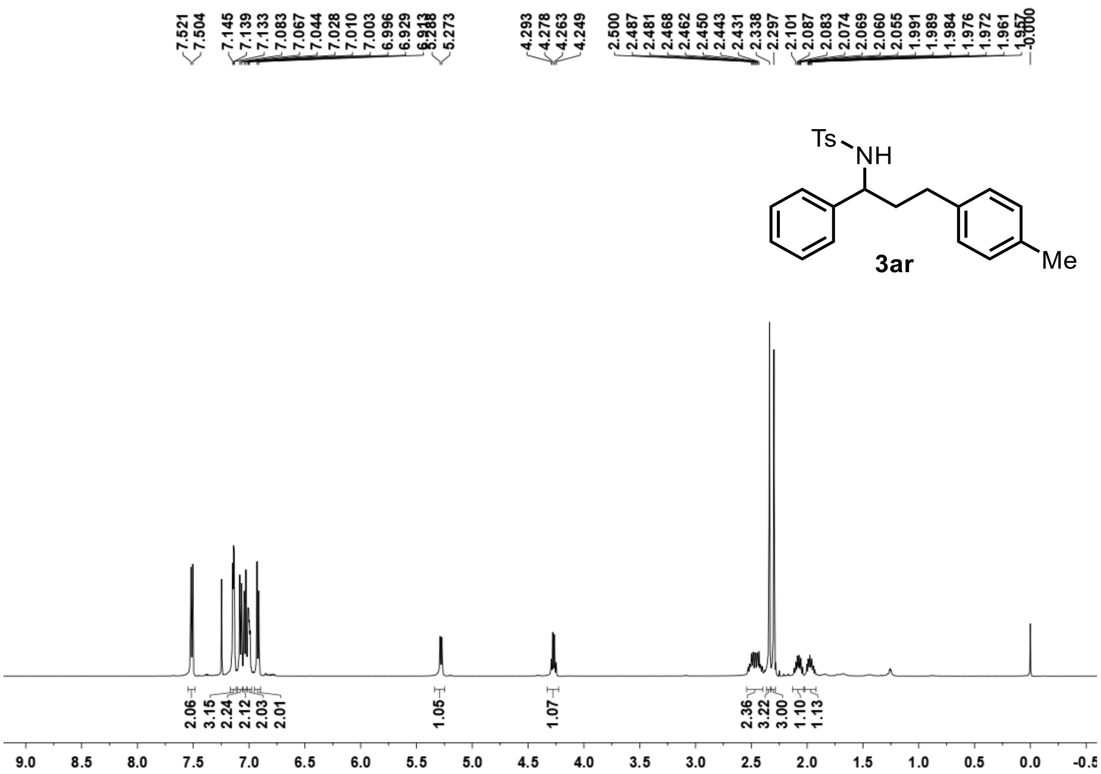

Supplementary Fig. 277 ^{1}H NMR (500 MHz, $CDCl_3$) of **3ar**

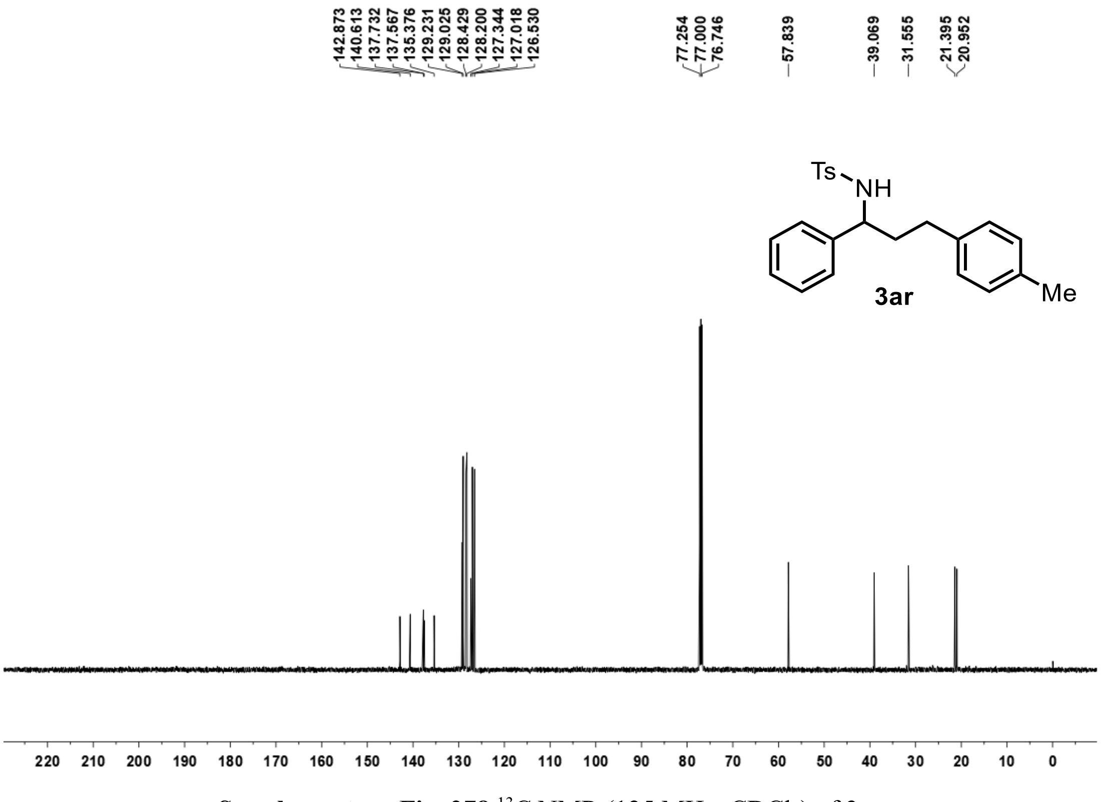

Supplementary Fig. 278 ^{13}C NMR (125 MHz, $CDCl_3$) of **3ar**

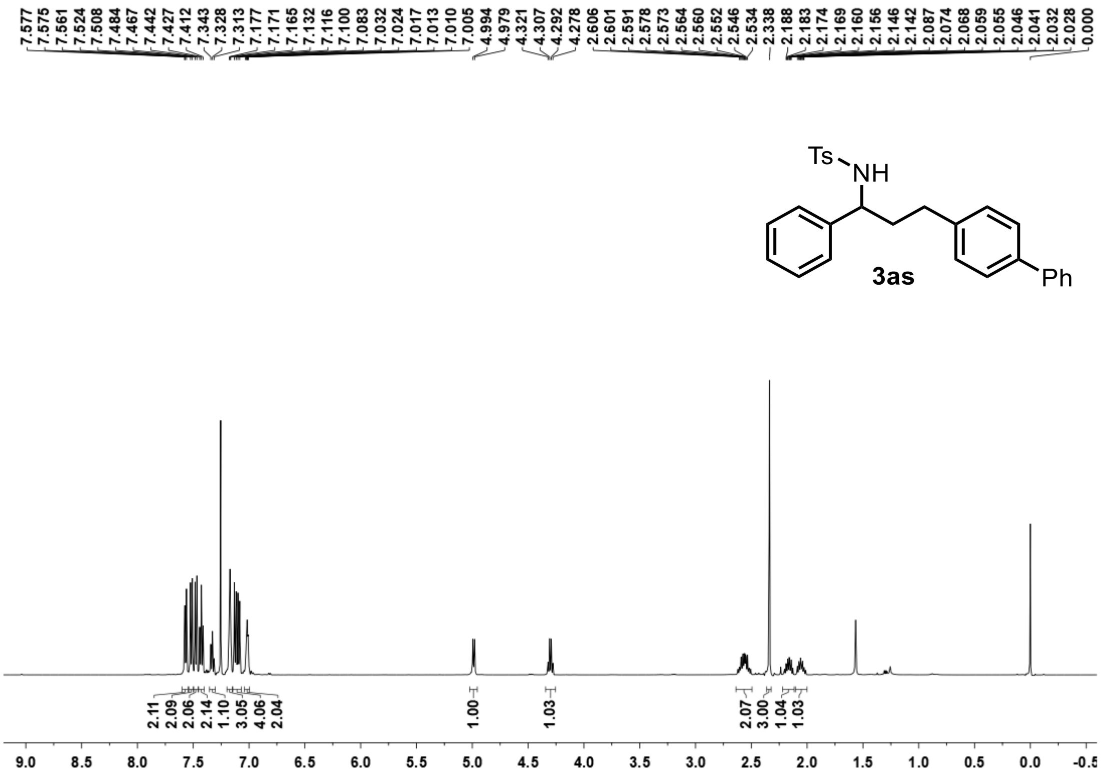

Supplementary Fig. 279 ^{1}H NMR (500 MHz, $CDCl_3$) of **3as**

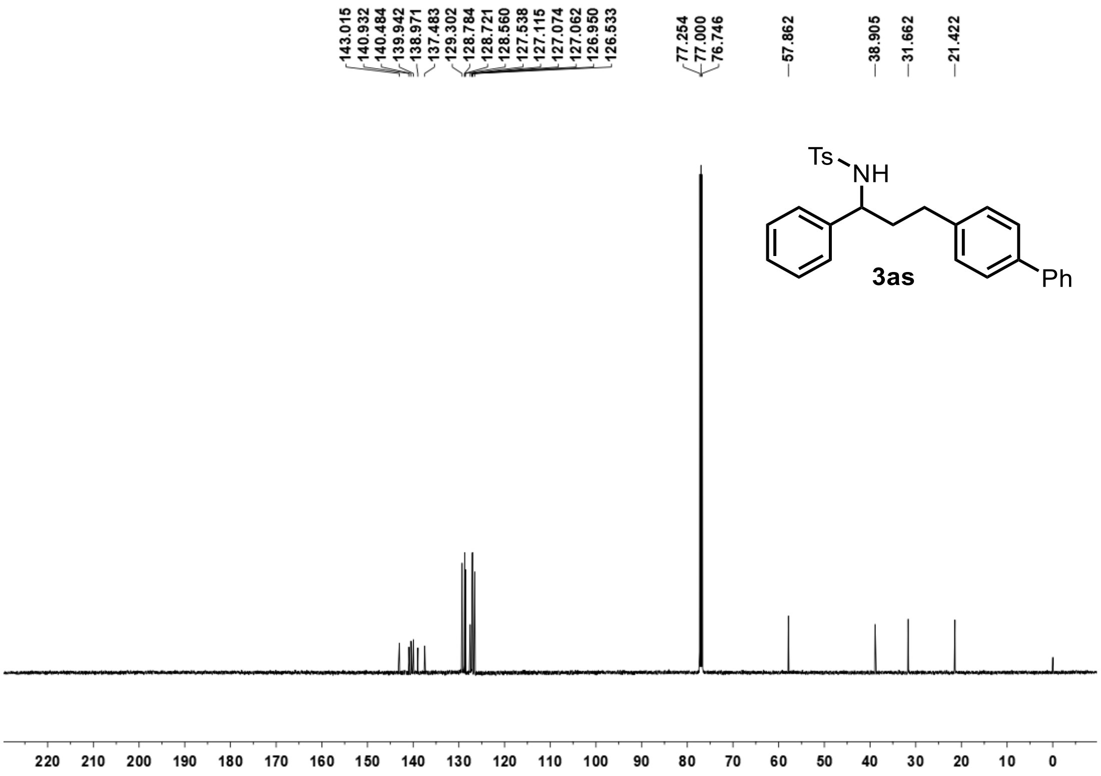

Supplementary Fig. 280 ^{13}C NMR (125 MHz, $CDCl_3$) of **3as**

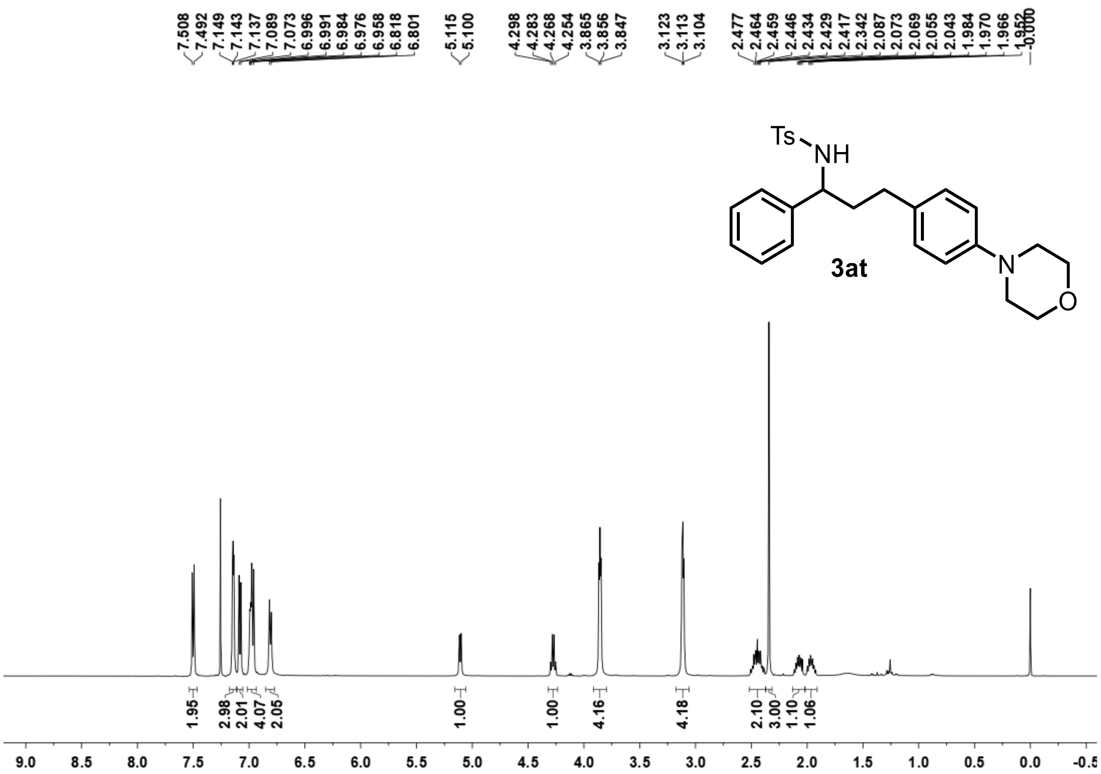

Supplementary Fig. 281 ^{1}H NMR (500 MHz, $CDCl_3$) of **3at**

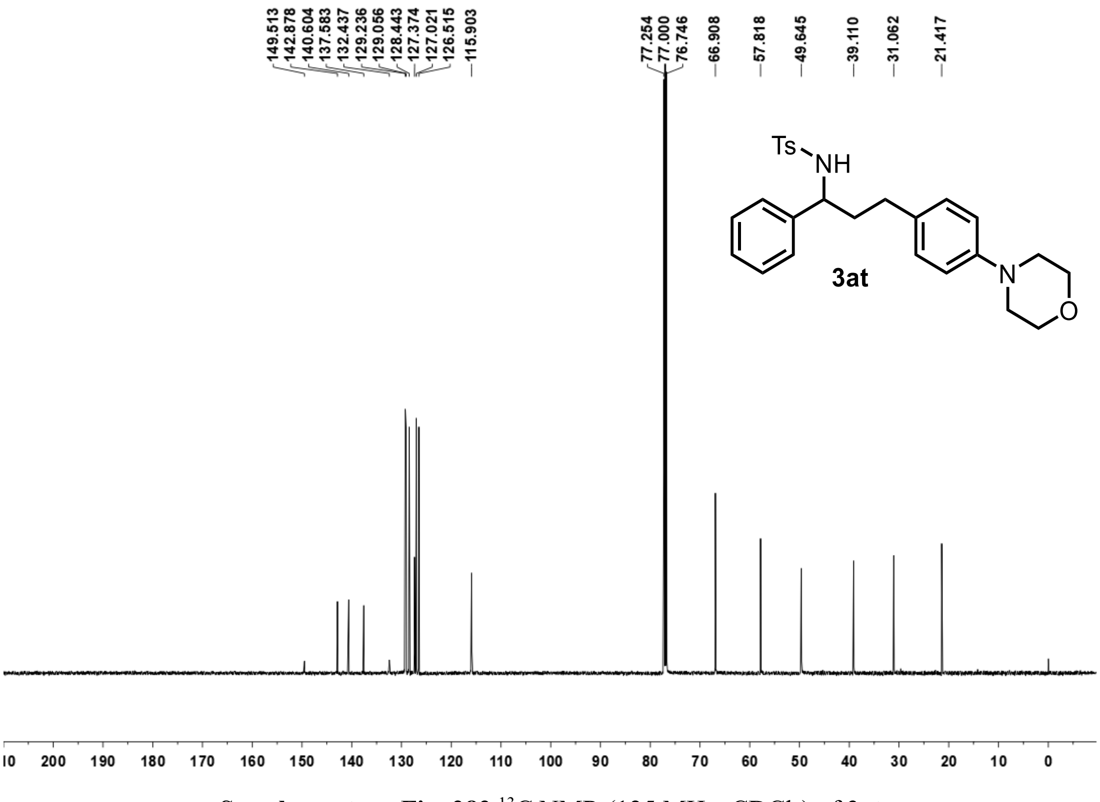

Supplementary Fig. 282 ^{13}C NMR (125 MHz, $CDCl_3$) of **3at**

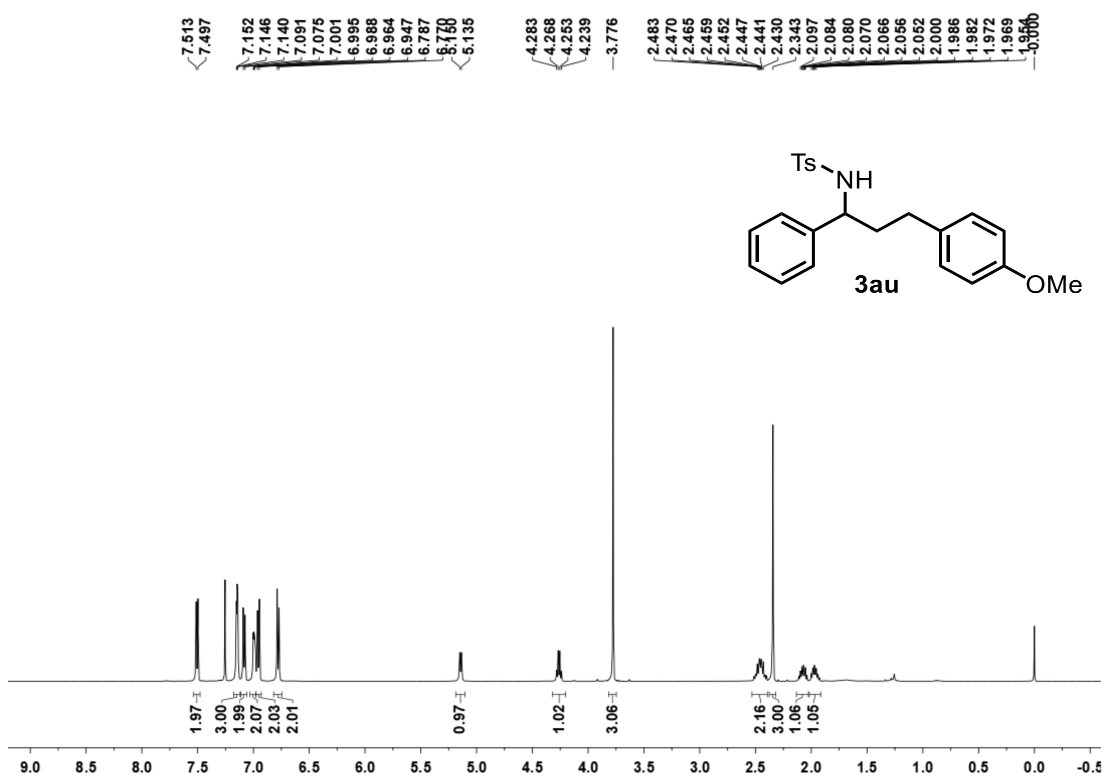

Supplementary Fig. 283 ^{1}H NMR (500 MHz, $CDCl_3$) of **3au**

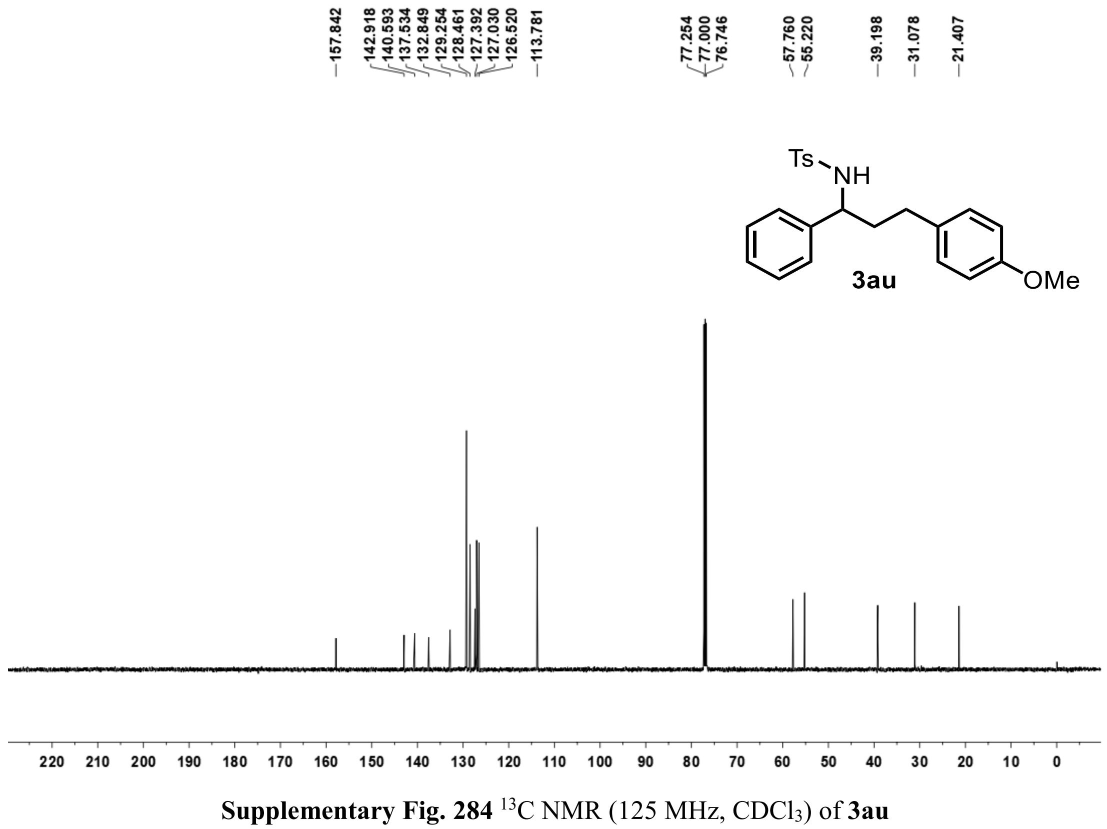

Supplementary Fig. 284 ^{13}C NMR (125 MHz, $CDCl_3$) of **3au**

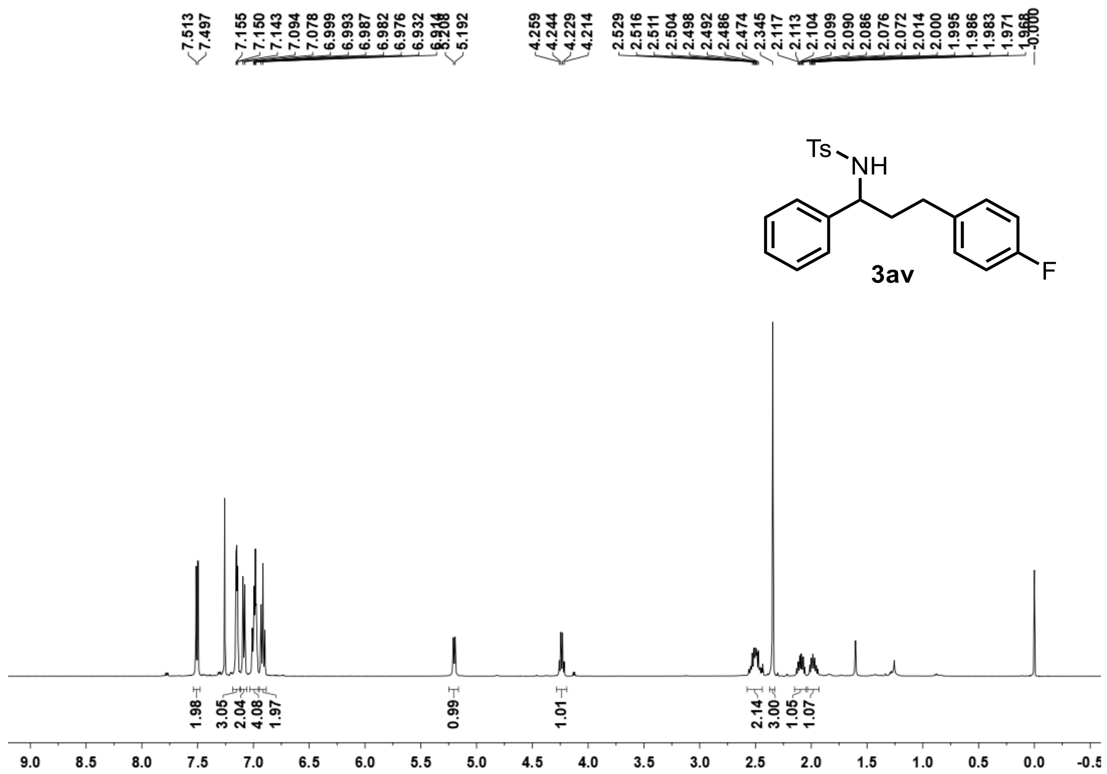

Supplementary Fig. 285 ^{1}H NMR (500 MHz, $CDCl_3$) of **3av**

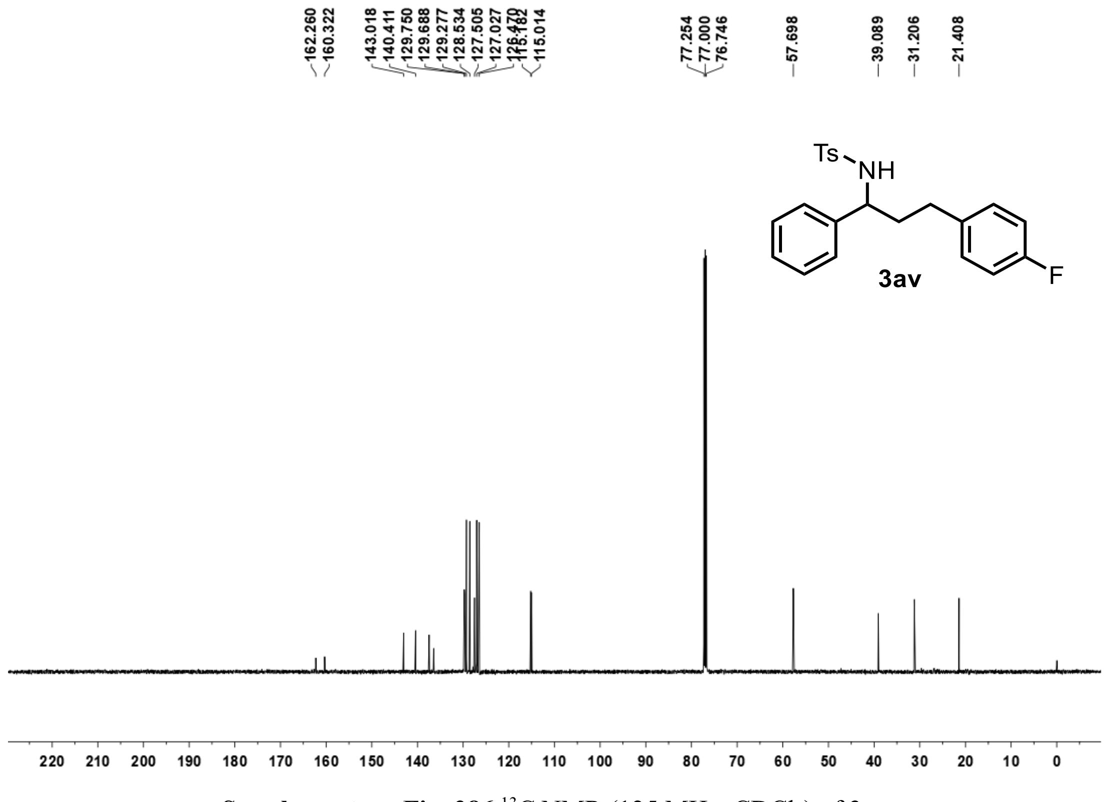

Supplementary Fig. 286 ^{13}C NMR (125 MHz, $CDCl_3$) of **3av**

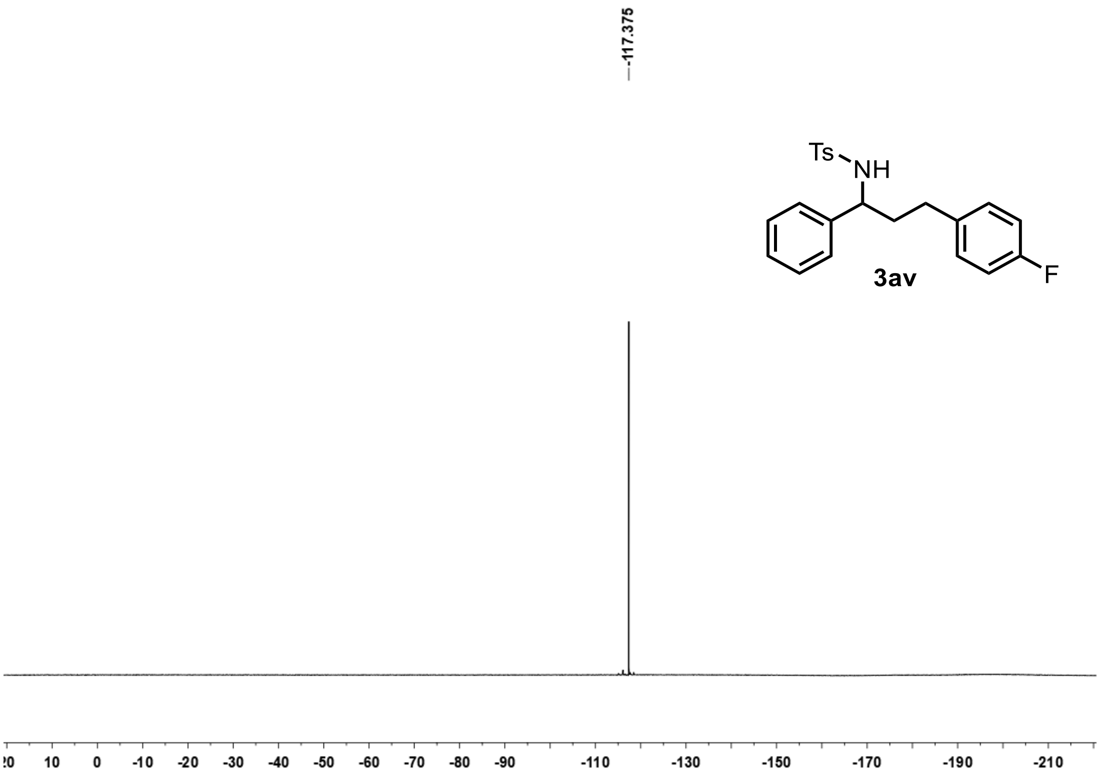

Supplementary Fig. 287 ^{19}F NMR (470 MHz, $CDCl_3$) of **3av**

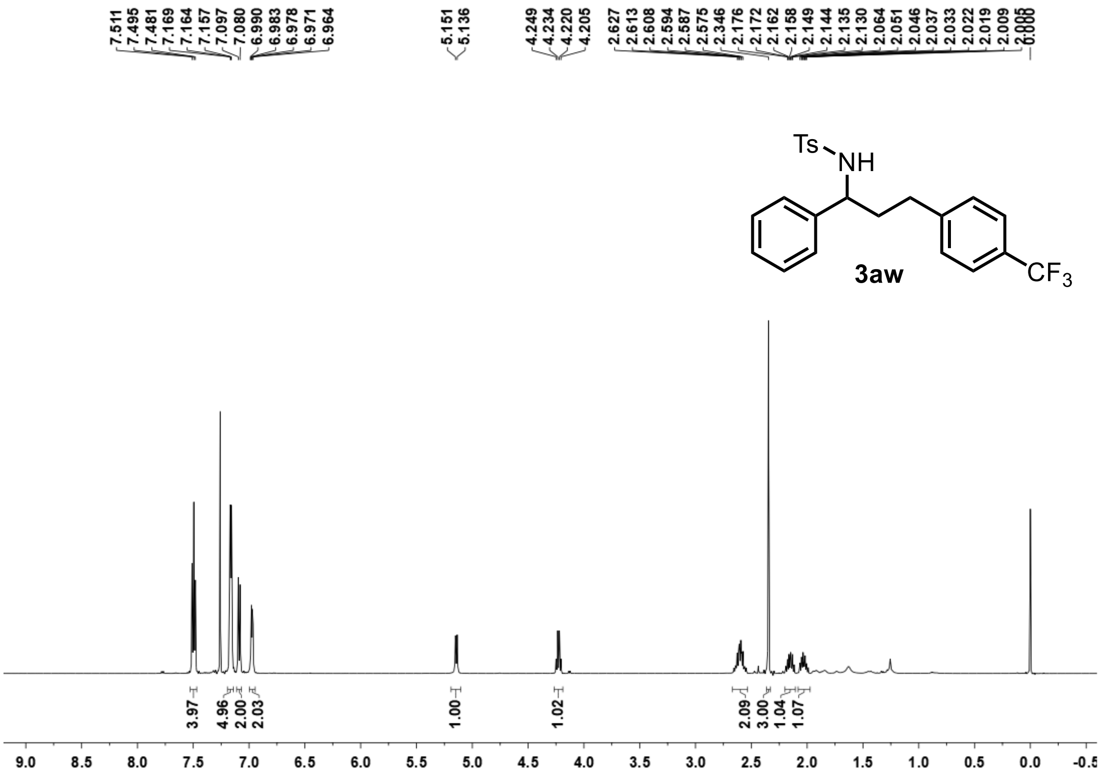

Supplementary Fig. 288 ^{1}H NMR (500 MHz, $CDCl_3$) of **3aw**

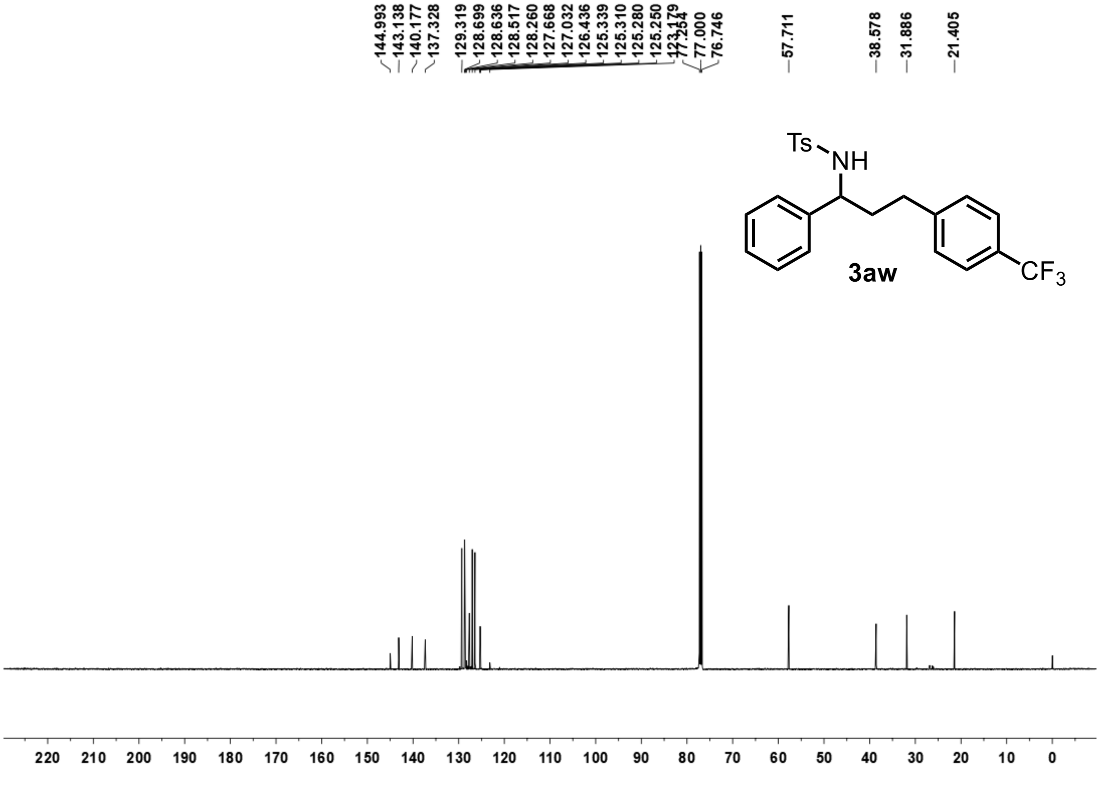

Supplementary Fig. 289 ^{13}C NMR (125 MHz, $CDCl_3$) of **3aw**

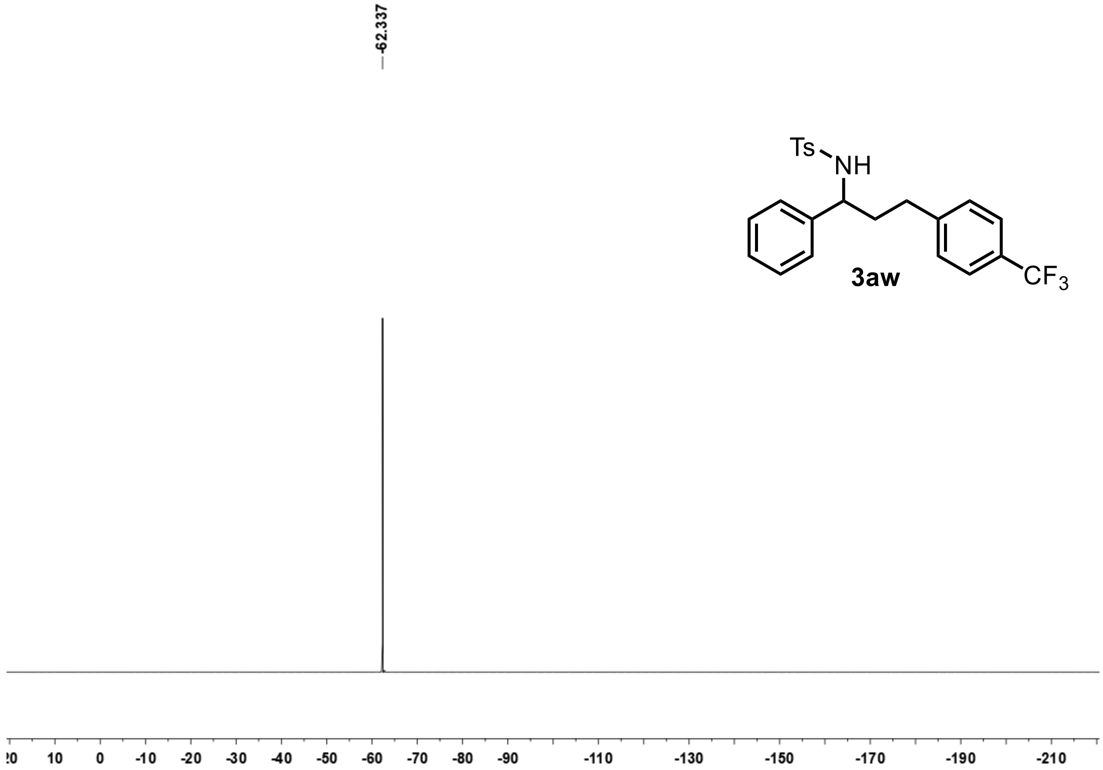

Supplementary Fig. 290 ^{19}F NMR (470 MHz, $CDCl_3$) of **3aw**

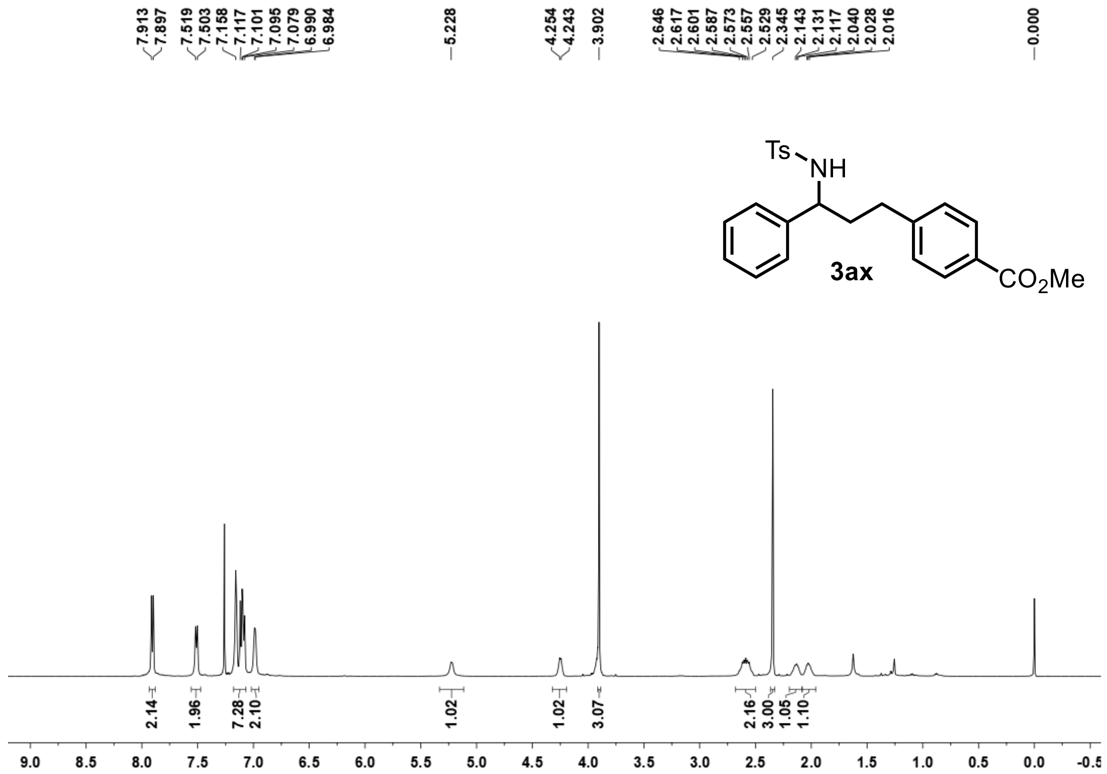

Supplementary Fig. 291 ^{1}H NMR (500 MHz, $CDCl_3$) of **3ax**

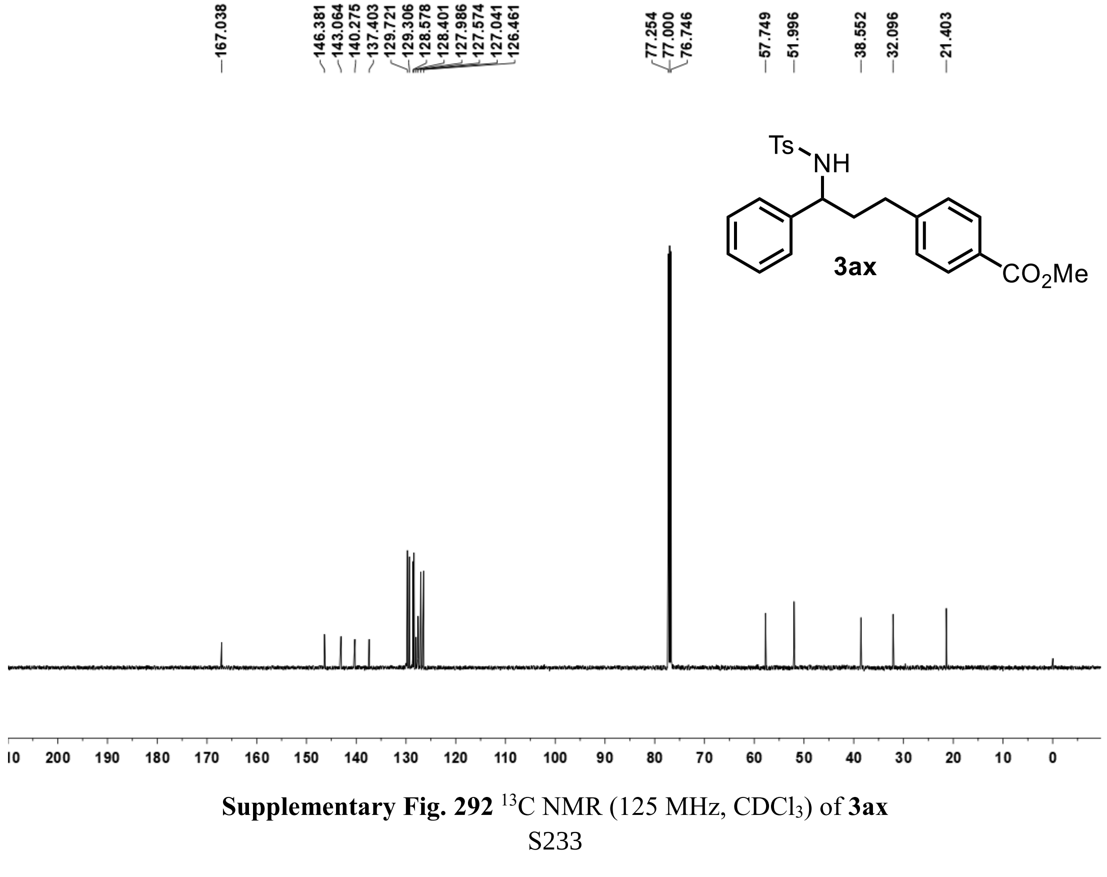

Supplementary Fig. 292 ^{13}C NMR (125 MHz, $CDCl_3$) of **3ax**

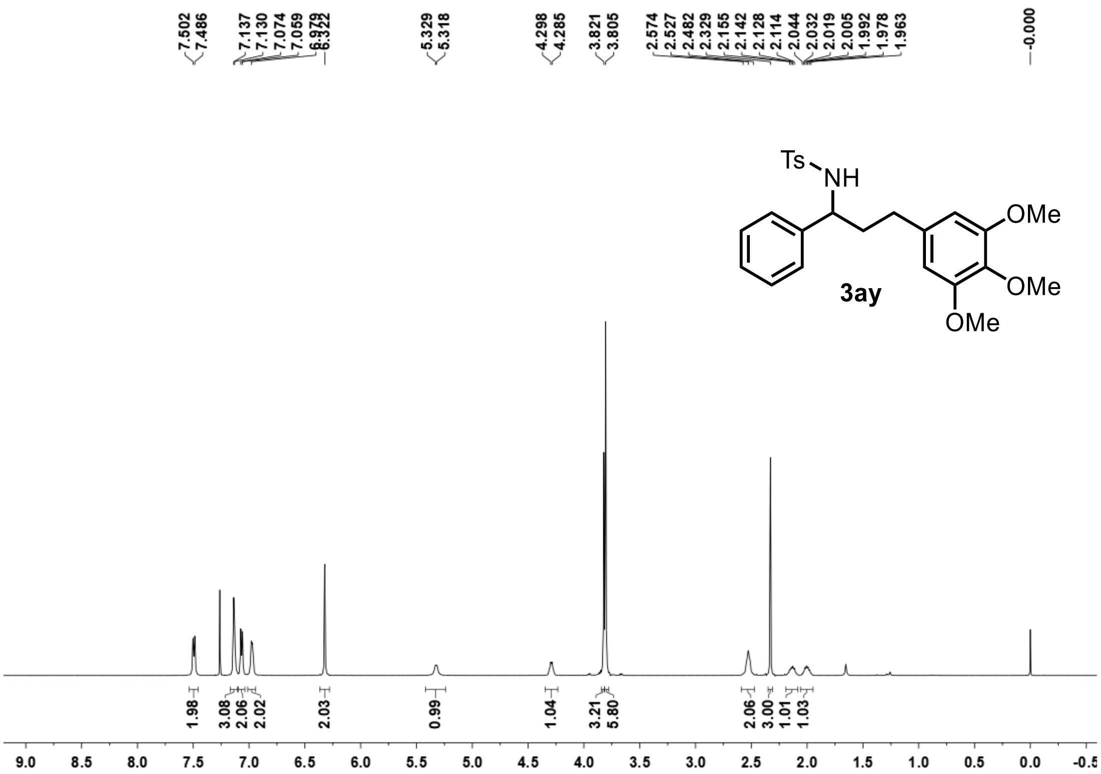

Supplementary Fig. 293 ^{1}H NMR (500 MHz, $CDCl_3$) of **3ay**

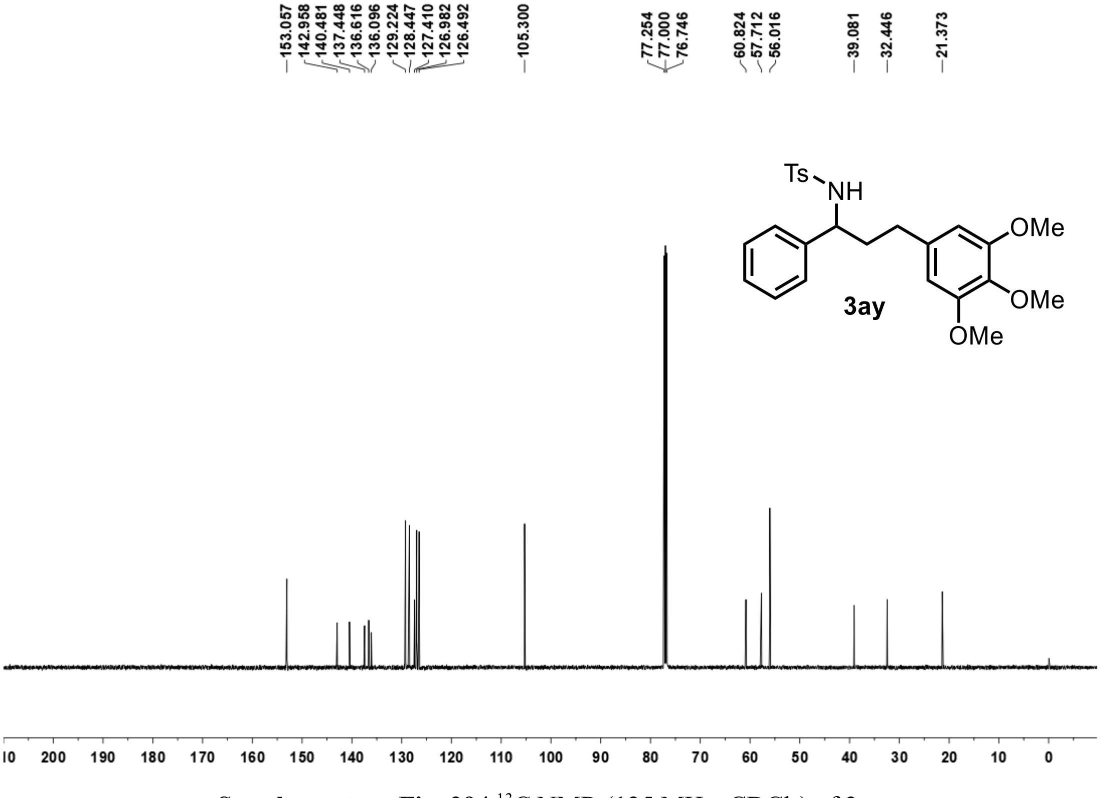

Supplementary Fig. 294 ^{13}C NMR (125 MHz, $CDCl_3$) of **3ay**

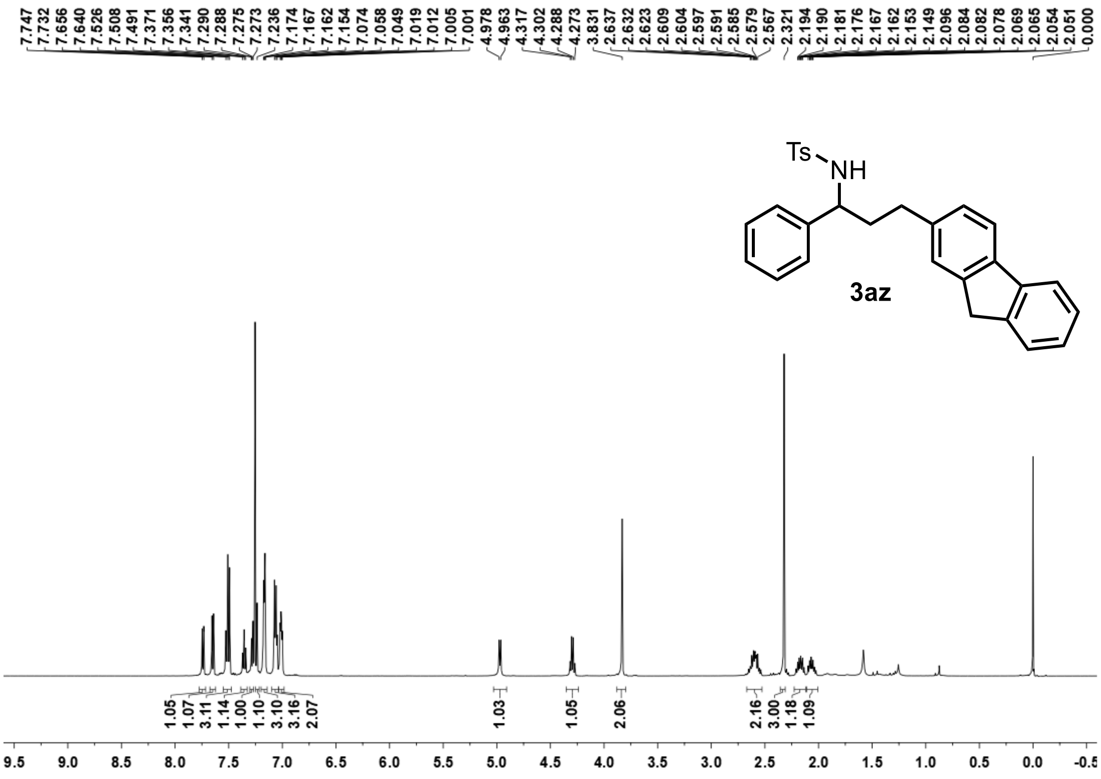

Supplementary Fig. 295 ^{1}H NMR (500 MHz, $CDCl_3$) of **3az**

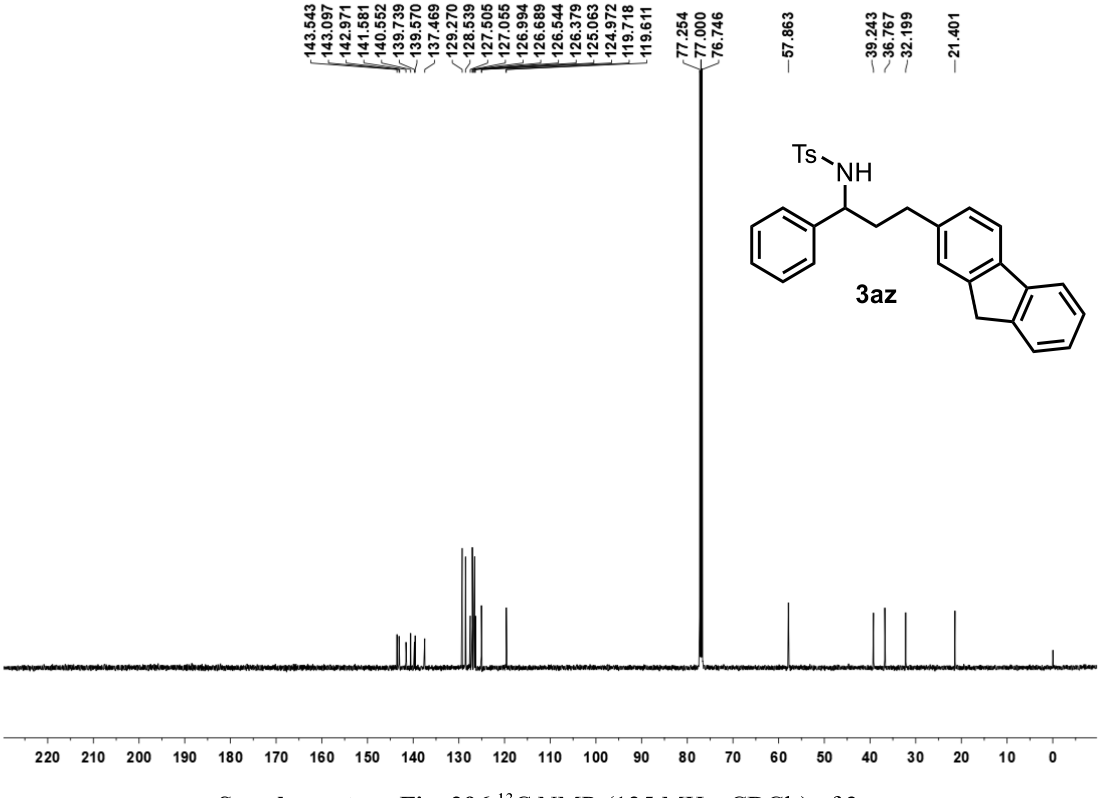

Supplementary Fig. 296 ^{13}C NMR (125 MHz, $CDCl_3$) of **3az**

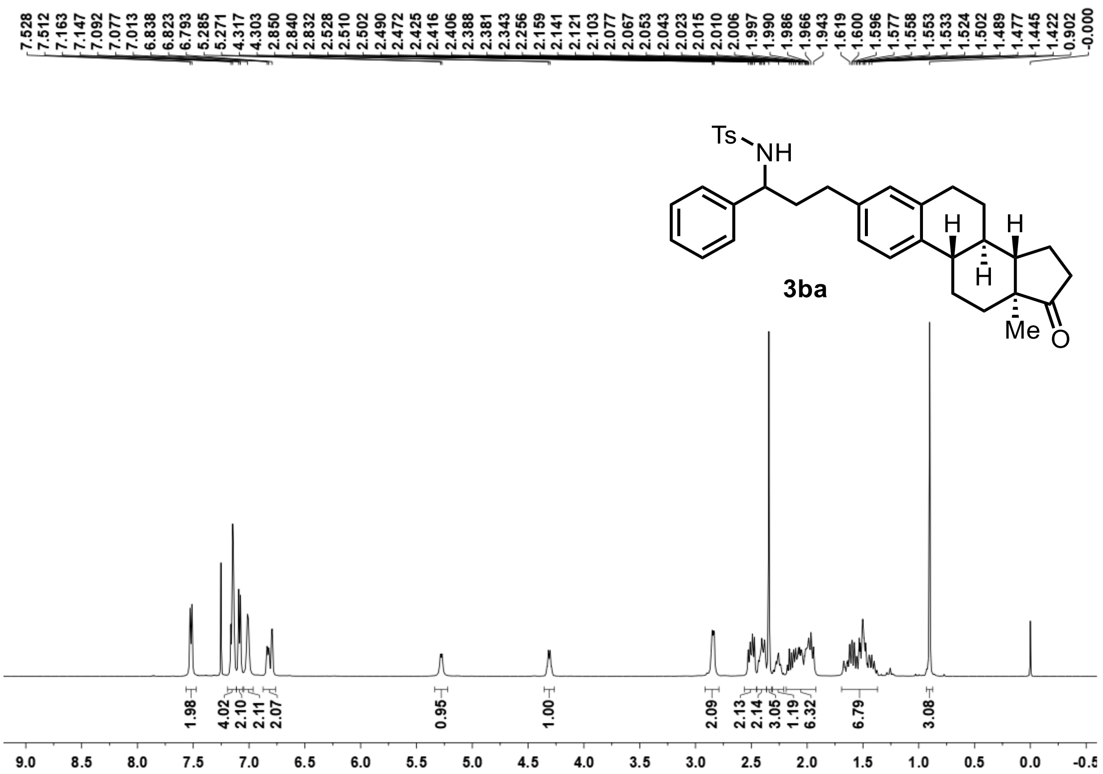

Supplementary Fig. 297 ^{1}H NMR (500 MHz, $CDCl_3$) of **3ba**

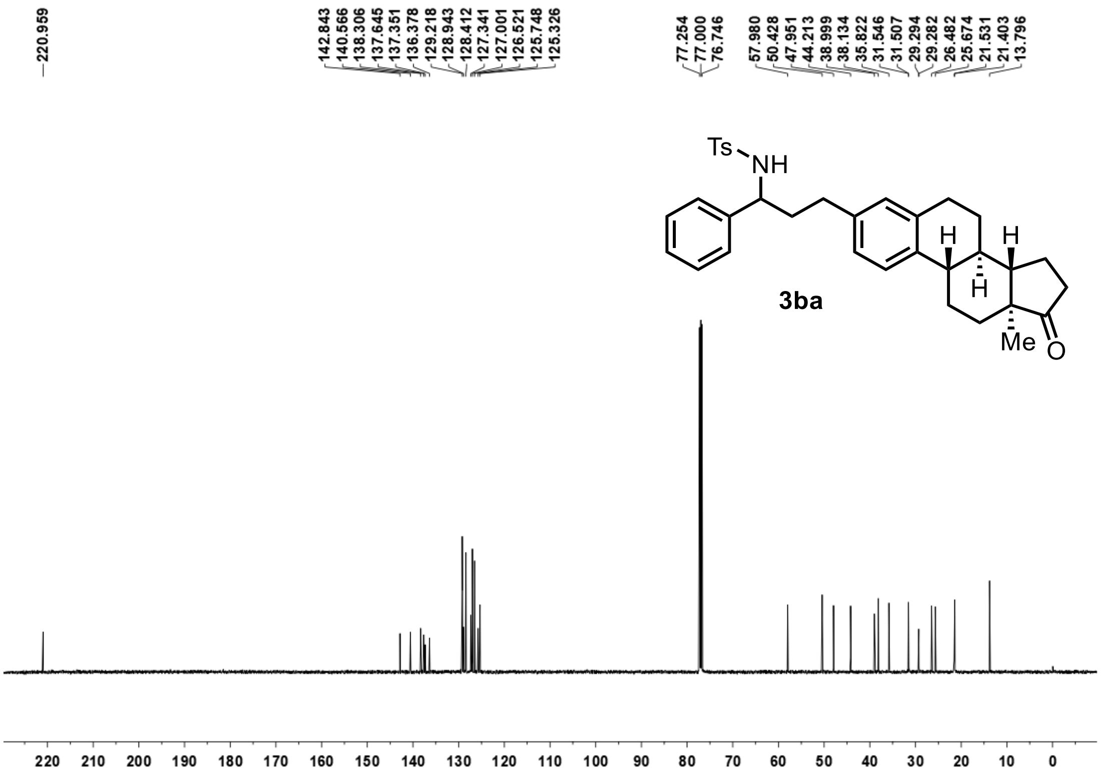

Supplementary Fig. 298 ^{13}C NMR (125 MHz, $CDCl_3$) of **3ba**

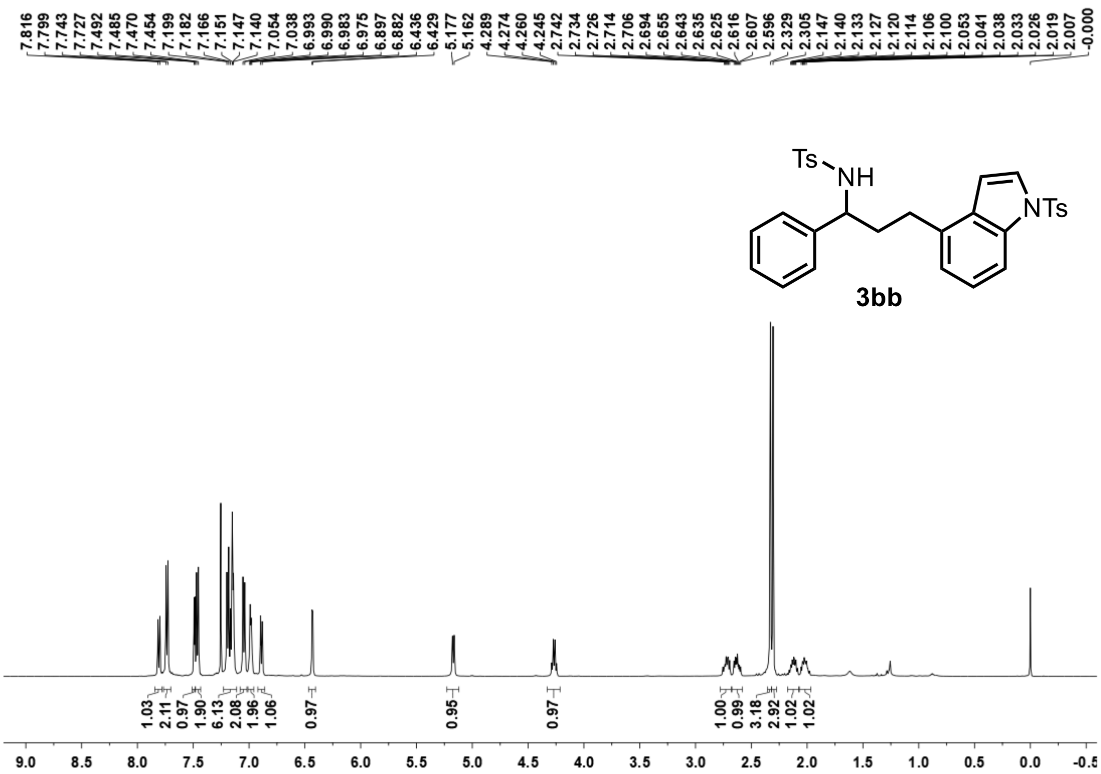

Supplementary Fig. 299 ^{1}H NMR (500 MHz, $CDCl_3$) of **3bb**

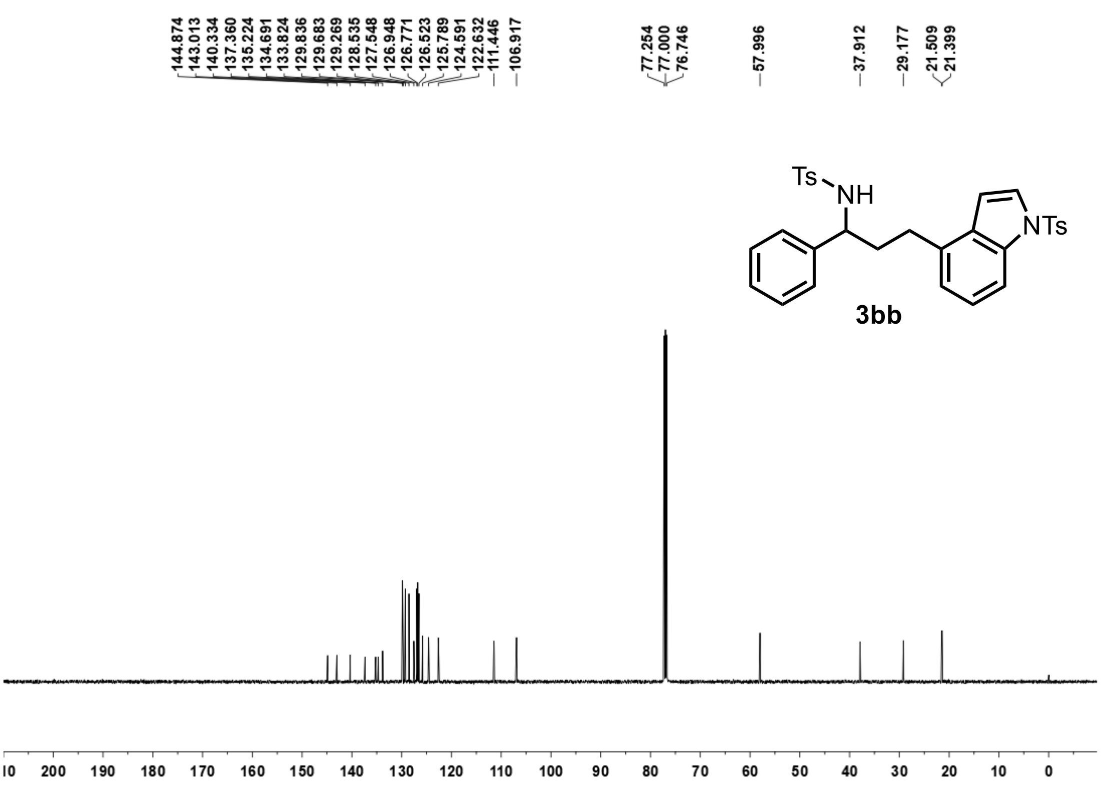

Supplementary Fig. 300 ^{13}C NMR (125 MHz, $CDCl_3$) of **3bb**

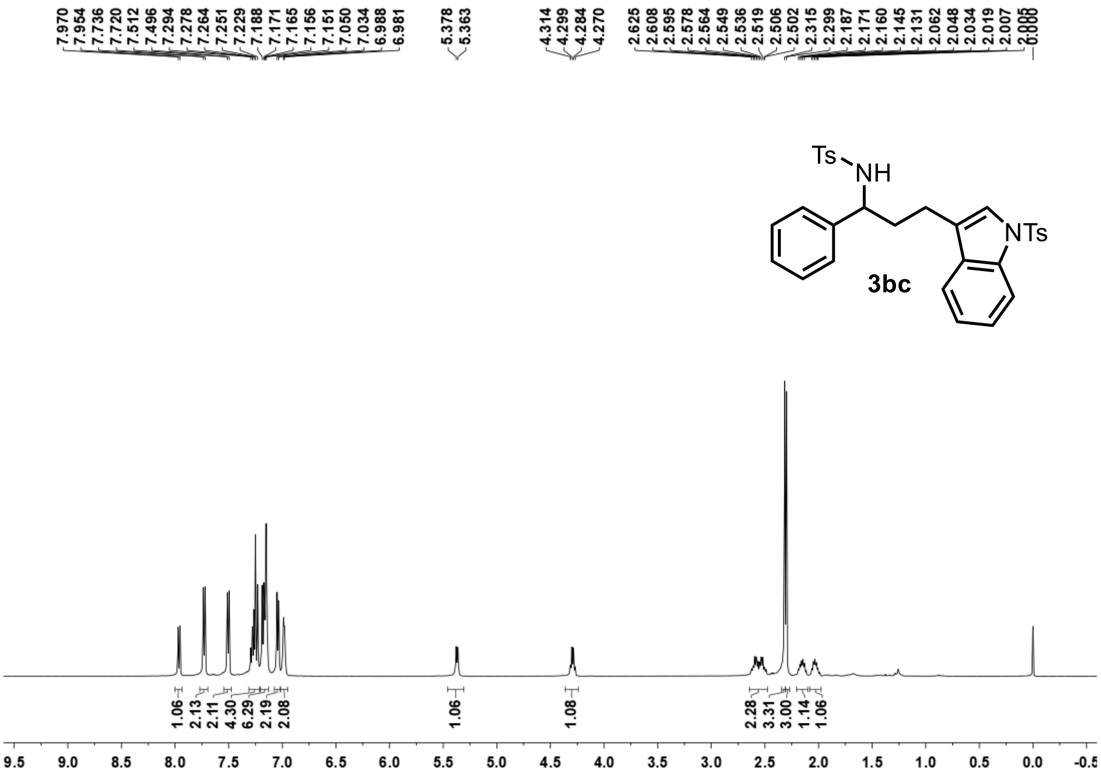

Supplementary Fig. 301 ^{1}H NMR (500 MHz, $CDCl_3$) of **3bc**

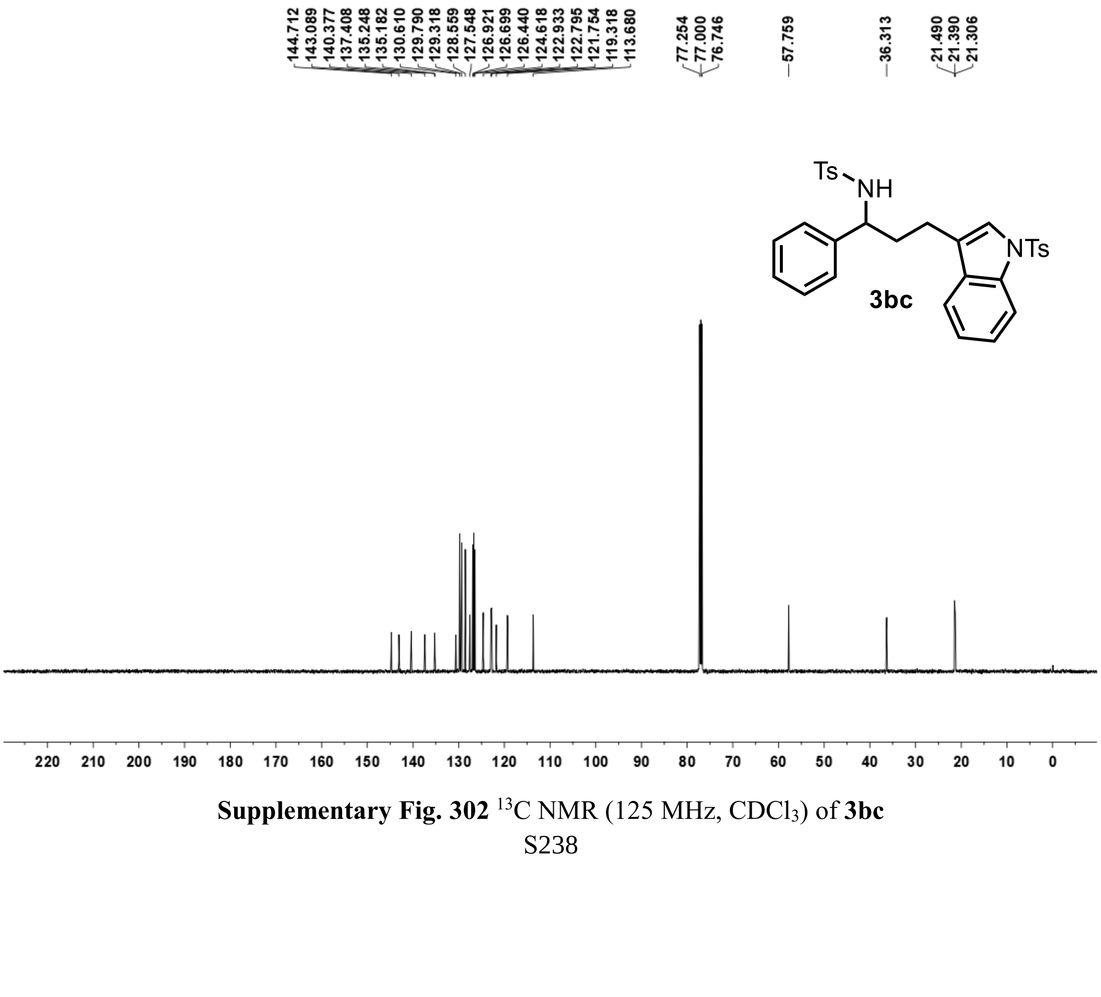

Supplementary Fig. 302 ^{13}C NMR (125 MHz, $CDCl_3$) of **3bc**

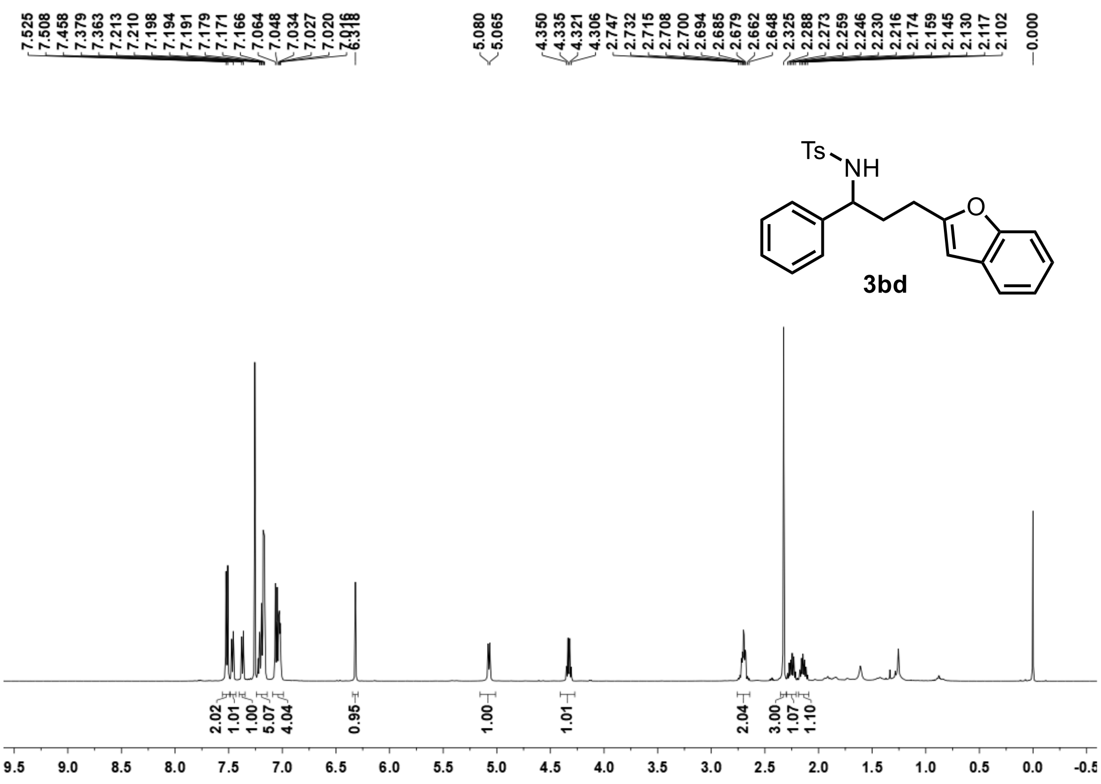

Supplementary Fig. 303 ^{1}H NMR (500 MHz, $CDCl_3$) of **3bd**

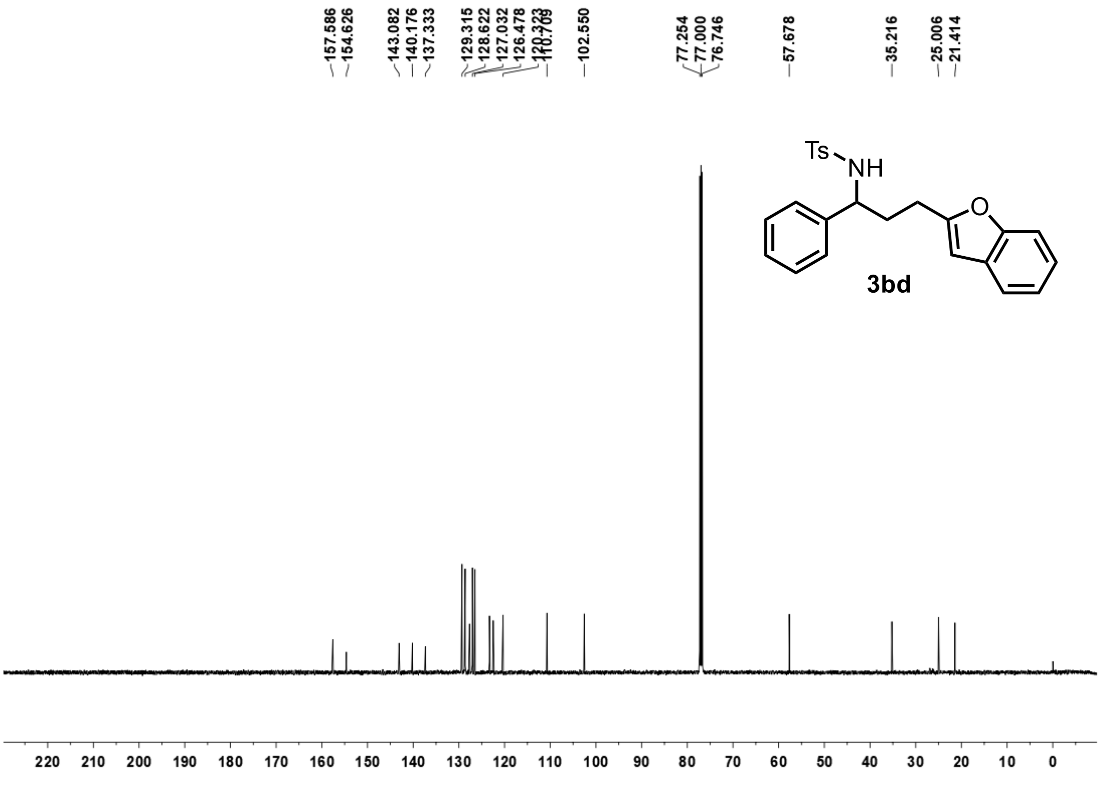

Supplementary Fig. 304 ^{13}C NMR (125 MHz, $CDCl_3$) of **3bd**

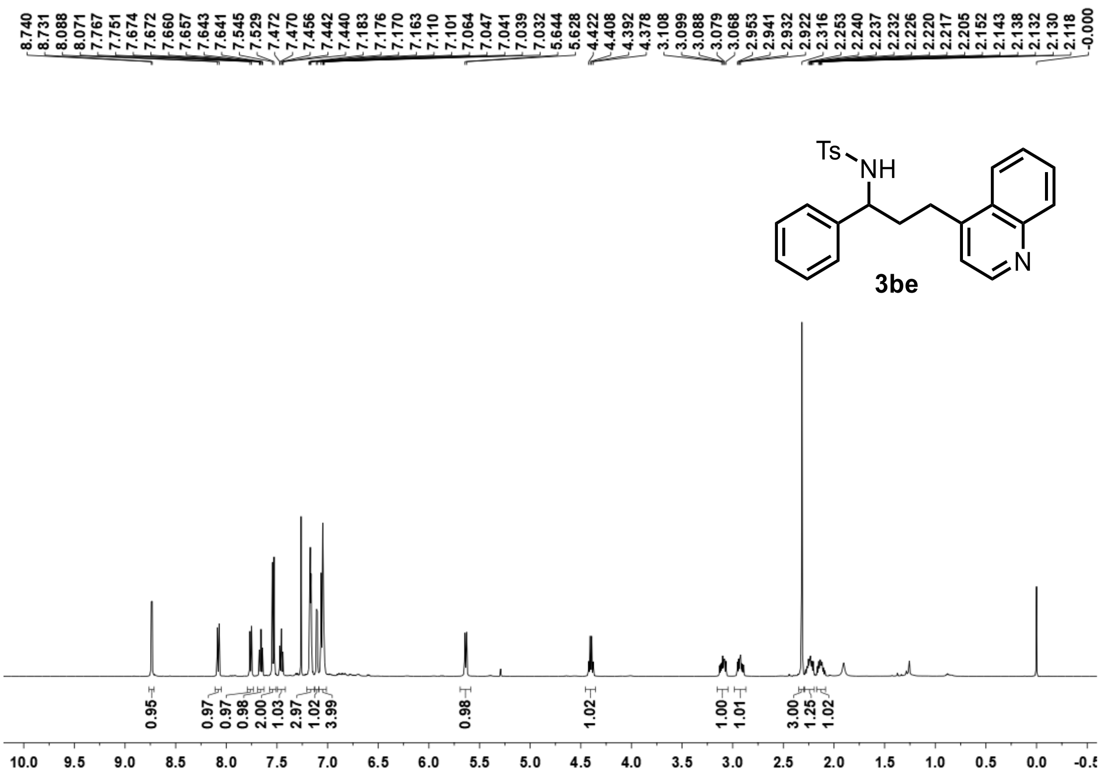

Supplementary Fig. 305 ^{1}H NMR (500 MHz, $CDCl_3$) of **3be**

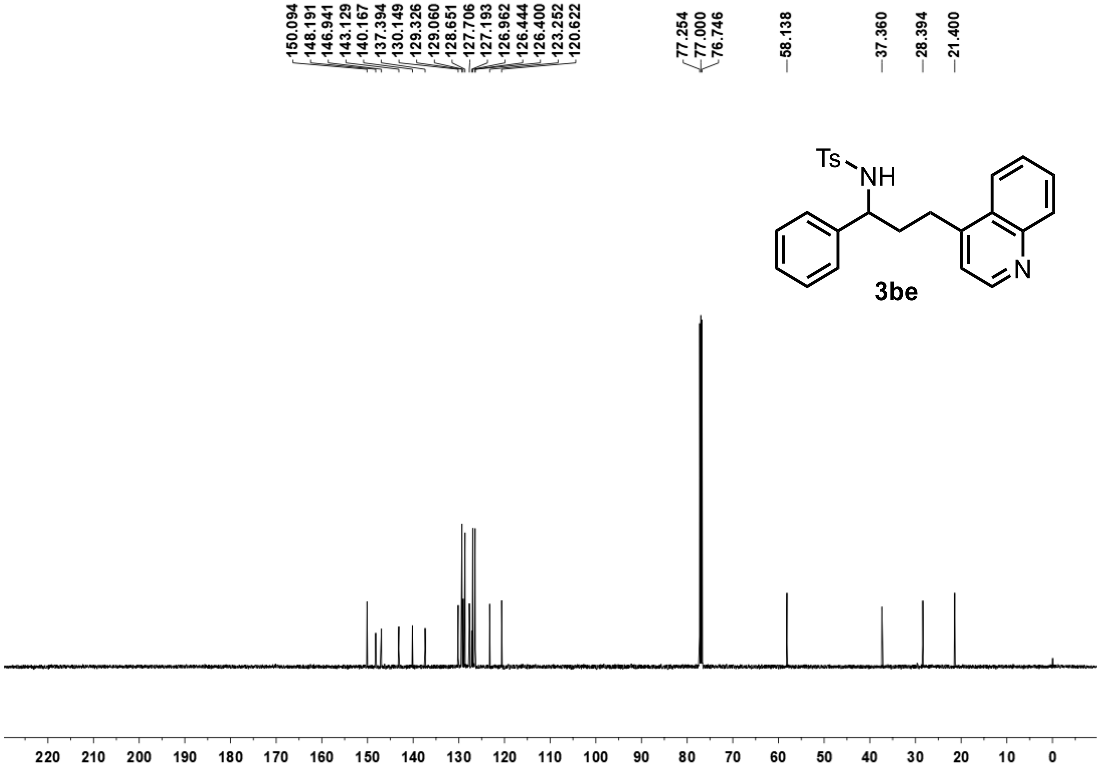

Supplementary Fig. 306 ^{13}C NMR (125 MHz, $CDCl_3$) of **3be**

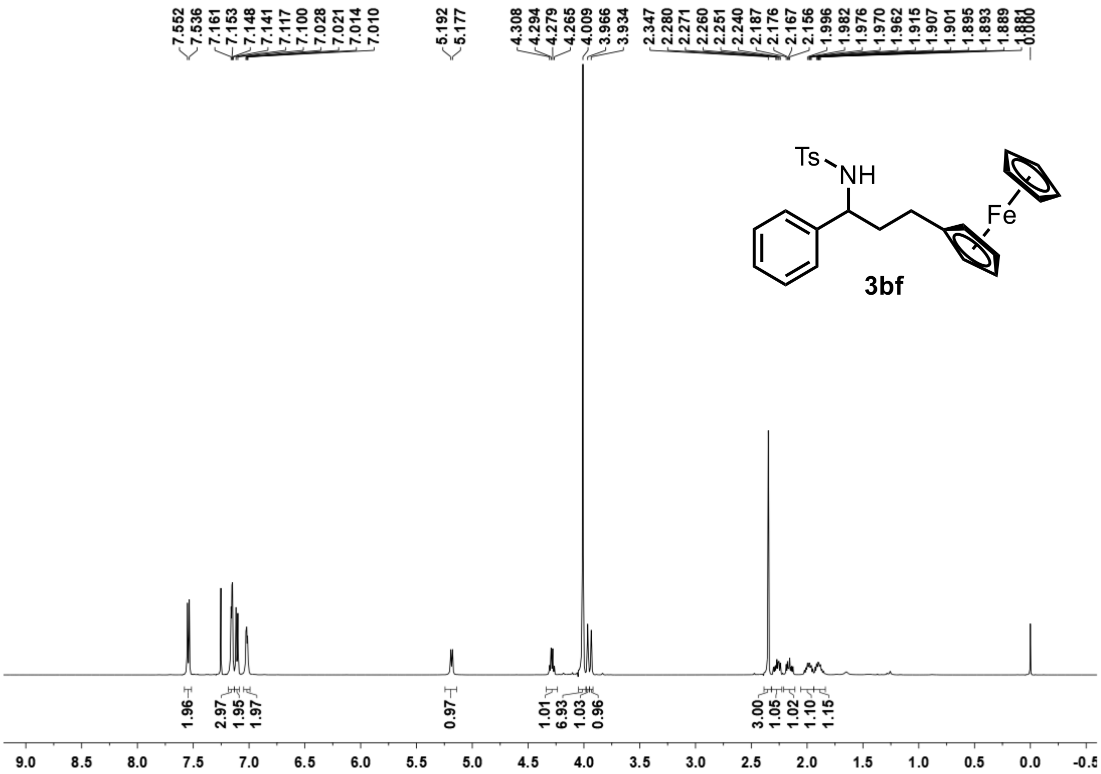

Supplementary Fig. 307 ^{1}H NMR (500 MHz, $CDCl_3$) of **3bf**

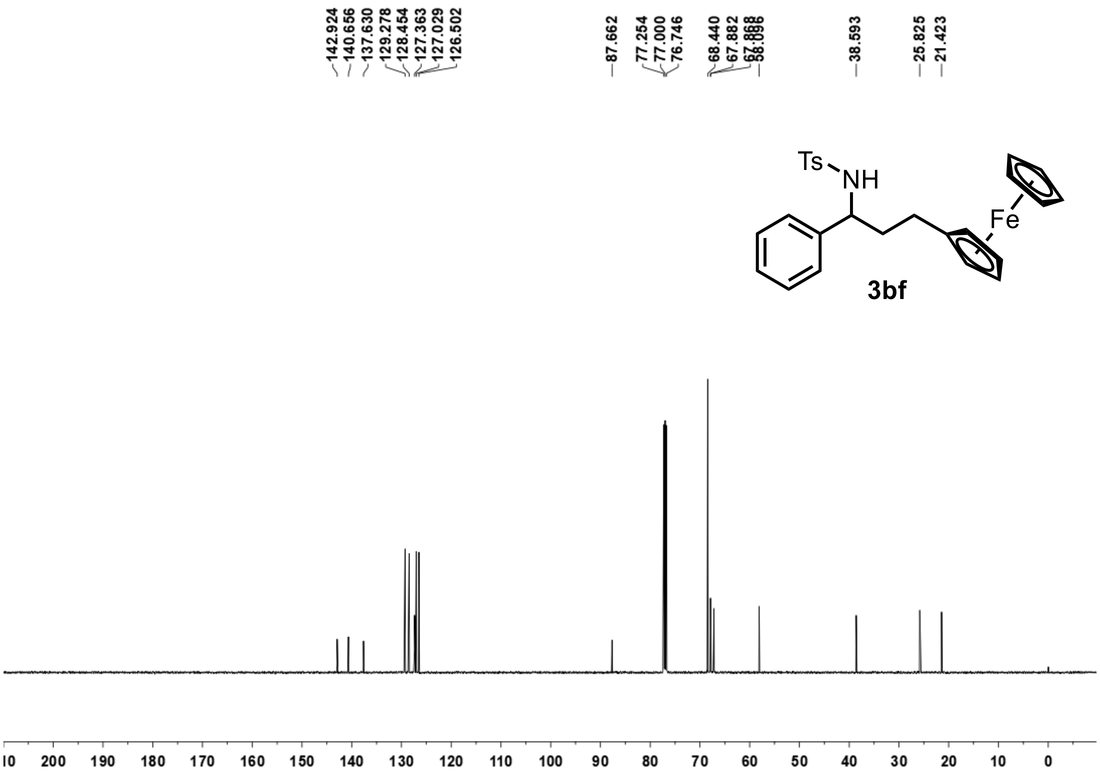

Supplementary Fig. 308 ^{13}C NMR (125 MHz, $CDCl_3$) of **3bf**

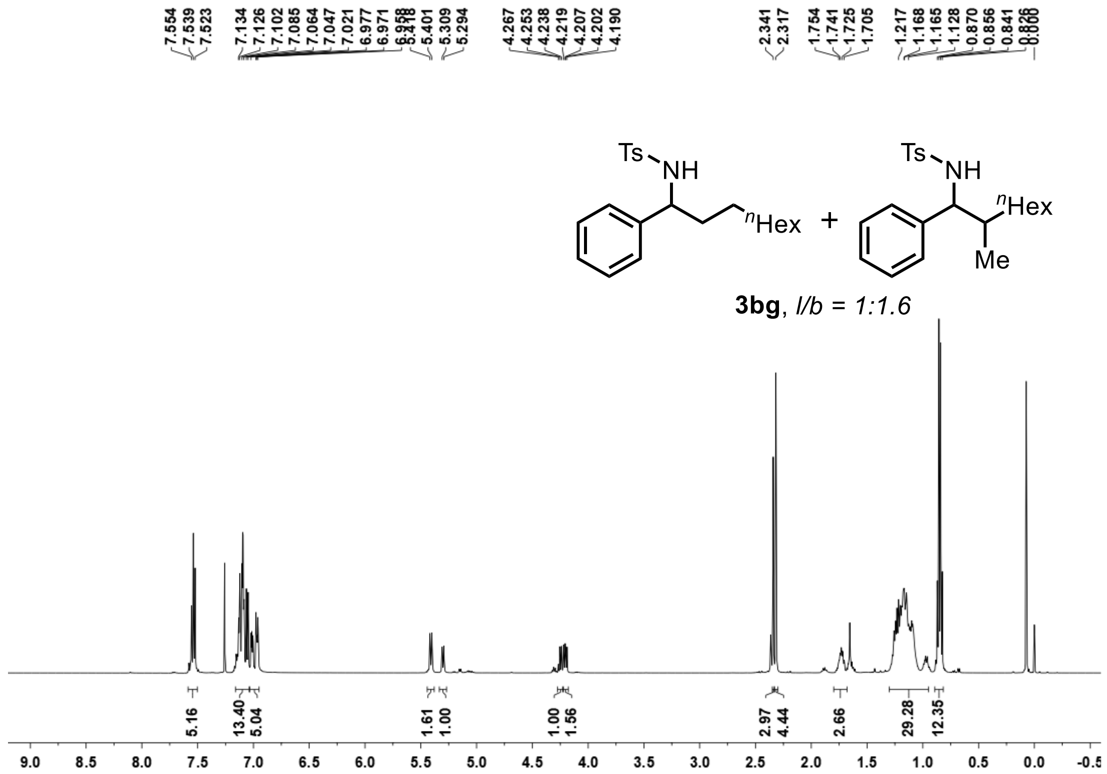

Supplementary Fig. 309 ^{1}H NMR (500 MHz, $CDCl_3$) of **3bg**

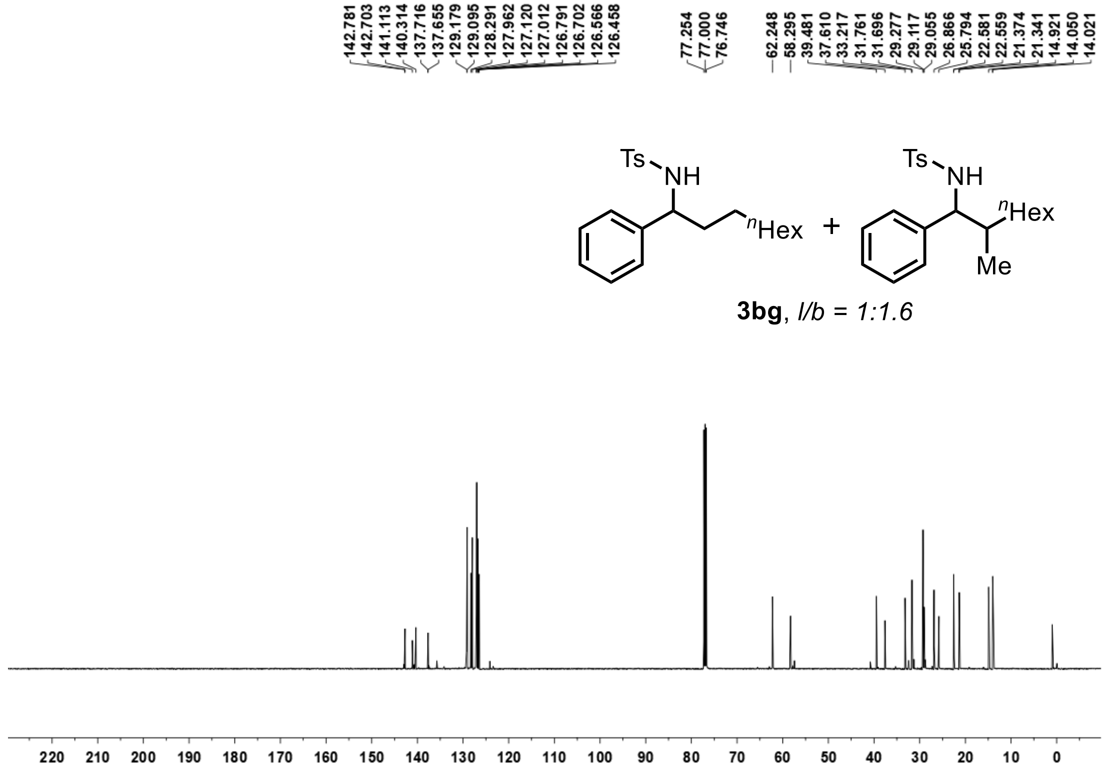

Supplementary Fig. 310 ^{13}C NMR (125 MHz, $CDCl_3$) of **3bg**

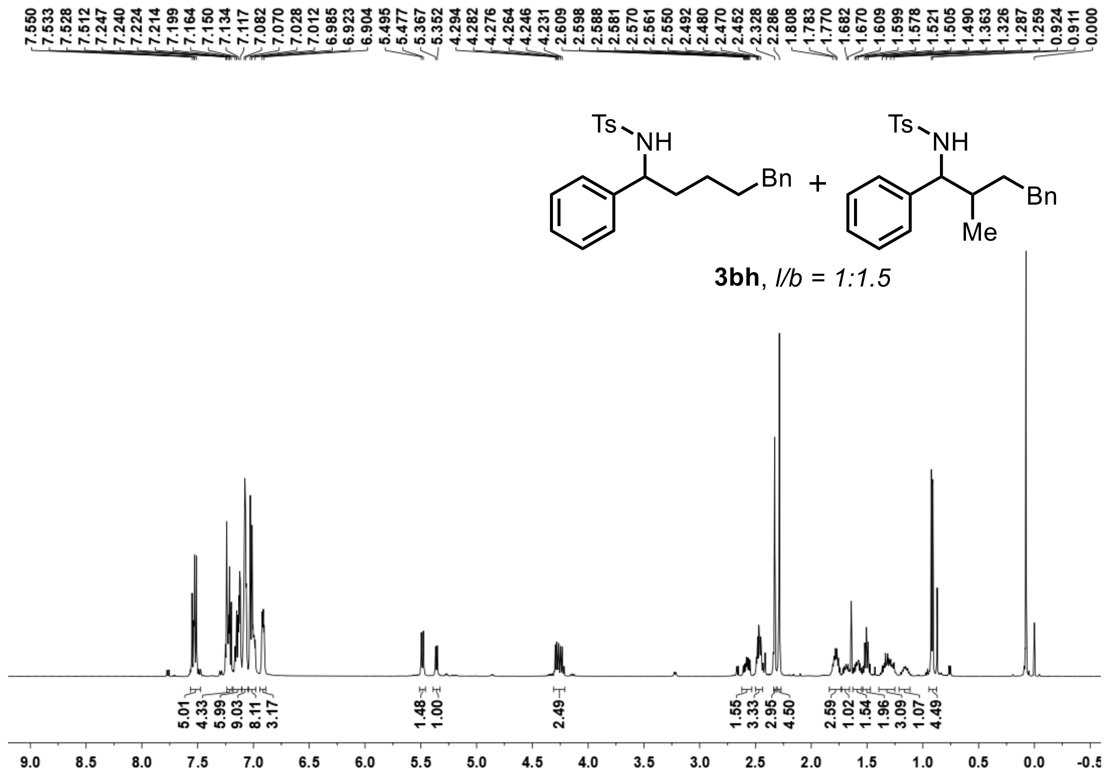

Supplementary Fig. 311 ^{1}H NMR (500 MHz, $CDCl_3$) of **3bh**

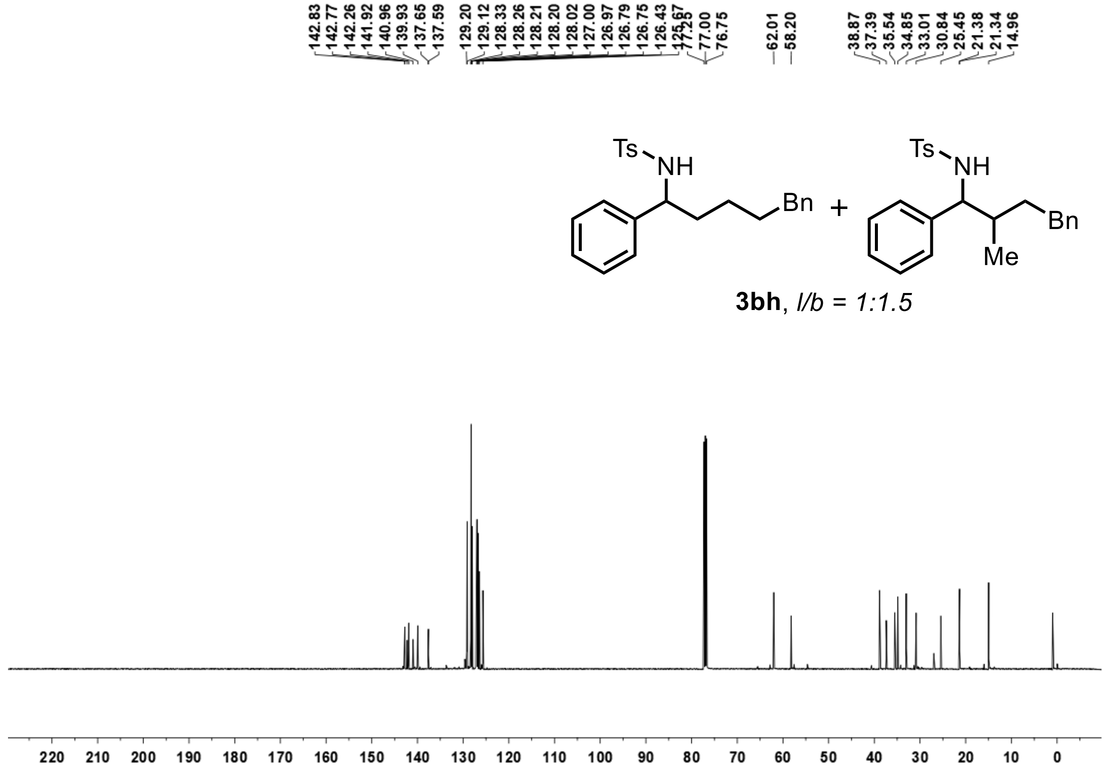

Supplementary Fig. 312 ^{13}C NMR (125 MHz, $CDCl_3$) of **3bh**

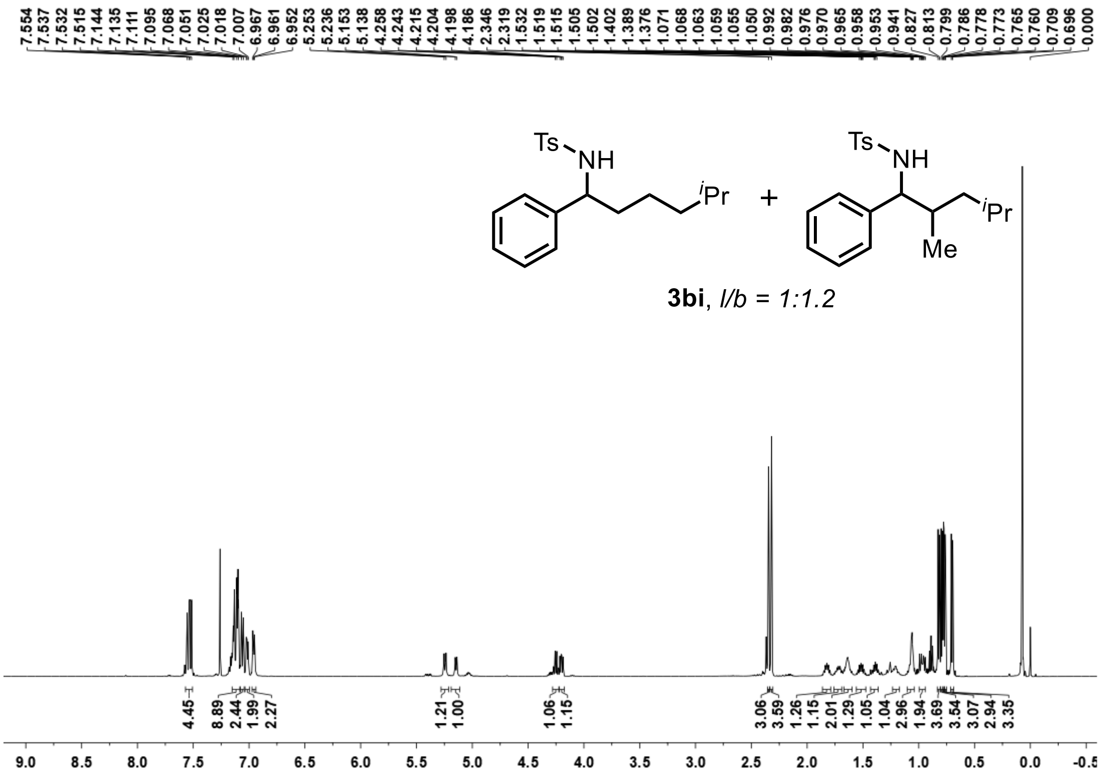

Supplementary Fig. 313 ^{1}H NMR (500 MHz, $CDCl_3$) of **3bi**

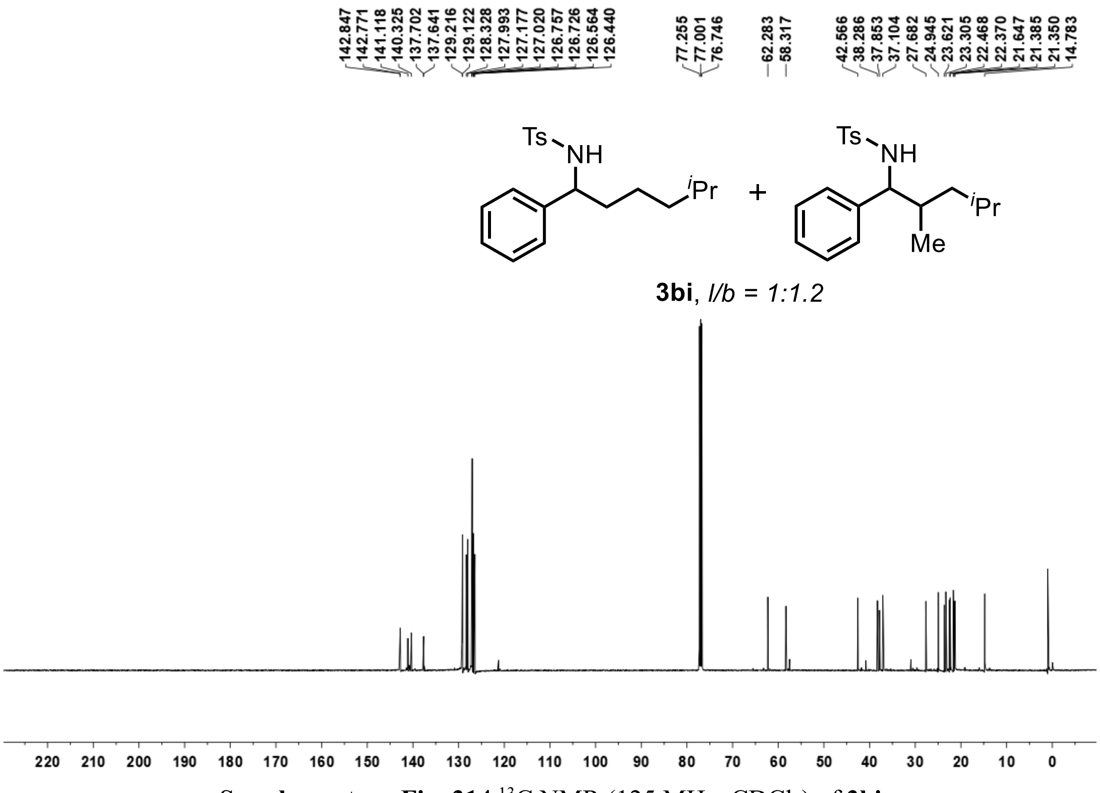

Supplementary Fig. 314 ^{13}C NMR (125 MHz, $CDCl_3$) of **3bi**

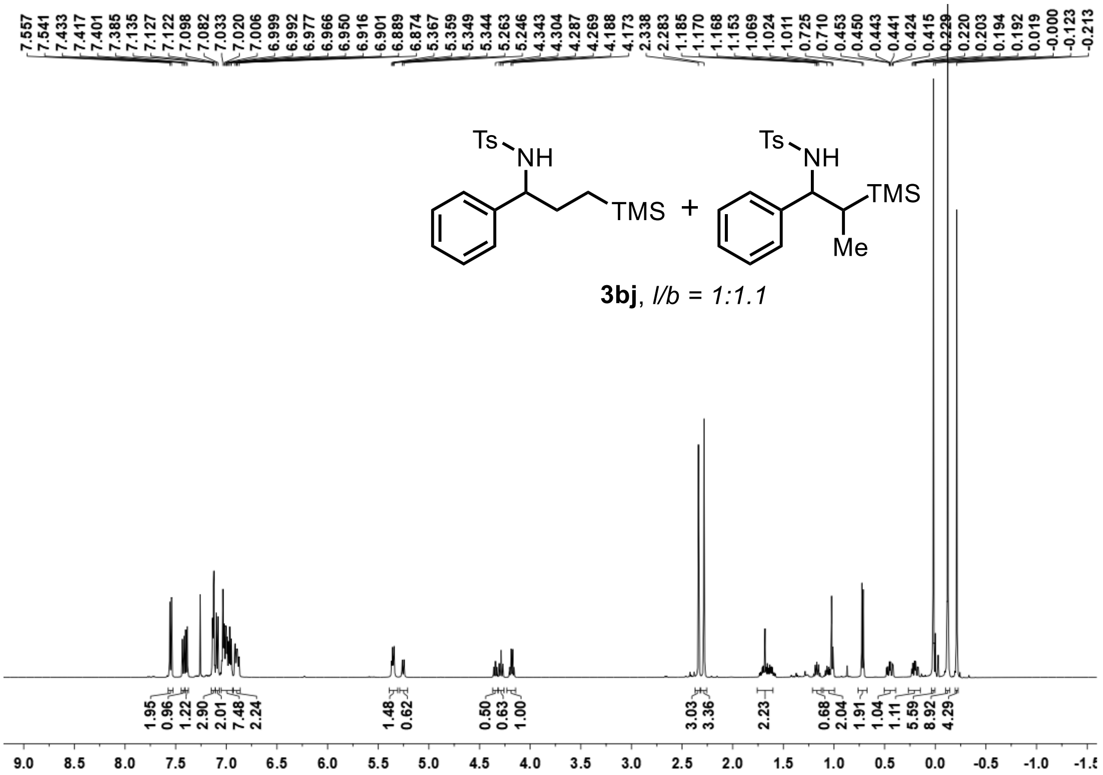

Supplementary Fig. 315 ^{1}H NMR (500 MHz, $CDCl_3$) of **3bj**

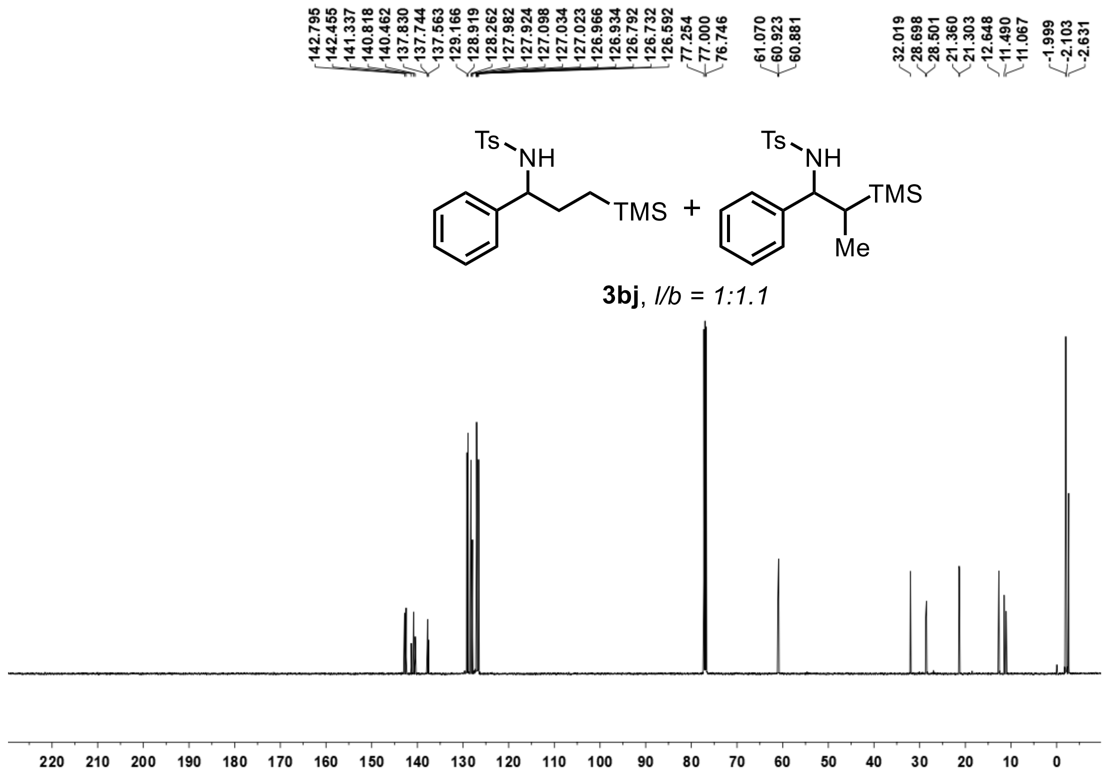

Supplementary Fig. 316 ^{13}C NMR (125 MHz, $CDCl_3$) of **3bj**

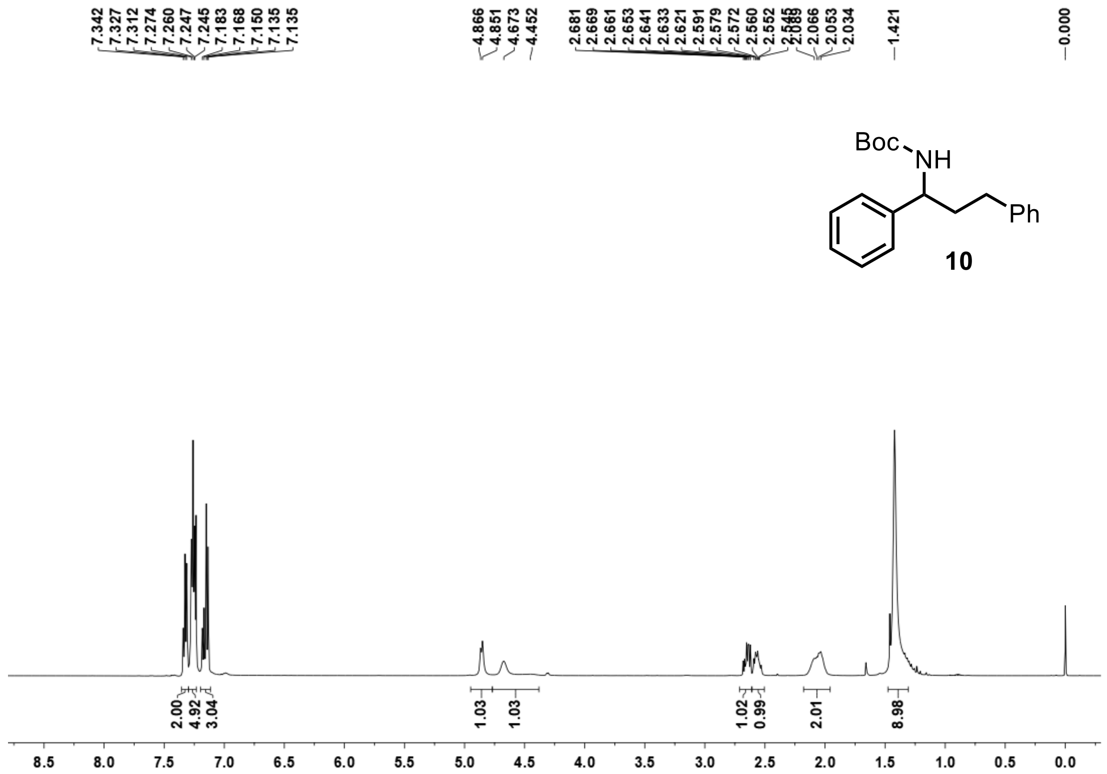

Supplementary Fig. 317 ^{1}H NMR (500 MHz, $CDCl_3$) of **10**

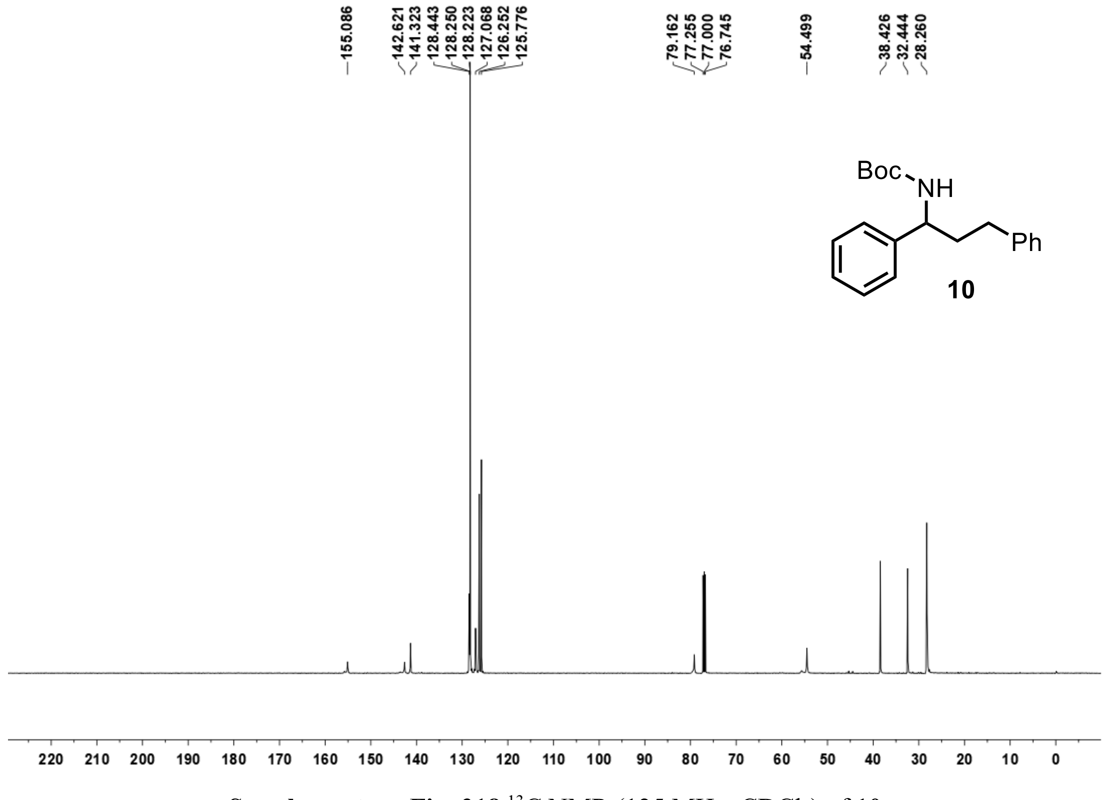

Supplementary Fig. 318 ^{13}C NMR (125 MHz, $CDCl_3$) of **10**

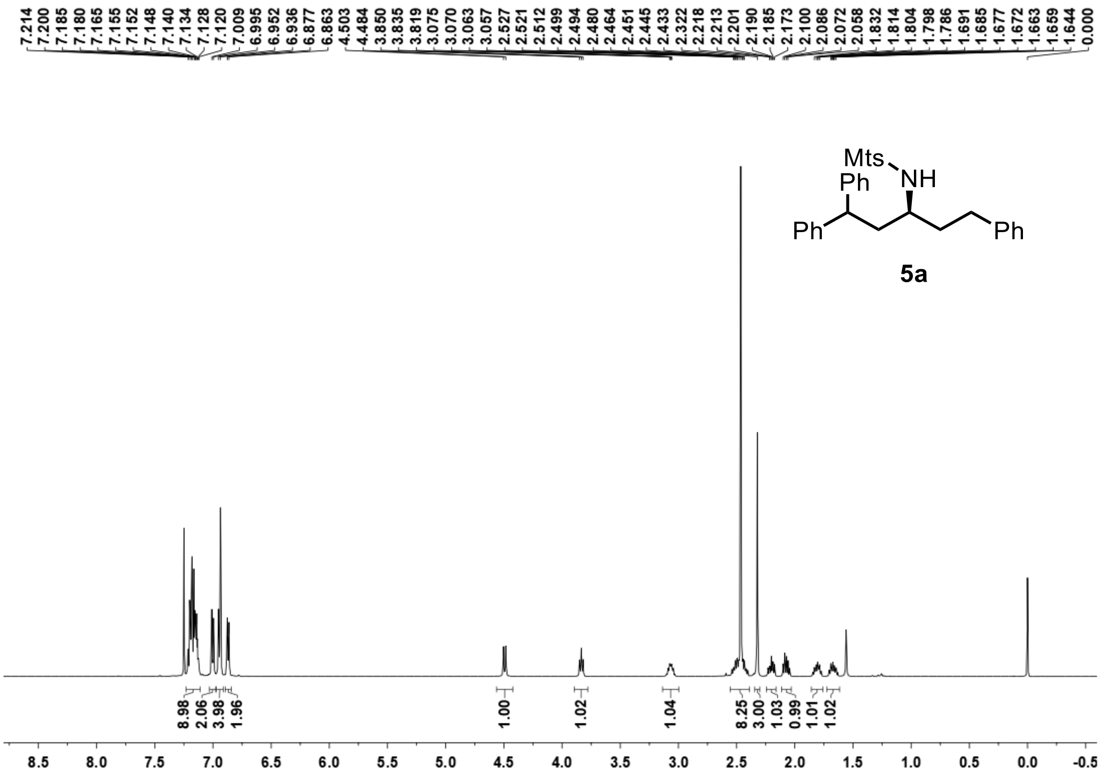

Supplementary Fig. 319 ^{1}H NMR (500 MHz, $CDCl_3$) of **5a**

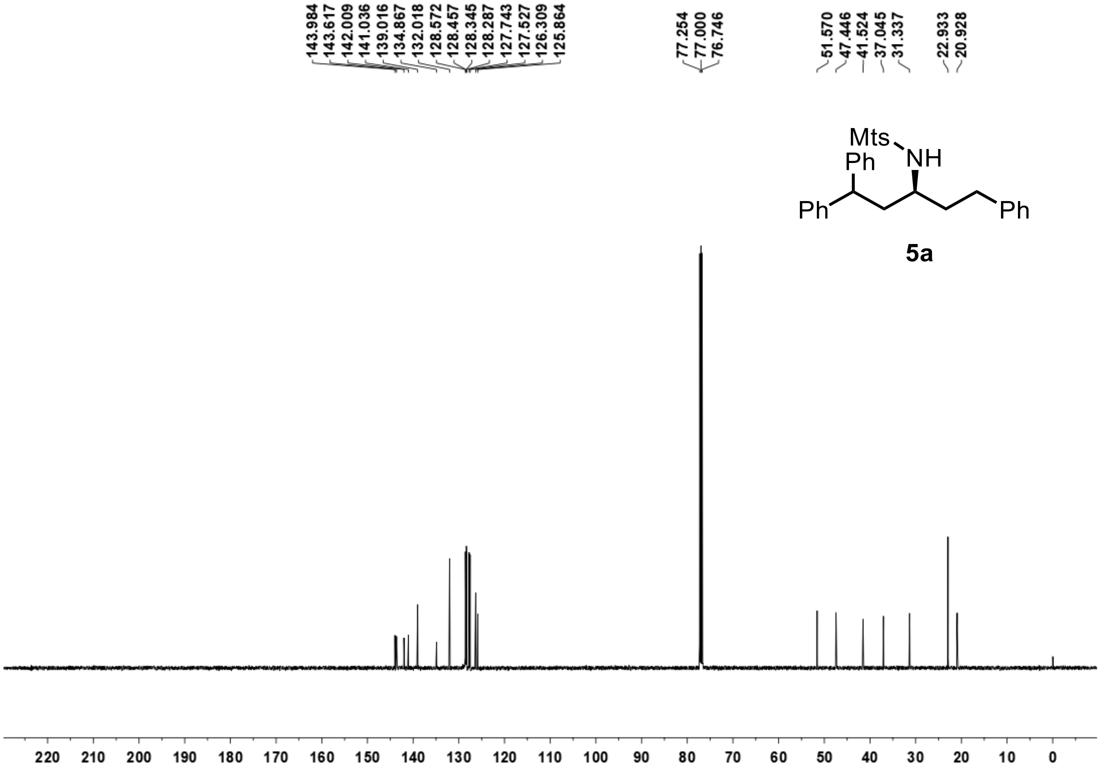

Supplementary Fig. 320 ^{13}C NMR (125 MHz, $CDCl_3$) of **5a**

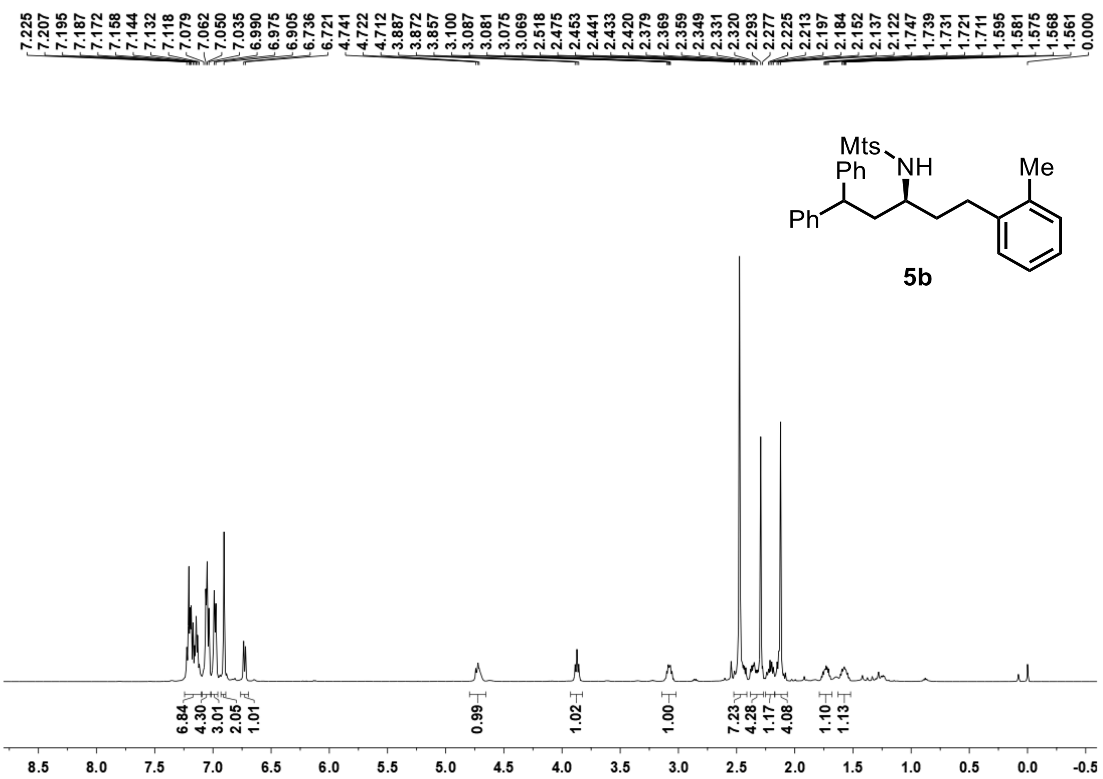

Supplementary Fig. 321 ^{1}H NMR (500 MHz, $CDCl_3$) of **5b**

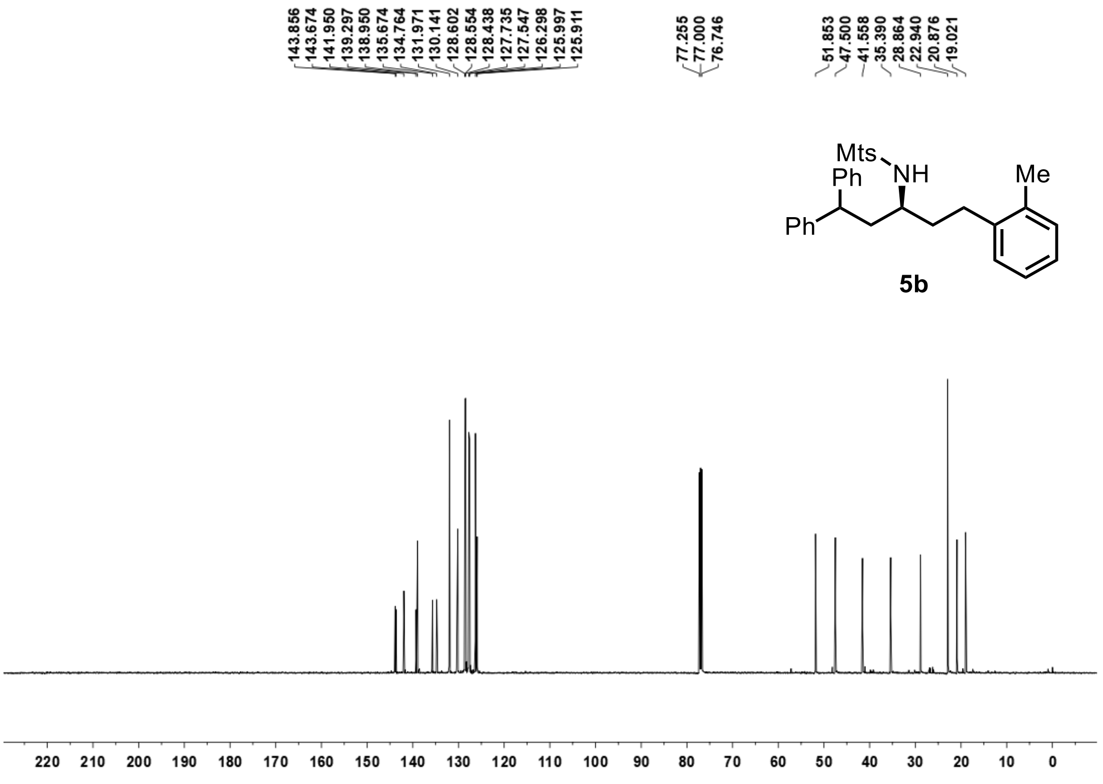

Supplementary Fig. 322 ^{13}C NMR (125 MHz, $CDCl_3$) of **5b**

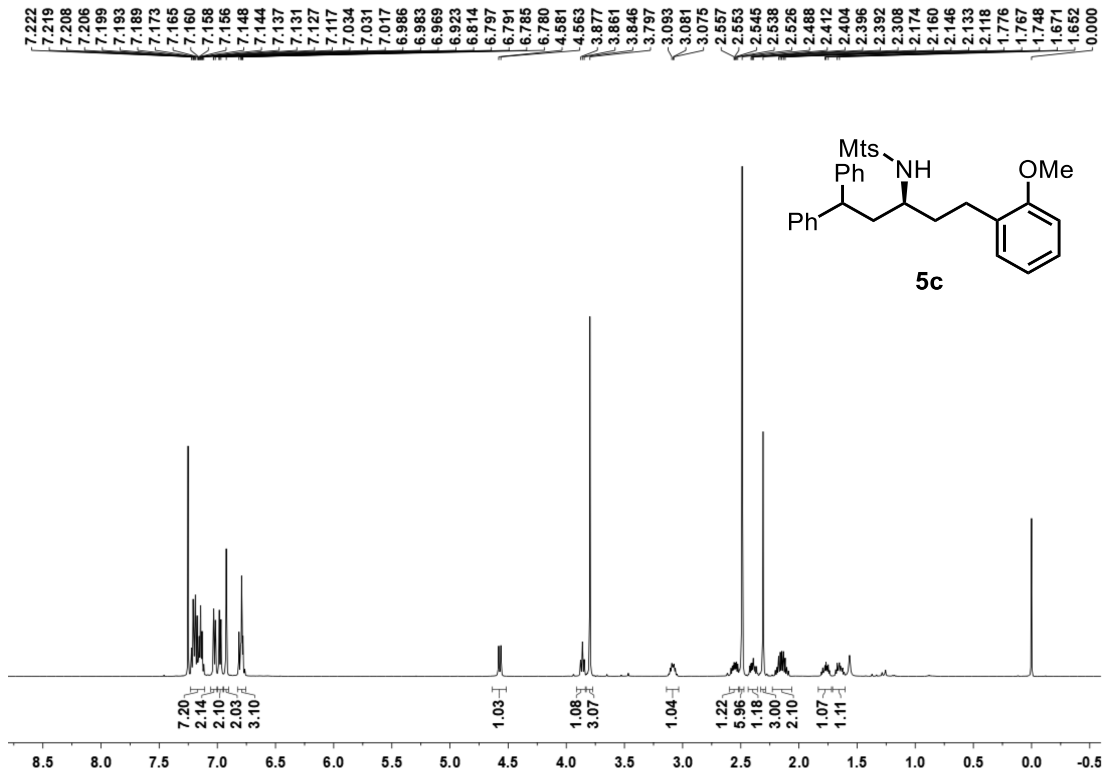

Supplementary Fig. 323 ^{1}H NMR (500 MHz, $CDCl_3$) of **5c**

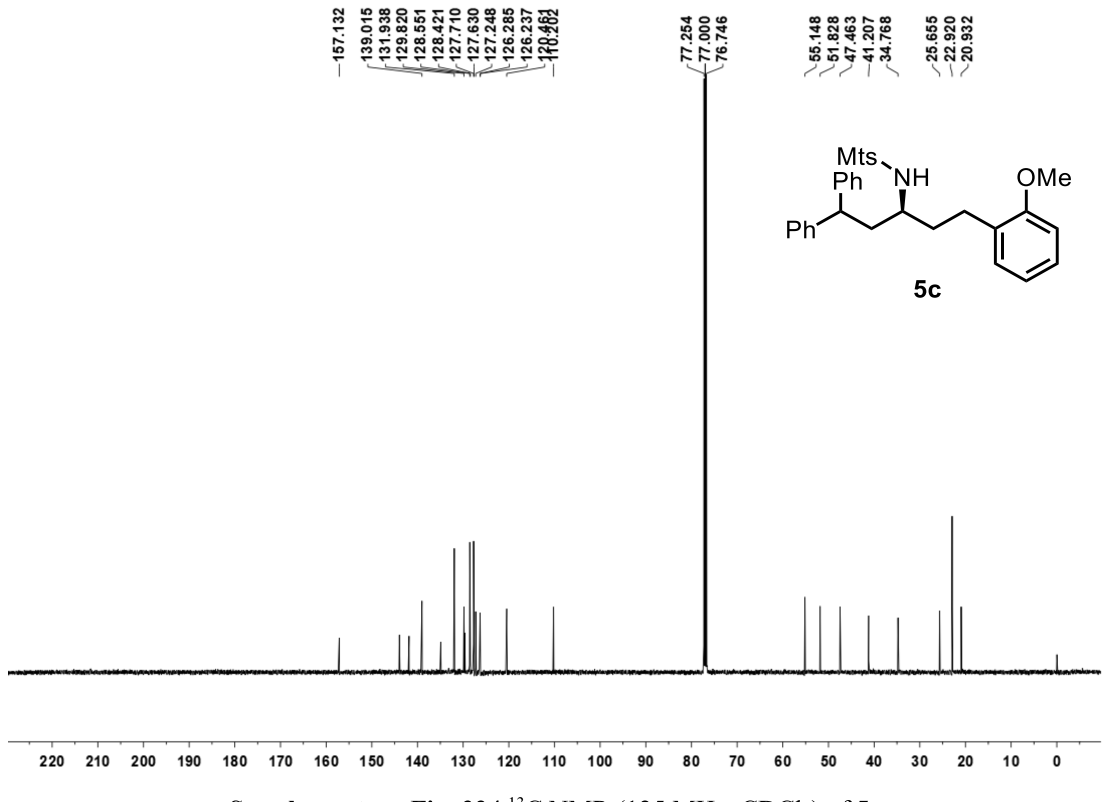

Supplementary Fig. 324 ^{13}C NMR (125 MHz, $CDCl_3$) of **5c**

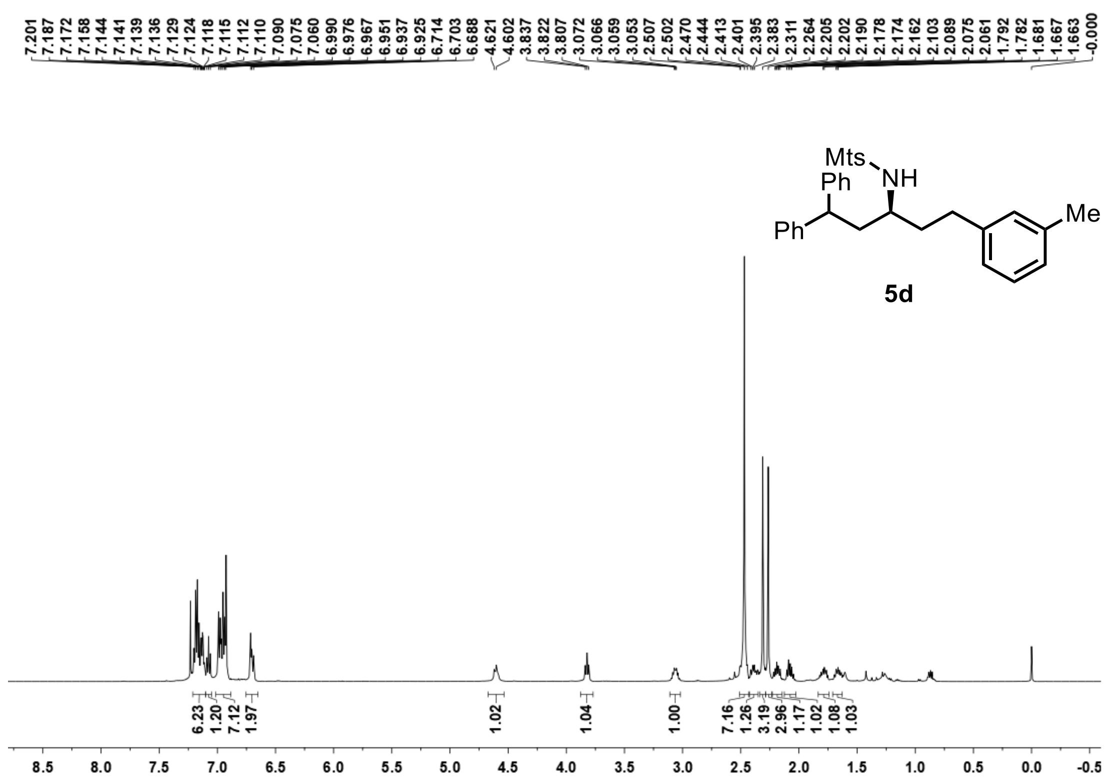

Supplementary Fig. 325 ^{1}H NMR (500 MHz, $CDCl_3$) of **5d**

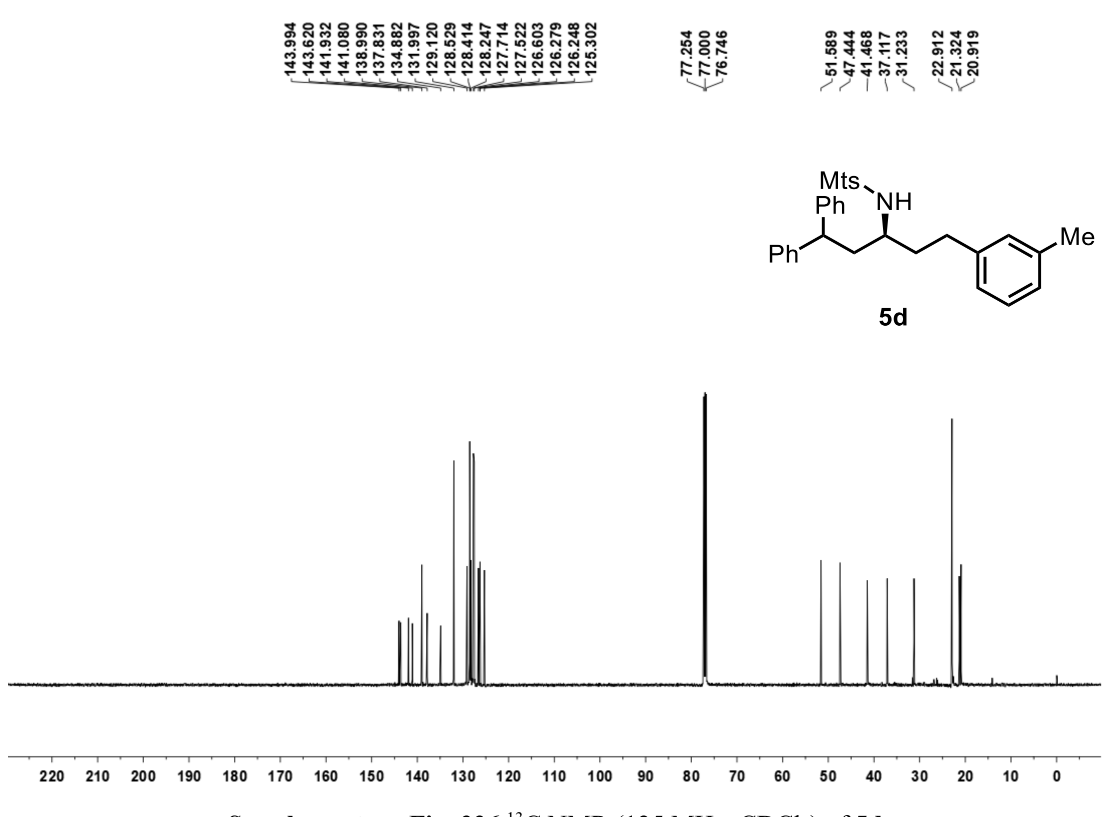

Supplementary Fig. 326 ^{13}C NMR (125 MHz, $CDCl_3$) of **5d**

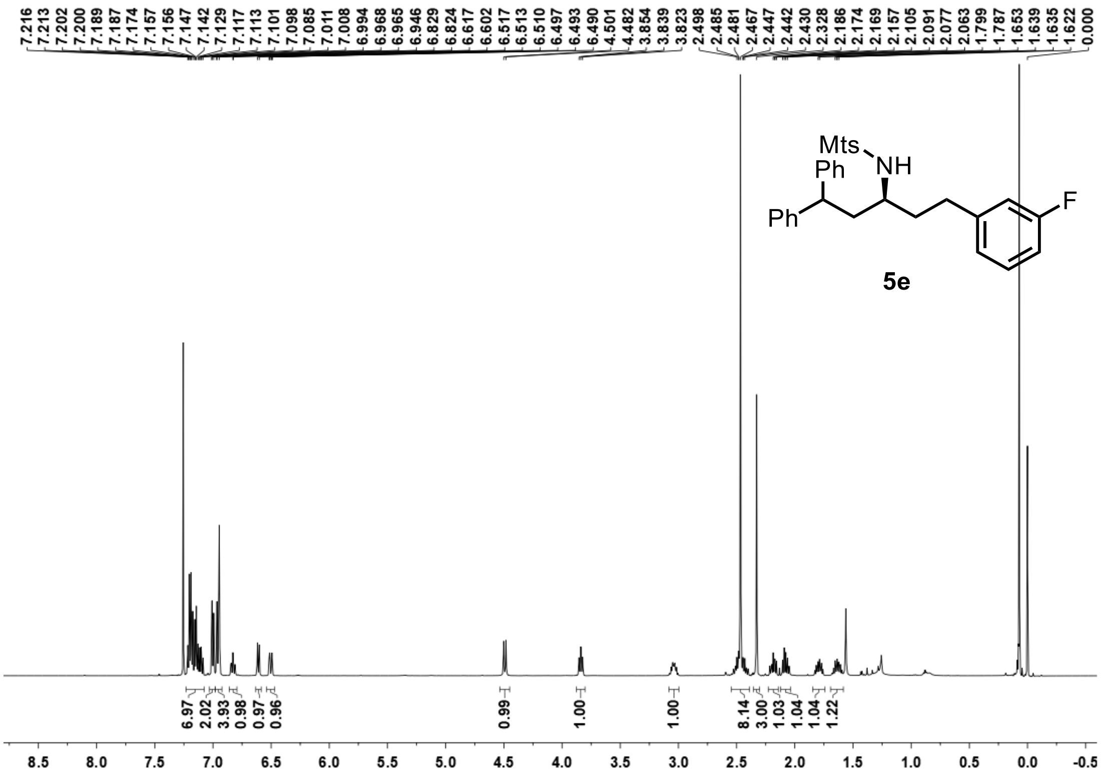

Supplementary Fig. 327 ^{1}H NMR (500 MHz, $CDCl_3$) of **5e**

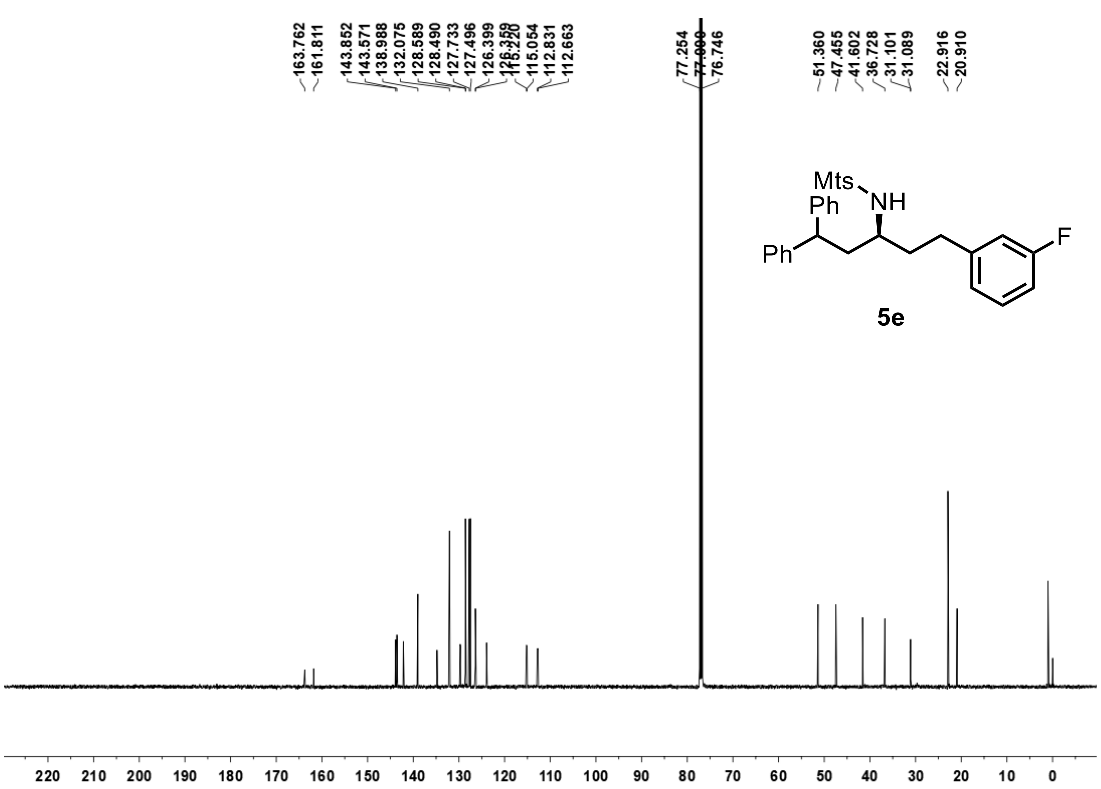

Supplementary Fig. 328 ^{13}C NMR (125 MHz, $CDCl_3$) of **5e**

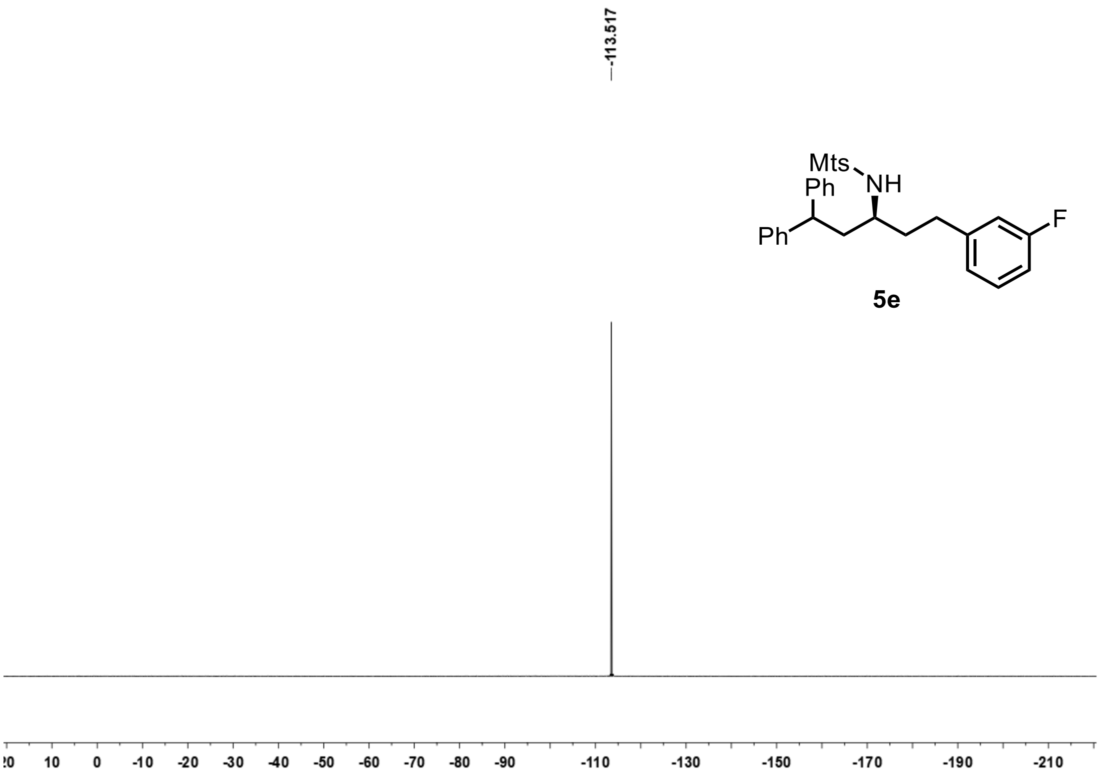

Supplementary Fig. 329 ^{19}F NMR (470 MHz, $CDCl_3$) of **5e**

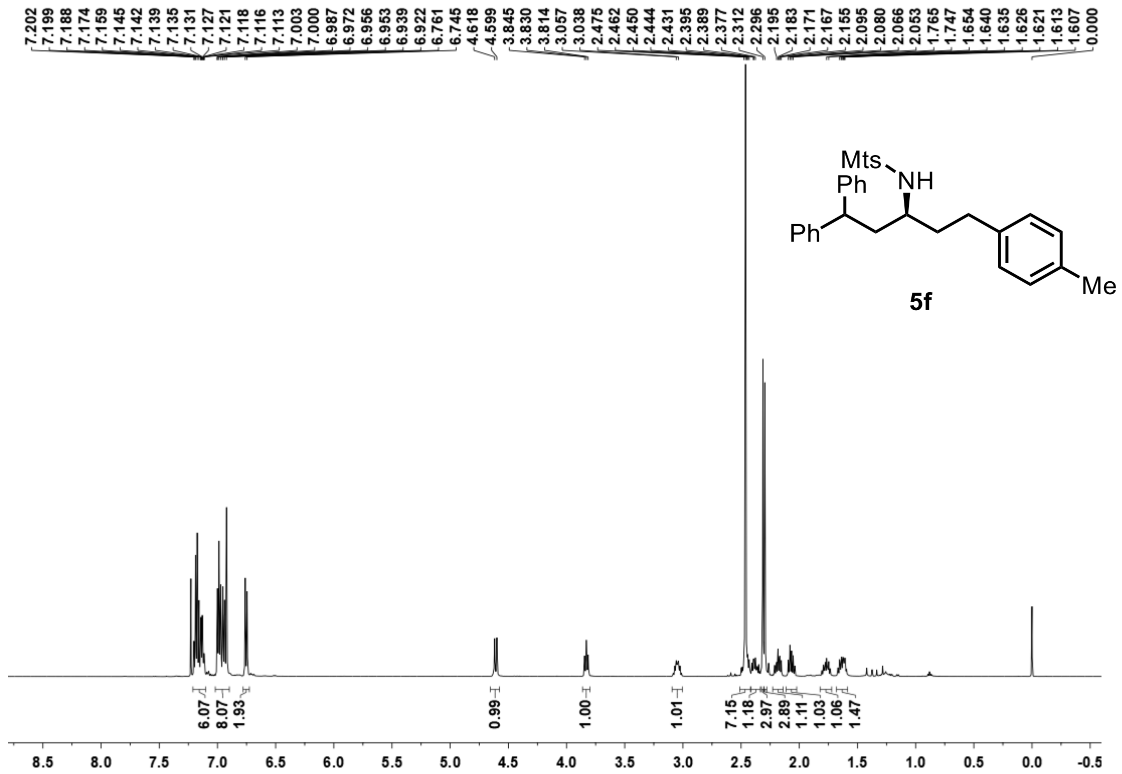

Supplementary Fig. 330 ^{1}H NMR (500 MHz, $CDCl_3$) of **5f**

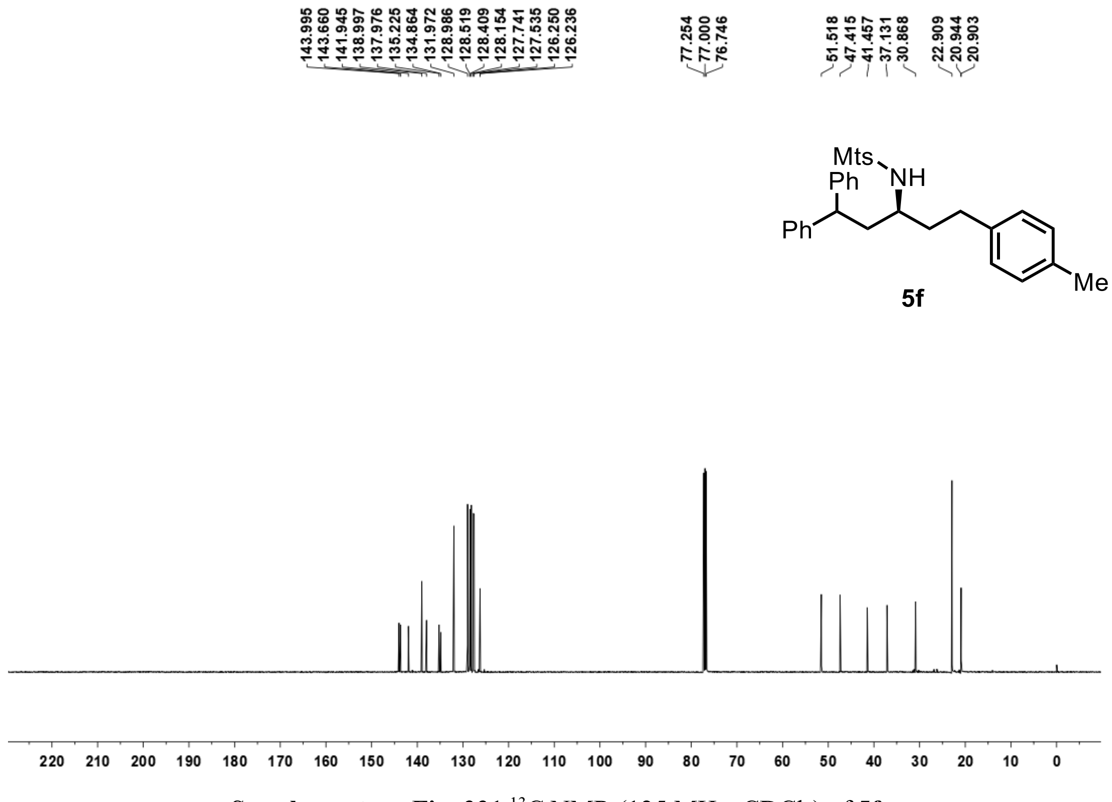

Supplementary Fig. 331 ^{13}C NMR (125 MHz, $CDCl_3$) of **5f**

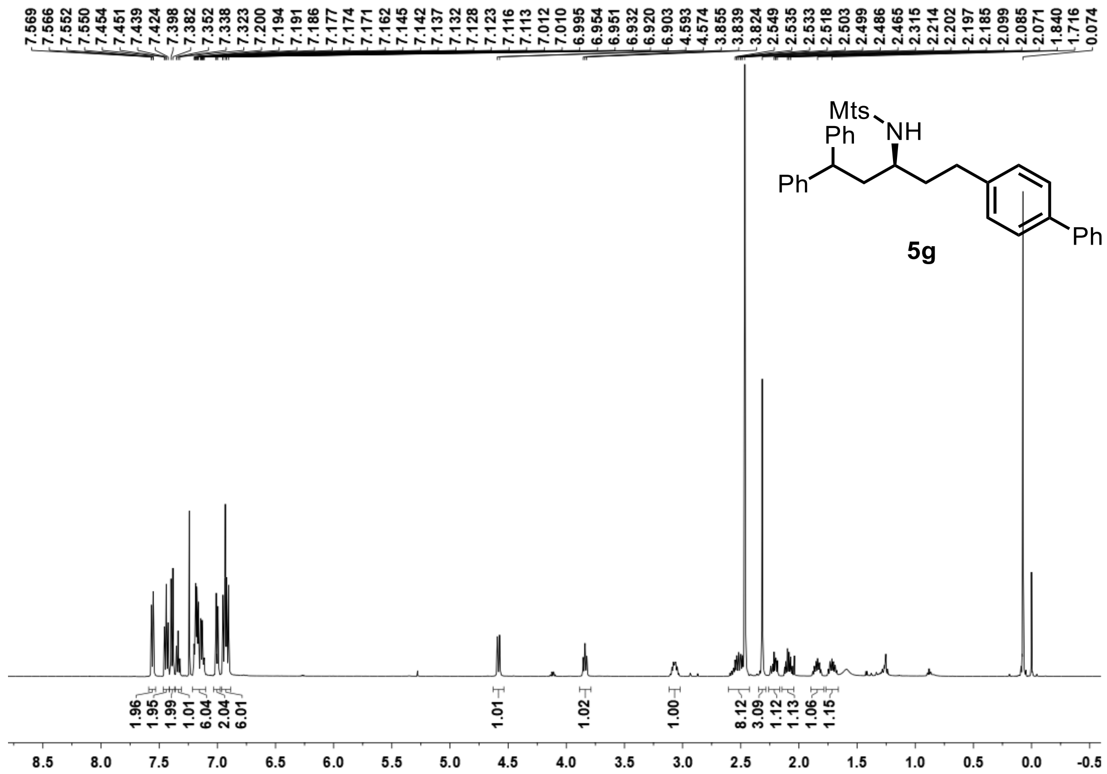

Supplementary Fig. 332 ^{1}H NMR (500 MHz, $CDCl_3$) of **5g**

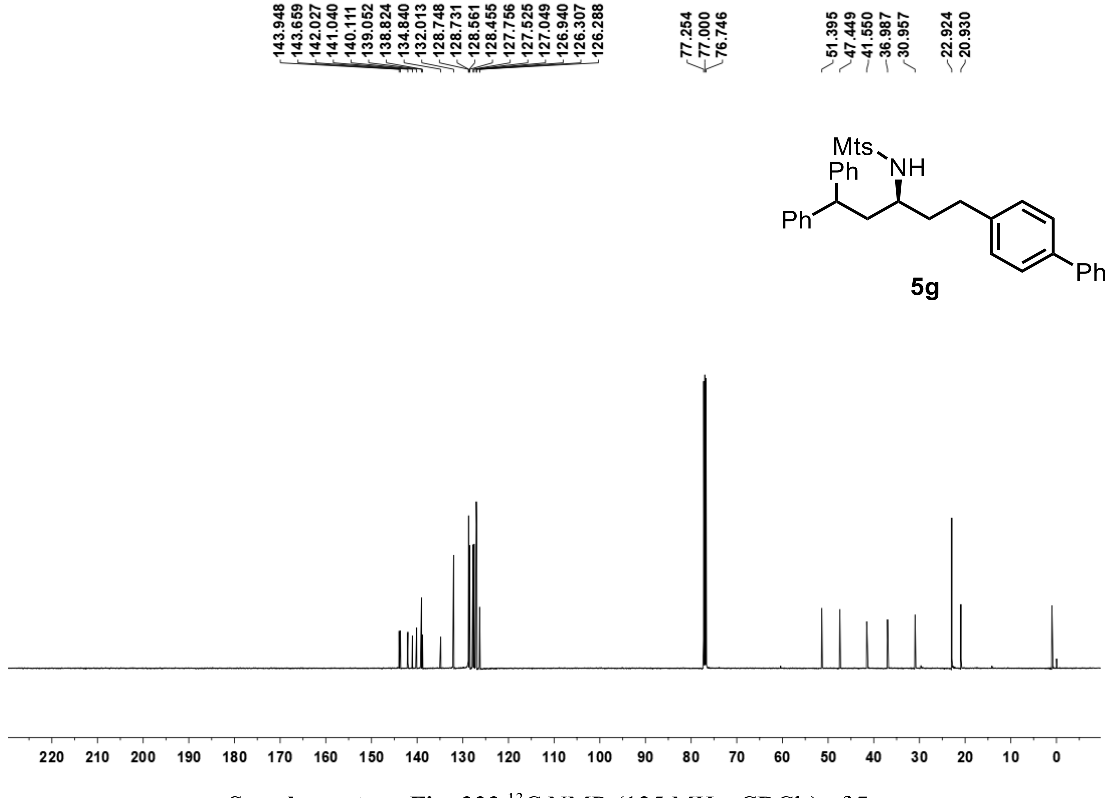

Supplementary Fig. 333 ^{13}C NMR (125 MHz, $CDCl_3$) of **5g**

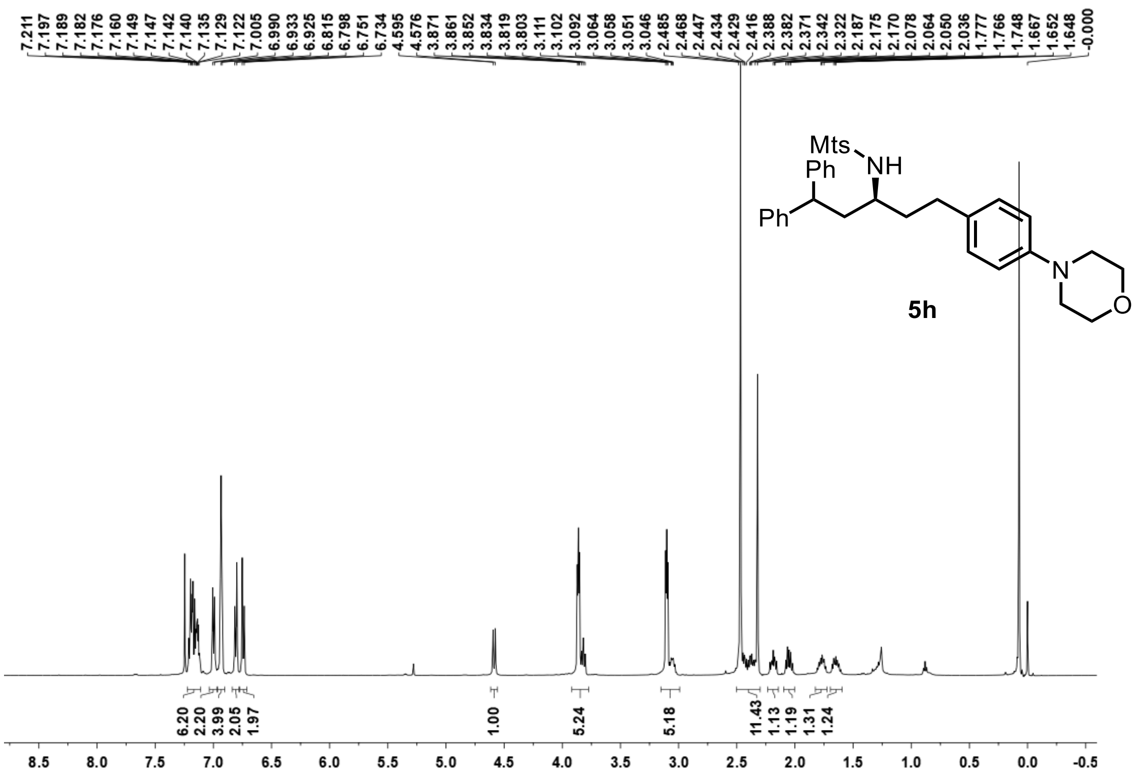

Supplementary Fig. 334 ^{1}H NMR (500 MHz, $CDCl_3$) of **5h**

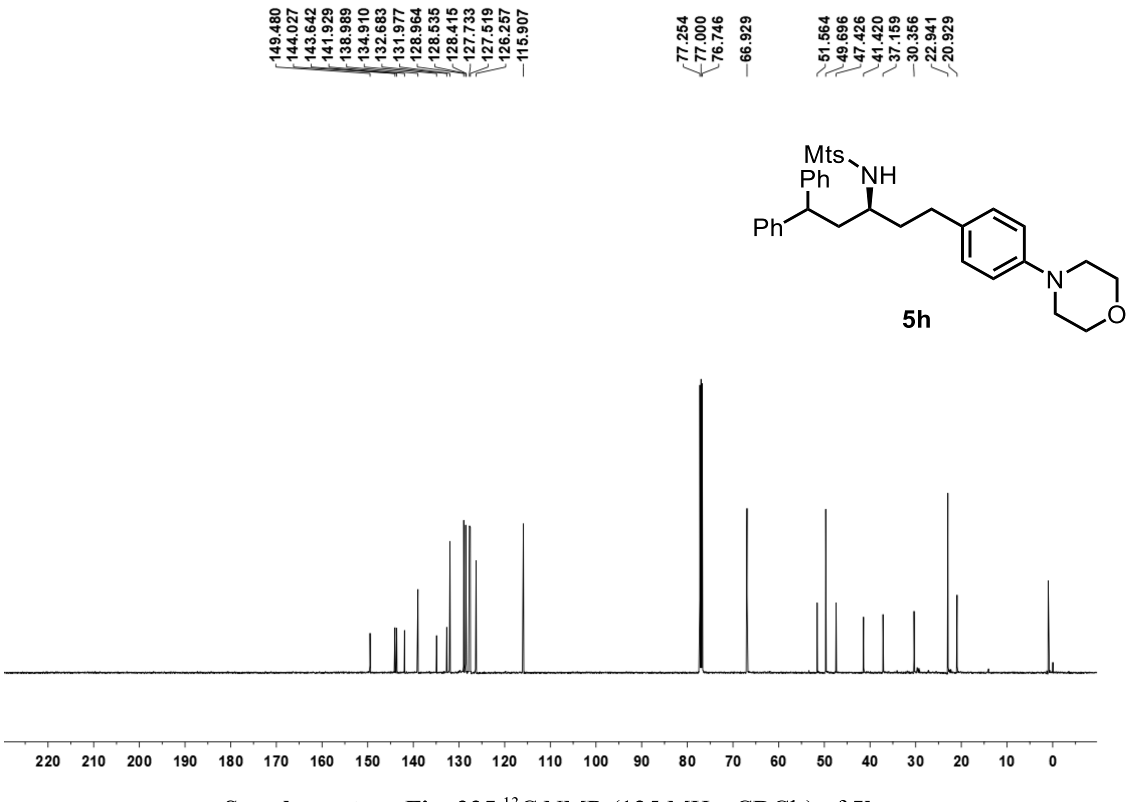

Supplementary Fig. 335 ^{13}C NMR (125 MHz, $CDCl_3$) of **5h**

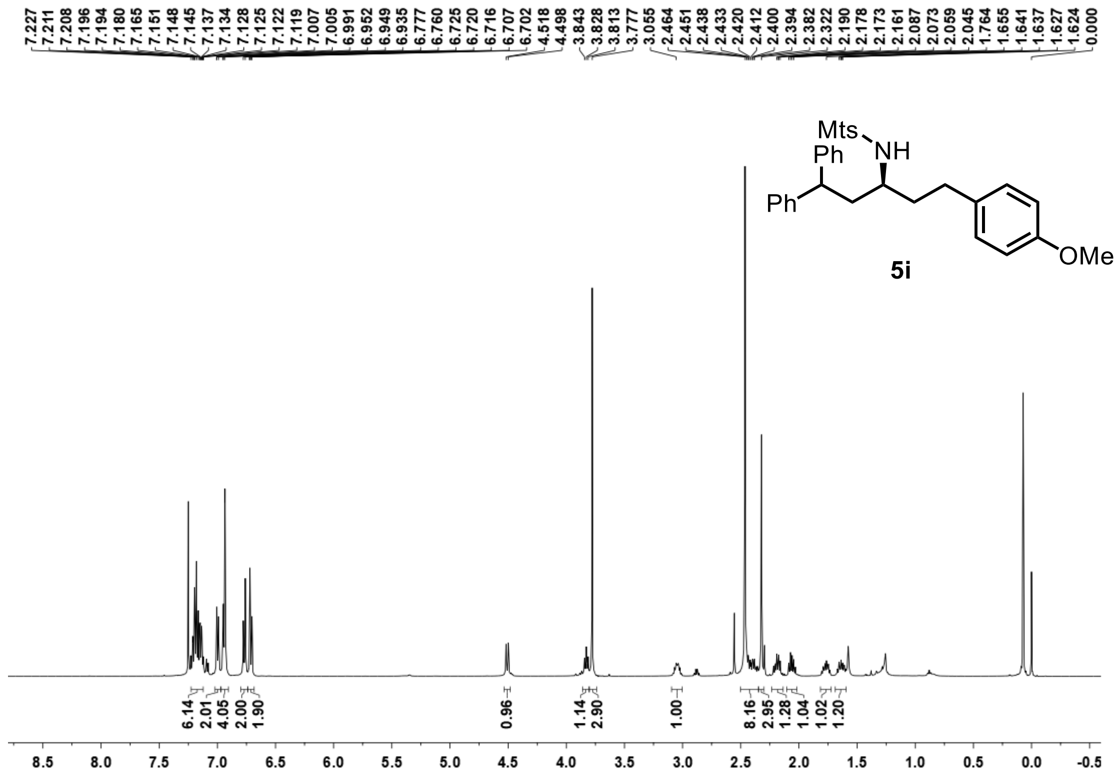

Supplementary Fig. 336 ^{1}H NMR (500 MHz, $CDCl_3$) of **5i**

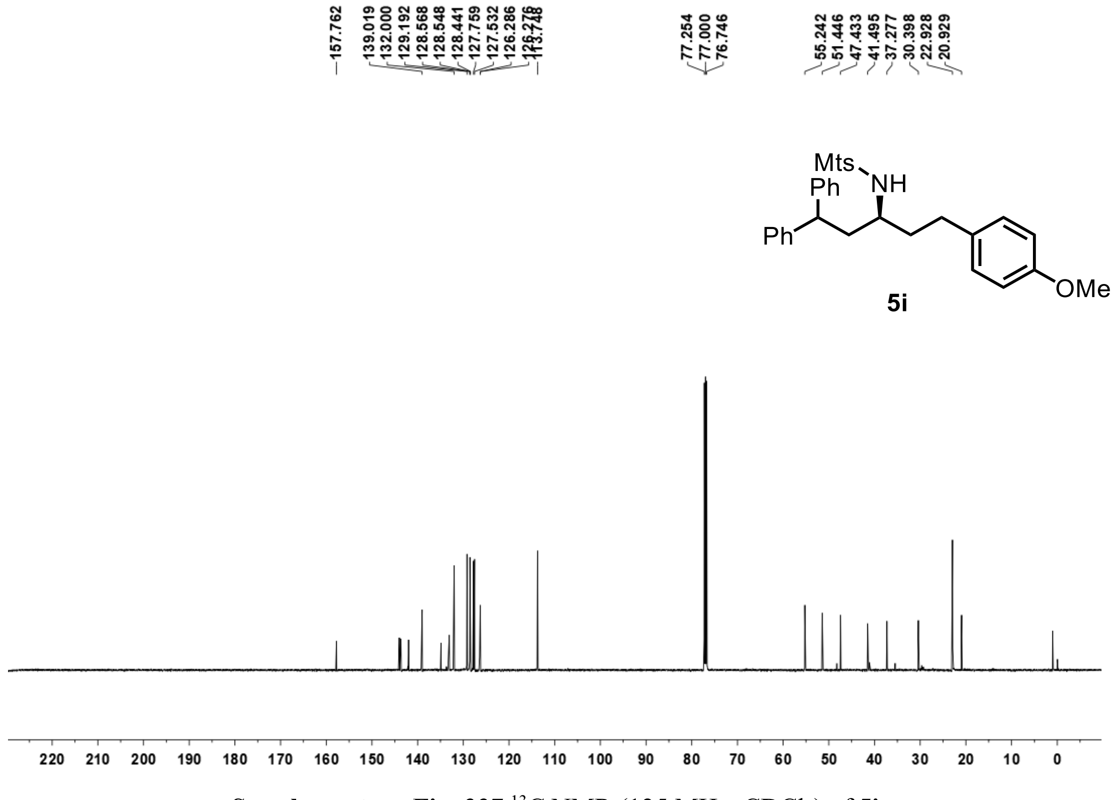

Supplementary Fig. 337 ^{13}C NMR (125 MHz, $CDCl_3$) of **5i**

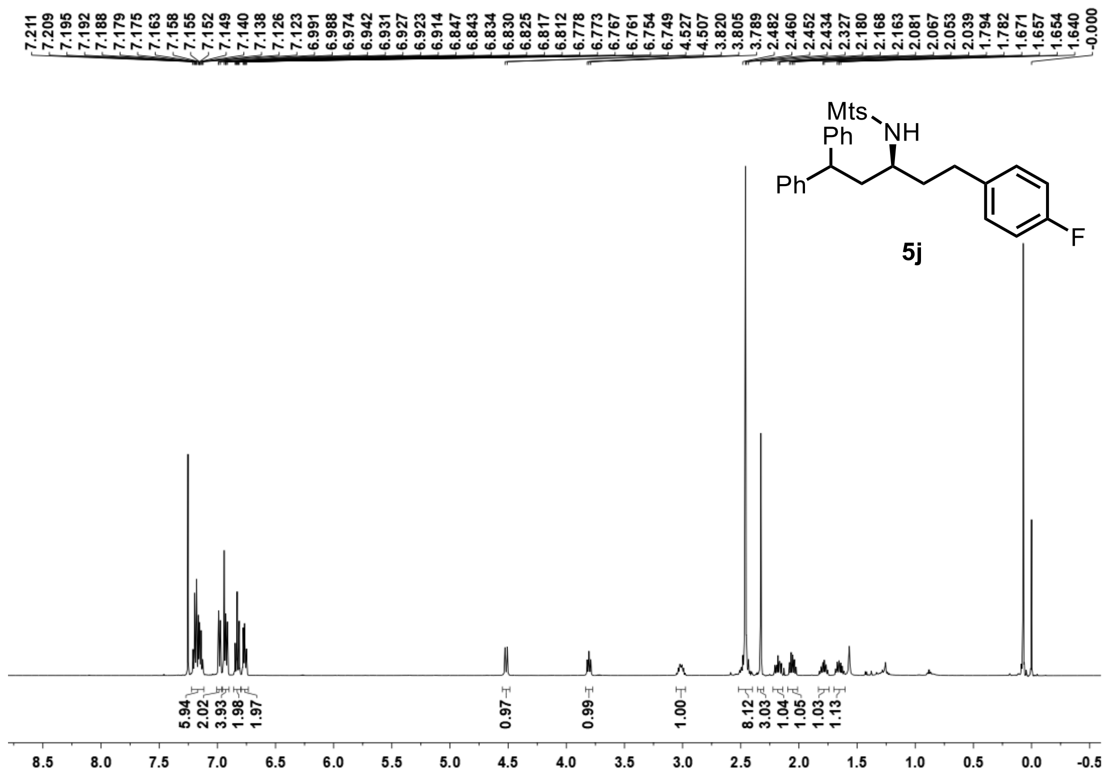

Supplementary Fig. 338 ^{1}H NMR (500 MHz, $CDCl_3$) of **5j**

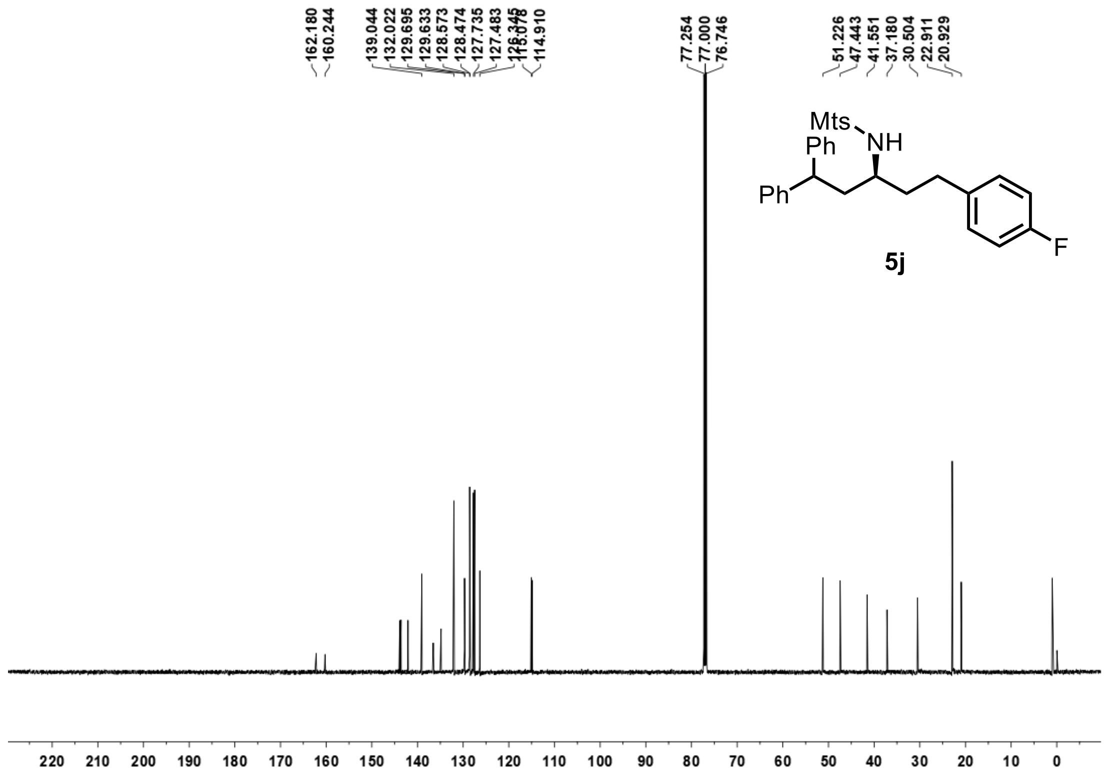

Supplementary Fig. 339 ^{13}C NMR (125 MHz, $CDCl_3$) of **5j**

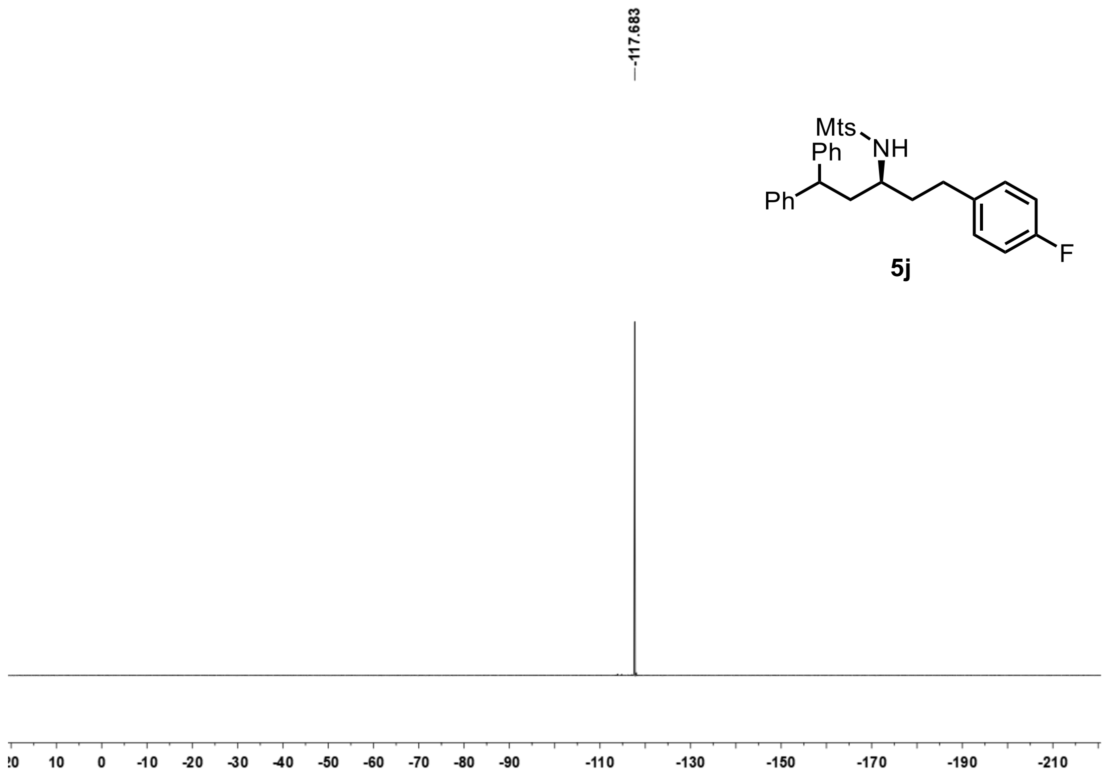

Supplementary Fig. 340 ^{19}F NMR (470 MHz, $CDCl_3$) of **5j**

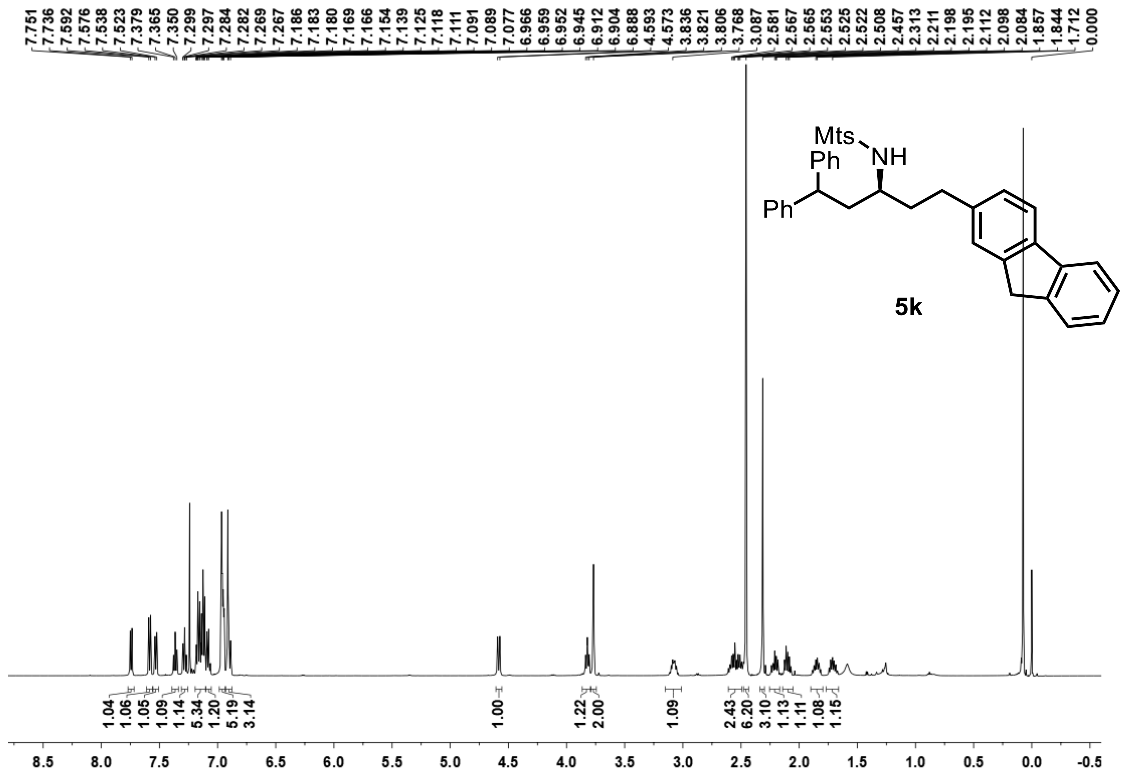

Supplementary Fig. 341 ^{1}H NMR (500 MHz, $CDCl_3$) of **5k**

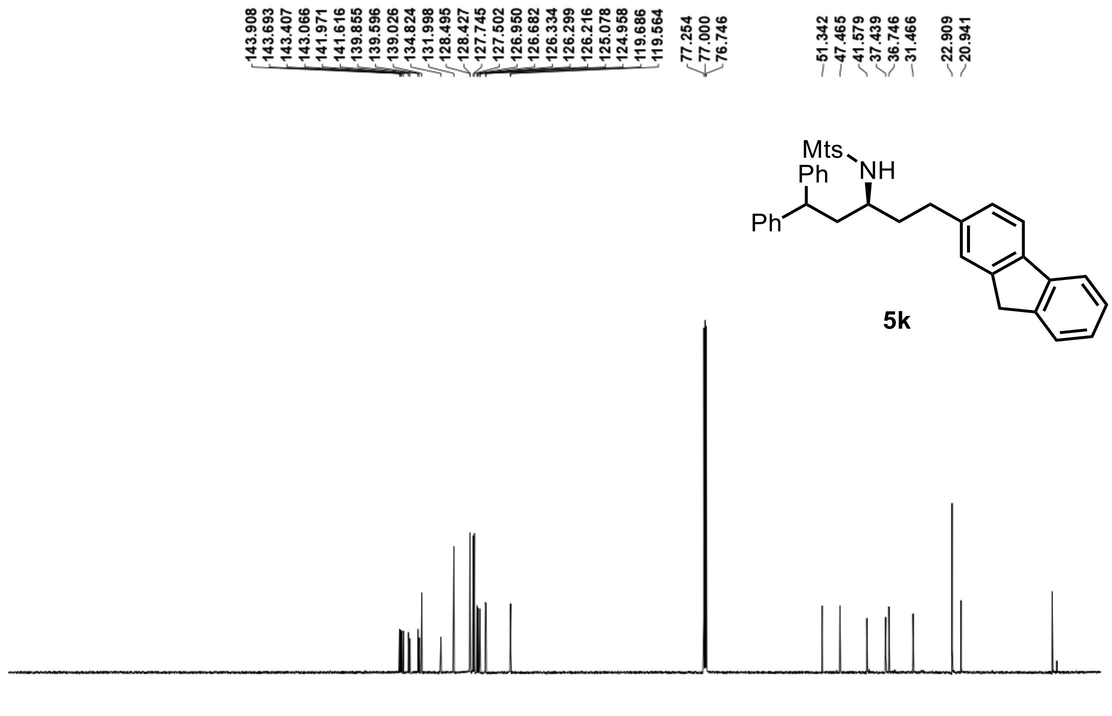

Supplementary Fig. 342 ^{13}C NMR (125 MHz, $CDCl_3$) of **5k**

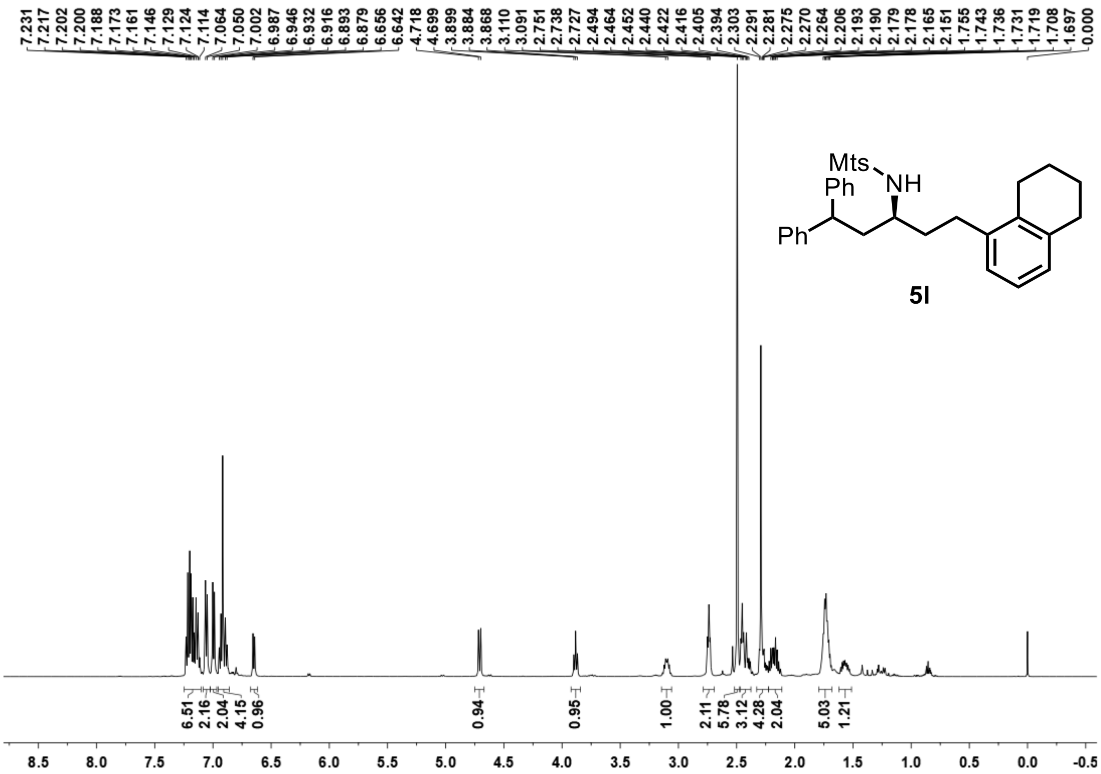

Supplementary Fig. 343 ^{1}H NMR (500 MHz, $CDCl_3$) of **5l**

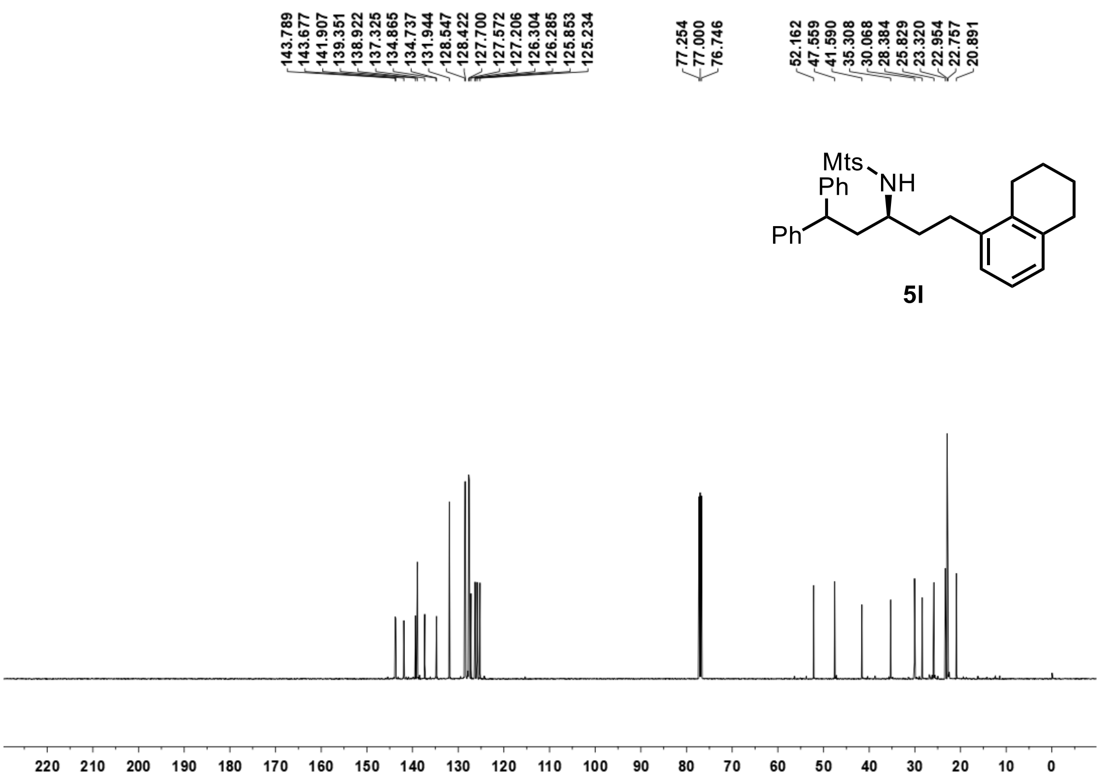

Supplementary Fig. 344 ^{13}C NMR (125 MHz, $CDCl_3$) of **5l**

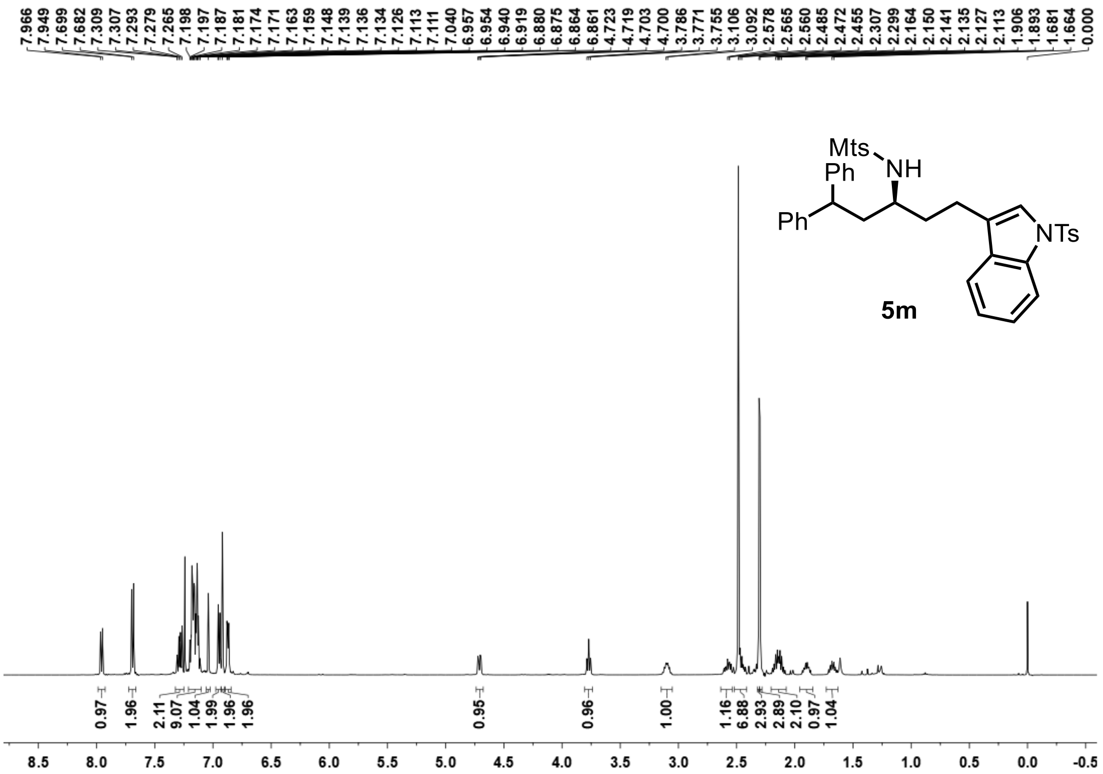

Supplementary Fig. 345 ^{1}H NMR (500 MHz, $CDCl_3$) of **5m**

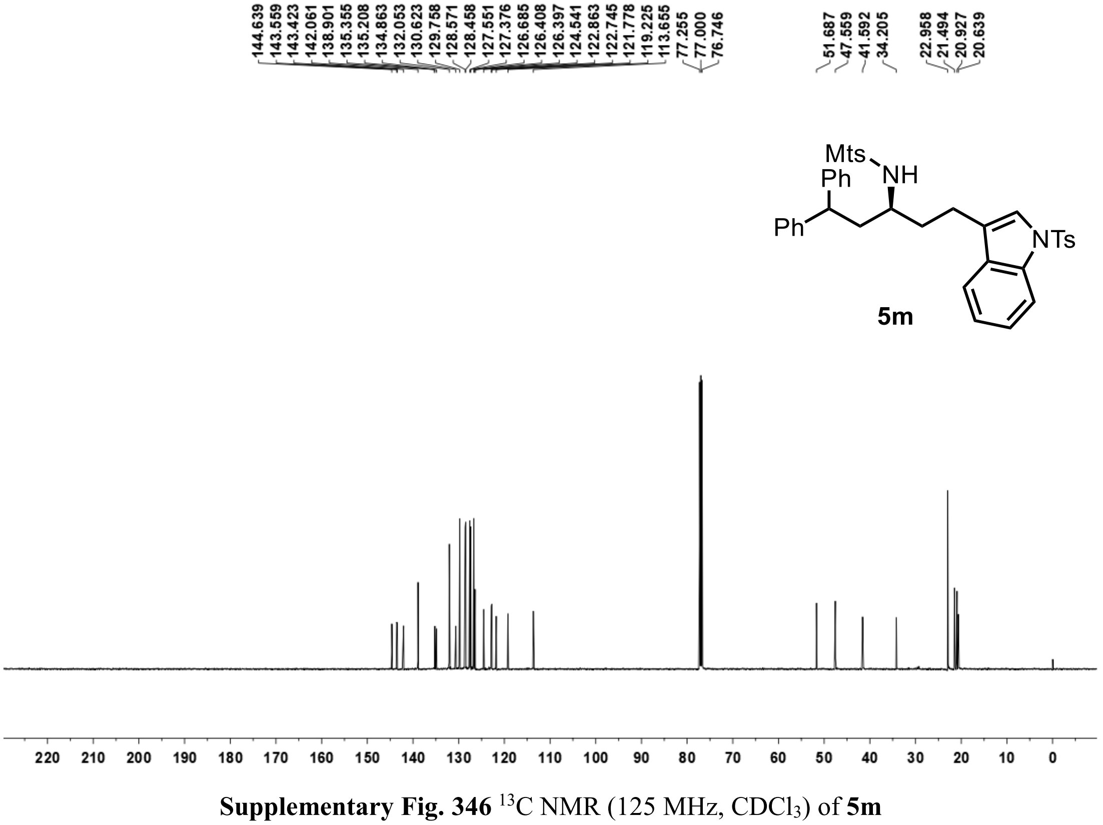

Supplementary Fig. 346 ^{13}C NMR (125 MHz, $CDCl_3$) of **5m**

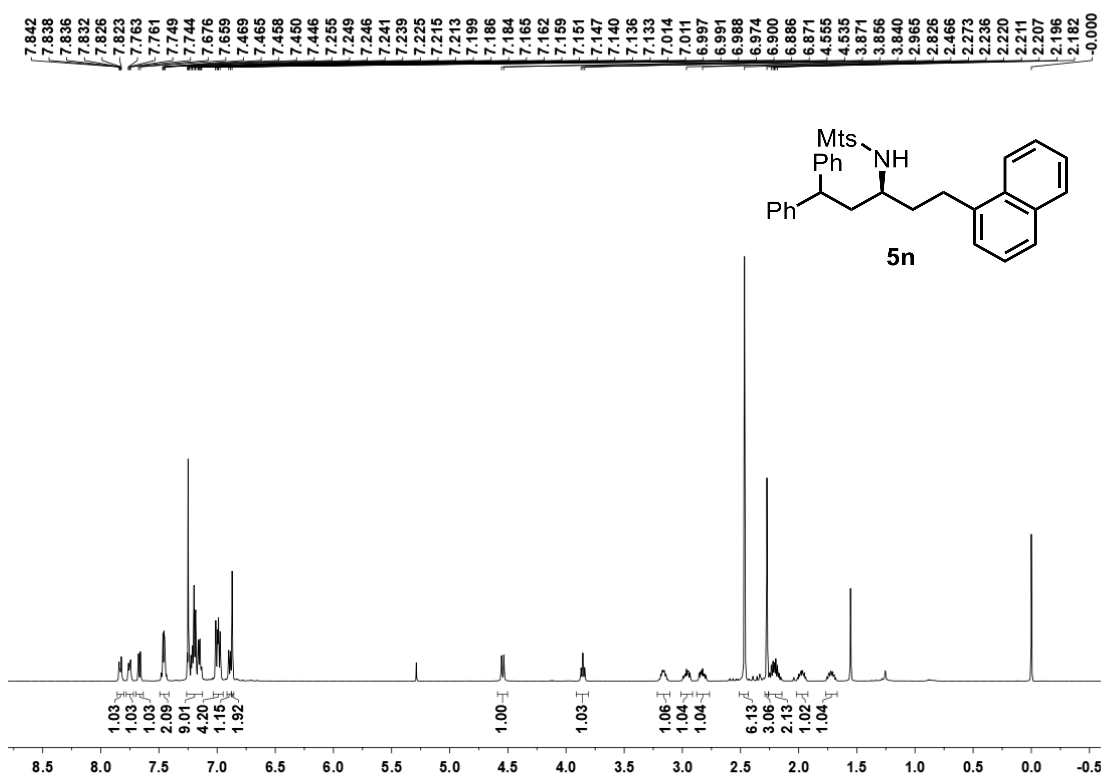

Supplementary Fig. 347 ^{1}H NMR (500 MHz, $CDCl_3$) of **5n**

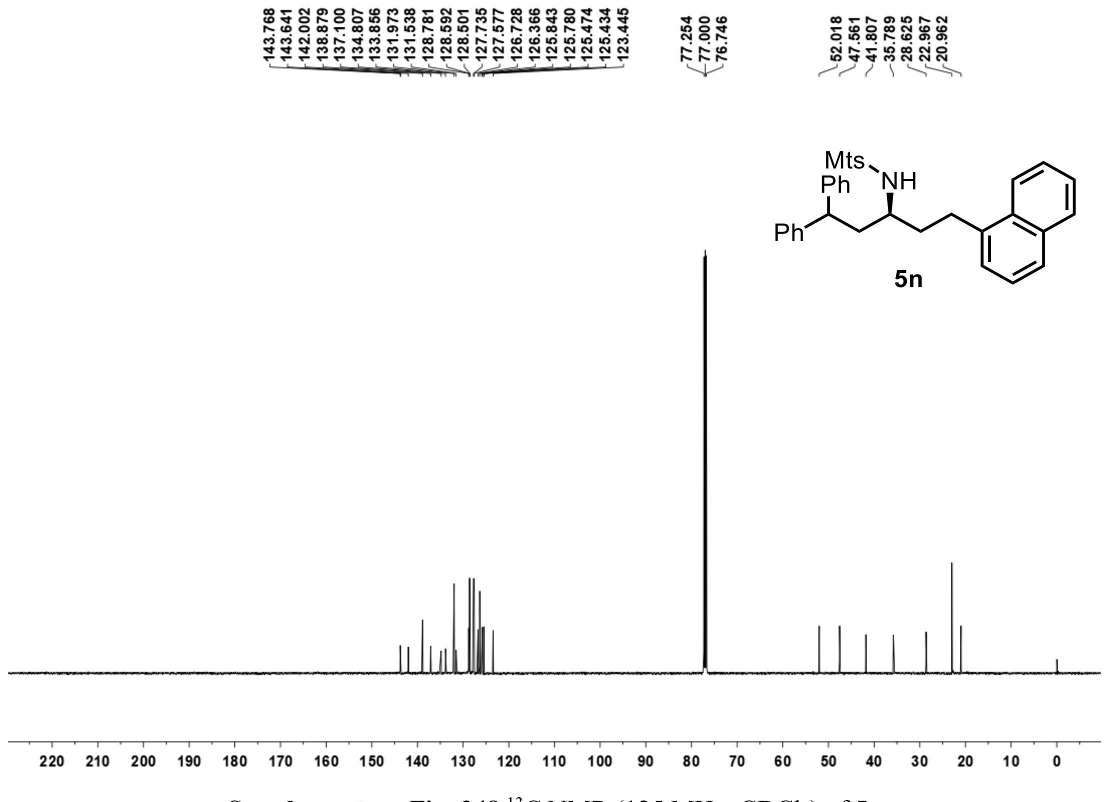

Supplementary Fig. 348 ^{13}C NMR (125 MHz, $CDCl_3$) of **5n**

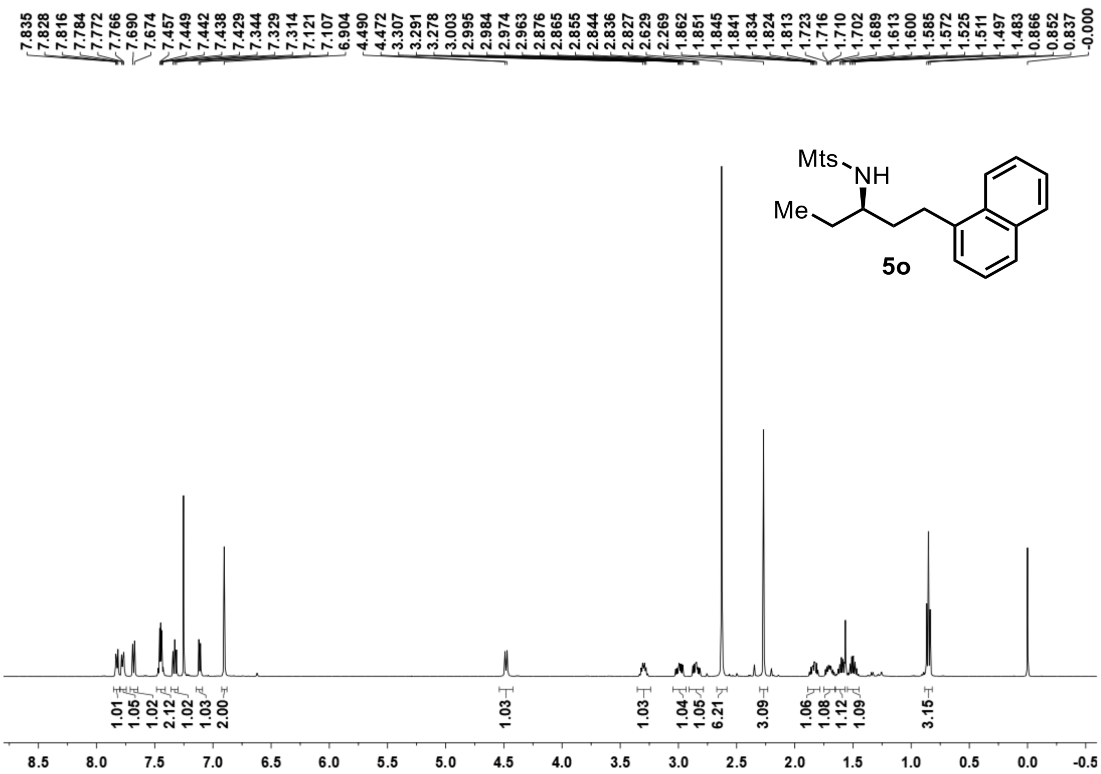

Supplementary Fig. 349 ^{1}H NMR (500 MHz, $CDCl_3$) of **5o**

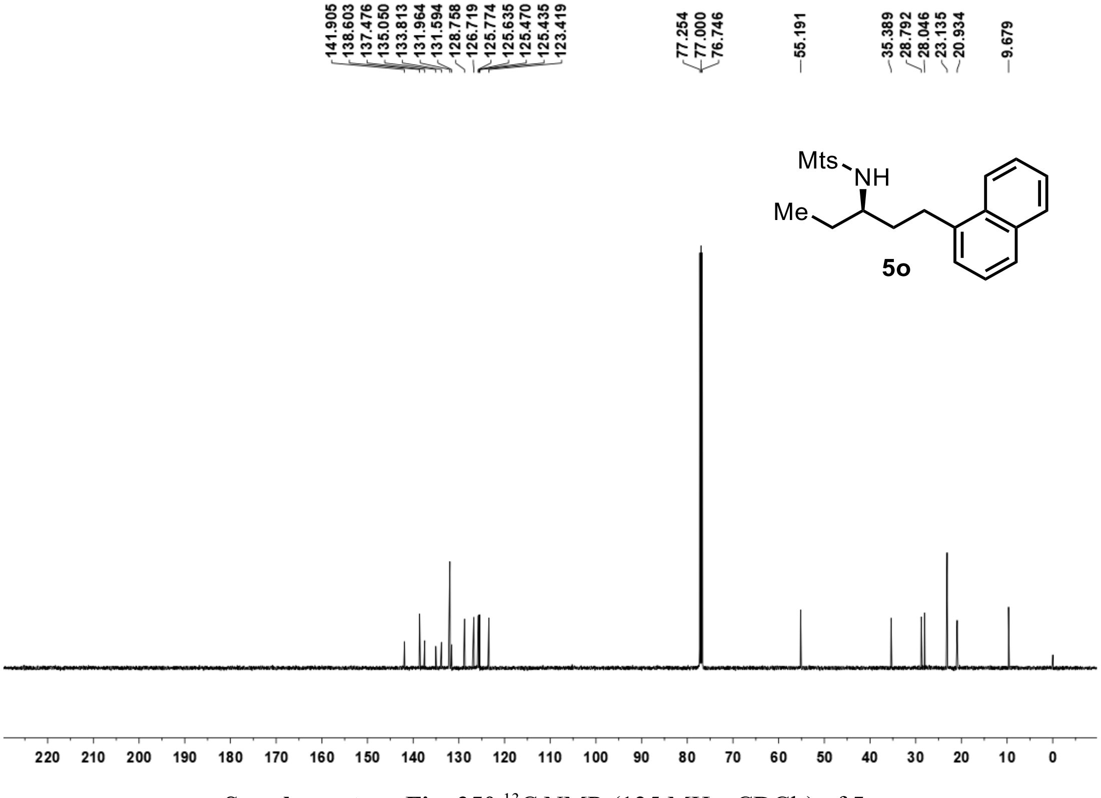

Supplementary Fig. 350 ^{13}C NMR (125 MHz, $CDCl_3$) of **5o**

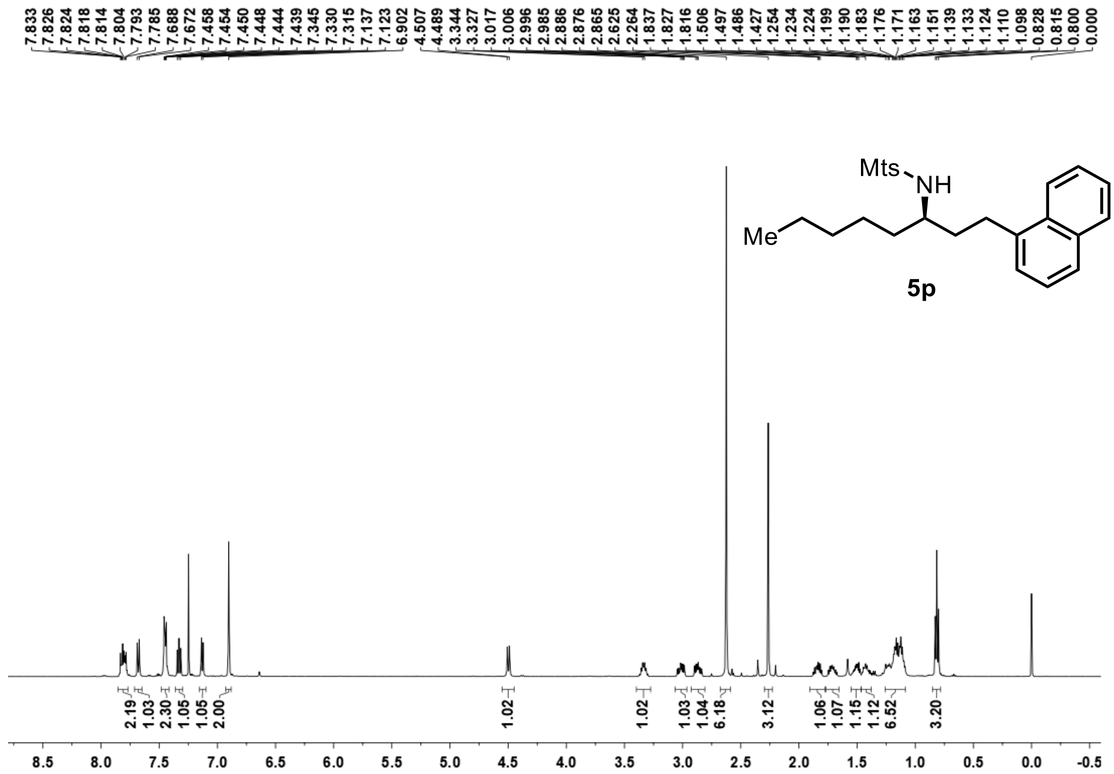

Supplementary Fig. 351 ^{1}H NMR (500 MHz, $CDCl_3$) of **5p**

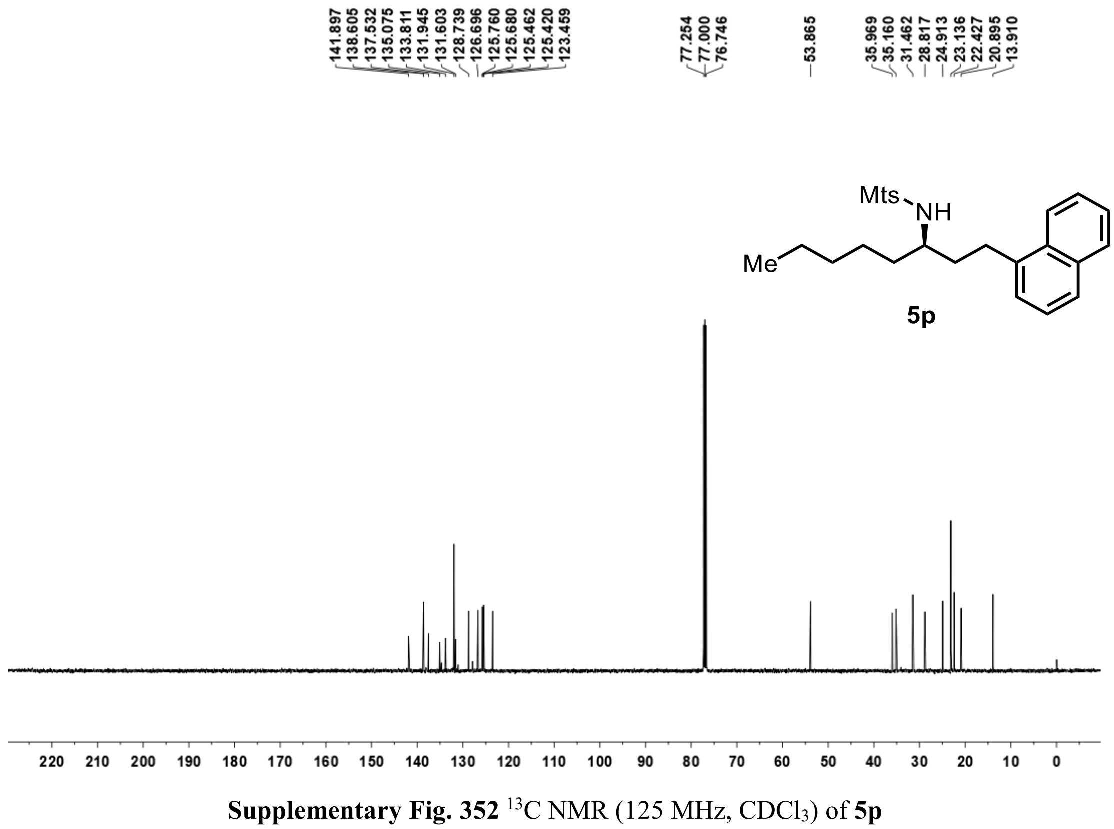

Supplementary Fig. 352 ^{13}C NMR (125 MHz, $CDCl_3$) of **5p**

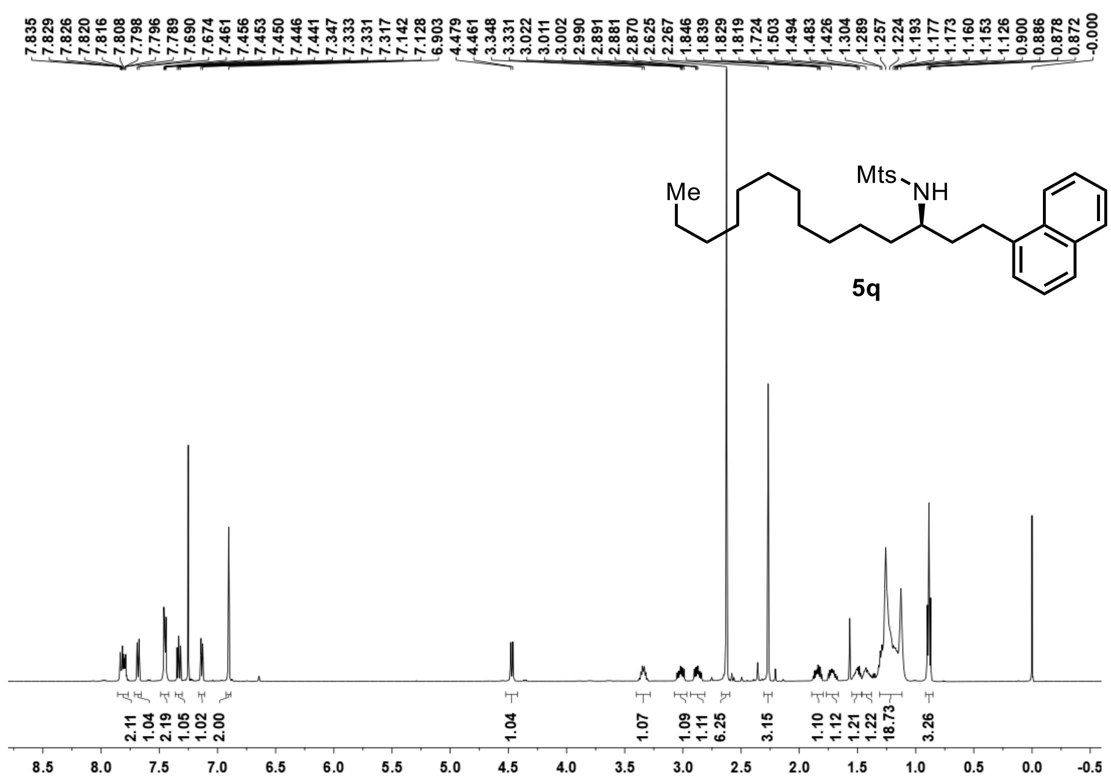

Supplementary Fig. 353 ^{1}H NMR (500 MHz, $CDCl_3$) of **5q**

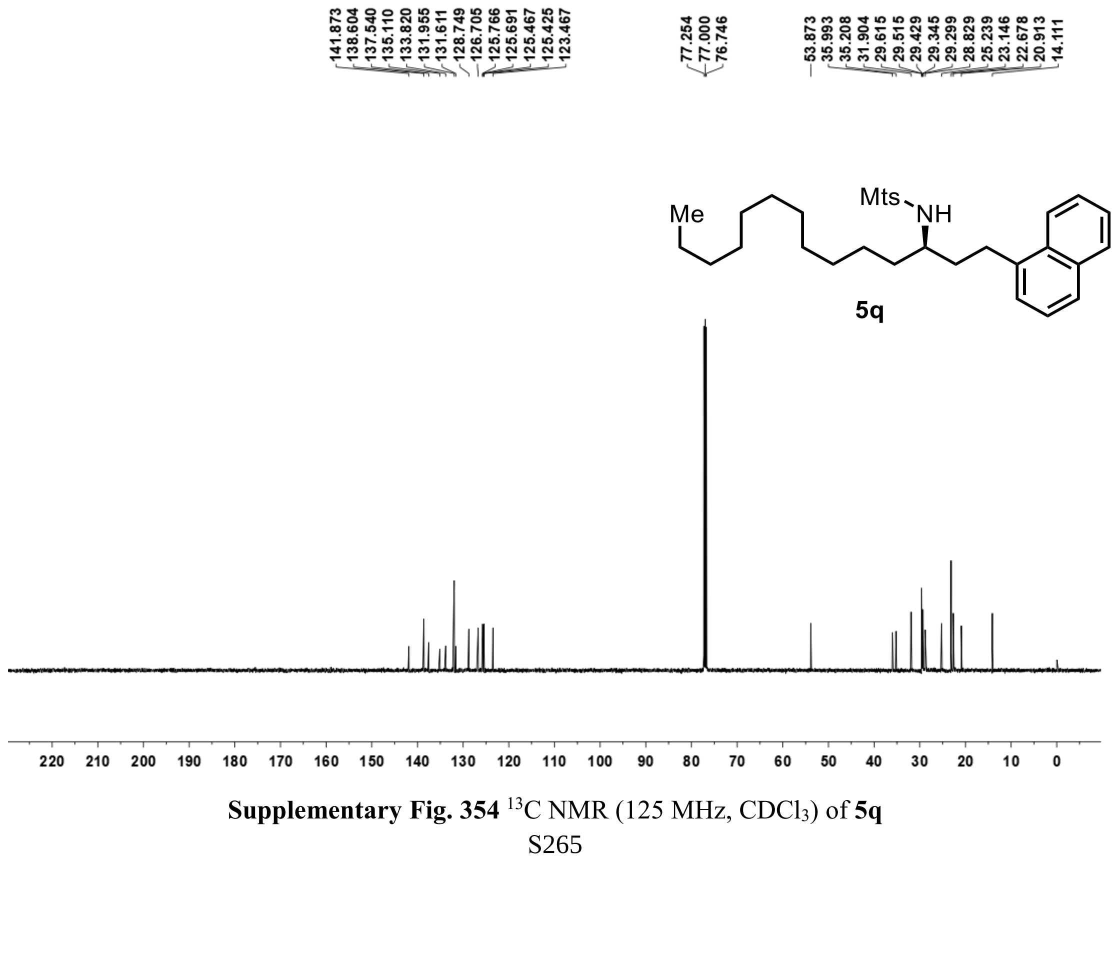

Supplementary Fig. 354 ^{13}C NMR (125 MHz, $CDCl_3$) of **5q**

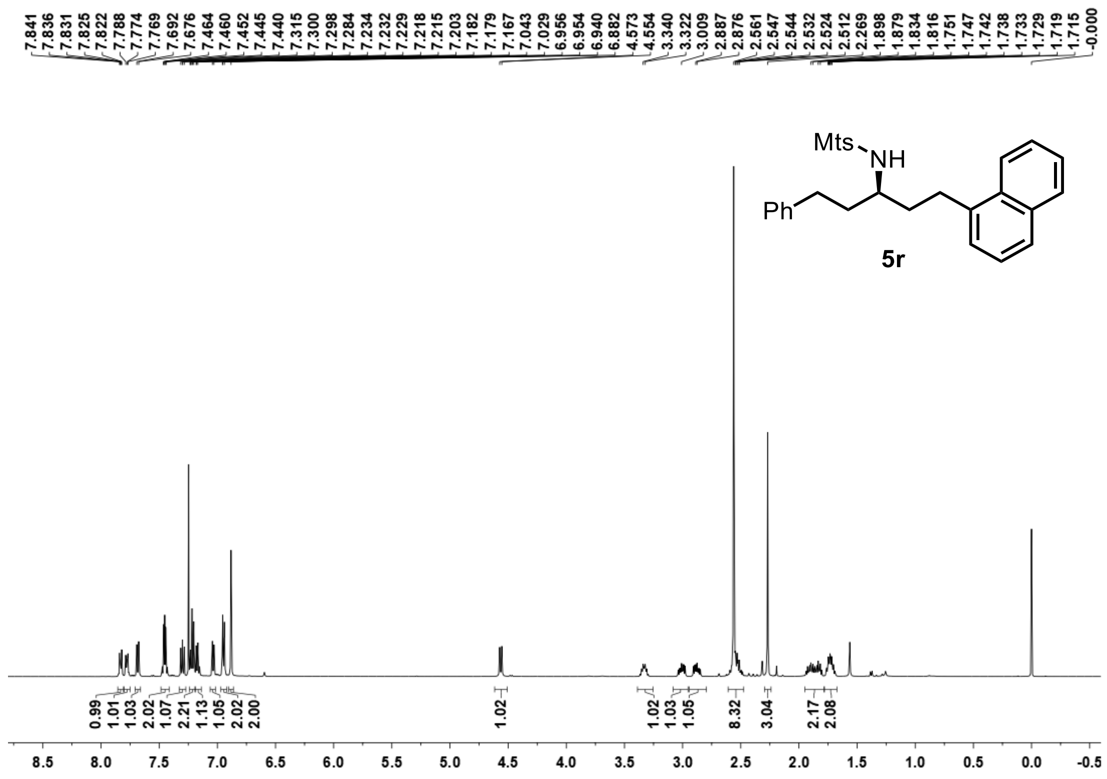

Supplementary Fig. 355 ^{1}H NMR (500 MHz, $CDCl_3$) of **5r**

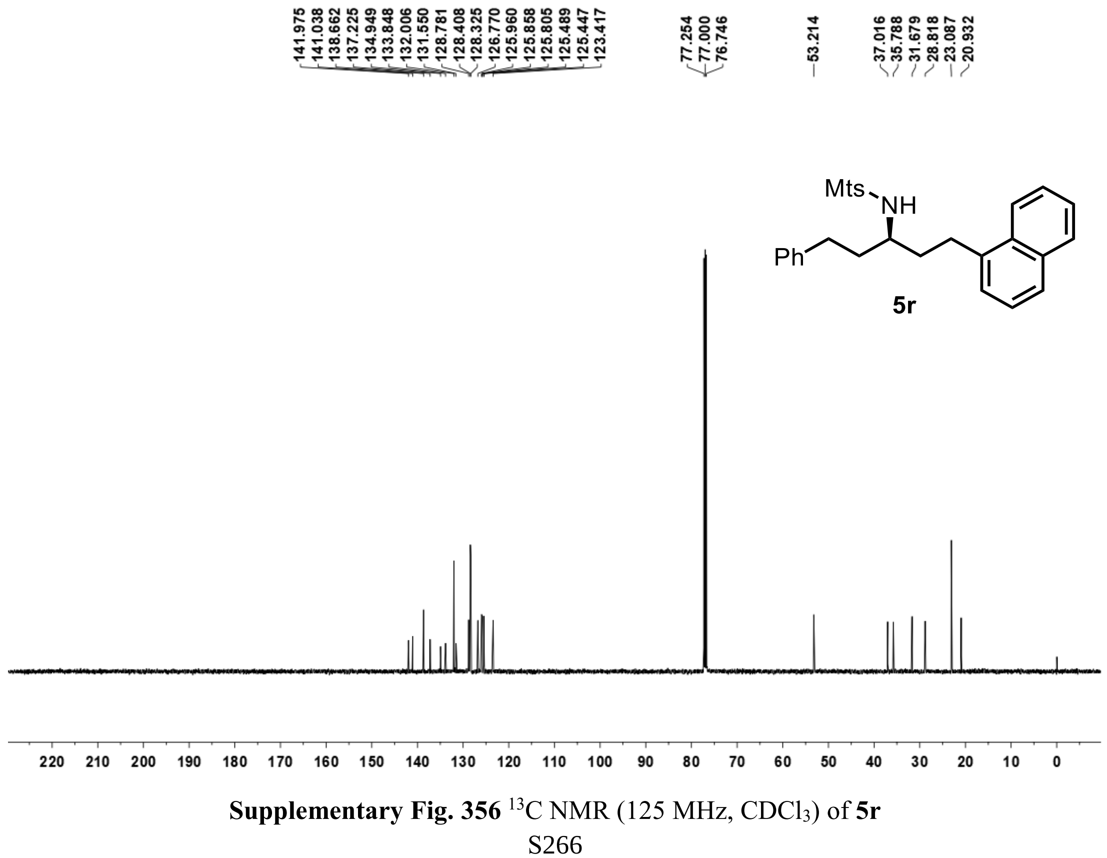

Supplementary Fig. 356 ^{13}C NMR (125 MHz, $CDCl_3$) of **5r**

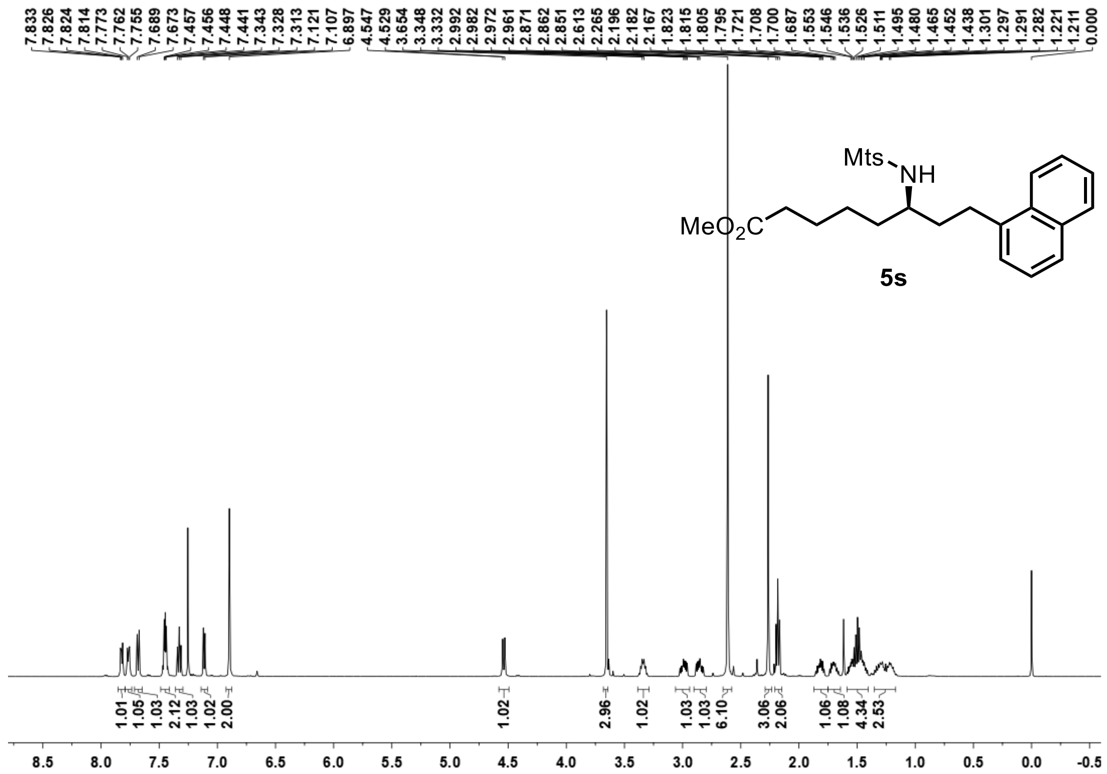

Supplementary Fig. 357 ^{1}H NMR (500 MHz, $CDCl_3$) of **5s**

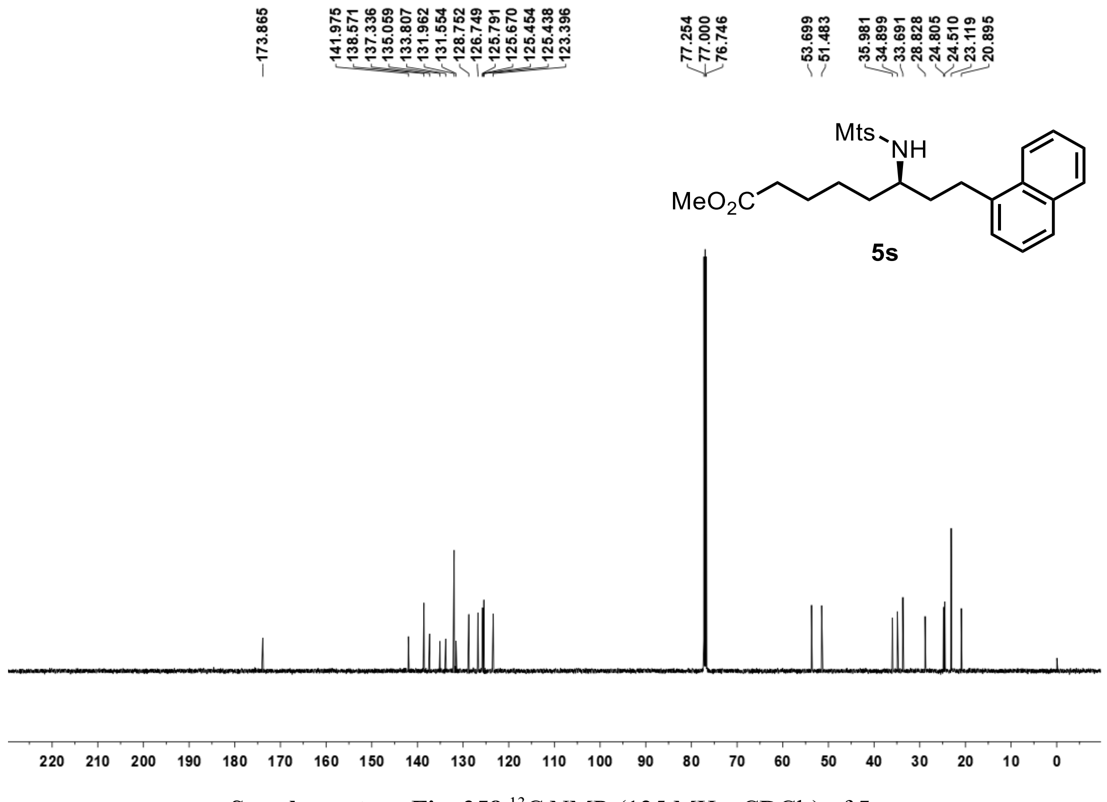

Supplementary Fig. 358 ^{13}C NMR (125 MHz, $CDCl_3$) of **5s**

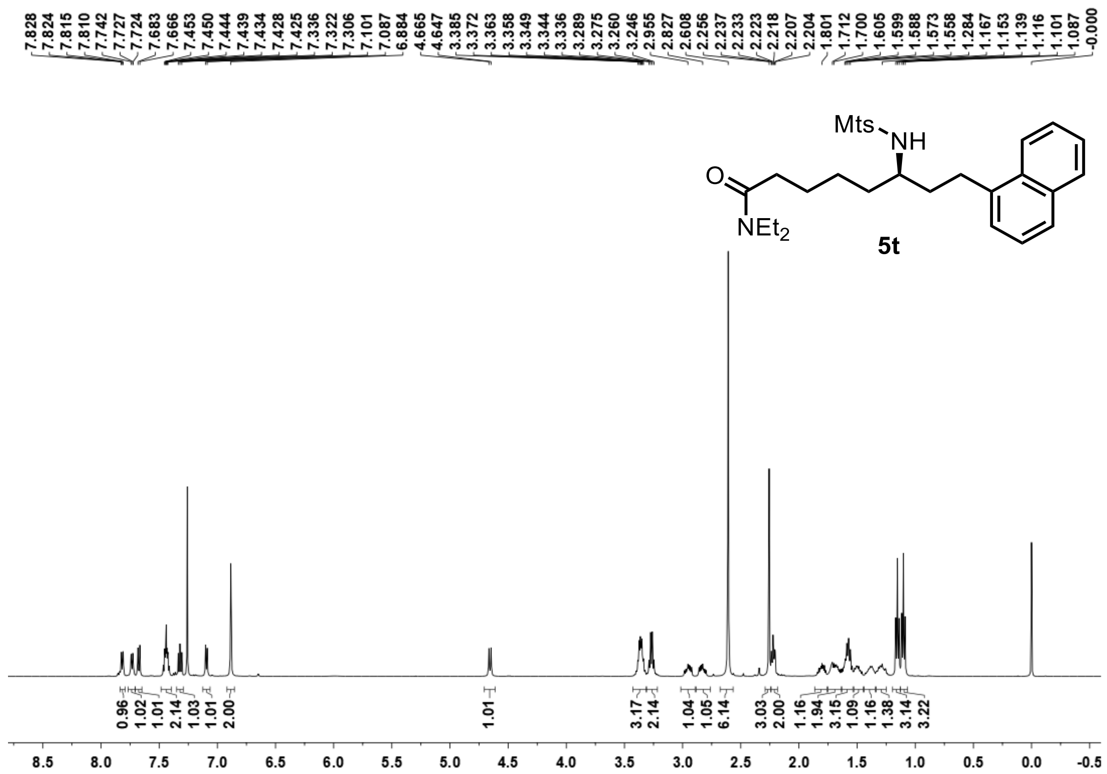

Supplementary Fig. 359 ^{1}H NMR (500 MHz, $CDCl_3$) of **5t**

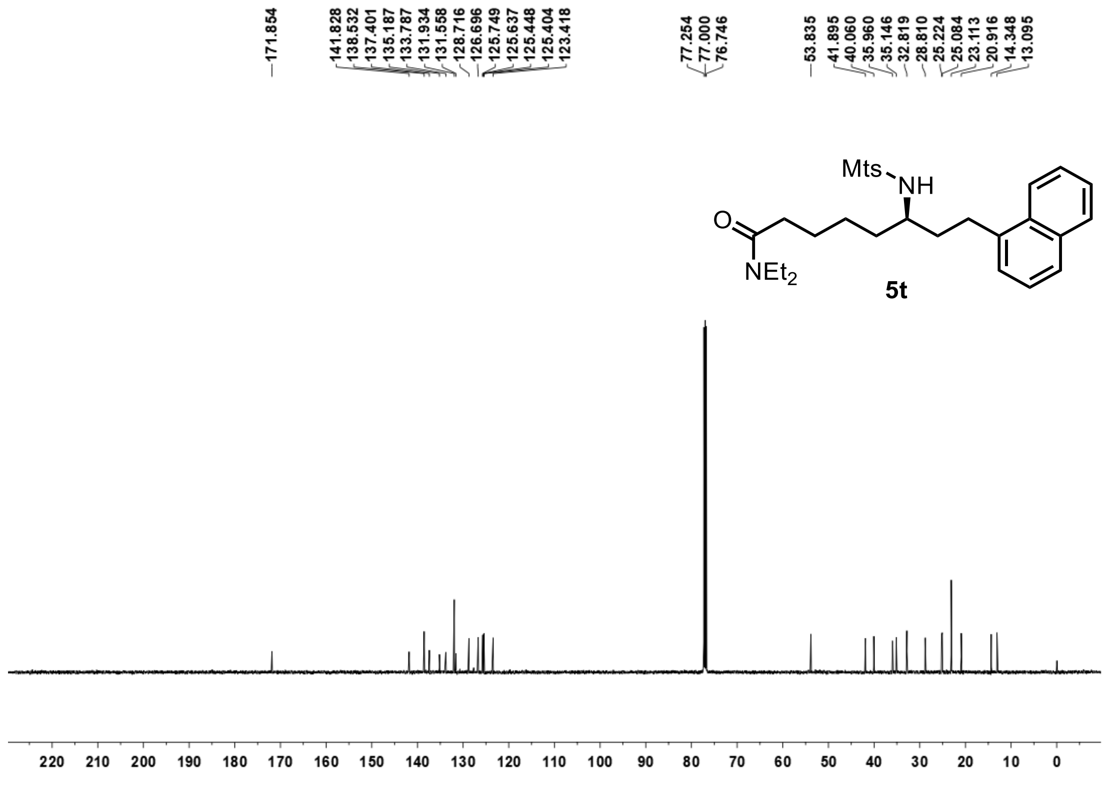

Supplementary Fig. 360 ^{13}C NMR (125 MHz, $CDCl_3$) of **5t**

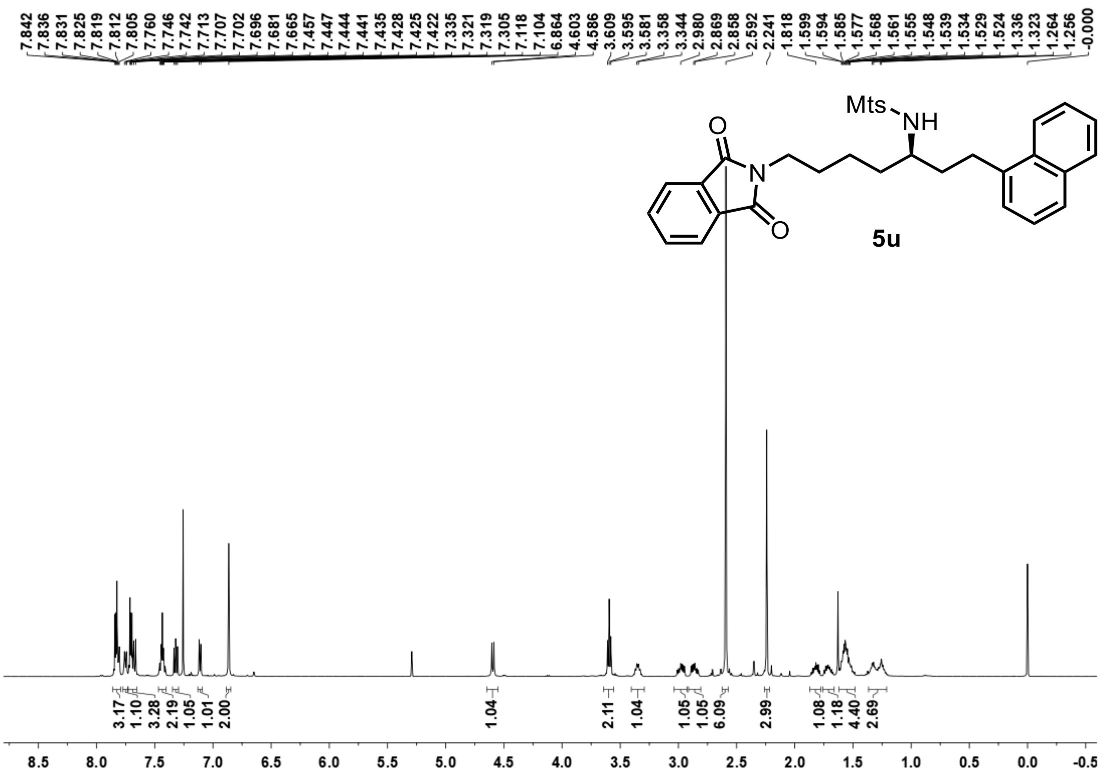

Supplementary Fig. 361 ^{1}H NMR (500 MHz, $CDCl_3$) of **5u**

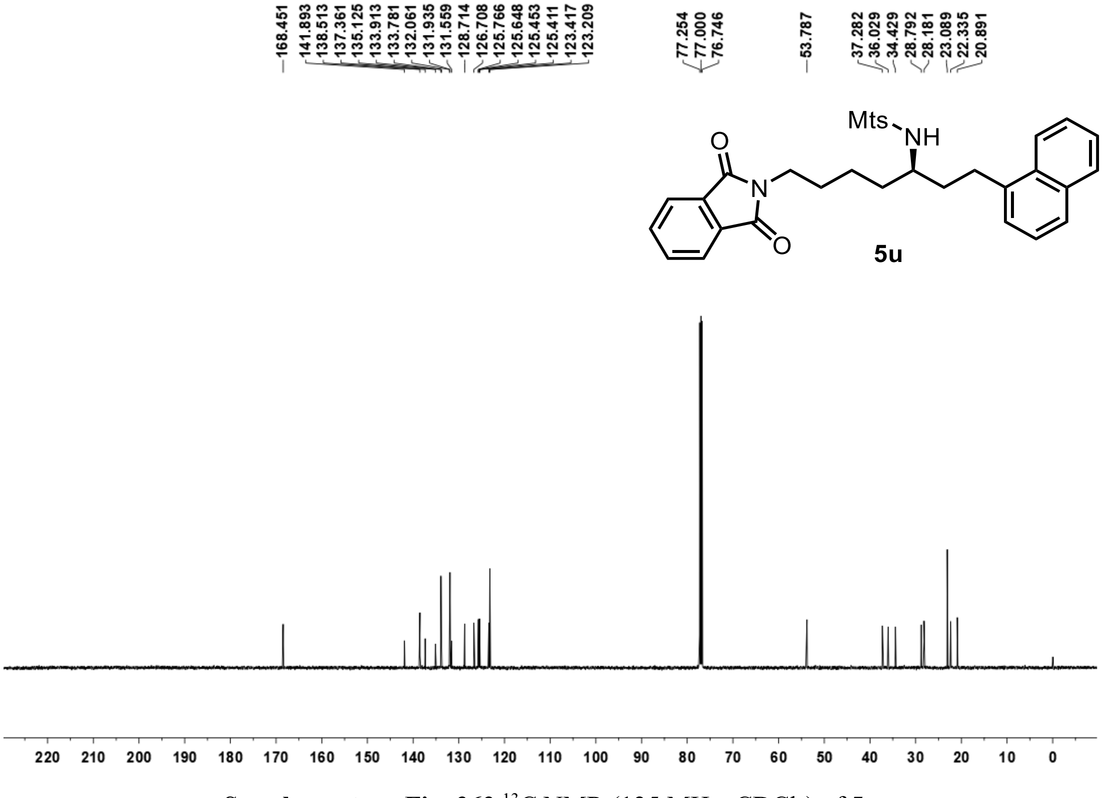

Supplementary Fig. 362 ^{13}C NMR (125 MHz, $CDCl_3$) of **5u**

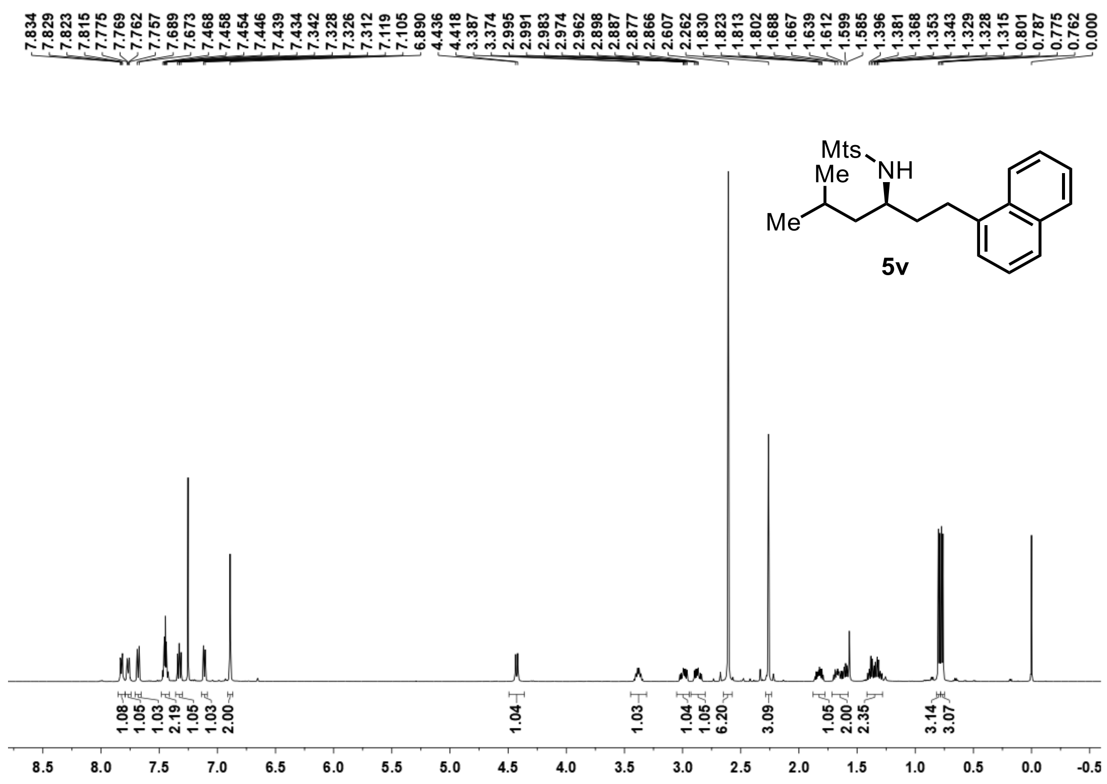

Supplementary Fig. 363 ^{1}H NMR (500 MHz, $CDCl_3$) of **5v**

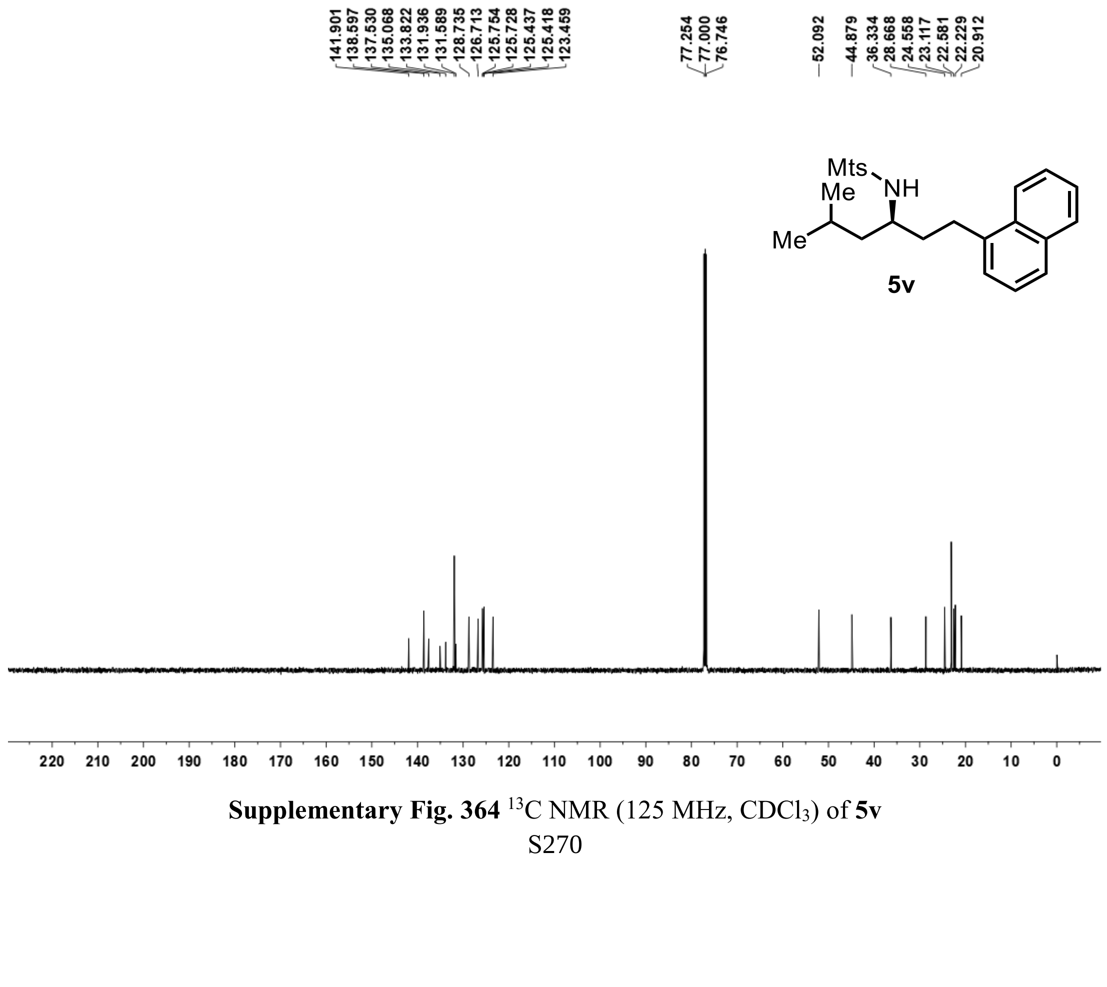

Supplementary Fig. 364 ^{13}C NMR (125 MHz, $CDCl_3$) of **5v**

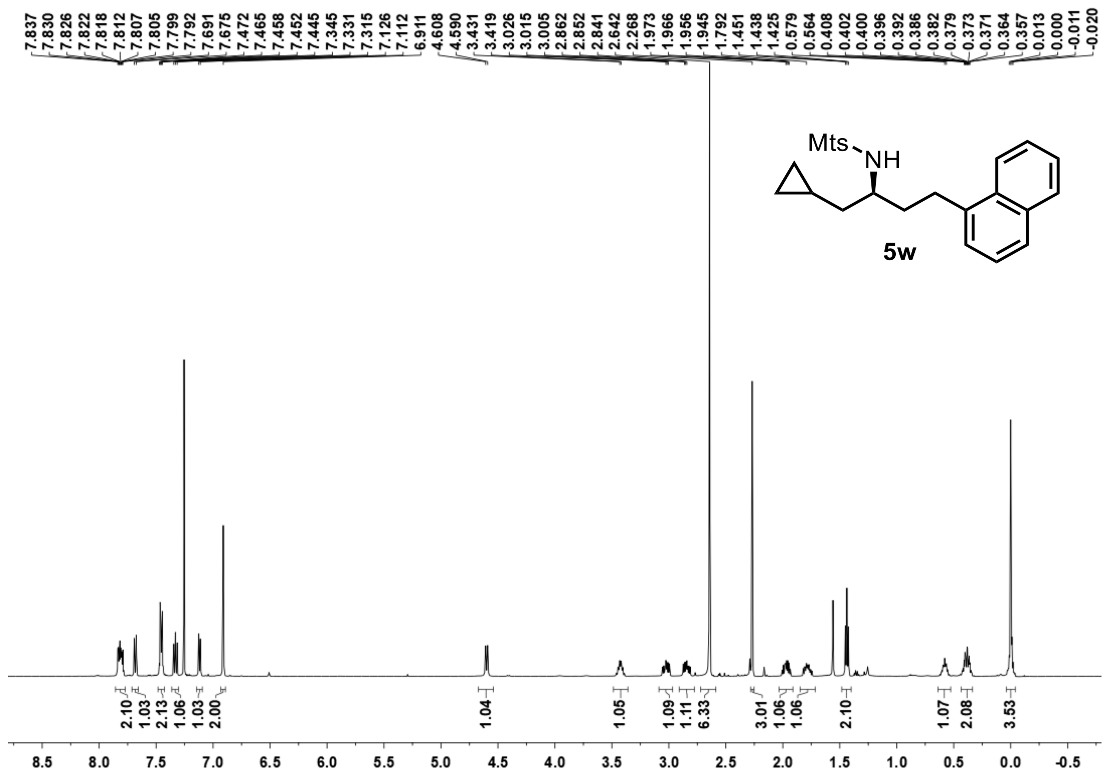

Supplementary Fig. 365 ^{1}H NMR (500 MHz, $CDCl_3$) of **5w**

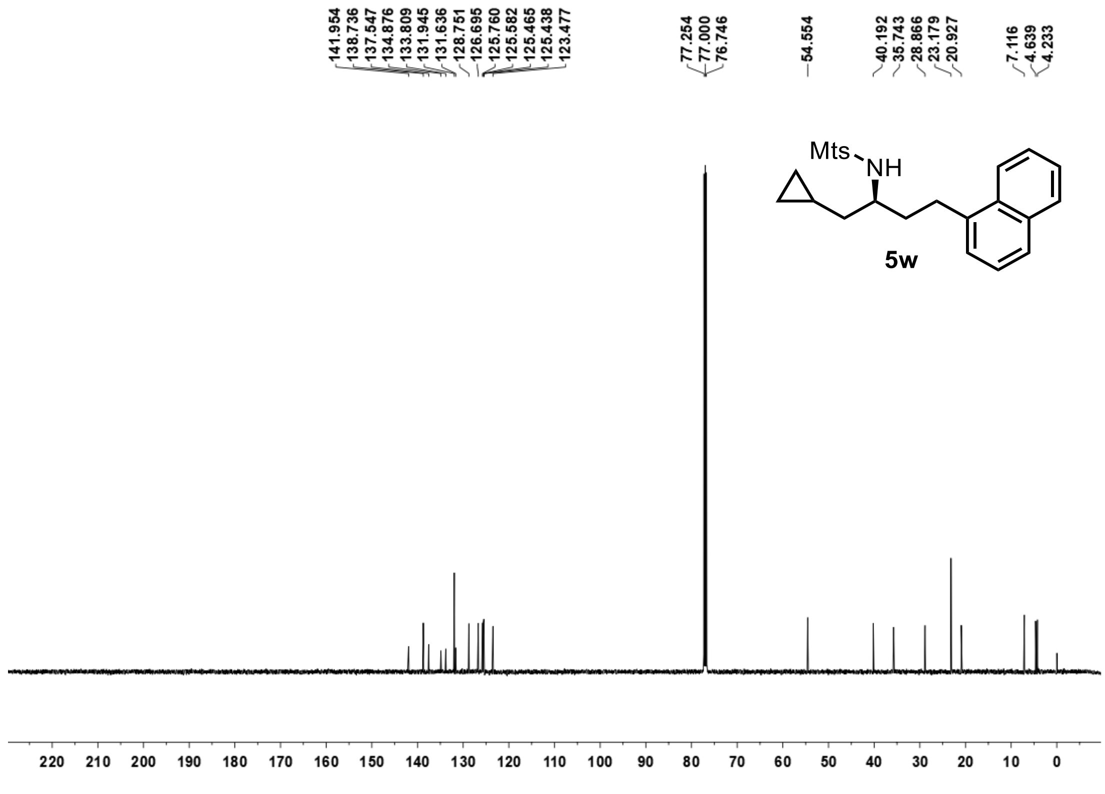

Supplementary Fig. 366 ^{13}C NMR (125 MHz, $CDCl_3$) of **5w**

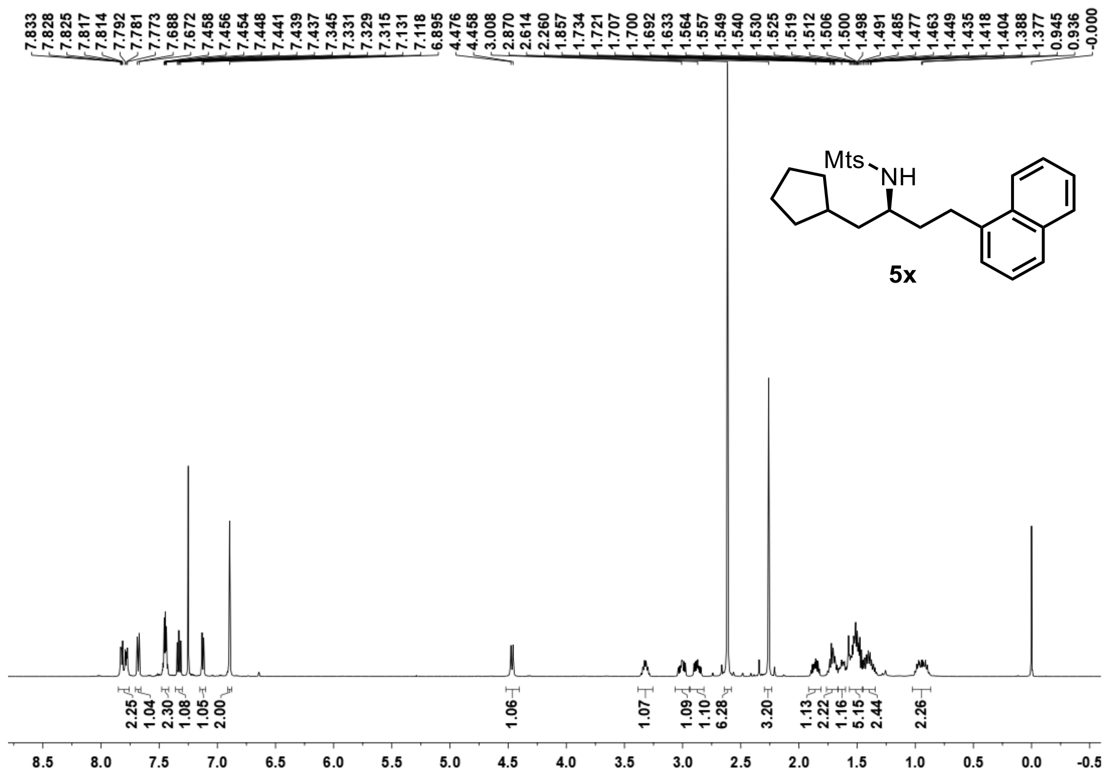

Supplementary Fig. 367 ^{1}H NMR (500 MHz, $CDCl_3$) of **5x**

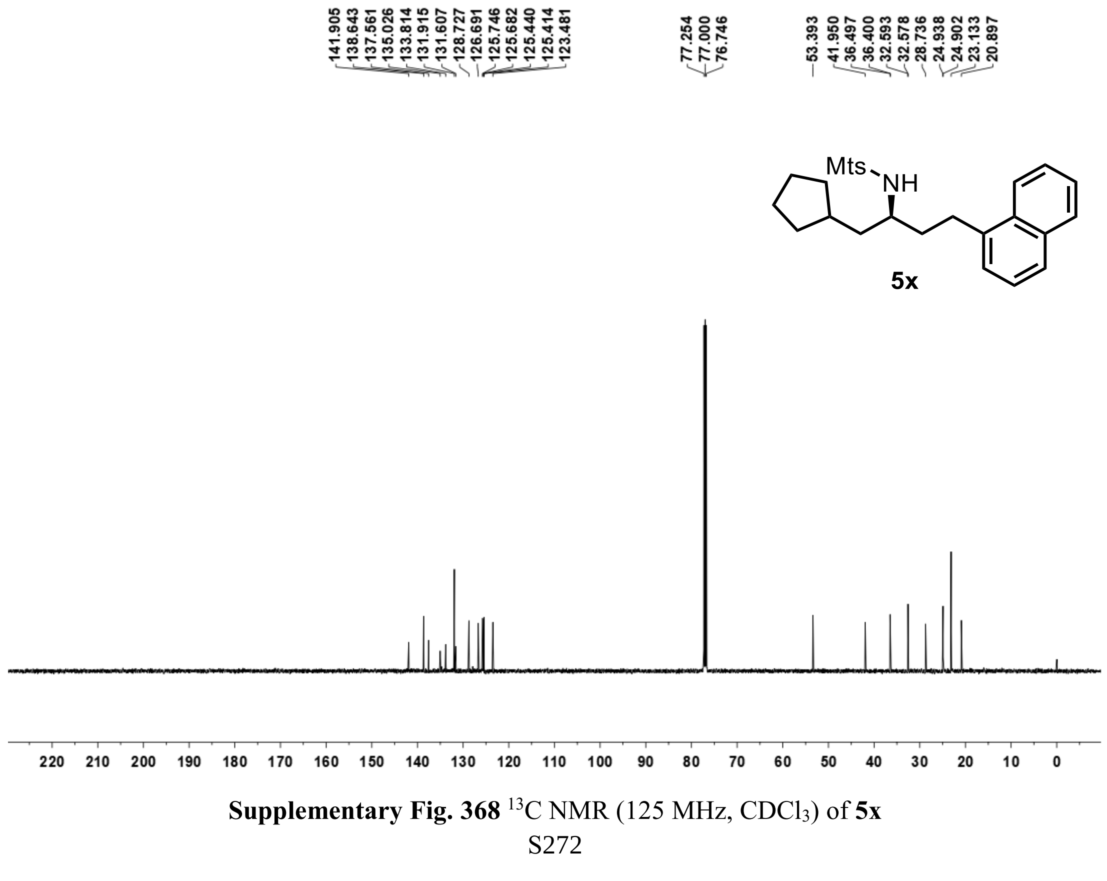

Supplementary Fig. 368 ^{13}C NMR (125 MHz, $CDCl_3$) of **5x**

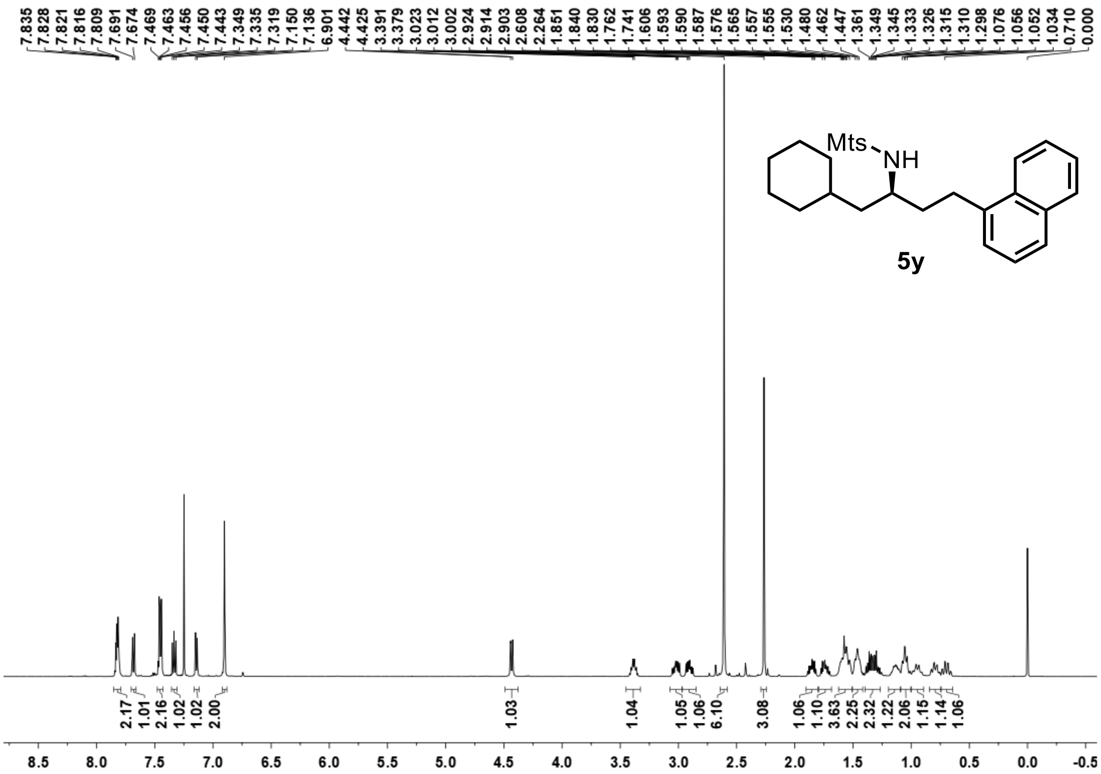

Supplementary Fig. 369 ^{1}H NMR (500 MHz, $CDCl_3$) of **5y**

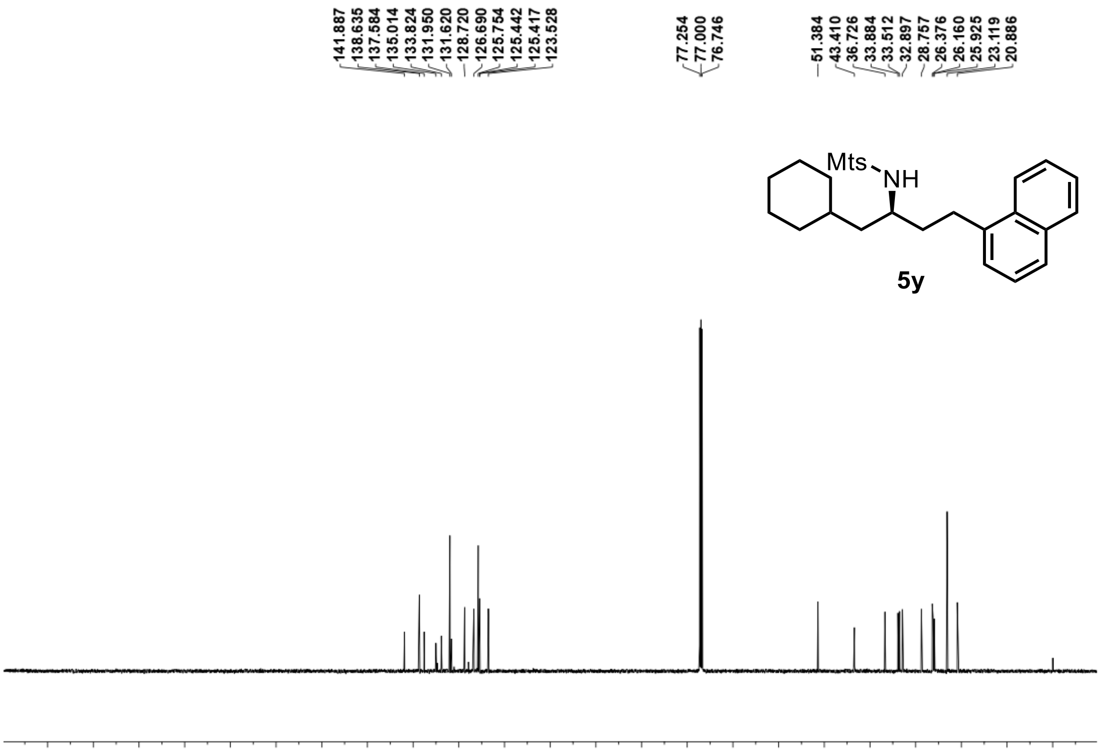

Supplementary Fig. 370 ^{13}C NMR (125 MHz, $CDCl_3$) of **5y**

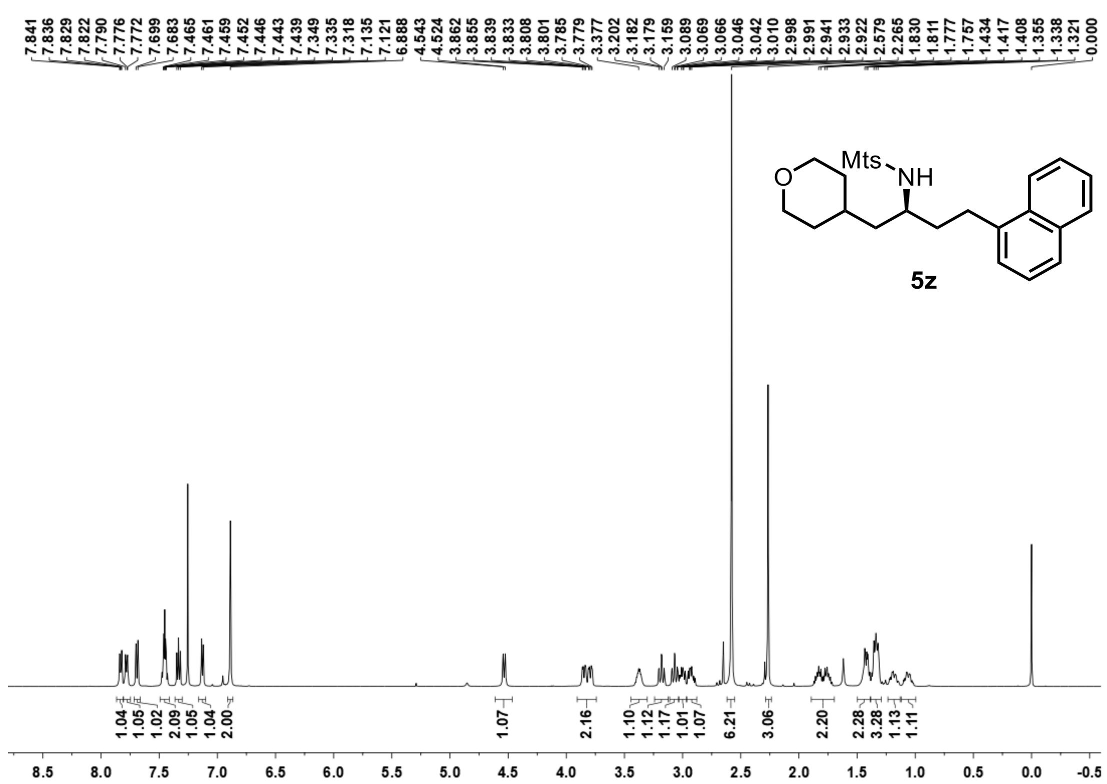

Supplementary Fig. 371 ^{1}H NMR (500 MHz, $CDCl_3$) of **5z**

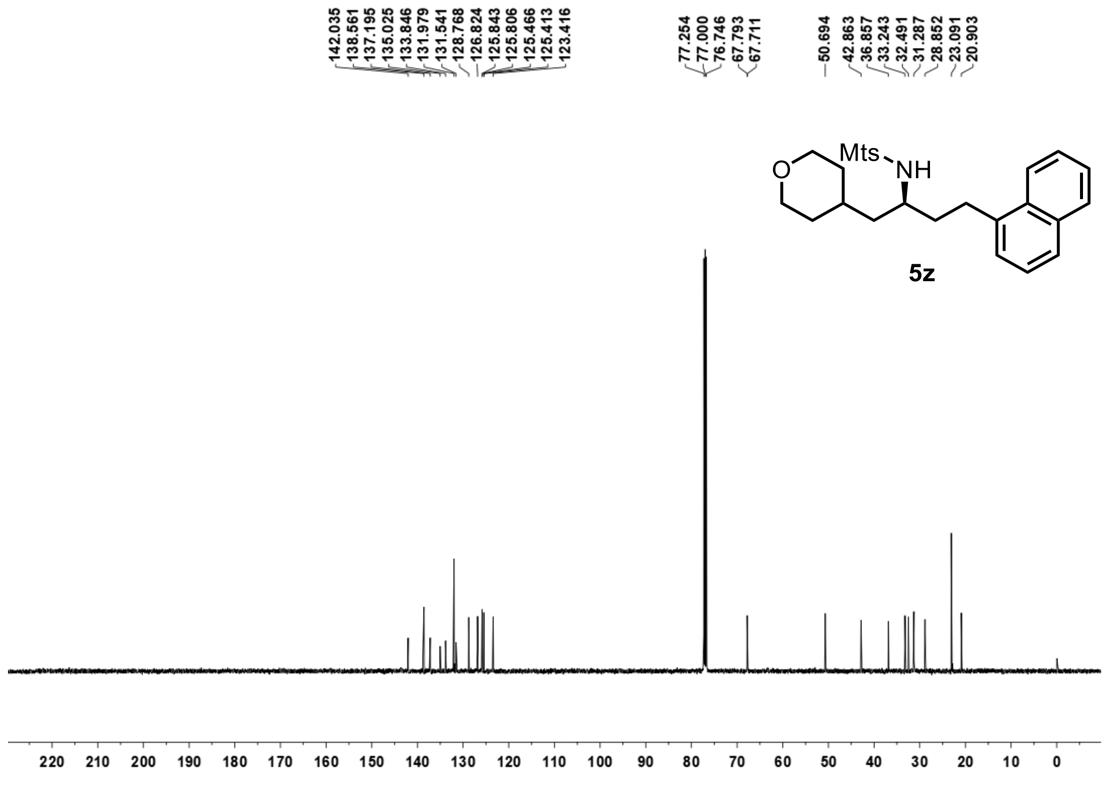

Supplementary Fig. 372 ^{13}C NMR (125 MHz, $CDCl_3$) of **5z**

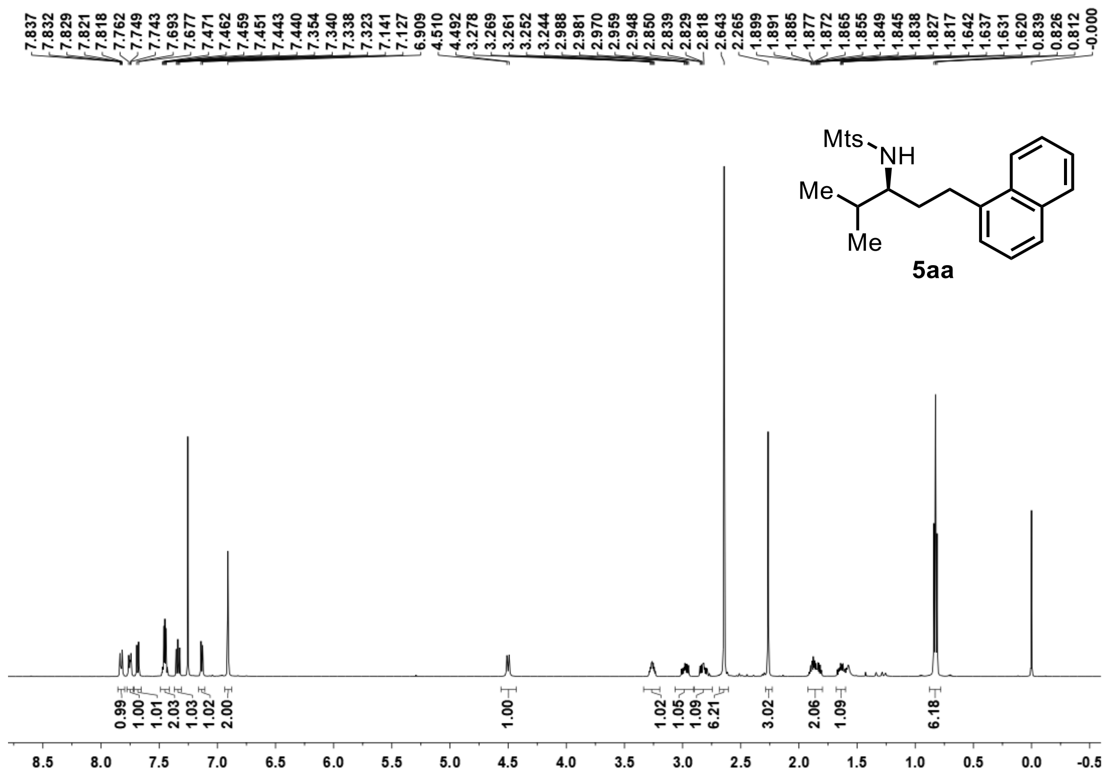

Supplementary Fig. 373 ^{1}H NMR (500 MHz, $CDCl_3$) of **5aa**

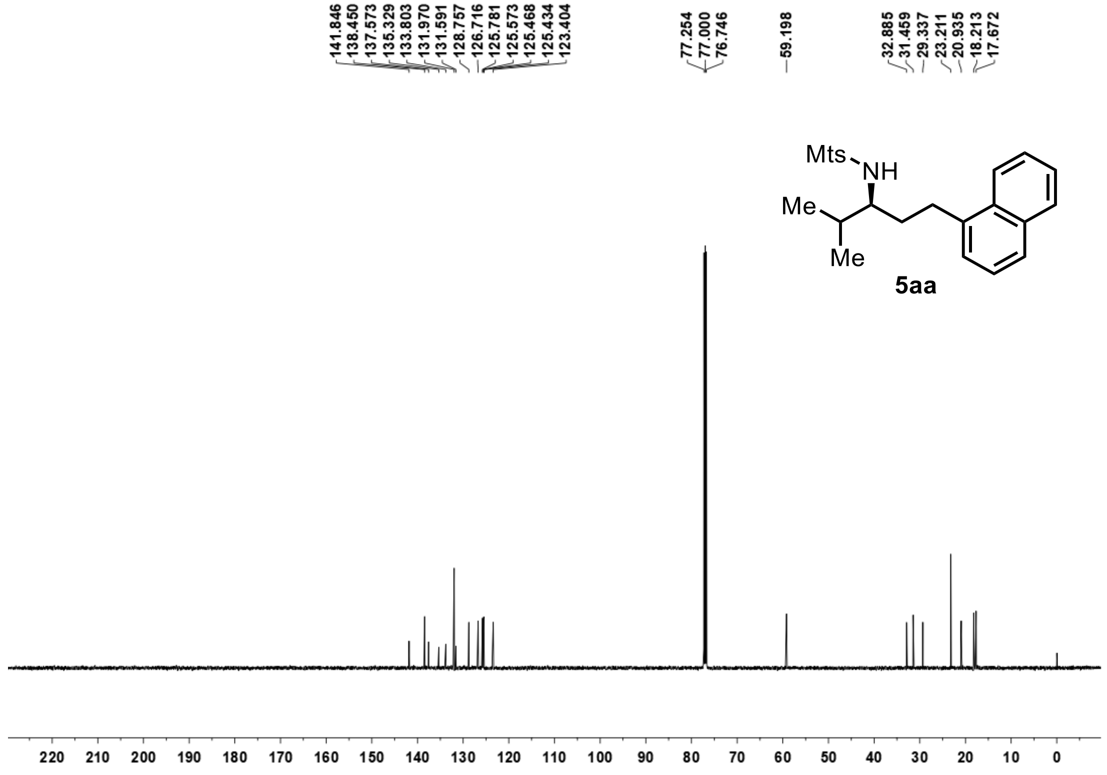

Supplementary Fig. 374 ^{13}C NMR (125 MHz, $CDCl_3$) of **5aa**

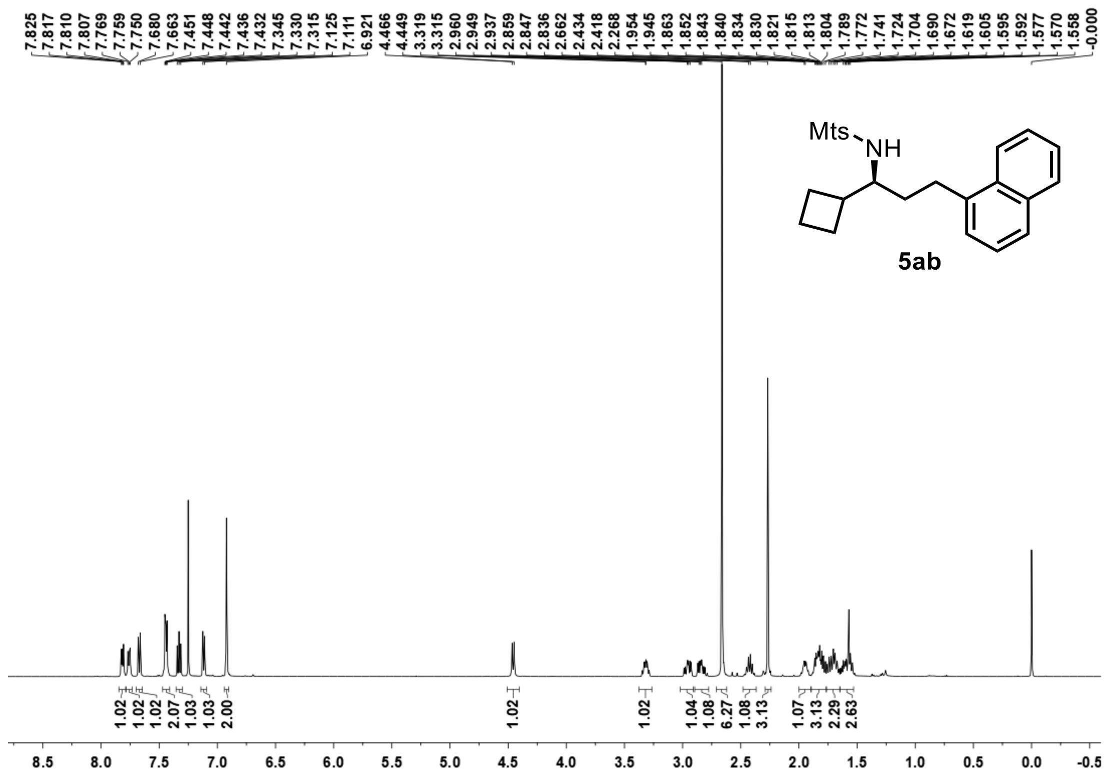

Supplementary Fig. 375 ^{1}H NMR (500 MHz, $CDCl_3$) of **5ab**

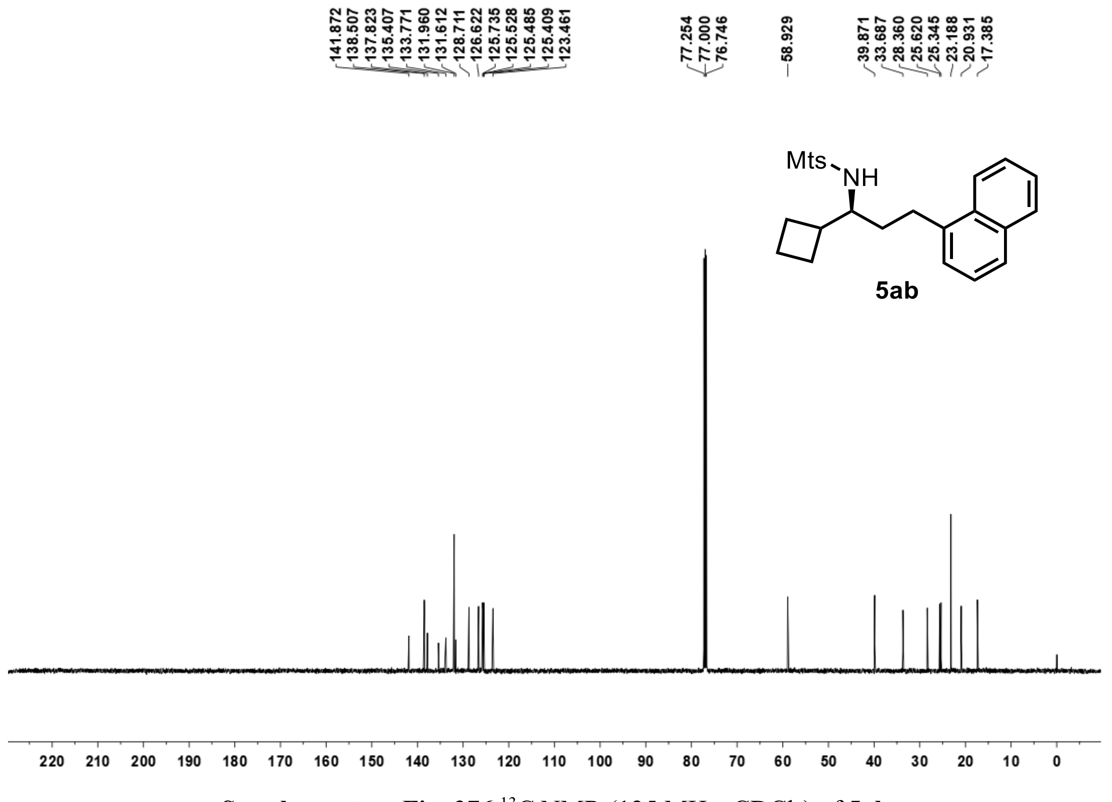

Supplementary Fig. 376 ^{13}C NMR (125 MHz, $CDCl_3$) of **5ab**

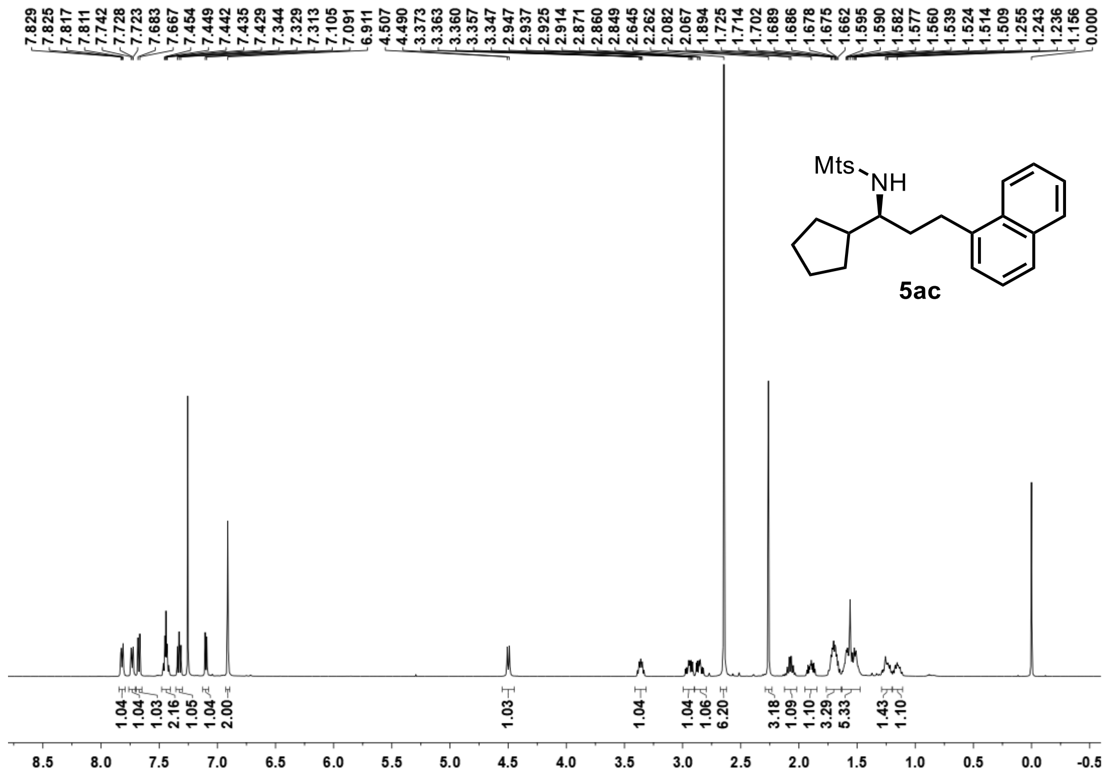

Supplementary Fig. 377 ^{1}H NMR (500 MHz, $CDCl_3$) of **5ac**

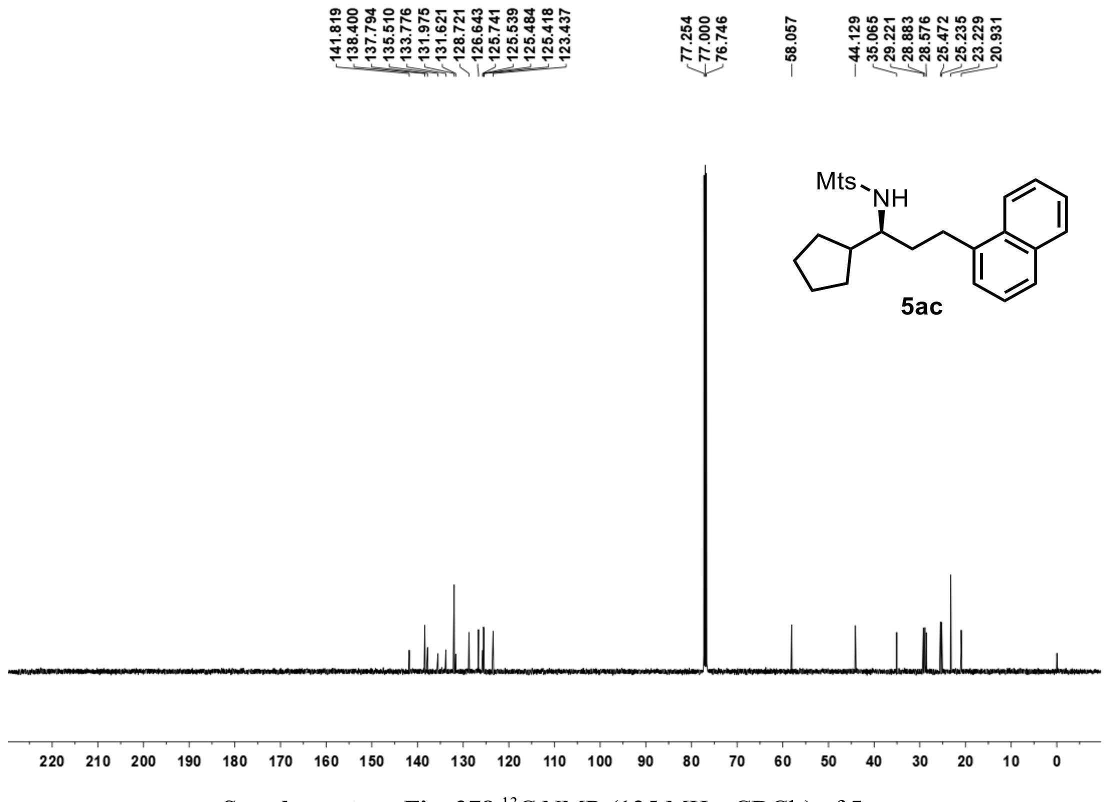

Supplementary Fig. 378 ^{13}C NMR (125 MHz, $CDCl_3$) of **5ac**

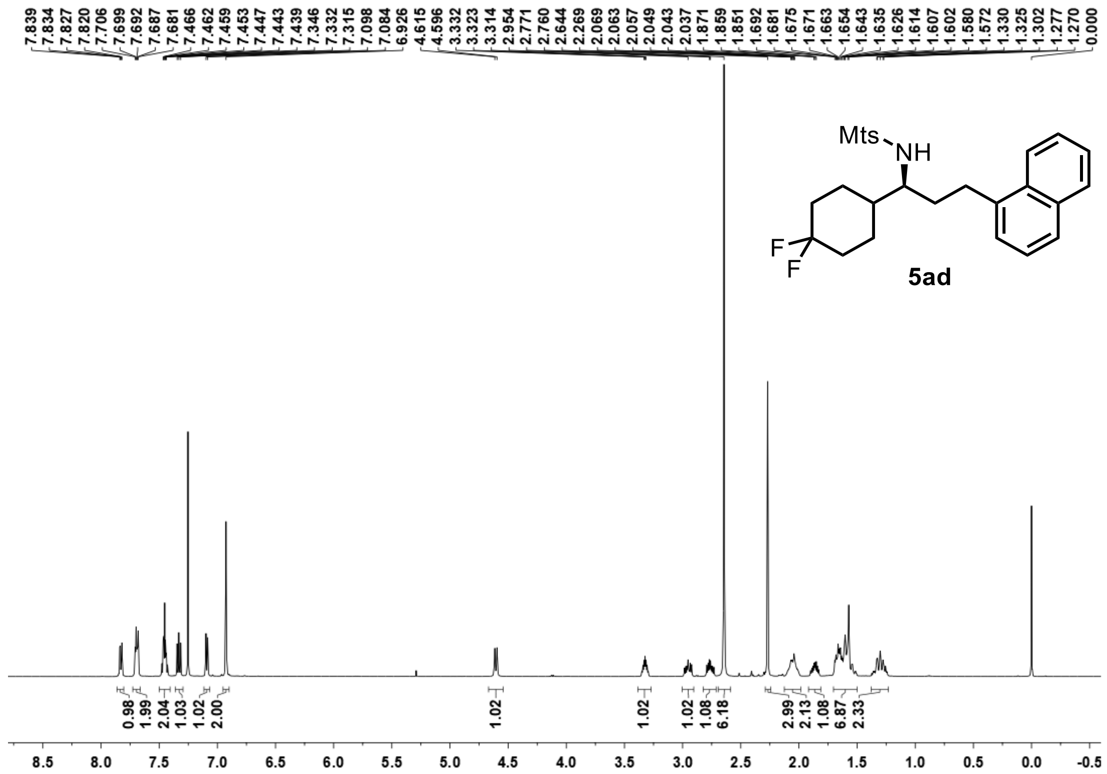

Supplementary Fig. 379 ^{1}H NMR (500 MHz, $CDCl_3$) of **5ad**

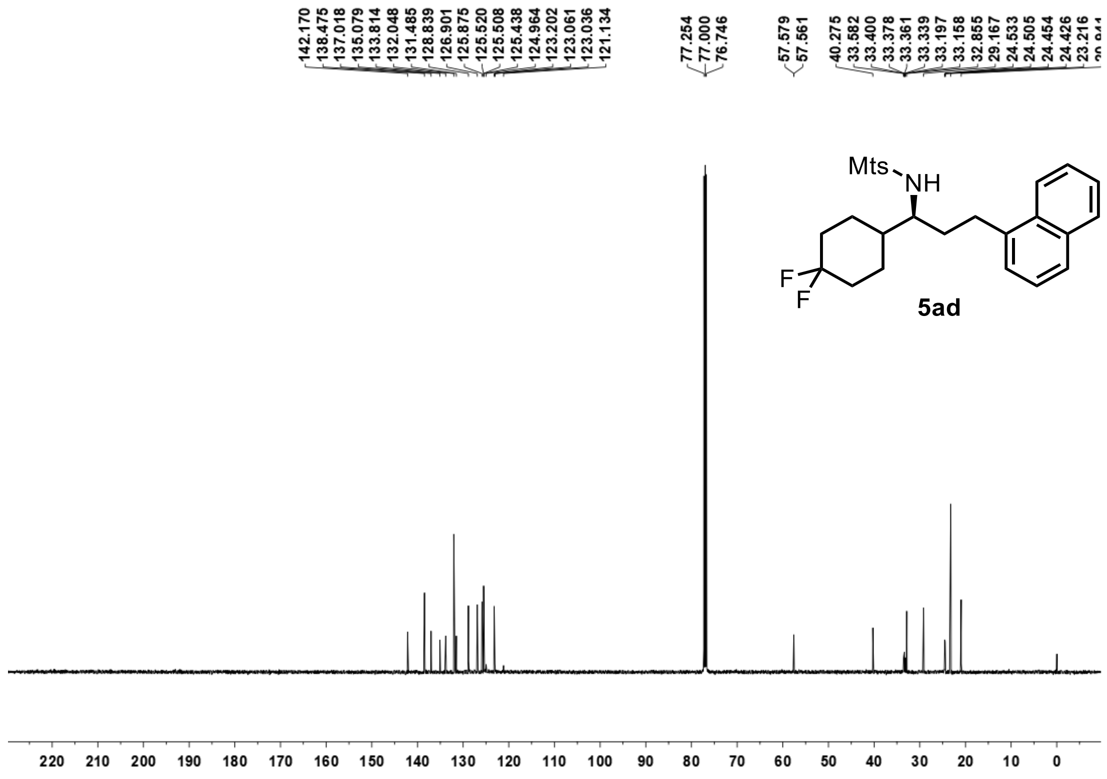

Supplementary Fig. 380 ^{13}C NMR (125 MHz, $CDCl_3$) of **5ad**

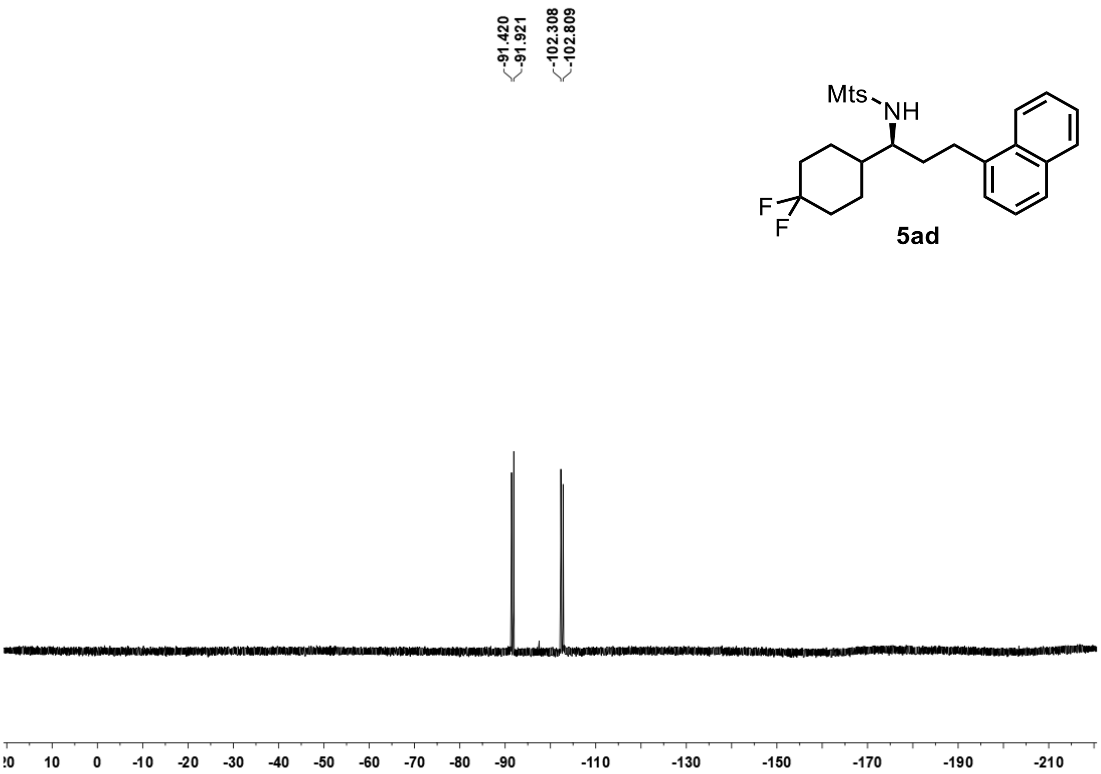

Supplementary Fig. 381 ^{19}F NMR (470 MHz, $CDCl_3$) of **5ad**

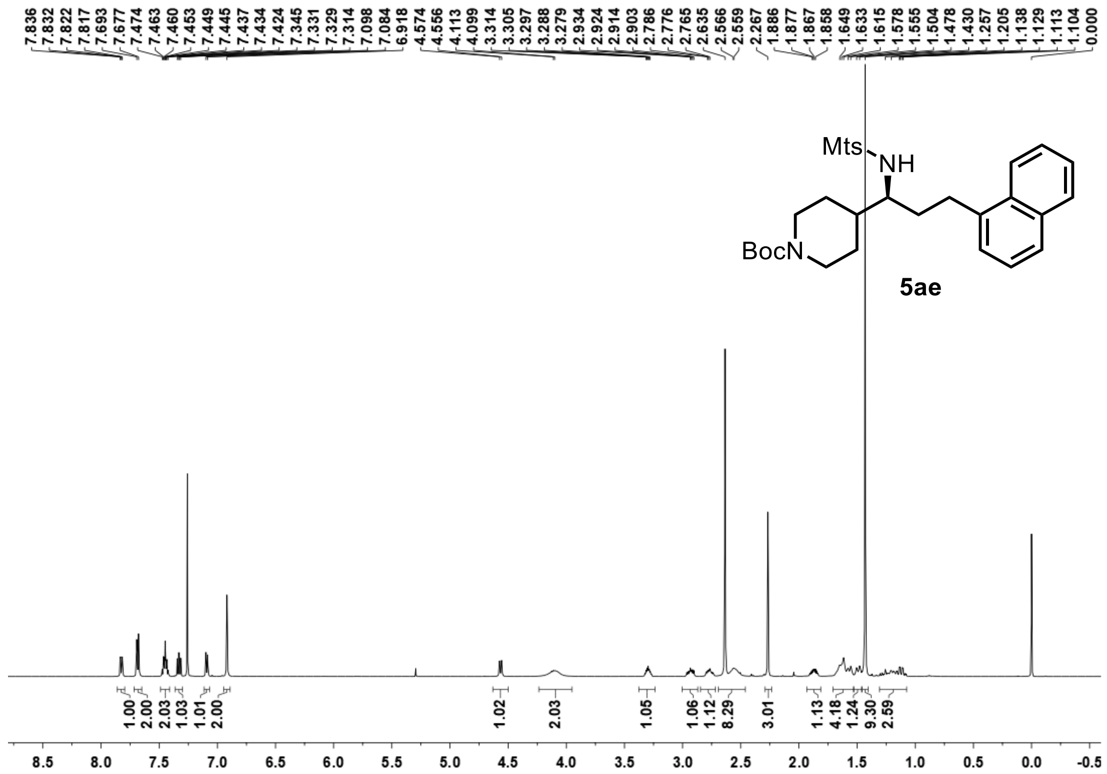

Supplementary Fig. 382 ^{1}H NMR (500 MHz, $CDCl_3$) of **5ae**

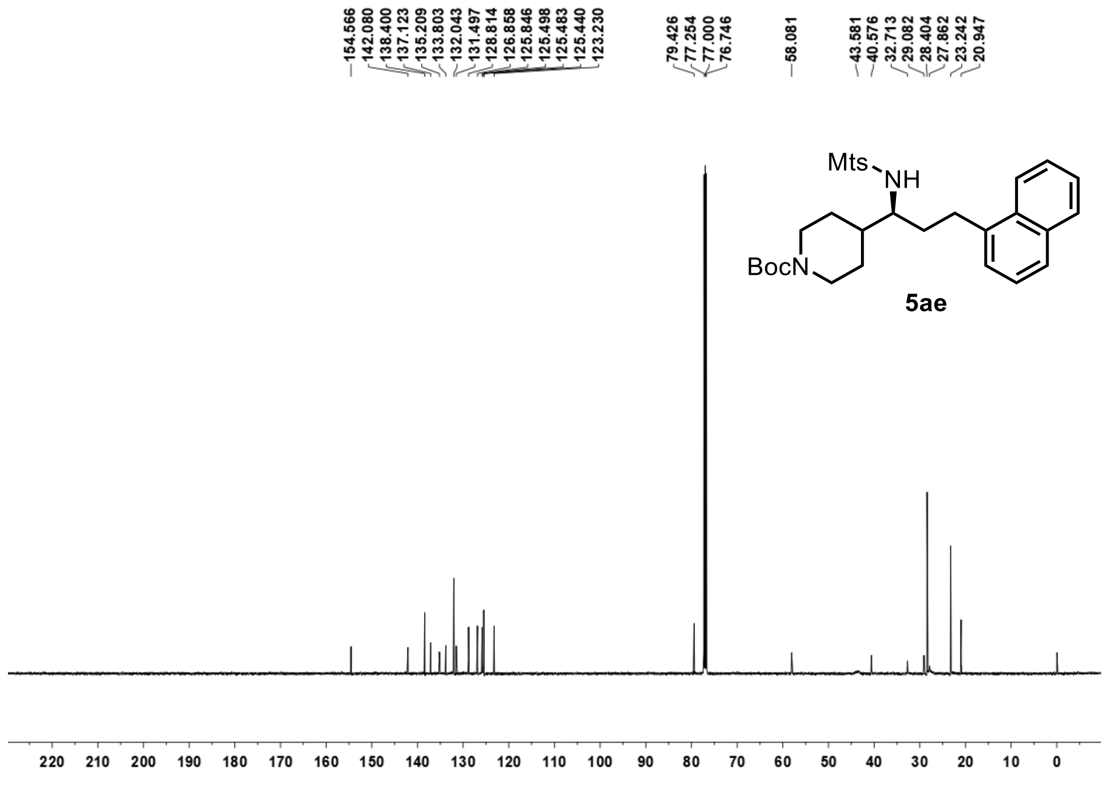

Supplementary Fig. 383 ^{13}C NMR (125 MHz, $CDCl_3$) of **5ae**

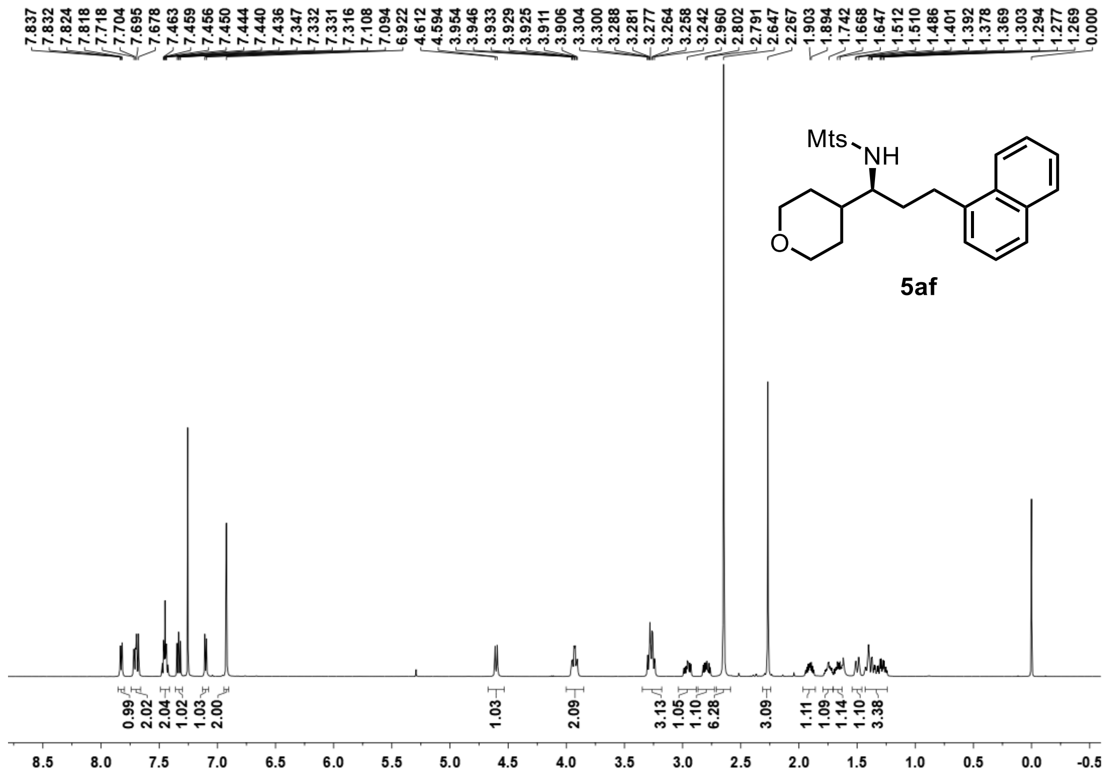

Supplementary Fig. 384 ^{1}H NMR (500 MHz, $CDCl_3$) of **5af**

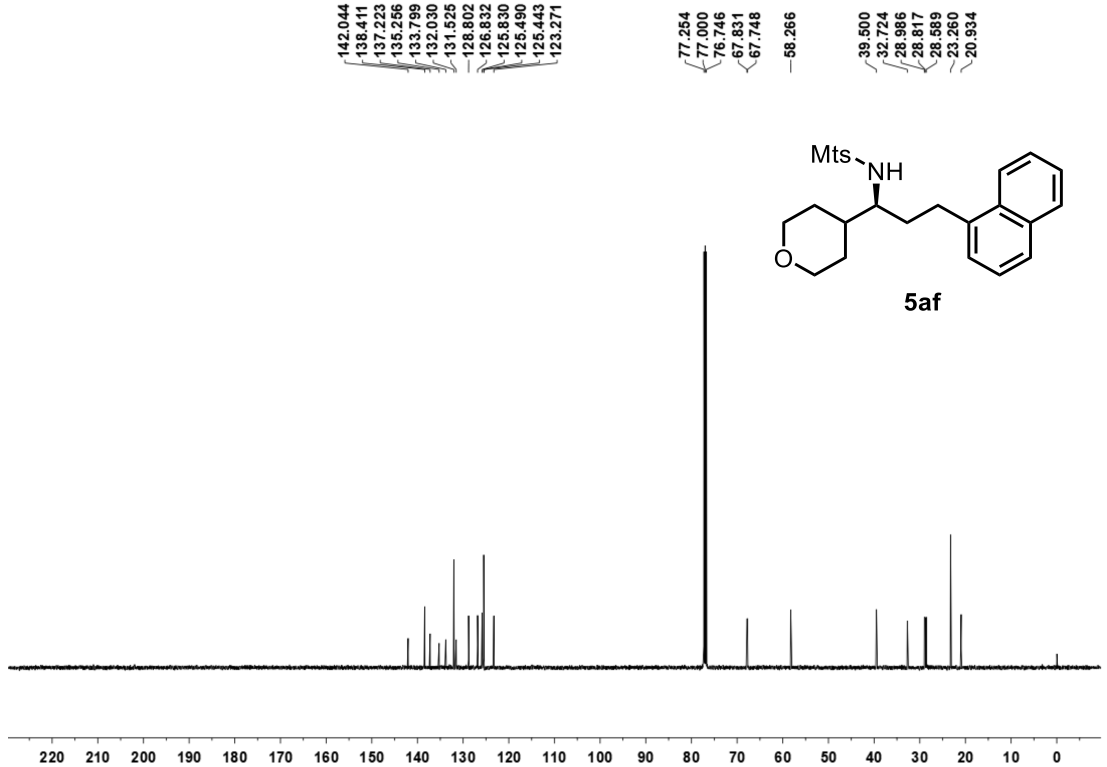

Supplementary Fig. 385 ^{13}C NMR (125 MHz, $CDCl_3$) of **5af**

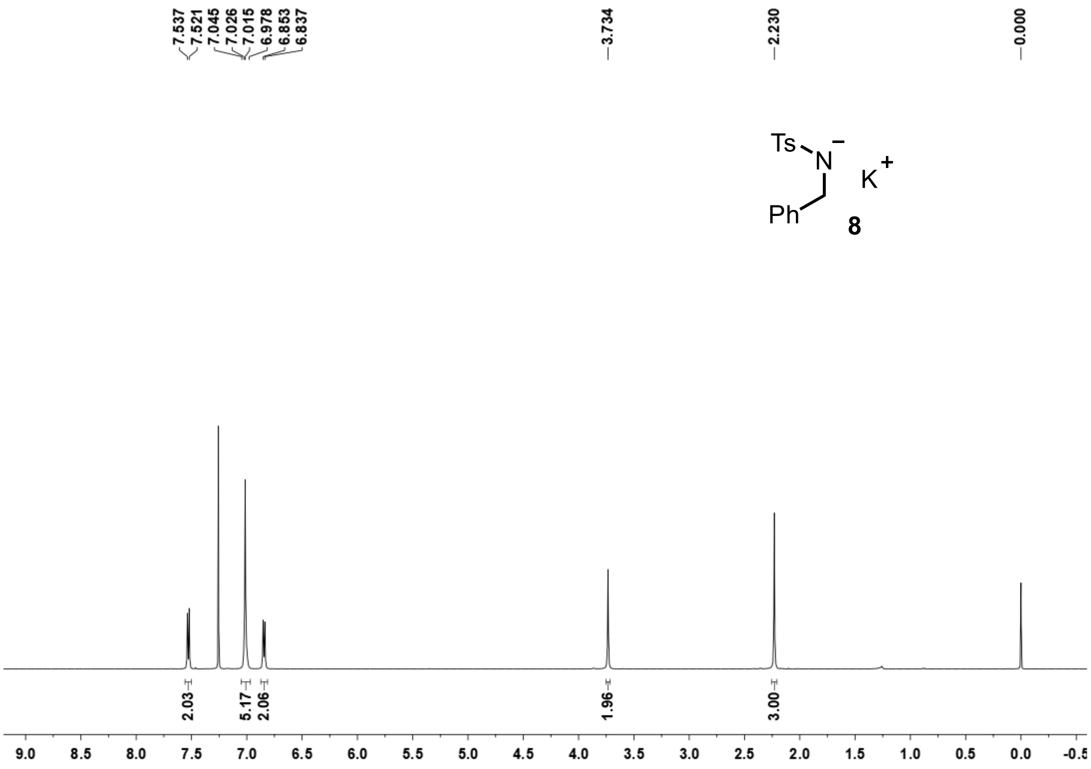

Supplementary Fig. 386 ^{1}H NMR (500 MHz, $CDCl_3$) of **8**

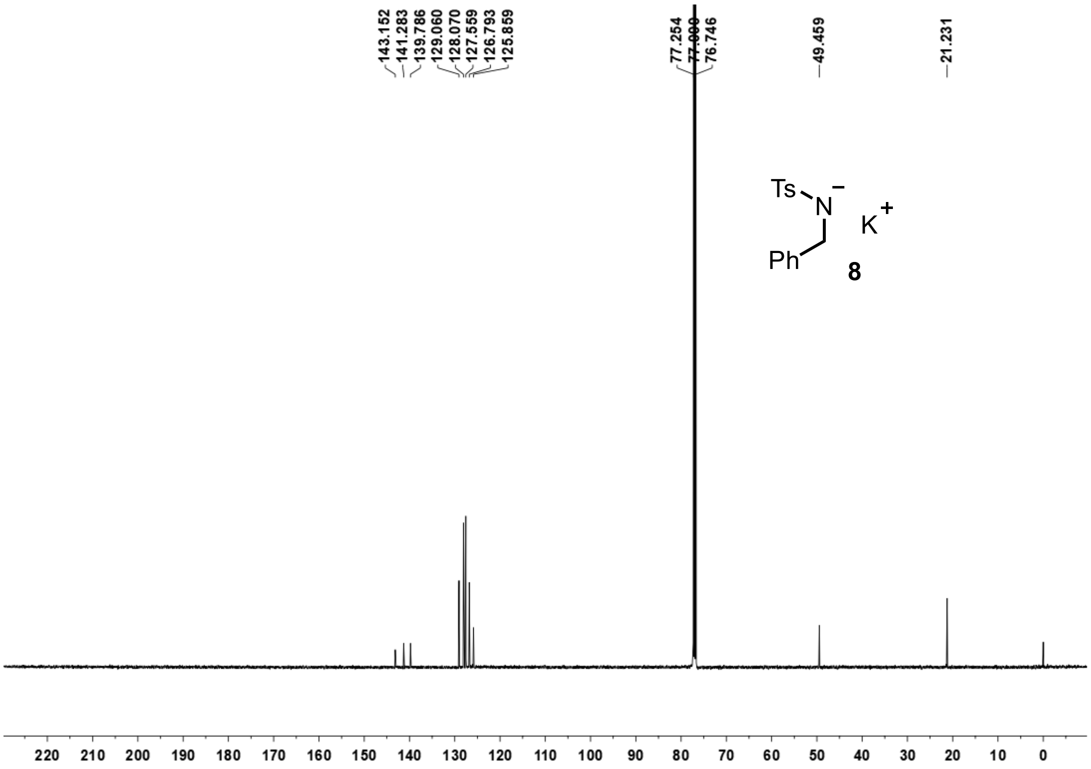

Supplementary Fig. 387 ^{13}C NMR (125 MHz, $CDCl_3$) of **8**

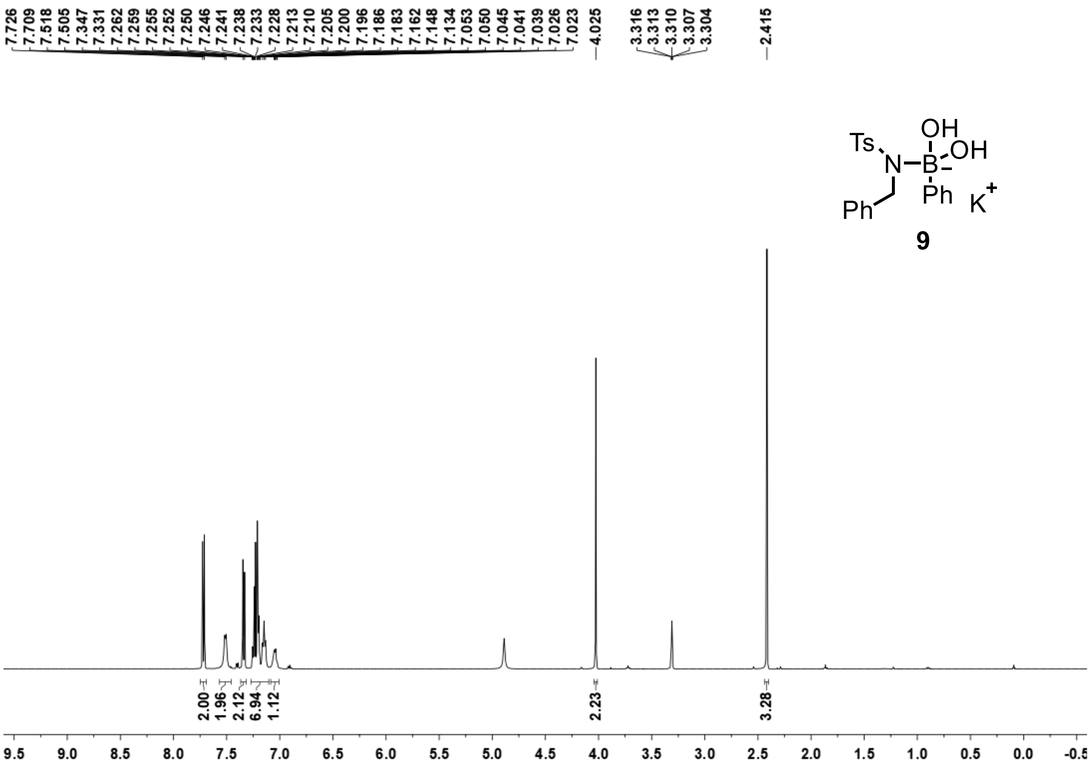

Supplementary Fig. 388 ^{1}H NMR (500 MHz, CD_3OD) of **9**

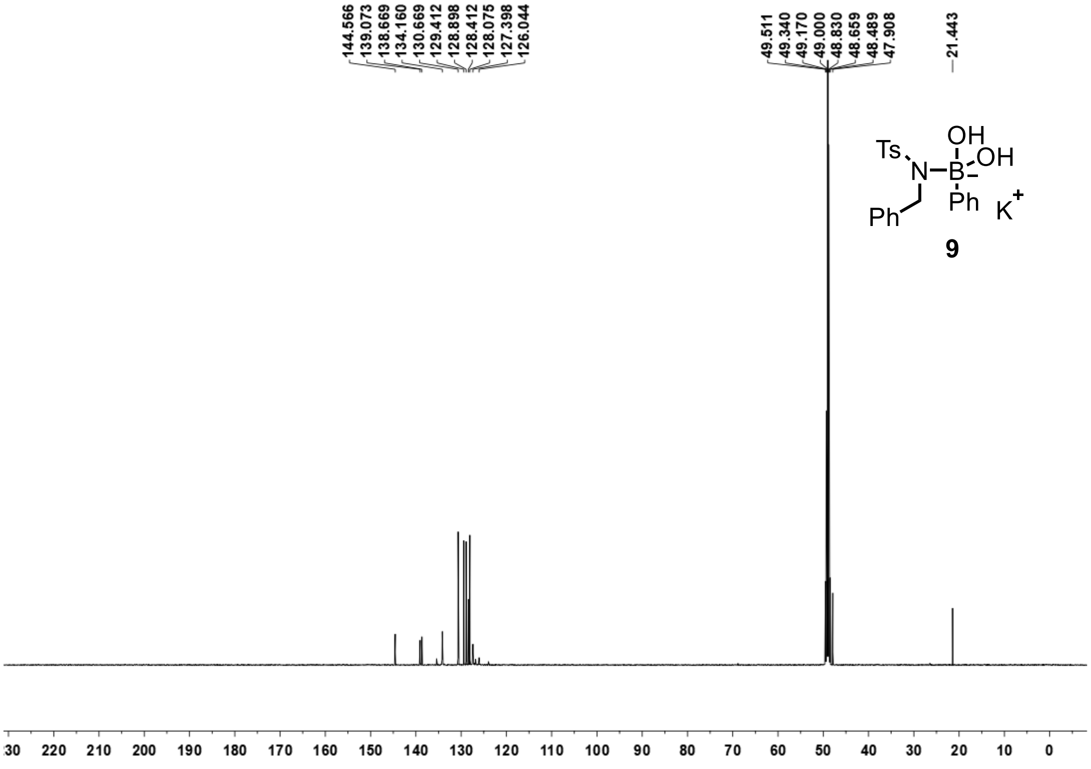

Supplementary Fig. 389 ^{13}C NMR (125 MHz, CD_3OD) of **9**

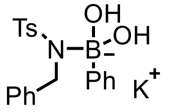

9

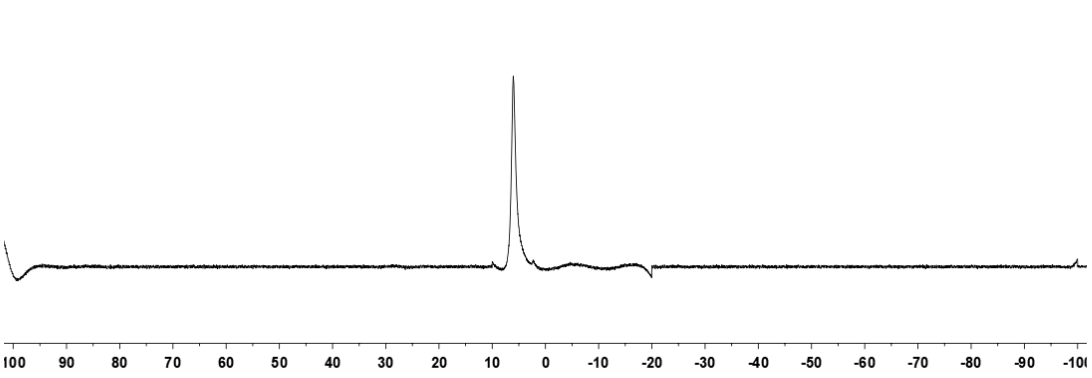

Supplementary Fig. 390 ^{11}B NMR (160 MHz, CD_3OD) of **9**

3. Supplementary Note 2

Crystal Data and Structure Refinement for 5n

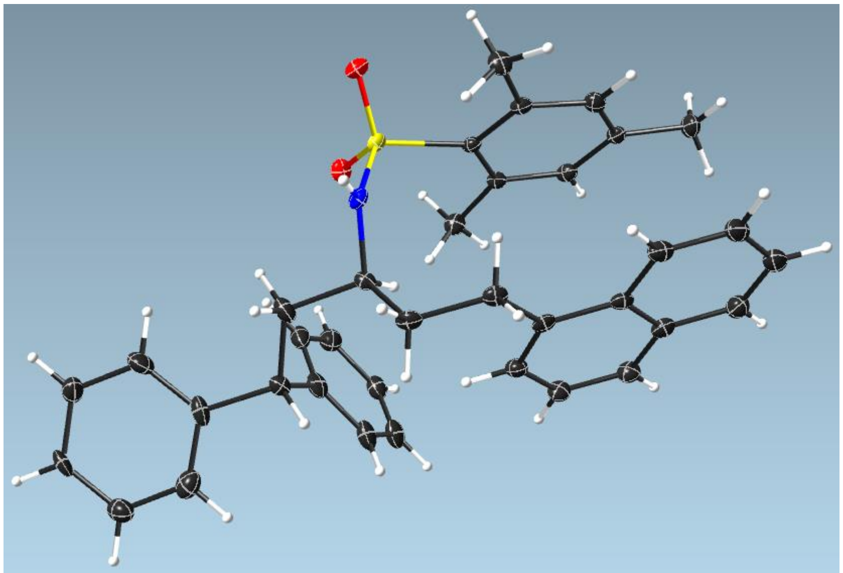

CCDC	2092983
Empirical formula	$C_{36}H_{37}NO_2S$
Formula weight	547.72
Temperature/K	100(2)
Crystal system	Orthorhombic
Space group	$P2_12_12_1$
a/Å	7.3566(6)
b/Å	12.1081(9)
c/Å	31.799(2)
α/°	90°
β/°	90°
γ/°	90°
Volume/Å3	2832.5(4)
Z	4
ρ_{calc}g/cm3	1.284
μ/mm^{-1}	0.149
F(000)	1168
Crystal size/mm^3	0.2 x 0.1 x 0.085
Radiation	MoKa (λ = 0.71073)

Theta range for data collection/°	2.114 to 28.283
Index ranges	-9<=h<=9, -16<=k<=16, -42<=l<=38
Reflections collected	50554
Refinement method	Full-matrix least-squares on F^2
Independent reflections	7014 [R(int) = 0.0705]
Data/restraints/parameters	7014 / 0 / 364
Goodness-of-fit on F^2	1.137
Final R indices [I>2sigma(I)]	R1 = 0.0550, wR2 = 0.1163
R indices (all data)	R1 = 0.0599, wR2 = 0.1189
Largest diff. peak and hole/ e Å^{-3}	0.760 and -0.392
Absolute structure parameter	0.01(3)

4. Supplementary References

[1] Chatterjee, I. & Oestreich, M. $B(C_6F_5)_3$-catalyzed transfer hydrogenation of imines and related heteroarenes using cyclohexa-1,4-dienes as a dihydrogen source. *Angew. Chem. Int. Ed.* **54**, 1965–1968 (2015).

[2] Huang, M., Li, Y., Liu, J., Lan, X.-B., Liu, Y., Zhao, C. & Ke, Z. A bifunctional strategy for *N*-heterocyclic carbene-stabilized iridium complex-catalyzed *N*-alkylation of amines with alcohols in aqueous media. *Green Chem.* **21**, 219–224 (2019).

[3] Hamid, M. H. S. A., Allen, C. L., Lamb, G. W., Maxwell, A. C., Maytum, H. C., Watson, A. J. A. & Williams, J. M. J. Ruthenium-catalyzed *N*-alkylation of amines and sulfonamides using borrowing hydrogen methodology. *J. Am. Chem. Soc*. **131**, 1766–1774 (2019).

[4] Verdelet, T., Ward, R. M. & Hall, D. G. Direct sulfonamidation of primary and secondary benzylic alcohols catalyzed by a boronic acid/oxalic acid system. *Eur. J. Org. Chem.* 5729–5738 (2017).

[5] Qu, P., Sun, C., Ma, J. & Li, F. The *N*-alkylation of sulfonamides with alcohols in water catalyzed by the water-soluble iridium complex {Cp*Ir[6,6′-$(OH)_2$bpy](H_2O)}$[OTf]_2$. *Adv. Synth. Catal.* **356**, 447–459 (2014).

[6] Cui, X., Shi, F., Tse, M. K., Gördes, D., Thurow, K., Beller, M. & Deng, Y. Copper-catalyzed *N*-alkylation of sulfonamides with benzylic alcohols: catalysis and mechanistic studies. *Adv. Synth. Catal.* **351**, 2949–2958 (2009).

[7] Laha, J. K., Dayal, N., Jain, R. & Patel, K. Palladium-catalyzed regiocontrolled domino synthesis of *N*-sulfonyl dihydrophenanthridines and dihydrodibenzo[*c*,*e*]azepines: control over the formation of biaryl aultams in the intramolecular direct arylation. *J. Org. Chem.* **79**, 10899−10907 (2014).

[8] Zhu, M., Fujita, K.-i. & Yamaguchi, R. Simple and versatile catalytic system for *N*-alkylation of sulfonamides with various alcohols. *Org. Lett.* **12**, 1336–1339 (2010).

[9] Hayashi, R., Shimizu, A., Song, Y., Ashikari, Y., Nokami, T. & Yoshida, J.-i. Metal-free benzylic C−H amination via electrochemically generated benzylaminosulfonium ions. *Chem. Eur. J.* **23**, 61–64 (2017).

[10] Kang, Q.-K., Lin, Y., Li, Y. & Shi, H. Ru(II)-catalyzed amination of aryl fluorides via η^6-coordination. *J. Am. Chem. Soc.* **142**, 3706−3711 (2020).

[11] Kloss, F., Neuwirth, T., Haensch, V. G. & Hertweck, C. Metal-free synthesis of pharmaceutically important biaryls by photosplicing. *Angew. Chem. Int. Ed.* **57**, 14476–14481 (2018).

[12] Bai, R., Liang, Z., Yoon, Y., Salgado, E., Feng, A., Gurbani, S. & Shim, H. Novel anti-inflammatory agents targeting CXCR4: design, synthesis, biological evaluation and preliminary pharmacokinetic study. *Eur. J. Med. Chem.* **136**, 360–371 (2017).

[13] Dai, Y., Zheng, J. & Zhang, Q. General strategy for stereoselective synthesis of β-*N*-glycosyl sulfonamides via palladium-catalyzed glycosylation. *Org. Lett.* **20**, 3923−3927 (2018).

[14] Drew, M. A., Arndt, S., Richardson, C., Rudolph, M., Hashmi, A. S. K. & Hyland, C. J. T. Divergent gold-catalysed reactions of cyclopropenylmethyl sulfonamides with tethered heteroaromatics. *Chem. Commun.* **55**, 13971−13974 (2019).

[15] Henry, M. C., Senn, H. M. & Sutherland, A. Synthesis of functionalized indolines and dihydrobenzofurans by iron and copper catalyzed aryl C–N and C–O bond formation. *J. Org. Chem.* **84**, 346−364 (2019).

[16] Zhou, T., Luo, F.-X., Yang, M.-Y. & Shi, Z.-J. Silver-catalyzed long-distance aryl migration from carbon center to nitrogen center. *J. Am. Chem. Soc.* **137**, 14586−14589 (2015).

[17] Nishikata, T. & Nagashima, H. N alkylation of tosylamides using esters as primary and tertiary alkyl sources: mediated by hydrosilanes activated by a ruthenium catalyst. *Angew. Chem. Int. Ed.* **51**, 5363–5366 (2012).

[18] Tang, X., Huang, L., Qi, C., Wu, X., Wu, W. & Jiang, H. Copper-catalyzed sulfonamides formation from sodium sulfinates and amines. *Chem. Commun.* **49**, 6102–6104 (2013).

[19] Bielefeld, J. & Doye, S. Fast titanium-catalyzed hydroaminomethylation of alkenes and the formal conversion of methylamine. *Angew. Chem. Int. Ed.* **59**, 6138–6143 (2020).

[20] Li, L., Liu, Y.-C. & Shi, H. Nickel-catalyzed enantioselective α-alkenylation of *N*-sulfonyl amines: modular access to chiral α-branched amines. *J. Am. Chem. Soc*. **143**, 4154−4161 (2021).

[21] Greenhalgh, M. D., Frank, D. J. & Thomas, S. P. Iron-catalysed chemo-, regio-, and stereoselective hydrosilylation of alkenes and alkynes using a bench-stable iron(II) pre-catalyst. *Adv. Synth. Catal*. **356**, 584–590 (2014).

[22] Huang, C.-Y. & Doyle, A. G. Nickel-catalyzed Negishi alkylations of styrenyl aziridines. *J. Am. Chem. Soc.* **134**, 9541−9544 (2012).

[23] Jui, N. T., Lee, E. C. Y. & MacMillan, D. W. C. Enantioselective organo-SOMO cascade cycloadditions: a rapid approach to molecular complexity from simple aldehydes and olefins. *J. Am. Chem. Soc.* **132**, 10015–10017 (2010).

[24] Yu, S., Noble, A., Bedford, R. B. & Aggarwal, V. K. Methylenespiro[2.3]hexanes via nickel-catalyzed cyclopropanations with [1.1.1]propellane. *J. Am. Chem. Soc.* **141**, 20325−20334 (2019).

[25] Seo, H., Liu, A. & Jamison, T. F. Direct β-selective hydrocarboxylation of styrenes with CO_2 enabled by continuous flow photoredox catalysis. *J. Am. Chem. Soc.* **139**, 13969−13972 (2017).

[26] Rodriguez, J. G. & Benito, Y. Synthesis of 4-vinylquinoline: pyrolytic rearrangement of the 4-(1-hydroxyethyl)quinoline and related derivatives. *J. Heterocyclic Chem.* **25**, 819−821 (1988).

[27] Thompson, G. L., Heyd, W. E. & Paquette, L. A. Solvolytic studies of unsaturated 11-hydroxymethylbicyclo[4.4.1]undecane 3,5-dinitrobenzoates. Valence isomerization leading to conformationally distinguishable annulated norcaradienylcarbinyl cations and the question of remote p.pi. stabilization of such .sigma.-delocalized systems. *J. Am. Chem. Soc.* **96**, 3177–3190 (1974).

[28] Too, P. C., Chan, G. H., Tnay, Y. L., Hirao, H. & Chiba, S. Hydride reduction by a sodium hydride–iodide composite. *Angew. Chem. Int. Ed.* **55**, 3719–3723 (2016).

[29] Kato, N., Shirai, T. & Yamamoto, Y. Rhodium-catalyzed enantioselective arylation of aliphatic imines. *Chem. Eur. J.* **22**, 7739–7742 (2016).

[30] Padwa, A., Zanka, A., Cassidy, M. P. & Harris, J. M. An efficient synthesis of furyl sulfonamides from the reaction of furan with in situ generated *N*-tosyl imines. *Tetrahedron* **59**, 4939–4944 (2003).

[31] Wu, F., Ariyarathna, J. P., Kaur, N., Alom, N.-E., Kennell, M. L., Bassiouni, O. H. & Li, W. Halogen-bond-induced consecutive $C_{sp}{}^{3}$–H aminations via hydrogen atom transfer relay strategy. *Org. Lett*. **22**, 2135−2140 (2020).

[32] Nakai, K., Yoshida, Y., Kurahashi, T. & Matsubara, S. Nickel-catalyzed redox-economical coupling of alcohols and alkynes to form allylic alcohols. *J. Am. Chem. Soc*. **136**, 7797−7800 (2014).
